# Factors associated with oral health care behaviors of pregnant women in a northeastern province in Thailand: A hospital-based cross-sectional study

**DOI:** 10.1371/journal.pone.0290334

**Published:** 2023-08-31

**Authors:** Pimchanok Bunnatee, Fatima Ibrahim Abdulsalam, Nitikorn Phoosuwan

**Affiliations:** 1 Faculty of Public Health, Kasetsart University Chalermphrakiat Sakon Nakhon Province Campus, Sakon Nakhon, Thailand; 2 Buengkhonglong Hospital, Buengkan Province, Thailand; 3 Department of Public Health and Caring Sciences, Faculty of Medicine, Uppsala University, Uppsala, Sweden; University of the Witwatersrand Johannesburg Faculty of Health Sciences, SOUTH AFRICA

## Abstract

**Background:**

Oral healthcare behavior leads to oral health status. Factors associated with oral healthcare behavior might affect oral hygiene in pregnant women, who are at high risk for gingivitis and dental caries. This study aimed to explore factors associated with oral healthcare behaviors during pregnancy among pregnant women in a northeastern province of Thailand.

**Method:**

A total of 405 pregnant women who attended antenatal care clinics at one of the government hospitals in the province were invited to participate in this cross-sectional study. Dentists in the hospitals measured pregnant women’s gingivitis and dental calculus status using mouth mirrors and explorers. A structured questionnaire was used to obtain variables of interest. Linear regression analysis, Beta and 95% confidence interval (CI) were applied.

**Results:**

The majority were 20–24 years old (33.6%). Most of the participants had received upper secondary education (37.6%). Majority had gingivitis (88.1%) and dental calculus (88.6%). The findings revealed that age (Beta = -0.129, 95%CI = -0.269, -0.016), educational level (Beta = 0.118, 95% CI = 0.110, 0.183), and oral health literacy (Beta = 0.283, 95% CI = 0.156, 0.319) were statistically significant factors associated with oral healthcare behaviors.

**Conclusion:**

Younger pregnant women had better oral healthcare behaviors than older pregnant women and pregnant women had better oral healthcare behaviors due to higher educational levels and oral health literacy. Oral health promotion should be improved through oral health literacy, and interventions should be added to improve oral care skills particularly in older pregnant women as they are at a greater risk for poor oral healthcare behaviors.

## Introduction

The need for dental care during pregnancy recommended internationally has been followed by nations worldwide [1], as oral health is an important indicator of overall health, well-being, and life quality [2]. Pregnancy is a period of particular vulnerability because many physiological changes in the body of pregnant women can affect the oral cavity [3]. In addition, the gestation period presents both physiological and psychological changes that expose the oral cavity to pathologies that can affect the mother’s general health, such as hormonal changes that interfere with the oral cavity, aggravating pathologies like periodontal disease, gingivitis, and dental caries [1].

Women are at risk of oral health problems during pregnancy [4]. A high prevalence and severity of caries and periodontal disease were found to be significantly related to common oral symptoms reported by pregnant women in a study where a higher prevalence of dental caries and periodontal disease was reported in pregnant women compared to non-pregnant women [5]. Several consequences are related to pregnant women who have oral health issues, such as a higher risk of developing pre-eclampsia [6]. If oral health problems occur between the beginning of fertilization and about 12 weeks of pregnancy, women may be linked to miscarriage and preterm birth [7] which is a major public health problem in all countries [8]. In addition, children whose mothers or caregivers have untreated dental caries were more likely to have dental caries including early childhood caries, the most common chronic infectious childhood disease, and a major public health problem in the coming years [5]. Previous studies have shown a reduced oral health-related quality of life in pregnant women [3].

In Asia and the Pacific, approximately 85,000 women die annually during childbirth or pregnancy-related complications, and about 90% of these deaths could have been prevented through quality antenatal [9]. The average pregnant women mortality rate in Asia-Pacific is extremely high, compared to high-income countries [9]. Quality antenatal care is essential in preventing pregnant women’s mortality, especially in low- and lower-middle-income countries [10], and a part of quality antenatal care is oral health care for pregnant women [11].

Thailand has been classified as a middle-income country and there are many unresolved public health problems including oral health problems [12] which increase the risk of miscarriage [8]. The 2016 report of the national oral health survey in Thailand stated that more than 90% of pregnant women had gingivitis and tooth decay, and the average number of needed fillings or extractions was 6.37 teeth per person [13]. As part of indicators set for the development of the health service system in 2022, the Thailand Ministry of Public Health has included that not less than 30% of pregnant women should receive oral health examinations, and oral health promotion and prevention services should be provided to at least 70% of pregnant women. These indicators could determine the direction and strategic planning of the oral healthcare service system [14]. However, according to the dental services report for the strategic planning of oral health standards in Buengkan province, the prevalence of dental caries in pregnant women was 74% and only 22% of pregnant women received oral health checks and care [15]. This information shows that pregnant women lack care in terms of oral health.

Therefore, routine adequate and effective care is essential to maintain sustainable oral and dental healthcare for a lifetime [16]. There seems to be a lack of oral health information particularly for pregnant women [3]. Hence, this study aims to investigate factors associated with oral healthcare behaviors among pregnant women in Thailand. Findings could contribute to a greater understanding of the oral healthcare behaviors of pregnant women and the quality of care for prenatal oral health. These efforts could contribute to improving oral healthcare behaviors among other patient groups, with the ultimate goal of improving health across the life course.

## Methods

### Participants

This descriptive cross-sectional study was carried out in Buengkan, a northeastern province of Thailand. Buengkan province has a mixture of residential areas (i.e. rural, and semi-urban). It has eight government hospitals divided into two levels, namely a standard-level hospital and a first-level hospital. Approximately 1,200 pregnant women visit all eight hospitals (eight antenatal care clinics) annually [17].

The sample size was calculated using a formula [18] (Z_α/2_ = standard normal distribution curve critical value for 95% confidence interval (CI) = 1.96, d = the acceptable margin of error (precision) = 0.05, standard deviation (SD) = 0.49) [19]. The required number of the largest sample size was 368, 10% of the total sample size was added to compensate for the non-response rate and the final sample size was 450 people.

All hospitals in Buengkan were selected for this study because each hospital had its specific characteristics like being close to a border country or being far away from the others. Hence, randomization of the hospitals might not represent hospitals in Buengkan. The participants were selected using a consecutive sampling method from pregnant women who visited antenatal care clinics at the data collection time. The inclusion criteria for pregnant women: (1) were at least 18 years old, (2) attending antenatal care clinics at government hospitals in Bueng Kan province at the time of data collection, (3) had a gestation period between eight to twenty-six weeks based on data from their last menstrual period (LMP) or ultrasound history in the maternal and child diary (Pink Book) according to guidelines for providing dental public health services among pregnant women [17], (4) could communicate in Thai, and (5) provided informed consent to participate in the study. The exclusion criteria were pregnant women who: (1) required emergency care including illnesses that require hospitalization, surgery, or severe infections, and (2) reported a history of mental illness.

### Instrument

This study used a structured questionnaire to investigate factors associated with the oral healthcare behaviors of pregnant women. The researchers developed a structured questionnaire based on a literature review. The questionnaire consisted of three parts, and it had been tested for content validity by four experts with the Content Validity Index (CVI) = 0.99 for the entire questionnaire. The reliability test for the questionnaire was conducted among 30 pregnant women in a province near Buengkan province who were not included in the study, and the overall Cronbach’s alpha coefficient = 0.88. The intra-examiner calibration of the oral examiners (eight dentists from eight selected hospitals) was highly satisfactory (Kappa value ≥0.80), while the calibration of the instrument was annually checked by the hospital staff.

Part I: Personal factors (16 items) gathered information about age, education, employment, marital status, family type, personal income per month, personal expenses per month, health insurance, obstetric history (i.e., gravida, parity, abortion, and living status), dental service history, and oral parameters (dental caries status, gingivitis status, and dental calculus status). A dentist in each hospital evaluated dental caries status, gingivitis status and dental calculus status. The dental caries status was evaluated, while mouth mirror and explorer were utilized. For the dental caries status, coded “No” meant having no dental caries and “Yes” meant having dental caries. Gingivitis status had two options where “No” meant having normal gingival and “Yes” meant having gingivitis (pain, swelling, redness, and heat of the gingival). For dental calculus status, “No” meant having no dental calculus and “Yes” meant having dental calculus. The coding “Yes” or “No” followed the guideline from the Ministry of Public Health, Thailand [17].

Part II: Oral health literacy part (14 items) referenced by the Thai version of the Health Literacy in Dentistry scale (HeLD-Th) 2019 [20]. It had seven dimensions: (1) receptivity, (2) understanding, (3) support (4) economic barriers, (5) access, (6) communication, (7) utilization, where each dimension had two questions and five options to evaluate frequency (viz. 0 = never done, 1 = can do, 2 = sometimes possible, 3 = frequently done, and 4 = do it regularly), each question provided a score of one if answered correct or zero if answered incorrectly. Therefore, the score of this part ranged between 0–56.

Part III: Oral healthcare behaviors (15 items) was assessed to the frequency of oral healthcare practice (nine questions), food consumption behavior (five questions), and medication behavior (one question), where each question had four options to evaluate frequency to actions (viz. 3 = always, 2 = often, 1 = sometimes, 0 = never). Therefore, each question ranged between 0–3; scores for the entire questionnaire ranged between 0–45.

### Ethical approval

The study was approved by ethical review boards in Thailand (CSC.KUREC-65/013) and was conducted in compliance with the ethical principles of the Declaration of Helsinki [21]. Permission to conduct this study was sought from the director of the government hospitals in Buengkan province. In addition, oral examination was done as a part of routine check-ups as indicated in the guideline for pregnant women from the Ministry of Public Health, Thailand [17]. All participants received written and oral information before signing a written consent form. The information emphasized the freedom of partaking in the study or not and that participants were fully entitled to withdraw at any time.

### Data collection

On average, approximately 20 to 30 patients per week attend antenatal care at each hospital. The researchers received a list of pregnant women from midwives at the antenatal care clinic in the hospital. Thereafter, midwives referred pregnant women to be screened for oral health status at dental clinics by dentists and asked pregnant women if they wanted to participate voluntarily and answer questionnaires. Oral health status was determined according to the Department of Health guidelines for pregnant women [17]. The pregnant women were invited to sit in a dental unit and a dentist in each hospital adjusted the unit and the dental light according to their convenience, and visibility to evaluate oral parameters for oral examination. The dentist used a mouth mirror to check for these parameters. The dentist coded the parameters and recorded them in the participants’ Pink book. Thereafter, each participant self-administered the questionnaire for about 15 to 20 minutes in a room at the hospital. The questionnaires were collected immediately upon completion. Data was collected from April to September 2022. In total, 405/411 (98.54%) pregnant women participated in this study.

### Statistical analysis

All data were entered and analyzed using a software program (Statistical Program for Social Science). The data variables were expressed by the mean and standard deviation (SD) (viz. age, personal income per month, personal expenses per month, and oral healthcare behaviours). The variables were described in frequency and percentage (%): educational level, employment status, marital status, family type, gravida (G), parity (P), abortion (A), living (L) status, dental treatment history, and oral parameters. A binary regression analysis was performed to explore the association between independent variables and oral healthcare behavior. An analysis of multivariable linear regression was used to predict factors associated with oral healthcare behavior.

The following independent variables were tested: age group, educational level, employment status, marital status, family type, personal income per month (Baht), personal expenses per month (Baht), G, P, A, L, and dental treatment history. Marital status (0 = not live together, 1 = live together), family type (0 = nuclear family, 1 = extended family), G (0 = primi gravida, 1 = multi gravida), A (0 = no, 1 = yes), L (0 = no child, 1 = one child or more), and dental treatment history (0 = no history, 1 = have history) were considered as dummy variables. In multivariable regression analysis, variables were selected using enter method. The importance of each selected variable was verified following the fit of assumption model, containing the test of normality dependent variable (Z_skewness_ = 0.94, Z_Kurtosis_ = 1.98), the test of the multicollinearity problems, and the Variance Inflation Factor (VIF) respectively. The final model was assessed for fitness using the significance level set at 0.05.

## Results

A total of 405 pregnant women participated in the study. The ages of the participants ranged from 18 to 43 years (mean 26.3 years; standard deviation 5.8). The majority of the participants were educated at the upper secondary level (n = 150), and more than half (52.6%) were farmers. For most women (51.6%), it was their first pregnancy, 52.8% had nulliparous status, 92.3% have never had an abortion, and 52.6% of them did not have children. More than half (53.6%) did not have regular dental care in the past six months, gingivitis (8.1%) and dental calculus (88.6%). See Table 1.

**Table 1:** Characteristics of pregnant women (n = 405).

Characteristics		Frequency (%)
Age group (years)		
	< 20	49 (12.1)
	20–24	136 (33.6)
	25–30	92 (22.7)
	31–35	88 (21.7)
	≥ 35	40 (9.9)
	Mean±SD (Range) = 26.3 ± 5.8 (18.0–43.0)	
Educational level		
	Primary	30 (7.4)
	Lower Secondary	79 (19.5)
	Upper Secondary or equivalent	150 (37.0)
	Post-secondary	70 (17.3)
	Tertiary	76 (18.8)
Employment		
	Farmers / Employees / Business owners	213 (52.6)
	Unemployed/ Housewife	135 (33.3)
	Government officers/ State enterprises/ Private employees	57 (14.1)
Marital status		
	Live together	377 (93.1)
	Not together	28 (6.9)
Family type		
	Nuclear family	293 (72.3)
	Extended family	112 (27.7)
Personal income per month (n = 384)		
	< 10,000 Baht	144 (35.6)
	10,000–19,999 Baht	197 (48.6)
	20,000–29,999 Baht	25 (6.2)
	≥ 30,000 Baht	25 (6.2)
	Mean±SD (Range) 17,700.9 ± 45,633.0 (1,200–608,800)	
Personal expenses per month (n = 382)		
	< 10,000 Baht	214 (52.8)
	10,000–19,999 Baht	151 (37.3)
	20,000–29,999 Baht	7 (1.7)
	≥ 30,000 Baht	10 (2.5)
	Mean±SD (Range) 10,434.6 ± 9,712.1 (500–89,500)	
Health Insurance		
	Universal Coverage	309 (76.3)
	Social Security Scheme	62 (15.3)
	Government / State Enterprise Officer / Local Government Officer Scheme	27 (6.7)
	Payment	7 (1.7)
Gravida status		
	Primigravida	209 (51.6)
	Multigravida	196 (48.4)
Parity		
	Nulliparous	214 (52.8)
	Primiparous	126 (31.1)
	Multiparous	65 (16.0)
Abortion		
	No	374 (92.3)
	Yes	31 (7.7)
Living		
	No child	213 (52.6)
	≥ 1	192 (47.4)
Dental treatment history (at least 6 months)		
	Yes (Filling/Scaling/Extraction/Surgical removal of the wisdom tooth)	217 (53.6)
	No	188 (46.4)
Dental caries status		
	No	207 (51.1)
	Yes	198 (48.9)
Gingivitis status		
	Yes	357 (88.1)
	No	48 (11.9)
Dental calculus status		
	Yes	359 (88.6)
	No	46 (11.4)

The majority of pregnant women were found to be able to seek advice from a dentist to make informed decisions about their dental health (mean = 2.95, SD = 0.99), followed by being able to make time for things that are good for dental or oral health (mean = 2.93, SD = 0.95). See Table 2.

**Table 2:** The oral health literacy among pregnant women. (n = 405).

Oral health literacy domains	Oral health literacy items	Mean Score (SD)
Receptivity (R)	(R1) Are you able to pay attention to your dental or oral health needs?	2.61 (1.03)
	(R2) Are you able to make time for things that are good for your dental or oral health?	2.93 (0.95)
Understanding (U)	(U1) Are you able to fill out dental forms such as the enrollment form?	2.67 (1.13)
	(U2) Are you able to read dental or oral health information brochures left in dental clinics and waiting rooms?	2.80 (1.15)
Support (S)	(S1) Are you able to take a family member or friend with you to a dental appointment?	2.82 (1.19)
	(S2) Are you able to ask a family member or friend for help to understand dental or oral health information?	2.88 (1.09)
Economic barriers (E)	(E1) Are you able to pay to see a dentist?	2.60 (1.21)
	(E2) Are you able to afford transportation to the dental clinic?	2.85 (1.11)
Access (A)	(A1) Do you know where a dentist can be contacted?	2.82 (1.09)
	(A2) Do you know what to do to get a dentist’s appointment?	2.80 (1.10)
Communication (C)	(C1) Are you able to look for a second opinion about your dental health from a dental health professional?	2.56 (1.06)
	(C2) Are you able to use information from a dentist to make decisions about your dental health?	2.75 (1.00)
Utilization (X)	(X1) Are you able to carry out instructions that a dentist gives you?	2.75 (1.00)
	(X2) Are you able to use advice from a dentist to make decisions about your dental health?	2.95 (0.99)

Note: SD, standard deviation

The findings revealed that age (Beta = -0.129, 95% CI = -0.269, -0.016), educational level (Beta = 0.118, 95% CI = 0.110, 1.183), and oral health literacy (Beta = 0.283, 95% CI = 0.156, 0.319) were statistically significant factors associated with oral healthcare behaviors of pregnant women. The variables were able to predict 12.5% of oral healthcare behavior during pregnancy (R^2^ = 0.125). See Table 3.

**Table 3:** Factors associated with oral healthcare behaviors among pregnant women by multivariable linear regression analysis. (n = 405).

Variables	Unstandardized Coefficients		Standardized Coefficients	t-value	p-value	95% Confidence for Interval for B	
	b	Std. Error	Beta			Lower Bound	Upper Bound
Age	-0.143	0.064	-0.129	-2.220	0.027*	-0.269	-0.016
Educational level	0.646	0.273	0.118	2.369	0.018*	0.110	1.183
Oral health literacy	0.157	0.027	0.283	5.799	<0.001*	0.103	0.210
(Constant)	21.557	1.890	-	11.407	<0.001	17.842	25.273
R = .354 R ^2^ = .125 F = 9.512							

*Correlation was significant at the p-value < 0.05

## Discussion

This explorative study aimed to describe factors associated with oral healthcare behaviors during pregnancy among 405 participants. It was found that about 88% of the participants had gingivitis during pregnancy. This number is quite similar to another study showing that 72% of Indian pregnant women have gingivitis [22]. It might be that the current study and the Indian study collected data among pregnant women who have low education and are daily farmers/employers. In order to reduce gingivitis among pregnant women, attention needs to be centered on vulnerable pregnant women, e.g. those in remote areas or those with a low level of education.

About 48% of the pregnant women in this study had dental caries. Pregnant women might suffer from dental caries more than non-pregnant women [22]. However, one study in central India shows that 63% of pregnant women have dental caries [22]. It could be because more than half of the pregnant women in this study received dental treatment within six months of pregnancy. This led to a low percentage of dental caries.

Findings also revealed significance with age, educational level, and oral health literacy. These findings are similar to other research that revealed several factors that cause dental caries including internal factors (i.e., age, behaviors, food behavior, teeth, saliva, plaque) and social factors such as wealth and education [23]. Therefore, older pregnant women should be taken into consideration as they are at greater risk for poor oral healthcare behaviors [23]. Oral health education before pregnancy and awareness of the relationship between poor maternal oral health and adverse pregnancy outcomes need to be implemented by healthcare professionals [24].

In this study, pregnant women had better oral healthcare behaviors at higher educational levels. Another study suggests that pregnant women with primary education had significantly greater odds of good knowledge during pregnancy compared to those with no education [2]. This shows that higher education level plays a role in better oral healthcare behaviors. Educational status, monthly income, occupation, access to health services, and receiving counseling on oral hygiene at antenatal care were some factors associated with good knowledge of oral health during pregnancy [2, 24]. Therefore strengthening counseling during antenatal care, improving access to a health facility, and improving educational status, monthly income, and career are crucial to enhancing the knowledge of women towards oral health during pregnancy.

This study found that oral health literacy was significant in the oral healthcare behavior of pregnant women. These findings are similar to other research that women with adequate oral health literacy were more likely to be those with higher education qualifications and were very satisfied or satisfied with their oral health status [5]. Besides, the participants who have poor oral hygiene and low-skill patient-provider communication bring inadequate oral health literacy. These findings suggest a lack of health literacy as individuals with limited oral health literacy levels may have poorer periodontal health [25]. This study also found that oral health literacy is related to enhancing oral health care, as health literacy is a key determinant influencing oral health behaviors and outcomes. These showed that low oral health literacy and inadequate patient-provider communication perpetuate poor oral health knowledge, attitudes, and practices during pregnancy [26]. Hence, a comprehensive understanding of women’s oral health literacy experiences and needs is essential in developing proper dental healthcare interventions that facilitate positive oral health hygiene and care-seeking practices during pregnancy [22]. Improving the oral health literacy of patients may help in the efforts to improve adherence to medical instructions, self-management skills, and overall pregnancy outcomes.

In addition to revealing factors associated with oral healthcare behaviors, this study also collected data on the oral health status of pregnant women, which found that more than half (53.6%) did not have regular dental care in the past six months, had gingivitis (8.1%), and dental calculus (88.6%). Previous studies have found that the majority of the mothers claimed that their oral health status was good, but only 29% of the mothers visited the dentist during the current pregnancy [2, 27]. Common excuses given by most mothers as to why they have had no dental visitations include perceptions of not having any oral health problems (65.9%), long waiting time at the clinic (71.6%), and no immediate treatment given by the dentist (64.8%) [26]. There was also a study that found that pregnant women with no history of oral problems, with a perception of medium or high income, and with good oral hygiene behaviors tend to have a good perception of their oral health [1]. However, mothers who reported dental visits were more likely to be those who had received oral health education before the current pregnancy and knew of the association between poor maternal oral health and adverse pregnancy outcomes. Dissatisfaction with the services rendered and perceptions of not having any oral health problems were the main barriers [27, 28]. On the contrary, our study did not find such oral health factors associated with oral healthcare behaviors.

### Strengths and limitations

The strength of this study is that the sample size is complete and representative of Bueng Kan province. The consecutive sampling method was appropriate because the study was aimed at investigation among pregnant women within 26 weeks of gestation due to guidelines for oral healthcare among pregnant women in Thailand [17]. In addition, a high response rate was also presented.

The findings from this study must be considered within the noted limitations that although Bueng Kan is one of the provinces in the Northeast, it cannot represent all other provinces in this region or another context. Moreover, because this study used a cross-sectional design method, the data is specific and might not be certain or representative of other future studies. Further case-control or cohort studies may be conducted in different contexts to obtain more certainty. Some biases might happen in this study, e.g. self-answering leads to recall biases and short data collection time provides selection bias.

## Conclusions

The younger pregnant women had better oral healthcare behaviors than the older pregnant women. While pregnant women had better oral healthcare behaviors due to higher educational levels and oral health literacy, older pregnant women should be taken into consideration as they are at greater risk for poor oral healthcare behaviors. Oral health promotion should be improved through oral health literacy, and interventions should be added to improve oral care skills. The findings have important implications for strategies aimed at improving the oral health status of pregnant women and the general population. Besides tailoring factors associated with oral healthcare behaviors to suit pregnant women, the future plan should also integrate oral health literacy into the school curriculum of children to enable them to become oral health-literate adults in the long term.

## Supporting information

S1 Checklist STROBE statement—checklist of items that should be included in reports of observational studies. (DOCX)

S1 File (PDF)
