# Supplementary material for: Factors associated with oral health care behaviors of pregnant women in a northeastern province in Thailand: A hospital-based cross-sectional study
Source: PLoS One. 2023 Aug 31;18(8):e0290334. doi: 10.1371/journal.pone.0290334 (PMC10470968; doi:10.1371/journal.pone.0290334)
Supplement: S1 Checklist — (DOCX) [file pone.0290334.s001.docx]

STROBE Statement—checklist of items that should be included in reports of observational studies

|  | Item No. | Recommendation | Page  No. | Relevant text from manuscript |
| --- | --- | --- | --- | --- |
| **Title and abstract** | 1 | (*a*) Indicate the study’s design with a commonly used term in the title or the abstract | 2 | A total of 405 pregnant women who attended antenatal care clinics at one of the government hospitals in the province were invited to participate in this cross-sectional study. |
|  |  | (*b*) Provide in the abstract an informative and balanced summary of what was done and what was found | 2 | Younger pregnant women had better oral healthcare behaviors than older pregnant women and pregnant women had better oral healthcare behaviors due to higher educational levels and oral health literacy. |
| Introduction | | | |  |
| Background/rationale | 2 | Explain the scientific background and rationale for the investigation being reported | 3-4 | - Women are at risk of oral health problems during pregnancy [4]. A high prevalence and severity of caries and periodontal disease were found to be significantly related to common oral symptoms reported by pregnant women in a study where a higher prevalence of dental caries and periodontal disease was reported in pregnant women compared to non-pregnant women [5].  - However, according to the dental services report for the strategic planning of oral health standards in Buengkan province, the prevalence of dental caries in pregnant women was 74% and only 22% of pregnant women received oral health checks and care [15]. This information shows that pregnant women lack care in terms of oral health. |
| Objectives | 3 | State specific objectives, including any prespecified hypotheses | 4 | Hence, this study aims to investigate factors associated with oral healthcare behaviors among pregnant women in Thailand. Findings could contribute to a greater understanding of the oral healthcare behaviors of pregnant women and the quality of care for prenatal oral health. |
| Methods | | | |  |
| Study design | 4 | Present key elements of study design early in the paper | 5 | This descriptive cross-sectional study was carried out in Buengkan, a northeastern province of Thailand. - |
| Setting | 5 | Describe the setting, locations, and relevant dates, including periods of recruitment, exposure, follow-up, and data collection | 5-8 | - This descriptive cross-sectional study was carried out in Buengkan, a northeastern province of Thailand.  - The sample size was calculated using a formula [18]  - the final sample size was 450 people.  - All hospitals in Buengkan were selected for this study because each hospital had its specific characteristics like being close to a border country or being far away from the others. Hence, randomization of the hospitals might not represent hospitals in Buengkan. The participants were selected using a consecutive sampling method from pregnant women who visited antenatal care clinics at the data collection time.  - This study used a structured questionnaire to investigate factors associated with the oral healthcare behaviors of pregnant women.  - The study was approved by ethical review boards in Thailand (CSC.KUREC-65/013) and was conducted in compliance with the ethical principles of the Declaration of Helsinki [21].  - The researchers received a list of pregnant women from midwives at the antenatal care clinic in the hospital. Thereafter, midwives referred pregnant women to be screened for oral health status at dental clinics by dentists and asked pregnant women if they wanted to participate voluntarily and answer questionnaires.  - All data were entered and analyzed using a software program. The data variables were expressed by the mean and standard deviation (SD) and the variables were described in frequency and percentage (%). A binary regression analysis was performed to explore the association between independent variables and oral healthcare behavior. An analysis of multivariable linear regression was used to predict factors associated with oral healthcare behavior. |
| Participants | 6 | (*a*) *Cohort study*—Give the eligibility criteria, and the sources and methods of selection of participants. Describe methods of follow-up  *Case-control study*—Give the eligibility criteria, and the sources and methods of case ascertainment and control selection. Give the rationale for the choice of cases and controls  *Cross-sectional study*—Give the eligibility criteria, and the sources and methods of selection of participants | 5 | The inclusion criteria for pregnant women: (1) were at least 18 years old, (2) attending antenatal care clinics at government hospitals in Bueng Kan province at the time of data collection, (3) had a gestation period between eight to twenty-six weeks based on data from their last menstrual period (LMP) or ultrasound history in the maternal and child diary (Pink Book) according to guidelines for providing dental public health services among pregnant women [17], (4) could communicate in Thai, and (5) provided informed consent to participate in the study. |
|  |  | (*b*) *Cohort study*—For matched studies, give matching criteria and number of exposed and unexposed  *Case-control study*—For matched studies, give matching criteria and the number of controls per case | 5 | The exclusion criteria were pregnant women who: (1) required emergency care including illnesses that require hospitalization, surgery, or severe infections, and (2) reported a history of mental illness. |
| Variables | 7 | Clearly define all outcomes, exposures, predictors, potential confounders, and effect modifiers. Give diagnostic criteria, if applicable | 7-8 | In multivariable regression analysis, variables were selected using enter method. The importance of each selected variable was verified following the fit of assumption model, containing the test of normality dependent variable (Zskewness = 0.94, ZKurtosis = 1.98), the test of the multicollinearity problems, and the Variance Inflation Factor (VIF) respectively. |
| Data sources/ measurement | 8* | For each variable of interest, give sources of data and details of methods of assessment (measurement). Describe comparability of assessment methods if there is more than one group | *5*  *8-9* | - All hospitals in Buengkan were selected for this study because each hospital had its specific characteristics like being close to a border country or being far away from the others. Hence, randomization of the hospitals might not represent hospitals in Buengkan. The participants were selected using a consecutive sampling method from pregnant women who visited antenatal care clinics at the data collection time.  - All data were entered and analyzed using a software program (Statistical Program for Social Science). The data variables were expressed by the mean and standard deviation (SD) (viz. age, personal income per month, personal expenses per month, and oral healthcare behaviours). The variables were described in frequency and percentage (%): educational level, employment status, marital status, family type, gravida (G), parity (P), abortion (A), living (L) status, dental treatment history, and oral parameters. A binary regression analysis was performed to explore the association between independent variables and oral healthcare behavior. An analysis of multivariable linear regression was used to predict factors associated with oral healthcare behavior.  - All relevant data are within the manuscript and its Supporting  Information files |
| Bias | 9 | Describe any efforts to address potential sources of bias | 13 | Some biases might happen in this study, e.g. self-answering leads to recall biases and short data collection time provides selection bias. |
| Study size | 10 | Explain how the study size was arrived at | 5 | The sample size was calculated using a formula [18] (Zalpha/2 = standard normal distribution curve critical value for 95% CI = 1.96, d = the acceptable margin of error (precision) = 0.05, standard deviation (SD) = 0.49) [19]. The required number of the largest sample size was 368, 10% of the total sample size was added to compensate for the non-response rate and the final sample size was 450 people. |

Continued on next page

| Quantitative variables | 11 | Explain how quantitative variables were handled in the analyses. If applicable, describe which groupings were chosen and why | 5  7 | The participants were selected using a consecutive sampling method.  The researchers received a list of pregnant women from midwives at the antenatal care clinic in the hospital. Thereafter, midwives referred pregnant women to be screened for oral health status at dental clinics by dentists and asked pregnant women if they wanted to participate voluntarily and answer questionnaires. |
| --- | --- | --- | --- | --- |
| Statistical methods | 12 | (*a*) Describe all statistical methods, including those used to control for confounding | 7 | All data were entered and analyzed using a software program. The data variables were expressed by the mean and standard deviation (SD) and the variables were described in frequency and percentage (%). A binary regression analysis was performed to explore the association between independent variables and oral healthcare behavior. An analysis of multivariable linear regression was used to predict factors associated with oral healthcare behavior. |
|  |  | (*b*) Describe any methods used to examine subgroups and interactions | 9 | An analysis of multivariable linear regression was used to predict factors associated with oral healthcare behavior. |
|  |  | (*c*) Explain how missing data were addressed | 8 | In total, 405/411 (98.54%) pregnant women participated in this study. |
|  |  | (*d*) *Cohort study*—If applicable, explain how loss to follow-up was addressed  *Case-control study*—If applicable, explain how matching of cases and controls was addressed  *Cross-sectional study*—If applicable, describe analytical methods taking account of sampling strategy | 8 | In total, 405/411 (98.54%) pregnant women participated in this study. |
|  |  | (*e*) Describe any sensitivity analyses | 6 | The questionnaire consisted of three parts, and it had been tested for content validity by four experts with the Content Validity Index (CVI) = 0.99 for the entire questionnaire. The reliability test for the questionnaire was conducted among 30 pregnant women in a province near Buengkan province who were not included in the study, and the overall Cronbach’s alpha coefficient = 0.88. The intra-examiner calibration of the oral examiners (eight dentists from eight selected hospitals) was highly satisfactory (Kappa value ≥0.80), while the calibration of the instrument was annually checked by the hospital staff. |
| Results | | | | |
| Participants | 13* | (a) Report numbers of individuals at each stage of study—eg numbers potentially eligible, examined for eligibility, confirmed eligible, included in the study, completing follow-up, and analysed | 8 | A total of 405 pregnant women participated in the study. |
|  |  | (b) Give reasons for non-participation at each stage | 8 | In total, 405/411 (98.54%) pregnant women participated in this study. |
|  |  | (c) Consider use of a flow diagram | Not applicable | Not applicable |
| Descriptive data | 14* | (a) Give characteristics of study participants (eg demographic, clinical, social) and information on exposures and potential confounders | 8 | The ages of the participants ranged from 20 to 24 years (mean 26.3 years; standard deviation 5.8) with the youngest being 18 and the oldest being 43. The majority of the participants were educated at the upper secondary level (n = 150), and more than half (52.6 %) were farmers. For most women (51.6%), it was their first pregnancy, 52.8% had nulliparous status, 92.3% have never had an abortion, and 52.6% of them did not have children. More than half (53.6 %) did not have regular dental care in the past six months, gingivitis (8.1%) and dental calculus (88.6%). |
|  |  | (b) Indicate number of participants with missing data for each variable of interest | Table 1 | Table 1 presents numbers of participants answered each variable of interest, e.g. Personal expenses per month (n=382) |
|  |  | (c) *Cohort study*—Summarise follow-up time (eg, average and total amount) | Not applicable | Not applicable |
| Outcome data | 15* | *Cohort study*—Report numbers of outcome events or summary measures over time | Not applicable | Not applicable- |
|  |  | *Case-control study—*Report numbers in each exposure category, or summary measures of exposure | Not applicable | Not applicable- |
|  |  | *Cross-sectional study—*Report numbers of outcome events or summary measures | 8  9 | The majority of pregnant women were found to be able to seek advice from a dentist to make informed decisions about their dental health (mean = 2.95, SD = 0.99), followed by being able to make time for things that are good for dental or oral health (mean = 2.93, SD = 0.95).  The findings revealed that age (Beta = -0.129, 95% CI = -0.269, -0.016), educational level (Beta = 0.118, 95% CI = 0.110, 1.183), and oral health literacy (Beta = 0.283, 95% CI = -0.103, 0.210) were statistically significant factors associated with oral healthcare behaviors of pregnant women. |
| Main results | 16 | (*a*) Give unadjusted estimates and, if applicable, confounder-adjusted estimates and their precision (eg, 95% confidence interval). Make clear which confounders were adjusted for and why they were included | 8 | A binary regression analysis was performed to explore the association between independent variables and oral healthcare behavior. An analysis of multivariable linear regression was used to predict factors associated with oral healthcare behavior. |
|  |  | (*b*) Report category boundaries when continuous variables were categorized | 9 | Marital status (0=not live together, 1=live together), family type (0=nuclear family, 1=extended family), G (0=primi gravida, 1=multi gravida), A (0=no, 1=yes), L (0=no child, 1=one child or more), and dental treatment history (0=no history, 1=have history) were considered as dummy variables. |
|  |  | (*c*) If relevant, consider translating estimates of relative risk into absolute risk for a meaningful time period | Not applicable | -Not applicable |

Continued on next page

| Other analyses | 17 | Report other analyses done—eg analyses of subgroups and interactions, and sensitivity analyses | Table 2 | Table 2 The oral health literacy domains among pregnant women (n = 405) |
| --- | --- | --- | --- | --- |
| Discussion | | | | |
| Key results | 18 | Summarise key results with reference to study objectives | 9-11 | -This explorative study aimed to describe factors associated with oral healthcare behaviors during pregnancy among 405 participants. Findings revealed significance with age, educational level, and oral health literacy. These findings are similar to other research that revealed several factors that cause dental caries including internal factors (i.e., age, behaviors, food behavior, teeth, saliva, plaque) and social factors such as wealth and education [22].  -In this study, pregnant women had better oral healthcare behaviors at higher educational levels. same as these findings, pregnant mothers with a primary educational status were 43% relative to women with no education, women with primary education had significantly greater odds of good knowledge during pregnancy [2].  - This study found that oral health literacy was significant with the oral healthcare behavior of pregnant women. These findings are similar to other research that women with adequate oral health literacy were more likely to be those with higher education qualifications and were very satisfied or satisfied with their oral health status [5]. Besides, the participants who have poor oral hygiene and low-skill patient-provider communication bring inadequate oral health literacy. These findings suggest a lack of health literacy as individuals with limited oral health literacy levels may have poorer periodontal health [23].  -However, mothers who reported dental visits were more likely to be those who had received oral health education before the current pregnancy and knew of the association between poor maternal oral health and adverse pregnancy outcomes. Dissatisfaction with the services rendered and perceptions of not having any oral health problems were the main barriers [25, 26]. On the contrary, our study did not find such oral health factors associated with oral healthcare behaviors. |
| Limitations | 19 | Discuss limitations of the study, taking into account sources of potential bias or imprecision. Discuss both direction and magnitude of any potential bias | 11 | However, the findings from this study must be considered within the noted limitations that although Bueng Kan is one of the provinces in the Northeast, it cannot represent all other provinces in this region. Therefore, the result of this study couldn't reference every pregnant woman in another context. Moreover, because this study used a cross-sectional design method, the data is specific and might not be certain or representative of other future studies. |
| Interpretation | 20 | Give a cautious overall interpretation of results considering objectives, limitations, multiplicity of analyses, results from similar studies, and other relevant evidence | 11 | Further case-control or cohort studies may be conducted in different contexts to obtain more certainty. Also, studies related to the difference between pregnant women regarding socio-demographics, access to medical and dental care, and parity status in the development and testing of prenatal oral health promotion interventions to maximize the acceptability of participants’ oral health literacy needs, and preferences remain to be explored. |
| Generalisability | 21 | Discuss the generalisability (external validity) of the study results | 12 | The younger pregnant women had better oral healthcare behaviors than the older pregnant women. While pregnant women had better oral healthcare behaviors due to higher educational levels and oral health literacy, older pregnant women should be taken into consideration as they are at greater risk for poor oral healthcare behaviors. Oral health promotion should be improved through oral health literacy, and interventions should be added to improve oral care skills. |
| Other information | |  | | |
| Funding | 22 | Give the source of funding and the role of the funders for the present study and, if applicable, for the original study on which the present article is based | 14 | The authors thank all respondents who participated in this study, the Faculty of Public Health Kasetsart University for supporting resources and materials, and the government hospital in Buengkan, Thailand. |

*Give information separately for cases and controls in case-control studies and, if applicable, for exposed and unexposed groups in cohort and cross-sectional studies.

**Note:** An Explanation and Elaboration article discusses each checklist item and gives methodological background and published examples of transparent reporting. The STROBE checklist is best used in conjunction with this article (freely available on the Web sites of PLoS Medicine at http://www.plosmedicine.org/, Annals of Internal Medicine at http://www.annals.org/, and Epidemiology at http://www.epidem.com/). Information on the STROBE Initiative is available at www.strobe-statement.org.
