# Supplementary material for: Factors associated with oral health care behaviors of pregnant women in a northeastern province in Thailand: A hospital-based cross-sectional study
Source: PLoS One. 2023 Aug 31;18(8):e0290334. doi: 10.1371/journal.pone.0290334 (PMC10470968; doi:10.1371/journal.pone.0290334)
Supplement: S1 File — (PDF) [file pone.0290334.s002.pdf]

id	age	edu	edu_other	อาชีพ	สถานภาพส	รายได้	รายจ่าย	ความพอเพี
1	20	ชั้นมัธยมตอนปลาย		ว่างงาน	แต่งงานอยู่	7000	7000	พอใช้ไม่เห
2	22	ชั้นมัธยมตอนปลาย		ว่างงาน	ไม่ได้แต่งง	9000	9000	พอใช้ไม่เห
3	22	ชั้นมัธยมศึกษาตอนต้น		ว่างงาน	ไม่ได้แต่งง	8000	8000	พอใช้ไม่เห
4	28	ระดับปริญญาตรี		รับราชการ/	แต่งงานแต่	30000	20000	พอใช้และเ
5	35	ระดับปริญญาตรี		ว่างงาน	แต่งงานอยู่	4500	4500	พอใช้ไม่เห
6	22	ชั้นมัธยมศึกษาตอนต้น		เกษตรกร	ไม่ได้แต่งง	8500	8500	พอใช้ไม่เห
7	21	ชั้นมัธยมตอนปลาย		รับจ้างรายวั	แต่งงานอยู่	6000	6000	พอใช้ไม่เห
8	27	ชั้นมัธยมตอนปลาย		รับราชการ/	แต่งงานอยู่	9300	4500	พอใช้ไม่เห
9	19	ประกาศนียบัตรวิชาชีพ		ว่างงาน	แต่งงานอยู่	5000	10000	ไม่พอใช้
10	18	ชั้นประถมศึกษา		ค้าขาย/ธุรกิ	แต่งงานอยู่	5000	4000	พอใช้และเ
11	31	ชั้นมัธยมศึกษาตอนต้น		ค้าขาย/ธุรกิ	ไม่ได้แต่งง	1200	1200	พอใช้ไม่เห
12	41	ชั้นมัธยมศึกษาตอนต้น		ว่างงาน	ไม่ได้แต่งง	20000	10000	พอใช้ไม่เห
13	20	ชั้นประถมศึกษา		รับราชการ/	ไม่ได้แต่งง	10000	3000	พอใช้ไม่เห
14	20	ชั้นมัธยมตอนปลาย		ว่างงาน	ไม่ได้แต่งง	10000	7000	พอใช้และเ
15	21	ชั้นมัธยมตอนปลาย		ค้าขาย/ธุรกิ	ไม่ได้แต่งง	6000	6000	พอใช้ไม่เห
16	25	ชั้นมัธยมตอนปลาย		ว่างงาน	แต่งงานอยู่	7000	5000	พอใช้ไม่เห
17	22	ชั้นมัธยมศึกษาตอนต้น		รับจ้างรายวั	ไม่ได้แต่งง	10000	80000	พอใช้ไม่เห
18	20	ชั้นมัธยมตอนปลาย		เกษตรกร	แต่งงานอยู่	15000	10000	พอใช้และเ
19	29	ระดับปริญญาตรี		ค้าขาย/ธุรกิ	แต่งงานอยู่	18000	16000	ไม่พอใช้
20	34	ระดับปริญญาตรี		รับราชการ/	แต่งงานแต่	20450	20000	พอใช้ไม่เห
21	23	ชั้นมัธยมตอนปลาย		รับราชการ/	ไม่ได้แต่งง	14800	12000	ไม่พอใช้
22	18	ชั้นมัธยมศึกษาตอนต้น		ว่างงาน	แต่งงานอยู่	7500	7000	พอใช้ไม่เห
23	29	ระดับปริญญาตรี		งานบริษัทเ	แต่งงานอยู่	29000	15000	พอใช้ไม่เห
24	24	ชั้นประถมศึกษา		ว่างงาน	ไม่ได้แต่งง	5000	5000	พอใช้ไม่เห
25	25	ชั้นมัธยมตอนปลาย		ค้าขาย/ธุรกิ	แต่งงานอยู่	15000	9000	พอใช้ไม่เห
26	31	ประกาศนียบัตรวิชาชีพ		ว่างงาน	ไม่ได้แต่งง	10000	10000	พอใช้ไม่เห
27	18	ประกาศนียบัตรวิชาชีพ		ว่างงาน	ไม่ได้แต่งง	5000	45000	พอใช้ไม่เห
28	18	ชั้นมัธยมตอนปลาย		ว่างงาน	ไม่ได้แต่งง	7000	5000	พอใช้และเ
29	18	ชั้นมัธยมตอนปลาย		ว่างงาน	ไม่ได้แต่งง	10000	8000	พอใช้และเ
30	25	ชั้นมัธยมตอนปลาย		รับจ้างรายวั	แต่งงานอยู่	9000	8000	พอใช้และเ
31	27	ชั้นมัธยมศึกษาตอนต้น		เกษตรกร	แต่งงานอยู่	10000	6000	พอใช้ไม่เห
32	24	ชั้นประถมศึกษา		ว่างงาน	แต่งงานอยู่	9000	7000	ไม่พอใช้
33	26	ชั้นมัธยมศึกษาตอนต้น		รับจ้างรายวั	แต่งงานอยู่	10000	7000	พอใช้ไม่เห
34	27	ชั้นมัธยมศึกษาตอนต้น		ค้าขาย/ธุรกิ	แต่งงานแต่	8000	5000	พอใช้และเ
35	33	ระดับปริญญาตรี		ค้าขาย/ธุรกิ	แต่งงานอยู่	30000	20000	พอใช้และเ
36	27	ชั้นประถมศึกษา		เกษตรกร	ไม่ได้แต่งง	9000	7000	พอใช้ไม่เห
37	18	ชั้นมัธยมตอนปลาย		ว่างงาน	ไม่ได้แต่งง	9000	5000	พอใช้และเ
38	23	ชั้นมัธยมตอนปลาย		ว่างงาน	แต่งงานอยู่	10000	10000	พอใช้ไม่เห
39	26	ระดับปริญญาตรี		ว่างงาน	ไม่ได้แต่งง	3000	3000	พอใช้ไม่เห
40	18	ชั้นมัธยมตอนปลาย		ว่างงาน	แต่งงานอยู่	7000	6000	พอใช้ไม่เห
41	27	ชั้นมัธยมตอนปลาย		รับจ้างรายวั	ไม่ได้แต่งง	15000	25000	ไม่พอใช้
42	38	ชั้นมัธยมศึกษาตอนต้น		ว่างงาน	แต่งงานอยู่	15000	15000	พอใช้ไม่เห
43	24	ชั้นมัธยมศึกษาตอนต้น		รับจ้างรายวั	ไม่ได้แต่งง	7000	5000	พอใช้ไม่เห
44	22	ประกาศนียบัตรวิชาชีพ		ว่างงาน	ไม่ได้แต่งง	10000	12000	ไม่พอใช้
45	18	ชั้นมัธยมตอนปลาย		ว่างงาน	ไม่ได้แต่งง	6000	500	ไม่พอใช้
46	26	ชั้นมัธยมตอนปลาย		ว่างงาน	ไม่ได้แต่งง	#NULL!	#NULL!	ไม่พอใช้
47	21	ชั้นมัธยมตอนปลาย		รับจ้างรายวั	ไม่ได้แต่งง	10000	8000	ไม่พอใช้
48	32	ชั้นประถมศึกษา		เกษตรกร	แต่งงานอยู่	10000	6000	พอใช้ไม่เห
49	30	ชั้นประถมศึกษา		เกษตรกร	แต่งงานอยู่	8000	9000	ไม่พอใช้
50	33	ประกาศนียบัตรวิชาชีพ		ว่างงาน	แต่งงานอยู่	#NULL!	#NULL!	พอใช้ไม่เห

51	27	ชั้นมัธยมตอนปลาย	เกษตรกร	ไม่ได้แต่งง	15000	10000	พอใช้ไม่เห
52	38	ชั้นมัธยมตอนปลาย	เกษตรกร	แต่งงานอยู่	#NULL!	#NULL!	พอใช้ไม่เห
53	18	ชั้นมัธยมศึกษาตอนต้น	ค้าขาย/ธุรกิ	แต่งงานอยู่	8000	7000	พอใช้ไม่เห
54	30	ประกาศนียบัตรวิชาชีพ	ว่างงาน	ไม่ได้แต่งง	15000	12000	พอใช้และเ
55	27	ระดับปริญญาตรี	ว่างงาน	แต่งงานอยู่	20000	10000	พอใช้และเ
56	21	ชั้นมัธยมศึกษาตอนต้น	เกษตรกร	ไม่ได้แต่งง	12000	90000	พอใช้ไม่เห
57	33	ระดับปริญญาตรี	รับราชการ/	แต่งงานอยู่	15000	30000	ไม่พอใช้
58	18	ชั้นมัธยมศึกษาตอนต้น	ว่างงาน	ไม่ได้แต่งง	5000	5000	ไม่พอใช้
59	19	ชั้นประถมศึกษา	ว่างงาน	แต่งงานอยู่	#NULL!	#NULL!	พอใช้ไม่เห
60	37	ชั้นมัธยมตอนปลาย	ว่างงาน	แต่งงานอยู่	10000	6000	พอใช้ไม่เห
61	32	ชั้นมัธยมศึกษาตอนต้น	ค้าขาย/ธุรกิ	แต่งงานอยู่	5000	2000	พอใช้และเ
62	33	ชั้นมัธยมตอนปลาย	รับจ้างรายวั	ไม่ได้แต่งง	8000	11000	พอใช้ไม่เห
63	34	ชั้นมัธยมตอนปลาย	ว่างงาน	แต่งงานอยู่	#NULL!	#NULL!	ไม่พอใช้
64	32	ชั้นมัธยมตอนปลาย	เกษตรกร	แต่งงานอยู่	15000	10000	ไม่พอใช้
65	36	ระดับปริญญาตรี	รับราชการ/	แต่งงานอยู่	30000	15000	พอใช้และเ
66	18	ชั้นมัธยมศึกษาตอนต้น	ค้าขาย/ธุรกิ	แต่งงานอยู่	7000	6000	พอใช้ไม่เห
67	25	ชั้นมัธยมศึกษาตอนต้น	ว่างงาน	แต่งงานอยู่	4000	6000	ไม่พอใช้
68	28	ประกาศนียบัตรวิชาชีพ	ว่างงาน	แต่งงานอยู่	10000	8000	พอใช้ไม่เห
69	23	ชั้นมัธยมตอนปลาย	รับจ้างรายวั	ไม่ได้แต่งง	6000	3800	พอใช้และเ
70	18	ชั้นมัธยมศึกษาตอนต้น	ว่างงาน	ไม่ได้แต่งง	6000	8000	ไม่พอใช้
71	18	ชั้นมัธยมศึกษาตอนต้น	เกษตรกร	แต่งงานอยู่	4000	12000	พอใช้ไม่เห
72	35	ระดับปริญญาตรี	ค้าขาย/ธุรกิ	แต่งงานอยู่	70000	50000	พอใช้และเ
73	30	ประกาศนียบัตรวิชาชีพร	งานรัฐวิสาห	แต่งงานอยู่	10000	5000	พอใช้ไม่เห
74	37	ชั้นมัธยมตอนปลาย	เกษตรกร	แต่งงานอยู่	5000	3000	พอใช้และเ
75	18	ประกาศนียบัตรวิชาชีพ	รับจ้างรายวั	แต่งงานอยู่	3000	2000	พอใช้ไม่เห
76	32	ชั้นมัธยมตอนปลาย	ค้าขาย/ธุรกิ	แต่งงานอยู่	30000	15000	พอใช้ไม่เห
77	34	ระดับปริญญาตรี	รับราชการ/	แต่งงานอยู่	30000	15000	ไม่พอใช้
78	23	ชั้นมัธยมตอนปลาย	เกษตรกร	แต่งงานอยู่	15000	13000	พอใช้ไม่เห
79	23	ประกาศนียบัตรวิชาชีพร	ว่างงาน	แต่งงานอยู่	9000	9000	พอใช้ไม่เห
80	19	ชั้นมัธยมตอนปลาย	ว่างงาน	แต่งงานอยู่	10000	9500	พอใช้ไม่เห
81	43	ชั้นมัธยมตอนปลาย	รับจ้างรายวั	แต่งงานอยู่	#NULL!	#NULL!	ไม่พอใช้
82	34	ชั้นมัธยมศึกษาตอนต้น	ว่างงาน	แต่งงานอยู่	12000	10000	พอใช้และเ
83	18	ชั้นมัธยมศึกษาตอนต้น	ว่างงาน	แต่งงานอยู่	9000	7800	พอใช้ไม่เห
84	20	ชั้นมัธยมตอนปลาย	รับจ้างรายวั	แต่งงานอยู่	12000	11000	พอใช้ไม่เห
85	30	ชั้นมัธยมตอนปลาย	เกษตรกร	แต่งงานอยู่	10000	10000	ไม่พอใช้
86	27	ชั้นมัธยมศึกษาตอนต้น	เกษตรกร	แต่งงานอยู่	12000	3000	พอใช้และเ
87	20	ชั้นมัธยมตอนปลาย	ว่างงาน	ไม่ได้แต่งง	10000	10000	ไม่พอใช้
88	22	ชั้นมัธยมตอนปลาย	ค้าขาย/ธุรกิ	ไม่ได้แต่งง	15000	60000	พอใช้และเ
89	23	ชั้นมัธยมตอนปลาย	รับจ้างรายวั	แต่งงานอยู่	90000	7800	พอใช้ไม่เห
90	24	ชั้นมัธยมตอนปลาย	รับจ้างรายวั	แต่งงานอยู่	12000	10000	พอใช้ไม่เห
91	37	ชั้นมัธยมศึกษาตอนต้น	เกษตรกร	แต่งงานอยู่	12000	1500	พอใช้ไม่เห
92	38	ชั้นมัธยมตอนปลาย	เกษตรกร	แต่งงานอยู่	13000	10000	พอใช้ไม่เห
93	20	ประกาศนียบัตรวิชาชีพร	ว่างงาน	แต่งงานอยู่	14000	5000	พอใช้ไม่เห
94	33	ชั้นมัธยมศึกษาตอนต้น	ว่างงาน	แต่งงานอยู่	20000	15000	พอใช้และเ
95	27	ประกาศนียบัตรวิชาชีพร	ค้าขาย/ธุรกิ	แต่งงานอยู่	15000	9000	พอใช้ไม่เห
96	22	ประกาศนียบัตรวิชาชีพ	ค้าขาย/ธุรกิ	แต่งงานอยู่	90000	15000	ไม่พอใช้
97	22	ชั้นมัธยมตอนปลาย	ค้าขาย/ธุรกิ	แต่งงานอยู่	12000	7000	ไม่พอใช้
98	26	ชั้นมัธยมศึกษาตอนต้น	ว่างงาน	ไม่ได้แต่งง	5000	5000	พอใช้ไม่เห
99	31	ชั้นประถมศึกษา	รับจ้างโรงง	ไม่ได้แต่งง	9000	7000	ไม่พอใช้
100	20	ระดับปริญญาตรี	ว่างงาน	แต่งงานแต่	4000	2000	พอใช้ไม่เห
101	27	ประกาศนียบัตรวิชาชีพ	งานบริษัทเ	ไม่ได้แต่งง	10000	6000	ไม่พอใช้

102	28	ชั้นมัธยมตอนปลาย	ค้าขาย/ธุรกิ	แต่งงานอยู่	12000	12000	พอใช้ไม่เห
103	39	ชั้นมัธยมตอนปลาย	เกษตรกร	แต่งงานอยู่	15000	14000	พอใช้ไม่เห
104	30	ชั้นมัธยมตอนปลาย	ว่างงาน	ไม่ได้แต่งง	8000	7000	ไม่พอใช้
105	33	ชั้นมัธยมศึกษาตอนต้น	ค้าขาย/ธุรกิ	ไม่ได้แต่งง	20000	15000	พอใช้ไม่เห
106	33	ประกาศนียบัตรวิชาชีพร	งานบริษัทเ	แต่งงานอยู่	15000	10000	พอใช้ไม่เห
107	20	ชั้นมัธยมศึกษาตอนต้น	เกษตรกร	แต่งงานอยู่	20000	12000	พอใช้และเ
108	21	ประกาศนียบัตรวิชาชีพ	ว่างงาน	ไม่ได้แต่งง	5000	5000	ไม่พอใช้
109	23	ประกาศนียบัตรวิชาชีพร	เกษตรกร	แต่งงานอยู่	4000	3000	พอใช้ไม่เห
110	20	ชั้นมัธยมตอนปลาย	ว่างงาน	แต่งงานอยู่	8000	7000	พอใช้ไม่เห
111	20	ประกาศนียบัตรวิชาชีพ	รับจ้างรายวั	แต่งงานอยู่	7500	7000	ไม่พอใช้
112	24	ชั้นมัธยมศึกษาตอนต้น	ว่างงาน	แต่งงานอยู่	9000	6000	พอใช้ไม่เห
113	19	ชั้นมัธยมตอนปลาย	รับจ้างรายวั	แต่งงานอยู่	8000	#NULL!	พอใช้ไม่เห
114	41	ชั้นประถมศึกษา	เกษตรกร	แต่งงานอยู่	8000	4000	พอใช้ไม่เห
115	23	ชั้นมัธยมตอนปลาย	ว่างงาน	แต่งงานอยู่	12000	10000	พอใช้ไม่เห
116	35	ชั้นมัธยมตอนปลาย	ค้าขาย/ธุรกิ	แต่งงานอยู่	50000	30000	พอใช้และเ
117	24	ประกาศนียบัตรวิชาชีพร	ว่างงาน	แต่งงานอยู่	8000	10000	พอใช้ไม่เห
118	23	ชั้นมัธยมตอนปลาย	ว่างงาน	แต่งงานแต่	15000	10000	พอใช้ไม่เห
119	31	ชั้นมัธยมตอนปลาย	ว่างงาน	แต่งงานอยู่	5000	4000	ไม่พอใช้
120	23	ชั้นมัธยมตอนปลาย	เกษตรกร	แต่งงานอยู่	8000	8000	พอใช้และเ
121	30	ระดับปริญญาตรี	ค้าขาย/ธุรกิ	แต่งงานแต่	15000	10000	พอใช้ไม่เห
122	20	ชั้นมัธยมตอนปลาย	เกษตรกร	ไม่ได้แต่งง	9000	8000	พอใช้ไม่เห
123	20	ชั้นมัธยมตอนปลาย	งานรัฐวิสาห	ไม่ได้แต่งง	10000	3000	พอใช้และเ
124	22	ประกาศนียบัตรวิชาชีพร	เกษตรกร	แต่งงานอยู่	15000	10000	พอใช้และเ
125	23	ชั้นประถมศึกษา	ว่างงาน	ไม่ได้แต่งง	5000	3500	ไม่พอใช้
126	20	ชั้นมัธยมตอนปลาย	ค้าขาย/ธุรกิ	แต่งงานอยู่	5000	3500	พอใช้และเ
127	23	ประกาศนียบัตรวิชาชีพร	ว่างงาน	ไม่ได้แต่งง	#NULL!	#NULL!	พอใช้ไม่เห
128	21	ประกาศนียบัตรวิชาชีพร	ว่างงาน	แต่งงานอยู่	10000	8000	พอใช้ไม่เห
129	33	ชั้นมัธยมศึกษาตอนต้น	ค้าขาย/ธุรกิ	แต่งงานอยู่	15000	15000	พอใช้ไม่เห
130	27	ชั้นมัธยมตอนปลาย	รับจ้างรายวั	แต่งงานอยู่	15000	5000	พอใช้และเ
131	38	ระดับปริญญาตรี	รับราชการ/	แต่งงานอยู่	15000	10000	พอใช้ไม่เห
132	23	ชั้นมัธยมตอนปลาย	ว่างงาน	แต่งงานอยู่	8000	6000	พอใช้ไม่เห
133	24	ชั้นมัธยมตอนปลาย	เกษตรกร	แต่งงานอยู่	10000	8000	พอใช้ไม่เห
134	29	ระดับปริญญาตรี	ค้าขาย/ธุรกิ	แต่งงานอยู่	15000	10000	พอใช้ไม่เห
135	19	ชั้นมัธยมตอนปลาย	ว่างงาน	แต่งงานอยู่	#NULL!	5000	พอใช้ไม่เห
136	19	ชั้นมัธยมตอนปลาย	รับจ้างรายวั	ไม่ได้แต่งง	10000	7000	พอใช้ไม่เห
137	31	ชั้นมัธยมศึกษาตอนต้น	ว่างงาน	ไม่ได้แต่งง	8000	8000	พอใช้ไม่เห
138	24	ระดับปริญญาตรี	ว่างงาน	แต่งงานอยู่	6000	8000	ไม่พอใช้
139	22	ชั้นมัธยมตอนปลาย	รับจ้างโรงง	แต่งงานอยู่	10500	3000	พอใช้และเ
140	18	ชั้นมัธยมตอนปลาย	ว่างงาน	แต่งงานอยู่	8000	8000	พอใช้ไม่เห
141	23	ชั้นมัธยมตอนปลาย	ว่างงาน	แต่งงานอยู่	15000	20000	ไม่พอใช้
142	21	ชั้นมัธยมตอนปลาย	ว่างงาน	ไม่ได้แต่งง	10000	9000	พอใช้และเ
143	18	ชั้นมัธยมตอนปลาย	ค้าขาย/ธุรกิ	แต่งงานอยู่	7000	5000	พอใช้ไม่เห
144	33	ระดับปริญญาตรี	ค้าขาย/ธุรกิ	แต่งงานอยู่	#NULL!	#NULL!	พอใช้ไม่เห
145	33	ชั้นมัธยมตอนปลาย	เกษตรกร	แต่งงานอยู่	15000	11000	พอใช้ไม่เห
146	28	ระดับปริญญาตรี	รับราชการ/	แต่งงานอยู่	15000	10000	พอใช้และเ
147	20	อื่นๆ ไม่ได้เรียน	ว่างงาน	แต่งงานอยู่	7000	5000	พอใช้และเ
148	24	ชั้นมัธยมศึกษาตอนต้น	เกษตรกร	แต่งงานอยู่	12000	9000	พอใช้และเ
149	22	ชั้นมัธยมศึกษาตอนต้น	รับจ้างรายวั	แต่งงานอยู่	10000	7000	พอใช้และเ
150	21	ชั้นประถมศึกษา	ว่างงาน	ไม่ได้แต่งง	5000	5000	ไม่พอใช้
151	24	ชั้นมัธยมตอนปลาย	ค้าขาย/ธุรกิ	ไม่ได้แต่งง	60000	60000	พอใช้และเ
152	22	ชั้นประถมศึกษา	เกษตรกร	แต่งงานอยู่	8000	5000	พอใช้และเ

153	35	ระดับปริญญาตรี	ว่างงาน	แต่งงานอยู่	5000	3000	พอใช้ไม่เห
154	26	ชั้นมัธยมตอนปลาย	เกษตรกร	แต่งงานอยู่	8000	5000	พอใช้และเ
155	22	ประกาศนียบัตรวิชาชีพ	เกษตรกร	แต่งงานอยู่	15000	5000	พอใช้และเ
156	30	ชั้นประถมศึกษา	เกษตรกร	ไม่ได้แต่งง	10000	8000	พอใช้และเ
157	30	ระดับปริญญาตรี	รับราชการ/	แต่งงานอยู่	19640	5000	พอใช้และเ
158	21	ประกาศนียบัตรวิชาชีพ	ว่างงาน	แต่งงานอยู่	10000	12000	พอใช้และเ
159	21	อื่นๆ ไม่ได้เรียน	รับจ้างรายวั	ไม่ได้แต่งง	15000	9000	พอใช้และเ
160	36	ชั้นมัธยมศึกษาตอนต้น	รับจ้างรายวั	แต่งงานอยู่	9000	11000	ไม่พอใช้
161	30	ชั้นประถมศึกษา	เกษตรกร	แต่งงานอยู่	6000	6000	ไม่พอใช้
162	25	ชั้นมัธยมตอนปลาย	ค้าขาย/ธุรกิ	แต่งงานอยู่	10000	5000	พอใช้ไม่เห
163	31	ระดับปริญญาตรี	รับราชการ/	แต่งงานอยู่	30000	20000	พอใช้และเ
164	23	ประกาศนียบัตรวิชาชีพ	รับจ้างรายวั	แต่งงานอยู่	12000	8000	พอใช้ไม่เห
165	29	ชั้นมัธยมตอนปลาย	ค้าขาย/ธุรกิ	ไม่ได้แต่งง	10000	10000	พอใช้ไม่เห
166	35	ชั้นมัธยมตอนปลาย	เกษตรกร	แต่งงานอยู่	8000	7000	ไม่พอใช้
167	30	ชั้นมัธยมศึกษาตอนต้น	รับจ้างรายวั	ไม่ได้แต่งง	7000	6000	พอใช้ไม่เห
168	22	ชั้นมัธยมศึกษาตอนต้น	เกษตรกร	แต่งงานอยู่	30000	10000	พอใช้ไม่เห
169	35	ชั้นมัธยมตอนปลาย	รับจ้างรายวั	แต่งงานอยู่	6000	4000	ไม่พอใช้
170	23	ระดับปริญญาตรี	ว่างงาน	แต่งงานอยู่	610000	5000	พอใช้ไม่เห
171	31	ชั้นมัธยมตอนปลาย	ค้าขาย/ธุรกิ	แต่งงานอยู่	10000	10000	พอใช้ไม่เห
172	23	ชั้นมัธยมศึกษาตอนต้น	เกษตรกร	แต่งงานอยู่	6000	4000	พอใช้ไม่เห
173	29	ระดับปริญญาตรี	ค้าขาย/ธุรกิ	แต่งงานอยู่	150000	10000	พอใช้และเ
174	24	ประกาศนียบัตรวิชาชีพ	เกษตรกร	แต่งงานอยู่	8000	6000	พอใช้ไม่เห
175	20	ประกาศนียบัตรวิชาชีพ	ว่างงาน	ไม่ได้แต่งง	7000	5000	พอใช้ไม่เห
176	23	ชั้นมัธยมศึกษาตอนต้น	ค้าขาย/ธุรกิ	แต่งงานอยู่	9000	9000	พอใช้ไม่เห
177	33	ชั้นมัธยมศึกษาตอนต้น	เกษตรกร	แต่งงานอยู่	18000	15000	พอใช้ไม่เห
178	23	ชั้นมัธยมศึกษาตอนต้น	รับจ้างรายวั	แต่งงานอยู่	9000	8000	พอใช้ไม่เห
179	32	ชั้นประถมศึกษา	ว่างงาน	แต่งงานอยู่	10000	12000	ไม่พอใช้
180	18	ชั้นมัธยมตอนปลาย	ว่างงาน	แต่งงานอยู่	6000	6000	ไม่พอใช้
181	23	ชั้นมัธยมศึกษาตอนต้น	ค้าขาย/ธุรกิ	แต่งงานอยู่	20000	15000	พอใช้และเ
182	24	ชั้นมัธยมตอนปลาย	ค้าขาย/ธุรกิ	แต่งงานอยู่	30000	10000	พอใช้และเ
183	30	ชั้นมัธยมศึกษาตอนต้น	ค้าขาย/ธุรกิ	แต่งงานอยู่	5000	4000	พอใช้ไม่เห
184	24	ชั้นมัธยมศึกษาตอนต้น	เกษตรกร	แต่งงานอยู่	10000	6000	พอใช้และเ
185	24	ชั้นมัธยมตอนปลาย	ว่างงาน	แต่งงานอยู่	5000	5000	พอใช้ไม่เห
186	18	ชั้นมัธยมตอนปลาย	ว่างงาน	ไม่ได้แต่งง	#NULL!	#NULL!	พอใช้ไม่เห
187	25	ประกาศนียบัตรวิชาชีพ	ว่างงาน	แต่งงานอยู่	22000	19000	พอใช้ไม่เห
188	25	ชั้นมัธยมตอนปลาย	เกษตรกร	แต่งงานอยู่	20000	18000	พอใช้และเ
189	26	ชั้นมัธยมตอนปลาย	ว่างงาน	แต่งงานอยู่	6000	6000	ไม่พอใช้
190	29	ชั้นมัธยมตอนปลาย	เกษตรกร	แต่งงานอยู่	12000	1000	พอใช้ไม่เห
191	29	ระดับปริญญาตรี	ค้าขาย/ธุรกิ	แต่งงานอยู่	10000	3000	พอใช้และเ
192	24	ประกาศนียบัตรวิชาชีพ	ว่างงาน	แต่งงานอยู่	7000	5000	พอใช้ไม่เห
193	26	ประกาศนียบัตรวิชาชีพ	ค้าขาย/ธุรกิ	แต่งงานอยู่	20000	10000	พอใช้และเ
194	21	ชั้นมัธยมตอนปลาย	ว่างงาน	แต่งงานอยู่	12000	10000	พอใช้ไม่เห
195	23	ชั้นมัธยมตอนปลาย	งานบริษัทเ	แต่งงานอยู่	20000	15000	พอใช้ไม่เห
196	31	ประกาศนียบัตรวิชาชีพ	เกษตรกร	ไม่ได้แต่งง	20000	8000	พอใช้และเ
197	34	ชั้นมัธยมตอนปลาย	เกษตรกร	แต่งงานอยู่	#NULL!	#NULL!	ไม่พอใช้
198	29	ระดับปริญญาตรี	รับราชการ/	แต่งงานอยู่	21000	18000	พอใช้ไม่เห
199	23	ชั้นประถมศึกษา	ว่างงาน	ไม่ได้แต่งง	10000	8000	พอใช้และเ
200	40	ชั้นมัธยมศึกษาตอนต้น	เกษตรกร	แต่งงานอยู่	20000	18000	พอใช้ไม่เห
201	18	ประกาศนียบัตรวิชาชีพ	ว่างงาน	ไม่ได้แต่งง	10000	7000	พอใช้ไม่เห
202	20	ชั้นมัธยมตอนปลาย	ว่างงาน	แต่งงานแต่	8000	8000	ไม่พอใช้
203	26	ชั้นมัธยมตอนปลาย	ค้าขาย/ธุรกิ	แต่งงานอยู่	50000	35000	พอใช้และเ

204	35	ชั้นประถมศึกษา	เกษตรกร	แต่งงานอยู่	18000	15000	พอใช้ไม่เห
205	28	ชั้นมัธยมตอนปลาย	เกษตรกร	แต่งงานอยู่	10000	5000	พอใช้และเ
206	21	ชั้นมัธยมตอนปลาย	รับจ้างรายวั	แต่งงานอยู่	10000	7500	พอใช้และเ
207	27	ชั้นมัธยมตอนปลาย	เกษตรกร	แต่งงานอยู่	10000	5000	พอใช้ไม่เห
208	27	ชั้นมัธยมศึกษาตอนต้น	เกษตรกร	ไม่ได้แต่งง	20000	12000	พอใช้และเ
209	40	ระดับปริญญาโท	รับราชการ/	แต่งงานอยู่	30000	28000	พอใช้และเ
210	22	ประกาศนียบัตรวิชาชีพ	ว่างงาน	แต่งงานอยู่	100000	80000	พอใช้ไม่เห
211	30	ชั้นมัธยมศึกษาตอนต้น	ค้าขาย/ธุรกิ	แต่งงานอยู่	16000	7000	พอใช้และเ
212	34	ประกาศนียบัตรวิชาชีพ	เกษตรกร	แต่งงานอยู่	7000	6000	พอใช้ไม่เห
213	34	ประกาศนียบัตรวิชาชีพ	เกษตรกร	แต่งงานอยู่	15000	15000	ไม่พอใช้
214	41	ชั้นประถมศึกษา	เกษตรกร	แต่งงานอยู่	15000	12000	ไม่พอใช้
215	28	ชั้นมัธยมตอนปลาย	รับจ้างรายวั	ไม่ได้แต่งง	10000	8000	พอใช้ไม่เห
216	33	ชั้นมัธยมตอนปลาย	เกษตรกร	แต่งงานอยู่	20000	7500	พอใช้และเ
217	25	ระดับปริญญาตรี	ค้าขาย/ธุรกิ	แต่งงานอยู่	20000	15000	พอใช้และเ
218	23	ชั้นมัธยมตอนปลาย	ค้าขาย/ธุรกิ	แต่งงานอยู่	20000	17000	พอใช้ไม่เห
219	28	ชั้นมัธยมตอนปลาย	ค้าขาย/ธุรกิ	แต่งงานอยู่	17000	12000	พอใช้ไม่เห
220	33	ชั้นมัธยมตอนปลาย	รับจ้างรายวั	แต่งงานอยู่	10000	7000	พอใช้ไม่เห
221	18	ชั้นมัธยมศึกษาตอนต้น	ว่างงาน	แต่งงานอยู่	10000	12000	ไม่พอใช้
222	27	ประกาศนียบัตรวิชาชีพ	ค้าขาย/ธุรกิ	ไม่ได้แต่งง	10000	6000	พอใช้และเ
223	35	ประกาศนียบัตรวิชาชีพ	ค้าขาย/ธุรกิ	แต่งงานอยู่	18000	12000	พอใช้และเ
224	33	ระดับปริญญาตรี	งานบริษัทเ	แต่งงานอยู่	25000	15000	พอใช้และเ
225	21	ประกาศนียบัตรวิชาชีพ	รับจ้างรายวั	ไม่ได้แต่งง	10000	5000	ไม่พอใช้
226	24	ชั้นมัธยมตอนปลาย	ว่างงาน	แต่งงานอยู่	8000	9000	ไม่พอใช้
227	34	ชั้นมัธยมตอนปลาย	ค้าขาย/ธุรกิ	แต่งงานอยู่	5000	#NULL!	พอใช้ไม่เห
228	20	ประกาศนียบัตรวิชาชีพ	ว่างงาน	แต่งงานแต่	#NULL!	#NULL!	ไม่พอใช้
229	40	ชั้นมัธยมศึกษาตอนต้น	รับจ้างรายวั	แต่งงานแต่	#NULL!	#NULL!	พอใช้ไม่เห
230	18	ชั้นมัธยมตอนปลาย	ว่างงาน	แต่งงานอยู่	#NULL!	#NULL!	พอใช้และเ
231	35	ชั้นมัธยมตอนปลาย	รับจ้างรายวั	แต่งงานอยู่	12000	10000	พอใช้และเ
232	31	ระดับปริญญาตรี	งานรัฐวิสาห	ไม่ได้แต่งง	18000	15000	พอใช้และเ
233	27	ระดับปริญญาตรี	เกษตรกร	ไม่ได้แต่งง	15000	9000	พอใช้ไม่เห
234	25	ชั้นมัธยมตอนปลาย	รับจ้างโรงง	แต่งงานอยู่	13000	11000	พอใช้ไม่เห
235	30	ระดับปริญญาตรี	ค้าขาย/ธุรกิ	ไม่ได้แต่งง	20000	10000	พอใช้และเ
236	28	ประกาศนียบัตรวิชาชีพ	งานบริษัทเ	แต่งงานอยู่	10000	4000	พอใช้ไม่เห
237	28	ระดับปริญญาตรี	รับราชการ/	แต่งงานอยู่	10000	15000	ไม่พอใช้
238	18	ชั้นมัธยมตอนปลาย	ว่างงาน	ไม่ได้แต่งง	50000	10000	พอใช้และเ
239	25	ระดับปริญญาตรี	รับราชการ/	แต่งงานอยู่	18000	15000	พอใช้ไม่เห
240	21	ชั้นมัธยมศึกษาตอนต้น	ว่างงาน	แต่งงานอยู่	5000	7000	ไม่พอใช้
241	25	ระดับปริญญาตรี	รับจ้างโรงง	แต่งงานอยู่	15000	10000	พอใช้และเ
242	22	ประกาศนียบัตรวิชาชีพ	ว่างงาน	แต่งงานอยู่	15000	7000	พอใช้ไม่เห
243	34	ประกาศนียบัตรวิชาชีพ	งานรัฐวิสาห	แต่งงานอยู่	20000	15000	พอใช้ไม่เห
244	33	ประกาศนียบัตรวิชาชีพ	รับจ้างรายวั	แต่งงานอยู่	10000	7500	พอใช้ไม่เห
245	21	ประกาศนียบัตรวิชาชีพ	เกษตรกร	ไม่ได้แต่งง	15000	7500	พอใช้และเ
246	22	ชั้นมัธยมตอนปลาย	รับจ้างรายวั	แต่งงานอยู่	9000	7000	พอใช้ไม่เห
247	26	ชั้นมัธยมตอนปลาย	ค้าขาย/ธุรกิ	แต่งงานอยู่	15000	12000	พอใช้ไม่เห
248	21	ชั้นมัธยมศึกษาตอนต้น	ว่างงาน	ไม่ได้แต่งง	8000	6000	พอใช้ไม่เห
249	32	ระดับปริญญาตรี	รับจ้างโรงง	แต่งงานอยู่	17000	10000	พอใช้ไม่เห
250	20	ประกาศนียบัตรวิชาชีพ	รับจ้างรายวั	ไม่ได้แต่งง	17000	7000	พอใช้และเ
251	18	ชั้นมัธยมตอนปลาย	รับจ้างรายวั	ไม่ได้แต่งง	10000	5000	พอใช้ไม่เห
252	28	ระดับปริญญาตรี	รับจ้างรายวั	แต่งงานอยู่	12000	10000	พอใช้ไม่เห
253	19	ชั้นมัธยมตอนปลาย	เกษตรกร	แต่งงานอยู่	12000	6000	ไม่พอใช้
254	19	ชั้นมัธยมตอนปลาย	เกษตรกร	แต่งงานอยู่	10000	5000	พอใช้ไม่เห

255	25	ชั้นมัธยมตอนปลาย	ว่างงาน	ไม่ได้แต่งง	7000	7000	ไม่พอใช้
256	34	ระดับปริญญาตรี	งานบริษัทเ	แต่งงานอยู่	12000	5000	พอใช้และเ
257	36	ระดับปริญญาตรี	รับราชการ/	ไม่ได้แต่งง	20000	10000	พอใช้และเ
258	20	ชั้นมัธยมศึกษาตอนต้น	ว่างงาน	ไม่ได้แต่งง	9000	8000	พอใช้ไม่เห
259	30	ประกาศนียบัตรวิชาชีพร	รับจ้างรายวั	ไม่ได้แต่งง	10000	3000	พอใช้และเ
260	23	ชั้นมัธยมตอนปลาย	ว่างงาน	ไม่ได้แต่งง	9000	7000	พอใช้ไม่เห
261	33	ระดับปริญญาตรี	งานบริษัทเ	แต่งงานอยู่	15000	13000	พอใช้ไม่เห
262	24	ชั้นมัธยมศึกษาตอนต้น	งานบริษัทเ	แต่งงานอยู่	10000	20000	พอใช้ไม่เห
263	31	ระดับปริญญาตรี	ค้าขาย/ธุรกิ	แต่งงานอยู่	90000	#NULL!	พอใช้และเ
264	22	ชั้นมัธยมตอนปลาย	ว่างงาน	แต่งงานอยู่	9000	6000	ไม่พอใช้
265	25	ชั้นประถมศึกษา	ค้าขาย/ธุรกิ	แต่งงานอยู่	15000	10000	พอใช้และเ
266	30	ชั้นมัธยมตอนปลาย	รับจ้างรายวั	แต่งงานอยู่	10000	8000	พอใช้ไม่เห
267	28	ชั้นประถมศึกษา	ว่างงาน	แต่งงานอยู่	8000	7000	พอใช้ไม่เห
268	30	ประกาศนียบัตรวิชาชีพร	เกษตรกร	แต่งงานอยู่	15000	10000	พอใช้ไม่เห
269	26	ระดับปริญญาตรี	ค้าขาย/ธุรกิ	แต่งงานอยู่	20000	10000	ไม่พอใช้
270	18	ชั้นมัธยมตอนปลาย	ว่างงาน	ไม่ได้แต่งง	5000	5000	ไม่พอใช้
271	18	ชั้นมัธยมตอนปลาย	ว่างงาน	แต่งงานอยู่	3000	1000	พอใช้ไม่เห
272	37	ระดับปริญญาตรี	รับราชการ/	แต่งงานอยู่	20000	10000	พอใช้และเ
273	30	ระดับปริญญาตรี	ค้าขาย/ธุรกิ	แต่งงานอยู่	20000	15000	พอใช้ไม่เห
274	27	ชั้นมัธยมศึกษาตอนต้น	รับจ้างรายวั	ไม่ได้แต่งง	9000	8400	ไม่พอใช้
275	25	ชั้นมัธยมตอนปลาย	ว่างงาน	แต่งงานอยู่	10000	6000	พอใช้ไม่เห
276	19	ชั้นมัธยมตอนปลาย	ว่างงาน	แต่งงานอยู่	15000	10000	พอใช้และเ
277	27	ระดับปริญญาตรี	งานบริษัทเ	แต่งงานอยู่	12000	6000	พอใช้ไม่เห
278	30	ประกาศนียบัตรวิชาชีพ	ว่างงาน	แต่งงานอยู่	2000	2000	พอใช้ไม่เห
279	37	ระดับปริญญาตรี	รับราชการ/	แต่งงานแต่	50000	40000	พอใช้และเ
280	27	ระดับปริญญาตรี	รับราชการ/	แต่งงานอยู่	20000	18000	พอใช้ไม่เห
281	24	ชั้นมัธยมศึกษาตอนต้น	ว่างงาน	ไม่ได้แต่งง	8000	8000	พอใช้ไม่เห
282	28	ระดับปริญญาตรี	งานบริษัทเ	แต่งงานอยู่	15000	11000	พอใช้และเ
283	24	ระดับปริญญาตรี	เกษตรกร	ไม่ได้แต่งง	10000	9000	ไม่พอใช้
284	24	ชั้นมัธยมตอนปลาย	รับจ้างโรงง	ไม่ได้แต่งง	7000	5000	พอใช้และเ
285	22	ประกาศนียบัตรวิชาชีพ	ว่างงาน	แต่งงานแต่	12000	10000	พอใช้ไม่เห
286	31	ระดับปริญญาตรี	งานบริษัทเ	แต่งงานอยู่	50000	20000	พอใช้ไม่เห
287	26	ระดับปริญญาตรี	งานบริษัทเ	แต่งงานอยู่	12000	5000	พอใช้ไม่เห
288	20	ชั้นมัธยมศึกษาตอนต้น	ว่างงาน	ไม่ได้แต่งง	10000	8000	พอใช้ไม่เห
289	22	ประกาศนียบัตรวิชาชีพร	รับจ้างรายวั	ไม่ได้แต่งง	12000	3000	พอใช้และเ
290	23	ชั้นมัธยมตอนปลาย	ว่างงาน	ไม่ได้แต่งง	9000	7000	พอใช้ไม่เห
291	33	ระดับปริญญาตรี	งานบริษัทเ	แต่งงานอยู่	#NULL!	#NULL!	พอใช้ไม่เห
292	24	ชั้นมัธยมศึกษาตอนต้น	งานบริษัทเ	แต่งงานอยู่	10000	20000	ไม่พอใช้
293	31	ระดับปริญญาตรี	ค้าขาย/ธุรกิ	แต่งงานอยู่	30000	20000	พอใช้และเ
294	22	ชั้นมัธยมตอนปลาย	ว่างงาน	แต่งงานอยู่	9000	6000	ไม่พอใช้
295	25	ระดับปริญญาตรี	ค้าขาย/ธุรกิ	แต่งงานอยู่	18000	13000	พอใช้และเ
296	30	ชั้นมัธยมตอนปลาย	รับจ้างรายวั	แต่งงานอยู่	18000	15000	พอใช้และเ
297	28	ชั้นประถมศึกษา	ว่างงาน	แต่งงานอยู่	20000	15000	พอใช้และเ
298	30	ประกาศนียบัตรวิชาชีพร	เกษตรกร	แต่งงานอยู่	15000	10000	พอใช้ไม่เห
299	26	ระดับปริญญาตรี	ค้าขาย/ธุรกิ	แต่งงานอยู่	20000	10000	ไม่พอใช้
300	18	ชั้นมัธยมตอนปลาย	ว่างงาน	ไม่ได้แต่งง	#NULL!	#NULL!	พอใช้และเ
301	18	ชั้นมัธยมตอนปลาย	ว่างงาน	แต่งงานอยู่	3000	1000	พอใช้ไม่เห
302	37	ระดับปริญญาตรี	รับราชการ/	แต่งงานอยู่	20000	10000	พอใช้และเ
303	30	ระดับปริญญาตรี	ค้าขาย/ธุรกิ	แต่งงานอยู่	20000	15000	พอใช้ไม่เห
304	27	ชั้นมัธยมศึกษาตอนต้น	รับจ้างรายวั	ไม่ได้แต่งง	9000	8000	ไม่พอใช้
305	25	ชั้นมัธยมตอนปลาย	ว่างงาน	แต่งงานอยู่	10000	6000	พอใช้ไม่เห

306	19	ชั้นมัธยมตอนปลาย	ว่างงาน	แต่งงานอยู่	15000	10000	พอใช้ไม่เห
307	27	ระดับปริญญาตรี	งานบริษัทเ	แต่งงานอยู่	12000	6000	พอใช้ไม่เห
308	30	ประกาศนียบัตรวิชาชีพ	ว่างงาน	แต่งงานอยู่	2000	2000	พอใช้ไม่เห
309	37	ประกาศนียบัตรวิชาชีพร	รับราชการ/	ไม่ได้แต่งง	50000	30000	พอใช้และเ
310	27	ระดับปริญญาตรี	รับราชการ/	แต่งงานอยู่	20000	18000	พอใช้ไม่เห
311	26	ชั้นมัธยมตอนปลาย	ว่างงาน	แต่งงานอยู่	7000	5000	พอใช้ไม่เห
312	21	ชั้นประถมศึกษา	เกษตรกร	แต่งงานอยู่	20000	10000	พอใช้และเ
313	25	ระดับปริญญาตรี	รับราชการ/	แต่งงานอยู่	20000	15000	พอใช้และเ
314	32	ชั้นมัธยมตอนปลาย	รับจ้างรายวั	แต่งงานอยู่	2000	5000	พอใช้ไม่เห
315	22	ชั้นมัธยมศึกษาตอนต้น	รับจ้างรายวั	ไม่ได้แต่งง	10000	8000	พอใช้และเ
316	20	ชั้นมัธยมศึกษาตอนต้น	ว่างงาน	ไม่ได้แต่งง	#NULL!	#NULL!	พอใช้ไม่เห
317	22	ประกาศนียบัตรวิชาชีพร	รับจ้างรายวั	ไม่ได้แต่งง	12000	3000	พอใช้และเ
318	23	ชั้นมัธยมตอนปลาย	ว่างงาน	ไม่ได้แต่งง	9000	7000	พอใช้ไม่เห
319	33	ระดับปริญญาตรี	งานบริษัทเ	แต่งงานอยู่	#NULL!	#NULL!	พอใช้ไม่เห
320	24	ชั้นมัธยมศึกษาตอนต้น	งานบริษัทเ	ไม่ได้แต่งง	20000	10000	พอใช้ไม่เห
321	31	ระดับปริญญาตรี	ค้าขาย/ธุรกิ	แต่งงานอยู่	30000	10000	พอใช้ไม่เห
322	22	ชั้นมัธยมตอนปลาย	ว่างงาน	แต่งงานอยู่	9000	6000	ไม่พอใช้
323	25	ระดับปริญญาตรี	ค้าขาย/ธุรกิ	แต่งงานอยู่	15000	12000	พอใช้และเ
324	30	ชั้นมัธยมตอนปลาย	รับจ้างรายวั	แต่งงานอยู่	#NULL!	#NULL!	ไม่พอใช้
325	28	ชั้นประถมศึกษา	ว่างงาน	แต่งงานอยู่	14000	14000	พอใช้ไม่เห
326	30	ประกาศนียบัตรวิชาชีพร	เกษตรกร	แต่งงานอยู่	15000	10000	พอใช้ไม่เห
327	26	ระดับปริญญาตรี	ค้าขาย/ธุรกิ	แต่งงานอยู่	20000	18000	พอใช้ไม่เห
328	18	ชั้นมัธยมตอนปลาย	ว่างงาน	ไม่ได้แต่งง	5000	3000	ไม่พอใช้
329	18	ชั้นมัธยมตอนปลาย	ว่างงาน	แต่งงานอยู่	3000	1000	พอใช้ไม่เห
330	32	ชั้นมัธยมตอนปลาย	รับจ้างรายวั	แต่งงานอยู่	9000	12000	ไม่พอใช้
331	27	ชั้นมัธยมศึกษาตอนต้น	ว่างงาน	ไม่ได้แต่งง	7000	9000	ไม่พอใช้
332	25	ระดับปริญญาตรี	รับราชการ/	แต่งงานอยู่	23000	19000	พอใช้ไม่เห
333	36	ชั้นมัธยมศึกษาตอนต้น	เกษตรกร	แต่งงานอยู่	12000	17000	ไม่พอใช้
334	27	ระดับปริญญาตรี	งานบริษัทเ	แต่งงานอยู่	22000	17000	พอใช้และเ
335	30	ชั้นประถมศึกษา	เกษตรกร	ไม่ได้แต่งง	8000	8000	พอใช้ไม่เห
336	28	ประกาศนียบัตรวิชาชีพร	ค้าขาย/ธุรกิ	แต่งงานอยู่	30000	28000	พอใช้ไม่เห
337	26	ชั้นมัธยมศึกษาตอนต้น	ว่างงาน	ไม่ได้แต่งง	4000	4000	ไม่พอใช้
338	28	ประกาศนียบัตรวิชาชีพร	รับราชการ/	ไม่ได้แต่งง	12000	10000	พอใช้ไม่เห
339	35	ชั้นมัธยมตอนปลาย	เกษตรกร	ไม่ได้แต่งง	5000	5000	ไม่พอใช้
340	25	ชั้นมัธยมตอนปลาย	รับจ้างรายวั	ไม่ได้แต่งง	9000	11000	ไม่พอใช้
341	35	ชั้นมัธยมตอนปลาย	เกษตรกร	แต่งงานอยู่	7000	7000	พอใช้ไม่เห
342	18	ชั้นมัธยมศึกษาตอนต้น	ว่างงาน	ไม่ได้แต่งง	3000	3000	ไม่พอใช้
343	23	ชั้นมัธยมตอนปลาย	ว่างงาน	ไม่ได้แต่งง	5000	5000	ไม่พอใช้
344	22	ประกาศนียบัตรวิชาชีพ	รับจ้างรายวั	แต่งงานอยู่	12000	15000	ไม่พอใช้
345	22	ประกาศนียบัตรวิชาชีพร	งานบริษัทเ	แต่งงานอยู่	12000	12000	พอใช้ไม่เห
346	19	ประกาศนียบัตรวิชาชีพ	ว่างงาน	ไม่ได้แต่งง	5000	7000	ไม่พอใช้
347	18	ชั้นมัธยมตอนปลาย	ว่างงาน	ไม่ได้แต่งง	6000	8000	ไม่พอใช้
348	39	ระดับปริญญาตรี	รับราชการ/	แต่งงานอยู่	32000	27000	พอใช้และเ
349	37	ระดับปริญญาตรี	รับราชการ/	แต่งงานอยู่	2000	10000	พอใช้และเ
350	21	ชั้นมัธยมตอนปลาย	เกษตรกร	แต่งงานอยู่	8000	8000	พอใช้ไม่เห
351	34	ชั้นมัธยมศึกษาตอนต้น	เกษตรกร	แต่งงานอยู่	9000	10000	ไม่พอใช้
352	19	ประกาศนียบัตรวิชาชีพ	รับจ้างโรงง	ไม่ได้แต่งง	13000	15000	ไม่พอใช้
353	18	ชั้นมัธยมศึกษาตอนต้น	ว่างงาน	ไม่ได้แต่งง	3000	3000	พอใช้ไม่เห
354	33	ชั้นมัธยมศึกษาตอนต้น	เกษตรกร	แต่งงานอยู่	18000	15000	พอใช้ไม่เห
355	23	ชั้นมัธยมศึกษาตอนต้น	รับจ้างรายวั	แต่งงานอยู่	9000	8000	พอใช้ไม่เห
356	32	ชั้นประถมศึกษา	ว่างงาน	แต่งงานอยู่	10000	12000	ไม่พอใช้

357	18	ชั้นมัธยมตอนปลาย	ว่างงาน	แต่งงานอยู่	6000	6000	ไม่พอใช้
358	23	ชั้นมัธยมศึกษาตอนต้น	ค้าขาย/ธุรกิ	แต่งงานอยู่	20000	15000	พอใช้และเ
359	24	ชั้นมัธยมตอนปลาย	ค้าขาย/ธุรกิ	แต่งงานอยู่	30000	10000	พอใช้และเ
360	30	ชั้นมัธยมศึกษาตอนต้น	ค้าขาย/ธุรกิ	แต่งงานอยู่	5000	4000	พอใช้ไม่เห
361	24	ชั้นมัธยมศึกษาตอนต้น	เกษตรกร	แต่งงานอยู่	10000	6000	พอใช้และเ
362	24	ชั้นมัธยมตอนปลาย	ว่างงาน	แต่งงานอยู่	5000	5000	พอใช้ไม่เห
363	18	ชั้นมัธยมตอนปลาย	ว่างงาน	ไม่ได้แต่งง	#NULL!	#NULL!	พอใช้ไม่เห
364	24	ประกาศนียบัตรวิชาชีพ	ว่างงาน	แต่งงานอยู่	7000	5000	พอใช้ไม่เห
365	29	ชั้นมัธยมตอนปลาย	เกษตรกร	แต่งงานอยู่	12000	1000	พอใช้ไม่เห
366	29	ระดับปริญญาตรี	ค้าขาย/ธุรกิ	แต่งงานอยู่	10000	3000	พอใช้และเ
367	24	ประกาศนียบัตรวิชาชีพ	ว่างงาน	แต่งงานอยู่	7000	5000	พอใช้ไม่เห
368	26	ประกาศนียบัตรวิชาชีพช	ค้าขาย/ธุรกิ	แต่งงานอยู่	20000	10000	พอใช้และเ
369	21	ชั้นมัธยมตอนปลาย	ว่างงาน	แต่งงานอยู่	12000	10000	พอใช้ไม่เห
370	23	ชั้นมัธยมตอนปลาย	งานบริษัทเ	แต่งงานอยู่	20000	15000	พอใช้ไม่เห
371	31	ประกาศนียบัตรวิชาชีพ	เกษตรกร	ไม่ได้แต่งง	20000	8000	พอใช้และเ
372	34	ชั้นมัธยมตอนปลาย	เกษตรกร	แต่งงานอยู่	#NULL!	#NULL!	ไม่พอใช้
373	29	ระดับปริญญาตรี	รับราชการ/	แต่งงานอยู่	21000	18000	พอใช้ไม่เห
374	23	ชั้นประถมศึกษา	ว่างงาน	ไม่ได้แต่งง	10000	8000	พอใช้และเ
375	40	ชั้นมัธยมศึกษาตอนต้น	เกษตรกร	แต่งงานอยู่	20000	18000	พอใช้ไม่เห
376	18	ประกาศนียบัตรวิชาชีพ	ว่างงาน	ไม่ได้แต่งง	10000	7000	พอใช้ไม่เห
377	20	ชั้นมัธยมตอนปลาย	ว่างงาน	แต่งงานแต่	8000	8000	ไม่พอใช้
378	26	ชั้นมัธยมตอนปลาย	ค้าขาย/ธุรกิ	แต่งงานอยู่	50000	35000	พอใช้และเ
379	35	ชั้นประถมศึกษา	เกษตรกร	แต่งงานอยู่	18000	15000	พอใช้ไม่เห
380	30	ชั้นประถมศึกษา	เกษตรกร	แต่งงานอยู่	6000	6000	ไม่พอใช้
381	25	ชั้นมัธยมตอนปลาย	ค้าขาย/ธุรกิ	แต่งงานอยู่	10000	5000	พอใช้ไม่เห
382	31	ระดับปริญญาตรี	รับราชการ/	แต่งงานอยู่	30000	20000	พอใช้และเ
383	25	ประกาศนียบัตรวิชาชีพ	รับจ้างรายวั	แต่งงานอยู่	12000	8000	พอใช้ไม่เห
384	29	ชั้นมัธยมตอนปลาย	ค้าขาย/ธุรกิ	ไม่ได้แต่งง	10000	10000	พอใช้ไม่เห
385	35	ชั้นมัธยมตอนปลาย	เกษตรกร	แต่งงานอยู่	8000	7000	ไม่พอใช้
386	30	ชั้นมัธยมศึกษาตอนต้น	รับจ้างรายวั	ไม่ได้แต่งง	7000	6000	พอใช้ไม่เห
387	26	ชั้นมัธยมศึกษาตอนต้น	เกษตรกร	แต่งงานอยู่	30000	10000	พอใช้ไม่เห
388	35	ชั้นมัธยมตอนปลาย	รับจ้างรายวั	แต่งงานอยู่	6000	4000	ไม่พอใช้
389	23	ระดับปริญญาตรี	ว่างงาน	แต่งงานอยู่	610000	5000	พอใช้ไม่เห
390	31	ชั้นมัธยมตอนปลาย	ค้าขาย/ธุรกิ	แต่งงานอยู่	10000	10000	พอใช้ไม่เห
391	23	ชั้นมัธยมศึกษาตอนต้น	เกษตรกร	แต่งงานอยู่	6000	4000	พอใช้ไม่เห
392	29	ระดับปริญญาตรี	ค้าขาย/ธุรกิ	แต่งงานอยู่	150000	10000	พอใช้และเ
393	24	ประกาศนียบัตรวิชาชีพช	เกษตรกร	แต่งงานอยู่	8000	6000	พอใช้ไม่เห
394	20	ประกาศนียบัตรวิชาชีพ	ว่างงาน	ไม่ได้แต่งง	7000	5000	พอใช้ไม่เห
395	23	ชั้นมัธยมศึกษาตอนต้น	ค้าขาย/ธุรกิ	แต่งงานอยู่	9000	9000	พอใช้ไม่เห
396	30	ชั้นมัธยมศึกษาตอนต้น	เกษตรกร	แต่งงานอยู่	18000	15000	พอใช้ไม่เห
397	23	ชั้นมัธยมศึกษาตอนต้น	รับจ้างรายวั	แต่งงานอยู่	9000	8000	พอใช้ไม่เห
398	32	ชั้นประถมศึกษา	ว่างงาน	แต่งงานอยู่	10000	12000	ไม่พอใช้
399	20	ชั้นมัธยมตอนปลาย	ว่างงาน	แต่งงานอยู่	6000	6000	ไม่พอใช้
400	23	ชั้นมัธยมศึกษาตอนต้น	ค้าขาย/ธุรกิ	แต่งงานอยู่	20000	15000	พอใช้และเ
401	24	ชั้นมัธยมตอนปลาย	ค้าขาย/ธุรกิ	แต่งงานอยู่	30000	10000	พอใช้และเ
402	30	ชั้นมัธยมศึกษาตอนต้น	ค้าขาย/ธุรกิ	แต่งงานอยู่	5000	4000	พอใช้ไม่เห
403	34	ระดับปริญญาตรี	รับราชการ/	แต่งงานแต่	20450	20000	พอใช้ไม่เห
404	23	ชั้นมัธยมตอนปลาย	รับราชการ/	ไม่ได้แต่งง	14800	12000	ไม่พอใช้
405	18	ประกาศนียบัตรวิชาชีพ	รับจ้างรายวั	แต่งงานอยู่	3000	2000	พอใช้ไม่เห

ครอบครัว	สิทธิ	person9_o	ศาสนา	โรคประจำตั	person11_	ประวัติทันต	สูบบุหรี่
ครอบครัวเดี	สิทธิหลักประกันสุขภาพ (บัตรทอ		พุทธ	ไม่มี		เคยรับบริก	ไม่เคย
ครอบครัวเดี	สิทธิหลักประกันสุขภาพ (บัตรทอ		พุทธ	ไม่มี		เคยรับบริก	ไม่เคย
ครอบครัวเดี	สิทธิหลักประกันสุขภาพ (บัตรทอ		พุทธ	ไม่มี		ไม่เคยรับบริ	ไม่เคย
ครอบครัวเดี	สิทธิข้าราชการ		พุทธ	ไม่มี		เคยรับบริก	เคยแต่เลิกแ
ครอบครัวเดี	สิทธิประกันสังคม		พุทธ	ไม่มี		เคยรับบริก	ไม่เคย
ครอบครัวเดี	สิทธิหลักประกันสุขภาพ (บัตรทอ		พุทธ	ไม่มี		เคยรับบริก	ไม่เคย
ครอบครัวขย	สิทธิหลักประกันสุขภาพ (บัตรทอ		พุทธ	ไม่มี		ไม่เคยรับบริ	ไม่เคย
ครอบครัวเดี	สิทธิประกันสังคม		พุทธ	ไม่มี		ไม่เคยรับบริ	ไม่เคย
ครอบครัวขย	สิทธิหลักประกันสุขภาพ (บัตรทอ		พุทธ	ไม่มี		เคยรับบริก	ไม่เคย
ครอบครัวเดี	สิทธิหลักประกันสุขภาพ (บัตรทอ		พุทธ	ไม่มี		ไม่เคยรับบริ	ไม่เคย
ครอบครัวเดี	สิทธิหลักประกันสุขภาพ (บัตรทอ		พุทธ	ไม่มี		ไม่เคยรับบริ	ไม่เคย
ครอบครัวขย	สิทธิหลักประกันสุขภาพ (บัตรทอ		พุทธ	ไม่มี		ไม่เคยรับบริ	ไม่เคย
ครอบครัวเดี	สิทธิหลักประกันสุขภาพ (บัตรทอ		พุทธ	ไม่มี		ไม่เคยรับบริ	ไม่เคย
ครอบครัวเดี	สิทธิหลักประกันสุขภาพ (บัตรทอ		พุทธ	ไม่มี		เคยรับบริก	ไม่เคย
ครอบครัวเดี	สิทธิหลักประกันสุขภาพ (บัตรทอ		พุทธ	ไม่มี		เคยรับบริก	ไม่เคย
ครอบครัวเดี	สิทธิหลักประกันสุขภาพ (บัตรทอ		พุทธ	ไม่มี		ไม่เคยรับบริ	ไม่เคย
ครอบครัวเดี	สิทธิหลักประกันสุขภาพ (บัตรทอ		พุทธ	ไม่มี		ไม่เคยรับบริ	ไม่เคย
ครอบครัวเดี	สิทธิประกันสังคม		พุทธ	ไม่มี		ไม่เคยรับบริ	ไม่เคย
ครอบครัวเดี	สิทธิหลักประกันสุขภาพ (บัตรทอ		พุทธ	ไม่มี		ไม่เคยรับบริ	ไม่เคย
ครอบครัวเดี	สิทธิข้าราชการ		พุทธ	ไม่มี		เคยรับบริก	ไม่เคย
ครอบครัวเดี	สิทธิข้าราชการ		พุทธ	ไม่มี		เคยรับบริก	ไม่เคย
ครอบครัวเดี	สิทธิหลักประกันสุขภาพ (บัตรทอ		พุทธ	ไม่มี		เคยรับบริก	ไม่เคย
ครอบครัวขย	สิทธิประกันสังคม		พุทธ	ไม่มี		เคยรับบริก	ไม่เคย
ครอบครัวเดี	สิทธิหลักประกันสุขภาพ (บัตรทอ		พุทธ	ไม่มี		เคยรับบริก	ไม่เคย
ครอบครัวเดี	สิทธิหลักประกันสุขภาพ (บัตรทอ		พุทธ	ไม่มี		ไม่เคยรับบริ	ไม่เคย
ครอบครัวขย	สิทธิหลักประกันสุขภาพ (บัตรทอ		พุทธ	ไม่มี		ไม่เคยรับบริ	ไม่เคย
ครอบครัวเดี	สิทธิหลักประกันสุขภาพ (บัตรทอ		พุทธ	ไม่มี		เคยรับบริก	ไม่เคย
ครอบครัวขย	สิทธิหลักประกันสุขภาพ (บัตรทอ		พุทธ	ไม่มี		ไม่เคยรับบริ	ไม่เคย
ครอบครัวขย	สิทธิหลักประกันสุขภาพ (บัตรทอ		พุทธ	ไม่มี		เคยรับบริก	ไม่เคย
ครอบครัวเดี	สิทธิประกันสังคม		พุทธ	ไม่มี		ไม่เคยรับบริ	ไม่เคย
ครอบครัวเดี	สิทธิหลักประกันสุขภาพ (บัตรทอ		พุทธ	ไม่มี		เคยรับบริก	ไม่เคย
ครอบครัวขย	สิทธิหลักประกันสุขภาพ (บัตรทอ		พุทธ	ไม่มี		ไม่เคยรับบริ	ไม่เคย
ครอบครัวเดี	สิทธิหลักประกันสุขภาพ (บัตรทอ		พุทธ	ไม่มี		เคยรับบริก	ไม่เคย
ครอบครัวเดี	สิทธิหลักประกันสุขภาพ (บัตรทอ		พุทธ	ไม่มี		เคยรับบริก	เคยแต่เลิกแ
ครอบครัวขย	สิทธิหลักประกันสุขภาพ (บัตรทอ		พุทธ	มี	ผนังหัวใจรั่	เคยรับบริก	ไม่เคย
ครอบครัวเดี	สิทธิหลักประกันสุขภาพ (บัตรทอ		พุทธ	ไม่มี		เคยรับบริก	เคยแต่เลิกแ
ครอบครัวเดี	สิทธิหลักประกันสุขภาพ (บัตรทอ		พุทธ	ไม่มี		ไม่เคยรับบริ	เคยแต่เลิกแ
ครอบครัวขย	สิทธิหลักประกันสุขภาพ (บัตรทอ		พุทธ	ไม่มี		ไม่เคยรับบริ	ไม่เคย
ครอบครัวเดี	สิทธิประกันสังคม		พุทธ	ไม่มี		ไม่เคยรับบริ	ไม่เคย
ครอบครัวเดี	สิทธิหลักประกันสุขภาพ (บัตรทอ		พุทธ	ไม่มี		ไม่เคยรับบริ	ไม่เคย
ครอบครัวเดี	สิทธิหลักประกันสุขภาพ (บัตรทอ		อิสลาม	ไม่มี		ไม่เคยรับบริ	ไม่เคย
ครอบครัวเดี	สิทธิหลักประกันสุขภาพ (บัตรทอ		พุทธ	ไม่มี		ไม่เคยรับบริ	ไม่เคย
ครอบครัวเดี	สิทธิหลักประกันสุขภาพ (บัตรทอ		พุทธ	ไม่มี		ไม่เคยรับบริ	เคยแต่เลิกแ
ครอบครัวขย	สิทธิหลักประกันสุขภาพ (บัตรทอ		พุทธ	มี	ภูมิแพ้	เคยรับบริก	ไม่เคย
ครอบครัวเดี	สิทธิหลักประกันสุขภาพ (บัตรทอ		พุทธ	ไม่มี		ไม่เคยรับบริ	ไม่เคย
ครอบครัวเดี	สิทธิประกันสังคม		อื่นๆ	ไม่มี		เคยรับบริก	ไม่เคย
ครอบครัวเดี	สิทธิหลักประกันสุขภาพ (บัตรทอ		พุทธ	ไม่มี		ไม่เคยรับบริ	ไม่เคย
ครอบครัวเดี	สิทธิหลักประกันสุขภาพ (บัตรทอ		พุทธ	ไม่มี		ไม่เคยรับบริ	ไม่เคย
ครอบครัวขย	สิทธิหลักประกันสุขภาพ (บัตรทอ		พุทธ	ไม่มี		เคยรับบริก	ไม่เคย
ครอบครัวเดี	สิทธิหลักประกันสุขภาพ (บัตรทอ		พุทธ	ไม่มี		เคยรับบริก	ไม่เคย

ครอบครัวเดี	สิทธิหลักประกันสุขภาพ (บัตรทอ		พุทธ	ไม่มี		เคยรับบริก	ไม่เคย
ครอบครัวเดี	สิทธิหลักประกันสุขภาพ (บัตรทอ		พุทธ	ไม่มี		ไม่เคยรับบริ	ไม่เคย
ครอบครัวข	สิทธิหลักประกันสุขภาพ (บัตรทอ		พุทธ	ไม่มี		ไม่เคยรับบริ	ไม่เคย
ครอบครัวเดี	สิทธิหลักประกันสุขภาพ (บัตรทอ		พุทธ	ไม่มี		เคยรับบริก	ไม่เคย
ครอบครัวเดี	สิทธิหลักประกันสุขภาพ (บัตรทอ		พุทธ	ไม่มี		เคยรับบริก	ไม่เคย
ครอบครัวเดี	สิทธิหลักประกันสุขภาพ (บัตรทอ		พุทธ	ไม่มี		ไม่เคยรับบริ	ไม่เคย
ครอบครัวข	สิทธิประกันสังคม		พุทธ	ไม่มี		ไม่เคยรับบริ	ไม่เคย
ครอบครัวเดี	สิทธิหลักประกันสุขภาพ (บัตรทอ		พุทธ	ไม่มี		ไม่เคยรับบริ	ไม่เคย
ครอบครัวเดี	สิทธิประกันสังคม		พุทธ	ไม่มี		เคยรับบริก	เคยแต่เลิกแ
ครอบครัวเดี	สิทธิหลักประกันสุขภาพ (บัตรทอ		พุทธ	ไม่มี		ไม่เคยรับบริ	ไม่เคย
ครอบครัวข	อื่นๆ	PPEXpress	พุทธ	ไม่มี		ไม่เคยรับบริ	ไม่เคย
ครอบครัวเดี	สิทธิประกันสังคม		พุทธ	ไม่มี		เคยรับบริก	ไม่เคย
ครอบครัวข	สิทธิหลักประกันสุขภาพ (บัตรทอ		คริสต์	ไม่มี		เคยรับบริก	ไม่เคย
ครอบครัวเดี	สิทธิหลักประกันสุขภาพ (บัตรทอ		พุทธ	ไม่มี		ไม่เคยรับบริ	ไม่เคย
ครอบครัวข	สิทธิข้าราชการ		พุทธ	ไม่มี		เคยรับบริก	ไม่เคย
ครอบครัวเดี	สิทธิหลักประกันสุขภาพ (บัตรทอ		พุทธ	ไม่มี		ไม่เคยรับบริ	ไม่เคย
ครอบครัวข	สิทธิหลักประกันสุขภาพ (บัตรทอ		พุทธ	ไม่มี		ไม่เคยรับบริ	ไม่เคย
ครอบครัวข	สิทธิประกันสังคม		พุทธ	ไม่มี		ไม่เคยรับบริ	ไม่เคย
ครอบครัวเดี	สิทธิหลักประกันสุขภาพ (บัตรทอ		พุทธ	ไม่มี		ไม่เคยรับบริ	ไม่เคย
ครอบครัวเดี	สิทธิหลักประกันสุขภาพ (บัตรทอ		พุทธ	ไม่มี		ไม่เคยรับบริ	ไม่เคย
ครอบครัวข	สิทธิหลักประกันสุขภาพ (บัตรทอ		พุทธ	มี	หอบหืด	เคยรับบริก	ไม่เคย
ครอบครัวข	สิทธิหลักประกันสุขภาพ (บัตรทอ		พุทธ	ไม่มี		ไม่เคยรับบริ	ไม่เคย
ครอบครัวเดี	สิทธิประกันสังคม		พุทธ	ไม่มี		ไม่เคยรับบริ	ไม่เคย
ครอบครัวเดี	สิทธิหลักประกันสุขภาพ (บัตรทอ		พุทธ	ไม่มี		เคยรับบริก	ไม่เคย
ครอบครัวข	สิทธิหลักประกันสุขภาพ (บัตรทอ		พุทธ	ไม่มี		ไม่เคยรับบริ	ไม่เคย
ครอบครัวเดี	สิทธิหลักประกันสุขภาพ (บัตรทอ		พุทธ	ไม่มี		ไม่เคยรับบริ	ไม่เคย
ครอบครัวข	สิทธิหลักประกันสุขภาพ (บัตรทอ		พุทธ	ไม่มี		เคยรับบริก	ไม่เคย
ครอบครัวเดี	สิทธิหลักประกันสุขภาพ (บัตรทอ		พุทธ	ไม่มี		ไม่เคยรับบริ	ไม่เคย
ครอบครัวเดี	สิทธิหลักประกันสุขภาพ (บัตรทอ		พุทธ	ไม่มี		เคยรับบริก	ไม่เคย
ครอบครัวเดี	สิทธิหลักประกันสุขภาพ (บัตรทอ		พุทธ	ไม่มี		ไม่เคยรับบริ	ไม่เคย
ครอบครัวเดี	สิทธิหลักประกันสุขภาพ (บัตรทอ		พุทธ	มี	เบาหวาน	ไม่เคยรับบริ	ไม่เคย
ครอบครัวเดี	สิทธิหลักประกันสุขภาพ (บัตรทอ		พุทธ	ไม่มี		ไม่เคยรับบริ	ไม่เคย
ครอบครัวข	สิทธิหลักประกันสุขภาพ (บัตรทอ		พุทธ	ไม่มี		ไม่เคยรับบริ	ไม่เคย
ครอบครัวเดี	สิทธิหลักประกันสุขภาพ (บัตรทอ		พุทธ	ไม่มี		ไม่เคยรับบริ	ไม่เคย
ครอบครัวเดี	สิทธิหลักประกันสุขภาพ (บัตรทอ		พุทธ	ไม่มี		ไม่เคยรับบริ	ไม่เคย
ครอบครัวเดี	สิทธิหลักประกันสุขภาพ (บัตรทอ		พุทธ	มี	ไทรอยด์	เคยรับบริก	ไม่เคย
ครอบครัวข	สิทธิหลักประกันสุขภาพ (บัตรทอ		พุทธ	ไม่มี		เคยรับบริก	ไม่เคย
ครอบครัวเดี	สิทธิหลักประกันสุขภาพ (บัตรทอ		พุทธ	ไม่มี		เคยรับบริก	ไม่เคย
ครอบครัวเดี	สิทธิหลักประกันสุขภาพ (บัตรทอ		พุทธ	ไม่มี		ไม่เคยรับบริ	ไม่เคย
ครอบครัวเดี	สิทธิหลักประกันสุขภาพ (บัตรทอ		พุทธ	ไม่มี		ไม่เคยรับบริ	ไม่เคย
ครอบครัวเดี	สิทธิหลักประกันสุขภาพ (บัตรทอ		พุทธ	ไม่มี		เคยรับบริก	ไม่เคย
ครอบครัวเดี	สิทธิหลักประกันสุขภาพ (บัตรทอ		พุทธ	ไม่มี		เคยรับบริก	ไม่เคย
ครอบครัวเดี	สิทธิหลักประกันสุขภาพ (บัตรทอ		พุทธ	ไม่มี		เคยรับบริก	ไม่เคย
ครอบครัวเดี	สิทธิประกันสังคม		พุทธ	ไม่มี		ไม่เคยรับบริ	ไม่เคย
ครอบครัวเดี	สิทธิหลักประกันสุขภาพ (บัตรทอ		พุทธ	ไม่มี		ไม่เคยรับบริ	ไม่เคย
ครอบครัวเดี	สิทธิหลักประกันสุขภาพ (บัตรทอ		พุทธ	ไม่มี		เคยรับบริก	ไม่เคย
ครอบครัวเดี	สิทธิหลักประกันสุขภาพ (บัตรทอ		พุทธ	ไม่มี		เคยรับบริก	ไม่เคย
ครอบครัวข	สิทธิหลักประกันสุขภาพ (บัตรทอ		พุทธ	ไม่มี		ไม่เคยรับบริ	ไม่เคย
ครอบครัวเดี	สิทธิประกันสังคม		พุทธ	ไม่มี		เคยรับบริก	ไม่เคย
ครอบครัวข	สิทธิหลักประกันสุขภาพ (บัตรทอ		พุทธ	ไม่มี		เคยรับบริก	ไม่เคย
ครอบครัวข	สิทธิประกันสังคม		พุทธ	ไม่มี		เคยรับบริก	ไม่เคย

ครอบครัวเดี่	สิทธิหลักประกันสุขภาพ (บัตรทอ		พุทธ	ไม่มี		เคยรับบริก	ไม่เคย
ครอบครัวขย	สิทธิหลักประกันสุขภาพ (บัตรทอ		พุทธ	ไม่มี		ไม่เคยรับบริ	ไม่เคย
ครอบครัวเดี่	สิทธิประกันสังคม		พุทธ	ไม่มี		ไม่เคยรับบริ	ไม่เคย
ครอบครัวเดี่	สิทธิหลักประกันสุขภาพ (บัตรทอ		พุทธ	ไม่มี		ไม่เคยรับบริ	ไม่เคย
ครอบครัวเดี่	สิทธิประกันสังคม		พุทธ	ไม่มี		ไม่เคยรับบริ	ไม่เคย
ครอบครัวเดี่	สิทธิหลักประกันสุขภาพ (บัตรทอ		พุทธ	ไม่มี		เคยรับบริก	ไม่เคย
ครอบครัวเดี่	สิทธิหลักประกันสุขภาพ (บัตรทอ		พุทธ	ไม่มี		ไม่เคยรับบริ	ไม่เคย
ครอบครัวเดี่	สิทธิหลักประกันสุขภาพ (บัตรทอ		พุทธ	ไม่มี		เคยรับบริก	ไม่เคย
ครอบครัวขย	สิทธิหลักประกันสุขภาพ (บัตรทอ		พุทธ	มี	มะเร็งเม็ดเลื	ไม่เคยรับบริ	ไม่เคย
ครอบครัวเดี่	สิทธิหลักประกันสุขภาพ (บัตรทอ		พุทธ	ไม่มี		ไม่เคยรับบริ	ไม่เคย
ครอบครัวเดี่	สิทธิหลักประกันสุขภาพ (บัตรทอ		พุทธ	ไม่มี		ไม่เคยรับบริ	ไม่เคย
ครอบครัวเดี่	สิทธิหลักประกันสุขภาพ (บัตรทอ		พุทธ	ไม่มี		ไม่เคยรับบริ	ไม่เคย
ครอบครัวเดี่	สิทธิหลักประกันสุขภาพ (บัตรทอ		พุทธ	ไม่มี		เคยรับบริก	ไม่เคย
ครอบครัวขย	สิทธิหลักประกันสุขภาพ (บัตรทอ		พุทธ	ไม่มี		ไม่เคยรับบริ	ไม่เคย
ครอบครัวขย	สิทธิหลักประกันสุขภาพ (บัตรทอ		พุทธ	ไม่มี		เคยรับบริก	ไม่เคย
ครอบครัวเดี่	สิทธิหลักประกันสุขภาพ (บัตรทอ		พุทธ	ไม่มี		เคยรับบริก	ไม่เคย
ครอบครัวเดี่	สิทธิหลักประกันสุขภาพ (บัตรทอ		พุทธ	ไม่มี		ไม่เคยรับบริ	ไม่เคย
ครอบครัวเดี่	สิทธิหลักประกันสุขภาพ (บัตรทอ		พุทธ	ไม่มี		ไม่เคยรับบริ	ไม่เคย
ครอบครัวเดี่	สิทธิหลักประกันสุขภาพ (บัตรทอ		พุทธ	ไม่มี		เคยรับบริก	ไม่เคย
ครอบครัวเดี่	สิทธิหลักประกันสุขภาพ (บัตรทอ		พุทธ	ไม่มี		ไม่เคยรับบริ	ไม่เคย
ครอบครัวเดี่	สิทธิหลักประกันสุขภาพ (บัตรทอ		พุทธ	ไม่มี		เคยรับบริก	ไม่เคย
ครอบครัวเดี่	สิทธิหลักประกันสุขภาพ (บัตรทอ		พุทธ	ไม่มี		เคยรับบริก	เคยแต่เลิกแ
ครอบครัวเดี่	อื่นๆ	จ่ายเอง	พุทธ	ไม่มี		ไม่เคยรับบริ	ไม่เคย
ครอบครัวขย	สิทธิหลักประกันสุขภาพ (บัตรทอ		พุทธ	ไม่มี		เคยรับบริก	ไม่เคย
ครอบครัวเดี่	สิทธิหลักประกันสุขภาพ (บัตรทอ		พุทธ	ไม่มี		ไม่เคยรับบริ	ไม่เคย
ครอบครัวเดี่	สิทธิหลักประกันสุขภาพ (บัตรทอ		พุทธ	ไม่มี		เคยรับบริก	ไม่เคย
ครอบครัวขย	อื่นๆ	จ่ายเอง	พุทธ	ไม่มี		ไม่เคยรับบริ	ไม่เคย
ครอบครัวขย	สิทธิหลักประกันสุขภาพ (บัตรทอ		พุทธ	ไม่มี		ไม่เคยรับบริ	ไม่เคย
ครอบครัวเดี่	สิทธิหลักประกันสุขภาพ (บัตรทอ		พุทธ	ไม่มี		เคยรับบริก	ไม่เคย
ครอบครัวเดี่	สิทธิประกันสังคม		พุทธ	ไม่มี		เคยรับบริก	ไม่เคย
ครอบครัวเดี่	สิทธิหลักประกันสุขภาพ (บัตรทอ		พุทธ	มี	ภูมิแพ้	ไม่เคยรับบริ	ไม่เคย
ครอบครัวเดี่	สิทธิหลักประกันสุขภาพ (บัตรทอ		พุทธ	มี	หอบหืด	เคยรับบริก	ไม่เคย
ครอบครัวขย	สิทธิหลักประกันสุขภาพ (บัตรทอ		พุทธ	มี	ไวรัสตับอัก	เคยรับบริก	ไม่เคย
ครอบครัวเดี่	อื่นๆ	จ่ายเอง	พุทธ	ไม่มี		เคยรับบริก	เคยแต่เลิกแ
ครอบครัวเดี่	สิทธิหลักประกันสุขภาพ (บัตรทอ		พุทธ	ไม่มี		เคยรับบริก	ไม่เคย
ครอบครัวเดี่	สิทธิประกันสังคม		พุทธ	ไม่มี		ไม่เคยรับบริ	ไม่เคย
ครอบครัวเดี่	สิทธิหลักประกันสุขภาพ (บัตรทอ		พุทธ	ไม่มี		เคยรับบริก	ไม่เคย
ครอบครัวเดี่	สิทธิหลักประกันสุขภาพ (บัตรทอ		พุทธ	ไม่มี		เคยรับบริก	ไม่เคย
ครอบครัวขย	สิทธิหลักประกันสุขภาพ (บัตรทอ		พุทธ	ไม่มี		เคยรับบริก	เคยแต่เลิกแ
ครอบครัวขย	สิทธิหลักประกันสุขภาพ (บัตรทอ		พุทธ	ไม่มี		เคยรับบริก	ไม่เคย
ครอบครัวเดี่	สิทธิประกันสังคม		พุทธ	ไม่มี		ไม่เคยรับบริ	ไม่เคย
ครอบครัวเดี่	สิทธิหลักประกันสุขภาพ (บัตรทอ		พุทธ	มี	หอบหืด	ไม่เคยรับบริ	ไม่เคย
ครอบครัวเดี่	สิทธิหลักประกันสุขภาพ (บัตรทอ		พุทธ	ไม่มี		เคยรับบริก	ไม่เคย
ครอบครัวเดี่	สิทธิหลักประกันสุขภาพ (บัตรทอ		พุทธ	ไม่มี		ไม่เคยรับบริ	ไม่เคย
ครอบครัวขย	สิทธิหลักประกันสุขภาพ (บัตรทอ		พุทธ	ไม่มี		ไม่เคยรับบริ	ไม่เคย
ครอบครัวเดี่	สิทธิหลักประกันสุขภาพ (บัตรทอ		พุทธ	ไม่มี		ไม่เคยรับบริ	ไม่เคย
ครอบครัวเดี่	สิทธิหลักประกันสุขภาพ (บัตรทอ		พุทธ	ไม่มี		เคยรับบริก	ไม่เคย
ครอบครัวเดี่	สิทธิหลักประกันสุขภาพ (บัตรทอ		พุทธ	ไม่มี		ไม่เคยรับบริ	ไม่เคย
ครอบครัวเดี่	สิทธิประกันสังคม		พุทธ	ไม่มี		เคยรับบริก	ไม่เคย
ครอบครัวขย	สิทธิหลักประกันสุขภาพ (บัตรทอ		พุทธ	ไม่มี		ไม่เคยรับบริ	ไม่เคย
ครอบครัวเดี่	สิทธิหลักประกันสุขภาพ (บัตรทอ		พุทธ	ไม่มี		เคยรับบริก	ไม่เคย

ครอบครัวเดี่	สิทธิหลักประกันสุขภาพ (บัตรทอ		พุทธ	ไม่มี		เคยรับบริก	ไม่เคย
ครอบครัวเดี่	สิทธิหลักประกันสุขภาพ (บัตรทอ		พุทธ	มี	ภูมิแพ้	ไม่เคยรับบริ	ไม่เคย
ครอบครัวเดี่	สิทธิหลักประกันสุขภาพ (บัตรทอ		พุทธ	ไม่มี		ไม่เคยรับบริ	ไม่เคย
ครอบครัวเดี่	สิทธิหลักประกันสุขภาพ (บัตรทอ		พุทธ	ไม่มี		ไม่เคยรับบริ	ไม่เคย
ครอบครัวเดี่	สิทธิข้าราชการ		พุทธ	ไม่มี		ไม่เคยรับบริ	ไม่เคย
ครอบครัวเดี่	สิทธิประกันสังคม		พุทธ	ไม่มี		ไม่เคยรับบริ	ไม่เคย
ครอบครัวเดี่	อื่นๆ	จ่ายเอง	พุทธ	ไม่มี		ไม่เคยรับบริ	ไม่เคย
ครอบครัวเดี่	สิทธิประกันสังคม		พุทธ	ไม่มี		ไม่เคยรับบริ	ไม่เคย
ครอบครัวเดี่	สิทธิหลักประกันสุขภาพ (บัตรทอ		พุทธ	ไม่มี		เคยรับบริก	ไม่เคย
ครอบครัวเดี่	สิทธิหลักประกันสุขภาพ (บัตรทอ		พุทธ	ไม่มี		ไม่เคยรับบริ	ไม่เคย
ครอบครัวเดี่	สิทธิหลักประกันสุขภาพ (บัตรทอ		พุทธ	ไม่มี		เคยรับบริก	ไม่เคย
ครอบครัวข	สิทธิหลักประกันสุขภาพ (บัตรทอ		พุทธ	ไม่มี		ไม่เคยรับบริ	ไม่เคย
ครอบครัวเดี่	สิทธิหลักประกันสุขภาพ (บัตรทอ		พุทธ	ไม่มี		เคยรับบริก	ไม่เคย
ครอบครัวข	สิทธิหลักประกันสุขภาพ (บัตรทอ		พุทธ	ไม่มี		ไม่เคยรับบริ	ไม่เคย
ครอบครัวเดี่	อื่นๆ	จ่ายเอง	พุทธ	ไม่มี		ไม่เคยรับบริ	ไม่เคย
ครอบครัวเดี่	สิทธิหลักประกันสุขภาพ (บัตรทอ		พุทธ	ไม่มี		ไม่เคยรับบริ	ไม่เคย
ครอบครัวเดี่	สิทธิหลักประกันสุขภาพ (บัตรทอ		พุทธ	ไม่มี		เคยรับบริก	ไม่เคย
ครอบครัวข	สิทธิหลักประกันสุขภาพ (บัตรทอ		พุทธ	ไม่มี		ไม่เคยรับบริ	ไม่เคย
ครอบครัวเดี่	สิทธิหลักประกันสุขภาพ (บัตรทอ		พุทธ	ไม่มี		ไม่เคยรับบริ	ไม่เคย
ครอบครัวเดี่	สิทธิหลักประกันสุขภาพ (บัตรทอ		พุทธ	ไม่มี		ไม่เคยรับบริ	ไม่เคย
ครอบครัวข	สิทธิหลักประกันสุขภาพ (บัตรทอ		พุทธ	ไม่มี		เคยรับบริก	ไม่เคย
ครอบครัวข	สิทธิหลักประกันสุขภาพ (บัตรทอ		พุทธ	ไม่มี		เคยรับบริก	ไม่เคย
ครอบครัวเดี่	สิทธิหลักประกันสุขภาพ (บัตรทอ		พุทธ	ไม่มี		เคยรับบริก	ไม่เคย
ครอบครัวเดี่	สิทธิหลักประกันสุขภาพ (บัตรทอ		พุทธ	ไม่มี		เคยรับบริก	ไม่เคย
ครอบครัวข	สิทธิหลักประกันสุขภาพ (บัตรทอ		พุทธ	ไม่มี		เคยรับบริก	ไม่เคย
ครอบครัวเดี่	สิทธิหลักประกันสุขภาพ (บัตรทอ		พุทธ	ไม่มี		ไม่เคยรับบริ	ไม่เคย
ครอบครัวเดี่	สิทธิหลักประกันสุขภาพ (บัตรทอ		พุทธ	ไม่มี		เคยรับบริก	ไม่เคย
ครอบครัวข	สิทธิหลักประกันสุขภาพ (บัตรทอ		พุทธ	ไม่มี		เคยรับบริก	ไม่เคย
ครอบครัวเดี่	สิทธิหลักประกันสุขภาพ (บัตรทอ		พุทธ	ไม่มี		เคยรับบริก	ไม่เคย
ครอบครัวเดี่	สิทธิประกันสังคม		พุทธ	ไม่มี		ไม่เคยรับบริ	ไม่เคย
ครอบครัวข	สิทธิหลักประกันสุขภาพ (บัตรทอ		พุทธ	ไม่มี		ไม่เคยรับบริ	ไม่เคย
ครอบครัวเดี่	สิทธิหลักประกันสุขภาพ (บัตรทอ		พุทธ	ไม่มี		ไม่เคยรับบริ	ไม่เคย
ครอบครัวเดี่	สิทธิหลักประกันสุขภาพ (บัตรทอ		พุทธ	ไม่มี		ไม่เคยรับบริ	เคยแต่เลิกแ
ครอบครัวเดี่	สิทธิหลักประกันสุขภาพ (บัตรทอ		พุทธ	ไม่มี		ไม่เคยรับบริ	ไม่เคย
ครอบครัวเดี่	สิทธิข้าราชการ		พุทธ	ไม่มี		เคยรับบริก	ไม่เคย
ครอบครัวเดี่	สิทธิหลักประกันสุขภาพ (บัตรทอ		พุทธ	ไม่มี		ไม่เคยรับบริ	ไม่เคย
ครอบครัวข	สิทธิหลักประกันสุขภาพ (บัตรทอ		พุทธ	ไม่มี		เคยรับบริก	ไม่เคย
ครอบครัวเดี่	สิทธิหลักประกันสุขภาพ (บัตรทอ		พุทธ	ไม่มี		เคยรับบริก	ไม่เคย
ครอบครัวเดี่	สิทธิประกันสังคม		พุทธ	ไม่มี		เคยรับบริก	ไม่เคย
ครอบครัวเดี่	สิทธิหลักประกันสุขภาพ (บัตรทอ		พุทธ	ไม่มี		เคยรับบริก	ไม่เคย
ครอบครัวเดี่	สิทธิหลักประกันสุขภาพ (บัตรทอ		พุทธ	ไม่มี		เคยรับบริก	ไม่เคย
ครอบครัวเดี่	สิทธิหลักประกันสุขภาพ (บัตรทอ		พุทธ	ไม่มี		เคยรับบริก	ไม่เคย
ครอบครัวเดี่	สิทธิประกันสังคม		พุทธ	ไม่มี		เคยรับบริก	ไม่เคย
ครอบครัวเดี่	สิทธิหลักประกันสุขภาพ (บัตรทอ		พุทธ	ไม่มี		ไม่เคยรับบริ	ไม่เคย
ครอบครัวเดี่	สิทธิหลักประกันสุขภาพ (บัตรทอ		พุทธ	ไม่มี		เคยรับบริก	ไม่เคย
ครอบครัวข	สิทธิข้าราชการ		พุทธ	ไม่มี		ไม่เคยรับบริ	ไม่เคย
ครอบครัวเดี่	สิทธิหลักประกันสุขภาพ (บัตรทอ		พุทธ	ไม่มี		เคยรับบริก	ไม่เคย
ครอบครัวเดี่	สิทธิหลักประกันสุขภาพ (บัตรทอ		พุทธ	ไม่มี		ไม่เคยรับบริ	ไม่เคย
ครอบครัวเดี่	สิทธิหลักประกันสุขภาพ (บัตรทอ		พุทธ	ไม่มี		เคยรับบริก	ไม่เคย
ครอบครัวเดี่	สิทธิหลักประกันสุขภาพ (บัตรทอ		พุทธ	ไม่มี		ไม่เคยรับบริ	ไม่เคย
ครอบครัวเดี่	สิทธิหลักประกันสุขภาพ (บัตรทอ		พุทธ	ไม่มี		ไม่เคยรับบริ	ไม่เคย

ครอบครัวเดี่	สิทธิหลักประกันสุขภาพ (บัตรทอ	พุทธ	ไม่มี		เคยรับบริก	ไม่เคย
ครอบครัวเดี่	สิทธิหลักประกันสุขภาพ (บัตรทอ	พุทธ	ไม่มี		ไม่เคยรับบริ	ไม่เคย
ครอบครัวเดี่	สิทธิหลักประกันสุขภาพ (บัตรทอ	พุทธ	ไม่มี		เคยรับบริก	ไม่เคย
ครอบครัวเดี่	สิทธิหลักประกันสุขภาพ (บัตรทอ	พุทธ	ไม่มี		ไม่เคยรับบริ	ไม่เคย
ครอบครัวเดี่	สิทธิหลักประกันสุขภาพ (บัตรทอ	พุทธ	ไม่มี		เคยรับบริก	ไม่เคย
ครอบครัวขย	สิทธิข้าราชการ	พุทธ	ไม่มี		ไม่เคยรับบริ	ไม่เคย
ครอบครัวเดี่	สิทธิหลักประกันสุขภาพ (บัตรทอ	พุทธ	ไม่มี		เคยรับบริก	ไม่เคย
ครอบครัวเดี่	สิทธิประกันสังคม	พุทธ	ไม่มี		เคยรับบริก	ไม่เคย
ครอบครัวเดี่	สิทธิหลักประกันสุขภาพ (บัตรทอ	พุทธ	ไม่มี		เคยรับบริก	ไม่เคย
ครอบครัวเดี่	สิทธิหลักประกันสุขภาพ (บัตรทอ	พุทธ	ไม่มี		ไม่เคยรับบริ	ไม่เคย
ครอบครัวเดี่	สิทธิหลักประกันสุขภาพ (บัตรทอ	พุทธ	ไม่มี		ไม่เคยรับบริ	ไม่เคย
ครอบครัวขย	สิทธิหลักประกันสุขภาพ (บัตรทอ	พุทธ	ไม่มี		เคยรับบริก	ไม่เคย
ครอบครัวขย	สิทธิหลักประกันสุขภาพ (บัตรทอ	พุทธ	ไม่มี		เคยรับบริก	ไม่เคย
ครอบครัวเดี่	สิทธิหลักประกันสุขภาพ (บัตรทอ	พุทธ	ไม่มี		เคยรับบริก	ไม่เคย
ครอบครัวเดี่	สิทธิหลักประกันสุขภาพ (บัตรทอ	พุทธ	ไม่มี		เคยรับบริก	ไม่เคย
ครอบครัวเดี่	สิทธิหลักประกันสุขภาพ (บัตรทอ	พุทธ	ไม่มี		เคยรับบริก	ไม่เคย
ครอบครัวเดี่	สิทธิหลักประกันสุขภาพ (บัตรทอ	พุทธ	ไม่มี		เคยรับบริก	ไม่เคย
ครอบครัวขย	สิทธิหลักประกันสุขภาพ (บัตรทอ	พุทธ	ไม่มี		เคยรับบริก	ไม่เคย
ครอบครัวเดี่	สิทธิหลักประกันสุขภาพ (บัตรทอ	พุทธ	ไม่มี		เคยรับบริก	ไม่เคย
ครอบครัวเดี่	สิทธิหลักประกันสุขภาพ (บัตรทอ	พุทธ	ไม่มี		เคยรับบริก	เคยแต่เลิก
ครอบครัวขย	สิทธิประกันสังคม	พุทธ	มี	หอบหืด	เคยรับบริก	ไม่เคย
ครอบครัวขย	สิทธิหลักประกันสุขภาพ (บัตรทอ	พุทธ	ไม่มี		เคยรับบริก	ไม่เคย
ครอบครัวเดี่	สิทธิหลักประกันสุขภาพ (บัตรทอ	พุทธ	ไม่มี		เคยรับบริก	ไม่เคย
ครอบครัวเดี่	สิทธิหลักประกันสุขภาพ (บัตรทอ	พุทธ	ไม่มี		เคยรับบริก	ไม่เคย
ครอบครัวขย	สิทธิหลักประกันสุขภาพ (บัตรทอ	พุทธ	ไม่มี		เคยรับบริก	ไม่เคย
ครอบครัวเดี่	สิทธิหลักประกันสุขภาพ (บัตรทอ	พุทธ	ไม่มี		ไม่เคยรับบริ	ไม่เคย
ครอบครัวขย	สิทธิหลักประกันสุขภาพ (บัตรทอ	คริสต์	ไม่มี		เคยรับบริก	ไม่เคย
ครอบครัวเดี่	สิทธิประกันสังคม	พุทธ	ไม่มี		เคยรับบริก	ไม่เคย
ครอบครัวขย	สิทธิข้าราชการ	พุทธ	ไม่มี		เคยรับบริก	ไม่เคย
ครอบครัวเดี่	สิทธิหลักประกันสุขภาพ (บัตรทอ	พุทธ	ไม่มี		เคยรับบริก	ไม่เคย
ครอบครัวเดี่	สิทธิประกันสังคม	พุทธ	ไม่มี		เคยรับบริก	ไม่เคย
ครอบครัวเดี่	สิทธิหลักประกันสุขภาพ (บัตรทอ	พุทธ	ไม่มี		เคยรับบริก	ไม่เคย
ครอบครัวเดี่	สิทธิประกันสังคม	พุทธ	ไม่มี		ไม่เคยรับบริ	ไม่เคย
ครอบครัวเดี่	สิทธิประกันสังคม	พุทธ	ไม่มี		เคยรับบริก	ไม่เคย
ครอบครัวขย	สิทธิหลักประกันสุขภาพ (บัตรทอ	พุทธ	ไม่มี		เคยรับบริก	เคยแต่เลิก
ครอบครัวเดี่	สิทธิข้าราชการ	พุทธ	ไม่มี		ไม่เคยรับบริ	ไม่เคย
ครอบครัวเดี่	สิทธิหลักประกันสุขภาพ (บัตรทอ	พุทธ	ไม่มี		เคยรับบริก	ไม่เคย
ครอบครัวเดี่	สิทธิหลักประกันสุขภาพ (บัตรทอ	พุทธ	ไม่มี		ไม่เคยรับบริ	เคยแต่เลิก
ครอบครัวเดี่	สิทธิหลักประกันสุขภาพ (บัตรทอ	พุทธ	ไม่มี		เคยรับบริก	เคยแต่เลิก
ครอบครัวเดี่	สิทธิประกันสังคม	พุทธ	มี	หอบหืด	เคยรับบริก	ไม่เคย
ครอบครัวเดี่	สิทธิหลักประกันสุขภาพ (บัตรทอ	พุทธ	ไม่มี		เคยรับบริก	ไม่เคย
ครอบครัวขย	สิทธิหลักประกันสุขภาพ (บัตรทอ	พุทธ	ไม่มี		เคยรับบริก	ไม่เคย
ครอบครัวเดี่	สิทธิหลักประกันสุขภาพ (บัตรทอ	พุทธ	ไม่มี		เคยรับบริก	ไม่เคย
ครอบครัวเดี่	สิทธิหลักประกันสุขภาพ (บัตรทอ	พุทธ	ไม่มี		เคยรับบริก	ไม่เคย
ครอบครัวเดี่	สิทธิหลักประกันสุขภาพ (บัตรทอ	พุทธ	ไม่มี		เคยรับบริก	ไม่เคย
ครอบครัวขย	สิทธิหลักประกันสุขภาพ (บัตรทอ	พุทธ	ไม่มี		เคยรับบริก	ไม่เคย
ครอบครัวขย	สิทธิหลักประกันสุขภาพ (บัตรทอ	พุทธ	ไม่มี		เคยรับบริก	ไม่เคย
ครอบครัวขย	สิทธิหลักประกันสุขภาพ (บัตรทอ	พุทธ	ไม่มี		เคยรับบริก	ไม่เคย
ครอบครัวขย	สิทธิประกันสังคม	พุทธ	ไม่มี		เคยรับบริก	ไม่เคย
ครอบครัวขย	สิทธิหลักประกันสุขภาพ (บัตรทอ	พุทธ	ไม่มี		เคยรับบริก	เคยแต่เลิก
ครอบครัวเดี่	สิทธิหลักประกันสุขภาพ (บัตรทอ	พุทธ	ไม่มี		เคยรับบริก	ไม่เคย

ครอบครัวเดี่	สิทธิหลักประกันสุขภาพ (บัตรทอ	พุทธ	ไม่มี		เคยรับบริก	เคยแต่เลิก
ครอบครัวข	สิทธิประกันสังคม	พุทธ	ไม่มี		ไม่เคยรับบ	ไม่เคย
ครอบครัวเดี่	สิทธิข้าราชการ	พุทธ	ไม่มี		ไม่เคยรับบ	ไม่เคย
ครอบครัวเดี่	สิทธิประกันสังคม	พุทธ	ไม่มี		ไม่เคยรับบ	ไม่เคย
ครอบครัวเดี่	สิทธิหลักประกันสุขภาพ (บัตรทอ	พุทธ	ไม่มี		ไม่เคยรับบ	ไม่เคย
ครอบครัวข	สิทธิประกันสังคม	พุทธ	ไม่มี		เคยรับบริก	ไม่เคย
ครอบครัวเดี่	สิทธิประกันสังคม	พุทธ	ไม่มี		ไม่เคยรับบ	ไม่เคย
ครอบครัวเดี่	สิทธิประกันสังคม	พุทธ	มี	ไทรอยด์	เคยรับบริก	ไม่เคย
ครอบครัวข	สิทธิหลักประกันสุขภาพ (บัตรทอ	พุทธ	ไม่มี		เคยรับบริก	ไม่เคย
ครอบครัวเดี่	สิทธิหลักประกันสุขภาพ (บัตรทอ	พุทธ	ไม่มี		ไม่เคยรับบ	ไม่เคย
ครอบครัวเดี่	สิทธิข้าราชการ	พุทธ	ไม่มี		ไม่เคยรับบ	ไม่เคย
ครอบครัวข	สิทธิหลักประกันสุขภาพ (บัตรทอ	พุทธ	ไม่มี		ไม่เคยรับบ	ไม่เคย
ครอบครัวเดี่	สิทธิหลักประกันสุขภาพ (บัตรทอ	พุทธ	ไม่มี		ไม่เคยรับบ	เคยแต่เลิก
ครอบครัวเดี่	สิทธิหลักประกันสุขภาพ (บัตรทอ	พุทธ	ไม่มี		เคยรับบริก	ไม่เคย
ครอบครัวเดี่	สิทธิหลักประกันสุขภาพ (บัตรทอ	พุทธ	ไม่มี		เคยรับบริก	ไม่เคย
ครอบครัวเดี่	สิทธิหลักประกันสุขภาพ (บัตรทอ	พุทธ	ไม่มี		ไม่เคยรับบ	เคยแต่เลิก
ครอบครัวข	สิทธิหลักประกันสุขภาพ (บัตรทอ	พุทธ	ไม่มี		เคยรับบริก	ไม่เคย
ครอบครัวเดี่	สิทธิข้าราชการ	พุทธ	ไม่มี		ไม่เคยรับบ	ไม่เคย
ครอบครัวข	สิทธิหลักประกันสุขภาพ (บัตรทอ	พุทธ	มี	ภูมิแพ้	ไม่เคยรับบ	เคยแต่เลิก
ครอบครัวเดี่	สิทธิหลักประกันสุขภาพ (บัตรทอ	พุทธ	ไม่มี		ไม่เคยรับบ	ไม่เคย
ครอบครัวเดี่	สิทธิหลักประกันสุขภาพ (บัตรทอ	พุทธ	ไม่มี		ไม่เคยรับบ	ไม่เคย
ครอบครัวข	สิทธิหลักประกันสุขภาพ (บัตรทอ	พุทธ	ไม่มี		ไม่เคยรับบ	ไม่เคย
ครอบครัวเดี่	สิทธิประกันสังคม	พุทธ	ไม่มี		ไม่เคยรับบ	ไม่เคย
ครอบครัวเดี่	สิทธิหลักประกันสุขภาพ (บัตรทอ	พุทธ	ไม่มี		ไม่เคยรับบ	ไม่เคย
ครอบครัวเดี่	สิทธิข้าราชการ	พุทธ	ไม่มี		ไม่เคยรับบ	ไม่เคย
ครอบครัวเดี่	สิทธิข้าราชการ	พุทธ	ไม่มี		เคยรับบริก	ไม่เคย
ครอบครัวเดี่	สิทธิหลักประกันสุขภาพ (บัตรทอ	พุทธ	ไม่มี		เคยรับบริก	ไม่เคย
ครอบครัวข	สิทธิประกันสังคม	พุทธ	ไม่มี		เคยรับบริก	เคยแต่เลิก
ครอบครัวเดี่	สิทธิหลักประกันสุขภาพ (บัตรทอ	พุทธ	ไม่มี		ไม่เคยรับบ	ไม่เคย
ครอบครัวข	สิทธิหลักประกันสุขภาพ (บัตรทอ	พุทธ	ไม่มี		เคยรับบริก	ไม่เคย
ครอบครัวข	สิทธิหลักประกันสุขภาพ (บัตรทอ	พุทธ	ไม่มี		เคยรับบริก	ไม่เคย
ครอบครัวเดี่	สิทธิประกันสังคม	พุทธ	ไม่มี		ไม่เคยรับบ	ไม่เคย
ครอบครัวข	สิทธิประกันสังคม	พุทธ	ไม่มี		ไม่เคยรับบ	ไม่เคย
ครอบครัวเดี่	สิทธิประกันสังคม	พุทธ	ไม่มี		ไม่เคยรับบ	ไม่เคย
ครอบครัวเดี่	สิทธิหลักประกันสุขภาพ (บัตรทอ	พุทธ	ไม่มี		ไม่เคยรับบ	ไม่เคย
ครอบครัวข	สิทธิประกันสังคม	พุทธ	ไม่มี		เคยรับบริก	ไม่เคย
ครอบครัวเดี่	สิทธิประกันสังคม	พุทธ	ไม่มี		ไม่เคยรับบ	ไม่เคย
ครอบครัวเดี่	สิทธิประกันสังคม	พุทธ	ไม่มี		เคยรับบริก	ไม่เคย
ครอบครัวข	สิทธิหลักประกันสุขภาพ (บัตรทอ	พุทธ	ไม่มี		เคยรับบริก	ไม่เคย
ครอบครัวเดี่	สิทธิหลักประกันสุขภาพ (บัตรทอ	พุทธ	ไม่มี		ไม่เคยรับบ	ไม่เคย
ครอบครัวเดี่	สิทธิข้าราชการ	พุทธ	ไม่มี		ไม่เคยรับบ	ไม่เคย
ครอบครัวข	สิทธิหลักประกันสุขภาพ (บัตรทอ	พุทธ	ไม่มี		ไม่เคยรับบ	ไม่เคย
ครอบครัวเดี่	สิทธิหลักประกันสุขภาพ (บัตรทอ	พุทธ	ไม่มี		ไม่เคยรับบ	เคยแต่เลิก
ครอบครัวเดี่	สิทธิหลักประกันสุขภาพ (บัตรทอ	พุทธ	ไม่มี		เคยรับบริก	ไม่เคย
ครอบครัวเดี่	สิทธิหลักประกันสุขภาพ (บัตรทอ	พุทธ	ไม่มี		เคยรับบริก	ไม่เคย
ครอบครัวเดี่	สิทธิหลักประกันสุขภาพ (บัตรทอ	พุทธ	ไม่มี		ไม่เคยรับบ	เคยแต่เลิก
ครอบครัวข	สิทธิหลักประกันสุขภาพ (บัตรทอ	พุทธ	ไม่มี		เคยรับบริก	ไม่เคย
ครอบครัวเดี่	สิทธิข้าราชการ	พุทธ	ไม่มี		ไม่เคยรับบ	ไม่เคย
ครอบครัวข	สิทธิหลักประกันสุขภาพ (บัตรทอ	พุทธ	ไม่มี		ไม่เคยรับบ	เคยแต่เลิก
ครอบครัวเดี่	สิทธิหลักประกันสุขภาพ (บัตรทอ	พุทธ	ไม่มี		ไม่เคยรับบ	ไม่เคย
ครอบครัวเดี่	สิทธิหลักประกันสุขภาพ (บัตรทอ	พุทธ	ไม่มี		ไม่เคยรับบ	ไม่เคย

ครอบครัวข	สิทธิหลักประกันสุขภาพ (บัตรทอ	พุทธ	ไม่มี	ไม่เคยรับบ	ไม่เคย
ครอบครัวเ	สิทธิประกันสังคม	พุทธ	ไม่มี	ไม่เคยรับบ	ไม่เคย
ครอบครัวเ	สิทธิหลักประกันสุขภาพ (บัตรทอ	พุทธ	ไม่มี	ไม่เคยรับบ	ไม่เคย
ครอบครัวเ	สิทธิข้าราชการ	พุทธ	ไม่มี	ไม่เคยรับบ	ไม่เคย
ครอบครัวเ	สิทธิข้าราชการ	พุทธ	ไม่มี	เคยรับบริก	ไม่เคย
ครอบครัวเ	สิทธิหลักประกันสุขภาพ (บัตรทอ	พุทธ	ไม่มี	เคยรับบริก	ไม่เคย
ครอบครัวเ	สิทธิหลักประกันสุขภาพ (บัตรทอ	พุทธ	ไม่มี	เคยรับบริก	ไม่เคย
ครอบครัวเ	สิทธิข้าราชการ	พุทธ	ไม่มี	เคยรับบริก	ไม่เคย
ครอบครัวเ	สิทธิหลักประกันสุขภาพ (บัตรทอ	พุทธ	ไม่มี	เคยรับบริก	ไม่เคย
ครอบครัวข	สิทธิประกันสังคม	พุทธ	ไม่มี	ไม่เคยรับบ	ไม่เคย
ครอบครัวเ	สิทธิประกันสังคม	พุทธ	ไม่มี	ไม่เคยรับบ	ไม่เคย
ครอบครัวเ	สิทธิหลักประกันสุขภาพ (บัตรทอ	พุทธ	ไม่มี	ไม่เคยรับบ	ไม่เคย
ครอบครัวข	สิทธิประกันสังคม	พุทธ	ไม่มี	เคยรับบริก	ไม่เคย
ครอบครัวเ	สิทธิประกันสังคม	พุทธ	ไม่มี	ไม่เคยรับบ	ไม่เคย
ครอบครัวเ	สิทธิประกันสังคม	พุทธ	ไม่มี	เคยรับบริก	ไม่เคย
ครอบครัวข	สิทธิหลักประกันสุขภาพ (บัตรทอ	พุทธ	ไม่มี	เคยรับบริก	ไม่เคย
ครอบครัวเ	สิทธิหลักประกันสุขภาพ (บัตรทอ	พุทธ	ไม่มี	ไม่เคยรับบ	ไม่เคย
ครอบครัวเ	สิทธิข้าราชการ	พุทธ	ไม่มี	ไม่เคยรับบ	ไม่เคย
ครอบครัวข	สิทธิหลักประกันสุขภาพ (บัตรทอ	พุทธ	ไม่มี	ไม่เคยรับบ	ไม่เคย
ครอบครัวเ	สิทธิหลักประกันสุขภาพ (บัตรทอ	พุทธ	ไม่มี	ไม่เคยรับบ	ไม่เคย
ครอบครัวเ	สิทธิหลักประกันสุขภาพ (บัตรทอ	พุทธ	ไม่มี	เคยรับบริก	ไม่เคย
ครอบครัวเ	สิทธิหลักประกันสุขภาพ (บัตรทอ	พุทธ	ไม่มี	เคยรับบริก	ไม่เคย
ครอบครัวเ	สิทธิหลักประกันสุขภาพ (บัตรทอ	พุทธ	ไม่มี	ไม่เคยรับบ	เคยแต่เลิก
ครอบครัวข	สิทธิหลักประกันสุขภาพ (บัตรทอ	พุทธ	ไม่มี	เคยรับบริก	ไม่เคย
ครอบครัวข	สิทธิหลักประกันสุขภาพ (บัตรทอ	พุทธ	ไม่มี	เคยรับบริก	ไม่เคย
ครอบครัวเ	สิทธิหลักประกันสุขภาพ (บัตรทอ	พุทธ	ไม่มี	เคยรับบริก	ไม่เคย
ครอบครัวเ	สิทธิข้าราชการ	พุทธ	ไม่มี	เคยรับบริก	ไม่เคย
ครอบครัวข	สิทธิหลักประกันสุขภาพ (บัตรทอ	พุทธ	ไม่มี	เคยรับบริก	ไม่เคย
ครอบครัวเ	สิทธิประกันสังคม	พุทธ	ไม่มี	เคยรับบริก	ไม่เคย
ครอบครัวข	สิทธิหลักประกันสุขภาพ (บัตรทอ	พุทธ	ไม่มี	เคยรับบริก	ไม่เคย
ครอบครัวเ	สิทธิหลักประกันสุขภาพ (บัตรทอ	พุทธ	ไม่มี	เคยรับบริก	ไม่เคย
ครอบครัวข	สิทธิหลักประกันสุขภาพ (บัตรทอ	พุทธ	ไม่มี	เคยรับบริก	ไม่เคย
ครอบครัวเ	สิทธิประกันสังคม	พุทธ	ไม่มี	เคยรับบริก	ไม่เคย
ครอบครัวข	สิทธิหลักประกันสุขภาพ (บัตรทอ	พุทธ	ไม่มี	เคยรับบริก	เคยแต่เลิก
ครอบครัวข	สิทธิหลักประกันสุขภาพ (บัตรทอ	พุทธ	ไม่มี	เคยรับบริก	เคยแต่เลิก
ครอบครัวข	สิทธิหลักประกันสุขภาพ (บัตรทอ	พุทธ	ไม่มี	เคยรับบริก	ไม่เคย
ครอบครัวข	สิทธิหลักประกันสุขภาพ (บัตรทอ	พุทธ	ไม่มี	ไม่เคยรับบ	ไม่เคย
ครอบครัวข	สิทธิหลักประกันสุขภาพ (บัตรทอ	พุทธ	ไม่มี	เคยรับบริก	ไม่เคย
ครอบครัวเ	สิทธิประกันสังคม	พุทธ	ไม่มี	เคยรับบริก	ไม่เคย
ครอบครัวข	สิทธิประกันสังคม	พุทธ	ไม่มี	เคยรับบริก	ไม่เคย
ครอบครัวเ	สิทธิหลักประกันสุขภาพ (บัตรทอ	พุทธ	ไม่มี	เคยรับบริก	ไม่เคย
ครอบครัวเ	สิทธิหลักประกันสุขภาพ (บัตรทอ	พุทธ	ไม่มี	เคยรับบริก	ไม่เคย
ครอบครัวเ	สิทธิข้าราชการ	พุทธ	ไม่มี	เคยรับบริก	ไม่เคย
ครอบครัวเ	สิทธิข้าราชการ	พุทธ	ไม่มี	ไม่เคยรับบ	ไม่เคย
ครอบครัวเ	สิทธิหลักประกันสุขภาพ (บัตรทอ	พุทธ	ไม่มี	เคยรับบริก	ไม่เคย
ครอบครัวเ	สิทธิหลักประกันสุขภาพ (บัตรทอ	พุทธ	ไม่มี	เคยรับบริก	ไม่เคย
ครอบครัวเ	สิทธิประกันสังคม	พุทธ	ไม่มี	เคยรับบริก	ไม่เคย
ครอบครัวข	สิทธิหลักประกันสุขภาพ (บัตรทอ	พุทธ	ไม่มี	เคยรับบริก	ไม่เคย
ครอบครัวข	สิทธิหลักประกันสุขภาพ (บัตรทอ	พุทธ	ไม่มี	เคยรับบริก	ไม่เคย
ครอบครัวเ	สิทธิหลักประกันสุขภาพ (บัตรทอ	พุทธ	ไม่มี	ไม่เคยรับบ	ไม่เคย
ครอบครัวเ	สิทธิหลักประกันสุขภาพ (บัตรทอ	พุทธ	ไม่มี	เคยรับบริก	ไม่เคย

ครอบครัวข	สิทธิหลักประกันสุขภาพ (บัตรทอ		พุทธ	ไม่มี	เคยรับบริก	ไม่เคย
ครอบครัวเ	สิทธิหลักประกันสุขภาพ (บัตรทอ		พุทธ	ไม่มี	เคยรับบริก	ไม่เคย
ครอบครัวเ	สิทธิประกันสังคม		พุทธ	ไม่มี	ไม่เคยรับบ	ไม่เคย
ครอบครัวข	สิทธิหลักประกันสุขภาพ (บัตรทอ		พุทธ	ไม่มี	ไม่เคยรับบ	ไม่เคย
ครอบครัวเ	สิทธิหลักประกันสุขภาพ (บัตรทอ		พุทธ	ไม่มี	ไม่เคยรับบ	ไม่เคย
ครอบครัวเ	สิทธิหลักประกันสุขภาพ (บัตรทอ		พุทธ	ไม่มี	ไม่เคยรับบ	เคยแต่เลิก
ครอบครัวเ	สิทธิหลักประกันสุขภาพ (บัตรทอ		พุทธ	ไม่มี	ไม่เคยรับบ	ไม่เคย
ครอบครัวเ	สิทธิหลักประกันสุขภาพ (บัตรทอ		พุทธ	ไม่มี	เคยรับบริก	ไม่เคย
ครอบครัวเ	สิทธิหลักประกันสุขภาพ (บัตรทอ		พุทธ	ไม่มี	เคยรับบริก	ไม่เคย
ครอบครัวเ	สิทธิประกันสังคม		พุทธ	ไม่มี	เคยรับบริก	ไม่เคย
ครอบครัวเ	สิทธิหลักประกันสุขภาพ (บัตรทอ		พุทธ	ไม่มี	เคยรับบริก	ไม่เคย
ครอบครัวเ	สิทธิหลักประกันสุขภาพ (บัตรทอ		พุทธ	ไม่มี	เคยรับบริก	ไม่เคย
ครอบครัวเ	สิทธิหลักประกันสุขภาพ (บัตรทอ		พุทธ	ไม่มี	เคยรับบริก	ไม่เคย
ครอบครัวเ	สิทธิประกันสังคม		พุทธ	ไม่มี	เคยรับบริก	ไม่เคย
ครอบครัวเ	สิทธิหลักประกันสุขภาพ (บัตรทอ		พุทธ	ไม่มี	ไม่เคยรับบ	ไม่เคย
ครอบครัวเ	สิทธิหลักประกันสุขภาพ (บัตรทอ		พุทธ	ไม่มี	เคยรับบริก	ไม่เคย
ครอบครัวข	สิทธิข้าราชการ		พุทธ	ไม่มี	ไม่เคยรับบ	ไม่เคย
ครอบครัวเ	สิทธิหลักประกันสุขภาพ (บัตรทอ		พุทธ	ไม่มี	เคยรับบริก	ไม่เคย
ครอบครัวเ	สิทธิหลักประกันสุขภาพ (บัตรทอ		พุทธ	ไม่มี	ไม่เคยรับบ	ไม่เคย
ครอบครัวเ	สิทธิหลักประกันสุขภาพ (บัตรทอ		พุทธ	ไม่มี	เคยรับบริก	ไม่เคย
ครอบครัวเ	สิทธิหลักประกันสุขภาพ (บัตรทอ		พุทธ	ไม่มี	ไม่เคยรับบ	ไม่เคย
ครอบครัวเ	สิทธิหลักประกันสุขภาพ (บัตรทอ		พุทธ	ไม่มี	ไม่เคยรับบ	ไม่เคย
ครอบครัวเ	สิทธิหลักประกันสุขภาพ (บัตรทอ		พุทธ	ไม่มี	เคยรับบริก	ไม่เคย
ครอบครัวเ	สิทธิหลักประกันสุขภาพ (บัตรทอ		พุทธ	ไม่มี	เคยรับบริก	ไม่เคย
ครอบครัวเ	สิทธิหลักประกันสุขภาพ (บัตรทอ		พุทธ	ไม่มี	ไม่เคยรับบ	ไม่เคย
ครอบครัวเ	สิทธิหลักประกันสุขภาพ (บัตรทอ		พุทธ	ไม่มี	เคยรับบริก	ไม่เคย
ครอบครัวข	สิทธิหลักประกันสุขภาพ (บัตรทอ		พุทธ	ไม่มี	ไม่เคยรับบ	ไม่เคย
ครอบครัวเ	สิทธิหลักประกันสุขภาพ (บัตรทอ		พุทธ	ไม่มี	เคยรับบริก	ไม่เคย
ครอบครัวข	สิทธิหลักประกันสุขภาพ (บัตรทอ		พุทธ	ไม่มี	ไม่เคยรับบ	ไม่เคย
ครอบครัวเ	อื่นๆ	จ่ายเอง	พุทธ	ไม่มี	ไม่เคยรับบ	ไม่เคย
ครอบครัวเ	สิทธิหลักประกันสุขภาพ (บัตรทอ		พุทธ	ไม่มี	ไม่เคยรับบ	ไม่เคย
ครอบครัวเ	สิทธิหลักประกันสุขภาพ (บัตรทอ		พุทธ	ไม่มี	เคยรับบริก	ไม่เคย
ครอบครัวข	สิทธิหลักประกันสุขภาพ (บัตรทอ		พุทธ	ไม่มี	ไม่เคยรับบ	ไม่เคย
ครอบครัวเ	สิทธิหลักประกันสุขภาพ (บัตรทอ		พุทธ	ไม่มี	ไม่เคยรับบ	ไม่เคย
ครอบครัวเ	สิทธิหลักประกันสุขภาพ (บัตรทอ		พุทธ	ไม่มี	ไม่เคยรับบ	ไม่เคย
ครอบครัวข	สิทธิหลักประกันสุขภาพ (บัตรทอ		พุทธ	ไม่มี	เคยรับบริก	ไม่เคย
ครอบครัวข	สิทธิหลักประกันสุขภาพ (บัตรทอ		พุทธ	ไม่มี	เคยรับบริก	ไม่เคย
ครอบครัวเ	สิทธิหลักประกันสุขภาพ (บัตรทอ		พุทธ	ไม่มี	เคยรับบริก	ไม่เคย
ครอบครัวเ	สิทธิหลักประกันสุขภาพ (บัตรทอ		พุทธ	ไม่มี	เคยรับบริก	ไม่เคย
ครอบครัวข	สิทธิหลักประกันสุขภาพ (บัตรทอ		พุทธ	ไม่มี	เคยรับบริก	ไม่เคย
ครอบครัวเ	สิทธิหลักประกันสุขภาพ (บัตรทอ		พุทธ	ไม่มี	ไม่เคยรับบ	ไม่เคย
ครอบครัวเ	สิทธิหลักประกันสุขภาพ (บัตรทอ		พุทธ	ไม่มี	เคยรับบริก	ไม่เคย
ครอบครัวข	สิทธิหลักประกันสุขภาพ (บัตรทอ		พุทธ	ไม่มี	เคยรับบริก	ไม่เคย
ครอบครัวเ	สิทธิหลักประกันสุขภาพ (บัตรทอ		พุทธ	ไม่มี	เคยรับบริก	ไม่เคย
ครอบครัวเ	สิทธิประกันสังคม		พุทธ	ไม่มี	ไม่เคยรับบ	ไม่เคย
ครอบครัวข	สิทธิหลักประกันสุขภาพ (บัตรทอ		พุทธ	ไม่มี	ไม่เคยรับบ	ไม่เคย
ครอบครัวเ	สิทธิข้าราชการ		พุทธ	ไม่มี	เคยรับบริก	ไม่เคย
ครอบครัวเ	สิทธิข้าราชการ		พุทธ	ไม่มี	เคยรับบริก	ไม่เคย
ครอบครัวข	สิทธิหลักประกันสุขภาพ (บัตรทอ		พุทธ	ไม่มี	ไม่เคยรับบ	ไม่เคย

ดื่มเหล้า	G	P	A	L	ภาวะเสี่ยง	person16_	suscept1	suscept2
ไม่เคย	1	0	0	0	ไม่มี		เห็นด้วย	ไม่เห็นด้วย
ไม่เคย	1	0	0	0	ไม่มี		เห็นด้วย	เห็นด้วย
เคยแต่เลิกเ	3	2	0	2	ไม่มี		ไม่เห็นด้วย	ไม่เห็นด้วยอ
ไม่เคย	3	1	0	0	ไม่มี		ไม่เห็นด้วย	ไม่เห็นด้วย
ไม่เคย	2	1	0	1	ไม่มี		เห็นด้วย	เห็นด้วย
ไม่เคย	1	0	0	0	ไม่มี		เห็นด้วย	เห็นด้วย
ไม่เคย	2	1	0	1	ไม่มี		เห็นด้วย	ไม่เห็นด้วย
ไม่เคย	3	1	1	1	ไม่มี		เห็นด้วย	เห็นด้วยอย่
เคยแต่เลิกเ	1	0	0	0	ไม่มี		เห็นด้วยอย่	เห็นด้วยอย่
ไม่เคย	1	0	0	0	ไม่มี		เห็นด้วย	เห็นด้วยอย่
เคยแต่เลิกเ	4	3	0	3	ไม่มี		เห็นด้วย	เห็นด้วย
ไม่เคย	2	1	0	1	ไม่มี		เห็นด้วย	เห็นด้วย
ไม่เคย	1	0	0	0	ไม่มี		เห็นด้วย	เห็นด้วย
เคยแต่เลิกเ	1	0	0	0	ไม่มี		เห็นด้วย	เห็นด้วย
เคยแต่เลิกเ	1	0	0	0	ไม่มี		เห็นด้วย	เห็นด้วย
ไม่เคย	1	0	0	0	ไม่มี		เห็นด้วย	เห็นด้วย
เคยแต่เลิกเ	1	0	0	0	ไม่มี		เห็นด้วย	เห็นด้วยอย่
เคยแต่เลิกเ	2	1	0	1	ไม่มี		เห็นด้วย	เห็นด้วย
ไม่เคย	1	0	0	0	ไม่มี		เห็นด้วยอย่	เห็นด้วย
เคยแต่เลิกเ	1	0	0	0	ไม่มี		เห็นด้วย	เห็นด้วย
เคยแต่เลิกเ	1	0	0	0	ไม่มี		เห็นด้วย	เห็นด้วย
ไม่เคย	2	1	0	1	ไม่มี		ไม่เห็นด้วยอ	ไม่เห็นด้วย
ไม่เคย	2	1	0	1	ไม่มี		ไม่เห็นด้วย	เห็นด้วย
ไม่เคย	1	0	0	0	ไม่มี		เห็นด้วย	เห็นด้วยอย่
เคยแต่เลิกเ	2	1	0	1	ไม่มี		เห็นด้วย	เห็นด้วย
เคยแต่เลิกเ	2	1	1	1	ไม่มี		เห็นด้วย	เห็นด้วย
เคยแต่เลิกเ	1	0	0	0	ไม่มี		เห็นด้วย	เห็นด้วย
ไม่เคย	1	0	0	0	ไม่มี		ไม่เห็นด้วยอ	ไม่เห็นด้วยอ
เคยแต่เลิกเ	1	0	0	0	ไม่มี		ไม่เห็นด้วย	เห็นด้วย
เคยแต่เลิกเ	2	1	0	1	ไม่มี		ไม่เห็นด้วย	ไม่เห็นด้วย
เคยแต่เลิกเ	1	0	0	0	ไม่มี		เห็นด้วย	เห็นด้วย
เคยแต่เลิกเ	1	0	0	0	ไม่มี		เห็นด้วยอย่	เห็นด้วยอย่
ไม่เคย	2	1	0	1	ไม่มี		เห็นด้วย	เห็นด้วย
เคยแต่เลิกเ	2	0	1	0	ไม่มี		เห็นด้วยอย่	ไม่เห็นด้วย
เคยแต่เลิกเ	1	0	0	0	ไม่มี		เห็นด้วย	เห็นด้วย
เคยแต่เลิกเ	2	1	0	1	ไม่มี		เห็นด้วย	เห็นด้วย
เคยแต่เลิกเ	1	0	0	0	ไม่มี		เห็นด้วย	เห็นด้วย
ไม่เคย	1	0	0	0	ไม่มี		เห็นด้วย	เห็นด้วย
เคยแต่เลิกเ	2	1	0	1	ไม่มี		เห็นด้วย	เห็นด้วย
ไม่เคย	1	0	0	0	ไม่มี		เห็นด้วย	เห็นด้วย
เคยแต่เลิกเ	2	1	0	1	ไม่มี		ไม่เห็นด้วย	ไม่เห็นด้วย
ไม่เคย	3	2	0	2	ไม่มี		เห็นด้วย	เห็นด้วย
เคย และปัจ	1	0	0	0	ไม่มี		เห็นด้วย	เห็นด้วย
เคยแต่เลิกเ	1	0	0	0	ไม่มี		เห็นด้วยอย่	เห็นด้วยอย่
เคยแต่เลิกเ	1	0	0	0	ไม่มี		เห็นด้วย	เห็นด้วย
เคยแต่เลิกเ	1	0	0	0	ไม่มี		เห็นด้วยอย่	ไม่เห็นด้วย
เคยแต่เลิกเ	1	0	0	0	ไม่มี		ไม่เห็นด้วย	เห็นด้วย
เคยแต่เลิกเ	2	1	0	1	ไม่มี		เห็นด้วย	เห็นด้วย
เคยแต่เลิกเ	2	1	0	1	ไม่มี		เห็นด้วย	เห็นด้วย
เคยแต่เลิกเ	2	1	0	1	ไม่มี		เห็นด้วย	เห็นด้วย

เคยแต่เลิกแ	1	0	0	0	ไม่มี	เห็นด้วย	เห็นด้วย
ไม่เคย	3	2	0	2	ไม่มี	เห็นด้วยอย่	เห็นด้วยอย่
ไม่เคย	1	0	0	0	ไม่มี	เห็นด้วย	เห็นด้วย
ไม่เคย	1	0	0	0	ไม่มี	เห็นด้วย	เห็นด้วย
เคย และปัจ	1	0	1	1	ไม่มี	เห็นด้วยอย่	เห็นด้วยอย่
ไม่เคย	1	0	0	0	ไม่มี	เห็นด้วย	เห็นด้วย
ไม่เคย	2	1	0	1	ไม่มี	เห็นด้วย	เห็นด้วย
เคยแต่เลิกแ	1	0	0	0	ไม่มี	เห็นด้วย	เห็นด้วยอย่
เคยแต่เลิกแ	1	0	0	0	ไม่มี	เห็นด้วย	เห็นด้วย
ไม่เคย	2	1	0	1	ไม่มี	ไม่เห็นด้วย	เห็นด้วย
เคยแต่เลิกแ	3	2	0	2	ไม่มี	เห็นด้วย	เห็นด้วย
เคยแต่เลิกแ	2	1	0	1	ไม่มี	เห็นด้วย	เห็นด้วยอย่
ไม่เคย	2	1	0	1	ไม่มี	ไม่เห็นด้วยa	ไม่เห็นด้วยa
เคยแต่เลิกแ	2	1	0	1	ไม่มี	เห็นด้วยอย่	เห็นด้วยอย่
เคยแต่เลิกแ	1	0	0	0	มี ได้แก่.... เบาหวาน	เห็นด้วย	เห็นด้วยอย่
ไม่เคย	1	0	0	0	ไม่มี	เห็นด้วย	เห็นด้วย
ไม่เคย	2	0	1	0	ไม่มี	เห็นด้วย	เห็นด้วย
ไม่เคย	2	1	0	1	ไม่มี	เห็นด้วย	เห็นด้วย
ไม่เคย	1	0	0	0	ไม่มี	เห็นด้วย	เห็นด้วย
ไม่เคย	1	0	0	0	ไม่มี	เห็นด้วย	เห็นด้วย
เคยแต่เลิกแ	1	0	0	0	มี ได้แก่.... ดาวซินโดระ	เห็นด้วย	เห็นด้วย
ไม่เคย	3	2	0	2	ไม่มี	เห็นด้วย	เห็นด้วยอย่
ไม่เคย	2	1	0	1	มี ได้แก่.... อายุเยอะ	ไม่เห็นด้วย	เห็นด้วยอย่
ไม่เคย	2	1	0	1	มี ได้แก่.... อายุเยอะ	เห็นด้วย	เห็นด้วย
เคยแต่เลิกแ	1	0	0	0	ไม่มี	เห็นด้วย	เห็นด้วย
ไม่เคย	3	2	0	2	ไม่มี	ไม่เห็นด้วย	ไม่เห็นด้วย
ไม่เคย	1	0	0	0	ไม่มี	ไม่เห็นด้วย	ไม่เห็นด้วย
ไม่เคย	1	0	0	0	ไม่มี	เห็นด้วยอย่	เห็นด้วย
เคยแต่เลิกแ	1	0	0	0	ไม่มี	เห็นด้วยอย่	เห็นด้วยอย่
ไม่เคย	2	1	0	1	ไม่มี	เห็นด้วย	เห็นด้วย
ไม่เคย	5	4	0	4	มี ได้แก่.... อายุเยอะ	เห็นด้วย	เห็นด้วย
ไม่เคย	2	1	0	1	ไม่มี	เห็นด้วย	เห็นด้วย
ไม่เคย	1	0	0	0	ไม่มี	เห็นด้วย	เห็นด้วย
ไม่เคย	1	0	0	0	ไม่มี	เห็นด้วย	เห็นด้วย
ไม่เคย	3	2	0	2	ไม่มี	เห็นด้วย	เห็นด้วย
ไม่เคย	2	1	0	1	ไม่มี	เห็นด้วย	เห็นด้วย
ไม่เคย	1	0	0	0	ไม่มี	เห็นด้วย	เห็นด้วย
เคยแต่เลิกแ	1	0	0	0	ไม่มี	ไม่เห็นด้วย	ไม่เห็นด้วย
ไม่เคย	1	0	0	0	ไม่มี	เห็นด้วย	เห็นด้วย
ไม่เคย	2	1	0	1	ไม่มี	เห็นด้วย	เห็นด้วย
ไม่เคย	3	2	0	2	ไม่มี	เห็นด้วย	เห็นด้วย
ไม่เคย	2	1	0	1	ไม่มี	เห็นด้วย	เห็นด้วย
เคยแต่เลิกแ	1	0	0	0	ไม่มี	เห็นด้วย	เห็นด้วย
ไม่เคย	3	2	0	2	ไม่มี	เห็นด้วย	เห็นด้วย
เคยแต่เลิกแ	3	2	0	2	ไม่มี	เห็นด้วย	เห็นด้วย
ไม่เคย	1	0	0	0	ไม่มี	ไม่เห็นด้วย	เห็นด้วย
ไม่เคย	2	1	0	1	ไม่มี	เห็นด้วย	เห็นด้วย
เคยแต่เลิกแ	1	0	0	0	ไม่มี	เห็นด้วย	เห็นด้วยอย่
เคยแต่เลิกแ	3	2	0	2	ไม่มี	ไม่เห็นด้วยa	เห็นด้วย
ไม่เคย	1	0	0	0	ไม่มี	เห็นด้วย	เห็นด้วย
ไม่เคย	2	1	0	1	ไม่มี	เห็นด้วย	เห็นด้วย

เคยแต่เลิกเ	2	1	0	1	ไม่มี	เห็นด้วย	เห็นด้วย
ไม่เคย	5	2	2	2	ไม่มี	เห็นด้วย	เห็นด้วย
เคยแต่เลิกเ	3	0	2	0	ไม่มี	ไม่เห็นด้วย	เห็นด้วย
เคยแต่เลิกเ	4	2	1	2	ไม่มี	เห็นด้วยอย่	เห็นด้วยอย่
ไม่เคย	2	1	0	1	ไม่มี	เห็นด้วย	เห็นด้วย
ไม่เคย	2	1	0	1	ไม่มี	เห็นด้วย	เห็นด้วย
เคยแต่เลิกเ	1	0	0	0	ไม่มี	ไม่เห็นด้วย	ไม่เห็นด้วย
ไม่เคย	1	0	0	0	ไม่มี	เห็นด้วย	เห็นด้วย
ไม่เคย	1	0	0	0	ไม่มี	เห็นด้วย	เห็นด้วยอย่
เคยแต่เลิกเ	1	0	0	0	ไม่มี	ไม่เห็นด้วย	ไม่เห็นด้วย
ไม่เคย	1	0	0	0	ไม่มี	ไม่เห็นด้วย	เห็นด้วย
เคยแต่เลิกเ	1	0	0	0	ไม่มี	ไม่เห็นด้วย	ไม่เห็นด้วย
เคยแต่เลิกเ	4	2	1	2	ไม่มี	เห็นด้วย	ไม่เห็นด้วย
เคยแต่เลิกเ	3	2	0	2	ไม่มี	เห็นด้วย	เห็นด้วยอย่
เคยแต่เลิกเ	4	2	4	2	มี ได้แก่.... อายุเยอะ	เห็นด้วย	เห็นด้วย
เคยแต่เลิกเ	3	2	1	1	ไม่มี	เห็นด้วย	เห็นด้วย
เคยแต่เลิกเ	1	0	0	0	ไม่มี	เห็นด้วย	เห็นด้วย
เคยแต่เลิกเ	2	1	0	1	ไม่มี	เห็นด้วย	เห็นด้วย
ไม่เคย	2	2	0	2	ไม่มี	เห็นด้วย	เห็นด้วย
เคยแต่เลิกเ	1	0	0	0	ไม่มี	เห็นด้วย	เห็นด้วย
เคยแต่เลิกเ	1	0	0	0	ไม่มี	เห็นด้วย	เห็นด้วย
เคยแต่เลิกเ	1	0	0	0	ไม่มี	เห็นด้วย	เห็นด้วย
เคยแต่เลิกเ	1	0	0	0	ไม่มี	ไม่เห็นด้วย	เห็นด้วย
ไม่เคย	2	2	1	1	ไม่มี	เห็นด้วย	เห็นด้วย
ไม่เคย	1	0	0	0	ไม่มี	ไม่เห็นด้วย	เห็นด้วย
เคยแต่เลิกเ	1	0	0	0	ไม่มี	เห็นด้วย	เห็นด้วย
ไม่เคย	2	0	1	0	ไม่มี	ไม่เห็นด้วยอ	ไม่เห็นด้วย
ไม่เคย	2	1	0	1	ไม่มี	เห็นด้วย	เห็นด้วย
ไม่เคย	1	1	0	1	ไม่มี	ไม่เห็นด้วย	ไม่เห็นด้วย
เคยแต่เลิกเ	1	1	0	1	ไม่มี	เห็นด้วย	เห็นด้วย
ไม่เคย	2	1	0	1	ไม่มี	เห็นด้วย	เห็นด้วย
ไม่เคย	1	0	0	0	ไม่มี	เห็นด้วย	เห็นด้วย
เคยแต่เลิกเ	2	1	0	1	ไม่มี	เห็นด้วย	เห็นด้วย
เคยแต่เลิกเ	1	0	0	0	ไม่มี	ไม่เห็นด้วย	เห็นด้วย
เคยแต่เลิกเ	1	0	0	0	ไม่มี	เห็นด้วย	เห็นด้วย
เคยแต่เลิกเ	3	2	0	2	ไม่มี	ไม่เห็นด้วย	เห็นด้วย
ไม่เคย	1	0	0	0	ไม่มี	เห็นด้วย	เห็นด้วยอย่
ไม่เคย	1	0	0	0	ไม่มี	เห็นด้วยอย่	ไม่เห็นด้วย
เคยแต่เลิกเ	1	0	0	0	ไม่มี	เห็นด้วย	เห็นด้วย
เคยแต่เลิกเ	2	1	0	1	ไม่มี	ไม่เห็นด้วย	เห็นด้วย
เคยแต่เลิกเ	2	1	0	1	ไม่มี	เห็นด้วย	เห็นด้วย
เคยแต่เลิกเ	1	0	0	0	ไม่มี	เห็นด้วย	เห็นด้วย
ไม่เคย	2	1	0	1	ไม่มี	ไม่เห็นด้วย	เห็นด้วย
เคยแต่เลิกเ	4	3	0	3	ไม่มี	เห็นด้วย	เห็นด้วย
เคยแต่เลิกเ	1	0	0	0	ไม่มี	เห็นด้วยอย่	เห็นด้วย
เคยแต่เลิกเ	1	0	0	0	ไม่มี	ไม่เห็นด้วย	ไม่เห็นด้วย
เคยแต่เลิกเ	3	2	0	2	ไม่มี	เห็นด้วย	เห็นด้วย
ไม่เคย	1	0	0	0	ไม่มี	ไม่เห็นด้วย	ไม่เห็นด้วย
ไม่เคย	3	1	1	1	ไม่มี	เห็นด้วย	เห็นด้วย
ไม่เคย	2	1	1	1	ไม่มี	เห็นด้วย	เห็นด้วย
ไม่เคย	2	1	0	1	ไม่มี	เห็นด้วย	เห็นด้วย

ไม่เคย	1	0	0	0	ไม่มี	เห็นด้วย	เห็นด้วย
เคยแต่เลิกแ	2	1	0	1	ไม่มี	เห็นด้วย	เห็นด้วย
เคยแต่เลิกแ	1	0	0	0	ไม่มี	ไม่เห็นด้วย	เห็นด้วย
เคยแต่เลิกแ	2	1	0	1	ไม่มี	เห็นด้วย	เห็นด้วย
ไม่เคย	2	1	0	1	ไม่มี	เห็นด้วย	เห็นด้วย
เคยแต่เลิกแ	1	0	0	0	ไม่มี	เห็นด้วย	เห็นด้วย
ไม่เคย	2	1	0	1	ไม่มี	ไม่เห็นด้วย	ไม่เห็นด้วย
ไม่เคย	4	3	1	3	ไม่มี	เห็นด้วย	เห็นด้วย
ไม่เคย	2	1	0	1	มี ได้แก่.... พาหะธาลัส	เห็นด้วย	เห็นด้วย
เคยแต่เลิกแ	3	2	0	2	ไม่มี	เห็นด้วย	เห็นด้วย
เคยแต่เลิกแ	3	2	0	2	ไม่มี	เห็นด้วย	เห็นด้วย
เคยแต่เลิกแ	1	0	0	0	ไม่มี	ไม่เห็นด้วย	ไม่เห็นด้วย
เคยแต่เลิกแ	1	0	0	0	ไม่มี	เห็นด้วย	เห็นด้วย
ไม่เคย	2	1	0	1	มี ได้แก่.... คลอดก่อนก	เห็นด้วยอย่	เห็นด้วยอย่
ไม่เคย	2	1	0	1	ไม่มี	ไม่เห็นด้วย	ไม่เห็นด้วย
เคยแต่เลิกแ	1	0	0	0	ไม่มี	เห็นด้วย	เห็นด้วย
ไม่เคย	6	2	3	2	มี ได้แก่.... แท้ง	ไม่เห็นด้วย	ไม่เห็นด้วย
เคยแต่เลิกแ	1	0	0	0	ไม่มี	ไม่เห็นด้วย	เห็นด้วย
เคยแต่เลิกแ	1	0	0	0	ไม่มี	เห็นด้วย	เห็นด้วย
เคยแต่เลิกแ	1	0	0	0	ไม่มี	เห็นด้วย	เห็นด้วยอย่
เคยแต่เลิกแ	2	1	0	1	มี ได้แก่.... น้ำหนักเกิน	เห็นด้วย	เห็นด้วย
เคยแต่เลิกแ	2	1	0	1	ไม่มี	เห็นด้วย	เห็นด้วย
เคยแต่เลิกแ	1	0	0	0	ไม่มี	เห็นด้วย	เห็นด้วย
เคยแต่เลิกแ	1	0	0	0	ไม่มี	เห็นด้วย	เห็นด้วยอย่
ไม่เคย	4	3	0	3	ไม่มี	เห็นด้วย	เห็นด้วย
ไม่เคย	1	0	0	0	ไม่มี	เห็นด้วย	เห็นด้วย
ไม่เคย	2	1	0	1	ไม่มี	เห็นด้วย	เห็นด้วยอย่
ไม่เคย	1	0	0	0	ไม่มี	เห็นด้วย	เห็นด้วย
ไม่เคย	1	0	0	0	ไม่มี	เห็นด้วย	เห็นด้วย
เคยแต่เลิกแ	1	0	0	0	ไม่มี	เห็นด้วย	เห็นด้วยอย่
เคยแต่เลิกแ	2	1	0	1	ไม่มี	เห็นด้วย	เห็นด้วย
เคยแต่เลิกแ	1	0	0	0	ไม่มี	เห็นด้วย	เห็นด้วย
เคยแต่เลิกแ	3	2	0	2	ไม่มี	เห็นด้วย	เห็นด้วย
ไม่เคย	1	0	0	0	ไม่มี	เห็นด้วย	เห็นด้วย
เคยแต่เลิกแ	1	0	0	0	ไม่มี	ไม่เห็นด้วยอ	ไม่เห็นด้วย
ไม่เคย	1	0	0	0	ไม่มี	เห็นด้วย	เห็นด้วย
ไม่เคย	1	0	0	0	ไม่มี	เห็นด้วย	เห็นด้วย
เคยแต่เลิกแ	2	1	0	1	ไม่มี	เห็นด้วยอย่	เห็นด้วยอย่
ไม่เคย	3	2	0	2	ไม่มี	เห็นด้วย	เห็นด้วย
ไม่เคย	2	1	0	2	ไม่มี	เห็นด้วย	ไม่เห็นด้วย
เคยแต่เลิกแ	2	1	0	1	ไม่มี	เห็นด้วย	เห็นด้วย
เคยแต่เลิกแ	1	0	0	0	ไม่มี	เห็นด้วยอย่	เห็นด้วยอย่
เคยแต่เลิกแ	1	0	0	0	ไม่มี	เห็นด้วยอย่	เห็นด้วย
ไม่เคย	2	1	0	1	ไม่มี	เห็นด้วย	เห็นด้วย
ไม่เคย	2	1	0	1	ไม่มี	ไม่เห็นด้วยอ	ไม่เห็นด้วยอ
เคยแต่เลิกแ	2	1	0	1	ไม่มี	เห็นด้วย	เห็นด้วย
เคยแต่เลิกแ	3	1	1	2	ไม่มี	เห็นด้วย	เห็นด้วยอย่
ไม่เคย	2	1	0	1	ไม่มี	เห็นด้วย	เห็นด้วย
เคยแต่เลิกแ	1	0	0	0	ไม่มี	เห็นด้วยอย่	เห็นด้วยอย่
เคยแต่เลิกแ	1	0	0	0	ไม่มี	เห็นด้วย	เห็นด้วย
เคยแต่เลิกแ	1	0	0	0	ไม่มี	เห็นด้วย	เห็นด้วย

เคยแต่เลิกเ	2	1	0	1	ไม่มี	เห็นด้วย	เห็นด้วย
เคยแต่เลิกเ	2	1	0	1	ไม่มี	เห็นด้วย	เห็นด้วย
เคยแต่เลิกเ	1	0	0	0	ไม่มี	เห็นด้วย	เห็นด้วย
เคยแต่เลิกเ	1	0	0	0	ไม่มี	เห็นด้วย	เห็นด้วย
ไม่เคย	3	1	1	1	ไม่มี	เห็นด้วย	เห็นด้วย
ไม่เคย	3	2	0	2	ไม่มี	เห็นด้วยอย่	เห็นด้วยอย่
ไม่เคย	2	1	0	1	ไม่มี	เห็นด้วย	เห็นด้วย
เคยแต่เลิกเ	2	1	0	1	ไม่มี	ไม่เห็นด้วย	เห็นด้วย
ไม่เคย	3	2	0	2	ไม่มี	เห็นด้วย	เห็นด้วย
เคยแต่เลิกเ	2	1	0	1	ไม่มี	เห็นด้วยอย่	เห็นด้วยอย่
ไม่เคย	5	4	0	4	ไม่มี	เห็นด้วย	เห็นด้วย
ไม่เคย	2	1	0	1	ไม่มี	เห็นด้วยอย่	เห็นด้วย
ไม่เคย	3	2	0	2	ไม่มี	เห็นด้วยอย่	เห็นด้วย
ไม่เคย	1	0	0	0	ไม่มี	เห็นด้วย	เห็นด้วย
ไม่เคย	1	0	0	0	ไม่มี	เห็นด้วย	เห็นด้วย
ไม่เคย	2	1	0	1	ไม่มี	เห็นด้วย	เห็นด้วย
ไม่เคย	2	1	0	1	ไม่มี	เห็นด้วยอย่	เห็นด้วย
ไม่เคย	1	0	0	0	ไม่มี	เห็นด้วย	ไม่เห็นด้วย
ไม่เคย	2	1	0	1	ไม่มี	เห็นด้วยอย่	เห็นด้วยอย่
เคยแต่เลิกเ	2	1	0	1	ไม่มี	เห็นด้วย	เห็นด้วยอย่
ไม่เคย	1	0	0	0	ไม่มี	ไม่เห็นด้วย	เห็นด้วย
ไม่เคย	1	0	0	0	ไม่มี	เห็นด้วย	เห็นด้วย
ไม่เคย	4	3	1	3	ไม่มี	ไม่เห็นด้วย	ไม่เห็นด้วย
เคยแต่เลิกเ	1	0	0	1	ไม่มี	เห็นด้วยอย่	เห็นด้วย
ไม่เคย	1	0	0	0	ไม่มี	เห็นด้วย	เห็นด้วยอย่
เคยแต่เลิกเ	3	2	0	2	ไม่มี	เห็นด้วย	เห็นด้วย
เคยแต่เลิกเ	1	0	0	0	ไม่มี	เห็นด้วย	เห็นด้วยอย่
ไม่เคย	2	1	0	1	ไม่มี	เห็นด้วย	เห็นด้วย
ไม่เคย	2	1	0	1	ไม่มี	เห็นด้วยอย่	เห็นด้วย
ไม่เคย	2	1	0	1	ไม่มี	เห็นด้วยอย่	เห็นด้วยอย่
ไม่เคย	1	0	0	0	ไม่มี	เห็นด้วย	เห็นด้วย
เคยแต่เลิกเ	2	1	0	0	ไม่มี	เห็นด้วย	เห็นด้วยอย่
ไม่เคย	4	1	2	1	ไม่มี	ไม่เห็นด้วย	ไม่เห็นด้วย
เคยแต่เลิกเ	3	2	0	2	ไม่มี	ไม่เห็นด้วย	ไม่เห็นด้วย
เคยแต่เลิกเ	1	0	0	0	ไม่มี	เห็นด้วยอย่	เห็นด้วยอย่
ไม่เคย	1	0	0	0	ไม่มี	เห็นด้วยอย่	เห็นด้วยอย่
ไม่เคย	1	0	0	0	ไม่มี	เห็นด้วย	เห็นด้วยอย่
เคยแต่เลิกเ	2	1	0	1	ไม่มี	ไม่เห็นด้วย	เห็นด้วย
เคยแต่เลิกเ	1	0	0	0	ไม่มี	เห็นด้วย	เห็นด้วยอย่
เคยแต่เลิกเ	3	2	0	2	ไม่มี	เห็นด้วย	เห็นด้วย
ไม่เคย	2	1	0	1	ไม่มี	เห็นด้วยอย่	เห็นด้วย
ไม่เคย	1	0	0	0	ไม่มี	เห็นด้วย	เห็นด้วย
ไม่เคย	1	0	0	0	ไม่มี	ไม่เห็นด้วย	เห็นด้วยอย่
ไม่เคย	2	1	0	0	ไม่มี	เห็นด้วย	เห็นด้วย
ไม่เคย	1	0	0	0	ไม่มี	ไม่เห็นด้วย	เห็นด้วย
เคยแต่เลิกเ	1	0	0	0	ไม่มี	เห็นด้วย	เห็นด้วย
ไม่เคย	1	0	0	0	ไม่มี	เห็นด้วยอย่	เห็นด้วยอย่
ไม่เคย	1	0	0	0	ไม่มี	เห็นด้วยอย่	เห็นด้วยอย่
ไม่เคย	1	0	0	0	ไม่มี	เห็นด้วยอย่	เห็นด้วย
เคยแต่เลิกเ	1	0	0	0	ไม่มี	เห็นด้วย	เห็นด้วย
เคยแต่เลิกเ	1	0	0	0	ไม่มี	เห็นด้วย	เห็นด้วย

เคยแต่เลิกแ	2	1	0	1	ไม่มี	เห็นด้วย	ไม่เห็นด้วย
ไม่เคย	3	2	0	2	ไม่มี	เห็นด้วย	ไม่เห็นด้วย
เคยแต่เลิกแ	2	1	0	1	มี ได้แก่.... อายุเยอะ	เห็นด้วย	เห็นด้วย
เคยแต่เลิกแ	2	0	1	0	ไม่มี	ไม่เห็นด้วย	ไม่เห็นด้วย
เคยแต่เลิกแ	1	0	0	0	ไม่มี	เห็นด้วย	เห็นด้วย
เคยแต่เลิกแ	1	0	0	0	ไม่มี	เห็นด้วยอย่	เห็นด้วยอย่
ไม่เคย	1	0	0	0	ไม่มี	เห็นด้วย	เห็นด้วยอย่
เคยแต่เลิกแ	1	0	0	1	ไม่มี	เห็นด้วย	เห็นด้วย
เคยแต่เลิกแ	1	0	0	0	ไม่มี	ไม่เห็นด้วย	เห็นด้วย
ไม่เคย	2	1	0	1	ไม่มี	ไม่เห็นด้วย	ไม่เห็นด้วย
เคยแต่เลิกแ	1	0	0	0	ไม่มี	เห็นด้วย	เห็นด้วย
ไม่เคย	3	2	0	2	ไม่มี	ไม่เห็นด้วย	เห็นด้วย
ไม่เคย	4	3	1	2	ไม่มี	ไม่เห็นด้วย	ไม่เห็นด้วย
เคยแต่เลิกแ	6	2	3	2	ไม่มี	ไม่เห็นด้วย	ไม่เห็นด้วย
เคยแต่เลิกแ	1	0	0	0	ไม่มี	เห็นด้วย	เห็นด้วย
เคยแต่เลิกแ	1	0	0	0	ไม่มี	ไม่เห็นด้วย	ไม่เห็นด้วย
เคยแต่เลิกแ	1	0	0	0	ไม่มี	ไม่เห็นด้วย	เห็นด้วย
เคยแต่เลิกแ	2	1	0	2	มี ได้แก่.... อายุเยอะ	เห็นด้วย	เห็นด้วย
เคยแต่เลิกแ	1	0	0	0	ไม่มี	เห็นด้วย	เห็นด้วย
เคยแต่เลิกแ	2	2	0	2	ไม่มี	เห็นด้วย	เห็นด้วย
เคยแต่เลิกแ	1	0	0	0	ไม่มี	เห็นด้วย	เห็นด้วย
เคยแต่เลิกแ	1	0	0	0	ไม่มี	เห็นด้วย	เห็นด้วย
ไม่เคย	2	1	0	1	ไม่มี	เห็นด้วย	เห็นด้วย
ไม่เคย	4	2	1	2	ไม่มี	ไม่เห็นด้วย	ไม่เห็นด้วย
เคยแต่เลิกแ	1	0	0	0	มี ได้แก่.... อายุเยอะ	ไม่เห็นด้วย	เห็นด้วย
ไม่เคย	1	0	0	0	ไม่มี	ไม่เห็นด้วย	เห็นด้วย
เคยแต่เลิกแ	3	2	0	2	ไม่มี	เห็นด้วย	เห็นด้วยอย่
ไม่เคย	3	0	2	1	มี ได้แก่.... ครรภ์เป็นพิ	เห็นด้วย	เห็นด้วยอย่
เคยแต่เลิกแ	1	0	0	0	ไม่มี	ไม่เห็นด้วย	เห็นด้วย
เคยแต่เลิกแ	1	0	0	0	ไม่มี	ไม่เห็นด้วย	เห็นด้วย
เคยแต่เลิกแ	1	0	0	0	ไม่มี	เห็นด้วย	เห็นด้วย
เคยแต่เลิกแ	2	1	0	2	ไม่มี	ไม่เห็นด้วย	ไม่เห็นด้วย
ไม่เคย	1	0	0	0	ไม่มี	เห็นด้วย	ไม่เห็นด้วย
เคยแต่เลิกแ	2	1	0	1	ไม่มี	ไม่เห็นด้วย	ไม่เห็นด้วย
ไม่เคย	3	1	0	0	ไม่มี	เห็นด้วย	เห็นด้วย
เคยแต่เลิกแ	1	0	0	0	ไม่มี	เห็นด้วยอย่	เห็นด้วยอย่
ไม่เคย	1	0	0	0	ไม่มี	เห็นด้วย	เห็นด้วยอย่
เคยแต่เลิกแ	1	0	0	0	ไม่มี	เห็นด้วย	เห็นด้วย
เคยแต่เลิกแ	1	0	0	0	ไม่มี	ไม่เห็นด้วย	เห็นด้วย
ไม่เคย	2	1	0	1	ไม่มี	ไม่เห็นด้วย	ไม่เห็นด้วย
เคยแต่เลิกแ	1	0	0	0	ไม่มี	เห็นด้วย	เห็นด้วย
ไม่เคย	3	2	0	2	ไม่มี	ไม่เห็นด้วย	เห็นด้วย
เคยแต่เลิกแ	4	3	0	3	ไม่มี	ไม่เห็นด้วย	ไม่เห็นด้วย
เคยแต่เลิกแ	4	3	0	3	ไม่มี	ไม่เห็นด้วย	ไม่เห็นด้วย
เคยแต่เลิกแ	1	0	0	0	ไม่มี	เห็นด้วย	เห็นด้วย
เคยแต่เลิกแ	1	0	0	0	ไม่มี	ไม่เห็นด้วย	ไม่เห็นด้วย
เคยแต่เลิกแ	1	0	0	0	ไม่มี	ไม่เห็นด้วย	เห็นด้วย
เคยแต่เลิกแ	2	1	0	1	ไม่มี	เห็นด้วย	เห็นด้วย
เคยแต่เลิกแ	1	0	0	1	ไม่มี	เห็นด้วย	เห็นด้วย
เคยแต่เลิกแ	2	2	0	2	ไม่มี	เห็นด้วย	เห็นด้วย
เคยแต่เลิกแ	1	0	0	0	ไม่มี	เห็นด้วย	เห็นด้วย

เคยแต่เลิกเ	1	0	0	0	ไม่มี	เห็นด้วย	เห็นด้วย
ไม่เคย	2	1	0	1	ไม่มี	เห็นด้วย	เห็นด้วย
ไม่เคย	4	2	1	3	ไม่มี	ไม่เห็นด้วย	ไม่เห็นด้วย
เคยแต่เลิกเ	1	0	0	0	มี ได้แก่.... อายุเยอะ	เห็นด้วย	เห็นด้วยอย่
เคยแต่เลิกเ	1	0	0	1	ไม่มี	ไม่เห็นด้วย	เห็นด้วย
ไม่เคย	1	0	0	0	ไม่มี	เห็นด้วย	เห็นด้วยอย่
เคยแต่เลิกเ	2	1	0	1	ไม่มี	เห็นด้วยอย่	เห็นด้วยอย่
ไม่เคย	1	0	0	0	ไม่มี	เห็นด้วย	เห็นด้วยอย่
ไม่เคย	3	2	0	2	ไม่มี	เห็นด้วย	เห็นด้วย
ไม่เคย	1	0	0	0	ไม่มี	เห็นด้วย	ไม่เห็นด้วย
เคยแต่เลิกเ	2	0	1	0	ไม่มี	ไม่เห็นด้วย	ไม่เห็นด้วย
เคยแต่เลิกเ	1	0	0	0	ไม่มี	เห็นด้วย	เห็นด้วย
เคยแต่เลิกเ	1	0	0	0	ไม่มี	เห็นด้วย	เห็นด้วย
ไม่เคย	1	0	0	0	ไม่มี	เห็นด้วย	เห็นด้วยอย่
เคยแต่เลิกเ	1	0	0	0	ไม่มี	เห็นด้วย	เห็นด้วย
เคยแต่เลิกเ	1	0	0	0	ไม่มี	ไม่เห็นด้วย	เห็นด้วย
ไม่เคย	2	1	0	1	ไม่มี	ไม่เห็นด้วย	ไม่เห็นด้วย
เคยแต่เลิกเ	1	0	0	0	ไม่มี	เห็นด้วย	เห็นด้วย
ไม่เคย	3	2	0	2	ไม่มี	ไม่เห็นด้วย	เห็นด้วย
ไม่เคย	4	2	4	2	ไม่มี	ไม่เห็นด้วย	ไม่เห็นด้วย
ไม่เคย	4	3	0	3	ไม่มี	ไม่เห็นด้วย	ไม่เห็นด้วย
เคยแต่เลิกเ	1	0	0	0	ไม่มี	เห็นด้วย	เห็นด้วย
เคยแต่เลิกเ	1	0	0	0	ไม่มี	ไม่เห็นด้วย	ไม่เห็นด้วย
เคยแต่เลิกเ	1	0	0	0	ไม่มี	ไม่เห็นด้วย	เห็นด้วย
ไม่เคย	2	1	0	1	ไม่มี	เห็นด้วย	เห็นด้วย
ไม่เคย	1	0	0	0	ไม่มี	เห็นด้วย	ไม่เห็นด้วย
ไม่เคย	1	0	0	0	ไม่มี	เห็นด้วย	เห็นด้วย
ไม่เคย	3	2	0	0	ไม่มี	เห็นด้วย	เห็นด้วย
ไม่เคย	2	1	0	1	ไม่มี	เห็นด้วย	เห็นด้วย
เคยแต่เลิกเ	3	2	0	2	ไม่มี	เห็นด้วย	เห็นด้วย
เคยแต่เลิกเ	2	1	0	1	ไม่มี	เห็นด้วยอย่	เห็นด้วยอย่
เคยแต่เลิกเ	1	0	0	0	ไม่มี	เห็นด้วยอย่	เห็นด้วยอย่
เคยแต่เลิกเ	1	0	0	0	ไม่มี	เห็นด้วย	เห็นด้วย
ไม่เคย	3	2	0	2	ไม่มี	เห็นด้วย	เห็นด้วย
เคยแต่เลิกเ	1	0	0	0	ไม่มี	เห็นด้วยอย่	เห็นด้วยอย่
เคยแต่เลิกเ	2	1	0	1	ไม่มี	เห็นด้วยอย่	เห็นด้วยอย่
ไม่เคย	1	0	0	0	ไม่มี	เห็นด้วยอย่	เห็นด้วยอย่
เคยแต่เลิกเ	1	0	0	0	ไม่มี	เห็นด้วย	เห็นด้วย
เคยแต่เลิกเ	2	1	0	1	ไม่มี	เห็นด้วย	เห็นด้วย
เคยแต่เลิกเ	1	0	0	0	ไม่มี	เห็นด้วย	เห็นด้วย
เคยแต่เลิกเ	1	0	0	0	ไม่มี	เห็นด้วย	เห็นด้วย
เคยแต่เลิกเ	1	0	0	0	ไม่มี	เห็นด้วยอย่	เห็นด้วยอย่
เคยแต่เลิกเ	2	1	0	1	ไม่มี	เห็นด้วยอย่	เห็นด้วยอย่
เคยแต่เลิกเ	2	1	0	1	มี ได้แก่.... อายุเยอะ	เห็นด้วย	เห็นด้วย
เคยแต่เลิกเ	1	0	0	0	ไม่มี	เห็นด้วย	เห็นด้วย
เคยแต่เลิกเ	2	1	0	1	ไม่มี	เห็นด้วยอย่	เห็นด้วยอย่
เคยแต่เลิกเ	1	0	0	0	ไม่มี	เห็นด้วยอย่	เห็นด้วยอย่
ไม่เคย	1	0	0	0	ไม่มี	เห็นด้วย	เห็นด้วย
ไม่เคย	4	3	0	3	ไม่มี	เห็นด้วย	เห็นด้วย
ไม่เคย	1	0	0	0	ไม่มี	เห็นด้วย	เห็นด้วย
ไม่เคย	2	1	0	1	ไม่มี	เห็นด้วย	เห็นด้วยอย่

ไม่เคย	1	0	0	0	ไม่มี	เห็นด้วย	เห็นด้วย
ไม่เคย	1	0	0	0	ไม่มี	เห็นด้วย	เห็นด้วย
เคยแต่เลิกเ	1	0	0	0	ไม่มี	เห็นด้วย	เห็นด้วยอย่
เคยแต่เลิกเ	2	1	0	1	ไม่มี	เห็นด้วย	เห็นด้วย
เคยแต่เลิกเ	1	0	0	0	ไม่มี	เห็นด้วย	เห็นด้วย
เคยแต่เลิกเ	3	2	0	2	ไม่มี	เห็นด้วย	เห็นด้วย
ไม่เคย	1	0	0	0	ไม่มี	เห็นด้วย	เห็นด้วย
ไม่เคย	2	1	0	2	ไม่มี	เห็นด้วย	ไม่เห็นด้วย
เคยแต่เลิกเ	2	1	0	1	ไม่มี	เห็นด้วยอย่	เห็นด้วยอย่
ไม่เคย	3	2	0	2	ไม่มี	เห็นด้วย	เห็นด้วย
ไม่เคย	2	1	0	2	ไม่มี	เห็นด้วย	ไม่เห็นด้วย
เคยแต่เลิกเ	2	1	0	1	ไม่มี	เห็นด้วย	เห็นด้วย
เคยแต่เลิกเ	1	0	0	0	ไม่มี	เห็นด้วยอย่	เห็นด้วยอย่
เคยแต่เลิกเ	1	0	0	0	ไม่มี	เห็นด้วยอย่	เห็นด้วย
ไม่เคย	2	1	0	1	ไม่มี	เห็นด้วย	เห็นด้วย
ไม่เคย	2	1	0	1	ไม่มี	ไม่เห็นด้วยเ	ไม่เห็นด้วยเ
เคยแต่เลิกเ	2	1	0	1	ไม่มี	เห็นด้วย	เห็นด้วย
เคยแต่เลิกเ	3	1	1	2	ไม่มี	เห็นด้วย	เห็นด้วยอย่
ไม่เคย	2	1	0	1	ไม่มี	เห็นด้วย	เห็นด้วย
เคยแต่เลิกเ	1	0	0	0	ไม่มี	เห็นด้วยอย่	เห็นด้วยอย่
เคยแต่เลิกเ	1	0	0	0	ไม่มี	เห็นด้วย	เห็นด้วย
เคยแต่เลิกเ	1	0	0	0	ไม่มี	เห็นด้วย	เห็นด้วย
เคยแต่เลิกเ	2	1	0	1	ไม่มี	เห็นด้วย	เห็นด้วย
ไม่เคย	2	1	0	1	ไม่มี	เห็นด้วย	เห็นด้วย
เคยแต่เลิกเ	3	2	0	2	ไม่มี	เห็นด้วย	เห็นด้วย
เคยแต่เลิกเ	3	2	0	2	ไม่มี	เห็นด้วย	เห็นด้วย
เคยแต่เลิกเ	1	0	0	0	ไม่มี	ไม่เห็นด้วย	ไม่เห็นด้วย
เคยแต่เลิกเ	1	0	0	0	ไม่มี	เห็นด้วย	เห็นด้วย
ไม่เคย	2	1	0	1	ไม่มี	เห็นด้วยอย่	เห็นด้วยอย่
ไม่เคย	2	1	0	1	ไม่มี	ไม่เห็นด้วย	ไม่เห็นด้วย
เคยแต่เลิกเ	1	0	0	0	ไม่มี	เห็นด้วย	เห็นด้วย
ไม่เคย	6	2	3	2	ไม่มี	ไม่เห็นด้วย	ไม่เห็นด้วย
เคยแต่เลิกเ	1	0	0	0	ไม่มี	ไม่เห็นด้วย	เห็นด้วย
เคยแต่เลิกเ	1	0	0	0	ไม่มี	เห็นด้วย	เห็นด้วย
เคยแต่เลิกเ	1	0	0	0	ไม่มี	เห็นด้วย	เห็นด้วยอย่
เคยแต่เลิกเ	2	1	0	1	ไม่มี	เห็นด้วย	เห็นด้วย
เคยแต่เลิกเ	2	1	0	1	ไม่มี	เห็นด้วย	เห็นด้วย
เคยแต่เลิกเ	1	0	0	0	ไม่มี	เห็นด้วย	เห็นด้วย
เคยแต่เลิกเ	1	0	0	0	ไม่มี	เห็นด้วย	เห็นด้วยอย่
ไม่เคย	4	3	0	3	ไม่มี	เห็นด้วย	เห็นด้วย
ไม่เคย	1	0	0	0	ไม่มี	เห็นด้วย	เห็นด้วย
ไม่เคย	2	1	0	1	ไม่มี	เห็นด้วย	เห็นด้วยอย่
ไม่เคย	1	0	0	0	ไม่มี	เห็นด้วย	เห็นด้วย
ไม่เคย	1	0	0	0	ไม่มี	เห็นด้วย	เห็นด้วย
เคยแต่เลิกเ	1	0	0	0	ไม่มี	เห็นด้วย	เห็นด้วยอย่
เคยแต่เลิกเ	2	1	0	1	ไม่มี	เห็นด้วย	เห็นด้วย
เคยแต่เลิกเ	1	0	0	0	ไม่มี	เห็นด้วย	เห็นด้วย
เคยแต่เลิกเ	1	0	0	0	ไม่มี	เห็นด้วย	เห็นด้วย
เคยแต่เลิกเ	1	0	0	0	ไม่มี	เห็นด้วย	เห็นด้วย

suscept3	suscept4	suscept5	suscept6	suscept7	suscept8	suscept9	suscept10	Sum_Susc
ไม่เห็นด้วย	เห็นด้วย	เห็นด้วย	เห็นด้วย	ไม่เห็นด้วย	เห็นด้วย	เห็นด้วยอย่	ไม่เห็นด้วย	30.00
ไม่เห็นด้วยอ	เห็นด้วย	เห็นด้วย	เห็นด้วย	ไม่เห็นด้วย	เห็นด้วย	ไม่เห็นด้วย	ไม่เห็นด้วย	32.00
ไม่เห็นด้วย	เห็นด้วย	เห็นด้วย	ไม่เห็นด้วย	ไม่เห็นด้วย	เห็นด้วย	ไม่เห็นด้วย	ไม่เห็นด้วย	38.00
ไม่เห็นด้วย	เห็นด้วย	เห็นด้วยอย่	ไม่เห็นด้วย	ไม่เห็นด้วยอ	เห็นด้วยอย่	ไม่เห็นด้วย	ไม่เห็นด้วย	20.00
เห็นด้วย	เห็นด้วย	เห็นด้วย	ไม่เห็นด้วย	ไม่เห็นด้วย	ไม่เห็นด้วย	ไม่เห็นด้วย	ไม่เห็นด้วย	30.00
เห็นด้วย	เห็นด้วย	เห็นด้วย	เห็นด้วย	เห็นด้วย	เห็นด้วย	เห็นด้วย	เห็นด้วย	32.00
เห็นด้วย	เห็นด้วย	เห็นด้วยอย่	ไม่เห็นด้วย	ไม่เห็นด้วย	ไม่เห็นด้วย	ไม่เห็นด้วย	เห็นด้วย	28.00
เห็นด้วยอย่	เห็นด้วยอย่	เห็นด้วยอย่	เห็นด้วยอย่	เห็นด้วยอย่	เห็นด้วยอย่	เห็นด้วยอย่	เห็นด้วยอย่	35.00
ไม่เห็นด้วย	เห็นด้วยอย่	เห็นด้วยอย่	เห็นด้วย	ไม่เห็นด้วย	เห็นด้วย	เห็นด้วย	ไม่เห็นด้วย	33.00
เห็นด้วยอย่	เห็นด้วยอย่	เห็นด้วยอย่	เห็นด้วย	เห็นด้วย	เห็นด้วยอย่	เห็นด้วยอย่	เห็นด้วย	30.00
ไม่เห็นด้วย	เห็นด้วย	เห็นด้วย	เห็นด้วย	เห็นด้วย	เห็นด้วย	เห็นด้วย	ไม่เห็นด้วย	34.00
เห็นด้วย	เห็นด้วย	เห็นด้วย	เห็นด้วย	ไม่เห็นด้วย	เห็นด้วย	เห็นด้วย	ไม่เห็นด้วย	30.00
เห็นด้วย	เห็นด้วย	เห็นด้วย	เห็นด้วย	เห็นด้วย	เห็นด้วย	เห็นด้วย	เห็นด้วย	30.00
ไม่เห็นด้วย	เห็นด้วย	เห็นด้วย	เห็นด้วย	เห็นด้วย	เห็นด้วย	เห็นด้วย	เห็นด้วย	34.00
เห็นด้วยอย่	เห็นด้วย	เห็นด้วย	เห็นด้วย	ไม่เห็นด้วย	เห็นด้วยอย่	เห็นด้วย	เห็นด้วย	35.00
เห็นด้วยอย่	เห็นด้วย	เห็นด้วยอย่	เห็นด้วยอย่	ไม่เห็นด้วย	เห็นด้วย	เห็นด้วย	เห็นด้วย	38.00
เห็นด้วย	เห็นด้วย	เห็นด้วย	เห็นด้วย	เห็นด้วย	เห็นด้วย	เห็นด้วย	เห็นด้วย	30.00
เห็นด้วย	เห็นด้วย	เห็นด้วยอย่	เห็นด้วยอย่	ไม่เห็นด้วย	เห็นด้วยอย่	เห็นด้วย	เห็นด้วย	29.00
เห็นด้วย	เห็นด้วย	เห็นด้วย	เห็นด้วยอย่	ไม่เห็นด้วย	ไม่เห็นด้วยอ	ไม่เห็นด้วยอ	เห็นด้วย	28.00
เห็นด้วย	เห็นด้วย	เห็นด้วย	เห็นด้วย	เห็นด้วย	เห็นด้วย	เห็นด้วย	เห็นด้วย	28.00
เห็นด้วย	เห็นด้วย	เห็นด้วย	ไม่เห็นด้วย	เห็นด้วยอย่	เห็นด้วยอย่	เห็นด้วยอย่	เห็นด้วยอย่	30.00
ไม่เห็นด้วย	ไม่เห็นด้วย	ไม่เห็นด้วย	ไม่เห็นด้วย	ไม่เห็นด้วย	เห็นด้วย	เห็นด้วย	ไม่เห็นด้วย	22.00
ไม่เห็นด้วยอ	เห็นด้วยอย่	เห็นด้วยอย่	เห็นด้วยอย่	เห็นด้วย	เห็นด้วยอย่	เห็นด้วย	เห็นด้วย	29.00
ไม่เห็นด้วย	เห็นด้วย	เห็นด้วย	ไม่เห็นด้วย	เห็นด้วย	เห็นด้วยอย่	เห็นด้วย	ไม่เห็นด้วย	29.00
เห็นด้วย	ไม่เห็นด้วย	เห็นด้วย	เห็นด้วย	เห็นด้วย	เห็นด้วย	เห็นด้วย	เห็นด้วย	30.00
เห็นด้วยอย่	เห็นด้วยอย่	เห็นด้วยอย่	เห็นด้วยอย่	เห็นด้วย	เห็นด้วยอย่	เห็นด้วย	เห็นด้วย	27.00
ไม่เห็นด้วยอ	เห็นด้วยอย่	เห็นด้วยอย่	เห็นด้วยอย่	ไม่เห็นด้วยอ	เห็นด้วยอย่	เห็นด้วยอย่	เห็นด้วย	38.00
ไม่เห็นด้วยอ	เห็นด้วย	เห็นด้วย	เห็นด้วย	ไม่เห็นด้วยอ	ไม่เห็นด้วยอ	ไม่เห็นด้วยอ	เห็นด้วย	29.00
ไม่เห็นด้วย	เห็นด้วย	เห็นด้วย	เห็นด้วย	ไม่เห็นด้วย	เห็นด้วย	เห็นด้วย	เห็นด้วย	30.00
ไม่เห็นด้วย	เห็นด้วย	เห็นด้วย	เห็นด้วย	ไม่เห็นด้วย	เห็นด้วย	เห็นด้วย	ไม่เห็นด้วย	30.00
เห็นด้วย	เห็นด้วย	เห็นด้วย	เห็นด้วย	ไม่เห็นด้วย	เห็นด้วย	เห็นด้วย	เห็นด้วย	29.00
เห็นด้วย	เห็นด้วย	เห็นด้วย	ไม่เห็นด้วย	ไม่เห็นด้วย	เห็นด้วย	เห็นด้วย	ไม่เห็นด้วย	28.00
เห็นด้วย	เห็นด้วย	เห็นด้วย	เห็นด้วย	เห็นด้วย	เห็นด้วย	เห็นด้วย	เห็นด้วย	36.00
เห็นด้วยอย่	เห็นด้วยอย่	เห็นด้วยอย่	เห็นด้วยอย่	เห็นด้วยอย่	เห็นด้วยอย่	เห็นด้วยอย่	เห็นด้วยอย่	36.00
เห็นด้วย	เห็นด้วย	เห็นด้วย	ไม่เห็นด้วย	ไม่เห็นด้วย	เห็นด้วย	เห็นด้วย	ไม่เห็นด้วย	32.00
เห็นด้วย	เห็นด้วย	เห็นด้วย	เห็นด้วย	ไม่เห็นด้วย	เห็นด้วยอย่	เห็นด้วย	ไม่เห็นด้วย	30.00
เห็นด้วย	เห็นด้วย	เห็นด้วยอย่	เห็นด้วย	ไม่เห็นด้วย	ไม่เห็นด้วยอ	ไม่เห็นด้วย	ไม่เห็นด้วยอ	30.00
เห็นด้วย	ไม่เห็นด้วย	เห็นด้วย	ไม่เห็นด้วย	ไม่เห็นด้วย	ไม่เห็นด้วย	ไม่เห็นด้วย	ไม่เห็นด้วย	28.00
ไม่เห็นด้วย	ไม่เห็นด้วย	เห็นด้วย	ไม่เห็นด้วย	ไม่เห็นด้วย	ไม่เห็นด้วย	ไม่เห็นด้วย	ไม่เห็นด้วย	25.00
เห็นด้วย	เห็นด้วย	เห็นด้วย	ไม่เห็นด้วย	ไม่เห็นด้วย	เห็นด้วย	เห็นด้วย	ไม่เห็นด้วย	33.00
เห็นด้วย	ไม่เห็นด้วย	ไม่เห็นด้วย	เห็นด้วย	ไม่เห็นด้วย	เห็นด้วย	ไม่เห็นด้วย	ไม่เห็นด้วย	36.00
เห็นด้วย	เห็นด้วย	เห็นด้วย	เห็นด้วย	เห็นด้วย	เห็นด้วย	ไม่เห็นด้วย	ไม่เห็นด้วย	23.00
เห็นด้วย	เห็นด้วย	ไม่เห็นด้วย	เห็นด้วย	เห็นด้วยอย่	เห็นด้วยอย่	เห็นด้วยอย่	เห็นด้วย	30.00
เห็นด้วยอย่	เห็นด้วยอย่	เห็นด้วยอย่	เห็นด้วยอย่	เห็นด้วยอย่	เห็นด้วยอย่	เห็นด้วยอย่	เห็นด้วยอย่	30.00
เห็นด้วย	เห็นด้วย	เห็นด้วย	เห็นด้วย	เห็นด้วย	เห็นด้วย	เห็นด้วย	เห็นด้วย	30.00
ไม่เห็นด้วย	ไม่เห็นด้วย	ไม่เห็นด้วย	ไม่เห็นด้วย	เห็นด้วย	เห็นด้วย	เห็นด้วย	เห็นด้วย	23.00
เห็นด้วยอย่	เห็นด้วยอย่	ไม่เห็นด้วย	เห็นด้วย	เห็นด้วย	เห็นด้วย	เห็นด้วย	เห็นด้วย	30.00
เห็นด้วย	เห็นด้วย	เห็นด้วย	เห็นด้วย	เห็นด้วย	เห็นด้วย	เห็นด้วย	เห็นด้วย	35.00
เห็นด้วย	เห็นด้วย	เห็นด้วย	เห็นด้วย	เห็นด้วย	เห็นด้วย	เห็นด้วย	เห็นด้วย	40.00
เห็นด้วย	เห็นด้วย	ไม่เห็นด้วย	ไม่เห็นด้วย	เห็นด้วย	เห็นด้วย	ไม่เห็นด้วย	ไม่เห็นด้วย	30.00

เห็นด้วย	เห็นด้วย	เห็นด้วย	เห็นด้วย	เห็นด้วย	เห็นด้วย	เห็นด้วย	เห็นด้วย	29.00
เห็นด้วยอย่	ไม่เห็นด้วย	เห็นด้วย	ไม่เห็นด้วย	ไม่เห็นด้วย	เห็นด้วย	เห็นด้วยอย่	เห็นด้วย	33.00
ไม่เห็นด้วย	เห็นด้วย	เห็นด้วย	ไม่เห็นด้วย	เห็นด้วย	ไม่เห็นด้วย	ไม่เห็นด้วย	เห็นด้วย	30.00
ไม่เห็นด้วย	เห็นด้วย	เห็นด้วย	เห็นด้วย	เห็นด้วย	เห็นด้วย	เห็นด้วยอย่	เห็นด้วย	26.00
เห็นด้วย	เห็นด้วยอย่	เห็นด้วยอย่	เห็นด้วย	เห็นด้วยอย่	เห็นด้วยอย่	เห็นด้วย	เห็นด้วยอย่	23.00
เห็นด้วย	เห็นด้วย	เห็นด้วย	เห็นด้วย	เห็นด้วยอย่	เห็นด้วย	เห็นด้วย	ไม่เห็นด้วย	36.00
เห็นด้วย	เห็นด้วย	เห็นด้วย	เห็นด้วย	เห็นด้วย	เห็นด้วย	เห็นด้วย	เห็นด้วย	22.00
เห็นด้วยอย่	เห็นด้วย	เห็นด้วย	เห็นด้วย	เห็นด้วย	เห็นด้วยอย่	เห็นด้วย	ไม่เห็นด้วย	30.00
เห็นด้วย	ไม่เห็นด้วย	ไม่เห็นด้วย	ไม่เห็นด้วย	เห็นด้วย	เห็นด้วย	เห็นด้วย	เห็นด้วย	33.00
เห็นด้วย	เห็นด้วย	เห็นด้วยอย่	เห็นด้วย	ไม่เห็นด้วยอ	เห็นด้วย	เห็นด้วย	ไม่เห็นด้วย	33.00
ไม่เห็นด้วย	ไม่เห็นด้วย	เห็นด้วย	เห็นด้วย	เห็นด้วย	เห็นด้วย	เห็นด้วย	เห็นด้วย	33.00
เห็นด้วยอย่	ไม่เห็นด้วย	ไม่เห็นด้วย	เห็นด้วย	ไม่เห็นด้วย	เห็นด้วยอย่	ไม่เห็นด้วย	ไม่เห็นด้วย	30.00
เห็นด้วย	ไม่เห็นด้วย	เห็นด้วย	เห็นด้วย	ไม่เห็นด้วยอ	เห็นด้วยอย่	เห็นด้วยอย่	เห็นด้วยอย่	25.00
ไม่เห็นด้วยอ	เห็นด้วย	เห็นด้วยอย่	เห็นด้วยอย่	ไม่เห็นด้วย	เห็นด้วยอย่	เห็นด้วยอย่	ไม่เห็นด้วย	30.00
เห็นด้วยอย่	เห็นด้วย	เห็นด้วย	เห็นด้วยอย่	เห็นด้วย	เห็นด้วยอย่	เห็นด้วยอย่	ไม่เห็นด้วย	26.00
เห็นด้วย	เห็นด้วยอย่	เห็นด้วยอย่	เห็นด้วยอย่	เห็นด้วยอย่	เห็นด้วย	เห็นด้วยอย่	เห็นด้วย	30.00
เห็นด้วย	เห็นด้วย	เห็นด้วย	เห็นด้วย	เห็นด้วย	เห็นด้วย	เห็นด้วย	เห็นด้วย	31.00
เห็นด้วย	เห็นด้วย	เห็นด้วย	เห็นด้วย	เห็นด้วย	เห็นด้วย	เห็นด้วย	เห็นด้วย	38.00
เห็นด้วย	เห็นด้วย	เห็นด้วย	เห็นด้วย	เห็นด้วย	เห็นด้วย	เห็นด้วย	เห็นด้วย	30.00
เห็นด้วยอย่	เห็นด้วย	เห็นด้วย	ไม่เห็นด้วย	ไม่เห็นด้วย	เห็นด้วย	เห็นด้วย	ไม่เห็นด้วย	30.00
เห็นด้วยอย่	เห็นด้วย	เห็นด้วย	ไม่เห็นด้วย	ไม่เห็นด้วย	เห็นด้วย	เห็นด้วย	ไม่เห็นด้วย	31.00
เห็นด้วยอย่	เห็นด้วย	เห็นด้วยอย่	เห็นด้วย	ไม่เห็นด้วย	เห็นด้วยอย่	เห็นด้วย	เห็นด้วย	29.00
เห็นด้วย	เห็นด้วย	เห็นด้วยอย่	เห็นด้วยอย่	ไม่เห็นด้วย	เห็นด้วยอย่	เห็นด้วยอย่	เห็นด้วยอย่	25.00
ไม่เห็นด้วย	เห็นด้วย	เห็นด้วย	เห็นด้วย	เห็นด้วย	เห็นด้วย	เห็นด้วย	ไม่เห็นด้วย	22.00
ไม่เห็นด้วย	เห็นด้วย	เห็นด้วย	เห็นด้วย	เห็นด้วย	ไม่เห็นด้วย	ไม่เห็นด้วย	เห็นด้วย	30.00
เห็นด้วย	เห็นด้วย	เห็นด้วย	เห็นด้วย	ไม่เห็นด้วย	เห็นด้วย	ไม่เห็นด้วย	ไม่เห็นด้วย	30.00
ไม่เห็นด้วย	เห็นด้วย	เห็นด้วย	เห็นด้วย	เห็นด้วย	เห็นด้วย	เห็นด้วย	เห็นด้วย	28.00
เห็นด้วย	เห็นด้วย	เห็นด้วย	เห็นด้วย	เห็นด้วย	เห็นด้วย	เห็นด้วย	เห็นด้วย	30.00
เห็นด้วยอย่	เห็นด้วยอย่	เห็นด้วยอย่	เห็นด้วยอย่	เห็นด้วยอย่	เห็นด้วยอย่	เห็นด้วยอย่	เห็นด้วยอย่	30.00
เห็นด้วย	เห็นด้วย	เห็นด้วย	เห็นด้วย	ไม่เห็นด้วย	เห็นด้วย	เห็นด้วย	เห็นด้วย	31.00
เห็นด้วย	เห็นด้วย	เห็นด้วย	เห็นด้วย	เห็นด้วย	เห็นด้วย	เห็นด้วย	เห็นด้วย	30.00
เห็นด้วย	เห็นด้วย	เห็นด้วย	เห็นด้วย	เห็นด้วย	เห็นด้วย	เห็นด้วย	เห็นด้วย	38.00
เห็นด้วย	เห็นด้วย	เห็นด้วย	เห็นด้วย	เห็นด้วย	เห็นด้วย	เห็นด้วย	เห็นด้วย	39.00
เห็นด้วย	เห็นด้วย	เห็นด้วย	เห็นด้วย	เห็นด้วย	เห็นด้วย	เห็นด้วย	เห็นด้วย	28.00
เห็นด้วย	เห็นด้วย	เห็นด้วย	เห็นด้วย	เห็นด้วย	เห็นด้วย	เห็นด้วย	เห็นด้วย	32.00
เห็นด้วย	เห็นด้วย	เห็นด้วยอย่	เห็นด้วย	ไม่เห็นด้วย	เห็นด้วย	เห็นด้วย	เห็นด้วย	33.00
ไม่เห็นด้วยอ	ไม่เห็นด้วย	เห็นด้วย	เห็นด้วย	เห็นด้วย	เห็นด้วย	เห็นด้วย	เห็นด้วย	36.00
ไม่เห็นด้วย	เห็นด้วย	ไม่เห็นด้วย	เห็นด้วย	เห็นด้วย	เห็นด้วย	เห็นด้วย	เห็นด้วย	35.00
เห็นด้วย	เห็นด้วย	เห็นด้วย	เห็นด้วย	เห็นด้วย	เห็นด้วย	เห็นด้วย	เห็นด้วย	28.00
เห็นด้วย	เห็นด้วยอย่	เห็นด้วย	เห็นด้วย	เห็นด้วย	เห็นด้วย	เห็นด้วย	เห็นด้วยอย่	33.00
เห็นด้วย	เห็นด้วย	เห็นด้วย	เห็นด้วย	เห็นด้วย	เห็นด้วย	เห็นด้วย	เห็นด้วย	37.00
เห็นด้วยอย่	เห็นด้วย	เห็นด้วย	เห็นด้วย	เห็นด้วย	เห็นด้วย	เห็นด้วย	เห็นด้วย	24.00
เห็นด้วย	เห็นด้วย	เห็นด้วย	เห็นด้วย	เห็นด้วย	เห็นด้วย	เห็นด้วย	เห็นด้วย	27.00
เห็นด้วย	เห็นด้วย	เห็นด้วย	เห็นด้วย	เห็นด้วย	เห็นด้วยอย่	เห็นด้วยอย่	เห็นด้วยอย่	26.00
เห็นด้วย	เห็นด้วย	เห็นด้วย	เห็นด้วย	เห็นด้วย	เห็นด้วย	เห็นด้วย	เห็นด้วย	30.00
เห็นด้วย	เห็นด้วย	ไม่เห็นด้วย	ไม่เห็นด้วย	ไม่เห็นด้วย	เห็นด้วย	เห็นด้วยอย่	เห็นด้วย	26.00
เห็นด้วย	เห็นด้วย	เห็นด้วย	เห็นด้วย	เห็นด้วย	เห็นด้วย	เห็นด้วย	เห็นด้วย	26.00
เห็นด้วย	เห็นด้วย	เห็นด้วย	เห็นด้วย	เห็นด้วย	เห็นด้วยอย่	เห็นด้วยอย่	เห็นด้วย	30.00
ไม่เห็นด้วย	เห็นด้วย	เห็นด้วย	เห็นด้วย	ไม่เห็นด้วย	เห็นด้วย	เห็นด้วย	เห็นด้วย	23.00
เห็นด้วย	เห็นด้วย	เห็นด้วย	เห็นด้วย	เห็นด้วย	เห็นด้วย	เห็นด้วย	เห็นด้วย	23.00
เห็นด้วยอย่	เห็นด้วย	เห็นด้วยอย่	เห็นด้วยอย่	เห็นด้วย	เห็นด้วยอย่	ไม่เห็นด้วย	ไม่เห็นด้วย	29.00

เห็นด้วย	เห็นด้วย	เห็นด้วย	เห็นด้วย	เห็นด้วย	เห็นด้วย	เห็นด้วย	เห็นด้วย	29.00
เห็นด้วย	เห็นด้วย	เห็นด้วย	เห็นด้วย	ไม่เห็นด้วย	เห็นด้วย	เห็นด้วย	ไม่เห็นด้วย	33.00
เห็นด้วย	เห็นด้วย	เห็นด้วยอย่	เห็นด้วยอย่	เห็นด้วย	เห็นด้วย	เห็นด้วยอย่	ไม่เห็นด้วย	30.00
เห็นด้วยอย่	เห็นด้วยอย่	เห็นด้วยอย่	เห็นด้วยอย่	ไม่เห็นด้วย	เห็นด้วยอย่	เห็นด้วยอย่	เห็นด้วยอย่	33.00
เห็นด้วย	เห็นด้วย	เห็นด้วย	เห็นด้วย	เห็นด้วย	เห็นด้วย	เห็นด้วย	เห็นด้วย	35.00
ไม่เห็นด้วย	เห็นด้วย	เห็นด้วยอย่	ไม่เห็นด้วย	ไม่เห็นด้วย	เห็นด้วยอย่	เห็นด้วยอย่	เห็นด้วย	31.00
ไม่เห็นด้วย	เห็นด้วย	ไม่เห็นด้วย	ไม่เห็นด้วย	ไม่เห็นด้วยอ	เห็นด้วย	ไม่เห็นด้วยอ	ไม่เห็นด้วย	35.00
เห็นด้วย	เห็นด้วย	เห็นด้วย	เห็นด้วย	เห็นด้วย	เห็นด้วย	เห็นด้วย	เห็นด้วย	33.00
เห็นด้วยอย่	เห็นด้วย	เห็นด้วยอย่	เห็นด้วยอย่	เห็นด้วยอย่	เห็นด้วยอย่	เห็นด้วยอย่	เห็นด้วย	30.00
เห็นด้วยอย่	เห็นด้วย	เห็นด้วย	ไม่เห็นด้วย	ไม่เห็นด้วย	เห็นด้วย	ไม่เห็นด้วย	เห็นด้วย	28.00
ไม่เห็นด้วย	เห็นด้วย	เห็นด้วย	เห็นด้วย	ไม่เห็นด้วย	เห็นด้วย	ไม่เห็นด้วย	ไม่เห็นด้วย	27.00
เห็นด้วยอย่	เห็นด้วย	เห็นด้วย	ไม่เห็นด้วย	ไม่เห็นด้วย	เห็นด้วย	ไม่เห็นด้วย	เห็นด้วย	22.00
เห็นด้วย	เห็นด้วย	เห็นด้วย	ไม่เห็นด้วย	ไม่เห็นด้วย	เห็นด้วย	เห็นด้วย	ไม่เห็นด้วย	31.00
เห็นด้วยอย่	เห็นด้วย	เห็นด้วยอย่	เห็นด้วย	ไม่เห็นด้วย	เห็นด้วย	เห็นด้วย	เห็นด้วย	35.00
เห็นด้วย	เห็นด้วยอย่	เห็นด้วยอย่	เห็นด้วยอย่	ไม่เห็นด้วย	ไม่เห็นด้วย	เห็นด้วย	ไม่เห็นด้วย	28.00
เห็นด้วย	เห็นด้วย	เห็นด้วย	เห็นด้วย	ไม่เห็นด้วย	เห็นด้วย	เห็นด้วย	ไม่เห็นด้วย	26.00
เห็นด้วย	เห็นด้วย	เห็นด้วย	เห็นด้วย	เห็นด้วย	เห็นด้วย	เห็นด้วย	เห็นด้วย	33.00
ไม่เห็นด้วย	เห็นด้วย	เห็นด้วย	เห็นด้วย	ไม่เห็นด้วย	เห็นด้วย	เห็นด้วย	ไม่เห็นด้วย	40.00
เห็นด้วย	เห็นด้วย	เห็นด้วย	เห็นด้วย	ไม่เห็นด้วย	เห็นด้วย	ไม่เห็นด้วย	ไม่เห็นด้วย	30.00
ไม่เห็นด้วยอ	เห็นด้วย	เห็นด้วย	เห็นด้วย	ไม่เห็นด้วย	เห็นด้วย	เห็นด้วยอย่	เห็นด้วยอย่	21.00
ไม่เห็นด้วยอ	เห็นด้วย	เห็นด้วย	เห็นด้วย	เห็นด้วย	เห็นด้วย	เห็นด้วยอย่	เห็นด้วย	27.00
เห็นด้วย	เห็นด้วย	เห็นด้วย	เห็นด้วย	เห็นด้วย	เห็นด้วย	เห็นด้วย	เห็นด้วย	35.00
เห็นด้วย	เห็นด้วย	เห็นด้วย	เห็นด้วย	เห็นด้วย	เห็นด้วย	เห็นด้วย	เห็นด้วย	30.00
ไม่เห็นด้วย	เห็นด้วย	ไม่เห็นด้วย	ไม่เห็นด้วย	ไม่เห็นด้วย	เห็นด้วย	เห็นด้วย	ไม่เห็นด้วย	40.00
ไม่เห็นด้วย	เห็นด้วย	เห็นด้วย	ไม่เห็นด้วย	เห็นด้วย	เห็นด้วย	ไม่เห็นด้วย	เห็นด้วย	36.00
ไม่เห็นด้วย	ไม่เห็นด้วย	เห็นด้วยอย่	เห็นด้วย	ไม่เห็นด้วย	ไม่เห็นด้วย	เห็นด้วย	ไม่เห็นด้วย	20.00
ไม่เห็นด้วย	เห็นด้วย	เห็นด้วย	ไม่เห็นด้วย	ไม่เห็นด้วย	เห็นด้วย	เห็นด้วย	ไม่เห็นด้วย	30.00
เห็นด้วย	เห็นด้วย	เห็นด้วย	เห็นด้วย	เห็นด้วย	เห็นด้วยอย่	เห็นด้วยอย่	เห็นด้วย	30.00
เห็นด้วย	เห็นด้วย	ไม่เห็นด้วย	ไม่เห็นด้วย	ไม่เห็นด้วย	ไม่เห็นด้วย	ไม่เห็นด้วย	ไม่เห็นด้วย	22.00
เห็นด้วยอย่	เห็นด้วยอย่	เห็นด้วยอย่	เห็นด้วยอย่	เห็นด้วย	เห็นด้วย	เห็นด้วย	เห็นด้วย	39.00
เห็นด้วย	ไม่เห็นด้วย	เห็นด้วย	ไม่เห็นด้วย	ไม่เห็นด้วย	เห็นด้วย	เห็นด้วย	เห็นด้วย	38.00
เห็นด้วย	เห็นด้วย	เห็นด้วย	เห็นด้วย	เห็นด้วย	เห็นด้วย	เห็นด้วย	เห็นด้วย	32.00
เห็นด้วยอย่	เห็นด้วยอย่	เห็นด้วยอย่	เห็นด้วยอย่	เห็นด้วยอย่	เห็นด้วยอย่	เห็นด้วยอย่	เห็นด้วยอย่	27.00
เห็นด้วย	เห็นด้วย	เห็นด้วย	เห็นด้วย	ไม่เห็นด้วย	เห็นด้วย	เห็นด้วย	ไม่เห็นด้วย	30.00
ไม่เห็นด้วยอ	ไม่เห็นด้วย	ไม่เห็นด้วย	เห็นด้วย	เห็นด้วย	เห็นด้วย	ไม่เห็นด้วย	เห็นด้วย	35.00
เห็นด้วย	เห็นด้วย	เห็นด้วยอย่	เห็นด้วย	ไม่เห็นด้วย	เห็นด้วยอย่	เห็นด้วย	ไม่เห็นด้วย	26.00
เห็นด้วยอย่	เห็นด้วย	เห็นด้วย	เห็นด้วย	เห็นด้วย	เห็นด้วยอย่	เห็นด้วย	เห็นด้วยอย่	29.00
เห็นด้วยอย่	เห็นด้วย	เห็นด้วย	ไม่เห็นด้วย	ไม่เห็นด้วยอ	เห็นด้วย	เห็นด้วย	ไม่เห็นด้วย	31.00
เห็นด้วย	เห็นด้วย	เห็นด้วย	เห็นด้วย	เห็นด้วย	เห็นด้วย	เห็นด้วย	เห็นด้วย	23.00
ไม่เห็นด้วยอ	ไม่เห็นด้วย	เห็นด้วย	เห็นด้วย	ไม่เห็นด้วย	ไม่เห็นด้วย	ไม่เห็นด้วย	เห็นด้วย	33.00
ไม่เห็นด้วย	ไม่เห็นด้วย	เห็นด้วย	เห็นด้วย	ไม่เห็นด้วย	เห็นด้วย	ไม่เห็นด้วย	ไม่เห็นด้วย	30.00
เห็นด้วย	ไม่เห็นด้วย	เห็นด้วย	เห็นด้วย	เห็นด้วย	เห็นด้วย	เห็นด้วย	เห็นด้วย	36.00
เห็นด้วย	ไม่เห็นด้วย	เห็นด้วย	เห็นด้วย	ไม่เห็นด้วย	เห็นด้วย	เห็นด้วย	ไม่เห็นด้วย	22.00
เห็นด้วยอย่	เห็นด้วย	เห็นด้วยอย่	เห็นด้วย	เห็นด้วย	เห็นด้วยอย่	เห็นด้วย	เห็นด้วย	35.00
เห็นด้วย	เห็นด้วย	เห็นด้วย	เห็นด้วย	เห็นด้วย	เห็นด้วย	เห็นด้วย	เห็นด้วย	25.00
ไม่เห็นด้วย	เห็นด้วย	เห็นด้วย	ไม่เห็นด้วย	ไม่เห็นด้วย	ไม่เห็นด้วย	ไม่เห็นด้วย	ไม่เห็นด้วย	33.00
เห็นด้วย	เห็นด้วย	เห็นด้วย	เห็นด้วย	เห็นด้วย	เห็นด้วย	เห็นด้วย	เห็นด้วย	29.00
ไม่เห็นด้วย	ไม่เห็นด้วย	เห็นด้วย	เห็นด้วย	ไม่เห็นด้วย	เห็นด้วย	เห็นด้วย	เห็นด้วย	29.00
ไม่เห็นด้วย	เห็นด้วย	เห็นด้วย	เห็นด้วย	ไม่เห็นด้วย	เห็นด้วย	เห็นด้วย	เห็นด้วย	20.00
เห็นด้วย	เห็นด้วย	เห็นด้วย	ไม่เห็นด้วย	เห็นด้วย	เห็นด้วย	เห็นด้วย	เห็นด้วย	26.00
เห็นด้วย	เห็นด้วย	เห็นด้วย	เห็นด้วย	เห็นด้วย	เห็นด้วย	เห็นด้วย	เห็นด้วย	30.00

ไม่เห็นด้วยอ	ไม่เห็นด้วย	เห็นด้วย	เห็นด้วย	เห็นด้วย	ไม่เห็นด้วย	ไม่เห็นด้วยอ	ไม่เห็นด้วย	24.00
เห็นด้วย	เห็นด้วย	เห็นด้วย	เห็นด้วย	เห็นด้วย	เห็นด้วย	เห็นด้วย	เห็นด้วย	30.00
ไม่เห็นด้วย	เห็นด้วย	เห็นด้วย	เห็นด้วย	ไม่เห็นด้วย	เห็นด้วย	เห็นด้วย	เห็นด้วย	30.00
ไม่เห็นด้วย	เห็นด้วย	เห็นด้วย	เห็นด้วย	เห็นด้วย	เห็นด้วย	เห็นด้วย	เห็นด้วย	24.00
ไม่เห็นด้วย	เห็นด้วย	เห็นด้วย	เห็นด้วย	เห็นด้วย	เห็นด้วย	เห็นด้วย	เห็นด้วย	24.00
เห็นด้วย	เห็นด้วย	เห็นด้วย	ไม่เห็นด้วย	ไม่เห็นด้วย	เห็นด้วย	เห็นด้วย	เห็นด้วย	30.00
เห็นด้วยอย่	เห็นด้วยอย่	เห็นด้วยอย่	ไม่เห็นด้วย	เห็นด้วยอย่	เห็นด้วยอย่	ไม่เห็นด้วย	เห็นด้วยอย่	22.00
เห็นด้วย	เห็นด้วย	เห็นด้วย	เห็นด้วย	เห็นด้วย	เห็นด้วย	เห็นด้วย	เห็นด้วย	28.00
เห็นด้วย	เห็นด้วยอย่	เห็นด้วย	เห็นด้วย	ไม่เห็นด้วย	เห็นด้วย	เห็นด้วย	เห็นด้วย	25.00
เห็นด้วยอย่	เห็นด้วย	เห็นด้วย	เห็นด้วย	เห็นด้วย	เห็นด้วย	เห็นด้วย	เห็นด้วย	31.00
เห็นด้วย	เห็นด้วย	เห็นด้วยอย่	เห็นด้วยอย่	ไม่เห็นด้วย	เห็นด้วย	เห็นด้วย	เห็นด้วย	30.00
ไม่เห็นด้วยอ	เห็นด้วย	เห็นด้วย	เห็นด้วย	ไม่เห็นด้วย	เห็นด้วย	ไม่เห็นด้วย	ไม่เห็นด้วยอ	33.00
เห็นด้วย	เห็นด้วย	เห็นด้วย	เห็นด้วย	เห็นด้วย	เห็นด้วย	เห็นด้วย	เห็นด้วย	30.00
ไม่เห็นด้วยอ	ไม่เห็นด้วย	เห็นด้วย	เห็นด้วย	เห็นด้วย	เห็นด้วย	เห็นด้วย	เห็นด้วย	29.00
ไม่เห็นด้วย	ไม่เห็นด้วย	เห็นด้วยอย่	ไม่เห็นด้วย	ไม่เห็นด้วย	เห็นด้วยอย่	เห็นด้วย	ไม่เห็นด้วย	35.00
เห็นด้วยอย่	เห็นด้วย	เห็นด้วย	เห็นด้วย	ไม่เห็นด้วยอ	ไม่เห็นด้วย	เห็นด้วย	เห็นด้วย	26.00
ไม่เห็นด้วย	ไม่เห็นด้วย	เห็นด้วย	เห็นด้วย	ไม่เห็นด้วย	เห็นด้วย	เห็นด้วย	ไม่เห็นด้วย	27.00
เห็นด้วยอย่	เห็นด้วย	เห็นด้วย	เห็นด้วย	เห็นด้วย	เห็นด้วย	เห็นด้วย	เห็นด้วย	34.00
เห็นด้วยอย่	เห็นด้วย	เห็นด้วย	เห็นด้วย	เห็นด้วย	เห็นด้วยอย่	เห็นด้วยอย่	เห็นด้วย	28.00
เห็นด้วย	เห็นด้วย	เห็นด้วยอย่	เห็นด้วย	เห็นด้วย	เห็นด้วย	ไม่เห็นด้วย	ไม่เห็นด้วย	25.00
เห็นด้วย	เห็นด้วย	เห็นด้วย	เห็นด้วย	ไม่เห็นด้วย	เห็นด้วยอย่	เห็นด้วยอย่	ไม่เห็นด้วย	30.00
เห็นด้วย	เห็นด้วย	เห็นด้วยอย่	เห็นด้วย	ไม่เห็นด้วย	เห็นด้วย	เห็นด้วย	ไม่เห็นด้วย	31.00
ไม่เห็นด้วย	เห็นด้วย	เห็นด้วย	เห็นด้วย	ไม่เห็นด้วย	เห็นด้วย	เห็นด้วย	เห็นด้วย	32.00
เห็นด้วยอย่	เห็นด้วย	เห็นด้วยอย่	เห็นด้วย	ไม่เห็นด้วย	เห็นด้วย	เห็นด้วยอย่	เห็นด้วย	26.00
เห็นด้วย	เห็นด้วย	เห็นด้วย	เห็นด้วย	เห็นด้วย	เห็นด้วย	เห็นด้วย	เห็นด้วย	34.00
เห็นด้วย	ไม่เห็นด้วย	เห็นด้วย	ไม่เห็นด้วย	ไม่เห็นด้วย	เห็นด้วย	เห็นด้วยอย่	เห็นด้วย	40.00
เห็นด้วยอย่	เห็นด้วย	ไม่เห็นด้วย	เห็นด้วย	เห็นด้วย	เห็นด้วยอย่	เห็นด้วยอย่	เห็นด้วย	27.00
เห็นด้วย	เห็นด้วย	เห็นด้วย	เห็นด้วย	เห็นด้วย	เห็นด้วย	เห็นด้วย	เห็นด้วย	31.00
ไม่เห็นด้วย	เห็นด้วย	เห็นด้วย	เห็นด้วย	เห็นด้วย	เห็นด้วย	ไม่เห็นด้วย	เห็นด้วย	30.00
เห็นด้วยอย่	เห็นด้วย	เห็นด้วยอย่	เห็นด้วย	ไม่เห็นด้วย	เห็นด้วย	เห็นด้วย	ไม่เห็นด้วย	26.00
เห็นด้วย	เห็นด้วย	เห็นด้วย	ไม่เห็นด้วย	ไม่เห็นด้วย	ไม่เห็นด้วย	เห็นด้วย	ไม่เห็นด้วย	30.00
เห็นด้วยอย่	เห็นด้วย	เห็นด้วยอย่	เห็นด้วยอย่	เห็นด้วย	เห็นด้วย	เห็นด้วย	เห็นด้วย	30.00
เห็นด้วยอย่	เห็นด้วยอย่	เห็นด้วยอย่	เห็นด้วย	เห็นด้วย	เห็นด้วย	เห็นด้วย	เห็นด้วย	27.00
ไม่เห็นด้วย	เห็นด้วย	เห็นด้วย	เห็นด้วย	เห็นด้วย	เห็นด้วย	เห็นด้วย	เห็นด้วย	39.00
เห็นด้วยอย่	เห็นด้วย	เห็นด้วย	เห็นด้วย	ไม่เห็นด้วย	เห็นด้วยอย่	ไม่เห็นด้วย	ไม่เห็นด้วย	34.00
เห็นด้วย	เห็นด้วย	เห็นด้วย	เห็นด้วย	เห็นด้วย	เห็นด้วย	เห็นด้วย	เห็นด้วย	30.00
เห็นด้วย	เห็นด้วย	เห็นด้วย	เห็นด้วย	เห็นด้วย	เห็นด้วย	เห็นด้วย	เห็นด้วย	22.00
เห็นด้วยอย่	เห็นด้วยอย่	เห็นด้วยอย่	เห็นด้วยอย่	เห็นด้วย	เห็นด้วยอย่	เห็นด้วยอย่	เห็นด้วยอย่	29.00
เห็นด้วย	เห็นด้วย	เห็นด้วยอย่	เห็นด้วย	ไม่เห็นด้วย	เห็นด้วย	เห็นด้วยอย่	เห็นด้วย	29.00
เห็นด้วยอย่	เห็นด้วยอย่	เห็นด้วยอย่	เห็นด้วยอย่	เห็นด้วย	เห็นด้วย	เห็นด้วยอย่	เห็นด้วยอย่	28.00
เห็นด้วย	เห็นด้วย	เห็นด้วย	เห็นด้วย	เห็นด้วย	เห็นด้วย	เห็นด้วย	เห็นด้วย	31.00
เห็นด้วยอย่	เห็นด้วยอย่	เห็นด้วยอย่	เห็นด้วยอย่	เห็นด้วย	เห็นด้วยอย่	ไม่เห็นด้วย	ไม่เห็นด้วย	31.00
เห็นด้วยอย่	เห็นด้วยอย่	เห็นด้วยอย่	เห็นด้วยอย่	เห็นด้วยอย่	เห็นด้วย	เห็นด้วยอย่	เห็นด้วยอย่	30.00
เห็นด้วย	เห็นด้วย	เห็นด้วย	เห็นด้วย	เห็นด้วย	เห็นด้วย	เห็นด้วย	เห็นด้วย	23.00
ไม่เห็นด้วยอ	เห็นด้วย	เห็นด้วย	เห็นด้วย	ไม่เห็นด้วยอ	เห็นด้วย	เห็นด้วย	ไม่เห็นด้วยอ	30.00
เห็นด้วย	เห็นด้วย	เห็นด้วย	เห็นด้วย	ไม่เห็นด้วย	เห็นด้วย	เห็นด้วย	เห็นด้วย	27.00
เห็นด้วยอย่	เห็นด้วยอย่	เห็นด้วยอย่	เห็นด้วยอย่	ไม่เห็นด้วย	เห็นด้วย	เห็นด้วย	ไม่เห็นด้วย	26.00
เห็นด้วยอย่	เห็นด้วยอย่	เห็นด้วยอย่	เห็นด้วย	เห็นด้วย	ไม่เห็นด้วย	เห็นด้วย	ไม่เห็นด้วย	23.00
เห็นด้วยอย่	เห็นด้วยอย่	เห็นด้วยอย่	เห็นด้วยอย่	เห็นด้วย	เห็นด้วย	เห็นด้วย	เห็นด้วย	31.00
เห็นด้วย	เห็นด้วย	เห็นด้วย	เห็นด้วย	เห็นด้วย	เห็นด้วย	เห็นด้วย	เห็นด้วย	23.00
เห็นด้วย	เห็นด้วย	เห็นด้วย	เห็นด้วย	เห็นด้วย	เห็นด้วย	เห็นด้วย	เห็นด้วย	26.00

เห็นด้วยอย่	เห็นด้วย	เห็นด้วย	เห็นด้วย	เห็นด้วย	เห็นด้วย	เห็นด้วย	เห็นด้วย	35.00
ไม่เห็นด้วย	เห็นด้วย	เห็นด้วย	เห็นด้วย	เห็นด้วย	เห็นด้วย	เห็นด้วย	เห็นด้วย	28.00
เห็นด้วยอย่	เห็นด้วย	เห็นด้วยอย่	เห็นด้วยอย่	เห็นด้วย	เห็นด้วยอย่	เห็นด้วยอย่	เห็นด้วย	18.00
เห็นด้วย	เห็นด้วย	เห็นด้วย	เห็นด้วย	เห็นด้วย	เห็นด้วย	เห็นด้วย	เห็นด้วย	25.00
เห็นด้วยอย่	เห็นด้วยอย่	เห็นด้วยอย่	เห็นด้วยอย่	ไม่เห็นด้วย	เห็นด้วย	เห็นด้วย	เห็นด้วย	33.00
เห็นด้วยอย่	เห็นด้วยอย่	เห็นด้วยอย่	เห็นด้วยอย่	เห็นด้วยอย่	เห็นด้วยอย่	เห็นด้วย	เห็นด้วย	27.00
เห็นด้วย	เห็นด้วย	เห็นด้วย	เห็นด้วย	เห็นด้วย	เห็นด้วย	เห็นด้วย	เห็นด้วย	30.00
เห็นด้วย	เห็นด้วย	เห็นด้วย	เห็นด้วย	ไม่เห็นด้วย	เห็นด้วย	เห็นด้วย	เห็นด้วย	33.00
เห็นด้วย	เห็นด้วย	เห็นด้วย	เห็นด้วย	เห็นด้วย	เห็นด้วย	เห็นด้วย	เห็นด้วย	40.00
เห็นด้วยอย่	เห็นด้วยอย่	เห็นด้วยอย่	เห็นด้วยอย่	เห็นด้วยอย่	เห็นด้วยอย่	เห็นด้วยอย่	เห็นด้วยอย่	35.00
เห็นด้วย	เห็นด้วย	เห็นด้วย	เห็นด้วย	เห็นด้วย	เห็นด้วย	เห็นด้วย	เห็นด้วย	28.00
เห็นด้วยอย่	เห็นด้วย	เห็นด้วยอย่	เห็นด้วย	เห็นด้วยอย่	เห็นด้วย	เห็นด้วยอย่	เห็นด้วยอย่	32.00
เห็นด้วยอย่	เห็นด้วย	เห็นด้วย	เห็นด้วย	เห็นด้วย	เห็นด้วย	เห็นด้วย	เห็นด้วย	35.00
เห็นด้วยอย่	เห็นด้วย	เห็นด้วย	เห็นด้วย	เห็นด้วย	เห็นด้วย	เห็นด้วยอย่	เห็นด้วย	30.00
เห็นด้วย	ไม่เห็นด้วย	เห็นด้วย	เห็นด้วย	เห็นด้วย	ไม่เห็นด้วย	เห็นด้วย	เห็นด้วย	29.00
เห็นด้วย	ไม่เห็นด้วย	เห็นด้วย	เห็นด้วย	เห็นด้วย	เห็นด้วย	ไม่เห็นด้วย	เห็นด้วย	31.00
เห็นด้วยอย่	เห็นด้วย	เห็นด้วย	เห็นด้วย	เห็นด้วย	เห็นด้วย	เห็นด้วยอย่	เห็นด้วยอย่	25.00
เห็นด้วย	เห็นด้วย	เห็นด้วย	เห็นด้วย	เห็นด้วย	เห็นด้วยอย่	เห็นด้วย	ไม่เห็นด้วย	26.00
เห็นด้วย	เห็นด้วย	เห็นด้วยอย่	เห็นด้วย	เห็นด้วย	เห็นด้วยอย่	เห็นด้วย	เห็นด้วยอย่	36.00
เห็นด้วย	ไม่เห็นด้วย	เห็นด้วย	ไม่เห็นด้วย	ไม่เห็นด้วย	เห็นด้วย	เห็นด้วย	ไม่เห็นด้วย	29.00
เห็นด้วย	เห็นด้วย	เห็นด้วย	เห็นด้วย	เห็นด้วย	เห็นด้วย	เห็นด้วย	เห็นด้วยอย่	30.00
เห็นด้วยอย่	ไม่เห็นด้วย	เห็นด้วยอย่	เห็นด้วย	ไม่เห็นด้วย	ไม่เห็นด้วย	ไม่เห็นด้วย	ไม่เห็นด้วย	20.00
ไม่เห็นด้วย	ไม่เห็นด้วย	เห็นด้วย	เห็นด้วย	ไม่เห็นด้วย	ไม่เห็นด้วย	เห็นด้วย	ไม่เห็นด้วย	30.00
เห็นด้วย	เห็นด้วย	เห็นด้วย	เห็นด้วย	เห็นด้วย	เห็นด้วย	เห็นด้วย	เห็นด้วย	32.00
เห็นด้วย	เห็นด้วย	เห็นด้วย	เห็นด้วย	ไม่เห็นด้วย	เห็นด้วย	ไม่เห็นด้วย	เห็นด้วย	26.00
เห็นด้วย	เห็นด้วย	เห็นด้วย	ไม่เห็นด้วย	เห็นด้วย	เห็นด้วย	เห็นด้วย	เห็นด้วย	28.00
เห็นด้วยอย่	เห็นด้วยอย่	เห็นด้วย	เห็นด้วย	เห็นด้วย	เห็นด้วย	เห็นด้วย	เห็นด้วย	29.00
เห็นด้วย	เห็นด้วย	ไม่เห็นด้วย	ไม่เห็นด้วย	ไม่เห็นด้วย	เห็นด้วย	เห็นด้วย	เห็นด้วย	40.00
เห็นด้วย	เห็นด้วย	เห็นด้วยอย่	เห็นด้วย	เห็นด้วยอย่	เห็นด้วยอย่	เห็นด้วยอย่	เห็นด้วย	27.00
เห็นด้วยอย่	เห็นด้วย	เห็นด้วยอย่	เห็นด้วย	เห็นด้วยอย่	ไม่เห็นด้วย	เห็นด้วย	ไม่เห็นด้วย	32.00
เห็นด้วย	เห็นด้วยอย่	ไม่เห็นด้วย	เห็นด้วย	เห็นด้วย	เห็นด้วย	เห็นด้วย	ไม่เห็นด้วย	27.00
เห็นด้วยอย่	เห็นด้วยอย่	เห็นด้วยอย่	เห็นด้วยอย่	เห็นด้วย	เห็นด้วยอย่	เห็นด้วย	ไม่เห็นด้วย	23.00
ไม่เห็นด้วย	เห็นด้วย	เห็นด้วย	เห็นด้วย	ไม่เห็นด้วย	ไม่เห็นด้วย	ไม่เห็นด้วย	ไม่เห็นด้วย	25.00
ไม่เห็นด้วย	ไม่เห็นด้วย	เห็นด้วย	เห็นด้วย	ไม่เห็นด้วย	เห็นด้วย	เห็นด้วย	เห็นด้วย	29.00
เห็นด้วย	เห็นด้วย	เห็นด้วยอย่	เห็นด้วยอย่	เห็นด้วย	เห็นด้วยอย่	เห็นด้วย	เห็นด้วยอย่	30.00
เห็นด้วยอย่	เห็นด้วยอย่	เห็นด้วยอย่	เห็นด้วย	ไม่เห็นด้วย	ไม่เห็นด้วย	ไม่เห็นด้วย	ไม่เห็นด้วย	30.00
เห็นด้วย	เห็นด้วย	ไม่เห็นด้วย	ไม่เห็นด้วย	ไม่เห็นด้วย	เห็นด้วย	เห็นด้วยอย่	ไม่เห็นด้วย	30.00
เห็นด้วยอย่	ไม่เห็นด้วย	เห็นด้วยอย่	เห็นด้วย	ไม่เห็นด้วย	เห็นด้วย	ไม่เห็นด้วย	ไม่เห็นด้วย	29.00
เห็นด้วย	เห็นด้วย	เห็นด้วยอย่	เห็นด้วยอย่	เห็นด้วยอย่	เห็นด้วย	เห็นด้วยอย่	เห็นด้วยอย่	23.00
เห็นด้วยอย่	เห็นด้วยอย่	เห็นด้วย	เห็นด้วยอย่	เห็นด้วย	เห็นด้วยอย่	เห็นด้วยอย่	เห็นด้วย	31.00
เห็นด้วยอย่	เห็นด้วยอย่	เห็นด้วย	เห็นด้วยอย่	ไม่เห็นด้วย	เห็นด้วย	เห็นด้วยอย่	เห็นด้วยอย่	34.00
ไม่เห็นด้วย	เห็นด้วย	เห็นด้วยอย่	เห็นด้วย	เห็นด้วยอย่	เห็นด้วย	เห็นด้วย	ไม่เห็นด้วย	32.00
เห็นด้วย	ไม่เห็นด้วย	เห็นด้วยอย่	ไม่เห็นด้วย	ไม่เห็นด้วย	เห็นด้วย	เห็นด้วย	ไม่เห็นด้วย	30.00
เห็นด้วย	ไม่เห็นด้วย	เห็นด้วย	ไม่เห็นด้วย	ไม่เห็นด้วย	ไม่เห็นด้วย	เห็นด้วย	ไม่เห็นด้วย	26.00
เห็นด้วย	เห็นด้วย	เห็นด้วยอย่	ไม่เห็นด้วย	ไม่เห็นด้วย	ไม่เห็นด้วย	ไม่เห็นด้วย	ไม่เห็นด้วย	26.00
เห็นด้วยอย่	ไม่เห็นด้วย	เห็นด้วยอย่	ไม่เห็นด้วย	เห็นด้วย	เห็นด้วย	เห็นด้วยอย่	ไม่เห็นด้วย	27.00
เห็นด้วยอย่	เห็นด้วยอย่	เห็นด้วยอย่	เห็นด้วย	เห็นด้วย	เห็นด้วย	เห็นด้วยอย่	เห็นด้วย	26.00
เห็นด้วยอย่	เห็นด้วยอย่	เห็นด้วย	เห็นด้วย	เห็นด้วยอย่	เห็นด้วยอย่	เห็นด้วย	เห็นด้วยอย่	29.00
เห็นด้วยอย่	เห็นด้วย	เห็นด้วยอย่	เห็นด้วย	เห็นด้วยอย่	เห็นด้วย	เห็นด้วยอย่	เห็นด้วย	30.00
เห็นด้วยอย่	เห็นด้วย	ไม่เห็นด้วย	เห็นด้วยอย่	เห็นด้วย	เห็นด้วย	เห็นด้วยอย่	เห็นด้วย	30.00
เห็นด้วย	เห็นด้วย	เห็นด้วย	เห็นด้วยอย่	เห็นด้วยอย่	เห็นด้วย	เห็นด้วยอย่	เห็นด้วยอย่	23.00

ไม่เห็นด้วย	เห็นด้วย	ไม่เห็นด้วย	เห็นด้วย	ไม่เห็นด้วย	เห็นด้วย	ไม่เห็นด้วย	ไม่เห็นด้วย	30.00
ไม่เห็นด้วยอ	ไม่เห็นด้วย	เห็นด้วย	ไม่เห็นด้วย	ไม่เห็นด้วย	ไม่เห็นด้วย	เห็นด้วย	เห็นด้วย	27.00
เห็นด้วย	เห็นด้วย	เห็นด้วย	เห็นด้วย	เห็นด้วย	เห็นด้วย	เห็นด้วย	ไม่เห็นด้วย	29.00
เห็นด้วย	ไม่เห็นด้วย	เห็นด้วย	เห็นด้วย	ไม่เห็นด้วย	ไม่เห็นด้วย	ไม่เห็นด้วย	ไม่เห็นด้วย	28.00
ไม่เห็นด้วยอ	เห็นด้วยอย่	เห็นด้วยอย่	เห็นด้วยอย่	เห็นด้วยอย่	ไม่เห็นด้วยอ	ไม่เห็นด้วยอ	ไม่เห็นด้วยอ	28.00
ไม่เห็นด้วย	เห็นด้วยอย่	เห็นด้วยอย่	เห็นด้วยอย่	ไม่เห็นด้วย	เห็นด้วยอย่	เห็นด้วยอย่	เห็นด้วยอย่	32.00
เห็นด้วยอย่	เห็นด้วยอย่	เห็นด้วยอย่	เห็นด้วย	เห็นด้วย	เห็นด้วย	เห็นด้วย	เห็นด้วย	22.00
เห็นด้วยอย่	เห็นด้วย	เห็นด้วยอย่	เห็นด้วย	เห็นด้วย	เห็นด้วยอย่	เห็นด้วยอย่	เห็นด้วย	27.00
เห็นด้วย	ไม่เห็นด้วย	เห็นด้วย	เห็นด้วย	ไม่เห็นด้วย	เห็นด้วยอย่	เห็นด้วย	เห็นด้วย	32.00
ไม่เห็นด้วย	เห็นด้วย	เห็นด้วย	ไม่เห็นด้วย	ไม่เห็นด้วย	ไม่เห็นด้วย	ไม่เห็นด้วย	ไม่เห็นด้วย	28.00
เห็นด้วย	ไม่เห็นด้วย	เห็นด้วย	ไม่เห็นด้วย	ไม่เห็นด้วย	เห็นด้วย	เห็นด้วย	เห็นด้วย	30.00
ไม่เห็นด้วย	เห็นด้วย	เห็นด้วย	เห็นด้วย	ไม่เห็นด้วย	เห็นด้วย	เห็นด้วย	ไม่เห็นด้วย	30.00
เห็นด้วย	เห็นด้วย	เห็นด้วย	เห็นด้วย	ไม่เห็นด้วย	ไม่เห็นด้วยอ	ไม่เห็นด้วย	ไม่เห็นด้วยอ	31.00
ไม่เห็นด้วย	ไม่เห็นด้วย	ไม่เห็นด้วย	เห็นด้วย	เห็นด้วย	เห็นด้วย	เห็นด้วย	เห็นด้วย	34.00
เห็นด้วยอย่	เห็นด้วย	เห็นด้วย	เห็นด้วย	เห็นด้วย	เห็นด้วย	เห็นด้วย	เห็นด้วย	28.00
เห็นด้วย	เห็นด้วย	เห็นด้วย	เห็นด้วย	ไม่เห็นด้วย	เห็นด้วย	เห็นด้วย	ไม่เห็นด้วย	40.00
เห็นด้วย	เห็นด้วย	เห็นด้วย	เห็นด้วยอย่	เห็นด้วย	เห็นด้วยอย่	เห็นด้วยอย่	เห็นด้วยอย่	31.00
เห็นด้วย	เห็นด้วย	เห็นด้วย	เห็นด้วย	เห็นด้วย	เห็นด้วย	เห็นด้วย	ไม่เห็นด้วย	27.00
เห็นด้วย	เห็นด้วย	เห็นด้วย	เห็นด้วย	เห็นด้วย	เห็นด้วย	เห็นด้วย	เห็นด้วย	30.00
เห็นด้วยอย่	เห็นด้วยอย่	เห็นด้วยอย่	เห็นด้วย	เห็นด้วย	เห็นด้วย	เห็นด้วย	เห็นด้วยอย่	31.00
เห็นด้วย	เห็นด้วย	เห็นด้วย	เห็นด้วย	เห็นด้วย	เห็นด้วย	เห็นด้วย	เห็นด้วย	28.00
เห็นด้วย	เห็นด้วยอย่	เห็นด้วยอย่	เห็นด้วย	เห็นด้วย	เห็นด้วย	เห็นด้วย	เห็นด้วย	29.00
ไม่เห็นด้วย	เห็นด้วย	เห็นด้วย	เห็นด้วย	เห็นด้วย	เห็นด้วย	เห็นด้วย	เห็นด้วย	22.00
ไม่เห็นด้วย	ไม่เห็นด้วย	ไม่เห็นด้วย	ไม่เห็นด้วย	ไม่เห็นด้วย	ไม่เห็นด้วย	ไม่เห็นด้วย	ไม่เห็นด้วย	33.00
เห็นด้วยอย่	ไม่เห็นด้วย	เห็นด้วย	ไม่เห็นด้วย	ไม่เห็นด้วย	เห็นด้วย	เห็นด้วยอย่	เห็นด้วย	26.00
เห็นด้วย	เห็นด้วย	เห็นด้วย	เห็นด้วย	เห็นด้วย	เห็นด้วย	เห็นด้วยอย่	เห็นด้วยอย่	28.00
ไม่เห็นด้วย	เห็นด้วยอย่	เห็นด้วย	เห็นด้วย	ไม่เห็นด้วย	เห็นด้วยอย่	เห็นด้วย	ไม่เห็นด้วย	26.00
เห็นด้วยอย่	ไม่เห็นด้วย	เห็นด้วยอย่	เห็นด้วย	ไม่เห็นด้วย	ไม่เห็นด้วย	เห็นด้วยอย่	เห็นด้วยอย่	30.00
เห็นด้วย	เห็นด้วยอย่	เห็นด้วยอย่	เห็นด้วย	ไม่เห็นด้วย	เห็นด้วย	เห็นด้วย	เห็นด้วย	22.00
เห็นด้วยอย่	ไม่เห็นด้วย	เห็นด้วย	เห็นด้วย	ไม่เห็นด้วย	เห็นด้วยอย่	เห็นด้วย	เห็นด้วย	30.00
เห็นด้วย	เห็นด้วย	เห็นด้วย	เห็นด้วย	เห็นด้วย	เห็นด้วย	เห็นด้วย	เห็นด้วย	27.00
ไม่เห็นด้วย	เห็นด้วยอย่	เห็นด้วยอย่	เห็นด้วยอย่	ไม่เห็นด้วย	เห็นด้วยอย่	เห็นด้วยอย่	ไม่เห็นด้วย	31.00
ไม่เห็นด้วยอ	ไม่เห็นด้วย	เห็นด้วย	ไม่เห็นด้วย	ไม่เห็นด้วย	ไม่เห็นด้วย	เห็นด้วย	เห็นด้วย	26.00
เห็นด้วย	ไม่เห็นด้วย	เห็นด้วย	เห็นด้วย	ไม่เห็นด้วย	ไม่เห็นด้วย	ไม่เห็นด้วย	ไม่เห็นด้วย	30.00
ไม่เห็นด้วยอ	เห็นด้วยอย่	เห็นด้วยอย่	เห็นด้วยอย่	เห็นด้วยอย่	ไม่เห็นด้วยอ	ไม่เห็นด้วยอ	ไม่เห็นด้วยอ	37.00
ไม่เห็นด้วย	เห็นด้วยอย่	เห็นด้วยอย่	เห็นด้วยอย่	ไม่เห็นด้วย	เห็นด้วยอย่	เห็นด้วยอย่	เห็นด้วยอย่	29.00
เห็นด้วยอย่	เห็นด้วย	เห็นด้วยอย่	เห็นด้วยอย่	เห็นด้วย	เห็นด้วย	เห็นด้วย	เห็นด้วย	28.00
เห็นด้วยอย่	เห็นด้วย	เห็นด้วยอย่	เห็นด้วย	เห็นด้วย	เห็นด้วยอย่	เห็นด้วยอย่	เห็นด้วย	25.00
เห็นด้วย	ไม่เห็นด้วย	เห็นด้วย	เห็นด้วย	ไม่เห็นด้วย	เห็นด้วยอย่	เห็นด้วย	เห็นด้วย	31.00
ไม่เห็นด้วย	เห็นด้วย	เห็นด้วย	ไม่เห็นด้วย	ไม่เห็นด้วย	ไม่เห็นด้วย	ไม่เห็นด้วย	ไม่เห็นด้วย	33.00
เห็นด้วย	ไม่เห็นด้วย	เห็นด้วย	ไม่เห็นด้วย	ไม่เห็นด้วย	เห็นด้วย	เห็นด้วย	เห็นด้วย	31.00
ไม่เห็นด้วย	เห็นด้วย	เห็นด้วย	เห็นด้วย	ไม่เห็นด้วย	เห็นด้วย	เห็นด้วย	ไม่เห็นด้วย	31.00
เห็นด้วย	เห็นด้วย	เห็นด้วย	เห็นด้วย	ไม่เห็นด้วย	ไม่เห็นด้วยอ	ไม่เห็นด้วย	ไม่เห็นด้วยอ	26.00
ไม่เห็นด้วย	ไม่เห็นด้วย	ไม่เห็นด้วย	เห็นด้วย	เห็นด้วย	เห็นด้วย	เห็นด้วย	ไม่เห็นด้วย	28.00
เห็นด้วยอย่	เห็นด้วย	เห็นด้วย	เห็นด้วย	เห็นด้วย	เห็นด้วย	เห็นด้วย	เห็นด้วย	34.00
เห็นด้วย	เห็นด้วย	เห็นด้วย	เห็นด้วย	ไม่เห็นด้วย	เห็นด้วย	เห็นด้วย	ไม่เห็นด้วย	40.00
เห็นด้วย	เห็นด้วย	เห็นด้วย	เห็นด้วยอย่	เห็นด้วย	เห็นด้วยอย่	เห็นด้วยอย่	เห็นด้วยอย่	30.00
เห็นด้วย	เห็นด้วย	เห็นด้วย	เห็นด้วย	เห็นด้วย	เห็นด้วย	เห็นด้วย	ไม่เห็นด้วย	23.00
เห็นด้วย	เห็นด้วย	เห็นด้วย	เห็นด้วย	เห็นด้วย	เห็นด้วย	เห็นด้วย	เห็นด้วย	30.00
เห็นด้วยอย่	เห็นด้วยอย่	เห็นด้วยอย่	เห็นด้วย	เห็นด้วย	เห็นด้วย	เห็นด้วย	เห็นด้วยอย่	30.00
เห็นด้วย	เห็นด้วย	เห็นด้วย	เห็นด้วย	เห็นด้วย	เห็นด้วย	เห็นด้วย	เห็นด้วย	24.00

เห็นด้วย	เห็นด้วยอย่	เห็นด้วยอย่	เห็นด้วย	เห็นด้วย	เห็นด้วย	เห็นด้วย	เห็นด้วย	25.00
ไม่เห็นด้วย	เห็นด้วย	เห็นด้วย	เห็นด้วย	เห็นด้วย	เห็นด้วย	เห็นด้วย	เห็นด้วย	24.00
ไม่เห็นด้วย	ไม่เห็นด้วย	ไม่เห็นด้วย	ไม่เห็นด้วย	ไม่เห็นด้วย	ไม่เห็นด้วย	ไม่เห็นด้วย	ไม่เห็นด้วย	29.00
เห็นด้วยอย่	เห็นด้วย	เห็นด้วย	ไม่เห็นด้วย	ไม่เห็นด้วย	เห็นด้วย	เห็นด้วยอย่	เห็นด้วย	31.00
เห็นด้วย	เห็นด้วย	เห็นด้วย	เห็นด้วย	เห็นด้วย	เห็นด้วย	เห็นด้วยอย่	เห็นด้วยอย่	30.00
เห็นด้วยอย่	เห็นด้วยอย่	เห็นด้วยอย่	เห็นด้วย	เห็นด้วย	เห็นด้วย	เห็นด้วยอย่	เห็นด้วย	31.00
เห็นด้วยอย่	เห็นด้วยอย่	เห็นด้วยอย่	เห็นด้วย	เห็นด้วย	เห็นด้วยอย่	เห็นด้วยอย่	เห็นด้วยอย่	40.00
เห็นด้วยอย่	เห็นด้วยอย่	เห็นด้วยอย่	เห็นด้วยอย่	เห็นด้วย	เห็นด้วยอย่	เห็นด้วยอย่	เห็นด้วยอย่	31.00
เห็นด้วย	เห็นด้วยอย่	เห็นด้วยอย่	เห็นด้วย	ไม่เห็นด้วย	เห็นด้วย	เห็นด้วยอย่	เห็นด้วย	25.00
ไม่เห็นด้วยอ	ไม่เห็นด้วย	เห็นด้วย	ไม่เห็นด้วย	ไม่เห็นด้วย	ไม่เห็นด้วย	เห็นด้วย	เห็นด้วย	31.00
เห็นด้วย	ไม่เห็นด้วย	เห็นด้วย	เห็นด้วย	ไม่เห็นด้วย	ไม่เห็นด้วย	ไม่เห็นด้วย	ไม่เห็นด้วย	33.00
ไม่เห็นด้วยอ	เห็นด้วยอย่	เห็นด้วยอย่	เห็นด้วยอย่	เห็นด้วยอย่	ไม่เห็นด้วยอ	ไม่เห็นด้วยอ	ไม่เห็นด้วยอ	28.00
ไม่เห็นด้วย	เห็นด้วย	เห็นด้วย	เห็นด้วย	ไม่เห็นด้วย	เห็นด้วย	เห็นด้วย	เห็นด้วย	20.00
เห็นด้วยอย่	เห็นด้วย	เห็นด้วยอย่	เห็นด้วยอย่	เห็นด้วย	เห็นด้วย	เห็นด้วย	เห็นด้วย	35.00
เห็นด้วยอย่	เห็นด้วย	เห็นด้วยอย่	เห็นด้วย	เห็นด้วย	เห็นด้วยอย่	เห็นด้วยอย่	เห็นด้วย	29.00
เห็นด้วย	ไม่เห็นด้วย	เห็นด้วย	เห็นด้วย	ไม่เห็นด้วย	เห็นด้วยอย่	เห็นด้วย	เห็นด้วย	33.00
ไม่เห็นด้วย	เห็นด้วย	เห็นด้วย	ไม่เห็นด้วย	ไม่เห็นด้วย	ไม่เห็นด้วย	ไม่เห็นด้วย	ไม่เห็นด้วย	34.00
เห็นด้วย	ไม่เห็นด้วย	เห็นด้วย	ไม่เห็นด้วย	ไม่เห็นด้วย	เห็นด้วย	เห็นด้วย	เห็นด้วย	29.00
ไม่เห็นด้วย	เห็นด้วย	เห็นด้วย	เห็นด้วย	ไม่เห็นด้วย	เห็นด้วย	เห็นด้วย	ไม่เห็นด้วย	36.00
เห็นด้วย	เห็นด้วย	เห็นด้วย	เห็นด้วย	ไม่เห็นด้วย	ไม่เห็นด้วยอ	ไม่เห็นด้วย	ไม่เห็นด้วยอ	28.00
ไม่เห็นด้วย	ไม่เห็นด้วย	ไม่เห็นด้วย	เห็นด้วย	เห็นด้วย	เห็นด้วย	เห็นด้วย	ไม่เห็นด้วย	34.00
เห็นด้วยอย่	เห็นด้วย	เห็นด้วย	เห็นด้วย	เห็นด้วย	เห็นด้วย	เห็นด้วย	เห็นด้วย	34.00
เห็นด้วย	เห็นด้วย	เห็นด้วย	ไม่เห็นด้วย	เห็นด้วย	เห็นด้วย	เห็นด้วย	ไม่เห็นด้วย	26.00
เห็นด้วย	เห็นด้วย	เห็นด้วย	เห็นด้วยอย่	เห็นด้วย	เห็นด้วยอย่	เห็นด้วยอย่	เห็นด้วยอย่	22.00
เห็นด้วย	เห็นด้วย	เห็นด้วย	เห็นด้วย	เห็นด้วย	เห็นด้วย	เห็นด้วย	เห็นด้วย	24.00
ไม่เห็นด้วย	ไม่เห็นด้วย	เห็นด้วย	ไม่เห็นด้วย	ไม่เห็นด้วย	ไม่เห็นด้วย	เห็นด้วย	ไม่เห็นด้วย	32.00
เห็นด้วย	เห็นด้วย	เห็นด้วย	เห็นด้วย	เห็นด้วย	เห็นด้วย	เห็นด้วย	เห็นด้วย	30.00
เห็นด้วย	เห็นด้วย	เห็นด้วย	ไม่เห็นด้วย	ไม่เห็นด้วย	ไม่เห็นด้วย	เห็นด้วย	ไม่เห็นด้วย	35.00
เห็นด้วยอย่	เห็นด้วยอย่	เห็นด้วยอย่	เห็นด้วยอย่	เห็นด้วยอย่	เห็นด้วย	เห็นด้วย	เห็นด้วย	33.00
เห็นด้วย	เห็นด้วย	เห็นด้วย	เห็นด้วย	เห็นด้วย	เห็นด้วย	เห็นด้วย	เห็นด้วย	40.00
เห็นด้วยอย่	เห็นด้วยอย่	เห็นด้วยอย่	เห็นด้วยอย่	เห็นด้วยอย่	เห็นด้วยอย่	เห็นด้วยอย่	เห็นด้วยอย่	27.00
เห็นด้วยอย่	เห็นด้วยอย่	เห็นด้วยอย่	เห็นด้วยอย่	เห็นด้วยอย่	เห็นด้วยอย่	เห็นด้วยอย่	เห็นด้วยอย่	26.00
เห็นด้วย	เห็นด้วยอย่	เห็นด้วยอย่	เห็นด้วยอย่	เห็นด้วย	เห็นด้วย	เห็นด้วย	เห็นด้วย	32.00
เห็นด้วยอย่	เห็นด้วย	เห็นด้วย	เห็นด้วยอย่	เห็นด้วย	เห็นด้วยอย่	เห็นด้วยอย่	เห็นด้วยอย่	30.00
เห็นด้วยอย่	เห็นด้วยอย่	เห็นด้วยอย่	เห็นด้วยอย่	เห็นด้วยอย่	เห็นด้วยอย่	เห็นด้วยอย่	เห็นด้วยอย่	29.00
เห็นด้วยอย่	เห็นด้วยอย่	เห็นด้วยอย่	เห็นด้วยอย่	เห็นด้วยอย่	เห็นด้วยอย่	เห็นด้วยอย่	เห็นด้วยอย่	34.00
เห็นด้วยอย่	เห็นด้วยอย่	เห็นด้วยอย่	เห็นด้วยอย่	เห็นด้วยอย่	เห็นด้วยอย่	เห็นด้วยอย่	เห็นด้วยอย่	30.00
เห็นด้วย	เห็นด้วย	เห็นด้วย	เห็นด้วย	เห็นด้วย	เห็นด้วย	เห็นด้วย	เห็นด้วย	30.00
เห็นด้วย	เห็นด้วย	เห็นด้วย	เห็นด้วย	เห็นด้วย	เห็นด้วย	เห็นด้วย	เห็นด้วย	34.00
เห็นด้วย	เห็นด้วย	เห็นด้วย	เห็นด้วยอย่	เห็นด้วยอย่	เห็นด้วยอย่	เห็นด้วยอย่	เห็นด้วยอย่	29.00
เห็นด้วย	เห็นด้วย	เห็นด้วย	เห็นด้วย	เห็นด้วย	เห็นด้วย	เห็นด้วย	เห็นด้วย	27.00
เห็นด้วยอย่	เห็นด้วยอย่	เห็นด้วยอย่	เห็นด้วยอย่	เห็นด้วยอย่	เห็นด้วยอย่	เห็นด้วยอย่	เห็นด้วยอย่	29.00
เห็นด้วยอย่	เห็นด้วยอย่	เห็นด้วยอย่	เห็นด้วยอย่	เห็นด้วยอย่	เห็นด้วยอย่	เห็นด้วยอย่	เห็นด้วยอย่	32.00
เห็นด้วย	เห็นด้วย	เห็นด้วย	เห็นด้วย	เห็นด้วย	เห็นด้วย	เห็นด้วย	เห็นด้วย	25.00
เห็นด้วย	เห็นด้วย	เห็นด้วย	เห็นด้วย	เห็นด้วย	เห็นด้วย	เห็นด้วย	เห็นด้วย	27.00
เห็นด้วย	เห็นด้วย	เห็นด้วย	เห็นด้วย	เห็นด้วย	เห็นด้วย	เห็นด้วย	เห็นด้วย	35.00
เห็นด้วยอย่	เห็นด้วยอย่	เห็นด้วยอย่	เห็นด้วยอย่	เห็นด้วยอย่	เห็นด้วยอย่	เห็นด้วยอย่	เห็นด้วยอย่	30.00
เห็นด้วย	เห็นด้วย	เห็นด้วย	เห็นด้วย	เห็นด้วย	เห็นด้วย	เห็นด้วย	เห็นด้วย	32.00
เห็นด้วย	เห็นด้วย	เห็นด้วย	เห็นด้วย	เห็นด้วย	เห็นด้วย	เห็นด้วย	เห็นด้วย	27.00
เห็นด้วย	ไม่เห็นด้วย	เห็นด้วย	ไม่เห็นด้วย	ไม่เห็นด้วย	เห็นด้วย	เห็นด้วยอย่	เห็นด้วย	30.00
เห็นด้วยอย่	เห็นด้วย	ไม่เห็นด้วย	เห็นด้วย	เห็นด้วย	เห็นด้วยอย่	เห็นด้วยอย่	เห็นด้วย	25.00

เห็นด้วย	เห็นด้วย	เห็นด้วย	เห็นด้วย	เห็นด้วย	เห็นด้วย	เห็นด้วย	เห็นด้วย	26.00
ไม่เห็นด้วย	เห็นด้วย	เห็นด้วย	เห็นด้วย	เห็นด้วย	เห็นด้วย	ไม่เห็นด้วย	เห็นด้วย	33.00
เห็นด้วยอย่	เห็นด้วย	เห็นด้วยอย่	เห็นด้วย	ไม่เห็นด้วย	เห็นด้วย	เห็นด้วย	ไม่เห็นด้วย	23.00
เห็นด้วย	เห็นด้วย	เห็นด้วย	ไม่เห็นด้วย	ไม่เห็นด้วย	ไม่เห็นด้วย	เห็นด้วย	ไม่เห็นด้วย	29.00
เห็นด้วยอย่	เห็นด้วย	เห็นด้วยอย่	เห็นด้วยอย่	เห็นด้วย	เห็นด้วย	เห็นด้วย	เห็นด้วย	30.00
เห็นด้วยอย่	เห็นด้วยอย่	เห็นด้วยอย่	เห็นด้วย	เห็นด้วย	เห็นด้วย	เห็นด้วย	เห็นด้วย	27.00
ไม่เห็นด้วย	เห็นด้วย	เห็นด้วย	เห็นด้วย	เห็นด้วย	เห็นด้วย	เห็นด้วย	เห็นด้วย	31.00
เห็นด้วยอย่	เห็นด้วยอย่	เห็นด้วยอย่	เห็นด้วยอย่	เห็นด้วย	เห็นด้วย	เห็นด้วยอย่	เห็นด้วยอย่	31.00
เห็นด้วยอย่	เห็นด้วยอย่	เห็นด้วยอย่	เห็นด้วยอย่	เห็นด้วย	เห็นด้วยอย่	เห็นด้วยอย่	เห็นด้วยอย่	30.00
เห็นด้วย	เห็นด้วย	เห็นด้วยอย่	เห็นด้วย	ไม่เห็นด้วย	เห็นด้วย	เห็นด้วยอย่	เห็นด้วย	30.00
เห็นด้วยอย่	เห็นด้วยอย่	เห็นด้วยอย่	เห็นด้วยอย่	เห็นด้วย	เห็นด้วย	เห็นด้วยอย่	เห็นด้วยอย่	30.00
เห็นด้วย	เห็นด้วย	เห็นด้วย	เห็นด้วย	เห็นด้วย	เห็นด้วย	เห็นด้วย	เห็นด้วย	31.00
เห็นด้วยอย่	เห็นด้วยอย่	เห็นด้วยอย่	เห็นด้วยอย่	เห็นด้วย	เห็นด้วยอย่	ไม่เห็นด้วย	ไม่เห็นด้วย	26.00
เห็นด้วยอย่	เห็นด้วยอย่	เห็นด้วยอย่	เห็นด้วยอย่	เห็นด้วยอย่	เห็นด้วย	เห็นด้วยอย่	เห็นด้วยอย่	31.00
เห็นด้วย	เห็นด้วย	เห็นด้วย	เห็นด้วย	เห็นด้วย	เห็นด้วย	เห็นด้วย	เห็นด้วย	30.00
ไม่เห็นด้วยอ	เห็นด้วย	เห็นด้วย	เห็นด้วย	ไม่เห็นด้วยอ	เห็นด้วย	เห็นด้วย	ไม่เห็นด้วยอ	33.00
เห็นด้วย	เห็นด้วย	เห็นด้วย	เห็นด้วย	ไม่เห็นด้วย	เห็นด้วย	เห็นด้วย	เห็นด้วย	27.00
เห็นด้วยอย่	เห็นด้วยอย่	เห็นด้วยอย่	เห็นด้วยอย่	ไม่เห็นด้วย	เห็นด้วย	เห็นด้วย	ไม่เห็นด้วย	30.00
เห็นด้วยอย่	เห็นด้วยอย่	เห็นด้วยอย่	เห็นด้วย	เห็นด้วย	ไม่เห็นด้วย	เห็นด้วย	ไม่เห็นด้วย	26.00
เห็นด้วยอย่	เห็นด้วยอย่	เห็นด้วยอย่	เห็นด้วยอย่	เห็นด้วย	เห็นด้วย	เห็นด้วย	เห็นด้วย	40.00
เห็นด้วย	เห็นด้วย	เห็นด้วย	เห็นด้วย	เห็นด้วย	เห็นด้วย	เห็นด้วย	เห็นด้วย	31.00
เห็นด้วย	เห็นด้วย	เห็นด้วย	เห็นด้วย	เห็นด้วย	เห็นด้วย	เห็นด้วย	เห็นด้วย	30.00
เห็นด้วยอย่	เห็นด้วย	เห็นด้วย	เห็นด้วย	เห็นด้วย	เห็นด้วย	เห็นด้วย	เห็นด้วย	33.00
เห็นด้วย	เห็นด้วยอย่	เห็นด้วย	เห็นด้วย	ไม่เห็นด้วย	เห็นด้วย	เห็นด้วย	เห็นด้วย	37.00
เห็นด้วยอย่	เห็นด้วย	เห็นด้วย	เห็นด้วย	เห็นด้วย	เห็นด้วย	เห็นด้วย	เห็นด้วย	35.00
เห็นด้วย	เห็นด้วย	เห็นด้วยอย่	เห็นด้วยอย่	ไม่เห็นด้วย	เห็นด้วย	เห็นด้วย	เห็นด้วย	30.00
ไม่เห็นด้วยอ	เห็นด้วย	เห็นด้วย	เห็นด้วย	ไม่เห็นด้วย	เห็นด้วย	ไม่เห็นด้วย	ไม่เห็นด้วยอ	29.00
เห็นด้วย	เห็นด้วย	เห็นด้วย	เห็นด้วย	เห็นด้วย	เห็นด้วย	เห็นด้วย	เห็นด้วย	28.00
ไม่เห็นด้วยอ	ไม่เห็นด้วย	เห็นด้วย	เห็นด้วย	เห็นด้วย	เห็นด้วย	เห็นด้วย	เห็นด้วย	28.00
ไม่เห็นด้วย	ไม่เห็นด้วย	เห็นด้วยอย่	ไม่เห็นด้วย	ไม่เห็นด้วย	เห็นด้วยอย่	เห็นด้วย	ไม่เห็นด้วย	38.00
เห็นด้วยอย่	เห็นด้วย	เห็นด้วย	เห็นด้วย	ไม่เห็นด้วยอ	ไม่เห็นด้วย	เห็นด้วย	เห็นด้วย	26.00
ไม่เห็นด้วย	ไม่เห็นด้วย	เห็นด้วย	เห็นด้วย	ไม่เห็นด้วย	เห็นด้วย	เห็นด้วย	ไม่เห็นด้วย	26.00
เห็นด้วยอย่	เห็นด้วย	เห็นด้วย	เห็นด้วย	เห็นด้วย	เห็นด้วย	เห็นด้วย	เห็นด้วย	28.00
เห็นด้วยอย่	เห็นด้วย	เห็นด้วย	เห็นด้วย	เห็นด้วย	เห็นด้วยอย่	เห็นด้วยอย่	เห็นด้วย	32.00
เห็นด้วย	เห็นด้วย	เห็นด้วยอย่	เห็นด้วย	เห็นด้วย	เห็นด้วย	ไม่เห็นด้วย	ไม่เห็นด้วย	29.00
เห็นด้วย	เห็นด้วย	เห็นด้วย	เห็นด้วย	ไม่เห็นด้วย	เห็นด้วยอย่	เห็นด้วยอย่	ไม่เห็นด้วย	29.00
เห็นด้วย	เห็นด้วย	เห็นด้วยอย่	เห็นด้วย	ไม่เห็นด้วย	เห็นด้วย	เห็นด้วย	ไม่เห็นด้วย	38.00
ไม่เห็นด้วย	เห็นด้วย	เห็นด้วย	เห็นด้วย	ไม่เห็นด้วย	เห็นด้วย	เห็นด้วย	เห็นด้วย	29.00
เห็นด้วยอย่	เห็นด้วย	เห็นด้วยอย่	เห็นด้วย	ไม่เห็นด้วย	เห็นด้วย	เห็นด้วยอย่	เห็นด้วย	33.00
เห็นด้วย	เห็นด้วย	เห็นด้วย	เห็นด้วย	เห็นด้วย	เห็นด้วย	เห็นด้วย	เห็นด้วย	30.00
เห็นด้วย	ไม่เห็นด้วย	เห็นด้วย	ไม่เห็นด้วย	ไม่เห็นด้วย	เห็นด้วย	เห็นด้วยอย่	เห็นด้วย	30.00
เห็นด้วยอย่	เห็นด้วย	ไม่เห็นด้วย	เห็นด้วย	เห็นด้วย	เห็นด้วยอย่	เห็นด้วยอย่	เห็นด้วย	27.00
เห็นด้วย	เห็นด้วย	เห็นด้วย	เห็นด้วย	เห็นด้วย	เห็นด้วย	เห็นด้วย	เห็นด้วย	24.00
ไม่เห็นด้วย	เห็นด้วย	เห็นด้วย	เห็นด้วย	เห็นด้วย	เห็นด้วย	ไม่เห็นด้วย	เห็นด้วย	27.00
เห็นด้วยอย่	เห็นด้วย	เห็นด้วยอย่	เห็นด้วย	ไม่เห็นด้วย	เห็นด้วย	เห็นด้วย	ไม่เห็นด้วย	32.00
เห็นด้วย	เห็นด้วย	เห็นด้วย	ไม่เห็นด้วย	ไม่เห็นด้วย	ไม่เห็นด้วย	เห็นด้วย	ไม่เห็นด้วย	28.00
เห็นด้วย	เห็นด้วย	เห็นด้วย	เห็นด้วย	เห็นด้วย	เห็นด้วย	เห็นด้วย	เห็นด้วย	34.00
เห็นด้วย	เห็นด้วย	เห็นด้วย	ไม่เห็นด้วย	เห็นด้วยอย่	เห็นด้วยอย่	เห็นด้วยอย่	เห็นด้วยอย่	30.00
ไม่เห็นด้วย	เห็นด้วย	เห็นด้วย	เห็นด้วย	เห็นด้วย	ไม่เห็นด้วย	ไม่เห็นด้วย	เห็นด้วย	31.00

severe1	severe2	severe3	severe4	severe5	severe6	Sum_Seve	benefit1	benefit2
เห็นด้วย	ไม่เห็นด้วย	เห็นด้วย	เห็นด้วย	เห็นด้วย	ไม่เห็นด้วย	21.00	ไม่เห็นด้วย	ไม่เห็นด้วย
เห็นด้วย	เห็นด้วย	เห็นด้วย	เห็นด้วย	เห็นด้วย	เห็นด้วย	24.00	เห็นด้วย	ไม่เห็นด้วย
เห็นด้วยอย่	เห็นด้วย	เห็นด้วย	ไม่เห็นด้วย	เห็นด้วย	ไม่เห็นด้วย	24.00	เห็นด้วย	เห็นด้วย
เห็นด้วยอย่	เห็นด้วยอย่	เห็นด้วยอย่	เห็นด้วยอย่	เห็นด้วยอย่	เห็นด้วย	18.00	เห็นด้วย	เห็นด้วยอย่
เห็นด้วยอย่	เห็นด้วย	เห็นด้วย	เห็นด้วย	เห็นด้วย	ไม่เห็นด้วย	18.00	เห็นด้วย	เห็นด้วย
เห็นด้วย	เห็นด้วย	เห็นด้วย	เห็นด้วย	เห็นด้วย	เห็นด้วย	20.00	เห็นด้วย	เห็นด้วย
เห็นด้วยอย่	เห็นด้วย	เห็นด้วยอย่	เห็นด้วยอย่	เห็นด้วย	เห็นด้วยอย่	17.00	เห็นด้วย	เห็นด้วย
เห็นด้วยอย่	เห็นด้วยอย่	เห็นด้วยอย่	เห็นด้วยอย่	เห็นด้วยอย่	เห็นด้วยอย่	21.00	เห็นด้วยอย่	เห็นด้วยอย่
เห็นด้วย	เห็นด้วย	ไม่เห็นด้วย	ไม่เห็นด้วย	เห็นด้วย	เห็นด้วย	23.00	เห็นด้วย	ไม่เห็นด้วย
เห็นด้วย	เห็นด้วยอย่	เห็นด้วยอย่	เห็นด้วยอย่	เห็นด้วย	เห็นด้วย	18.00	เห็นด้วย	เห็นด้วยอย่
เห็นด้วย	เห็นด้วย	เห็นด้วย	เห็นด้วย	เห็นด้วย	ไม่เห็นด้วย	19.00	เห็นด้วย	เห็นด้วย
เห็นด้วย	เห็นด้วย	เห็นด้วย	เห็นด้วย	เห็นด้วย	เห็นด้วย	18.00	เห็นด้วยอย่	เห็นด้วย
เห็นด้วย	เห็นด้วย	เห็นด้วย	เห็นด้วย	เห็นด้วย	เห็นด้วย	22.00	เห็นด้วย	เห็นด้วย
เห็นด้วย	เห็นด้วย	เห็นด้วย	เห็นด้วย	เห็นด้วย	เห็นด้วย	16.00	เห็นด้วยอย่	เห็นด้วย
เห็นด้วยอย่	เห็นด้วยอย่	เห็นด้วย	เห็นด้วย	เห็นด้วย	เห็นด้วย	19.00	เห็นด้วย	เห็นด้วย
เห็นด้วยอย่	เห็นด้วยอย่	เห็นด้วยอย่	เห็นด้วย	เห็นด้วย	เห็นด้วย	18.00	เห็นด้วย	เห็นด้วย
เห็นด้วย	เห็นด้วยอย่	เห็นด้วย	เห็นด้วย	เห็นด้วย	เห็นด้วย	18.00	เห็นด้วยอย่	เห็นด้วยอย่
เห็นด้วย	เห็นด้วย	เห็นด้วย	เห็นด้วย	เห็นด้วยอย่	ไม่เห็นด้วย	18.00	เห็นด้วยอย่	เห็นด้วย
เห็นด้วย	เห็นด้วยอย่	ไม่เห็นด้วย	เห็นด้วย	เห็นด้วย	ไม่เห็นด้วย	16.00	เห็นด้วยอย่	เห็นด้วยอย่
เห็นด้วย	เห็นด้วย	เห็นด้วย	เห็นด้วย	เห็นด้วย	เห็นด้วย	16.00	เห็นด้วย	เห็นด้วย
เห็นด้วย	เห็นด้วย	เห็นด้วย	เห็นด้วย	เห็นด้วย	เห็นด้วย	18.00	เห็นด้วย	เห็นด้วย
ไม่เห็นด้วย	ไม่เห็นด้วย	ไม่เห็นด้วย	ไม่เห็นด้วย	ไม่เห็นด้วย	เห็นด้วย	19.00	เห็นด้วย	ไม่เห็นด้วย
เห็นด้วยอย่	เห็นด้วยอย่	เห็นด้วยอย่	เห็นด้วยอย่	เห็นด้วย	เห็นด้วยอย่	21.00	เห็นด้วยอย่	เห็นด้วยอย่
เห็นด้วยอย่	เห็นด้วย	เห็นด้วย	ไม่เห็นด้วย	เห็นด้วย	เห็นด้วย	18.00	เห็นด้วย	ไม่เห็นด้วย
เห็นด้วยอย่	เห็นด้วยอย่	เห็นด้วยอย่	เห็นด้วยอย่	เห็นด้วย	เห็นด้วย	18.00	เห็นด้วยอย่	เห็นด้วยอย่
เห็นด้วยอย่	เห็นด้วยอย่	เห็นด้วยอย่	เห็นด้วย	เห็นด้วย	เห็นด้วย	17.00	เห็นด้วยอย่	เห็นด้วยอย่
เห็นด้วยอย่	เห็นด้วยอย่	เห็นด้วยอย่	เห็นด้วยอย่	เห็นด้วยอย่	เห็นด้วยอย่	22.00	เห็นด้วยอย่	เห็นด้วยอย่
เห็นด้วย	เห็นด้วย	เห็นด้วย	เห็นด้วยอย่	เห็นด้วยอย่	ไม่เห็นด้วย	18.00	เห็นด้วยอย่	เห็นด้วยอย่
เห็นด้วยอย่	เห็นด้วยอย่	เห็นด้วยอย่	เห็นด้วยอย่	เห็นด้วยอย่	ไม่เห็นด้วย	20.00	เห็นด้วยอย่	เห็นด้วยอย่
ไม่เห็นด้วย	เห็นด้วย	เห็นด้วย	เห็นด้วย	เห็นด้วย	เห็นด้วย	18.00	เห็นด้วย	เห็นด้วย
เห็นด้วย	เห็นด้วย	เห็นด้วย	เห็นด้วย	เห็นด้วย	เห็นด้วย	17.00	เห็นด้วย	เห็นด้วย
เห็นด้วยอย่	เห็นด้วยอย่	ไม่เห็นด้วย	เห็นด้วย	เห็นด้วย	ไม่เห็นด้วย	15.00	เห็นด้วย	เห็นด้วย
เห็นด้วย	เห็นด้วย	เห็นด้วย	เห็นด้วย	เห็นด้วย	เห็นด้วย	23.00	เห็นด้วย	เห็นด้วย
เห็นด้วยอย่	เห็นด้วยอย่	เห็นด้วยอย่	เห็นด้วยอย่	เห็นด้วยอย่	เห็นด้วยอย่	18.00	เห็นด้วยอย่	เห็นด้วยอย่
เห็นด้วยอย่	เห็นด้วยอย่	เห็นด้วย	เห็นด้วย	เห็นด้วย	ไม่เห็นด้วย	17.00	เห็นด้วย	เห็นด้วย
เห็นด้วย	เห็นด้วย	เห็นด้วย	เห็นด้วย	เห็นด้วย	เห็นด้วย	18.00	เห็นด้วย	เห็นด้วย
เห็นด้วยอย่	เห็นด้วยอย่	เห็นด้วย	เห็นด้วยอย่	เห็นด้วยอย่	ไม่เห็นด้วย	20.00	ไม่เห็นด้วย	เห็นด้วย
เห็นด้วย	เห็นด้วย	ไม่เห็นด้วย	เห็นด้วย	เห็นด้วย	เห็นด้วย	18.00	เห็นด้วย	เห็นด้วย
เห็นด้วย	เห็นด้วย	เห็นด้วย	เห็นด้วย	เห็นด้วย	เห็นด้วย	17.00	เห็นด้วย	เห็นด้วย
เห็นด้วย	เห็นด้วย	เห็นด้วย	เห็นด้วย	เห็นด้วย	เห็นด้วย	18.00	เห็นด้วย	เห็นด้วย
เห็นด้วยอย่	เห็นด้วย	เห็นด้วยอย่	เห็นด้วย	เห็นด้วย	เห็นด้วย	24.00	เห็นด้วย	เห็นด้วย
เห็นด้วย	เห็นด้วย	ไม่เห็นด้วย	เห็นด้วย	ไม่เห็นด้วย	ไม่เห็นด้วย	16.00	ไม่เห็นด้วย	เห็นด้วย
เห็นด้วย	เห็นด้วย	เห็นด้วย	เห็นด้วย	ไม่เห็นด้วย	เห็นด้วย	18.00	เห็นด้วยอย่	เห็นด้วยอย่
เห็นด้วย	เห็นด้วย	เห็นด้วย	เห็นด้วย	เห็นด้วย	เห็นด้วย	18.00	เห็นด้วย	เห็นด้วย
เห็นด้วย	เห็นด้วยอย่	เห็นด้วย	เห็นด้วย	เห็นด้วยอย่	เห็นด้วย	18.00	เห็นด้วยอย่	เห็นด้วยอย่
เห็นด้วย	เห็นด้วย	เห็นด้วย	เห็นด้วย	เห็นด้วย	ไม่เห็นด้วย	17.00	เห็นด้วย	เห็นด้วย
เห็นด้วยอย่	เห็นด้วย	เห็นด้วย	เห็นด้วยอย่	เห็นด้วยอย่	เห็นด้วย	18.00	เห็นด้วย	เห็นด้วยอย่
เห็นด้วย	เห็นด้วย	เห็นด้วย	เห็นด้วย	เห็นด้วย	เห็นด้วย	19.00	เห็นด้วยอย่	เห็นด้วยอย่
เห็นด้วย	เห็นด้วย	เห็นด้วย	เห็นด้วย	เห็นด้วย	เห็นด้วย	24.00	เห็นด้วย	เห็นด้วย
ไม่เห็นด้วย	เห็นด้วย	เห็นด้วย	เห็นด้วย	เห็นด้วย	เห็นด้วย	18.00	เห็นด้วย	เห็นด้วย

เห็นด้วยอย่	เห็นด้วย	เห็นด้วย	เห็นด้วย	เห็นด้วย	เห็นด้วย	24.00	เห็นด้วย	เห็นด้วย
เห็นด้วยอย่	เห็นด้วย	เห็นด้วยอย่	เห็นด้วย	เห็นด้วย	ไม่เห็นด้วย	22.00	เห็นด้วยอย่	เห็นด้วย
เห็นด้วย	เห็นด้วยอย่	เห็นด้วย	เห็นด้วย	เห็นด้วย	เห็นด้วย	20.00	เห็นด้วย	เห็นด้วย
เห็นด้วย	เห็นด้วย	เห็นด้วย	เห็นด้วยอย่	เห็นด้วยอย่	เห็นด้วยอย่	22.00	เห็นด้วยอย่	เห็นด้วยอย่
เห็นด้วยอย่	เห็นด้วยอย่	เห็นด้วยอย่	เห็นด้วยอย่	เห็นด้วย	เห็นด้วยอย่	19.00	เห็นด้วยอย่	เห็นด้วยอย่
เห็นด้วยอย่	เห็นด้วยอย่	เห็นด้วย	เห็นด้วย	เห็นด้วยอย่	เห็นด้วย	24.00	เห็นด้วยอย่	เห็นด้วย
เห็นด้วย	เห็นด้วย	เห็นด้วย	เห็นด้วย	เห็นด้วย	เห็นด้วย	19.00	เห็นด้วย	เห็นด้วย
เห็นด้วย	เห็นด้วย	เห็นด้วย	เห็นด้วย	เห็นด้วย	เห็นด้วย	19.00	เห็นด้วย	เห็นด้วยอย่
เห็นด้วย	เห็นด้วยอย่	เห็นด้วย	เห็นด้วยอย่	เห็นด้วย	ไม่เห็นด้วย	19.00	เห็นด้วย	เห็นด้วย
เห็นด้วยอย่	เห็นด้วย	เห็นด้วย	เห็นด้วย	เห็นด้วย	ไม่เห็นด้วย	22.00	เห็นด้วย	เห็นด้วย
เห็นด้วย	เห็นด้วย	เห็นด้วย	เห็นด้วย	เห็นด้วย	เห็นด้วย	21.00	เห็นด้วย	เห็นด้วย
เห็นด้วยอย่	เห็นด้วยอย่	เห็นด้วย	เห็นด้วย	เห็นด้วย	ไม่เห็นด้วย	23.00	เห็นด้วยอย่	เห็นด้วย
เห็นด้วยอย่	เห็นด้วยอย่	เห็นด้วยอย่	เห็นด้วยอย่	เห็นด้วยอย่	เห็นด้วยอย่	18.00	เห็นด้วยอย่	เห็นด้วยอย่
เห็นด้วยอย่	เห็นด้วยอย่	ไม่เห็นด้วย	เห็นด้วยอย่	เห็นด้วยอย่	ไม่เห็นด้วย	21.00	เห็นด้วยอย่	เห็นด้วย
เห็นด้วยอย่	เห็นด้วยอย่	เห็นด้วยอย่	เห็นด้วยอย่	เห็นด้วยอย่	เห็นด้วยอย่	24.00	เห็นด้วยอย่	เห็นด้วยอย่
เห็นด้วย	เห็นด้วย	เห็นด้วย	เห็นด้วย	เห็นด้วย	เห็นด้วยอย่	23.00	เห็นด้วย	เห็นด้วย
เห็นด้วย	เห็นด้วย	เห็นด้วย	เห็นด้วย	เห็นด้วย	เห็นด้วย	16.00	เห็นด้วย	เห็นด้วย
เห็นด้วย	เห็นด้วย	เห็นด้วย	เห็นด้วย	เห็นด้วย	เห็นด้วย	18.00	เห็นด้วย	เห็นด้วย
เห็นด้วย	เห็นด้วย	เห็นด้วย	เห็นด้วย	เห็นด้วย	เห็นด้วย	19.00	เห็นด้วย	เห็นด้วย
เห็นด้วย	เห็นด้วย	เห็นด้วย	เห็นด้วย	เห็นด้วยอย่	เห็นด้วยอย่	22.00	เห็นด้วย	เห็นด้วย
เห็นด้วย	เห็นด้วยอย่	เห็นด้วยอย่	เห็นด้วยอย่	เห็นด้วยอย่	เห็นด้วยอย่	19.00	เห็นด้วย	เห็นด้วย
เห็นด้วยอย่	เห็นด้วยอย่	เห็นด้วยอย่	เห็นด้วยอย่	เห็นด้วยอย่	เห็นด้วยอย่	16.00	เห็นด้วย	เห็นด้วย
เห็นด้วยอย่	เห็นด้วยอย่	เห็นด้วย	เห็นด้วยอย่	เห็นด้วยอย่	เห็นด้วย	17.00	เห็นด้วย	เห็นด้วย
เห็นด้วย	เห็นด้วย	เห็นด้วย	เห็นด้วย	เห็นด้วย	เห็นด้วย	16.00	เห็นด้วย	เห็นด้วย
เห็นด้วย	เห็นด้วย	เห็นด้วย	เห็นด้วย	เห็นด้วย	เห็นด้วย	18.00	เห็นด้วย	เห็นด้วย
เห็นด้วย	เห็นด้วย	เห็นด้วย	เห็นด้วย	เห็นด้วย	เห็นด้วย	18.00	เห็นด้วย	เห็นด้วย
เห็นด้วยอย่	เห็นด้วยอย่	เห็นด้วยอย่	เห็นด้วยอย่	เห็นด้วยอย่	เห็นด้วยอย่	18.00	เห็นด้วย	เห็นด้วย
เห็นด้วย	เห็นด้วย	เห็นด้วย	เห็นด้วย	เห็นด้วย	เห็นด้วย	18.00	เห็นด้วย	เห็นด้วย
เห็นด้วย	เห็นด้วย	เห็นด้วย	เห็นด้วย	เห็นด้วย	เห็นด้วย	18.00	เห็นด้วย	เห็นด้วย
เห็นด้วย	เห็นด้วย	เห็นด้วย	เห็นด้วย	เห็นด้วย	เห็นด้วย	16.00	เห็นด้วยอย่	เห็นด้วยอย่
เห็นด้วย	เห็นด้วย	เห็นด้วย	เห็นด้วย	เห็นด้วย	เห็นด้วย	20.00	เห็นด้วย	เห็นด้วย
เห็นด้วย	เห็นด้วย	เห็นด้วย	เห็นด้วย	เห็นด้วย	เห็นด้วย	24.00	เห็นด้วย	เห็นด้วย
เห็นด้วย	เห็นด้วย	เห็นด้วย	เห็นด้วย	เห็นด้วย	เห็นด้วย	24.00	เห็นด้วย	เห็นด้วย
เห็นด้วย	เห็นด้วย	เห็นด้วย	เห็นด้วย	เห็นด้วย	เห็นด้วย	17.00	เห็นด้วย	เห็นด้วย
เห็นด้วย	เห็นด้วย	เห็นด้วย	เห็นด้วย	เห็นด้วย	เห็นด้วย	18.00	เห็นด้วย	เห็นด้วย
เห็นด้วยอย่	เห็นด้วย	เห็นด้วย	เห็นด้วย	เห็นด้วยอย่	เห็นด้วยอย่	23.00	เห็นด้วยอย่	เห็นด้วยอย่
เห็นด้วยอย่	เห็นด้วยอย่	เห็นด้วยอย่	เห็นด้วยอย่	เห็นด้วยอย่	เห็นด้วยอย่	21.00	เห็นด้วยอย่	เห็นด้วยอย่
เห็นด้วย	เห็นด้วย	เห็นด้วย	เห็นด้วย	เห็นด้วย	เห็นด้วย	24.00	เห็นด้วย	เห็นด้วย
เห็นด้วยอย่	เห็นด้วย	เห็นด้วย	เห็นด้วย	เห็นด้วย	เห็นด้วยอย่	21.00	เห็นด้วย	เห็นด้วย
เห็นด้วย	เห็นด้วยอย่	เห็นด้วย	เห็นด้วย	เห็นด้วย	ไม่เห็นด้วย	19.00	เห็นด้วยอย่	เห็นด้วยอย่
เห็นด้วย	เห็นด้วย	เห็นด้วย	เห็นด้วย	เห็นด้วย	เห็นด้วย	22.00	เห็นด้วย	เห็นด้วย
เห็นด้วยอย่	เห็นด้วยอย่	เห็นด้วยอย่	เห็นด้วยอย่	เห็นด้วยอย่	เห็นด้วยอย่	16.00	เห็นด้วยอย่	เห็นด้วยอย่
เห็นด้วย	เห็นด้วย	เห็นด้วย	เห็นด้วย	เห็นด้วย	เห็นด้วย	16.00	เห็นด้วย	เห็นด้วย
เห็นด้วย	เห็นด้วยอย่	เห็นด้วย	เห็นด้วยอย่	เห็นด้วยอย่	เห็นด้วย	18.00	เห็นด้วย	เห็นด้วย
เห็นด้วย	เห็นด้วย	เห็นด้วย	เห็นด้วย	เห็นด้วย	เห็นด้วย	17.00	เห็นด้วยอย่	เห็นด้วย
เห็นด้วย	เห็นด้วย	ไม่เห็นด้วย	เห็นด้วย	เห็นด้วย	เห็นด้วย	17.00	เห็นด้วย	เห็นด้วยอย่
เห็นด้วย	เห็นด้วย	เห็นด้วย	เห็นด้วย	เห็นด้วย	เห็นด้วย	22.00	เห็นด้วย	เห็นด้วย
เห็นด้วยอย่	เห็นด้วยอย่	เห็นด้วยอย่	เห็นด้วยอย่	เห็นด้วยอย่	เห็นด้วย	18.00	เห็นด้วยอย่	เห็นด้วย
เห็นด้วย	เห็นด้วย	เห็นด้วย	เห็นด้วย	เห็นด้วย	ไม่เห็นด้วย	17.00	เห็นด้วย	เห็นด้วย
เห็นด้วยอย่	เห็นด้วยอย่	เห็นด้วยอย่	เห็นด้วยอย่	เห็นด้วยอย่	เห็นด้วยอย่	20.00	เห็นด้วยอย่	เห็นด้วยอย่
เห็นด้วยอย่	เห็นด้วยอย่	เห็นด้วยอย่	เห็นด้วยอย่	เห็นด้วยอย่	เห็นด้วยอย่	17.00	เห็นด้วย	เห็นด้วย

เห็นด้วย	เห็นด้วย	เห็นด้วย	เห็นด้วย	เห็นด้วย	เห็นด้วย	18.00	เห็นด้วย	เห็นด้วย
เห็นด้วย	เห็นด้วย	ไม่เห็นด้วย	เห็นด้วย	เห็นด้วย	เห็นด้วย	22.00	เห็นด้วย	เห็นด้วย
เห็นด้วยอย่	เห็นด้วย	เห็นด้วยอย่	เห็นด้วยอย่	เห็นด้วย	เห็นด้วย	22.00	เห็นด้วยอย่	เห็นด้วย
เห็นด้วยอย่	เห็นด้วยอย่	เห็นด้วยอย่	เห็นด้วยอย่	เห็นด้วยอย่	เห็นด้วยอย่	21.00	เห็นด้วยอย่	เห็นด้วยอย่
เห็นด้วยอย่	เห็นด้วยอย่	เห็นด้วยอย่	เห็นด้วย	เห็นด้วย	เห็นด้วย	23.00	ไม่เห็นด้วย	เห็นด้วย
เห็นด้วยอย่	เห็นด้วยอย่	ไม่เห็นด้วย	เห็นด้วยอย่	เห็นด้วยอย่	เห็นด้วยอย่	21.00	เห็นด้วยอย่	เห็นด้วยอย่
เห็นด้วย	เห็นด้วย	เห็นด้วย	เห็นด้วย	เห็นด้วย	เห็นด้วย	21.00	เห็นด้วย	ไม่เห็นด้วย
เห็นด้วย	เห็นด้วย	เห็นด้วย	เห็นด้วย	เห็นด้วย	เห็นด้วย	23.00	เห็นด้วย	เห็นด้วย
เห็นด้วยอย่	เห็นด้วยอย่	เห็นด้วย	เห็นด้วยอย่	เห็นด้วยอย่	เห็นด้วย	18.00	เห็นด้วยอย่	เห็นด้วยอย่
เห็นด้วย	เห็นด้วยอย่	ไม่เห็นด้วย	เห็นด้วยอย่	เห็นด้วยอย่	ไม่เห็นด้วย	18.00	เห็นด้วยอย่	เห็นด้วยอย่
เห็นด้วย	เห็นด้วย	เห็นด้วย	เห็นด้วย	ไม่เห็นด้วย	ไม่เห็นด้วย	17.00	เห็นด้วย	ไม่เห็นด้วย
เห็นด้วย	เห็นด้วยอย่	ไม่เห็นด้วย	เห็นด้วยอย่	เห็นด้วยอย่	ไม่เห็นด้วย	18.00	เห็นด้วยอย่	เห็นด้วยอย่
เห็นด้วย	เห็นด้วย	ไม่เห็นด้วย	เห็นด้วย	เห็นด้วย	เห็นด้วย	18.00	เห็นด้วยอย่	เห็นด้วยอย่
เห็นด้วยอย่	เห็นด้วยอย่	เห็นด้วยอย่	เห็นด้วยอย่	เห็นด้วยอย่	ไม่เห็นด้วย	20.00	เห็นด้วยอย่	เห็นด้วยอย่
เห็นด้วยอย่	เห็นด้วยอย่	เห็นด้วยอย่	เห็นด้วยอย่	เห็นด้วยอย่	ไม่เห็นด้วย	17.00	เห็นด้วยอย่	เห็นด้วย
เห็นด้วย	เห็นด้วย	เห็นด้วย	เห็นด้วย	เห็นด้วย	เห็นด้วย	21.00	เห็นด้วย	เห็นด้วย
เห็นด้วยอย่	เห็นด้วยอย่	เห็นด้วย	เห็นด้วยอย่	เห็นด้วยอย่	เห็นด้วยอย่	18.00	เห็นด้วยอย่	เห็นด้วย
เห็นด้วย	เห็นด้วย	เห็นด้วย	เห็นด้วย	เห็นด้วย	เห็นด้วย	24.00	เห็นด้วย	เห็นด้วย
เห็นด้วย	เห็นด้วย	เห็นด้วย	เห็นด้วย	เห็นด้วย	เห็นด้วย	18.00	เห็นด้วย	ไม่เห็นด้วย
เห็นด้วยอย่	เห็นด้วยอย่	เห็นด้วยอย่	เห็นด้วย	เห็นด้วย	เห็นด้วย	13.00	เห็นด้วยอย่	เห็นด้วยอย่
เห็นด้วยอย่	เห็นด้วยอย่	เห็นด้วยอย่	เห็นด้วยอย่	เห็นด้วยอย่	เห็นด้วย	22.00	เห็นด้วย	เห็นด้วย
เห็นด้วย	เห็นด้วย	เห็นด้วย	เห็นด้วย	เห็นด้วย	เห็นด้วย	23.00	เห็นด้วย	เห็นด้วย
เห็นด้วย	เห็นด้วย	เห็นด้วย	เห็นด้วย	เห็นด้วย	เห็นด้วย	18.00	เห็นด้วย	เห็นด้วย
ไม่เห็นด้วย	เห็นด้วย	เห็นด้วย	เห็นด้วย	เห็นด้วย	เห็นด้วย	24.00	เห็นด้วย	เห็นด้วย
ไม่เห็นด้วย	เห็นด้วย	เห็นด้วย	เห็นด้วย	เห็นด้วย	เห็นด้วยอย่	22.00	เห็นด้วย	ไม่เห็นด้วย
เห็นด้วย	เห็นด้วย	เห็นด้วย	เห็นด้วยอย่	เห็นด้วย	เห็นด้วย	20.00	เห็นด้วย	เห็นด้วย
เห็นด้วยอย่	เห็นด้วย	เห็นด้วย	เห็นด้วยอย่	เห็นด้วย	เห็นด้วย	18.00	ไม่เห็นด้วย	เห็นด้วย
เห็นด้วยอย่	เห็นด้วยอย่	เห็นด้วยอย่	เห็นด้วยอย่	เห็นด้วย	เห็นด้วย	17.00	เห็นด้วยอย่	เห็นด้วย
เห็นด้วย	เห็นด้วย	เห็นด้วย	เห็นด้วย	เห็นด้วย	เห็นด้วย	18.00	เห็นด้วย	ไม่เห็นด้วย
เห็นด้วย	ไม่เห็นด้วย	ไม่เห็นด้วย	เห็นด้วย	เห็นด้วย	เห็นด้วย	22.00	เห็นด้วย	เห็นด้วย
เห็นด้วย	เห็นด้วย	เห็นด้วย	เห็นด้วย	เห็นด้วย	ไม่เห็นด้วย	24.00	เห็นด้วย	เห็นด้วย
เห็นด้วยอย่	เห็นด้วยอย่	เห็นด้วย	เห็นด้วย	เห็นด้วย	เห็นด้วย	18.00	เห็นด้วย	เห็นด้วย
เห็นด้วยอย่	เห็นด้วยอย่	เห็นด้วยอย่	เห็นด้วยอย่	เห็นด้วยอย่	เห็นด้วยอย่	19.00	เห็นด้วยอย่	เห็นด้วยอย่
เห็นด้วย	เห็นด้วย	เห็นด้วย	เห็นด้วย	เห็นด้วย	ไม่เห็นด้วย	18.00	เห็นด้วย	เห็นด้วย
เห็นด้วย	ไม่เห็นด้วย	ไม่เห็นด้วย	เห็นด้วย	ไม่เห็นด้วย	ไม่เห็นด้วย	22.00	เห็นด้วย	ไม่เห็นด้วย
เห็นด้วยอย่	เห็นด้วยอย่	เห็นด้วยอย่	เห็นด้วย	เห็นด้วย	เห็นด้วย	18.00	เห็นด้วยอย่	เห็นด้วย
เห็นด้วย	เห็นด้วยอย่	เห็นด้วยอย่	เห็นด้วยอย่	เห็นด้วยอย่	เห็นด้วยอย่	18.00	เห็นด้วย	เห็นด้วยอย่
เห็นด้วย	เห็นด้วย	ไม่เห็นด้วย	เห็นด้วย	เห็นด้วย	เห็นด้วยอย่	19.00	เห็นด้วย	เห็นด้วยอย่
เห็นด้วย	เห็นด้วย	เห็นด้วย	เห็นด้วย	เห็นด้วย	เห็นด้วย	17.00	เห็นด้วย	เห็นด้วย
เห็นด้วย	เห็นด้วย	เห็นด้วย	เห็นด้วย	เห็นด้วยอย่	เห็นด้วย	23.00	เห็นด้วยอย่	เห็นด้วยอย่
เห็นด้วย	เห็นด้วย	ไม่เห็นด้วย	เห็นด้วย	เห็นด้วย	ไม่เห็นด้วย	18.00	เห็นด้วย	ไม่เห็นด้วย
เห็นด้วย	เห็นด้วย	เห็นด้วย	เห็นด้วย	เห็นด้วย	เห็นด้วย	22.00	เห็นด้วย	เห็นด้วย
เห็นด้วย	เห็นด้วย	เห็นด้วย	เห็นด้วย	เห็นด้วย	ไม่เห็นด้วย	17.00	เห็นด้วย	เห็นด้วย
ไม่เห็นด้วย	เห็นด้วย	ไม่เห็นด้วย	เห็นด้วยอย่	เห็นด้วย	เห็นด้วย	20.00	เห็นด้วยอย่	เห็นด้วย
เห็นด้วยอย่	เห็นด้วยอย่	เห็นด้วยอย่	เห็นด้วยอย่	เห็นด้วยอย่	เห็นด้วยอย่	20.00	เห็นด้วยอย่	เห็นด้วยอย่
เห็นด้วย	เห็นด้วย	เห็นด้วย	เห็นด้วย	ไม่เห็นด้วย	เห็นด้วย	24.00	เห็นด้วย	ไม่เห็นด้วย
เห็นด้วย	เห็นด้วย	เห็นด้วย	เห็นด้วย	เห็นด้วย	เห็นด้วย	18.00	เห็นด้วย	เห็นด้วย
เห็นด้วยอย่	เห็นด้วยอย่	เห็นด้วย	เห็นด้วย	เห็นด้วย	เห็นด้วย	21.00	เห็นด้วยอย่	เห็นด้วย
เห็นด้วย	เห็นด้วย	เห็นด้วย	เห็นด้วย	เห็นด้วย	ไม่เห็นด้วย	20.00	เห็นด้วย	เห็นด้วย
เห็นด้วย	เห็นด้วยอย่	เห็นด้วยอย่	เห็นด้วยอย่	เห็นด้วย	เห็นด้วย	17.00	เห็นด้วยอย่	เห็นด้วยอย่
เห็นด้วยอย่	เห็นด้วยอย่	เห็นด้วย	เห็นด้วยอย่	เห็นด้วย	เห็นด้วย	18.00	เห็นด้วย	เห็นด้วย

เห็นด้วยอย่	เห็นด้วยอย่	เห็นด้วยอย่	เห็นด้วยอย่	เห็นด้วยอย่	เห็นด้วยอย่	17.00	เห็นด้วย	เห็นด้วย
เห็นด้วย	เห็นด้วย	เห็นด้วย	เห็นด้วย	เห็นด้วย	เห็นด้วย	24.00	เห็นด้วยอย่	เห็นด้วยอย่
เห็นด้วย	เห็นด้วย	เห็นด้วย	เห็นด้วย	เห็นด้วย	เห็นด้วยอย่	22.00	เห็นด้วย	เห็นด้วย
เห็นด้วย	เห็นด้วย	เห็นด้วย	เห็นด้วย	เห็นด้วย	เห็นด้วย	17.00	เห็นด้วย	เห็นด้วย
เห็นด้วย	เห็นด้วย	เห็นด้วย	เห็นด้วย	เห็นด้วย	เห็นด้วย	23.00	เห็นด้วย	เห็นด้วย
เห็นด้วย	เห็นด้วย	เห็นด้วย	เห็นด้วย	เห็นด้วย	เห็นด้วย	22.00	เห็นด้วย	เห็นด้วย
เห็นด้วย	เห็นด้วย	เห็นด้วย	ไม่เห็นด้วย	ไม่เห็นด้วย	เห็นด้วย	17.00	เห็นด้วย	เห็นด้วย
เห็นด้วยอย่	เห็นด้วยอย่	เห็นด้วยอย่	เห็นด้วยอย่	เห็นด้วยอย่	เห็นด้วยอย่	18.00	เห็นด้วย	เห็นด้วย
เห็นด้วยอย่	เห็นด้วย	เห็นด้วยอย่	เห็นด้วย	เห็นด้วย	เห็นด้วย	14.00	เห็นด้วย	เห็นด้วย
เห็นด้วยอย่	เห็นด้วยอย่	เห็นด้วย	เห็นด้วย	เห็นด้วย	ไม่เห็นด้วย	23.00	เห็นด้วยอย่	เห็นด้วยอย่
เห็นด้วย	เห็นด้วย	เห็นด้วย	เห็นด้วย	เห็นด้วย	เห็นด้วย	18.00	เห็นด้วย	เห็นด้วย
เห็นด้วยอย่	เห็นด้วยอย่	เห็นด้วย	เห็นด้วย	เห็นด้วย	ไม่เห็นด้วย	23.00	เห็นด้วย	เห็นด้วย
เห็นด้วย	เห็นด้วย	เห็นด้วย	เห็นด้วย	เห็นด้วย	เห็นด้วย	18.00	เห็นด้วย	เห็นด้วย
เห็นด้วย	เห็นด้วย	เห็นด้วย	เห็นด้วย	เห็นด้วย	เห็นด้วย	17.00	เห็นด้วยอย่	เห็นด้วยอย่
เห็นด้วยอย่	เห็นด้วยอย่	เห็นด้วยอย่	เห็นด้วย	เห็นด้วย	เห็นด้วย	18.00	เห็นด้วยอย่	เห็นด้วยอย่
เห็นด้วย	เห็นด้วยอย่	เห็นด้วยอย่	เห็นด้วยอย่	เห็นด้วย	เห็นด้วย	17.00	เห็นด้วยอย่	เห็นด้วย
เห็นด้วย	เห็นด้วย	เห็นด้วย	เห็นด้วย	เห็นด้วย	เห็นด้วย	24.00	เห็นด้วย	เห็นด้วย
เห็นด้วย	เห็นด้วย	เห็นด้วย	เห็นด้วย	เห็นด้วย	เห็นด้วย	23.00	เห็นด้วย	เห็นด้วย
เห็นด้วยอย่	เห็นด้วยอย่	เห็นด้วย	เห็นด้วยอย่	เห็นด้วยอย่	เห็นด้วย	18.00	เห็นด้วยอย่	เห็นด้วยอย่
เห็นด้วย	เห็นด้วย	เห็นด้วย	เห็นด้วยอย่	เห็นด้วย	เห็นด้วย	16.00	เห็นด้วย	เห็นด้วย
เห็นด้วยอย่	เห็นด้วยอย่	เห็นด้วย	เห็นด้วยอย่	เห็นด้วยอย่	เห็นด้วย	18.00	เห็นด้วย	เห็นด้วยอย่
เห็นด้วย	เห็นด้วย	เห็นด้วย	เห็นด้วย	เห็นด้วย	ไม่เห็นด้วย	21.00	เห็นด้วยอย่	เห็นด้วย
เห็นด้วย	เห็นด้วย	ไม่เห็นด้วย	เห็นด้วย	เห็นด้วย	เห็นด้วย	21.00	เห็นด้วยอย่	เห็นด้วย
เห็นด้วยอย่	เห็นด้วยอย่	ไม่เห็นด้วย	เห็นด้วยอย่	เห็นด้วยอย่	เห็นด้วย	17.00	เห็นด้วย	เห็นด้วย
เห็นด้วย	เห็นด้วย	เห็นด้วย	เห็นด้วย	เห็นด้วย	เห็นด้วย	23.00	เห็นด้วย	เห็นด้วย
เห็นด้วย	เห็นด้วย	เห็นด้วย	เห็นด้วย	เห็นด้วย	เห็นด้วย	24.00	เห็นด้วย	เห็นด้วย
เห็นด้วย	เห็นด้วย	เห็นด้วย	เห็นด้วย	เห็นด้วย	เห็นด้วย	18.00	เห็นด้วย	เห็นด้วย
เห็นด้วย	เห็นด้วย	เห็นด้วย	เห็นด้วย	เห็นด้วย	เห็นด้วย	21.00	เห็นด้วย	เห็นด้วย
เห็นด้วย	เห็นด้วย	เห็นด้วย	เห็นด้วย	เห็นด้วย	เห็นด้วย	18.00	เห็นด้วย	ไม่เห็นด้วย
เห็นด้วยอย่	เห็นด้วยอย่	เห็นด้วย	เห็นด้วยอย่	เห็นด้วยอย่	เห็นด้วย	19.00	เห็นด้วยอย่	เห็นด้วยอย่
เห็นด้วยอย่	เห็นด้วยอย่	เห็นด้วย	เห็นด้วยอย่	เห็นด้วยอย่	ไม่เห็นด้วย	22.00	เห็นด้วย	เห็นด้วยอย่
เห็นด้วยอย่	เห็นด้วยอย่	เห็นด้วยอย่	เห็นด้วยอย่	เห็นด้วยอย่	เห็นด้วยอย่	19.00	เห็นด้วยอย่	เห็นด้วยอย่
เห็นด้วยอย่	เห็นด้วยอย่	เห็นด้วย	เห็นด้วยอย่	เห็นด้วยอย่	เห็นด้วย	18.00	เห็นด้วยอย่	เห็นด้วย
เห็นด้วย	เห็นด้วย	เห็นด้วย	เห็นด้วย	เห็นด้วย	เห็นด้วย	22.00	เห็นด้วย	เห็นด้วย
เห็นด้วยอย่	เห็นด้วยอย่	เห็นด้วย	เห็นด้วยอย่	เห็นด้วย	เห็นด้วยอย่	24.00	ไม่เห็นด้วย	ไม่เห็นด้วย
เห็นด้วย	เห็นด้วย	เห็นด้วย	เห็นด้วย	เห็นด้วย	ไม่เห็นด้วย	18.00	เห็นด้วย	เห็นด้วย
เห็นด้วย	เห็นด้วย	เห็นด้วย	เห็นด้วย	เห็นด้วย	เห็นด้วย	18.00	เห็นด้วย	เห็นด้วย
เห็นด้วยอย่	เห็นด้วยอย่	เห็นด้วยอย่	เห็นด้วยอย่	เห็นด้วยอย่	ไม่เห็นด้วย	23.00	เห็นด้วยอย่	เห็นด้วย
เห็นด้วยอย่	เห็นด้วยอย่	เห็นด้วยอย่	เห็นด้วยอย่	เห็นด้วยอย่	เห็นด้วย	18.00	เห็นด้วย	เห็นด้วยอย่
เห็นด้วยอย่	เห็นด้วยอย่	เห็นด้วยอย่	เห็นด้วยอย่	เห็นด้วยอย่	เห็นด้วยอย่	21.00	เห็นด้วยอย่	เห็นด้วย
เห็นด้วย	เห็นด้วย	เห็นด้วย	เห็นด้วย	เห็นด้วย	เห็นด้วย	21.00	เห็นด้วย	เห็นด้วย
เห็นด้วย	เห็นด้วย	เห็นด้วย	เห็นด้วย	เห็นด้วย	เห็นด้วย	22.00	เห็นด้วย	เห็นด้วย
เห็นด้วยอย่	เห็นด้วยอย่	เห็นด้วยอย่	เห็นด้วยอย่	เห็นด้วยอย่	ไม่เห็นด้วย	19.00	เห็นด้วย	เห็นด้วยอย่
เห็นด้วย	เห็นด้วย	เห็นด้วย	เห็นด้วย	เห็นด้วย	เห็นด้วย	24.00	เห็นด้วย	เห็นด้วย
ไม่เห็นด้วย	เห็นด้วย	เห็นด้วยอย่	เห็นด้วยอย่	เห็นด้วยอย่	เห็นด้วยอย่	18.00	เห็นด้วยอย่	เห็นด้วยอย่
เห็นด้วย	เห็นด้วย	เห็นด้วย	เห็นด้วย	เห็นด้วย	เห็นด้วย	19.00	เห็นด้วย	เห็นด้วย
เห็นด้วยอย่	เห็นด้วยอย่	เห็นด้วยอย่	เห็นด้วยอย่	เห็นด้วยอย่	เห็นด้วย	17.00	เห็นด้วย	เห็นด้วย
เห็นด้วยอย่	เห็นด้วยอย่	เห็นด้วย	เห็นด้วย	เห็นด้วยอย่	เห็นด้วย	17.00	เห็นด้วย	เห็นด้วย
เห็นด้วย	เห็นด้วย	เห็นด้วย	เห็นด้วย	เห็นด้วย	เห็นด้วย	18.00	เห็นด้วยอย่	เห็นด้วยอย่
เห็นด้วย	เห็นด้วย	เห็นด้วย	เห็นด้วย	เห็นด้วย	เห็นด้วย	16.00	เห็นด้วย	เห็นด้วย
เห็นด้วย	เห็นด้วย	เห็นด้วย	เห็นด้วย	เห็นด้วย	เห็นด้วย	17.00	เห็นด้วยอย่	เห็นด้วยอย่

เห็นด้วย	เห็นด้วย	เห็นด้วย	เห็นด้วย	เห็นด้วย	เห็นด้วย	22.00	เห็นด้วย	เห็นด้วย
เห็นด้วยอย่	เห็นด้วยอย่	เห็นด้วยอย่	เห็นด้วยอย่	เห็นด้วยอย่	เห็นด้วยอย่	17.00	เห็นด้วยอย่	เห็นด้วยอย่
เห็นด้วยอย่	เห็นด้วยอย่	เห็นด้วยอย่	เห็นด้วยอย่	เห็นด้วย	เห็นด้วยอย่	19.00	เห็นด้วยอย่	เห็นด้วยอย่
เห็นด้วยอย่	เห็นด้วยอย่	เห็นด้วย	เห็นด้วย	เห็นด้วย	เห็นด้วย	16.00	เห็นด้วย	เห็นด้วย
เห็นด้วยอย่	เห็นด้วย	เห็นด้วย	เห็นด้วย	เห็นด้วย	เห็นด้วย	18.00	เห็นด้วย	เห็นด้วย
เห็นด้วยอย่	เห็นด้วยอย่	เห็นด้วยอย่	เห็นด้วยอย่	เห็นด้วยอย่	เห็นด้วยอย่	17.00	เห็นด้วย	เห็นด้วย
เห็นด้วย	เห็นด้วย	เห็นด้วย	เห็นด้วย	เห็นด้วย	เห็นด้วย	19.00	เห็นด้วย	เห็นด้วย
เห็นด้วยอย่	เห็นด้วยอย่	เห็นด้วยอย่	เห็นด้วยอย่	เห็นด้วยอย่	เห็นด้วย	19.00	เห็นด้วยอย่	เห็นด้วยอย่
เห็นด้วย	เห็นด้วย	เห็นด้วย	เห็นด้วย	เห็นด้วย	เห็นด้วย	24.00	เห็นด้วย	เห็นด้วย
เห็นด้วย	เห็นด้วยอย่	เห็นด้วยอย่	เห็นด้วยอย่	เห็นด้วยอย่	เห็นด้วยอย่	18.00	เห็นด้วยอย่	เห็นด้วยอย่
เห็นด้วย	เห็นด้วย	เห็นด้วย	เห็นด้วย	เห็นด้วย	เห็นด้วย	18.00	เห็นด้วย	เห็นด้วย
เห็นด้วยอย่	เห็นด้วยอย่	เห็นด้วย	เห็นด้วยอย่	เห็นด้วยอย่	เห็นด้วยอย่	21.00	เห็นด้วยอย่	เห็นด้วย
เห็นด้วยอย่	เห็นด้วยอย่	เห็นด้วย	เห็นด้วย	เห็นด้วยอย่	เห็นด้วย	21.00	เห็นด้วยอย่	เห็นด้วยอย่
เห็นด้วย	เห็นด้วย	เห็นด้วย	เห็นด้วยอย่	เห็นด้วย	เห็นด้วย	17.00	เห็นด้วย	เห็นด้วย
ไม่เห็นด้วย	เห็นด้วย	เห็นด้วย	เห็นด้วย	ไม่เห็นด้วย	เห็นด้วย	17.00	เห็นด้วย	เห็นด้วย
เห็นด้วย	ไม่เห็นด้วย	เห็นด้วย	เห็นด้วย	เห็นด้วย	ไม่เห็นด้วย	21.00	เห็นด้วย	เห็นด้วย
เห็นด้วย	เห็นด้วย	เห็นด้วย	เห็นด้วย	เห็นด้วย	เห็นด้วยอย่	18.00	เห็นด้วยอย่	เห็นด้วยอย่
เห็นด้วย	ไม่เห็นด้วย	เห็นด้วย	เห็นด้วย	เห็นด้วย	เห็นด้วยอย่	19.00	เห็นด้วย	เห็นด้วย
เห็นด้วยอย่	เห็นด้วย	เห็นด้วยอย่	เห็นด้วยอย่	เห็นด้วย	เห็นด้วยอย่	22.00	เห็นด้วย	เห็นด้วย
เห็นด้วยอย่	เห็นด้วยอย่	ไม่เห็นด้วย	ไม่เห็นด้วย	เห็นด้วย	ไม่เห็นด้วย	18.00	เห็นด้วยอย่	เห็นด้วยอย่
เห็นด้วย	เห็นด้วยอย่	เห็นด้วยอย่	เห็นด้วยอย่	เห็นด้วยอย่	เห็นด้วยอย่	18.00	เห็นด้วยอย่	เห็นด้วยอย่
เห็นด้วยอย่	เห็นด้วยอย่	เห็นด้วย	เห็นด้วย	ไม่เห็นด้วย	ไม่เห็นด้วย	18.00	เห็นด้วยอย่	เห็นด้วยอย่
เห็นด้วย	เห็นด้วย	ไม่เห็นด้วย	เห็นด้วย	เห็นด้วย	ไม่เห็นด้วย	18.00	เห็นด้วย	เห็นด้วย
เห็นด้วยอย่	เห็นด้วยอย่	เห็นด้วย	เห็นด้วยอย่	เห็นด้วยอย่	เห็นด้วย	18.00	เห็นด้วย	เห็นด้วย
เห็นด้วยอย่	เห็นด้วยอย่	เห็นด้วย	เห็นด้วยอย่	เห็นด้วยอย่	ไม่เห็นด้วย	17.00	เห็นด้วย	เห็นด้วย
เห็นด้วย	เห็นด้วย	เห็นด้วย	เห็นด้วย	เห็นด้วย	เห็นด้วย	18.00	เห็นด้วย	เห็นด้วย
เห็นด้วย	เห็นด้วยอย่	เห็นด้วยอย่	เห็นด้วยอย่	เห็นด้วยอย่	เห็นด้วยอย่	18.00	เห็นด้วยอย่	เห็นด้วยอย่
เห็นด้วย	เห็นด้วย	เห็นด้วย	เห็นด้วย	เห็นด้วย	เห็นด้วย	23.00	เห็นด้วย	เห็นด้วย
เห็นด้วยอย่	เห็นด้วยอย่	เห็นด้วย	เห็นด้วยอย่	เห็นด้วยอย่	เห็นด้วย	23.00	เห็นด้วย	เห็นด้วยอย่
เห็นด้วยอย่	เห็นด้วยอย่	เห็นด้วย	เห็นด้วยอย่	เห็นด้วยอย่	เห็นด้วย	19.00	เห็นด้วยอย่	เห็นด้วยอย่
เห็นด้วย	เห็นด้วย	เห็นด้วย	ไม่เห็นด้วย	เห็นด้วย	เห็นด้วย	14.00	เห็นด้วย	เห็นด้วย
เห็นด้วยอย่	เห็นด้วยอย่	เห็นด้วย	เห็นด้วยอย่	เห็นด้วยอย่	เห็นด้วยอย่	18.00	เห็นด้วยอย่	เห็นด้วยอย่
เห็นด้วย	เห็นด้วยอย่	เห็นด้วย	เห็นด้วยอย่	เห็นด้วย	เห็นด้วย	14.00	เห็นด้วยอย่	เห็นด้วยอย่
เห็นด้วย	เห็นด้วย	เห็นด้วย	เห็นด้วย	เห็นด้วย	ไม่เห็นด้วย	18.00	เห็นด้วย	เห็นด้วย
เห็นด้วยอย่	เห็นด้วยอย่	เห็นด้วย	เห็นด้วยอย่	เห็นด้วยอย่	เห็นด้วย	18.00	เห็นด้วยอย่	เห็นด้วยอย่
เห็นด้วย	เห็นด้วยอย่	เห็นด้วย	เห็นด้วย	ไม่เห็นด้วย	ไม่เห็นด้วย	18.00	เห็นด้วยอย่	เห็นด้วยอย่
เห็นด้วย	เห็นด้วย	ไม่เห็นด้วย	เห็นด้วยอย่	เห็นด้วย	ไม่เห็นด้วย	24.00	เห็นด้วย	เห็นด้วยอย่
เห็นด้วย	เห็นด้วยอย่	เห็นด้วย	เห็นด้วย	เห็นด้วย	เห็นด้วย	21.00	เห็นด้วยอย่	เห็นด้วยอย่
เห็นด้วยอย่	เห็นด้วย	เห็นด้วย	เห็นด้วยอย่	เห็นด้วยอย่	เห็นด้วยอย่	16.00	เห็นด้วย	เห็นด้วยอย่
เห็นด้วย	เห็นด้วยอย่	เห็นด้วย	เห็นด้วยอย่	เห็นด้วย	เห็นด้วยอย่	24.00	เห็นด้วยอย่	เห็นด้วยอย่
เห็นด้วย	เห็นด้วยอย่	เห็นด้วย	เห็นด้วย	เห็นด้วย	เห็นด้วยอย่	22.00	เห็นด้วย	เห็นด้วย
เห็นด้วย	ไม่เห็นด้วย	เห็นด้วย	ไม่เห็นด้วย	เห็นด้วย	ไม่เห็นด้วย	24.00	ไม่เห็นด้วย	เห็นด้วย
ไม่เห็นด้วย	เห็นด้วย	ไม่เห็นด้วย	เห็นด้วย	ไม่เห็นด้วย	ไม่เห็นด้วย	21.00	เห็นด้วยอย่	เห็นด้วยอย่
ไม่เห็นด้วย	เห็นด้วย	เห็นด้วย	เห็นด้วย	ไม่เห็นด้วย	ไม่เห็นด้วย	19.00	เห็นด้วยอย่	เห็นด้วยอย่
เห็นด้วย	เห็นด้วย	เห็นด้วย	เห็นด้วย	เห็นด้วย	ไม่เห็นด้วย	16.00	เห็นด้วย	เห็นด้วยอย่
เห็นด้วยอย่	เห็นด้วยอย่	เห็นด้วยอย่	เห็นด้วยอย่	เห็นด้วย	ไม่เห็นด้วย	17.00	เห็นด้วยอย่	เห็นด้วยอย่
เห็นด้วยอย่	เห็นด้วยอย่	เห็นด้วย	เห็นด้วยอย่	เห็นด้วย	เห็นด้วยอย่	17.00	เห็นด้วย	เห็นด้วย
เห็นด้วยอย่	เห็นด้วย	เห็นด้วยอย่	เห็นด้วยอย่	เห็นด้วยอย่	เห็นด้วยอย่	21.00	เห็นด้วยอย่	เห็นด้วยอย่
เห็นด้วยอย่	เห็นด้วยอย่	เห็นด้วยอย่	เห็นด้วยอย่	ไม่เห็นด้วย	เห็นด้วย	18.00	เห็นด้วยอย่	เห็นด้วยอย่
เห็นด้วย	เห็นด้วย	เห็นด้วยอย่	เห็นด้วย	เห็นด้วยอย่	เห็นด้วย	24.00	เห็นด้วยอย่	เห็นด้วยอย่
เห็นด้วย	เห็นด้วยอย่	เห็นด้วยอย่	เห็นด้วยอย่	เห็นด้วยอย่	เห็นด้วย	16.00	เห็นด้วยอย่	เห็นด้วยอย่

เห็นด้วย	เห็นด้วย	เห็นด้วย	ไม่เห็นด้วย	เห็นด้วย	ไม่เห็นด้วย	15.00	ไม่เห็นด้วย	เห็นด้วย
เห็นด้วย	ไม่เห็นด้วย	เห็นด้วย	เห็นด้วย	เห็นด้วย	ไม่เห็นด้วย	17.00	เห็นด้วย	เห็นด้วย
เห็นด้วย	เห็นด้วย	เห็นด้วย	เห็นด้วย	เห็นด้วย	ไม่เห็นด้วย	18.00	เห็นด้วย	เห็นด้วย
เห็นด้วย	เห็นด้วย	เห็นด้วย	เห็นด้วย	เห็นด้วย	ไม่เห็นด้วย	18.00	ไม่เห็นด้วย	เห็นด้วย
ไม่เห็นด้วย	เห็นด้วยอย่	เห็นด้วยอย่	เห็นด้วยอย่	เห็นด้วยอย่	เห็นด้วยอย่	19.00	เห็นด้วยอย่	เห็นด้วยอย่
เห็นด้วยอย่	เห็นด้วยอย่	เห็นด้วยอย่	เห็นด้วยอย่	เห็นด้วยอย่	เห็นด้วยอย่	21.00	เห็นด้วยอย่	เห็นด้วยอย่
เห็นด้วย	เห็นด้วย	เห็นด้วย	เห็นด้วยอย่	เห็นด้วย	เห็นด้วย	16.00	เห็นด้วย	เห็นด้วย
เห็นด้วยอย่	เห็นด้วยอย่	เห็นด้วยอย่	เห็นด้วยอย่	เห็นด้วยอย่	เห็นด้วย	19.00	เห็นด้วยอย่	เห็นด้วยอย่
เห็นด้วยอย่	เห็นด้วยอย่	เห็นด้วยอย่	เห็นด้วย	เห็นด้วยอย่	เห็นด้วย	18.00	เห็นด้วย	เห็นด้วย
ไม่เห็นด้วย	เห็นด้วย	เห็นด้วย	เห็นด้วย	เห็นด้วย	ไม่เห็นด้วย	23.00	เห็นด้วย	เห็นด้วย
ไม่เห็นด้วย	ไม่เห็นด้วย	ไม่เห็นด้วย	เห็นด้วย	เห็นด้วย	ไม่เห็นด้วย	18.00	เห็นด้วย	เห็นด้วย
เห็นด้วยอย่	เห็นด้วยอย่	ไม่เห็นด้วย	เห็นด้วย	เห็นด้วย	ไม่เห็นด้วย	18.00	เห็นด้วยอย่	เห็นด้วย
เห็นด้วย	เห็นด้วย	เห็นด้วย	เห็นด้วย	เห็นด้วยอย่	ไม่เห็นด้วย	22.00	ไม่เห็นด้วย	ไม่เห็นด้วย
เห็นด้วย	เห็นด้วย	เห็นด้วย	เห็นด้วย	เห็นด้วย	เห็นด้วย	23.00	เห็นด้วย	เห็นด้วย
เห็นด้วยอย่	เห็นด้วยอย่	เห็นด้วย	เห็นด้วยอย่	เห็นด้วยอย่	ไม่เห็นด้วย	24.00	เห็นด้วย	เห็นด้วย
เห็นด้วย	เห็นด้วย	เห็นด้วย	เห็นด้วย	เห็นด้วย	ไม่เห็นด้วย	24.00	เห็นด้วย	เห็นด้วย
เห็นด้วยอย่	เห็นด้วยอย่	เห็นด้วย	เห็นด้วย	เห็นด้วย	ไม่เห็นด้วย	24.00	เห็นด้วย	เห็นด้วยอย่
เห็นด้วย	เห็นด้วย	เห็นด้วย	เห็นด้วย	เห็นด้วย	ไม่เห็นด้วย	16.00	เห็นด้วย	เห็นด้วย
เห็นด้วย	เห็นด้วยอย่	เห็นด้วยอย่	เห็นด้วยอย่	เห็นด้วยอย่	เห็นด้วย	21.00	เห็นด้วยอย่	เห็นด้วยอย่
เห็นด้วยอย่	เห็นด้วยอย่	เห็นด้วยอย่	เห็นด้วยอย่	เห็นด้วยอย่	ไม่เห็นด้วย	21.00	เห็นด้วย	เห็นด้วย
เห็นด้วย	เห็นด้วย	เห็นด้วย	เห็นด้วย	เห็นด้วย	เห็นด้วย	21.00	เห็นด้วยอย่	เห็นด้วยอย่
เห็นด้วยอย่	เห็นด้วยอย่	เห็นด้วยอย่	เห็นด้วยอย่	เห็นด้วย	ไม่เห็นด้วย	18.00	เห็นด้วย	เห็นด้วย
เห็นด้วยอย่	เห็นด้วยอย่	เห็นด้วย	เห็นด้วยอย่	เห็นด้วย	ไม่เห็นด้วย	18.00	เห็นด้วยอย่	เห็นด้วยอย่
เห็นด้วย	เห็นด้วย	เห็นด้วย	เห็นด้วย	เห็นด้วย	เห็นด้วย	19.00	เห็นด้วย	เห็นด้วย
เห็นด้วย	เห็นด้วยอย่	เห็นด้วยอย่	เห็นด้วย	เห็นด้วย	เห็นด้วยอย่	21.00	เห็นด้วยอย่	เห็นด้วยอย่
เห็นด้วยอย่	เห็นด้วยอย่	เห็นด้วย	เห็นด้วยอย่	เห็นด้วยอย่	เห็นด้วย	20.00	เห็นด้วย	เห็นด้วยอย่
เห็นด้วยอย่	เห็นด้วยอย่	เห็นด้วยอย่	เห็นด้วยอย่	เห็นด้วย	เห็นด้วย	19.00	เห็นด้วย	เห็นด้วย
เห็นด้วยอย่	เห็นด้วยอย่	เห็นด้วยอย่	เห็นด้วยอย่	เห็นด้วยอย่	เห็นด้วยอย่	18.00	เห็นด้วย	เห็นด้วยอย่
เห็นด้วยอย่	เห็นด้วยอย่	เห็นด้วย	เห็นด้วยอย่	เห็นด้วยอย่	เห็นด้วย	16.00	เห็นด้วยอย่	เห็นด้วย
เห็นด้วย	เห็นด้วย	ไม่เห็นด้วย	เห็นด้วย	เห็นด้วย	ไม่เห็นด้วย	18.00	เห็นด้วยอย่	เห็นด้วยอย่
เห็นด้วยอย่	เห็นด้วยอย่	เห็นด้วย	เห็นด้วย	เห็นด้วย	ไม่เห็นด้วย	14.00	เห็นด้วย	เห็นด้วย
เห็นด้วยอย่	เห็นด้วยอย่	เห็นด้วยอย่	เห็นด้วยอย่	เห็นด้วยอย่	เห็นด้วยอย่	19.00	เห็นด้วยอย่	เห็นด้วยอย่
เห็นด้วย	ไม่เห็นด้วย	เห็นด้วย	เห็นด้วย	เห็นด้วย	ไม่เห็นด้วย	18.00	เห็นด้วย	เห็นด้วย
เห็นด้วย	เห็นด้วย	เห็นด้วย	เห็นด้วย	เห็นด้วย	ไม่เห็นด้วย	18.00	ไม่เห็นด้วย	เห็นด้วย
ไม่เห็นด้วย	เห็นด้วยอย่	เห็นด้วยอย่	เห็นด้วยอย่	เห็นด้วยอย่	เห็นด้วยอย่	23.00	เห็นด้วยอย่	เห็นด้วยอย่
เห็นด้วยอย่	เห็นด้วยอย่	เห็นด้วยอย่	เห็นด้วยอย่	เห็นด้วยอย่	เห็นด้วยอย่	20.00	เห็นด้วยอย่	เห็นด้วยอย่
เห็นด้วย	เห็นด้วย	เห็นด้วย	เห็นด้วยอย่	เห็นด้วย	เห็นด้วย	22.00	เห็นด้วย	เห็นด้วย
เห็นด้วยอย่	เห็นด้วยอย่	เห็นด้วยอย่	เห็นด้วยอย่	เห็นด้วยอย่	เห็นด้วย	21.00	เห็นด้วยอย่	เห็นด้วยอย่
เห็นด้วยอย่	เห็นด้วยอย่	เห็นด้วยอย่	เห็นด้วย	เห็นด้วยอย่	เห็นด้วย	23.00	เห็นด้วย	เห็นด้วย
ไม่เห็นด้วย	เห็นด้วย	เห็นด้วย	เห็นด้วย	เห็นด้วย	ไม่เห็นด้วย	24.00	เห็นด้วย	เห็นด้วย
เห็นด้วย	เห็นด้วย	เห็นด้วย	เห็นด้วย	เห็นด้วย	ไม่เห็นด้วย	20.00	เห็นด้วย	เห็นด้วย
เห็นด้วยอย่	เห็นด้วยอย่	ไม่เห็นด้วย	เห็นด้วย	เห็นด้วย	ไม่เห็นด้วย	18.00	เห็นด้วยอย่	เห็นด้วย
เห็นด้วย	เห็นด้วย	เห็นด้วย	เห็นด้วย	เห็นด้วยอย่	ไม่เห็นด้วย	21.00	ไม่เห็นด้วย	ไม่เห็นด้วย
เห็นด้วย	เห็นด้วย	เห็นด้วย	เห็นด้วย	เห็นด้วย	เห็นด้วย	22.00	เห็นด้วย	เห็นด้วย
เห็นด้วยอย่	เห็นด้วยอย่	เห็นด้วย	เห็นด้วยอย่	เห็นด้วยอย่	ไม่เห็นด้วย	19.00	เห็นด้วย	เห็นด้วย
เห็นด้วย	เห็นด้วย	เห็นด้วย	เห็นด้วย	เห็นด้วย	ไม่เห็นด้วย	24.00	เห็นด้วย	เห็นด้วย
เห็นด้วยอย่	เห็นด้วยอย่	เห็นด้วย	เห็นด้วย	เห็นด้วย	ไม่เห็นด้วย	18.00	เห็นด้วย	เห็นด้วย
เห็นด้วย	เห็นด้วย	เห็นด้วย	เห็นด้วย	เห็นด้วย	ไม่เห็นด้วย	20.00	เห็นด้วย	เห็นด้วย
เห็นด้วย	เห็นด้วยอย่	เห็นด้วยอย่	เห็นด้วยอย่	เห็นด้วยอย่	เห็นด้วย	22.00	เห็นด้วยอย่	เห็นด้วยอย่
เห็นด้วยอย่	เห็นด้วยอย่	เห็นด้วยอย่	เห็นด้วยอย่	เห็นด้วยอย่	ไม่เห็นด้วย	20.00	เห็นด้วย	เห็นด้วย
เห็นด้วย	เห็นด้วย	เห็นด้วย	เห็นด้วย	เห็นด้วย	เห็นด้วย	18.00	เห็นด้วยอย่	เห็นด้วยอย่

เห็นด้วยอย่	เห็นด้วยอย่	เห็นด้วยอย่	เห็นด้วยอย่	เห็นด้วย	ไม่เห็นด้วย	21.00	เห็นด้วย	เห็นด้วย
เห็นด้วยอย่	เห็นด้วยอย่	เห็นด้วย	เห็นด้วยอย่	เห็นด้วย	ไม่เห็นด้วย	18.00	เห็นด้วยอย่	เห็นด้วยอย่
เห็นด้วย	เห็นด้วย	เห็นด้วย	เห็นด้วย	เห็นด้วย	เห็นด้วย	17.00	เห็นด้วย	เห็นด้วย
เห็นด้วย	เห็นด้วยอย่	เห็นด้วยอย่	เห็นด้วย	เห็นด้วย	เห็นด้วยอย่	22.00	เห็นด้วยอย่	เห็นด้วยอย่
เห็นด้วยอย่	เห็นด้วยอย่	เห็นด้วย	เห็นด้วยอย่	เห็นด้วยอย่	เห็นด้วย	18.00	เห็นด้วย	เห็นด้วยอย่
เห็นด้วย	เห็นด้วย	เห็นด้วย	เห็นด้วยอย่	เห็นด้วย	เห็นด้วย	18.00	เห็นด้วย	เห็นด้วย
เห็นด้วย	เห็นด้วย	เห็นด้วย	เห็นด้วย	เห็นด้วย	เห็นด้วย	18.00	เห็นด้วยอย่	เห็นด้วยอย่
เห็นด้วย	เห็นด้วย	เห็นด้วย	เห็นด้วย	เห็นด้วย	เห็นด้วย	18.00	เห็นด้วย	เห็นด้วย
เห็นด้วย	เห็นด้วย	เห็นด้วยอย่	เห็นด้วยอย่	เห็นด้วย	เห็นด้วย	21.00	เห็นด้วย	เห็นด้วย
เห็นด้วย	ไม่เห็นด้วย	เห็นด้วย	เห็นด้วย	เห็นด้วย	ไม่เห็นด้วย	21.00	เห็นด้วย	เห็นด้วย
เห็นด้วย	เห็นด้วย	เห็นด้วย	เห็นด้วย	เห็นด้วย	ไม่เห็นด้วย	21.00	เห็นด้วย	ไม่เห็นด้วย
ไม่เห็นด้วยอ	เห็นด้วยอย่	เห็นด้วยอย่	เห็นด้วยอย่	เห็นด้วยอย่	เห็นด้วยอย่	18.00	เห็นด้วยอย่	เห็นด้วยอย่
เห็นด้วยอย่	เห็นด้วยอย่	เห็นด้วยอย่	เห็นด้วยอย่	เห็นด้วยอย่	เห็นด้วยอย่	18.00	เห็นด้วยอย่	เห็นด้วยอย่
เห็นด้วย	เห็นด้วย	เห็นด้วย	เห็นด้วยอย่	เห็นด้วย	เห็นด้วย	20.00	เห็นด้วย	เห็นด้วย
เห็นด้วยอย่	เห็นด้วยอย่	เห็นด้วยอย่	เห็นด้วยอย่	เห็นด้วยอย่	เห็นด้วย	18.00	เห็นด้วยอย่	เห็นด้วยอย่
เห็นด้วยอย่	เห็นด้วยอย่	เห็นด้วยอย่	เห็นด้วย	เห็นด้วยอย่	เห็นด้วย	18.00	เห็นด้วย	เห็นด้วย
ไม่เห็นด้วย	เห็นด้วย	เห็นด้วย	เห็นด้วย	เห็นด้วย	ไม่เห็นด้วย	22.00	เห็นด้วย	เห็นด้วย
เห็นด้วย	เห็นด้วย	เห็นด้วย	เห็นด้วย	เห็นด้วย	ไม่เห็นด้วย	18.00	เห็นด้วย	เห็นด้วย
เห็นด้วย	เห็นด้วย	ไม่เห็นด้วย	เห็นด้วย	เห็นด้วย	ไม่เห็นด้วย	18.00	เห็นด้วย	ไม่เห็นด้วย
เห็นด้วย	เห็นด้วย	เห็นด้วย	เห็นด้วย	เห็นด้วยอย่	ไม่เห็นด้วย	18.00	ไม่เห็นด้วยอ	ไม่เห็นด้วยอ
เห็นด้วย	เห็นด้วย	เห็นด้วย	เห็นด้วย	เห็นด้วย	เห็นด้วย	22.00	เห็นด้วย	เห็นด้วย
เห็นด้วยอย่	เห็นด้วยอย่	เห็นด้วย	เห็นด้วยอย่	เห็นด้วยอย่	ไม่เห็นด้วย	23.00	เห็นด้วย	เห็นด้วย
เห็นด้วย	เห็นด้วย	เห็นด้วย	เห็นด้วย	เห็นด้วย	ไม่เห็นด้วย	17.00	เห็นด้วย	เห็นด้วย
เห็นด้วยอย่	เห็นด้วยอย่	เห็นด้วย	เห็นด้วย	เห็นด้วย	ไม่เห็นด้วย	17.00	เห็นด้วย	เห็นด้วยอย่
เห็นด้วย	เห็นด้วย	เห็นด้วย	เห็นด้วย	เห็นด้วย	ไม่เห็นด้วย	18.00	เห็นด้วย	เห็นด้วย
เห็นด้วย	เห็นด้วย	เห็นด้วย	เห็นด้วย	เห็นด้วย	ไม่เห็นด้วย	20.00	เห็นด้วยอย่	เห็นด้วยอย่
เห็นด้วยอย่	เห็นด้วยอย่	เห็นด้วยอย่	เห็นด้วยอย่	เห็นด้วยอย่	เห็นด้วย	21.00	เห็นด้วยอย่	เห็นด้วยอย่
เห็นด้วย	เห็นด้วย	เห็นด้วย	เห็นด้วย	เห็นด้วย	ไม่เห็นด้วย	24.00	เห็นด้วย	เห็นด้วย
เห็นด้วยอย่	เห็นด้วย	เห็นด้วย	เห็นด้วยอย่	เห็นด้วย	เห็นด้วย	24.00	เห็นด้วย	เห็นด้วย
เห็นด้วย	เห็นด้วย	เห็นด้วย	เห็นด้วย	เห็นด้วยอย่	เห็นด้วย	24.00	เห็นด้วย	เห็นด้วย
เห็นด้วยอย่	เห็นด้วยอย่	เห็นด้วยอย่	เห็นด้วยอย่	เห็นด้วยอย่	เห็นด้วยอย่	18.00	เห็นด้วยอย่	เห็นด้วยอย่
เห็นด้วยอย่	เห็นด้วยอย่	เห็นด้วยอย่	เห็นด้วยอย่	เห็นด้วยอย่	เห็นด้วยอย่	21.00	เห็นด้วยอย่	เห็นด้วยอย่
เห็นด้วยอย่	เห็นด้วยอย่	เห็นด้วยอย่	เห็นด้วยอย่	เห็นด้วยอย่	เห็นด้วย	22.00	เห็นด้วย	เห็นด้วย
เห็นด้วย	เห็นด้วย	เห็นด้วย	เห็นด้วย	เห็นด้วยอย่	เห็นด้วยอย่	18.00	เห็นด้วย	เห็นด้วย
เห็นด้วยอย่	เห็นด้วยอย่	เห็นด้วยอย่	เห็นด้วยอย่	เห็นด้วยอย่	เห็นด้วยอย่	18.00	เห็นด้วย	เห็นด้วย
เห็นด้วยอย่	เห็นด้วยอย่	เห็นด้วยอย่	เห็นด้วยอย่	เห็นด้วยอย่	เห็นด้วยอย่	19.00	เห็นด้วย	เห็นด้วย
เห็นด้วยอย่	เห็นด้วยอย่	เห็นด้วยอย่	เห็นด้วยอย่	เห็นด้วยอย่	เห็นด้วยอย่	18.00	เห็นด้วยอย่	เห็นด้วยอย่
เห็นด้วย	เห็นด้วย	เห็นด้วย	เห็นด้วย	เห็นด้วย	เห็นด้วย	20.00	เห็นด้วย	เห็นด้วย
เห็นด้วยอย่	เห็นด้วยอย่	เห็นด้วยอย่	เห็นด้วยอย่	เห็นด้วยอย่	เห็นด้วยอย่	22.00	เห็นด้วย	เห็นด้วย
เห็นด้วยอย่	เห็นด้วยอย่	เห็นด้วยอย่	เห็นด้วยอย่	เห็นด้วยอย่	เห็นด้วยอย่	22.00	เห็นด้วยอย่	เห็นด้วยอย่
เห็นด้วย	เห็นด้วย	เห็นด้วย	เห็นด้วย	เห็นด้วย	เห็นด้วย	18.00	เห็นด้วย	เห็นด้วย
เห็นด้วยอย่	เห็นด้วยอย่	เห็นด้วยอย่	เห็นด้วยอย่	เห็นด้วยอย่	เห็นด้วยอย่	21.00	เห็นด้วยอย่	เห็นด้วยอย่
เห็นด้วยอย่	เห็นด้วยอย่	เห็นด้วยอย่	เห็นด้วยอย่	เห็นด้วยอย่	เห็นด้วยอย่	21.00	เห็นด้วยอย่	เห็นด้วยอย่
เห็นด้วย	เห็นด้วย	เห็นด้วย	เห็นด้วย	เห็นด้วย	ไม่เห็นด้วย	18.00	เห็นด้วย	เห็นด้วย
เห็นด้วย	เห็นด้วย	เห็นด้วย	เห็นด้วย	เห็นด้วย	เห็นด้วย	18.00	เห็นด้วย	เห็นด้วย
เห็นด้วยอย่	เห็นด้วยอย่	เห็นด้วยอย่	เห็นด้วยอย่	เห็นด้วยอย่	เห็นด้วยอย่	24.00	เห็นด้วย	เห็นด้วย
เห็นด้วยอย่	เห็นด้วยอย่	เห็นด้วยอย่	เห็นด้วยอย่	เห็นด้วยอย่	เห็นด้วยอย่	18.00	เห็นด้วยอย่	เห็นด้วยอย่
เห็นด้วย	เห็นด้วย	เห็นด้วย	เห็นด้วย	เห็นด้วย	เห็นด้วย	24.00	เห็นด้วย	เห็นด้วย
เห็นด้วย	เห็นด้วย	เห็นด้วย	เห็นด้วย	เห็นด้วย	เห็นด้วย	18.00	เห็นด้วย	เห็นด้วย
เห็นด้วย	เห็นด้วย	เห็นด้วย	เห็นด้วย	เห็นด้วย	เห็นด้วย	18.00	เห็นด้วย	เห็นด้วย
เห็นด้วย	เห็นด้วย	เห็นด้วย	เห็นด้วย	เห็นด้วย	เห็นด้วย	18.00	เห็นด้วย	เห็นด้วย

เห็นด้วย	เห็นด้วย	เห็นด้วย	เห็นด้วย	เห็นด้วย	เห็นด้วย	17.00	เห็นด้วย	เห็นด้วย
เห็นด้วย	เห็นด้วย	เห็นด้วย	เห็นด้วย	เห็นด้วย	เห็นด้วย	18.00	เห็นด้วย	ไม่เห็นด้วย
เห็นด้วยอย่	เห็นด้วยอย่	เห็นด้วย	เห็นด้วยอย่	เห็นด้วยอย่	เห็นด้วย	20.00	เห็นด้วยอย่	เห็นด้วยอย่
เห็นด้วยอย่	เห็นด้วยอย่	เห็นด้วย	เห็นด้วยอย่	เห็นด้วยอย่	ไม่เห็นด้วย	17.00	เห็นด้วย	เห็นด้วยอย่
เห็นด้วยอย่	เห็นด้วยอย่	เห็นด้วยอย่	เห็นด้วยอย่	เห็นด้วยอย่	เห็นด้วยอย่	24.00	เห็นด้วยอย่	เห็นด้วยอย่
เห็นด้วยอย่	เห็นด้วยอย่	เห็นด้วย	เห็นด้วยอย่	เห็นด้วยอย่	เห็นด้วย	18.00	เห็นด้วยอย่	เห็นด้วย
เห็นด้วย	เห็นด้วย	เห็นด้วย	เห็นด้วย	เห็นด้วย	เห็นด้วย	23.00	เห็นด้วย	เห็นด้วย
เห็นด้วยอย่	เห็นด้วยอย่	เห็นด้วยอย่	เห็นด้วยอย่	เห็นด้วยอย่	เห็นด้วยอย่	22.00	เห็นด้วยอย่	เห็นด้วย
เห็นด้วยอย่	เห็นด้วยอย่	เห็นด้วยอย่	เห็นด้วยอย่	เห็นด้วยอย่	ไม่เห็นด้วย	18.00	เห็นด้วยอย่	เห็นด้วย
เห็นด้วยอย่	เห็นด้วยอย่	เห็นด้วยอย่	เห็นด้วยอย่	เห็นด้วยอย่	เห็นด้วย	18.00	เห็นด้วย	เห็นด้วยอย่
เห็นด้วยอย่	เห็นด้วยอย่	เห็นด้วยอย่	เห็นด้วยอย่	เห็นด้วยอย่	เห็นด้วยอย่	22.00	เห็นด้วยอย่	เห็นด้วย
เห็นด้วย	เห็นด้วย	เห็นด้วย	เห็นด้วย	เห็นด้วย	เห็นด้วย	22.00	เห็นด้วย	เห็นด้วย
เห็นด้วย	เห็นด้วย	เห็นด้วย	เห็นด้วย	เห็นด้วย	เห็นด้วย	21.00	เห็นด้วย	เห็นด้วย
เห็นด้วยอย่	เห็นด้วยอย่	เห็นด้วยอย่	เห็นด้วยอย่	เห็นด้วยอย่	ไม่เห็นด้วย	19.00	เห็นด้วย	เห็นด้วยอย่
เห็นด้วย	เห็นด้วย	เห็นด้วย	เห็นด้วย	เห็นด้วย	เห็นด้วย	18.00	เห็นด้วย	เห็นด้วย
ไม่เห็นด้วย	เห็นด้วย	เห็นด้วยอย่	เห็นด้วยอย่	เห็นด้วยอย่	เห็นด้วยอย่	22.00	เห็นด้วยอย่	เห็นด้วยอย่
เห็นด้วย	เห็นด้วย	เห็นด้วย	เห็นด้วย	เห็นด้วย	เห็นด้วย	18.00	เห็นด้วย	เห็นด้วย
เห็นด้วยอย่	เห็นด้วยอย่	เห็นด้วยอย่	เห็นด้วยอย่	เห็นด้วยอย่	เห็นด้วย	23.00	เห็นด้วย	เห็นด้วย
เห็นด้วยอย่	เห็นด้วยอย่	เห็นด้วย	เห็นด้วย	เห็นด้วยอย่	เห็นด้วย	21.00	เห็นด้วย	เห็นด้วย
เห็นด้วย	เห็นด้วย	เห็นด้วย	เห็นด้วย	เห็นด้วย	เห็นด้วย	18.00	เห็นด้วยอย่	เห็นด้วยอย่
เห็นด้วย	เห็นด้วย	เห็นด้วย	เห็นด้วย	เห็นด้วย	เห็นด้วย	22.00	เห็นด้วย	เห็นด้วย
เห็นด้วย	เห็นด้วย	เห็นด้วย	เห็นด้วย	เห็นด้วย	เห็นด้วย	21.00	เห็นด้วยอย่	เห็นด้วยอย่
เห็นด้วย	เห็นด้วย	เห็นด้วย	เห็นด้วย	เห็นด้วย	เห็นด้วย	22.00	เห็นด้วย	เห็นด้วย
เห็นด้วยอย่	เห็นด้วย	เห็นด้วยอย่	เห็นด้วย	เห็นด้วย	เห็นด้วย	23.00	เห็นด้วย	เห็นด้วย
เห็นด้วยอย่	เห็นด้วยอย่	เห็นด้วย	เห็นด้วย	เห็นด้วย	ไม่เห็นด้วย	24.00	เห็นด้วยอย่	เห็นด้วยอย่
เห็นด้วย	เห็นด้วย	เห็นด้วย	เห็นด้วย	เห็นด้วย	เห็นด้วย	18.00	เห็นด้วย	เห็นด้วย
เห็นด้วยอย่	เห็นด้วยอย่	เห็นด้วย	เห็นด้วย	เห็นด้วย	ไม่เห็นด้วย	18.00	เห็นด้วย	เห็นด้วย
เห็นด้วย	เห็นด้วย	เห็นด้วย	เห็นด้วย	เห็นด้วย	เห็นด้วย	18.00	เห็นด้วย	เห็นด้วย
เห็นด้วย	เห็นด้วย	เห็นด้วย	เห็นด้วย	เห็นด้วย	เห็นด้วย	17.00	เห็นด้วยอย่	เห็นด้วยอย่
เห็นด้วยอย่	เห็นด้วยอย่	เห็นด้วยอย่	เห็นด้วย	เห็นด้วย	เห็นด้วย	22.00	เห็นด้วยอย่	เห็นด้วยอย่
เห็นด้วย	เห็นด้วยอย่	เห็นด้วยอย่	เห็นด้วยอย่	เห็นด้วย	เห็นด้วย	19.00	เห็นด้วยอย่	เห็นด้วย
เห็นด้วย	เห็นด้วย	เห็นด้วย	เห็นด้วย	เห็นด้วย	เห็นด้วย	18.00	เห็นด้วย	เห็นด้วย
เห็นด้วย	เห็นด้วย	เห็นด้วย	เห็นด้วย	เห็นด้วย	เห็นด้วย	22.00	เห็นด้วย	เห็นด้วย
เห็นด้วยอย่	เห็นด้วยอย่	เห็นด้วย	เห็นด้วยอย่	เห็นด้วยอย่	เห็นด้วย	15.00	เห็นด้วยอย่	เห็นด้วยอย่
เห็นด้วย	เห็นด้วย	เห็นด้วย	เห็นด้วยอย่	เห็นด้วย	เห็นด้วย	18.00	เห็นด้วย	เห็นด้วย
เห็นด้วยอย่	เห็นด้วยอย่	เห็นด้วย	เห็นด้วยอย่	เห็นด้วยอย่	เห็นด้วย	18.00	เห็นด้วย	เห็นด้วยอย่
เห็นด้วย	เห็นด้วย	เห็นด้วย	เห็นด้วย	เห็นด้วย	ไม่เห็นด้วย	24.00	เห็นด้วยอย่	เห็นด้วย
เห็นด้วย	เห็นด้วย	ไม่เห็นด้วย	เห็นด้วย	เห็นด้วย	เห็นด้วย	20.00	เห็นด้วยอย่	เห็นด้วย
เห็นด้วยอย่	เห็นด้วยอย่	ไม่เห็นด้วย	เห็นด้วยอย่	เห็นด้วยอย่	เห็นด้วย	17.00	เห็นด้วย	เห็นด้วย
เห็นด้วย	เห็นด้วย	เห็นด้วย	เห็นด้วย	เห็นด้วย	เห็นด้วย	18.00	เห็นด้วย	เห็นด้วย
เห็นด้วย	เห็นด้วย	เห็นด้วย	เห็นด้วย	เห็นด้วย	เห็นด้วย	18.00	เห็นด้วย	เห็นด้วย
เห็นด้วย	เห็นด้วย	เห็นด้วย	เห็นด้วย	เห็นด้วย	เห็นด้วย	24.00	เห็นด้วย	เห็นด้วย
เห็นด้วย	เห็นด้วย	เห็นด้วย	เห็นด้วย	เห็นด้วย	เห็นด้วย	18.00	เห็นด้วย	เห็นด้วย
เห็นด้วย	เห็นด้วย	เห็นด้วย	เห็นด้วย	เห็นด้วย	เห็นด้วย	18.00	เห็นด้วย	ไม่เห็นด้วย
เห็นด้วยอย่	เห็นด้วยอย่	เห็นด้วย	เห็นด้วยอย่	เห็นด้วยอย่	เห็นด้วย	22.00	เห็นด้วยอย่	เห็นด้วยอย่
เห็นด้วยอย่	เห็นด้วยอย่	เห็นด้วย	เห็นด้วยอย่	เห็นด้วยอย่	ไม่เห็นด้วย	17.00	เห็นด้วย	เห็นด้วยอย่
เห็นด้วย	เห็นด้วย	เห็นด้วย	เห็นด้วย	เห็นด้วย	เห็นด้วย	19.00	เห็นด้วย	เห็นด้วย
เห็นด้วย	เห็นด้วย	เห็นด้วย	เห็นด้วย	เห็นด้วย	เห็นด้วย	18.00	เห็นด้วย	เห็นด้วย
เห็นด้วย	เห็นด้วย	เห็นด้วย	เห็นด้วย	เห็นด้วย	เห็นด้วย	24.00	เห็นด้วย	เห็นด้วย

benefit3	benefit4	benefit5	benefit6	benefit7	Sum_Bene	barrier1	barrier2	barrier3
เห็นด้วย	เห็นด้วย	ไม่เห็นด้วย	เห็นด้วย	เห็นด้วย	21.00	ไม่เห็นด้วย	เห็นด้วย	ไม่เห็นด้วย
ไม่เห็นด้วย	ไม่เห็นด้วย	ไม่เห็นด้วย	เห็นด้วย	เห็นด้วย	25.00	ไม่เห็นด้วย	ไม่เห็นด้วย	ไม่เห็นด้วย
เห็นด้วยอย่	เห็นด้วย	ไม่เห็นด้วย	เห็นด้วย	เห็นด้วย	23.00	ไม่เห็นด้วย	เห็นด้วย	เห็นด้วย
ไม่เห็นด้วย	ไม่เห็นด้วย	เห็นด้วยอย่	เห็นด้วยอย่	ไม่เห็นด้วยอ	18.00	ไม่เห็นด้วย	ไม่เห็นด้วย	ไม่เห็นด้วย
เห็นด้วย	เห็นด้วย	ไม่เห็นด้วย	เห็นด้วย	เห็นด้วย	21.00	ไม่เห็นด้วย	เห็นด้วย	เห็นด้วย
เห็นด้วย	เห็นด้วย	เห็นด้วย	เห็นด้วย	เห็นด้วย	26.00	เห็นด้วย	เห็นด้วย	เห็นด้วย
ไม่เห็นด้วย	เห็นด้วยอย่	เห็นด้วยอย่	เห็นด้วยอย่	เห็นด้วย	21.00	ไม่เห็นด้วย	ไม่เห็นด้วย	เห็นด้วย
เห็นด้วยอย่	เห็นด้วยอย่	เห็นด้วยอย่	เห็นด้วยอย่	เห็นด้วยอย่	24.00	ไม่เห็นด้วยอ	เห็นด้วย	ไม่เห็นด้วยอ
ไม่เห็นด้วย	เห็นด้วย	ไม่เห็นด้วย	ไม่เห็นด้วย	ไม่เห็นด้วย	21.00	ไม่เห็นด้วย	เห็นด้วย	เห็นด้วย
เห็นด้วย	เห็นด้วย	เห็นด้วย	เห็นด้วยอย่	เห็นด้วยอย่	21.00	เห็นด้วย	เห็นด้วย	เห็นด้วยอย่
ไม่เห็นด้วย	เห็นด้วย	ไม่เห็นด้วย	เห็นด้วย	เห็นด้วย	21.00	ไม่เห็นด้วย	ไม่เห็นด้วย	เห็นด้วย
ไม่เห็นด้วย	เห็นด้วย	เห็นด้วย	เห็นด้วย	เห็นด้วย	21.00	ไม่เห็นด้วย	ไม่เห็นด้วย	ไม่เห็นด้วย
เห็นด้วย	เห็นด้วย	เห็นด้วย	เห็นด้วย	เห็นด้วย	26.00	เห็นด้วย	เห็นด้วย	เห็นด้วย
เห็นด้วย	เห็นด้วย	เห็นด้วย	เห็นด้วยอย่	เห็นด้วย	21.00	ไม่เห็นด้วย	ไม่เห็นด้วย	ไม่เห็นด้วย
เห็นด้วย	ไม่เห็นด้วย	ไม่เห็นด้วย	เห็นด้วย	เห็นด้วย	20.00	ไม่เห็นด้วย	ไม่เห็นด้วย	เห็นด้วย
เห็นด้วย	เห็นด้วย	เห็นด้วย	เห็นด้วย	เห็นด้วย	21.00	ไม่เห็นด้วย	เห็นด้วย	ไม่เห็นด้วย
เห็นด้วย	เห็นด้วย	เห็นด้วยอย่	เห็นด้วย	เห็นด้วย	21.00	เห็นด้วยอย่	เห็นด้วย	เห็นด้วย
เห็นด้วย	ไม่เห็นด้วยอ	ไม่เห็นด้วย	เห็นด้วย	เห็นด้วย	22.00	ไม่เห็นด้วย	ไม่เห็นด้วย	เห็นด้วย
เห็นด้วยอย่	เห็นด้วย	เห็นด้วย	เห็นด้วย	เห็นด้วย	20.00	ไม่เห็นด้วย	เห็นด้วย	ไม่เห็นด้วย
เห็นด้วย	เห็นด้วย	เห็นด้วย	เห็นด้วย	เห็นด้วย	22.00	เห็นด้วยอย่	เห็นด้วย	ไม่เห็นด้วย
เห็นด้วย	ไม่เห็นด้วย	ไม่เห็นด้วย	เห็นด้วย	เห็นด้วย	21.00	ไม่เห็นด้วย	เห็นด้วย	ไม่เห็นด้วย
ไม่เห็นด้วย	ไม่เห็นด้วย	เห็นด้วย	ไม่เห็นด้วย	ไม่เห็นด้วย	20.00	ไม่เห็นด้วย	ไม่เห็นด้วย	เห็นด้วย
เห็นด้วยอย่	เห็นด้วยอย่	เห็นด้วยอย่	เห็นด้วยอย่	เห็นด้วยอย่	22.00	เห็นด้วยอย่	เห็นด้วยอย่	ไม่เห็นด้วยอ
ไม่เห็นด้วย	เห็นด้วย	เห็นด้วย	เห็นด้วยอย่	เห็นด้วย	19.00	ไม่เห็นด้วย	เห็นด้วย	เห็นด้วย
เห็นด้วยอย่	เห็นด้วยอย่	เห็นด้วยอย่	เห็นด้วย	เห็นด้วยอย่	21.00	ไม่เห็นด้วย	ไม่เห็นด้วย	ไม่เห็นด้วย
เห็นด้วยอย่	เห็นด้วย	เห็นด้วย	เห็นด้วย	เห็นด้วย	22.00	เห็นด้วย	เห็นด้วยอย่	ไม่เห็นด้วย
เห็นด้วยอย่	เห็นด้วย	เห็นด้วย	เห็นด้วยอย่	เห็นด้วยอย่	27.00	ไม่เห็นด้วยอ	เห็นด้วยอย่	เห็นด้วย
เห็นด้วย	เห็นด้วยอย่	ไม่เห็นด้วย	เห็นด้วย	เห็นด้วยอย่	25.00	ไม่เห็นด้วย	ไม่เห็นด้วย	ไม่เห็นด้วย
เห็นด้วยอย่	เห็นด้วยอย่	เห็นด้วย	เห็นด้วยอย่	เห็นด้วยอย่	26.00	ไม่เห็นด้วย	เห็นด้วย	ไม่เห็นด้วย
เห็นด้วย	เห็นด้วย	เห็นด้วย	เห็นด้วย	เห็นด้วย	22.00	ไม่เห็นด้วย	เห็นด้วย	เห็นด้วย
เห็นด้วย	เห็นด้วย	เห็นด้วย	เห็นด้วย	เห็นด้วย	19.00	เห็นด้วย	เห็นด้วย	เห็นด้วย
เห็นด้วย	เห็นด้วย	ไม่เห็นด้วย	เห็นด้วย	เห็นด้วยอย่	18.00	ไม่เห็นด้วย	ไม่เห็นด้วย	เห็นด้วย
เห็นด้วย	เห็นด้วย	เห็นด้วย	เห็นด้วย	เห็นด้วย	25.00	เห็นด้วยอย่	เห็นด้วย	เห็นด้วย
เห็นด้วยอย่	เห็นด้วยอย่	เห็นด้วยอย่	เห็นด้วยอย่	เห็นด้วยอย่	27.00	เห็นด้วยอย่	เห็นด้วยอย่	เห็นด้วยอย่
เห็นด้วย	เห็นด้วย	เห็นด้วย	เห็นด้วย	เห็นด้วย	24.00	ไม่เห็นด้วย	เห็นด้วย	เห็นด้วย
เห็นด้วย	เห็นด้วย	เห็นด้วย	เห็นด้วย	เห็นด้วย	21.00	ไม่เห็นด้วย	เห็นด้วย	ไม่เห็นด้วย
เห็นด้วย	เห็นด้วยอย่	ไม่เห็นด้วย	เห็นด้วย	เห็นด้วยอย่	22.00	ไม่เห็นด้วย	เห็นด้วย	เห็นด้วย
เห็นด้วย	เห็นด้วย	เห็นด้วย	เห็นด้วย	เห็นด้วย	22.00	ไม่เห็นด้วย	ไม่เห็นด้วย	ไม่เห็นด้วย
เห็นด้วย	เห็นด้วย	ไม่เห็นด้วย	เห็นด้วย	เห็นด้วย	20.00	ไม่เห็นด้วย	ไม่เห็นด้วย	ไม่เห็นด้วย
เห็นด้วย	เห็นด้วย	ไม่เห็นด้วย	เห็นด้วย	เห็นด้วย	24.00	ไม่เห็นด้วย	เห็นด้วย	เห็นด้วย
เห็นด้วย	เห็นด้วย	ไม่เห็นด้วย	เห็นด้วย	เห็นด้วย	25.00	ไม่เห็นด้วย	เห็นด้วย	เห็นด้วย
ไม่เห็นด้วย	เห็นด้วย	ไม่เห็นด้วย	เห็นด้วย	เห็นด้วย	21.00	ไม่เห็นด้วย	เห็นด้วย	เห็นด้วย
เห็นด้วยอย่	เห็นด้วย	เห็นด้วย	เห็นด้วย	เห็นด้วย	21.00	เห็นด้วยอย่	เห็นด้วย	เห็นด้วย
เห็นด้วย	เห็นด้วย	เห็นด้วย	เห็นด้วย	เห็นด้วย	21.00	เห็นด้วย	เห็นด้วย	เห็นด้วย
เห็นด้วยอย่	เห็นด้วยอย่	เห็นด้วย	เห็นด้วย	เห็นด้วยอย่	21.00	ไม่เห็นด้วย	ไม่เห็นด้วย	ไม่เห็นด้วย
เห็นด้วย	เห็นด้วย	ไม่เห็นด้วย	เห็นด้วย	เห็นด้วย	20.00	ไม่เห็นด้วย	ไม่เห็นด้วย	ไม่เห็นด้วย
เห็นด้วยอย่	เห็นด้วยอย่	เห็นด้วยอย่	เห็นด้วยอย่	เห็นด้วยอย่	21.00	เห็นด้วยอย่	เห็นด้วย	เห็นด้วย
เห็นด้วยอย่	เห็นด้วย	เห็นด้วย	เห็นด้วย	เห็นด้วย	21.00	เห็นด้วย	เห็นด้วย	เห็นด้วย
เห็นด้วย	เห็นด้วยอย่	เห็นด้วย	เห็นด้วย	เห็นด้วย	28.00	ไม่เห็นด้วย	เห็นด้วย	ไม่เห็นด้วย
เห็นด้วย	ไม่เห็นด้วย	ไม่เห็นด้วย	เห็นด้วย	เห็นด้วย	24.00	ไม่เห็นด้วย	ไม่เห็นด้วย	เห็นด้วย

เห็นด้วย	เห็นด้วย	เห็นด้วย	เห็นด้วย	เห็นด้วย	24.00	ไม่เห็นด้วย	เห็นด้วย	ไม่เห็นด้วย
เห็นด้วย	เห็นด้วยอย่	เห็นด้วย	เห็นด้วย	เห็นด้วย	23.00	ไม่เห็นด้วย	เห็นด้วย	ไม่เห็นด้วย
ไม่เห็นด้วย	เห็นด้วย	ไม่เห็นด้วย	เห็นด้วยอย่	เห็นด้วยอย่	21.00	ไม่เห็นด้วย	ไม่เห็นด้วย	ไม่เห็นด้วย
เห็นด้วยอย่	เห็นด้วยอย่	เห็นด้วย	เห็นด้วยอย่	เห็นด้วยอย่	19.00	ไม่เห็นด้วย	ไม่เห็นด้วย	ไม่เห็นด้วย
เห็นด้วยอย่	เห็นด้วยอย่	เห็นด้วยอย่	เห็นด้วยอย่	ไม่เห็นด้วย	25.00	เห็นด้วยอย่	เห็นด้วย	ไม่เห็นด้วย
ไม่เห็นด้วย	เห็นด้วย	เห็นด้วย	เห็นด้วย	เห็นด้วย	25.00	เห็นด้วย	ไม่เห็นด้วย	เห็นด้วยอย่
เห็นด้วย	เห็นด้วย	เห็นด้วย	เห็นด้วย	เห็นด้วย	20.00	ไม่เห็นด้วย	ไม่เห็นด้วย	ไม่เห็นด้วย
เห็นด้วย	เห็นด้วย	เห็นด้วย	เห็นด้วย	เห็นด้วย	22.00	เห็นด้วย	เห็นด้วย	เห็นด้วย
เห็นด้วย	เห็นด้วย	ไม่เห็นด้วย	ไม่เห็นด้วย	เห็นด้วย	24.00	เห็นด้วย	เห็นด้วย	ไม่เห็นด้วย
เห็นด้วย	เห็นด้วย	ไม่เห็นด้วย	เห็นด้วย	เห็นด้วยอย่	23.00	ไม่เห็นด้วย	ไม่เห็นด้วย	ไม่เห็นด้วย
เห็นด้วย	เห็นด้วย	เห็นด้วย	เห็นด้วย	เห็นด้วย	22.00	เห็นด้วย	เห็นด้วย	เห็นด้วย
ไม่เห็นด้วย	เห็นด้วยอย่	เห็นด้วย	เห็นด้วยอย่	เห็นด้วย	25.00	ไม่เห็นด้วย	ไม่เห็นด้วย	เห็นด้วย
เห็นด้วยอย่	เห็นด้วยอย่	เห็นด้วยอย่	เห็นด้วยอย่	เห็นด้วยอย่	20.00	ไม่เห็นด้วย	เห็นด้วย	เห็นด้วย
เห็นด้วย	เห็นด้วยอย่	เห็นด้วย	เห็นด้วย	เห็นด้วยอย่	26.00	ไม่เห็นด้วย	ไม่เห็นด้วย	ไม่เห็นด้วย
เห็นด้วย	เห็นด้วย	เห็นด้วยอย่	เห็นด้วยอย่	เห็นด้วยอย่	28.00	ไม่เห็นด้วย	ไม่เห็นด้วย	ไม่เห็นด้วย
เห็นด้วย	เห็นด้วย	เห็นด้วย	เห็นด้วย	เห็นด้วย	24.00	เห็นด้วย	ไม่เห็นด้วย	ไม่เห็นด้วย
เห็นด้วย	เห็นด้วย	เห็นด้วย	เห็นด้วย	เห็นด้วย	26.00	ไม่เห็นด้วย	ไม่เห็นด้วย	ไม่เห็นด้วย
เห็นด้วย	เห็นด้วย	เห็นด้วย	เห็นด้วย	เห็นด้วย	23.00	ไม่เห็นด้วย	เห็นด้วย	ไม่เห็นด้วย
เห็นด้วย	เห็นด้วย	เห็นด้วย	เห็นด้วย	เห็นด้วย	21.00	เห็นด้วย	ไม่เห็นด้วย	เห็นด้วย
เห็นด้วย	เห็นด้วย	เห็นด้วย	เห็นด้วย	เห็นด้วย	19.00	ไม่เห็นด้วย	เห็นด้วย	เห็นด้วย
เห็นด้วย	เห็นด้วย	เห็นด้วย	เห็นด้วย	เห็นด้วย	27.00	ไม่เห็นด้วย	เห็นด้วย	ไม่เห็นด้วย
เห็นด้วย	เห็นด้วยอย่	เห็นด้วย	เห็นด้วย	เห็นด้วยอย่	22.00	ไม่เห็นด้วย	ไม่เห็นด้วย	ไม่เห็นด้วย
เห็นด้วยอย่	เห็นด้วยอย่	เห็นด้วย	เห็นด้วย	เห็นด้วย	21.00	ไม่เห็นด้วย	เห็นด้วย	เห็นด้วย
ไม่เห็นด้วย	เห็นด้วย	ไม่เห็นด้วย	เห็นด้วย	เห็นด้วย	21.00	ไม่เห็นด้วย	เห็นด้วย	ไม่เห็นด้วย
เห็นด้วย	ไม่เห็นด้วย	ไม่เห็นด้วย	เห็นด้วย	เห็นด้วย	21.00	ไม่เห็นด้วย	ไม่เห็นด้วย	ไม่เห็นด้วย
เห็นด้วย	ไม่เห็นด้วย	เห็นด้วย	ไม่เห็นด้วย	เห็นด้วย	21.00	ไม่เห็นด้วย	ไม่เห็นด้วย	ไม่เห็นด้วย
เห็นด้วย	เห็นด้วยอย่	เห็นด้วย	เห็นด้วย	เห็นด้วย	21.00	ไม่เห็นด้วย	เห็นด้วย	ไม่เห็นด้วย
เห็นด้วย	เห็นด้วย	เห็นด้วย	เห็นด้วย	เห็นด้วย	21.00	ไม่เห็นด้วย	ไม่เห็นด้วย	ไม่เห็นด้วย
เห็นด้วย	เห็นด้วย	เห็นด้วย	เห็นด้วย	เห็นด้วย	21.00	เห็นด้วย	เห็นด้วย	เห็นด้วย
เห็นด้วยอย่	เห็นด้วย	เห็นด้วย	เห็นด้วย	เห็นด้วย	16.00	ไม่เห็นด้วย	เห็นด้วย	ไม่เห็นด้วย
เห็นด้วย	เห็นด้วยอย่	เห็นด้วย	เห็นด้วย	เห็นด้วย	20.00	ไม่เห็นด้วย	เห็นด้วย	ไม่เห็นด้วย
เห็นด้วย	เห็นด้วย	เห็นด้วย	เห็นด้วย	เห็นด้วย	28.00	เห็นด้วย	เห็นด้วย	เห็นด้วย
เห็นด้วย	เห็นด้วย	เห็นด้วย	เห็นด้วย	เห็นด้วย	28.00	เห็นด้วย	เห็นด้วย	ไม่เห็นด้วย
เห็นด้วย	เห็นด้วย	เห็นด้วย	เห็นด้วย	เห็นด้วย	19.00	เห็นด้วย	เห็นด้วย	เห็นด้วย
เห็นด้วย	เห็นด้วย	เห็นด้วย	เห็นด้วย	เห็นด้วย	22.00	ไม่เห็นด้วย	เห็นด้วย	เห็นด้วย
เห็นด้วยอย่	เห็นด้วย	เห็นด้วย	เห็นด้วยอย่	เห็นด้วยอย่	22.00	เห็นด้วย	เห็นด้วยอย่	ไม่เห็นด้วย
เห็นด้วยอย่	เห็นด้วยอย่	เห็นด้วยอย่	เห็นด้วยอย่	เห็นด้วยอย่	24.00	ไม่เห็นด้วย	เห็นด้วย	ไม่เห็นด้วย
เห็นด้วย	เห็นด้วย	เห็นด้วย	เห็นด้วย	เห็นด้วย	25.00	ไม่เห็นด้วย	เห็นด้วย	เห็นด้วย
เห็นด้วย	เห็นด้วย	เห็นด้วย	เห็นด้วย	เห็นด้วย	23.00	ไม่เห็นด้วย	เห็นด้วย	ไม่เห็นด้วย
เห็นด้วยอย่	เห็นด้วยอย่	เห็นด้วยอย่	เห็นด้วย	เห็นด้วยอย่	24.00	เห็นด้วยอย่	เห็นด้วยอย่	เห็นด้วยอย่
เห็นด้วย	เห็นด้วย	เห็นด้วย	เห็นด้วย	เห็นด้วย	27.00	เห็นด้วย	เห็นด้วย	เห็นด้วย
เห็นด้วย	เห็นด้วย	เห็นด้วย	เห็นด้วย	เห็นด้วย	18.00	ไม่เห็นด้วย	เห็นด้วย	เห็นด้วย
เห็นด้วย	เห็นด้วย	เห็นด้วย	เห็นด้วย	เห็นด้วย	24.00	ไม่เห็นด้วย	ไม่เห็นด้วย	ไม่เห็นด้วย
เห็นด้วย	เห็นด้วย	เห็นด้วย	เห็นด้วยอย่	เห็นด้วย	21.00	ไม่เห็นด้วย	เห็นด้วย	ไม่เห็นด้วย
เห็นด้วย	เห็นด้วย	เห็นด้วย	เห็นด้วย	เห็นด้วย	19.00	ไม่เห็นด้วย	ไม่เห็นด้วย	ไม่เห็นด้วย
เห็นด้วย	เห็นด้วย	เห็นด้วย	เห็นด้วย	เห็นด้วย	21.00	ไม่เห็นด้วย	ไม่เห็นด้วย	ไม่เห็นด้วย
เห็นด้วย	เห็นด้วย	เห็นด้วย	เห็นด้วย	เห็นด้วย	23.00	เห็นด้วย	เห็นด้วย	เห็นด้วย
เห็นด้วย	เห็นด้วย	เห็นด้วย	เห็นด้วยอย่	เห็นด้วยอย่	21.00	ไม่เห็นด้วย	เห็นด้วย	ไม่เห็นด้วย
เห็นด้วย	ไม่เห็นด้วย	ไม่เห็นด้วย	เห็นด้วย	เห็นด้วย	20.00	เห็นด้วย	ไม่เห็นด้วย	ไม่เห็นด้วย
เห็นด้วยอย่	เห็นด้วยอย่	เห็นด้วย	เห็นด้วย	เห็นด้วยอย่	28.00	ไม่เห็นด้วย	เห็นด้วย	เห็นด้วย
เห็นด้วยอย่	เห็นด้วยอย่	เห็นด้วย	เห็นด้วยอย่	เห็นด้วยอย่	21.00	เห็นด้วยอย่	เห็นด้วย	เห็นด้วย

เห็นด้วย	เห็นด้วย	เห็นด้วย	เห็นด้วย	เห็นด้วย	21.00	เห็นด้วย	เห็นด้วย	เห็นด้วย
เห็นด้วย	เห็นด้วย	เห็นด้วย	เห็นด้วย	เห็นด้วย	25.00	ไม่เห็นด้วย	ไม่เห็นด้วย	ไม่เห็นด้วย
ไม่เห็นด้วย	เห็นด้วยอย่	เห็นด้วย	เห็นด้วยอย่	เห็นด้วย	26.00	ไม่เห็นด้วย	เห็นด้วย	เห็นด้วยอย่
เห็นด้วยอย่	เห็นด้วยอย่	เห็นด้วยอย่	เห็นด้วยอย่	เห็นด้วยอย่	21.00	ไม่เห็นด้วย	เห็นด้วยอย่	ไม่เห็นด้วย
เห็นด้วย	เห็นด้วย	เห็นด้วย	เห็นด้วย	เห็นด้วย	27.00	ไม่เห็นด้วย	เห็นด้วย	ไม่เห็นด้วย
เห็นด้วยอย่	เห็นด้วย	เห็นด้วย	เห็นด้วยอย่	เห็นด้วย	23.00	ไม่เห็นด้วย	เห็นด้วยอย่	ไม่เห็นด้วย
ไม่เห็นด้วย	เห็นด้วย	ไม่เห็นด้วย	เห็นด้วย	เห็นด้วย	27.00	ไม่เห็นด้วย	เห็นด้วย	ไม่เห็นด้วย
เห็นด้วย	เห็นด้วย	เห็นด้วย	เห็นด้วย	เห็นด้วย	24.00	ไม่เห็นด้วย	ไม่เห็นด้วย	ไม่เห็นด้วย
เห็นด้วยอย่	เห็นด้วยอย่	เห็นด้วย	เห็นด้วยอย่	เห็นด้วยอย่	24.00	เห็นด้วยอย่	เห็นด้วยอย่	เห็นด้วย
เห็นด้วย	ไม่เห็นด้วย	เห็นด้วย	เห็นด้วย	เห็นด้วยอย่	22.00	ไม่เห็นด้วย	เห็นด้วยอย่	เห็นด้วย
ไม่เห็นด้วย	เห็นด้วย	ไม่เห็นด้วย	เห็นด้วย	เห็นด้วย	19.00	ไม่เห็นด้วย	ไม่เห็นด้วย	ไม่เห็นด้วย
เห็นด้วย	ไม่เห็นด้วย	เห็นด้วย	เห็นด้วย	เห็นด้วยอย่	18.00	ไม่เห็นด้วย	เห็นด้วยอย่	เห็นด้วย
เห็นด้วยอย่	ไม่เห็นด้วย	ไม่เห็นด้วย	ไม่เห็นด้วย	เห็นด้วย	21.00	ไม่เห็นด้วย	ไม่เห็นด้วย	ไม่เห็นด้วย
เห็นด้วยอย่	เห็นด้วย	เห็นด้วย	เห็นด้วยอย่	เห็นด้วยอย่	24.00	ไม่เห็นด้วย	เห็นด้วย	เห็นด้วย
ไม่เห็นด้วย	ไม่เห็นด้วย	เห็นด้วย	เห็นด้วย	เห็นด้วย	19.00	ไม่เห็นด้วย	ไม่เห็นด้วย	เห็นด้วย
ไม่เห็นด้วย	เห็นด้วย	เห็นด้วย	เห็นด้วย	เห็นด้วย	28.00	ไม่เห็นด้วย	ไม่เห็นด้วย	เห็นด้วย
เห็นด้วย	เห็นด้วย	เห็นด้วย	เห็นด้วยอย่	เห็นด้วยอย่	24.00	ไม่เห็นด้วย	ไม่เห็นด้วย	ไม่เห็นด้วย
เห็นด้วย	เห็นด้วย	ไม่เห็นด้วย	ไม่เห็นด้วย	เห็นด้วย	28.00	ไม่เห็นด้วย	เห็นด้วย	เห็นด้วย
ไม่เห็นด้วย	เห็นด้วย	ไม่เห็นด้วย	เห็นด้วย	เห็นด้วย	22.00	ไม่เห็นด้วย	เห็นด้วย	ไม่เห็นด้วย
เห็นด้วยอย่	เห็นด้วย	เห็นด้วย	เห็นด้วย	เห็นด้วย	16.00	ไม่เห็นด้วย	ไม่เห็นด้วย	ไม่เห็นด้วย
เห็นด้วย	เห็นด้วย	เห็นด้วย	เห็นด้วยอย่	เห็นด้วยอย่	27.00	ไม่เห็นด้วย	เห็นด้วย	เห็นด้วย
ไม่เห็นด้วย	ไม่เห็นด้วย	ไม่เห็นด้วย	เห็นด้วย	เห็นด้วย	26.00	ไม่เห็นด้วย	เห็นด้วย	เห็นด้วย
เห็นด้วย	เห็นด้วย	เห็นด้วย	เห็นด้วย	เห็นด้วย	21.00	เห็นด้วย	เห็นด้วย	ไม่เห็นด้วย
เห็นด้วย	เห็นด้วย	ไม่เห็นด้วย	เห็นด้วย	เห็นด้วย	23.00	ไม่เห็นด้วย	เห็นด้วย	ไม่เห็นด้วย
เห็นด้วย	เห็นด้วย	ไม่เห็นด้วย	เห็นด้วยอย่	เห็นด้วย	25.00	ไม่เห็นด้วย	เห็นด้วย	เห็นด้วยอย่
เห็นด้วย	เห็นด้วยอย่	ไม่เห็นด้วย	เห็นด้วย	เห็นด้วยอย่	22.00	ไม่เห็นด้วย	เห็นด้วย	ไม่เห็นด้วย
เห็นด้วย	เห็นด้วย	เห็นด้วย	เห็นด้วย	เห็นด้วย	21.00	เห็นด้วย	เห็นด้วย	เห็นด้วย
เห็นด้วย	เห็นด้วย	เห็นด้วย	เห็นด้วย	เห็นด้วยอย่	21.00	ไม่เห็นด้วย	เห็นด้วย	เห็นด้วย
เห็นด้วย	เห็นด้วย	ไม่เห็นด้วย	เห็นด้วย	ไม่เห็นด้วย	11.00	ไม่เห็นด้วย	ไม่เห็นด้วย	ไม่เห็นด้วย
เห็นด้วย	เห็นด้วย	เห็นด้วย	เห็นด้วย	เห็นด้วย	22.00	เห็นด้วย	เห็นด้วย	เห็นด้วย
ไม่เห็นด้วย	เห็นด้วย	เห็นด้วย	เห็นด้วย	เห็นด้วย	23.00	ไม่เห็นด้วย	ไม่เห็นด้วย	เห็นด้วย
เห็นด้วย	ไม่เห็นด้วย	เห็นด้วย	เห็นด้วย	เห็นด้วย	24.00	เห็นด้วย	เห็นด้วย	เห็นด้วย
เห็นด้วย	เห็นด้วยอย่	เห็นด้วย	ไม่เห็นด้วย	เห็นด้วยอย่	16.00	เห็นด้วย	ไม่เห็นด้วย	ไม่เห็นด้วย
ไม่เห็นด้วย	ไม่เห็นด้วย	เห็นด้วย	เห็นด้วย	เห็นด้วย	21.00	ไม่เห็นด้วย	ไม่เห็นด้วย	เห็นด้วย
ไม่เห็นด้วย	เห็นด้วย	เห็นด้วย	เห็นด้วย	ไม่เห็นด้วย	23.00	ไม่เห็นด้วย	เห็นด้วย	ไม่เห็นด้วย
ไม่เห็นด้วย	เห็นด้วยอย่	เห็นด้วย	เห็นด้วย	เห็นด้วย	20.00	ไม่เห็นด้วย	เห็นด้วย	เห็นด้วย
เห็นด้วยอย่	เห็นด้วย	เห็นด้วย	เห็นด้วยอย่	เห็นด้วย	19.00	ไม่เห็นด้วย	เห็นด้วย	เห็นด้วย
เห็นด้วย	เห็นด้วย	เห็นด้วย	เห็นด้วยอย่	เห็นด้วย	27.00	เห็นด้วย	ไม่เห็นด้วย	ไม่เห็นด้วย
เห็นด้วย	เห็นด้วย	เห็นด้วย	เห็นด้วย	เห็นด้วย	19.00	เห็นด้วย	เห็นด้วย	ไม่เห็นด้วย
เห็นด้วยอย่	เห็นด้วยอย่	เห็นด้วย	เห็นด้วย	เห็นด้วย	20.00	ไม่เห็นด้วย	ไม่เห็นด้วย	ไม่เห็นด้วย
ไม่เห็นด้วย	เห็นด้วย	เห็นด้วย	เห็นด้วย	เห็นด้วย	21.00	ไม่เห็นด้วย	ไม่เห็นด้วย	ไม่เห็นด้วย
เห็นด้วย	เห็นด้วย	เห็นด้วย	เห็นด้วย	เห็นด้วย	26.00	ไม่เห็นด้วย	เห็นด้วย	ไม่เห็นด้วย
เห็นด้วย	ไม่เห็นด้วย	ไม่เห็นด้วย	ไม่เห็นด้วย	ไม่เห็นด้วย	21.00	ไม่เห็นด้วย	เห็นด้วย	ไม่เห็นด้วย
เห็นด้วย	เห็นด้วย	เห็นด้วย	เห็นด้วย	เห็นด้วยอย่	21.00	ไม่เห็นด้วย	ไม่เห็นด้วย	ไม่เห็นด้วย
เห็นด้วยอย่	เห็นด้วยอย่	เห็นด้วย	เห็นด้วยอย่	เห็นด้วยอย่	21.00	ไม่เห็นด้วย	ไม่เห็นด้วย	เห็นด้วย
ไม่เห็นด้วย	เห็นด้วย	ไม่เห็นด้วย	เห็นด้วย	เห็นด้วย	23.00	ไม่เห็นด้วย	เห็นด้วย	เห็นด้วย
เห็นด้วย	เห็นด้วย	เห็นด้วย	เห็นด้วย	เห็นด้วย	21.00	เห็นด้วย	เห็นด้วย	เห็นด้วย
ไม่เห็นด้วย	ไม่เห็นด้วย	เห็นด้วย	เห็นด้วย	เห็นด้วยอย่	24.00	ไม่เห็นด้วย	เห็นด้วย	เห็นด้วย
เห็นด้วย	ไม่เห็นด้วย	ไม่เห็นด้วย	เห็นด้วย	เห็นด้วย	22.00	ไม่เห็นด้วย	เห็นด้วย	ไม่เห็นด้วย
เห็นด้วย	เห็นด้วยอย่	เห็นด้วย	เห็นด้วย	เห็นด้วย	19.00	ไม่เห็นด้วย	เห็นด้วย	ไม่เห็นด้วย
เห็นด้วย	เห็นด้วย	เห็นด้วย	เห็นด้วย	เห็นด้วย	21.00	ไม่เห็นด้วย	เห็นด้วย	เห็นด้วย

เห็นด้วย	เห็นด้วย	เห็นด้วย	เห็นด้วย	เห็นด้วย	21.00	ไม่เห็นด้วย	ไม่เห็นด้วย	เห็นด้วย
ไม่เห็นด้วย	เห็นด้วย	เห็นด้วย	เห็นด้วย	เห็นด้วย	21.00	เห็นด้วย	เห็นด้วย	เห็นด้วย
ไม่เห็นด้วย	ไม่เห็นด้วย	ไม่เห็นด้วย	ไม่เห็นด้วย	ไม่เห็นด้วย	20.00	ไม่เห็นด้วย	ไม่เห็นด้วย	ไม่เห็นด้วย
เห็นด้วย	เห็นด้วย	เห็นด้วย	เห็นด้วย	เห็นด้วย	21.00	ไม่เห็นด้วย	ไม่เห็นด้วย	ไม่เห็นด้วย
เห็นด้วย	เห็นด้วย	เห็นด้วย	เห็นด้วย	เห็นด้วย	20.00	ไม่เห็นด้วย	เห็นด้วย	ไม่เห็นด้วย
เห็นด้วย	เห็นด้วย	เห็นด้วย	เห็นด้วย	เห็นด้วย	24.00	ไม่เห็นด้วย	เห็นด้วย	เห็นด้วย
เห็นด้วย	เห็นด้วย	เห็นด้วย	เห็นด้วย	เห็นด้วย	21.00	เห็นด้วย	เห็นด้วย	เห็นด้วย
เห็นด้วย	เห็นด้วย	เห็นด้วย	เห็นด้วย	เห็นด้วย	20.00	ไม่เห็นด้วย	เห็นด้วย	ไม่เห็นด้วย
เห็นด้วย	เห็นด้วย	เห็นด้วย	เห็นด้วยอย่	เห็นด้วย	27.00	ไม่เห็นด้วย	เห็นด้วย	เห็นด้วย
เห็นด้วยอย่	เห็นด้วย	เห็นด้วยอย่	เห็นด้วยอย่	เห็นด้วยอย่	25.00	ไม่เห็นด้วยอ	ไม่เห็นด้วยอ	ไม่เห็นด้วยอ
เห็นด้วย	เห็นด้วย	เห็นด้วย	เห็นด้วย	เห็นด้วย	22.00	ไม่เห็นด้วย	ไม่เห็นด้วย	ไม่เห็นด้วย
ไม่เห็นด้วย	เห็นด้วย	ไม่เห็นด้วย	เห็นด้วย	เห็นด้วยอย่	22.00	ไม่เห็นด้วย	ไม่เห็นด้วย	เห็นด้วย
เห็นด้วย	เห็นด้วย	เห็นด้วย	เห็นด้วย	เห็นด้วย	21.00	ไม่เห็นด้วย	ไม่เห็นด้วย	ไม่เห็นด้วย
เห็นด้วย	เห็นด้วยอย่	เห็นด้วยอย่	เห็นด้วย	เห็นด้วย	19.00	ไม่เห็นด้วยอ	ไม่เห็นด้วย	ไม่เห็นด้วยอ
เห็นด้วย	เห็นด้วย	เห็นด้วย	เห็นด้วยอย่	เห็นด้วย	19.00	ไม่เห็นด้วย	เห็นด้วย	เห็นด้วย
เห็นด้วย	เห็นด้วย	เห็นด้วย	เห็นด้วยอย่	เห็นด้วย	21.00	ไม่เห็นด้วย	เห็นด้วยอย่	เห็นด้วย
ไม่เห็นด้วย	เห็นด้วย	เห็นด้วย	เห็นด้วย	เห็นด้วย	28.00	ไม่เห็นด้วย	ไม่เห็นด้วย	ไม่เห็นด้วย
เห็นด้วย	เห็นด้วย	เห็นด้วย	เห็นด้วย	เห็นด้วย	27.00	ไม่เห็นด้วย	เห็นด้วย	เห็นด้วย
เห็นด้วย	เห็นด้วย	เห็นด้วย	เห็นด้วยอย่	เห็นด้วยอย่	21.00	ไม่เห็นด้วย	ไม่เห็นด้วย	ไม่เห็นด้วย
เห็นด้วย	เห็นด้วย	เห็นด้วย	เห็นด้วย	เห็นด้วยอย่	19.00	ไม่เห็นด้วย	เห็นด้วย	เห็นด้วย
เห็นด้วย	เห็นด้วย	เห็นด้วย	เห็นด้วยอย่	เห็นด้วยอย่	21.00	เห็นด้วย	เห็นด้วยอย่	เห็นด้วยอย่
ไม่เห็นด้วย	เห็นด้วยอย่	ไม่เห็นด้วย	เห็นด้วย	เห็นด้วย	23.00	ไม่เห็นด้วย	เห็นด้วย	ไม่เห็นด้วย
เห็นด้วย	เห็นด้วย	ไม่เห็นด้วย	เห็นด้วย	เห็นด้วย	21.00	ไม่เห็นด้วย	ไม่เห็นด้วย	ไม่เห็นด้วย
เห็นด้วยอย่	เห็นด้วย	ไม่เห็นด้วย	เห็นด้วย	เห็นด้วย	17.00	ไม่เห็นด้วย	เห็นด้วยอย่	เห็นด้วย
เห็นด้วย	เห็นด้วย	เห็นด้วย	เห็นด้วย	เห็นด้วย	24.00	ไม่เห็นด้วย	เห็นด้วย	เห็นด้วย
เห็นด้วย	เห็นด้วย	เห็นด้วย	เห็นด้วยอย่	เห็นด้วย	28.00	ไม่เห็นด้วย	ไม่เห็นด้วย	ไม่เห็นด้วย
เห็นด้วย	เห็นด้วยอย่	เห็นด้วย	เห็นด้วยอย่	เห็นด้วยอย่	19.00	ไม่เห็นด้วยอ	เห็นด้วย	ไม่เห็นด้วย
เห็นด้วย	เห็นด้วย	เห็นด้วย	เห็นด้วย	เห็นด้วย	23.00	เห็นด้วย	เห็นด้วย	เห็นด้วย
เห็นด้วย	เห็นด้วย	ไม่เห็นด้วย	เห็นด้วย	เห็นด้วย	21.00	ไม่เห็นด้วย	เห็นด้วย	ไม่เห็นด้วย
เห็นด้วย	เห็นด้วย	เห็นด้วย	เห็นด้วยอย่	เห็นด้วยอย่	22.00	ไม่เห็นด้วย	ไม่เห็นด้วย	ไม่เห็นด้วย
ไม่เห็นด้วย	ไม่เห็นด้วย	เห็นด้วย	เห็นด้วย	เห็นด้วยอย่	24.00	ไม่เห็นด้วย	เห็นด้วย	ไม่เห็นด้วย
เห็นด้วยอย่	เห็นด้วย	ไม่เห็นด้วย	เห็นด้วย	เห็นด้วย	21.00	ไม่เห็นด้วย	ไม่เห็นด้วย	ไม่เห็นด้วย
เห็นด้วย	เห็นด้วย	เห็นด้วย	เห็นด้วย	เห็นด้วยอย่	19.00	ไม่เห็นด้วย	เห็นด้วย	ไม่เห็นด้วย
เห็นด้วย	ไม่เห็นด้วย	ไม่เห็นด้วย	เห็นด้วย	เห็นด้วย	22.00	ไม่เห็นด้วย	เห็นด้วย	ไม่เห็นด้วย
เห็นด้วย	เห็นด้วย	เห็นด้วย	เห็นด้วย	เห็นด้วย	26.00	ไม่เห็นด้วย	ไม่เห็นด้วย	ไม่เห็นด้วย
เห็นด้วย	ไม่เห็นด้วย	ไม่เห็นด้วย	เห็นด้วย	เห็นด้วย	22.00	เห็นด้วย	ไม่เห็นด้วย	เห็นด้วย
เห็นด้วย	เห็นด้วย	เห็นด้วย	เห็นด้วย	เห็นด้วย	11.00	เห็นด้วย	เห็นด้วย	เห็นด้วย
เห็นด้วย	เห็นด้วย	เห็นด้วย	เห็นด้วย	เห็นด้วย	23.00	ไม่เห็นด้วย	เห็นด้วย	ไม่เห็นด้วย
เห็นด้วยอย่	เห็นด้วยอย่	เห็นด้วย	เห็นด้วย	เห็นด้วยอย่	21.00	ไม่เห็นด้วยอ	ไม่เห็นด้วยอ	ไม่เห็นด้วย
เห็นด้วยอย่	เห็นด้วย	เห็นด้วย	เห็นด้วยอย่	เห็นด้วยอย่	25.00	ไม่เห็นด้วยอ	เห็นด้วย	เห็นด้วย
เห็นด้วย	เห็นด้วย	เห็นด้วย	เห็นด้วย	เห็นด้วย	25.00	ไม่เห็นด้วย	ไม่เห็นด้วย	ไม่เห็นด้วย
เห็นด้วย	เห็นด้วย	เห็นด้วย	ไม่เห็นด้วย	ไม่เห็นด้วย	25.00	ไม่เห็นด้วย	ไม่เห็นด้วย	เห็นด้วยอย่
เห็นด้วยอย่	เห็นด้วยอย่	เห็นด้วยอย่	เห็นด้วยอย่	เห็นด้วยอย่	21.00	ไม่เห็นด้วย	เห็นด้วยอย่	ไม่เห็นด้วย
เห็นด้วย	เห็นด้วย	เห็นด้วย	เห็นด้วย	เห็นด้วย	21.00	ไม่เห็นด้วย	ไม่เห็นด้วย	ไม่เห็นด้วย
เห็นด้วยอย่	ไม่เห็นด้วยอ	ไม่เห็นด้วยอ	เห็นด้วยอย่	เห็นด้วยอย่	21.00	เห็นด้วยอย่	ไม่เห็นด้วยอ	ไม่เห็นด้วยอ
เห็นด้วย	เห็นด้วยอย่	เห็นด้วย	เห็นด้วยอย่	เห็นด้วย	19.00	ไม่เห็นด้วย	เห็นด้วย	ไม่เห็นด้วย
เห็นด้วยอย่	เห็นด้วย	เห็นด้วย	เห็นด้วย	เห็นด้วย	26.00	ไม่เห็นด้วย	เห็นด้วย	ไม่เห็นด้วย
เห็นด้วย	เห็นด้วย	ไม่เห็นด้วย	เห็นด้วย	เห็นด้วย	19.00	ไม่เห็นด้วย	เห็นด้วย	ไม่เห็นด้วย
เห็นด้วยอย่	เห็นด้วย	เห็นด้วยอย่	เห็นด้วยอย่	เห็นด้วยอย่	21.00	เห็นด้วยอย่	เห็นด้วย	เห็นด้วย
เห็นด้วย	เห็นด้วย	เห็นด้วย	เห็นด้วย	เห็นด้วย	17.00	ไม่เห็นด้วย	เห็นด้วย	ไม่เห็นด้วย
เห็นด้วย	เห็นด้วย	ไม่เห็นด้วย	เห็นด้วย	เห็นด้วย	20.00	ไม่เห็นด้วย	ไม่เห็นด้วย	เห็นด้วย

เห็นด้วย	เห็นด้วย	เห็นด้วย	เห็นด้วย	เห็นด้วย	25.00	ไม่เห็นด้วย	ไม่เห็นด้วย	ไม่เห็นด้วย
เห็นด้วยอย่	เห็นด้วย	เห็นด้วย	เห็นด้วย	เห็นด้วย	21.00	ไม่เห็นด้วย	ไม่เห็นด้วย	ไม่เห็นด้วย
เห็นด้วยอย่	เห็นด้วย	เห็นด้วย	เห็นด้วยอย่	เห็นด้วยอย่	24.00	ไม่เห็นด้วย	เห็นด้วย	เห็นด้วย
เห็นด้วย	เห็นด้วย	เห็นด้วย	เห็นด้วยอย่	เห็นด้วย	18.00	เห็นด้วยอย่	เห็นด้วย	เห็นด้วย
เห็นด้วย	เห็นด้วย	ไม่เห็นด้วย	เห็นด้วย	เห็นด้วย	24.00	ไม่เห็นด้วย	เห็นด้วย	ไม่เห็นด้วย
เห็นด้วย	เห็นด้วยอย่	เห็นด้วย	เห็นด้วย	เห็นด้วยอย่	19.00	ไม่เห็นด้วย	เห็นด้วย	ไม่เห็นด้วย
เห็นด้วย	เห็นด้วย	เห็นด้วย	เห็นด้วย	เห็นด้วย	22.00	เห็นด้วย	เห็นด้วย	เห็นด้วย
เห็นด้วยอย่	เห็นด้วยอย่	เห็นด้วยอย่	เห็นด้วยอย่	เห็นด้วยอย่	20.00	ไม่เห็นด้วย	ไม่เห็นด้วย	ไม่เห็นด้วย
เห็นด้วย	เห็นด้วย	เห็นด้วย	เห็นด้วย	เห็นด้วย	28.00	เห็นด้วย	เห็นด้วย	เห็นด้วย
เห็นด้วยอย่	เห็นด้วยอย่	เห็นด้วยอย่	เห็นด้วยอย่	เห็นด้วยอย่	19.00	ไม่เห็นด้วย	เห็นด้วยอย่	เห็นด้วยอย่
เห็นด้วย	ไม่เห็นด้วย	ไม่เห็นด้วย	ไม่เห็นด้วย	ไม่เห็นด้วย	19.00	ไม่เห็นด้วย	ไม่เห็นด้วย	ไม่เห็นด้วย
เห็นด้วยอย่	เห็นด้วยอย่	เห็นด้วยอย่	เห็นด้วย	เห็นด้วย	26.00	เห็นด้วยอย่	เห็นด้วย	เห็นด้วยอย่
เห็นด้วย	เห็นด้วยอย่	เห็นด้วยอย่	เห็นด้วยอย่	เห็นด้วย	26.00	เห็นด้วยอย่	เห็นด้วย	เห็นด้วยอย่
เห็นด้วย	เห็นด้วย	เห็นด้วย	เห็นด้วย	เห็นด้วยอย่	19.00	เห็นด้วย	เห็นด้วย	เห็นด้วย
เห็นด้วย	เห็นด้วย	ไม่เห็นด้วย	เห็นด้วย	เห็นด้วย	19.00	เห็นด้วย	ไม่เห็นด้วย	เห็นด้วย
เห็นด้วยอย่	เห็นด้วย	เห็นด้วย	เห็นด้วย	เห็นด้วย	20.00	ไม่เห็นด้วย	เห็นด้วย	เห็นด้วย
เห็นด้วยอย่	เห็นด้วย	เห็นด้วย	เห็นด้วยอย่	เห็นด้วย	21.00	ไม่เห็นด้วย	ไม่เห็นด้วย	เห็นด้วย
เห็นด้วย	เห็นด้วย	เห็นด้วย	เห็นด้วยอย่	เห็นด้วย	24.00	เห็นด้วย	เห็นด้วย	เห็นด้วย
เห็นด้วย	เห็นด้วย	เห็นด้วยอย่	เห็นด้วย	เห็นด้วยอย่	24.00	เห็นด้วยอย่	เห็นด้วย	เห็นด้วยอย่
เห็นด้วยอย่	เห็นด้วย	เห็นด้วย	เห็นด้วยอย่	เห็นด้วยอย่	21.00	ไม่เห็นด้วย	เห็นด้วยอย่	เห็นด้วย
เห็นด้วยอย่	เห็นด้วย	เห็นด้วย	เห็นด้วยอย่	เห็นด้วยอย่	21.00	เห็นด้วยอย่	ไม่เห็นด้วย	ไม่เห็นด้วย
เห็นด้วยอย่	เห็นด้วย	เห็นด้วย	เห็นด้วยอย่	เห็นด้วยอย่	19.00	ไม่เห็นด้วย	เห็นด้วย	เห็นด้วย
ไม่เห็นด้วย	เห็นด้วย	ไม่เห็นด้วย	ไม่เห็นด้วย	ไม่เห็นด้วย	24.00	เห็นด้วยอย่	เห็นด้วย	ไม่เห็นด้วย
เห็นด้วย	เห็นด้วย	เห็นด้วย	เห็นด้วย	เห็นด้วย	27.00	ไม่เห็นด้วย	เห็นด้วย	ไม่เห็นด้วย
เห็นด้วย	เห็นด้วย	เห็นด้วย	เห็นด้วย	เห็นด้วย	21.00	ไม่เห็นด้วย	เห็นด้วย	เห็นด้วย
เห็นด้วย	เห็นด้วย	เห็นด้วย	เห็นด้วย	เห็นด้วย	19.00	ไม่เห็นด้วย	ไม่เห็นด้วย	ไม่เห็นด้วย
เห็นด้วยอย่	ไม่เห็นด้วย	ไม่เห็นด้วย	ไม่เห็นด้วย	ไม่เห็นด้วย	23.00	ไม่เห็นด้วย	ไม่เห็นด้วย	ไม่เห็นด้วย
ไม่เห็นด้วย	เห็นด้วย	เห็นด้วย	เห็นด้วย	เห็นด้วย	28.00	ไม่เห็นด้วย	ไม่เห็นด้วย	ไม่เห็นด้วย
เห็นด้วย	เห็นด้วยอย่	เห็นด้วยอย่	เห็นด้วย	เห็นด้วยอย่	28.00	เห็นด้วย	เห็นด้วย	เห็นด้วย
เห็นด้วย	เห็นด้วยอย่	เห็นด้วยอย่	เห็นด้วย	เห็นด้วยอย่	22.00	เห็นด้วย	เห็นด้วยอย่	เห็นด้วย
ไม่เห็นด้วย	เห็นด้วย	เห็นด้วย	ไม่เห็นด้วย	เห็นด้วย	19.00	เห็นด้วย	ไม่เห็นด้วย	เห็นด้วย
เห็นด้วยอย่	เห็นด้วยอย่	เห็นด้วยอย่	เห็นด้วยอย่	เห็นด้วย	20.00	ไม่เห็นด้วย	ไม่เห็นด้วย	ไม่เห็นด้วย
เห็นด้วยอย่	เห็นด้วยอย่	เห็นด้วยอย่	เห็นด้วยอย่	เห็นด้วยอย่	18.00	เห็นด้วยอย่	เห็นด้วยอย่	เห็นด้วยอย่
เห็นด้วย	เห็นด้วย	เห็นด้วย	เห็นด้วย	เห็นด้วย	21.00	ไม่เห็นด้วย	ไม่เห็นด้วย	ไม่เห็นด้วย
เห็นด้วย	เห็นด้วยอย่	เห็นด้วย	เห็นด้วยอย่	เห็นด้วย	21.00	เห็นด้วยอย่	เห็นด้วยอย่	เห็นด้วยอย่
เห็นด้วยอย่	เห็นด้วย	เห็นด้วย	เห็นด้วยอย่	เห็นด้วยอย่	21.00	ไม่เห็นด้วย	เห็นด้วย	เห็นด้วย
เห็นด้วย	เห็นด้วยอย่	เห็นด้วย	เห็นด้วยอย่	เห็นด้วยอย่	26.00	ไม่เห็นด้วย	ไม่เห็นด้วย	เห็นด้วย
เห็นด้วยอย่	เห็นด้วย	เห็นด้วย	เห็นด้วย	เห็นด้วย	24.00	เห็นด้วยอย่	เห็นด้วยอย่	เห็นด้วย
เห็นด้วยอย่	เห็นด้วย	เห็นด้วยอย่	เห็นด้วยอย่	เห็นด้วยอย่	21.00	เห็นด้วยอย่	เห็นด้วยอย่	เห็นด้วยอย่
เห็นด้วยอย่	เห็นด้วยอย่	เห็นด้วย	เห็นด้วยอย่	เห็นด้วยอย่	26.00	ไม่เห็นด้วย	ไม่เห็นด้วย	ไม่เห็นด้วย
เห็นด้วย	เห็นด้วยอย่	เห็นด้วยอย่	เห็นด้วย	เห็นด้วยอย่	22.00	เห็นด้วยอย่	เห็นด้วยอย่	เห็นด้วย
เห็นด้วย	เห็นด้วย	ไม่เห็นด้วย	เห็นด้วย	เห็นด้วยอย่	27.00	เห็นด้วย	ไม่เห็นด้วย	เห็นด้วย
เห็นด้วย	เห็นด้วย	เห็นด้วย	เห็นด้วยอย่	เห็นด้วยอย่	21.00	ไม่เห็นด้วย	เห็นด้วย	ไม่เห็นด้วย
เห็นด้วยอย่	เห็นด้วยอย่	เห็นด้วย	เห็นด้วยอย่	เห็นด้วยอย่	23.00	ไม่เห็นด้วย	ไม่เห็นด้วย	เห็นด้วย
ไม่เห็นด้วย	ไม่เห็นด้วย	เห็นด้วย	เห็นด้วย	เห็นด้วยอย่	18.00	ไม่เห็นด้วย	เห็นด้วย	เห็นด้วย
เห็นด้วยอย่	เห็นด้วย	ไม่เห็นด้วย	เห็นด้วยอย่	ไม่เห็นด้วย	20.00	ไม่เห็นด้วย	เห็นด้วย	เห็นด้วย
เห็นด้วย	เห็นด้วยอย่	เห็นด้วยอย่	เห็นด้วย	เห็นด้วยอย่	19.00	เห็นด้วยอย่	เห็นด้วย	เห็นด้วยอย่
เห็นด้วยอย่	เห็นด้วย	เห็นด้วยอย่	เห็นด้วยอย่	เห็นด้วยอย่	22.00	เห็นด้วยอย่	เห็นด้วยอย่	เห็นด้วย
เห็นด้วย	เห็นด้วยอย่	เห็นด้วย	เห็นด้วยอย่	เห็นด้วยอย่	17.00	เห็นด้วยอย่	เห็นด้วยอย่	เห็นด้วยอย่
เห็นด้วยอย่	เห็นด้วยอย่	เห็นด้วยอย่	เห็นด้วย	เห็นด้วย	21.00	เห็นด้วยอย่	เห็นด้วยอย่	เห็นด้วยอย่
เห็นด้วยอย่	เห็นด้วย	เห็นด้วย	เห็นด้วยอย่	เห็นด้วยอย่	20.00	เห็นด้วยอย่	เห็นด้วยอย่	เห็นด้วย

เห็นด้วย	เห็นด้วย	ไม่เห็นด้วย	ไม่เห็นด้วย	เห็นด้วย	20.00	ไม่เห็นด้วย	เห็นด้วย	เห็นด้วย
เห็นด้วย	เห็นด้วย	เห็นด้วย	เห็นด้วย	เห็นด้วย	19.00	ไม่เห็นด้วย	เห็นด้วย	ไม่เห็นด้วย
เห็นด้วย	ไม่เห็นด้วย	ไม่เห็นด้วย	เห็นด้วย	เห็นด้วย	21.00	ไม่เห็นด้วย	ไม่เห็นด้วย	ไม่เห็นด้วย
ไม่เห็นด้วย	เห็นด้วย	ไม่เห็นด้วย	เห็นด้วย	เห็นด้วยอย่	22.00	ไม่เห็นด้วย	ไม่เห็นด้วย	เห็นด้วย
เห็นด้วยอย่	เห็นด้วยอย่	เห็นด้วยอย่	เห็นด้วยอย่	เห็นด้วยอย่	23.00	ไม่เห็นด้วยอ	ไม่เห็นด้วยอ	ไม่เห็นด้วยอ
เห็นด้วยอย่	เห็นด้วย	ไม่เห็นด้วย	เห็นด้วยอย่	เห็นด้วยอย่	21.00	ไม่เห็นด้วย	เห็นด้วย	ไม่เห็นด้วย
เห็นด้วย	เห็นด้วย	เห็นด้วย	เห็นด้วย	เห็นด้วย	21.00	ไม่เห็นด้วย	เห็นด้วย	เห็นด้วย
เห็นด้วยอย่	เห็นด้วยอย่	เห็นด้วย	เห็นด้วยอย่	เห็นด้วยอย่	21.00	ไม่เห็นด้วย	ไม่เห็นด้วย	ไม่เห็นด้วย
เห็นด้วยอย่	เห็นด้วยอย่	เห็นด้วย	เห็นด้วย	เห็นด้วยอย่	19.00	เห็นด้วยอย่	ไม่เห็นด้วย	เห็นด้วย
เห็นด้วย	เห็นด้วย	เห็นด้วย	เห็นด้วย	เห็นด้วย	21.00	ไม่เห็นด้วย	เห็นด้วย	ไม่เห็นด้วย
เห็นด้วย	ไม่เห็นด้วย	ไม่เห็นด้วย	เห็นด้วย	เห็นด้วย	21.00	ไม่เห็นด้วย	ไม่เห็นด้วย	ไม่เห็นด้วย
ไม่เห็นด้วย	เห็นด้วย	เห็นด้วย	เห็นด้วย	เห็นด้วย	21.00	ไม่เห็นด้วย	เห็นด้วย	ไม่เห็นด้วย
ไม่เห็นด้วยอ	ไม่เห็นด้วย	ไม่เห็นด้วย	เห็นด้วย	ไม่เห็นด้วยอ	25.00	ไม่เห็นด้วยอ	เห็นด้วย	เห็นด้วย
เห็นด้วย	เห็นด้วย	เห็นด้วย	เห็นด้วย	เห็นด้วย	27.00	ไม่เห็นด้วย	ไม่เห็นด้วย	เห็นด้วย
เห็นด้วยอย่	เห็นด้วย	เห็นด้วย	เห็นด้วย	เห็นด้วยอย่	25.00	ไม่เห็นด้วย	เห็นด้วยอย่	ไม่เห็นด้วย
เห็นด้วย	เห็นด้วย	เห็นด้วย	เห็นด้วย	เห็นด้วย	21.00	เห็นด้วย	เห็นด้วย	ไม่เห็นด้วย
เห็นด้วยอย่	เห็นด้วย	เห็นด้วย	เห็นด้วยอย่	เห็นด้วย	27.00	ไม่เห็นด้วย	เห็นด้วย	ไม่เห็นด้วยอ
เห็นด้วย	ไม่เห็นด้วย	ไม่เห็นด้วย	เห็นด้วย	เห็นด้วย	18.00	ไม่เห็นด้วย	ไม่เห็นด้วย	ไม่เห็นด้วย
เห็นด้วยอย่	เห็นด้วย	เห็นด้วย	เห็นด้วยอย่	เห็นด้วยอย่	20.00	ไม่เห็นด้วย	ไม่เห็นด้วย	ไม่เห็นด้วย
เห็นด้วย	เห็นด้วยอย่	เห็นด้วย	เห็นด้วย	เห็นด้วย	20.00	ไม่เห็นด้วย	เห็นด้วย	เห็นด้วย
เห็นด้วยอย่	เห็นด้วย	เห็นด้วย	เห็นด้วย	เห็นด้วย	23.00	ไม่เห็นด้วย	เห็นด้วย	เห็นด้วย
เห็นด้วย	เห็นด้วย	เห็นด้วย	เห็นด้วย	เห็นด้วย	25.00	ไม่เห็นด้วย	เห็นด้วยอย่	เห็นด้วยอย่
เห็นด้วย	ไม่เห็นด้วย	เห็นด้วย	เห็นด้วย	เห็นด้วยอย่	11.00	ไม่เห็นด้วย	เห็นด้วย	ไม่เห็นด้วย
เห็นด้วย	ไม่เห็นด้วย	ไม่เห็นด้วย	เห็นด้วย	เห็นด้วย	23.00	ไม่เห็นด้วย	ไม่เห็นด้วย	ไม่เห็นด้วย
เห็นด้วยอย่	เห็นด้วย	เห็นด้วย	เห็นด้วยอย่	เห็นด้วย	21.00	ไม่เห็นด้วย	ไม่เห็นด้วย	ไม่เห็นด้วย
เห็นด้วยอย่	เห็นด้วย	เห็นด้วย	เห็นด้วย	เห็นด้วย	21.00	ไม่เห็นด้วย	เห็นด้วยอย่	เห็นด้วยอย่
เห็นด้วย	เห็นด้วย	เห็นด้วย	เห็นด้วย	เห็นด้วย	21.00	เห็นด้วยอย่	เห็นด้วยอย่	เห็นด้วย
เห็นด้วยอย่	เห็นด้วยอย่	เห็นด้วยอย่	เห็นด้วยอย่	เห็นด้วยอย่	21.00	ไม่เห็นด้วย	เห็นด้วย	ไม่เห็นด้วย
ไม่เห็นด้วย	ไม่เห็นด้วย	ไม่เห็นด้วย	เห็นด้วย	เห็นด้วย	21.00	เห็นด้วย	เห็นด้วยอย่	ไม่เห็นด้วย
เห็นด้วย	เห็นด้วย	ไม่เห็นด้วย	เห็นด้วย	เห็นด้วย	21.00	เห็นด้วย	ไม่เห็นด้วย	ไม่เห็นด้วย
เห็นด้วย	เห็นด้วย	เห็นด้วย	เห็นด้วย	เห็นด้วย	25.00	ไม่เห็นด้วย	เห็นด้วย	ไม่เห็นด้วย
เห็นด้วยอย่	เห็นด้วยอย่	ไม่เห็นด้วย	เห็นด้วยอย่	เห็นด้วยอย่	24.00	ไม่เห็นด้วย	เห็นด้วยอย่	เห็นด้วย
เห็นด้วย	เห็นด้วย	เห็นด้วย	เห็นด้วย	เห็นด้วย	21.00	ไม่เห็นด้วย	เห็นด้วย	ไม่เห็นด้วย
ไม่เห็นด้วย	เห็นด้วย	ไม่เห็นด้วย	เห็นด้วย	เห็นด้วยอย่	21.00	ไม่เห็นด้วย	ไม่เห็นด้วย	เห็นด้วย
เห็นด้วยอย่	เห็นด้วยอย่	เห็นด้วยอย่	เห็นด้วยอย่	เห็นด้วยอย่	25.00	ไม่เห็นด้วยอ	ไม่เห็นด้วยอ	ไม่เห็นด้วยอ
เห็นด้วยอย่	เห็นด้วย	ไม่เห็นด้วย	เห็นด้วยอย่	เห็นด้วยอย่	23.00	ไม่เห็นด้วย	เห็นด้วย	ไม่เห็นด้วย
เห็นด้วย	เห็นด้วย	เห็นด้วย	เห็นด้วย	เห็นด้วย	24.00	ไม่เห็นด้วย	เห็นด้วย	เห็นด้วย
เห็นด้วยอย่	เห็นด้วยอย่	เห็นด้วย	เห็นด้วยอย่	เห็นด้วยอย่	21.00	ไม่เห็นด้วย	ไม่เห็นด้วย	ไม่เห็นด้วย
เห็นด้วยอย่	เห็นด้วย	เห็นด้วย	เห็นด้วยอย่	เห็นด้วยอย่	28.00	ไม่เห็นด้วย	เห็นด้วย	ไม่เห็นด้วย
เห็นด้วย	เห็นด้วย	เห็นด้วย	เห็นด้วย	เห็นด้วย	23.00	ไม่เห็นด้วย	เห็นด้วย	ไม่เห็นด้วย
เห็นด้วย	ไม่เห็นด้วย	ไม่เห็นด้วย	เห็นด้วย	เห็นด้วย	19.00	ไม่เห็นด้วย	ไม่เห็นด้วย	ไม่เห็นด้วย
ไม่เห็นด้วย	เห็นด้วย	เห็นด้วย	เห็นด้วย	เห็นด้วย	21.00	ไม่เห็นด้วย	เห็นด้วย	ไม่เห็นด้วย
ไม่เห็นด้วยอ	ไม่เห็นด้วย	ไม่เห็นด้วย	เห็นด้วย	ไม่เห็นด้วยอ	21.00	ไม่เห็นด้วยอ	เห็นด้วย	เห็นด้วย
เห็นด้วย	เห็นด้วย	เห็นด้วย	เห็นด้วย	เห็นด้วย	24.00	ไม่เห็นด้วย	ไม่เห็นด้วย	เห็นด้วย
เห็นด้วยอย่	เห็นด้วย	เห็นด้วย	เห็นด้วย	เห็นด้วยอย่	21.00	ไม่เห็นด้วย	เห็นด้วยอย่	ไม่เห็นด้วย
เห็นด้วย	เห็นด้วย	เห็นด้วย	เห็นด้วย	เห็นด้วย	21.00	เห็นด้วย	เห็นด้วย	ไม่เห็นด้วย
เห็นด้วยอย่	เห็นด้วย	เห็นด้วย	เห็นด้วยอย่	เห็นด้วย	21.00	ไม่เห็นด้วย	เห็นด้วย	ไม่เห็นด้วยอ
เห็นด้วย	ไม่เห็นด้วย	ไม่เห็นด้วย	เห็นด้วย	เห็นด้วย	20.00	ไม่เห็นด้วย	ไม่เห็นด้วย	ไม่เห็นด้วย
เห็นด้วยอย่	เห็นด้วย	เห็นด้วย	เห็นด้วยอย่	เห็นด้วยอย่	25.00	ไม่เห็นด้วย	ไม่เห็นด้วย	ไม่เห็นด้วย
เห็นด้วย	เห็นด้วยอย่	เห็นด้วย	เห็นด้วย	เห็นด้วย	22.00	ไม่เห็นด้วย	เห็นด้วย	เห็นด้วย
เห็นด้วยอย่	เห็นด้วย	เห็นด้วย	เห็นด้วย	เห็นด้วย	20.00	ไม่เห็นด้วย	เห็นด้วย	เห็นด้วย

เห็นด้วย	เห็นด้วย	เห็นด้วย	เห็นด้วย	เห็นด้วย	24.00	ไม่เห็นด้วย	เห็นด้วย	เห็นด้วย
เห็นด้วย	ไม่เห็นด้วย	เห็นด้วย	เห็นด้วย	เห็นด้วยอย่	21.00	ไม่เห็นด้วย	เห็นด้วย	ไม่เห็นด้วย
เห็นด้วย	ไม่เห็นด้วย	ไม่เห็นด้วย	เห็นด้วย	เห็นด้วย	19.00	ไม่เห็นด้วย	ไม่เห็นด้วย	ไม่เห็นด้วย
เห็นด้วยอย่	เห็นด้วย	เห็นด้วย	เห็นด้วยอย่	เห็นด้วย	25.00	ไม่เห็นด้วย	ไม่เห็นด้วย	ไม่เห็นด้วย
เห็นด้วยอย่	เห็นด้วย	เห็นด้วย	เห็นด้วย	เห็นด้วย	21.00	ไม่เห็นด้วย	เห็นด้วยอย่	เห็นด้วยอย่
เห็นด้วย	เห็นด้วย	ไม่เห็นด้วย	เห็นด้วย	เห็นด้วย	21.00	เห็นด้วย	เห็นด้วย	ไม่เห็นด้วย
เห็นด้วย	เห็นด้วย	เห็นด้วย	เห็นด้วย	เห็นด้วย	21.00	ไม่เห็นด้วย	ไม่เห็นด้วย	ไม่เห็นด้วย
เห็นด้วย	เห็นด้วย	เห็นด้วย	เห็นด้วย	เห็นด้วย	21.00	ไม่เห็นด้วย	ไม่เห็นด้วย	ไม่เห็นด้วย
เห็นด้วย	เห็นด้วย	เห็นด้วย	เห็นด้วย	เห็นด้วย	24.00	ไม่เห็นด้วย	ไม่เห็นด้วย	ไม่เห็นด้วย
เห็นด้วย	เห็นด้วย	ไม่เห็นด้วย	เห็นด้วย	เห็นด้วย	23.00	ไม่เห็นด้วย	ไม่เห็นด้วย	ไม่เห็นด้วย
เห็นด้วย	เห็นด้วย	ไม่เห็นด้วย	เห็นด้วย	เห็นด้วยอย่	21.00	ไม่เห็นด้วย	ไม่เห็นด้วย	เห็นด้วย
เห็นด้วยอย่	เห็นด้วยอย่	เห็นด้วยอย่	เห็นด้วยอย่	เห็นด้วยอย่	21.00	ไม่เห็นด้วยอ	ไม่เห็นด้วยอ	ไม่เห็นด้วยอ
เห็นด้วยอย่	เห็นด้วย	ไม่เห็นด้วย	เห็นด้วยอย่	เห็นด้วยอย่	19.00	ไม่เห็นด้วย	เห็นด้วย	ไม่เห็นด้วย
เห็นด้วย	เห็นด้วย	เห็นด้วย	เห็นด้วย	เห็นด้วย	21.00	ไม่เห็นด้วย	เห็นด้วย	เห็นด้วย
เห็นด้วยอย่	เห็นด้วยอย่	เห็นด้วย	เห็นด้วยอย่	เห็นด้วยอย่	21.00	ไม่เห็นด้วย	ไม่เห็นด้วย	ไม่เห็นด้วย
เห็นด้วยอย่	เห็นด้วย	เห็นด้วย	เห็นด้วยอย่	เห็นด้วยอย่	19.00	ไม่เห็นด้วย	เห็นด้วย	ไม่เห็นด้วย
เห็นด้วย	เห็นด้วย	เห็นด้วย	เห็นด้วย	เห็นด้วย	23.00	ไม่เห็นด้วย	เห็นด้วย	ไม่เห็นด้วย
เห็นด้วย	ไม่เห็นด้วย	ไม่เห็นด้วย	เห็นด้วย	เห็นด้วย	23.00	ไม่เห็นด้วย	ไม่เห็นด้วย	ไม่เห็นด้วย
ไม่เห็นด้วยอ	เห็นด้วย	เห็นด้วย	เห็นด้วย	เห็นด้วย	27.00	ไม่เห็นด้วย	เห็นด้วย	ไม่เห็นด้วย
ไม่เห็นด้วยอ	ไม่เห็นด้วย	ไม่เห็นด้วย	เห็นด้วย	ไม่เห็นด้วยอ	19.00	ไม่เห็นด้วยอ	เห็นด้วย	เห็นด้วย
เห็นด้วย	เห็นด้วย	เห็นด้วย	เห็นด้วย	เห็นด้วย	26.00	ไม่เห็นด้วย	ไม่เห็นด้วย	เห็นด้วย
เห็นด้วยอย่	เห็นด้วย	เห็นด้วย	เห็นด้วย	เห็นด้วยอย่	27.00	เห็นด้วย	เห็นด้วยอย่	ไม่เห็นด้วย
เห็นด้วย	เห็นด้วย	เห็นด้วย	เห็นด้วย	เห็นด้วย	20.00	เห็นด้วย	เห็นด้วย	ไม่เห็นด้วย
เห็นด้วยอย่	เห็นด้วย	เห็นด้วย	เห็นด้วยอย่	เห็นด้วย	18.00	ไม่เห็นด้วย	เห็นด้วย	ไม่เห็นด้วยอ
เห็นด้วย	เห็นด้วย	เห็นด้วย	เห็นด้วย	เห็นด้วย	20.00	ไม่เห็นด้วย	เห็นด้วย	ไม่เห็นด้วย
ไม่เห็นด้วย	เห็นด้วย	ไม่เห็นด้วย	ไม่เห็นด้วย	เห็นด้วย	21.00	ไม่เห็นด้วย	เห็นด้วย	เห็นด้วย
เห็นด้วยอย่	เห็นด้วย	เห็นด้วย	เห็นด้วย	เห็นด้วยอย่	27.00	ไม่เห็นด้วยอ	ไม่เห็นด้วย	ไม่เห็นด้วยอ
ไม่เห็นด้วย	เห็นด้วยอย่	ไม่เห็นด้วย	เห็นด้วย	เห็นด้วย	25.00	ไม่เห็นด้วย	เห็นด้วย	เห็นด้วย
เห็นด้วย	เห็นด้วย	เห็นด้วย	เห็นด้วย	เห็นด้วย	23.00	ไม่เห็นด้วย	เห็นด้วย	ไม่เห็นด้วย
เห็นด้วย	เห็นด้วย	เห็นด้วย	เห็นด้วย	เห็นด้วย	28.00	ไม่เห็นด้วย	เห็นด้วย	เห็นด้วย
เห็นด้วยอย่	เห็นด้วยอย่	เห็นด้วยอย่	เห็นด้วยอย่	เห็นด้วยอย่	20.00	ไม่เห็นด้วยอ	เห็นด้วย	ไม่เห็นด้วยอ
เห็นด้วยอย่	เห็นด้วยอย่	เห็นด้วยอย่	เห็นด้วยอย่	เห็นด้วยอย่	28.00	ไม่เห็นด้วยอ	ไม่เห็นด้วยอ	ไม่เห็นด้วยอ
เห็นด้วย	เห็นด้วย	เห็นด้วย	เห็นด้วย	เห็นด้วย	26.00	ไม่เห็นด้วย	ไม่เห็นด้วย	ไม่เห็นด้วย
เห็นด้วย	เห็นด้วย	เห็นด้วย	เห็นด้วย	เห็นด้วย	21.00	เห็นด้วย	เห็นด้วย	เห็นด้วย
เห็นด้วย	เห็นด้วย	เห็นด้วย	เห็นด้วย	เห็นด้วย	20.00	ไม่เห็นด้วย	ไม่เห็นด้วย	ไม่เห็นด้วย
เห็นด้วย	เห็นด้วย	เห็นด้วย	เห็นด้วย	เห็นด้วย	21.00	ไม่เห็นด้วย	ไม่เห็นด้วย	ไม่เห็นด้วย
เห็นด้วยอย่	เห็นด้วยอย่	เห็นด้วยอย่	เห็นด้วยอย่	เห็นด้วยอย่	21.00	ไม่เห็นด้วยอ	ไม่เห็นด้วยอ	ไม่เห็นด้วยอ
เห็นด้วย	เห็นด้วย	เห็นด้วย	เห็นด้วย	เห็นด้วย	22.00	ไม่เห็นด้วยอ	ไม่เห็นด้วยอ	ไม่เห็นด้วยอ
เห็นด้วย	เห็นด้วย	เห็นด้วย	เห็นด้วย	เห็นด้วย	22.00	ไม่เห็นด้วย	เห็นด้วย	ไม่เห็นด้วย
เห็นด้วยอย่	เห็นด้วยอย่	เห็นด้วยอย่	เห็นด้วยอย่	เห็นด้วยอย่	27.00	ไม่เห็นด้วยอ	ไม่เห็นด้วยอ	ไม่เห็นด้วยอ
เห็นด้วย	เห็นด้วย	เห็นด้วย	เห็นด้วย	เห็นด้วย	20.00	ไม่เห็นด้วย	ไม่เห็นด้วย	ไม่เห็นด้วย
เห็นด้วย	เห็นด้วย	เห็นด้วย	เห็นด้วย	เห็นด้วย	21.00	ไม่เห็นด้วย	ไม่เห็นด้วย	ไม่เห็นด้วย
เห็นด้วยอย่	เห็นด้วยอย่	เห็นด้วยอย่	เห็นด้วยอย่	เห็นด้วยอย่	21.00	ไม่เห็นด้วยอ	ไม่เห็นด้วยอ	ไม่เห็นด้วยอ
เห็นด้วย	ไม่เห็นด้วย	ไม่เห็นด้วย	เห็นด้วย	เห็นด้วย	17.00	ไม่เห็นด้วย	ไม่เห็นด้วย	ไม่เห็นด้วย
เห็นด้วย	เห็นด้วย	เห็นด้วย	เห็นด้วย	เห็นด้วย	26.00	ไม่เห็นด้วย	ไม่เห็นด้วย	ไม่เห็นด้วย
เห็นด้วย	เห็นด้วย	เห็นด้วย	เห็นด้วย	เห็นด้วย	28.00	ไม่เห็นด้วย	ไม่เห็นด้วย	ไม่เห็นด้วย
เห็นด้วยอย่	เห็นด้วยอย่	เห็นด้วยอย่	เห็นด้วยอย่	เห็นด้วยอย่	21.00	ไม่เห็นด้วยอ	ไม่เห็นด้วยอ	ไม่เห็นด้วยอ
เห็นด้วย	เห็นด้วย	เห็นด้วย	เห็นด้วย	เห็นด้วย	21.00	ไม่เห็นด้วย	ไม่เห็นด้วย	ไม่เห็นด้วย
เห็นด้วย	เห็นด้วย	เห็นด้วย	เห็นด้วย	เห็นด้วย	23.00	ไม่เห็นด้วย	เห็นด้วย	เห็นด้วย
เห็นด้วย	เห็นด้วย	เห็นด้วย	เห็นด้วยอย่	เห็นด้วย	21.00	ไม่เห็นด้วย	ไม่เห็นด้วย	ไม่เห็นด้วย
เห็นด้วย	เห็นด้วยอย่	เห็นด้วย	เห็นด้วยอย่	เห็นด้วยอย่	19.00	ไม่เห็นด้วยอ	เห็นด้วย	ไม่เห็นด้วย

เห็นด้วย	เห็นด้วย	เห็นด้วย	เห็นด้วย	เห็นด้วย	21.00	เห็นด้วย	เห็นด้วย	เห็นด้วย
เห็นด้วย	เห็นด้วย	ไม่เห็นด้วย	เห็นด้วย	เห็นด้วย	19.00	ไม่เห็นด้วย	เห็นด้วย	ไม่เห็นด้วย
เห็นด้วย	เห็นด้วย	เห็นด้วย	เห็นด้วยอย่	เห็นด้วยอย่	20.00	ไม่เห็นด้วย	ไม่เห็นด้วย	ไม่เห็นด้วย
ไม่เห็นด้วย	ไม่เห็นด้วย	เห็นด้วย	เห็นด้วย	เห็นด้วยอย่	21.00	ไม่เห็นด้วย	เห็นด้วย	ไม่เห็นด้วย
เห็นด้วยอย่	เห็นด้วย	ไม่เห็นด้วย	เห็นด้วย	เห็นด้วย	26.00	ไม่เห็นด้วย	ไม่เห็นด้วย	ไม่เห็นด้วย
เห็นด้วย	เห็นด้วย	เห็นด้วย	เห็นด้วย	เห็นด้วยอย่	18.00	ไม่เห็นด้วย	เห็นด้วย	ไม่เห็นด้วย
เห็นด้วย	ไม่เห็นด้วย	ไม่เห็นด้วย	เห็นด้วย	เห็นด้วย	25.00	ไม่เห็นด้วย	เห็นด้วย	ไม่เห็นด้วย
เห็นด้วยอย่	เห็นด้วย	เห็นด้วย	เห็นด้วยอย่	เห็นด้วยอย่	23.00	ไม่เห็นด้วยอ	เห็นด้วย	เห็นด้วย
เห็นด้วย	เห็นด้วย	เห็นด้วย	เห็นด้วย	เห็นด้วย	18.00	ไม่เห็นด้วย	เห็นด้วย	ไม่เห็นด้วย
เห็นด้วยอย่	เห็นด้วยอย่	เห็นด้วย	เห็นด้วย	เห็นด้วยอย่	21.00	ไม่เห็นด้วยอ	ไม่เห็นด้วยอ	ไม่เห็นด้วย
เห็นด้วยอย่	เห็นด้วย	เห็นด้วย	เห็นด้วยอย่	เห็นด้วยอย่	21.00	ไม่เห็นด้วยอ	เห็นด้วย	เห็นด้วย
เห็นด้วย	เห็นด้วย	เห็นด้วย	เห็นด้วย	เห็นด้วย	23.00	ไม่เห็นด้วย	ไม่เห็นด้วย	ไม่เห็นด้วย
เห็นด้วย	เห็นด้วย	เห็นด้วย	ไม่เห็นด้วย	ไม่เห็นด้วย	21.00	ไม่เห็นด้วย	ไม่เห็นด้วย	เห็นด้วยอย่
เห็นด้วยอย่	เห็นด้วยอย่	เห็นด้วยอย่	เห็นด้วยอย่	เห็นด้วยอย่	23.00	ไม่เห็นด้วย	เห็นด้วยอย่	ไม่เห็นด้วย
เห็นด้วย	เห็นด้วย	เห็นด้วย	เห็นด้วย	เห็นด้วย	22.00	ไม่เห็นด้วย	ไม่เห็นด้วย	ไม่เห็นด้วย
เห็นด้วยอย่	ไม่เห็นด้วยอ	ไม่เห็นด้วยอ	เห็นด้วยอย่	เห็นด้วยอย่	25.00	เห็นด้วยอย่	ไม่เห็นด้วยอ	ไม่เห็นด้วยอ
เห็นด้วย	เห็นด้วยอย่	เห็นด้วย	เห็นด้วยอย่	เห็นด้วย	21.00	ไม่เห็นด้วย	เห็นด้วย	ไม่เห็นด้วย
เห็นด้วยอย่	เห็นด้วย	เห็นด้วย	เห็นด้วย	เห็นด้วย	26.00	ไม่เห็นด้วย	เห็นด้วย	ไม่เห็นด้วย
เห็นด้วย	เห็นด้วย	ไม่เห็นด้วย	เห็นด้วย	เห็นด้วย	28.00	ไม่เห็นด้วย	เห็นด้วย	ไม่เห็นด้วย
เห็นด้วยอย่	เห็นด้วย	เห็นด้วยอย่	เห็นด้วยอย่	เห็นด้วยอย่	21.00	เห็นด้วยอย่	เห็นด้วย	เห็นด้วย
เห็นด้วย	เห็นด้วย	เห็นด้วย	เห็นด้วย	เห็นด้วย	21.00	ไม่เห็นด้วย	เห็นด้วย	ไม่เห็นด้วย
เห็นด้วย	เห็นด้วย	ไม่เห็นด้วย	เห็นด้วย	เห็นด้วย	27.00	ไม่เห็นด้วย	ไม่เห็นด้วย	เห็นด้วย
เห็นด้วย	เห็นด้วย	เห็นด้วย	เห็นด้วย	เห็นด้วย	26.00	ไม่เห็นด้วย	ไม่เห็นด้วย	ไม่เห็นด้วย
เห็นด้วย	เห็นด้วย	เห็นด้วย	เห็นด้วยอย่	เห็นด้วย	27.00	ไม่เห็นด้วย	เห็นด้วย	เห็นด้วย
เห็นด้วยอย่	เห็นด้วย	เห็นด้วยอย่	เห็นด้วยอย่	เห็นด้วยอย่	25.00	ไม่เห็นด้วยอ	ไม่เห็นด้วยอ	ไม่เห็นด้วยอ
เห็นด้วย	เห็นด้วย	เห็นด้วย	เห็นด้วย	เห็นด้วย	21.00	ไม่เห็นด้วย	ไม่เห็นด้วย	ไม่เห็นด้วย
ไม่เห็นด้วย	เห็นด้วย	ไม่เห็นด้วย	เห็นด้วย	เห็นด้วยอย่	23.00	ไม่เห็นด้วย	ไม่เห็นด้วย	เห็นด้วย
เห็นด้วย	เห็นด้วย	เห็นด้วย	เห็นด้วย	เห็นด้วย	19.00	ไม่เห็นด้วย	ไม่เห็นด้วย	ไม่เห็นด้วย
เห็นด้วย	เห็นด้วยอย่	เห็นด้วยอย่	เห็นด้วย	เห็นด้วย	25.00	ไม่เห็นด้วยอ	ไม่เห็นด้วย	ไม่เห็นด้วยอ
เห็นด้วย	เห็นด้วย	เห็นด้วย	เห็นด้วยอย่	เห็นด้วย	27.00	ไม่เห็นด้วย	เห็นด้วย	เห็นด้วย
เห็นด้วย	เห็นด้วย	เห็นด้วย	เห็นด้วยอย่	เห็นด้วย	23.00	ไม่เห็นด้วย	เห็นด้วยอย่	เห็นด้วย
ไม่เห็นด้วย	เห็นด้วย	เห็นด้วย	เห็นด้วย	เห็นด้วย	21.00	ไม่เห็นด้วย	ไม่เห็นด้วย	ไม่เห็นด้วย
เห็นด้วย	เห็นด้วย	เห็นด้วย	เห็นด้วย	เห็นด้วย	24.00	ไม่เห็นด้วย	เห็นด้วย	เห็นด้วย
เห็นด้วย	เห็นด้วย	เห็นด้วย	เห็นด้วยอย่	เห็นด้วยอย่	21.00	ไม่เห็นด้วย	ไม่เห็นด้วย	ไม่เห็นด้วย
เห็นด้วย	เห็นด้วย	เห็นด้วย	เห็นด้วย	เห็นด้วยอย่	21.00	ไม่เห็นด้วย	เห็นด้วย	เห็นด้วย
เห็นด้วย	เห็นด้วย	เห็นด้วย	เห็นด้วยอย่	เห็นด้วยอย่	24.00	เห็นด้วย	เห็นด้วยอย่	เห็นด้วยอย่
ไม่เห็นด้วย	เห็นด้วยอย่	ไม่เห็นด้วย	เห็นด้วย	เห็นด้วย	28.00	ไม่เห็นด้วย	เห็นด้วย	ไม่เห็นด้วย
เห็นด้วย	เห็นด้วย	ไม่เห็นด้วย	เห็นด้วย	เห็นด้วย	23.00	ไม่เห็นด้วย	ไม่เห็นด้วย	ไม่เห็นด้วย
เห็นด้วยอย่	เห็นด้วย	ไม่เห็นด้วย	เห็นด้วย	เห็นด้วย	23.00	ไม่เห็นด้วย	เห็นด้วยอย่	เห็นด้วย
เห็นด้วย	เห็นด้วย	เห็นด้วย	เห็นด้วย	เห็นด้วย	21.00	ไม่เห็นด้วย	เห็นด้วย	เห็นด้วย
เห็นด้วย	เห็นด้วย	เห็นด้วย	เห็นด้วยอย่	เห็นด้วย	21.00	ไม่เห็นด้วย	ไม่เห็นด้วย	ไม่เห็นด้วย
เห็นด้วย	เห็นด้วยอย่	เห็นด้วย	เห็นด้วยอย่	เห็นด้วยอย่	22.00	ไม่เห็นด้วยอ	เห็นด้วย	ไม่เห็นด้วย
เห็นด้วย	เห็นด้วย	เห็นด้วย	เห็นด้วย	เห็นด้วย	21.00	เห็นด้วย	เห็นด้วย	เห็นด้วย
เห็นด้วย	เห็นด้วย	ไม่เห็นด้วย	เห็นด้วย	เห็นด้วย	19.00	ไม่เห็นด้วย	เห็นด้วย	ไม่เห็นด้วย
เห็นด้วย	เห็นด้วย	เห็นด้วย	เห็นด้วยอย่	เห็นด้วยอย่	23.00	ไม่เห็นด้วย	ไม่เห็นด้วย	ไม่เห็นด้วย
ไม่เห็นด้วย	ไม่เห็นด้วย	เห็นด้วย	เห็นด้วย	เห็นด้วยอย่	21.00	ไม่เห็นด้วย	เห็นด้วย	ไม่เห็นด้วย
เห็นด้วย	เห็นด้วย	เห็นด้วย	เห็นด้วย	เห็นด้วย	25.00	เห็นด้วยอย่	เห็นด้วย	ไม่เห็นด้วย
เห็นด้วย	ไม่เห็นด้วย	ไม่เห็นด้วย	เห็นด้วย	เห็นด้วย	22.00	ไม่เห็นด้วย	เห็นด้วย	ไม่เห็นด้วย
เห็นด้วย	ไม่เห็นด้วย	ไม่เห็นด้วย	เห็นด้วย	เห็นด้วย	23.00	ไม่เห็นด้วย	ไม่เห็นด้วย	ไม่เห็นด้วย

barrier4	barrier5	barrier6	Sum_Barri	HeLD1	HeLD2	HeLD3	HeLD4	HeLD5
ไม่เห็นด้วย	เห็นด้วย	ไม่เห็นด้วย	14.00	ทำได้บ่อยๆ	ทำเป็นประ	ทำได้เป็นบ	ทำได้เป็นบ	ทำเป็นประ
ไม่เห็นด้วย	เห็นด้วย	เห็นด้วย	9.00	ทำได้บ่อยๆ	ทำได้บ่อยๆ	ทำเป็นประ	ทำเป็นประ	ทำได้บ่อยๆ
เห็นด้วย	ไม่เห็นด้วย	ไม่เห็นด้วย	20.00	ทำได้บ่อยๆ	ทำเป็นประ	ทำได้เป็นบ	ทำได้เป็นบ	ทำเป็นประ
ไม่เห็นด้วย	เห็นด้วยอย่	เห็นด้วย	16.00	ทำได้บ่อยๆ	ทำได้บ่อยๆ	ทำได้บ่อยๆ	ทำได้บ่อยๆ	ทำได้บ่อยๆ
เห็นด้วย	ไม่เห็นด้วย	ไม่เห็นด้วย	18.00	ทำได้บ้าง/	ไม่เคยทำเ	ไม่เคยทำเ	ทำได้บ้าง/	ทำได้บ้าง/
เห็นด้วย	เห็นด้วย	เห็นด้วย	8.00	ทำได้เป็นบ	ทำได้บ่อยๆ	ทำได้บ่อยๆ	ทำได้บ่อยๆ	ทำได้บ่อยๆ
เห็นด้วย	ไม่เห็นด้วย	ไม่เห็นด้วย	18.00	ทำได้เป็นบ	ทำได้บ่อยๆ	ทำได้เป็นบ	ทำได้บ่อยๆ	ทำได้บ่อยๆ
ไม่เห็นด้วย	ไม่เห็นด้วย	ไม่เห็นด้วย	12.00	ทำเป็นประ	ทำเป็นประ	ทำเป็นประ	ทำเป็นประ	ทำเป็นประ
เห็นด้วย	เห็นด้วย	เห็นด้วย	18.00	ทำได้เป็นบ	ทำได้เป็นบ	ทำได้เป็นบ	ทำได้เป็นบ	ทำได้เป็นบ
เห็นด้วย	เห็นด้วย	เห็นด้วย	18.00	ทำได้บ้าง/	ทำได้บ้าง/	ทำได้บ่อยๆ	ทำได้บ่อยๆ	ทำเป็นประ
เห็นด้วย	เห็นด้วย	เห็นด้วย	12.00	ทำได้บ้าง/	ทำได้เป็นบ	ทำได้บ่อยๆ	ทำได้บ่อยๆ	ทำได้เป็นบ
ไม่เห็นด้วย	ไม่เห็นด้วย	ไม่เห็นด้วย	18.00	ทำได้บ่อยๆ	ทำเป็นประ	ทำเป็นประ	ทำเป็นประ	ทำเป็นประ
เห็นด้วย	เห็นด้วย	เห็นด้วย	16.00	ทำได้เป็นบ	ทำได้บ่อยๆ	ทำได้เป็นบ	ทำได้บ่อยๆ	ทำได้บ่อยๆ
ไม่เห็นด้วย	ไม่เห็นด้วย	ไม่เห็นด้วย	12.00	ทำได้บ่อยๆ	ทำได้บ่อยๆ	ทำได้บ่อยๆ	ทำได้บ่อยๆ	ทำเป็นประ
เห็นด้วย	ไม่เห็นด้วย	ไม่เห็นด้วย	15.00	ทำได้บ่อยๆ	ทำได้เป็นบ	ทำได้เป็นบ	ทำได้บ่อยๆ	ทำได้เป็นบ
ไม่เห็นด้วย	ไม่เห็นด้วย	ไม่เห็นด้วย	18.00	ทำได้เป็นบ	ทำได้เป็นบ	ทำได้เป็นบ	ทำเป็นประ	ทำได้บ้าง/
เห็นด้วยอย่	เห็นด้วย	เห็นด้วยอย่	18.00	ทำได้บ่อยๆ	ทำได้บ่อยๆ	ทำได้บ่อยๆ	ทำได้บ่อยๆ	ทำได้เป็นบ
ไม่เห็นด้วย	ไม่เห็นด้วย	ไม่เห็นด้วย	13.00	ทำเป็นประ	ทำได้บ่อยๆ	ทำได้เป็นบ	ทำได้บ้าง/	ทำได้เป็นบ
ไม่เห็นด้วย	ไม่เห็นด้วย	ไม่เห็นด้วย	13.00	ทำได้บ่อยๆ	ทำได้เป็นบ	ทำเป็นประ	ทำเป็นประ	ไม่เคยทำเ
ไม่เห็นด้วย	เห็นด้วย	เห็นด้วย	13.00	ทำได้เป็นบ	ทำเป็นประ	ไม่เคยทำเ	ทำได้เป็นบ	ทำได้บ้าง/
ไม่เห็นด้วย	ไม่เห็นด้วย	ไม่เห็นด้วย	17.00	ทำได้บ่อยๆ	ทำเป็นประ	ทำได้บ่อยๆ	ทำได้บ่อยๆ	ทำเป็นประ
เห็นด้วย	ไม่เห็นด้วย	เห็นด้วย	16.00	ทำได้บ้าง/	ทำได้บ้าง/	ทำได้บ้าง/	ไม่เคยทำเ	ทำได้บ้าง/
ไม่เห็นด้วย	ไม่เห็นด้วย	ไม่เห็นด้วย	16.00	ทำเป็นประ	ทำเป็นประ	ทำเป็นประ	ทำได้บ่อยๆ	ไม่เคยทำเ
เห็นด้วยอย่	เห็นด้วยอย่	เห็นด้วย	17.00	ทำได้บ้าง/	ทำได้เป็นบ	ทำได้เป็นบ	ทำได้เป็นบ	ทำได้เป็นบ
ไม่เห็นด้วย	เห็นด้วย	ไม่เห็นด้วย	12.00	ทำได้บ่อยๆ	ทำได้บ่อยๆ	ทำได้บ่อยๆ	ทำได้บ่อยๆ	ทำได้บ่อยๆ
เห็นด้วย	เห็นด้วย	เห็นด้วย	18.00	ทำเป็นประ	ทำเป็นประ	ทำได้บ่อยๆ	ทำได้บ่อยๆ	ทำเป็นประ
เห็นด้วย	ไม่เห็นด้วย	ไม่เห็นด้วย	14.00	ทำได้บ้าง/	ทำเป็นประ	ทำได้บ่อยๆ	ทำเป็นประ	ทำเป็นประ
ไม่เห็นด้วย	ไม่เห็นด้วย	ไม่เห็นด้วย	21.00	ทำได้บ่อยๆ	ทำได้บ้าง/	ทำเป็นประ	ทำได้บ่อยๆ	ทำเป็นประ
ไม่เห็นด้วย	ไม่เห็นด้วย	ไม่เห็นด้วย	18.00	ทำเป็นประ	ทำได้บ่อยๆ	ทำเป็นประ	ทำเป็นประ	ทำเป็นประ
ไม่เห็นด้วย	ไม่เห็นด้วย	ไม่เห็นด้วย	18.00	ทำได้เป็นบ	ทำได้บ่อยๆ	ทำได้บ่อยๆ	ทำเป็นประ	ทำเป็นประ
เห็นด้วย	เห็นด้วย	เห็นด้วย	18.00	ทำได้เป็นบ	ทำได้เป็นบ	ทำได้บ่อยๆ	ทำได้บ่อยๆ	ทำได้เป็นบ
เห็นด้วย	ไม่เห็นด้วย	ไม่เห็นด้วย	14.00	ทำเป็นประ	ทำเป็นประ	ทำเป็นประ	ทำเป็นประ	ทำเป็นประ
เห็นด้วย	เห็นด้วย	เห็นด้วยอย่	8.00	ทำได้เป็นบ	ทำเป็นประ	ทำได้เป็นบ	ทำได้เป็นบ	ทำเป็นประ
เห็นด้วยอย่	เห็นด้วยอย่	เห็นด้วยอย่	11.00	ทำได้บ่อยๆ	ทำได้บ่อยๆ	ทำได้บ่อยๆ	ทำได้บ่อยๆ	ทำเป็นประ
ไม่เห็นด้วย	ไม่เห็นด้วย	ไม่เห็นด้วย	10.00	ทำได้เป็นบ	ทำได้บ่อยๆ	ทำเป็นประ	ทำเป็นประ	ทำเป็นประ
ไม่เห็นด้วย	ไม่เห็นด้วย	ไม่เห็นด้วย	18.00	ทำได้เป็นบ	ทำได้เป็นบ	ทำเป็นประ	ทำเป็นประ	ทำเป็นประ
ไม่เห็นด้วย	ไม่เห็นด้วย	ไม่เห็นด้วย	14.00	ทำได้บ่อยๆ	ทำได้บ่อยๆ	ทำได้เป็นบ	ทำเป็นประ	ทำเป็นประ
ไม่เห็นด้วย	ไม่เห็นด้วย	ไม่เห็นด้วย	18.00	ทำได้เป็นบ	ไม่เคยทำเ	ไม่เคยทำเ	ทำได้เป็นบ	ทำได้บ้าง/
ไม่เห็นด้วย	ไม่เห็นด้วย	เห็นด้วย	15.00	ไม่เคยทำเ	ทำได้เป็นบ	ทำได้บ้าง/	ทำเป็นประ	ทำเป็นประ
เห็นด้วย	ไม่เห็นด้วย	ไม่เห็นด้วย	18.00	ทำได้เป็นบ	ทำได้บ้าง/	ทำได้บ้าง/	ทำได้บ้าง/	ทำได้บ้าง/
เห็นด้วย	ไม่เห็นด้วย	ไม่เห็นด้วย	16.00	ทำได้เป็นบ	ทำได้บ่อยๆ	ทำได้บ่อยๆ	ทำได้บ่อยๆ	ทำได้เป็นบ
เห็นด้วย	เห็นด้วย	ไม่เห็นด้วย	17.00	ทำได้บ้าง/	ทำได้เป็นบ	ทำเป็นประ	ทำเป็นประ	ทำได้บ่อยๆ
เห็นด้วยอย่	เห็นด้วย	เห็นด้วย	12.00	ทำได้บ้าง/	ทำได้เป็นบ	ทำได้เป็นบ	ไม่เคยทำเ	ทำได้บ้าง/
เห็นด้วย	เห็นด้วย	เห็นด้วย	12.00	ทำได้บ่อยๆ	ทำได้เป็นบ	ทำได้บ่อยๆ	ทำได้เป็นบ	ทำได้เป็นบ
ไม่เห็นด้วย	ไม่เห็นด้วย	ไม่เห็นด้วย	12.00	ทำได้เป็นบ	ทำได้เป็นบ	ทำได้เป็นบ	ทำได้บ่อยๆ	ทำเป็นประ
ไม่เห็นด้วย	ไม่เห็นด้วย	ไม่เห็นด้วย	16.00	ทำได้เป็นบ	ทำได้บ้าง/	ทำได้เป็นบ	ทำได้เป็นบ	ทำได้บ่อยๆ
เห็นด้วยอย่	เห็นด้วยอย่	เห็นด้วยอย่	15.00	ทำเป็นประ	ทำเป็นประ	ทำได้บ่อยๆ	ทำเป็นประ	ทำเป็นประ
เห็นด้วย	เห็นด้วย	เห็นด้วย	18.00	ทำเป็นประ	ทำเป็นประ	ทำได้บ่อยๆ	ทำเป็นประ	ทำเป็นประ
ไม่เห็นด้วย	ไม่เห็นด้วย	ไม่เห็นด้วย	24.00	ทำได้เป็นบ	ทำได้เป็นบ	ทำได้เป็นบ	ทำได้เป็นบ	ทำได้เป็นบ
ไม่เห็นด้วย	ไม่เห็นด้วย	ไม่เห็นด้วย	13.00	ทำได้บ่อยๆ	ทำได้บ่อยๆ	ทำได้เป็นบ	ทำได้เป็นบ	ทำได้เป็นบ

ไม่เห็นด้วย	ไม่เห็นด้วย	ไม่เห็นด้วย	18.00	ทำได้เป็นบ	ทำได้บ่อยๆ	ทำได้บ่อยๆ	ทำได้เป็นบ	ทำได้บ่อยๆ
ไม่เห็นด้วย	เห็นด้วย	ไม่เห็นด้วย	16.00	ทำได้เป็นบ	ทำได้บ่อยๆ	ไม่เคยทำเล	ทำได้บ้าง/	ทำได้เป็นบ
ไม่เห็นด้วย	ไม่เห็นด้วย	ไม่เห็นด้วย	14.00	ทำได้เป็นบ	ทำได้บ่อยๆ	ทำเป็นประ	ทำเป็นประ	ทำได้บ่อยๆ
ไม่เห็นด้วย	ไม่เห็นด้วย	ไม่เห็นด้วย	18.00	ทำได้เป็นบ	ทำเป็นประ	ทำเป็นประ	ทำได้เป็นบ	ทำเป็นประ
ไม่เห็นด้วย	ไม่เห็นด้วย	เห็นด้วยอย่	19.00	ทำได้บ่อยๆ	ทำเป็นประ	ทำได้บ่อยๆ	ทำได้บ่อยๆ	ทำได้บ่อยๆ
ไม่เห็นด้วย	ไม่เห็นด้วย	ไม่เห็นด้วย	16.00	ทำได้บ้าง/	ทำได้บ้าง/	ทำได้บ่อยๆ	ทำได้บ้าง/	ทำเป็นประ
ไม่เห็นด้วย	ไม่เห็นด้วย	ไม่เห็นด้วย	16.00	ทำได้บ่อยๆ	ทำเป็นประ	ทำได้บ่อยๆ	ทำได้บ่อยๆ	ทำได้บ่อยๆ
เห็นด้วย	เห็นด้วย	เห็นด้วย	16.00	ทำได้เป็นบ	ไม่เคยทำเล	ทำได้เป็นบ	ไม่เคยทำเล	ไม่เคยทำเล
ไม่เห็นด้วย	ไม่เห็นด้วย	เห็นด้วย	19.00	ทำได้เป็นบ	ทำได้เป็นบ	ทำได้บ่อยๆ	ทำได้บ่อยๆ	ทำได้เป็นบ
ไม่เห็นด้วย	ไม่เห็นด้วย	เห็นด้วย	16.00	ทำได้เป็นบ	ทำได้บ้าง/	ทำได้เป็นบ	ทำได้บ่อยๆ	ทำได้บ้าง/
เห็นด้วย	เห็นด้วย	เห็นด้วย	16.00	ทำเป็นประ	ทำเป็นประ	ทำเป็นประ	ทำได้บ่อยๆ	ทำได้บ่อยๆ
ไม่เห็นด้วย	เห็นด้วย	ไม่เห็นด้วย	23.00	ทำได้เป็นบ	ทำได้บ้าง/	ทำได้บ่อยๆ	ไม่เคยทำเล	ทำได้บ้าง/
ไม่เห็นด้วย	ไม่เห็นด้วย	ไม่เห็นด้วย	15.00	ทำเป็นประ	ทำเป็นประ	ทำเป็นประ	ทำเป็นประ	ทำเป็นประ
ไม่เห็นด้วย	ไม่เห็นด้วย	เห็นด้วยอย่	14.00	ทำได้บ่อยๆ	ทำได้บ่อยๆ	ทำเป็นประ	ทำเป็นประ	ทำเป็นประ
ไม่เห็นด้วย	ไม่เห็นด้วย	ไม่เห็นด้วย	18.00	ทำได้บ่อยๆ	ทำเป็นประ	ทำเป็นประ	ทำเป็นประ	ทำได้เป็นบ
ไม่เห็นด้วย	ไม่เห็นด้วย	ไม่เห็นด้วย	16.00	ทำได้บ้าง/	ทำได้บ่อยๆ	ทำได้บ่อยๆ	ทำเป็นประ	ทำเป็นประ
ไม่เห็นด้วย	ไม่เห็นด้วย	ไม่เห็นด้วย	15.00	ทำได้บ้าง/	ทำได้บ้าง/	ทำได้บ่อยๆ	ทำได้บ่อยๆ	ทำได้บ่อยๆ
ไม่เห็นด้วย	ไม่เห็นด้วย	เห็นด้วย	18.00	ทำได้เป็นบ	ทำได้บ่อยๆ	ทำได้เป็นบ	ทำได้บ่อยๆ	ทำได้เป็นบ
เห็นด้วย	เห็นด้วย	ไม่เห็นด้วย	13.00	ทำได้เป็นบ	ทำได้เป็นบ	ทำได้เป็นบ	ทำได้เป็นบ	ทำได้เป็นบ
เห็นด้วย	ไม่เห็นด้วย	ไม่เห็นด้วย	10.00	ทำได้เป็นบ	ทำได้บ่อยๆ	ทำได้เป็นบ	ทำได้เป็นบ	ทำได้บ่อยๆ
เห็นด้วย	ไม่เห็นด้วย	ไม่เห็นด้วย	24.00	ทำได้บ่อยๆ	ทำได้บ่อยๆ	ทำได้บ่อยๆ	ทำได้บ่อยๆ	ทำได้บ่อยๆ
ไม่เห็นด้วย	เห็นด้วยอย่	ไม่เห็นด้วย	17.00	ทำได้บ่อยๆ	ทำได้บ่อยๆ	ทำได้บ่อยๆ	ทำได้บ่อยๆ	ทำได้เป็นบ
ไม่เห็นด้วย	ไม่เห็นด้วย	ไม่เห็นด้วย	16.00	ทำเป็นประ	ทำได้บ่อยๆ	ทำเป็นประ	ทำเป็นประ	ทำเป็นประ
ไม่เห็นด้วย	ไม่เห็นด้วย	ไม่เห็นด้วย	15.00	ทำได้เป็นบ	ทำเป็นประ	ทำได้บ้าง/	ทำได้บ้าง/	ทำเป็นประ
ไม่เห็นด้วย	ไม่เห็นด้วย	ไม่เห็นด้วย	13.00	ทำได้บ่อยๆ	ทำได้บ่อยๆ	ทำได้บ่อยๆ	ทำได้บ่อยๆ	ทำเป็นประ
เห็นด้วย	ไม่เห็นด้วย	ไม่เห็นด้วย	18.00	ทำได้บ้าง/	ทำได้บ่อยๆ	ไม่เคยทำเล	ไม่เคยทำเล	ทำได้บ้าง/
ไม่เห็นด้วย	ไม่เห็นด้วย	ไม่เห็นด้วย	18.00	ทำเป็นประ	ทำเป็นประ	ทำเป็นประ	ทำเป็นประ	ทำเป็นประ
ไม่เห็นด้วย	ไม่เห็นด้วย	ไม่เห็นด้วย	15.00	ทำได้บ่อยๆ	ทำได้บ่อยๆ	ทำได้บ่อยๆ	ทำได้บ่อยๆ	ทำได้บ่อยๆ
เห็นด้วย	เห็นด้วย	เห็นด้วย	13.00	ทำเป็นประ	ทำเป็นประ	ทำเป็นประ	ทำเป็นประ	ทำเป็นประ
ไม่เห็นด้วย	ไม่เห็นด้วย	เห็นด้วย	13.00	ทำเป็นประ	ทำเป็นประ	ทำเป็นประ	ทำเป็นประ	ทำเป็นประ
ไม่เห็นด้วย	ไม่เห็นด้วย	ไม่เห็นด้วย	12.00	ทำได้บ่อยๆ	ทำได้บ่อยๆ	ทำได้บ่อยๆ	ทำได้บ่อยๆ	ทำได้บ่อยๆ
เห็นด้วย	ไม่เห็นด้วย	ไม่เห็นด้วย	6.00	ทำเป็นประ	ทำเป็นประ	ทำเป็นประ	ทำเป็นประ	ทำเป็นประ
เห็นด้วย	ไม่เห็นด้วย	เห็นด้วย	22.00	ทำเป็นประ	ทำเป็นประ	ทำเป็นประ	ทำเป็นประ	ทำเป็นประ
เห็นด้วย	เห็นด้วย	เห็นด้วย	14.00	ทำเป็นประ	ทำเป็นประ	ทำเป็นประ	ทำเป็นประ	ทำเป็นประ
เห็นด้วย	ไม่เห็นด้วย	ไม่เห็นด้วย	12.00	ทำได้บ่อยๆ	ทำได้บ่อยๆ	ทำเป็นประ	ทำเป็นประ	ทำเป็นประ
ไม่เห็นด้วย	เห็นด้วย	เห็นด้วย	16.00	ทำได้บ่อยๆ	ทำเป็นประ	ทำได้เป็นบ	ทำได้เป็นบ	ทำได้บ่อยๆ
ไม่เห็นด้วย	ไม่เห็นด้วย	ไม่เห็นด้วย	11.00	ทำเป็นประ	ทำเป็นประ	ทำเป็นประ	ทำเป็นประ	ทำเป็นประ
เห็นด้วย	ไม่เห็นด้วย	ไม่เห็นด้วย	17.00	ทำได้บ้าง/	ทำได้เป็นบ	ทำได้บ่อยๆ	ทำได้บ่อยๆ	ทำได้เป็นบ
เห็นด้วย	เห็นด้วย	เห็นด้วย	13.00	ทำได้เป็นบ	ทำได้บ่อยๆ	ทำได้บ่อยๆ	ทำเป็นประ	ทำได้บ่อยๆ
เห็นด้วย	เห็นด้วยอย่	เห็นด้วยอย่	19.00	ทำได้เป็นบ	ทำได้บ้าง/	ทำได้เป็นบ	ทำได้เป็นบ	ทำได้เป็นบ
เห็นด้วย	ไม่เห็นด้วย	เห็นด้วย	14.00	ทำได้บ่อยๆ	ทำได้บ่อยๆ	ทำได้บ่อยๆ	ทำเป็นประ	ทำได้บ่อยๆ
ไม่เห็นด้วย	ไม่เห็นด้วย	เห็นด้วย	14.00	ทำได้เป็นบ	ทำได้บ่อยๆ	ทำได้บ่อยๆ	ทำได้บ่อยๆ	ทำได้บ่อยๆ
ไม่เห็นด้วย	ไม่เห็นด้วย	ไม่เห็นด้วย	17.00	ทำเป็นประ	ทำเป็นประ	ทำเป็นประ	ทำเป็นประ	ทำเป็นประ
เห็นด้วย	ไม่เห็นด้วย	ไม่เห็นด้วย	16.00	ทำได้เป็นบ	ทำได้บ่อยๆ	ทำได้เป็นบ	ทำได้เป็นบ	ทำได้บ่อยๆ
ไม่เห็นด้วย	ไม่เห็นด้วย	ไม่เห็นด้วย	18.00	ทำได้เป็นบ	ทำได้บ่อยๆ	ทำได้เป็นบ	ทำได้เป็นบ	ทำได้เป็นบ
ไม่เห็นด้วย	ไม่เห็นด้วย	ไม่เห็นด้วย	14.00	ทำได้บ่อยๆ	ทำได้บ่อยๆ	ทำได้เป็นบ	ทำได้เป็นบ	ทำได้เป็นบ
เห็นด้วย	เห็นด้วย	เห็นด้วย	17.00	ทำเป็นประ	ทำเป็นประ	ทำเป็นประ	ทำเป็นประ	ทำเป็นประ
เห็นด้วย	ไม่เห็นด้วย	เห็นด้วย	18.00	ทำได้บ้าง/	ทำได้เป็นบ	ทำได้บ้าง/	ไม่เคยทำเล	ทำได้บ้าง/
เห็นด้วย	เห็นด้วย	เห็นด้วย	15.00	ทำได้เป็นบ	ทำได้บ่อยๆ	ทำได้บ้าง/	ทำได้บ้าง/	ทำได้บ่อยๆ
เห็นด้วย	เห็นด้วย	เห็นด้วย	6.00	ทำได้บ้าง/	ทำได้เป็นบ	ทำเป็นประ	ทำได้บ้าง/	ทำเป็นประ
เห็นด้วย	เห็นด้วยอย่	เห็นด้วยอย่	18.00	ทำได้เป็นบ	ทำได้เป็นบ	ไม่เคยทำเล	ไม่เคยทำเล	ไม่เคยทำเล

เห็นด้วย	เห็นด้วย	เห็นด้วย	17.00	ทำได้บ่อยๆ	ทำได้บ่อยๆ	ทำได้บ่อยๆ	ทำได้บ่อยๆ	ทำได้บ่อยๆ
เห็นด้วย	เห็นด้วย	ไม่เห็นด้วย	19.00	ทำได้บ่อยๆ	ทำได้บ่อยๆ	ทำได้บ่อยๆ	ทำได้บ่อยๆ	ทำได้บ่อยๆ
ไม่เห็นด้วย	ไม่เห็นด้วย	ไม่เห็นด้วย	16.00	ทำเป็นประ	ทำได้เป็นบ	ทำได้บ่อยๆ	ทำได้เป็นบ	ทำเป็นประ
ไม่เห็นด้วย	ไม่เห็นด้วย	เห็นด้วยอย่	14.00	ทำได้บ่อยๆ	ทำได้บ่อยๆ	ทำได้บ่อยๆ	ทำได้บ่อยๆ	ทำได้บ่อยๆ
เห็นด้วย	เห็นด้วย	เห็นด้วย	19.00	ทำได้เป็นบ	ทำได้บ่อยๆ	ทำได้บ้าง/	ทำได้บ่อยๆ	ทำได้บ่อยๆ
ไม่เห็นด้วย	ไม่เห็นด้วย	ไม่เห็นด้วย	13.00	ทำได้เป็นบ	ทำได้บ่อยๆ	ทำได้เป็นบ	ทำได้เป็นบ	ทำเป็นประ
ไม่เห็นด้วย	ไม่เห็นด้วย	เห็นด้วย	21.00	ทำได้เป็นบ	ทำได้บ่อยๆ	ทำได้บ่อยๆ	ทำได้บ่อยๆ	ทำเป็นประ
ไม่เห็นด้วย	ไม่เห็นด้วย	ไม่เห็นด้วย	15.00	ทำเป็นประ	ทำได้เป็นบ	ทำเป็นประ	ทำเป็นประ	ทำเป็นประ
เห็นด้วย	ไม่เห็นด้วย	ไม่เห็นด้วย	12.00	ทำได้บ่อยๆ	ทำได้บ่อยๆ	ทำเป็นประ	ทำเป็นประ	ทำเป็นประ
ไม่เห็นด้วย	ไม่เห็นด้วย	ไม่เห็นด้วย	18.00	ทำเป็นประ	ทำได้บ่อยๆ	ทำได้บ่อยๆ	ทำได้เป็นบ	ทำได้บ่อยๆ
ไม่เห็นด้วย	ไม่เห็นด้วย	ไม่เห็นด้วย	18.00	ทำได้เป็นบ	ทำได้เป็นบ	ทำได้เป็นบ	ทำได้บ้าง/	ทำได้บ้าง/
ไม่เห็นด้วย	ไม่เห็นด้วย	ไม่เห็นด้วย	18.00	ทำเป็นประ	ทำได้บ่อยๆ	ทำได้บ่อยๆ	ทำได้เป็นบ	ทำได้บ่อยๆ
ไม่เห็นด้วย	เห็นด้วย	เห็นด้วย	18.00	ทำได้บ่อยๆ	ทำได้บ่อยๆ	ทำได้บ่อยๆ	ทำเป็นประ	ทำได้บ่อยๆ
ไม่เห็นด้วย	ไม่เห็นด้วย	ไม่เห็นด้วย	8.00	ทำได้เป็นบ	ทำได้เป็นบ	ทำได้บ่อยๆ	ทำได้บ่อยๆ	ทำได้บ่อยๆ
เห็นด้วย	ไม่เห็นด้วย	ไม่เห็นด้วย	16.00	ทำเป็นประ	ทำเป็นประ	ทำเป็นประ	ทำเป็นประ	ทำเป็นประ
ไม่เห็นด้วย	ไม่เห็นด้วย	ไม่เห็นด้วย	24.00	ทำได้บ่อยๆ	ทำได้บ่อยๆ	ทำได้บ่อยๆ	ทำได้บ่อยๆ	ทำได้บ่อยๆ
ไม่เห็นด้วย	เห็นด้วย	เห็นด้วย	18.00	ทำเป็นประ	ทำเป็นประ	ทำได้เป็นบ	ทำเป็นประ	ทำได้เป็นบ
ไม่เห็นด้วย	ไม่เห็นด้วย	ไม่เห็นด้วย	24.00	ทำได้เป็นบ	ทำได้บ่อยๆ	ทำได้บ้าง/	ทำได้บ่อยๆ	ทำได้เป็นบ
เห็นด้วย	ไม่เห็นด้วย	ไม่เห็นด้วย	17.00	ทำได้เป็นบ	ทำได้เป็นบ	ทำได้เป็นบ	ทำได้บ้าง/	ทำได้เป็นบ
ไม่เห็นด้วย	ไม่เห็นด้วย	ไม่เห็นด้วย	16.00	ทำเป็นประ	ทำเป็นประ	ทำเป็นประ	ทำเป็นประ	ทำเป็นประ
เห็นด้วย	เห็นด้วย	ไม่เห็นด้วย	17.00	ทำได้บ่อยๆ	ทำเป็นประ	ทำเป็นประ	ทำเป็นประ	ทำเป็นประ
เห็นด้วย	ไม่เห็นด้วย	ไม่เห็นด้วย	17.00	ทำได้เป็นบ	ทำได้เป็นบ	ทำเป็นประ	ทำได้บ่อยๆ	ไม่เคยทำเ
ไม่เห็นด้วย	ไม่เห็นด้วย	ไม่เห็นด้วย	15.00	ทำได้บ้าง/	ทำได้บ่อยๆ	ทำได้เป็นบ	ทำเป็นประ	ทำเป็นประ
เห็นด้วย	เห็นด้วย	ไม่เห็นด้วย	18.00	ทำได้บ่อยๆ	ทำได้บ่อยๆ	ไม่เคยทำเ	ทำได้เป็นบ	ทำได้บ่อยๆ
เห็นด้วยอย่	ไม่เห็นด้วย	ไม่เห็นด้วย	6.00	ทำได้บ่อยๆ	ทำได้บ่อยๆ	ทำได้เป็นบ	ทำได้บ่อยๆ	ทำเป็นประ
ไม่เห็นด้วย	ไม่เห็นด้วย	ไม่เห็นด้วย	15.00	ทำเป็นประ	ทำเป็นประ	ทำได้บ้าง/	ทำได้บ้าง/	ทำได้บ่อยๆ
เห็นด้วย	เห็นด้วย	เห็นด้วย	12.00	ทำได้บ่อยๆ	ทำได้เป็นบ	ทำได้เป็นบ	ทำเป็นประ	ทำเป็นประ
ไม่เห็นด้วย	ไม่เห็นด้วย	ไม่เห็นด้วย	16.00	ทำได้เป็นบ	ทำได้บ่อยๆ	ทำได้บ้าง/	ทำได้เป็นบ	ทำได้บ่อยๆ
ไม่เห็นด้วย	ไม่เห็นด้วย	ไม่เห็นด้วย	14.00	ทำได้บ่อยๆ	ทำได้บ่อยๆ	ทำได้บ่อยๆ	ทำได้บ่อยๆ	ทำได้เป็นบ
เห็นด้วย	เห็นด้วย	เห็นด้วย	17.00	ทำได้บ้าง/	ทำได้บ่อยๆ	ทำได้บ่อยๆ	ทำได้บ่อยๆ	ทำได้บ่อยๆ
เห็นด้วย	ไม่เห็นด้วย	เห็นด้วย	11.00	ทำได้บ่อยๆ	ทำได้บ่อยๆ	ทำเป็นประ	ทำเป็นประ	ทำได้บ่อยๆ
เห็นด้วย	เห็นด้วย	เห็นด้วย	19.00	ทำเป็นประ	ทำได้บ่อยๆ	ทำได้บ่อยๆ	ทำได้บ่อยๆ	ทำเป็นประ
เห็นด้วยอย่	เห็นด้วยอย่	เห็นด้วยอย่	18.00	ทำได้เป็นบ	ทำได้บ่อยๆ	ทำได้เป็นบ	ไม่เคยทำเ	ทำได้บ่อยๆ
เห็นด้วย	ไม่เห็นด้วย	ไม่เห็นด้วย	12.00	ทำได้เป็นบ	ทำเป็นประ	ทำได้บ่อยๆ	ทำได้บ่อยๆ	ทำได้บ่อยๆ
ไม่เห็นด้วย	เห็นด้วย	ไม่เห็นด้วย	7.00	ทำได้บ้าง/	ทำได้บ่อยๆ	ทำเป็นประ	ทำได้บ่อยๆ	ไม่เคยทำเ
เห็นด้วย	ไม่เห็นด้วย	ไม่เห็นด้วย	13.00	ทำได้บ่อยๆ	ทำได้บ่อยๆ	ทำได้บ่อยๆ	ทำเป็นประ	ทำได้บ่อยๆ
ไม่เห็นด้วย	ไม่เห็นด้วย	ไม่เห็นด้วย	17.00	ทำได้เป็นบ	ทำได้เป็นบ	ทำได้บ่อยๆ	ทำได้บ่อยๆ	ทำเป็นประ
ไม่เห็นด้วย	ไม่เห็นด้วย	ไม่เห็นด้วย	24.00	ทำได้บ้าง/	ทำเป็นประ	ทำได้บ่อยๆ	ทำได้บ้าง/	ทำเป็นประ
ไม่เห็นด้วย	ไม่เห็นด้วย	ไม่เห็นด้วย	16.00	ทำได้เป็นบ	ทำได้เป็นบ	ทำได้บ่อยๆ	ทำได้บ่อยๆ	ทำได้เป็นบ
ไม่เห็นด้วย	ไม่เห็นด้วย	ไม่เห็นด้วย	18.00	ทำได้เป็นบ	ทำได้บ่อยๆ	ทำได้เป็นบ	ทำได้บ่อยๆ	ทำได้บ่อยๆ
ไม่เห็นด้วย	ไม่เห็นด้วย	เห็นด้วย	16.00	ทำได้บ่อยๆ	ทำเป็นประ	ทำเป็นประ	ทำเป็นประ	ทำเป็นประ
ไม่เห็นด้วย	เห็นด้วย	ไม่เห็นด้วย	7.00	ทำเป็นประ	ทำได้บ่อยๆ	ทำได้บ่อยๆ	ทำได้เป็นบ	ทำเป็นประ
ไม่เห็นด้วย	ไม่เห็นด้วย	ไม่เห็นด้วย	16.00	ทำได้บ่อยๆ	ทำได้บ้าง/	ทำได้บ่อยๆ	ทำได้เป็นบ	ทำได้บ้าง/
ไม่เห็นด้วย	ไม่เห็นด้วย	ไม่เห็นด้วย	12.00	ทำได้เป็นบ	ทำได้บ่อยๆ	ทำได้เป็นบ	ทำได้เป็นบ	ทำได้บ่อยๆ
ไม่เห็นด้วย	ไม่เห็นด้วย	เห็นด้วย	14.00	ทำได้บ่อยๆ	ทำได้บ่อยๆ	ทำได้บ่อยๆ	ทำเป็นประ	ทำได้บ่อยๆ
เห็นด้วย	ไม่เห็นด้วย	ไม่เห็นด้วย	18.00	ทำเป็นประ	ทำได้บ่อยๆ	ทำได้บ้าง/	ทำได้บ่อยๆ	ทำได้บ้าง/
เห็นด้วย	เห็นด้วย	เห็นด้วย	16.00	ทำได้บ่อยๆ	ทำได้บ่อยๆ	ทำได้บ่อยๆ	ทำได้บ่อยๆ	ทำได้บ่อยๆ
เห็นด้วย	เห็นด้วย	ไม่เห็นด้วย	17.00	ทำเป็นประ	ทำได้บ่อยๆ	ทำได้เป็นบ	ทำได้เป็นบ	ทำเป็นประ
ไม่เห็นด้วย	เห็นด้วย	ไม่เห็นด้วย	15.00	ทำได้บ้าง/	ทำได้เป็นบ	ทำได้เป็นบ	ทำได้เป็นบ	ทำได้บ้าง/
ไม่เห็นด้วย	ไม่เห็นด้วย	ไม่เห็นด้วย	17.00	ทำได้บ่อยๆ	ทำเป็นประ	ทำเป็นประ	ทำเป็นประ	ทำเป็นประ
เห็นด้วย	เห็นด้วย	ไม่เห็นด้วย	24.00	ทำได้เป็นบ	ทำได้เป็นบ	ทำได้บ่อยๆ	ทำได้เป็นบ	ทำได้เป็นบ

ไม่เห็นด้วย	ไม่เห็นด้วย	ไม่เห็นด้วย	18.00	ทำได้บ้าง/ท	ทำได้บ่อยๆ	ทำได้เป็นบ	ทำได้เป็นบ	ทำได้บ่อยๆ
เห็นด้วย	เห็นด้วย	ไม่เห็นด้วย	16.00	ทำได้บ่อยๆ	ทำได้บ่อยๆ	ทำได้บ่อยๆ	ทำได้บ่อยๆ	ทำได้บ่อยๆ
ไม่เห็นด้วย	ไม่เห็นด้วย	ไม่เห็นด้วย	16.00	ทำได้บ่อยๆ	ทำเป็นประจ	ทำได้บ้าง/ท	ทำได้บ่อยๆ	ทำเป็นประจ
ไม่เห็นด้วย	ไม่เห็นด้วย	ไม่เห็นด้วย	18.00	ทำเป็นประจ	ทำเป็นประจ	ทำเป็นประจ	ทำได้บ่อยๆ	ทำได้บ่อยๆ
ไม่เห็นด้วย	ไม่เห็นด้วย	เห็นด้วย	16.00	ทำเป็นประจ	ทำเป็นประจ	ทำเป็นประจ	ทำเป็นประจ	ทำเป็นประจ
ไม่เห็นด้วย	ไม่เห็นด้วย	ไม่เห็นด้วย	12.00	ทำได้บ้าง/ท	ทำได้บ้าง/ท	ทำได้บ้าง/ท	ทำได้บ้าง/ท	ทำได้บ้าง/ท
เห็นด้วย	เห็นด้วย	เห็นด้วย	15.00	ทำเป็นประจ	ทำเป็นประจ	ทำเป็นประจ	ทำเป็นประจ	ทำเป็นประจ
เห็นด้วย	ไม่เห็นด้วย	ไม่เห็นด้วย	17.00	ทำได้บ่อยๆ	ทำเป็นประจ	ทำเป็นประจ	ทำเป็นประจ	ทำเป็นประจ
เห็นด้วย	เห็นด้วย	ไม่เห็นด้วย	15.00	ทำได้บ้าง/ท	ทำเป็นประจ	ทำได้บ้าง/ท	ทำได้บ้าง/ท	ไม่เคยทำเล
ไม่เห็นด้วยอ	ไม่เห็นด้วยอ	ไม่เห็นด้วยอ	19.00	ทำได้เป็นบ	ทำได้บ่อยๆ	ทำได้บ่อยๆ	ทำเป็นประจ	ทำเป็นประจ
ไม่เห็นด้วย	ไม่เห็นด้วย	ไม่เห็นด้วย	17.00	ทำได้บ่อยๆ	ทำเป็นประจ	ทำได้บ่อยๆ	ทำเป็นประจ	ทำเป็นประจ
เห็นด้วย	ไม่เห็นด้วย	ไม่เห็นด้วย	16.00	ทำได้เป็นบ	ทำได้บ้าง/ท	ไม่เคยทำเล	ทำได้เป็นบ	ไม่เคยทำเล
ไม่เห็นด้วย	ไม่เห็นด้วย	ไม่เห็นด้วย	12.00	ทำได้บ่อยๆ	ทำเป็นประจ	ทำเป็นประจ	ทำได้เป็นบ	ทำได้เป็นบ
ไม่เห็นด้วยอ	ไม่เห็นด้วย	ไม่เห็นด้วย	14.00	ทำได้บ่อยๆ	ทำเป็นประจ	ทำได้บ้าง/ท	ทำเป็นประจ	ทำได้เป็นบ
เห็นด้วย	ไม่เห็นด้วย	ไม่เห็นด้วย	15.00	ทำได้เป็นบ	ทำได้บ้าง/ท	ไม่เคยทำเล	ทำได้บ่อยๆ	ทำได้บ่อยๆ
เห็นด้วยอย่	ไม่เห็นด้วย	ไม่เห็นด้วย	16.00	ทำได้บ่อยๆ	ทำได้เป็นบ	ทำได้บ้าง/ท	ทำเป็นประจ	ทำเป็นประจ
ไม่เห็นด้วย	ไม่เห็นด้วย	ไม่เห็นด้วย	17.00	ทำเป็นประจ	ทำเป็นประจ	ทำเป็นประจ	ทำเป็นประจ	ทำเป็นประจ
เห็นด้วย	ไม่เห็นด้วย	ไม่เห็นด้วย	18.00	ทำได้เป็นบ	ทำได้เป็นบ	ทำได้เป็นบ	ทำได้บ้าง/ท	ทำได้เป็นบ
ไม่เห็นด้วย	ไม่เห็นด้วยอ	ไม่เห็นด้วย	16.00	ทำได้บ่อยๆ	ทำเป็นประจ	ทำได้บ่อยๆ	ทำได้บ่อยๆ	ทำเป็นประจ
ไม่เห็นด้วย	ไม่เห็นด้วย	ไม่เห็นด้วย	17.00	ทำได้บ่อยๆ	ทำได้บ่อยๆ	ทำได้บ่อยๆ	ทำได้บ่อยๆ	ทำได้บ่อยๆ
เห็นด้วย	ไม่เห็นด้วย	ไม่เห็นด้วย	14.00	ทำได้บ่อยๆ	ทำเป็นประจ	ทำได้เป็นบ	ทำได้บ่อยๆ	ทำเป็นประจ
ไม่เห็นด้วย	ไม่เห็นด้วยอ	ไม่เห็นด้วย	15.00	ทำเป็นประจ	ทำเป็นประจ	ทำได้บ่อยๆ	ทำได้บ่อยๆ	ทำเป็นประจ
ไม่เห็นด้วย	ไม่เห็นด้วย	ไม่เห็นด้วย	11.00	ทำได้เป็นบ	ทำได้เป็นบ	ทำเป็นประจ	ทำเป็นประจ	ทำเป็นประจ
ไม่เห็นด้วย	เห็นด้วย	ไม่เห็นด้วย	17.00	ทำได้เป็นบ	ทำได้บ่อยๆ	ทำได้บ่อยๆ	ทำได้บ่อยๆ	ทำได้เป็นบ
เห็นด้วย	ไม่เห็นด้วย	ไม่เห็นด้วย	16.00	ทำได้เป็นบ	ทำได้บ่อยๆ	ทำได้บ่อยๆ	ทำได้บ่อยๆ	ทำได้เป็นบ
ไม่เห็นด้วย	ไม่เห็นด้วย	ไม่เห็นด้วย	24.00	ทำได้เป็นบ	ทำได้เป็นบ	ทำได้เป็นบ	ทำได้เป็นบ	ทำได้บ้าง/ท
ไม่เห็นด้วย	ไม่เห็นด้วย	ไม่เห็นด้วย	18.00	ทำได้บ่อยๆ	ทำได้บ่อยๆ	ทำเป็นประจ	ทำเป็นประจ	ทำเป็นประจ
เห็นด้วย	เห็นด้วย	เห็นด้วย	12.00	ทำได้เป็นบ	ทำได้เป็นบ	ทำได้บ้าง/ท	ทำได้เป็นบ	ทำได้เป็นบ
ไม่เห็นด้วย	เห็นด้วย	เห็นด้วย	13.00	ไม่เคยทำเล	ไม่เคยทำเล	ไม่เคยทำเล	ไม่เคยทำเล	ไม่เคยทำเล
ไม่เห็นด้วยอ	ไม่เห็นด้วยอ	ไม่เห็นด้วยอ	18.00	ทำได้บ่อยๆ	ทำได้บ่อยๆ	ทำได้เป็นบ	ทำได้บ่อยๆ	ทำได้บ่อยๆ
ไม่เห็นด้วย	ไม่เห็นด้วยอ	ไม่เห็นด้วย	12.00	ทำได้บ่อยๆ	ทำได้เป็นบ	ทำได้เป็นบ	ทำได้บ่อยๆ	ทำเป็นประจ
ไม่เห็นด้วย	ไม่เห็นด้วย	ไม่เห็นด้วย	18.00	ทำได้บ้าง/ท	ทำได้เป็นบ	ทำได้บ่อยๆ	ทำได้เป็นบ	ทำได้บ่อยๆ
ไม่เห็นด้วย	เห็นด้วย	ไม่เห็นด้วย	16.00	ทำเป็นประจ	ทำเป็นประจ	ทำได้เป็นบ	ทำได้บ่อยๆ	ทำได้บ่อยๆ
ไม่เห็นด้วย	ไม่เห็นด้วย	ไม่เห็นด้วย	17.00	ทำได้เป็นบ	ทำได้บ่อยๆ	ไม่เคยทำเล	ไม่เคยทำเล	ทำเป็นประจ
ไม่เห็นด้วย	ไม่เห็นด้วยอ	เห็นด้วย	22.00	ทำเป็นประจ	ทำเป็นประจ	ทำได้บ่อยๆ	ทำเป็นประจ	ทำได้บ่อยๆ
ไม่เห็นด้วย	ไม่เห็นด้วย	ไม่เห็นด้วย	17.00	ทำได้บ่อยๆ	ทำได้บ่อยๆ	ทำได้บ่อยๆ	ทำเป็นประจ	ทำเป็นประจ
เห็นด้วย	เห็นด้วย	เห็นด้วย	14.00	ทำได้เป็นบ	ทำได้เป็นบ	ทำได้บ้าง/ท	ทำได้เป็นบ	ทำได้เป็นบ
ไม่เห็นด้วย	ไม่เห็นด้วย	ไม่เห็นด้วย	16.00	ทำได้เป็นบ	ทำเป็นประจ	ทำได้เป็นบ	ทำได้เป็นบ	ทำได้บ่อยๆ
เห็นด้วย	ไม่เห็นด้วย	ไม่เห็นด้วย	16.00	ทำเป็นประจ	ทำเป็นประจ	ทำเป็นประจ	ทำเป็นประจ	ทำเป็นประจ
ไม่เห็นด้วย	ไม่เห็นด้วย	ไม่เห็นด้วย	18.00	ทำได้บ่อยๆ	ทำได้บ่อยๆ	ทำได้เป็นบ	ทำได้เป็นบ	ทำได้เป็นบ
ไม่เห็นด้วย	ไม่เห็นด้วย	ไม่เห็นด้วย	18.00	ทำได้เป็นบ	ทำได้บ่อยๆ	ทำได้บ้าง/ท	ทำได้เป็นบ	ทำได้บ่อยๆ
ไม่เห็นด้วย	เห็นด้วย	ไม่เห็นด้วย	21.00	ทำได้บ่อยๆ	ทำได้เป็นบ	ทำได้บ่อยๆ	ทำได้บ่อยๆ	ทำได้บ่อยๆ
เห็นด้วย	ไม่เห็นด้วย	เห็นด้วย	17.00	ทำเป็นประจ	ทำเป็นประจ	ทำได้บ่อยๆ	ทำเป็นประจ	ทำเป็นประจ
ไม่เห็นด้วย	ไม่เห็นด้วย	ไม่เห็นด้วย	17.00	ทำได้บ่อยๆ	ทำเป็นประจ	ทำได้บ่อยๆ	ทำเป็นประจ	ทำได้บ่อยๆ
ไม่เห็นด้วยอ	เห็นด้วยอย่	เห็นด้วยอย่	15.00	ทำได้บ่อยๆ	ทำได้บ่อยๆ	ทำได้เป็นบ	ทำได้บ่อยๆ	ทำได้บ่อยๆ
เห็นด้วย	เห็นด้วย	ไม่เห็นด้วย	15.00	ทำเป็นประจ	ทำได้บ่อยๆ	ทำเป็นประจ	ทำเป็นประจ	ทำเป็นประจ
ไม่เห็นด้วย	ไม่เห็นด้วย	เห็นด้วย	14.00	ทำได้บ่อยๆ	ทำได้เป็นบ	ทำได้บ่อยๆ	ทำได้บ่อยๆ	ทำได้บ่อยๆ
ไม่เห็นด้วย	ไม่เห็นด้วย	ไม่เห็นด้วย	16.00	ทำได้บ่อยๆ	ทำเป็นประจ	ทำได้เป็นบ	ทำเป็นประจ	ทำเป็นประจ
เห็นด้วย	เห็นด้วย	เห็นด้วย	18.00	ทำได้บ่อยๆ	ทำเป็นประจ	ทำได้บ่อยๆ	ทำได้บ่อยๆ	ทำได้บ่อยๆ
ไม่เห็นด้วย	ไม่เห็นด้วย	ไม่เห็นด้วย	14.00	ทำได้บ้าง/ท	ทำได้บ่อยๆ	ทำได้บ่อยๆ	ทำเป็นประจ	ทำได้บ่อยๆ
ไม่เห็นด้วย	ไม่เห็นด้วย	ไม่เห็นด้วย	18.00	ทำเป็นประจ	ทำเป็นประจ	ทำเป็นประจ	ทำเป็นประจ	ทำเป็นประจ

ไม่เห็นด้วย	ไม่เห็นด้วย	ไม่เห็นด้วย	12.00	ทำได้บ่อยๆ	ทำเป็นประ	ทำได้เป็นบ	ทำได้เป็นบ	ทำได้เป็นบ
ไม่เห็นด้วย	ไม่เห็นด้วย	ไม่เห็นด้วย	16.00	ทำได้เป็นบ	ทำได้บ่อยๆ	ทำได้บ่อยๆ	ทำเป็นประ	ทำเป็นประ
เห็นด้วย	ไม่เห็นด้วย	ไม่เห็นด้วย	18.00	ทำเป็นประ	ทำเป็นประ	ทำเป็นประ	ทำเป็นประ	ทำเป็นประ
ไม่เห็นด้วย	ไม่เห็นด้วย	ไม่เห็นด้วย	18.00	ทำได้บ่อยๆ	ทำเป็นประ	ทำเป็นประ	ทำเป็นประ	ทำเป็นประ
ไม่เห็นด้วย	ไม่เห็นด้วย	ไม่เห็นด้วย	18.00	ทำได้เป็นบ	ทำได้เป็นบ	ทำได้บ้าง/	ทำได้บ่อยๆ	ทำได้บ้าง/
ไม่เห็นด้วย	ไม่เห็นด้วย	ไม่เห็นด้วย	16.00	ทำได้บ่อยๆ	ทำเป็นประ	ทำได้บ่อยๆ	ทำเป็นประ	ทำเป็นประ
เห็นด้วย	เห็นด้วย	เห็นด้วย	16.00	ทำได้บ่อยๆ	ทำได้บ่อยๆ	ทำได้บ่อยๆ	ทำได้บ่อยๆ	ทำได้บ่อยๆ
ไม่เห็นด้วย	ไม่เห็นด้วย	ไม่เห็นด้วย	17.00	ทำได้บ่อยๆ	ทำเป็นประ	ทำได้บ่อยๆ	ทำได้บ่อยๆ	ทำได้เป็นบ
เห็นด้วย	เห็นด้วย	เห็นด้วย	22.00	ทำได้เป็นบ	ทำได้เป็นบ	ทำได้เป็นบ	ทำได้เป็นบ	ทำได้เป็นบ
เห็นด้วยอย่	เห็นด้วย	เห็นด้วย	15.00	ทำได้บ่อยๆ	ทำได้บ่อยๆ	ทำได้บ่อยๆ	ทำเป็นประ	ทำได้บ่อยๆ
ไม่เห็นด้วย	ไม่เห็นด้วย	ไม่เห็นด้วย	17.00	ทำได้บ่อยๆ	ทำเป็นประ	ทำเป็นประ	ทำเป็นประ	ทำเป็นประ
เห็นด้วยอย่	เห็นด้วยอย่	เห็นด้วย	8.00	ทำได้บ่อยๆ	ทำได้บ่อยๆ	ทำได้เป็นบ	ทำได้บ่อยๆ	ทำได้บ่อยๆ
เห็นด้วยอย่	เห็นด้วย	เห็นด้วยอย่	8.00	ทำได้บ่อยๆ	ทำได้บ่อยๆ	ทำได้บ่อยๆ	ทำได้บ่อยๆ	ทำได้บ่อยๆ
เห็นด้วย	เห็นด้วย	ไม่เห็นด้วย	16.00	ทำได้บ่อยๆ	ทำเป็นประ	ทำได้บ่อยๆ	ทำได้บ่อยๆ	ทำเป็นประ
เห็นด้วย	เห็นด้วย	เห็นด้วย	18.00	ทำได้บ่อยๆ	ทำได้เป็นบ	ทำได้บ่อยๆ	ทำได้เป็นบ	ทำได้บ่อยๆ
เห็นด้วย	เห็นด้วย	เห็นด้วย	17.00	ทำได้บ่อยๆ	ทำได้เป็นบ	ทำได้บ่อยๆ	ทำเป็นประ	ทำได้บ่อยๆ
ไม่เห็นด้วย	ไม่เห็นด้วย	เห็นด้วย	14.00	ทำได้เป็นบ	ทำได้บ่อยๆ	ทำได้บ่อยๆ	ทำได้เป็นบ	ทำได้เป็นบ
เห็นด้วย	ไม่เห็นด้วย	เห็นด้วย	12.00	ทำเป็นประ	ทำได้บ่อยๆ	ทำได้เป็นบ	ทำได้บ่อยๆ	ทำได้บ่อยๆ
เห็นด้วยอย่	เห็นด้วยอย่	เห็นด้วยอย่	8.00	ทำได้เป็นบ	ทำได้เป็นบ	ทำได้บ่อยๆ	ทำได้บ่อยๆ	ทำได้บ่อยๆ
ไม่เห็นด้วย	เห็นด้วย	เห็นด้วย	16.00	ทำเป็นประ	ทำได้บ่อยๆ	ทำได้บ่อยๆ	ทำเป็นประ	ทำได้บ่อยๆ
ไม่เห็นด้วย	ไม่เห็นด้วย	เห็นด้วย	18.00	ทำเป็นประ	ทำเป็นประ	ทำได้เป็นบ	ทำเป็นประ	ทำเป็นประ
เห็นด้วย	ไม่เห็นด้วย	เห็นด้วย	18.00	ทำได้บ่อยๆ	ทำได้บ่อยๆ	ทำได้เป็นบ	ทำได้บ้าง/	ทำได้บ่อยๆ
ไม่เห็นด้วย	ไม่เห็นด้วย	เห็นด้วย	13.00	ทำได้เป็นบ	ทำได้เป็นบ	ทำได้เป็นบ	ทำได้บ้าง/	ทำได้เป็นบ
ไม่เห็นด้วย	ไม่เห็นด้วย	ไม่เห็นด้วย	7.00	ทำเป็นประ	ทำเป็นประ	ทำเป็นประ	ทำเป็นประ	ทำเป็นประ
ไม่เห็นด้วย	ไม่เห็นด้วย	เห็นด้วย	14.00	ทำได้บ่อยๆ	ทำได้เป็นบ	ทำได้บ้าง/	ทำได้เป็นบ	ทำได้เป็นบ
เห็นด้วย	ไม่เห็นด้วย	ไม่เห็นด้วย	15.00	ทำได้เป็นบ	ทำได้เป็นบ	ทำได้บ้าง/	ทำได้บ้าง/	ทำได้เป็นบ
ไม่เห็นด้วย	ไม่เห็นด้วย	ไม่เห็นด้วย	15.00	ทำได้บ่อยๆ	ทำได้บ่อยๆ	ทำได้บ่อยๆ	ทำได้บ่อยๆ	ทำได้บ่อยๆ
ไม่เห็นด้วย	เห็นด้วย	ไม่เห็นด้วย	10.00	ทำได้บ่อยๆ	ทำได้บ่อยๆ	ทำได้เป็นบ	ทำได้บ่อยๆ	ทำได้เป็นบ
เห็นด้วย	เห็นด้วย	เห็นด้วย	18.00	ทำได้บ่อยๆ	ทำได้บ่อยๆ	ทำได้บ่อยๆ	ทำได้เป็นบ	ทำได้บ่อยๆ
เห็นด้วย	เห็นด้วย	เห็นด้วยอย่	13.00	ทำได้บ่อยๆ	ทำได้บ่อยๆ	ทำได้เป็นบ	ทำได้เป็นบ	ทำได้บ่อยๆ
เห็นด้วย	ไม่เห็นด้วย	เห็นด้วย	18.00	ทำได้บ่อยๆ	ทำได้บ่อยๆ	ทำได้เป็นบ	ทำได้เป็นบ	ทำได้บ่อยๆ
ไม่เห็นด้วย	ไม่เห็นด้วย	ไม่เห็นด้วย	17.00	ทำเป็นประ	ทำได้บ่อยๆ	ทำเป็นประ	ทำเป็นประ	ทำเป็นประ
เห็นด้วยอย่	เห็นด้วยอย่	เห็นด้วยอย่	16.00	ทำได้บ่อยๆ	ทำเป็นประ	ทำได้บ่อยๆ	ทำได้เป็นบ	ทำได้บ่อยๆ
ไม่เห็นด้วย	ไม่เห็นด้วย	ไม่เห็นด้วย	18.00	ทำได้บ้าง/	ทำเป็นประ	ทำได้บ่อยๆ	ทำได้บ่อยๆ	ทำเป็นประ
เห็นด้วยอย่	เห็นด้วยอย่	เห็นด้วยอย่	18.00	ทำเป็นประ	ทำเป็นประ	ทำได้บ่อยๆ	ทำได้บ่อยๆ	ทำเป็นประ
เห็นด้วย	ไม่เห็นด้วย	ไม่เห็นด้วย	12.00	ทำเป็นประ	ทำเป็นประ	ทำได้บ่อยๆ	ทำได้บ่อยๆ	ทำได้บ่อยๆ
ไม่เห็นด้วย	ไม่เห็นด้วย	เห็นด้วย	13.00	ทำเป็นประ	ทำได้บ่อยๆ	ทำได้บ่อยๆ	ทำได้บ่อยๆ	ทำเป็นประ
เห็นด้วย	ไม่เห็นด้วย	ไม่เห็นด้วย	20.00	ทำได้บ่อยๆ	ทำเป็นประ	ทำเป็นประ	ทำเป็นประ	ทำเป็นประ
เห็นด้วย	เห็นด้วยอย่	เห็นด้วยอย่	17.00	ทำได้บ่อยๆ	ทำได้เป็นบ	ทำได้เป็นบ	ทำได้บ่อยๆ	ทำได้บ่อยๆ
ไม่เห็นด้วย	ไม่เห็นด้วย	ไม่เห็นด้วย	15.00	ทำได้บ่อยๆ	ทำเป็นประ	ทำเป็นประ	ทำเป็นประ	ทำเป็นประ
เห็นด้วยอย่	เห็นด้วย	เห็นด้วยอย่	14.00	ทำได้บ่อยๆ	ทำได้บ่อยๆ	ทำได้เป็นบ	ทำได้บ่อยๆ	ทำได้เป็นบ
เห็นด้วย	เห็นด้วย	ไม่เห็นด้วย	18.00	ทำได้เป็นบ	ทำได้บ่อยๆ	ทำได้เป็นบ	ทำได้บ่อยๆ	ทำได้บ้าง/
ไม่เห็นด้วย	ไม่เห็นด้วย	ไม่เห็นด้วย	16.00	ทำเป็นประ	ทำเป็นประ	ทำเป็นประ	ทำเป็นประ	ทำเป็นประ
เห็นด้วย	เห็นด้วย	เห็นด้วย	15.00	ทำเป็นประ	ทำเป็นประ	ทำได้บ่อยๆ	ทำได้บ่อยๆ	ทำได้เป็นบ
เห็นด้วย	ไม่เห็นด้วย	ไม่เห็นด้วย	16.00	ทำได้บ่อยๆ	ทำได้บ่อยๆ	ทำได้บ้าง/	ทำได้เป็นบ	ทำได้บ่อยๆ
ไม่เห็นด้วย	เห็นด้วย	เห็นด้วย	15.00	ทำเป็นประ	ทำได้บ่อยๆ	ทำเป็นประ	ทำได้เป็นบ	ทำได้บ่อยๆ
เห็นด้วยอย่	เห็นด้วยอย่	เห็นด้วย	14.00	ทำเป็นประ	ทำเป็นประ	ทำเป็นประ	ทำเป็นประ	ทำได้บ่อยๆ
เห็นด้วยอย่	เห็นด้วยอย่	เห็นด้วยอย่	14.00	ทำได้บ่อยๆ	ทำได้บ่อยๆ	ทำได้บ่อยๆ	ทำได้เป็นบ	ทำได้เป็นบ
เห็นด้วยอย่	เห็นด้วย	เห็นด้วย	18.00	ทำได้บ่อยๆ	ทำได้บ่อยๆ	ทำได้บ่อยๆ	ทำได้เป็นบ	ทำได้บ่อยๆ
เห็นด้วยอย่	เห็นด้วย	เห็นด้วย	16.00	ทำได้บ้าง/	ทำได้บ้าง/	ทำได้เป็นบ	ทำได้เป็นบ	ทำได้บ้าง/
เห็นด้วย	เห็นด้วยอย่	เห็นด้วยอย่	18.00	ทำได้บ้าง/	ทำได้บ้าง/	ทำเป็นประ	ทำเป็นประ	ทำได้เป็นบ

ไม่เห็นด้วย	เห็นด้วย	เห็นด้วย	14.00	ทำได้บ่อยๆ	ทำได้เป็นบ	ทำได้บ่อยๆ	ทำได้เป็นบ	ทำได้บ่อยๆ
ไม่เห็นด้วย	ไม่เห็นด้วย	ไม่เห็นด้วย	18.00	ทำได้เป็นบ	ทำได้บ่อยๆ	ทำได้บ่อยๆ	ทำได้บ้าง/ไ	ไม่เคยทำเล
ไม่เห็นด้วย	ไม่เห็นด้วย	ไม่เห็นด้วย	12.00	ทำเป็นประจ	ทำเป็นประจ	ทำเป็นประจ	ทำเป็นประจ	ทำเป็นประจ
ไม่เห็นด้วย	ไม่เห็นด้วย	เห็นด้วย	18.00	ทำได้บ่อยๆ	ทำได้บ่อยๆ	ทำได้เป็นบ	ทำเป็นประจ	ทำได้บ้าง/ไ
ไม่เห็นด้วยอ	ไม่เห็นด้วยอ	ไม่เห็นด้วยอ	16.00	ทำได้เป็นบ	ทำเป็นประจ	ทำได้บ่อยๆ	ทำเป็นประจ	ทำเป็นประจ
เห็นด้วย	ไม่เห็นด้วย	ไม่เห็นด้วย	14.00	ทำได้บ่อยๆ	ทำได้เป็นบ	ทำได้เป็นบ	ทำได้เป็นบ	ทำได้เป็นบ
เห็นด้วย	เห็นด้วย	เห็นด้วยอย่	15.00	ทำได้บ้าง/ไ	ทำได้บ่อยๆ	ทำได้บ่อยๆ	ทำได้เป็นบ	ทำได้เป็นบ
ไม่เห็นด้วย	ไม่เห็นด้วย	ไม่เห็นด้วย	16.00	ทำเป็นประจ	ทำเป็นประจ	ทำเป็นประจ	ทำเป็นประจ	ทำได้บ่อยๆ
ไม่เห็นด้วย	ไม่เห็นด้วย	ไม่เห็นด้วยอ	17.00	ทำได้บ้าง/ไ	ทำเป็นประจ	ทำเป็นประจ	ทำเป็นประจ	ทำเป็นประจ
ไม่เห็นด้วย	เห็นด้วย	เห็นด้วย	16.00	ทำได้บ่อยๆ	ทำเป็นประจ	ทำเป็นประจ	ทำได้บ่อยๆ	ทำได้บ่อยๆ
ไม่เห็นด้วย	ไม่เห็นด้วย	ไม่เห็นด้วย	12.00	ทำเป็นประจ	ทำเป็นประจ	ทำเป็นประจ	ทำเป็นประจ	ทำเป็นประจ
ไม่เห็นด้วย	ไม่เห็นด้วย	เห็นด้วย	17.00	ทำได้บ้าง/ไ	ทำได้เป็นบ	ทำเป็นประจ	ทำได้บ้าง/ไ	ไม่เคยทำเล
เห็นด้วย	เห็นด้วย	เห็นด้วย	21.00	ทำได้บ้าง/ไ	ทำได้เป็นบ	ไม่เคยทำเล	ไม่เคยทำเล	ไม่เคยทำเล
เห็นด้วย	เห็นด้วย	เห็นด้วย	18.00	ทำได้บ่อยๆ	ทำได้บ่อยๆ	ทำเป็นประจ	ทำได้เป็นบ	ทำได้เป็นบ
เห็นด้วย	เห็นด้วย	เห็นด้วย	16.00	ทำได้เป็นบ	ทำได้เป็นบ	ทำได้บ้าง/ไ	ไม่เคยทำเล	ทำได้เป็นบ
เห็นด้วย	ไม่เห็นด้วย	เห็นด้วย	18.00	ทำได้บ่อยๆ	ทำได้เป็นบ	ทำได้บ้าง/ไ	ทำเป็นประจ	ทำได้เป็นบ
ไม่เห็นด้วยอ	ไม่เห็นด้วย	ไม่เห็นด้วย	16.00	ทำได้บ่อยๆ	ทำได้บ่อยๆ	ทำเป็นประจ	ทำเป็นประจ	ทำเป็นประจ
ไม่เห็นด้วย	ไม่เห็นด้วย	ไม่เห็นด้วย	16.00	ทำเป็นประจ	ทำเป็นประจ	ทำเป็นประจ	ทำเป็นประจ	ทำเป็นประจ
เห็นด้วย	ไม่เห็นด้วย	เห็นด้วย	14.00	ทำได้เป็นบ	ทำเป็นประจ	ทำได้เป็นบ	ทำได้เป็นบ	ทำได้บ่อยๆ
เห็นด้วย	ไม่เห็นด้วย	เห็นด้วย	17.00	ทำได้เป็นบ	ทำได้เป็นบ	ทำได้บ้าง/ไ	ไม่เคยทำเล	ทำได้บ้าง/ไ
เห็นด้วย	เห็นด้วย	เห็นด้วย	13.00	ทำได้บ้าง/ไ	ทำได้เป็นบ	ไม่เคยทำเล	ไม่เคยทำเล	ทำเป็นประจ
ไม่เห็นด้วยอ	เห็นด้วยอย่	เห็นด้วยอย่	21.00	ทำได้เป็นบ	ทำได้เป็นบ	ทำได้บ้าง/ไ	ทำได้เป็นบ	ไม่เคยทำเล
ไม่เห็นด้วย	ไม่เห็นด้วย	ไม่เห็นด้วย	14.00	ทำได้บ่อยๆ	ทำได้บ่อยๆ	ทำได้บ่อยๆ	ทำได้บ้าง/ไ	ไม่เคยทำเล
ไม่เห็นด้วย	ไม่เห็นด้วย	ไม่เห็นด้วย	19.00	ทำได้เป็นบ	ทำได้บ่อยๆ	ทำได้เป็นบ	ทำได้บ่อยๆ	ทำได้บ้าง/ไ
ไม่เห็นด้วย	ไม่เห็นด้วย	ไม่เห็นด้วย	18.00	ทำได้บ่อยๆ	ทำได้เป็นบ	ทำได้บ่อยๆ	ทำเป็นประจ	ทำได้บ่อยๆ
เห็นด้วยอย่	เห็นด้วย	ไม่เห็นด้วย	15.00	ทำเป็นประจ	ทำได้บ่อยๆ	ทำเป็นประจ	ทำได้บ่อยๆ	ทำเป็นประจ
เห็นด้วย	เห็นด้วย	ไม่เห็นด้วย	19.00	ทำเป็นประจ	ทำได้เป็นบ	ทำเป็นประจ	ทำเป็นประจ	ทำเป็นประจ
ไม่เห็นด้วย	ไม่เห็นด้วย	ไม่เห็นด้วยอ	12.00	ทำเป็นประจ	ทำเป็นประจ	ทำเป็นประจ	ทำเป็นประจ	ทำได้บ้าง/ไ
เห็นด้วยอย่	เห็นด้วย	เห็นด้วยอย่	15.00	ทำได้เป็นบ	ทำได้บ้าง/ไ	ทำได้เป็นบ	ทำได้บ่อยๆ	ทำเป็นประจ
ไม่เห็นด้วย	ไม่เห็นด้วย	ไม่เห็นด้วย	18.00	ทำได้บ่อยๆ	ทำได้เป็นบ	ทำได้บ่อยๆ	ทำได้เป็นบ	ทำได้บ่อยๆ
ไม่เห็นด้วย	ไม่เห็นด้วยอ	ไม่เห็นด้วย	17.00	ทำได้บ้าง/ไ	ทำเป็นประจ	ทำเป็นประจ	ทำเป็นประจ	ทำได้บ่อยๆ
ไม่เห็นด้วย	ไม่เห็นด้วย	ไม่เห็นด้วย	9.00	ทำเป็นประจ	ทำเป็นประจ	ทำเป็นประจ	ทำเป็นประจ	ทำเป็นประจ
ไม่เห็นด้วย	ไม่เห็นด้วย	ไม่เห็นด้วย	16.00	ทำได้เป็นบ	ทำได้บ่อยๆ	ทำได้บ่อยๆ	ทำได้บ้าง/ไ	ไม่เคยทำเล
ไม่เห็นด้วย	ไม่เห็นด้วย	เห็นด้วย	15.00	ทำได้บ่อยๆ	ทำได้บ่อยๆ	ทำได้เป็นบ	ทำเป็นประจ	ทำได้บ้าง/ไ
ไม่เห็นด้วยอ	ไม่เห็นด้วยอ	ไม่เห็นด้วยอ	14.00	ทำได้เป็นบ	ทำเป็นประจ	ทำได้บ่อยๆ	ทำเป็นประจ	ทำเป็นประจ
เห็นด้วย	ไม่เห็นด้วย	ไม่เห็นด้วย	17.00	ทำได้บ่อยๆ	ทำได้เป็นบ	ทำได้เป็นบ	ทำได้เป็นบ	ทำได้เป็นบ
เห็นด้วย	เห็นด้วย	เห็นด้วยอย่	19.00	ทำได้บ้าง/ไ	ทำได้บ่อยๆ	ทำได้บ่อยๆ	ทำได้เป็นบ	ทำได้เป็นบ
ไม่เห็นด้วย	ไม่เห็นด้วย	ไม่เห็นด้วย	16.00	ทำเป็นประจ	ทำเป็นประจ	ทำเป็นประจ	ทำเป็นประจ	ทำได้บ่อยๆ
ไม่เห็นด้วย	ไม่เห็นด้วยอ	ไม่เห็นด้วยอ	16.00	ทำเป็นประจ	ทำเป็นประจ	ทำเป็นประจ	ทำเป็นประจ	ทำเป็นประจ
ไม่เห็นด้วย	เห็นด้วย	เห็นด้วย	16.00	ทำได้บ่อยๆ	ทำเป็นประจ	ทำเป็นประจ	ทำได้บ่อยๆ	ทำได้บ่อยๆ
ไม่เห็นด้วย	ไม่เห็นด้วย	ไม่เห็นด้วย	16.00	ทำเป็นประจ	ทำเป็นประจ	ทำเป็นประจ	ทำเป็นประจ	ทำเป็นประจ
ไม่เห็นด้วย	ไม่เห็นด้วย	เห็นด้วย	18.00	ทำได้บ้าง/ไ	ทำได้เป็นบ	ทำเป็นประจ	ทำได้บ้าง/ไ	ไม่เคยทำเล
เห็นด้วย	เห็นด้วย	เห็นด้วย	18.00	ทำได้บ้าง/ไ	ทำได้เป็นบ	ไม่เคยทำเล	ไม่เคยทำเล	ไม่เคยทำเล
เห็นด้วย	เห็นด้วย	เห็นด้วย	16.00	ทำได้บ่อยๆ	ทำได้บ่อยๆ	ทำเป็นประจ	ทำได้เป็นบ	ทำได้เป็นบ
เห็นด้วย	เห็นด้วย	เห็นด้วย	12.00	ทำได้เป็นบ	ทำได้เป็นบ	ทำได้บ้าง/ไ	ไม่เคยทำเล	ทำได้เป็นบ
เห็นด้วย	ไม่เห็นด้วย	เห็นด้วย	18.00	ทำได้บ่อยๆ	ทำได้เป็นบ	ทำได้บ้าง/ไ	ทำเป็นประจ	ทำได้เป็นบ
ไม่เห็นด้วยอ	ไม่เห็นด้วย	ไม่เห็นด้วย	12.00	ทำได้บ่อยๆ	ทำได้บ่อยๆ	ทำเป็นประจ	ทำเป็นประจ	ทำเป็นประจ
ไม่เห็นด้วย	ไม่เห็นด้วย	ไม่เห็นด้วย	15.00	ทำเป็นประจ	ทำเป็นประจ	ทำเป็นประจ	ทำเป็นประจ	ทำเป็นประจ
เห็นด้วย	ไม่เห็นด้วย	เห็นด้วย	18.00	ทำได้เป็นบ	ทำเป็นประจ	ทำได้เป็นบ	ทำได้เป็นบ	ทำได้บ่อยๆ
เห็นด้วย	ไม่เห็นด้วย	เห็นด้วย	14.00	ทำได้เป็นบ	ทำได้เป็นบ	ทำได้บ้าง/ไ	ไม่เคยทำเล	ทำได้บ้าง/ไ
เห็นด้วย	เห็นด้วย	เห็นด้วย	18.00	ทำได้บ้าง/ไ	ทำได้เป็นบ	ไม่เคยทำเล	ไม่เคยทำเล	ทำเป็นประจ

ไม่เห็นด้วย	เห็นด้วย	เห็นด้วย	15.00	ทำได้เป็นบ	ทำได้เป็นบ	ทำได้บ้าง/ไ	ทำได้เป็นบ	ไม่เคยทำเล
ไม่เห็นด้วย	ไม่เห็นด้วย	ไม่เห็นด้วย	14.00	ทำได้บ่อยๆ	ทำได้บ่อยๆ	ทำได้บ่อยๆ	ทำได้บ้าง/ไ	ไม่เคยทำเล
ไม่เห็นด้วย	ไม่เห็นด้วย	ไม่เห็นด้วย	18.00	ทำได้เป็นบ	ทำได้บ่อยๆ	ทำได้เป็นบ	ทำได้บ่อยๆ	ทำได้บ้าง/ไ
ไม่เห็นด้วย	ไม่เห็นด้วย	ไม่เห็นด้วย	21.00	ทำได้บ่อยๆ	ทำได้เป็นบ	ทำได้บ่อยๆ	ทำเป็นประจ	ทำได้บ่อยๆ
เห็นด้วยอย่	เห็นด้วย	ไม่เห็นด้วย	14.00	ทำเป็นประจ	ทำได้บ่อยๆ	ทำเป็นประจ	ทำได้บ่อยๆ	ทำเป็นประจ
ไม่เห็นด้วย	ไม่เห็นด้วย	เห็นด้วย	18.00	ทำได้เป็นบ	ทำได้เป็นบ	ทำได้เป็นบ	ทำได้เป็นบ	ทำได้เป็นบ
ไม่เห็นด้วย	ไม่เห็นด้วย	ไม่เห็นด้วย	12.00	ทำได้บ่อยๆ	ทำเป็นประจ	ทำเป็นประจ	ทำเป็นประจ	ทำเป็นประจ
ไม่เห็นด้วย	ไม่เห็นด้วย	ไม่เห็นด้วย	18.00	ทำได้บ่อยๆ	ทำได้บ่อยๆ	ทำได้บ่อยๆ	ทำได้บ่อยๆ	ทำได้บ่อยๆ
ไม่เห็นด้วย	เห็นด้วย	ไม่เห็นด้วย	15.00	ทำได้เป็นบ	ทำได้เป็นบ	ทำได้เป็นบ	ทำได้เป็นบ	ทำได้บ่อยๆ
ไม่เห็นด้วย	ไม่เห็นด้วย	ไม่เห็นด้วย	13.00	ทำได้เป็นบ	ทำได้บ่อยๆ	ทำได้บ่อยๆ	ทำได้บ้าง/ไ	ไม่เคยทำเล
ไม่เห็นด้วย	ไม่เห็นด้วย	เห็นด้วย	14.00	ทำได้บ่อยๆ	ทำได้บ่อยๆ	ทำได้เป็นบ	ทำเป็นประจ	ทำได้บ้าง/ไ
ไม่เห็นด้วยอ	ไม่เห็นด้วยอ	ไม่เห็นด้วยอ	12.00	ทำได้เป็นบ	ทำเป็นประจ	ทำได้บ่อยๆ	ทำเป็นประจ	ทำเป็นประจ
เห็นด้วย	ไม่เห็นด้วย	ไม่เห็นด้วย	18.00	ทำได้บ่อยๆ	ทำได้เป็นบ	ทำได้เป็นบ	ทำได้เป็นบ	ทำได้เป็นบ
เห็นด้วย	เห็นด้วย	เห็นด้วยอย่	17.00	ทำได้บ้าง/ไ	ทำได้บ่อยๆ	ทำได้บ่อยๆ	ทำได้เป็นบ	ทำได้เป็นบ
ไม่เห็นด้วย	ไม่เห็นด้วย	ไม่เห็นด้วย	17.00	ทำเป็นประจ	ทำเป็นประจ	ทำเป็นประจ	ทำเป็นประจ	ทำได้บ่อยๆ
ไม่เห็นด้วย	ไม่เห็นด้วยอ	ไม่เห็นด้วยอ	17.00	ทำเป็นประจ	ทำเป็นประจ	ทำเป็นประจ	ทำเป็นประจ	ทำเป็นประจ
ไม่เห็นด้วย	เห็นด้วย	เห็นด้วย	16.00	ทำได้บ่อยๆ	ทำเป็นประจ	ทำเป็นประจ	ทำได้บ่อยๆ	ทำได้บ่อยๆ
ไม่เห็นด้วย	ไม่เห็นด้วย	ไม่เห็นด้วย	18.00	ทำเป็นประจ	ทำเป็นประจ	ทำเป็นประจ	ทำเป็นประจ	ทำเป็นประจ
ไม่เห็นด้วย	ไม่เห็นด้วย	เห็นด้วย	11.00	ทำได้บ้าง/ไ	ทำได้เป็นบ	ทำเป็นประจ	ทำได้บ้าง/ไ	ไม่เคยทำเล
เห็นด้วย	เห็นด้วย	เห็นด้วย	15.00	ทำได้บ้าง/ไ	ทำได้เป็นบ	ไม่เคยทำเล	ไม่เคยทำเล	ทำได้บ้าง/ไ
เห็นด้วย	เห็นด้วย	เห็นด้วย	8.00	ทำได้บ่อยๆ	ทำได้บ่อยๆ	ทำเป็นประจ	ทำได้เป็นบ	ทำได้เป็นบ
เห็นด้วย	เห็นด้วย	เห็นด้วย	18.00	ทำได้เป็นบ	ทำได้เป็นบ	ทำได้บ้าง/ไ	ไม่เคยทำเล	ทำได้เป็นบ
เห็นด้วย	ไม่เห็นด้วย	เห็นด้วย	13.00	ทำได้บ่อยๆ	ทำได้เป็นบ	ทำได้บ้าง/ไ	ทำเป็นประจ	ทำได้เป็นบ
ไม่เห็นด้วยอ	ไม่เห็นด้วย	ไม่เห็นด้วย	15.00	ทำได้บ่อยๆ	ทำได้บ่อยๆ	ทำเป็นประจ	ทำเป็นประจ	ทำเป็นประจ
ไม่เห็นด้วย	ไม่เห็นด้วย	เห็นด้วย	18.00	ทำได้เป็นบ	ทำเป็นประจ	ทำได้บ่อยๆ	ทำได้บ่อยๆ	ทำได้บ่อยๆ
เห็นด้วย	ไม่เห็นด้วย	ไม่เห็นด้วย	17.00	ไม่เคยทำเล	ทำได้เป็นบ	ทำได้เป็นบ	ทำได้บ่อยๆ	ทำได้เป็นบ
ไม่เห็นด้วยอ	ไม่เห็นด้วยอ	ไม่เห็นด้วยอ	18.00	ทำได้บ่อยๆ	ทำเป็นประจ	ทำเป็นประจ	ทำเป็นประจ	ทำเป็นประจ
เห็นด้วย	เห็นด้วย	เห็นด้วย	17.00	ไม่เคยทำเล	ทำได้บ้าง/ไ	ทำได้บ้าง/ไ	ทำได้เป็นบ	ทำได้เป็นบ
ไม่เห็นด้วย	ไม่เห็นด้วย	ไม่เห็นด้วย	18.00	ทำได้เป็นบ	ทำได้บ่อยๆ	ทำได้บ่อยๆ	ทำได้บ่อยๆ	ทำได้บ่อยๆ
เห็นด้วย	เห็นด้วย	เห็นด้วย	24.00	ไม่เคยทำเล	ทำได้เป็นบ	ทำได้เป็นบ	ทำได้เป็นบ	ทำได้เป็นบ
ไม่เห็นด้วยอ	ไม่เห็นด้วยอ	ไม่เห็นด้วยอ	15.00	ทำได้เป็นบ	ทำเป็นประจ	ทำเป็นประจ	ทำเป็นประจ	ทำเป็นประจ
ไม่เห็นด้วยอ	ไม่เห็นด้วยอ	ไม่เห็นด้วยอ	24.00	ทำได้บ่อยๆ	ทำได้บ่อยๆ	ทำได้บ่อยๆ	ทำได้บ่อยๆ	ทำได้บ้าง/ไ
ไม่เห็นด้วย	ไม่เห็นด้วย	ไม่เห็นด้วย	16.00	ทำได้บ้าง/ไ	ทำได้บ่อยๆ	ทำเป็นประจ	ทำเป็นประจ	ทำเป็นประจ
เห็นด้วย	เห็นด้วย	เห็นด้วย	18.00	ไม่เคยทำเล	ทำได้บ้าง/ไ	ทำได้เป็นบ	ทำได้เป็นบ	ทำได้เป็นบ
ไม่เห็นด้วย	ไม่เห็นด้วย	ไม่เห็นด้วย	11.00	ทำเป็นประจ	ทำเป็นประจ	ทำเป็นประจ	ทำเป็นประจ	ทำเป็นประจ
ไม่เห็นด้วย	ไม่เห็นด้วย	ไม่เห็นด้วย	12.00	ทำได้บ่อยๆ	ทำได้เป็นบ	ทำได้บ่อยๆ	ทำได้บ่อยๆ	ทำได้บ่อยๆ
ไม่เห็นด้วยอ	ไม่เห็นด้วยอ	ไม่เห็นด้วยอ	12.00	ทำเป็นประจ	ทำเป็นประจ	ทำเป็นประจ	ทำเป็นประจ	ทำเป็นประจ
ไม่เห็นด้วยอ	ไม่เห็นด้วยอ	ไม่เห็นด้วยอ	14.00	ทำได้บ่อยๆ	ทำได้เป็นบ	ทำได้บ่อยๆ	ทำเป็นประจ	ทำเป็นประจ
เห็นด้วย	ไม่เห็นด้วย	ไม่เห็นด้วย	14.00	ทำได้บ้าง/ไ	ทำเป็นประจ	ทำได้บ่อยๆ	ทำได้บ่อยๆ	ทำได้บ่อยๆ
ไม่เห็นด้วยอ	ไม่เห็นด้วยอ	ไม่เห็นด้วยอ	17.00	ทำเป็นประจ	ทำเป็นประจ	ทำเป็นประจ	ทำเป็นประจ	ทำเป็นประจ
ไม่เห็นด้วย	ไม่เห็นด้วย	ไม่เห็นด้วย	17.00	ทำได้บ้าง/ไ	ทำได้เป็นบ	ทำได้บ่อยๆ	ทำได้บ่อยๆ	ทำได้บ่อยๆ
ไม่เห็นด้วย	ไม่เห็นด้วย	ไม่เห็นด้วย	15.00	ไม่เคยทำเล	ทำได้บ่อยๆ	ทำได้บ่อยๆ	ทำได้บ่อยๆ	ทำได้บ่อยๆ
ไม่เห็นด้วยอ	ไม่เห็นด้วยอ	ไม่เห็นด้วยอ	17.00	ทำเป็นประจ	ทำเป็นประจ	ทำเป็นประจ	ทำเป็นประจ	ทำเป็นประจ
ไม่เห็นด้วย	ไม่เห็นด้วย	ไม่เห็นด้วย	16.00	ทำเป็นประจ	ทำเป็นประจ	ทำเป็นประจ	ทำเป็นประจ	ทำเป็นประจ
ไม่เห็นด้วย	ไม่เห็นด้วย	ไม่เห็นด้วย	14.00	ไม่เคยทำเล	ทำได้บ่อยๆ	ทำได้บ่อยๆ	ทำได้บ่อยๆ	ทำได้บ่อยๆ
ไม่เห็นด้วย	ไม่เห็นด้วย	ไม่เห็นด้วย	24.00	ไม่เคยทำเล	ทำได้เป็นบ	ทำได้เป็นบ	ทำได้เป็นบ	ทำได้เป็นบ
ไม่เห็นด้วยอ	ไม่เห็นด้วยอ	ไม่เห็นด้วยอ	18.00	ทำได้เป็นบ	ทำได้บ่อยๆ	ทำได้บ่อยๆ	ทำได้บ่อยๆ	ทำได้บ่อยๆ
ไม่เห็นด้วย	ไม่เห็นด้วย	ไม่เห็นด้วย	18.00	ไม่เคยทำเล	ทำได้บ้าง/ไ	ทำได้เป็นบ	ทำได้เป็นบ	ทำได้เป็นบ
เห็นด้วย	ไม่เห็นด้วย	ไม่เห็นด้วย	18.00	ทำได้เป็นบ	ทำได้บ่อยๆ	ทำได้บ่อยๆ	ทำได้บ่อยๆ	ทำได้เป็นบ
ไม่เห็นด้วย	ไม่เห็นด้วย	ไม่เห็นด้วย	18.00	ทำได้เป็นบ	ทำได้เป็นบ	ทำได้เป็นบ	ทำได้เป็นบ	ทำได้บ้าง/ไ
ไม่เห็นด้วย	ไม่เห็นด้วย	ไม่เห็นด้วย	17.00	ทำได้บ่อยๆ	ทำได้บ่อยๆ	ทำเป็นประจ	ทำเป็นประจ	ทำเป็นประจ

เห็นด้วย	เห็นด้วย	เห็นด้วย	14.00	ทำได้เป็นบ	ทำได้เป็นบ	ทำได้บ้าง/	ทำได้เป็นบ	ทำได้เป็นบ
ไม่เห็นด้วย	เห็นด้วย	เห็นด้วย	17.00	ไม่เคยทำเ	ไม่เคยทำเ	ไม่เคยทำเ	ไม่เคยทำเ	ไม่เคยทำเ
ไม่เห็นด้วยอ	ไม่เห็นด้วยอ	ไม่เห็นด้วยอ	12.00	ทำได้บ่อยๆ	ทำได้บ่อยๆ	ทำได้เป็นบ	ทำได้บ่อยๆ	ทำได้บ่อยๆ
ไม่เห็นด้วย	ไม่เห็นด้วยอ	ไม่เห็นด้วย	18.00	ทำได้บ่อยๆ	ทำได้เป็นบ	ทำได้เป็นบ	ทำได้บ่อยๆ	ทำเป็นประ
ไม่เห็นด้วย	ไม่เห็นด้วย	ไม่เห็นด้วย	15.00	ทำได้บ้าง/	ทำได้เป็นบ	ทำได้บ่อยๆ	ทำได้เป็นบ	ทำได้บ่อยๆ
ไม่เห็นด้วย	เห็นด้วย	ไม่เห็นด้วย	16.00	ทำเป็นประ	ทำเป็นประ	ทำได้เป็นบ	ทำได้บ่อยๆ	ทำได้บ่อยๆ
ไม่เห็นด้วย	ไม่เห็นด้วย	ไม่เห็นด้วย	19.00	ทำได้เป็นบ	ทำได้บ่อยๆ	ไม่เคยทำเ	ไม่เคยทำเ	ทำเป็นประ
ไม่เห็นด้วย	ไม่เห็นด้วย	ไม่เห็นด้วย	11.00	ทำได้บ่อยๆ	ทำได้บ่อยๆ	ทำได้เป็นบ	ทำได้เป็นบ	ทำได้เป็นบ
ไม่เห็นด้วย	ไม่เห็นด้วย	ไม่เห็นด้วย	15.00	ทำได้เป็นบ	ทำเป็นประ	ทำได้เป็นบ	ทำได้เป็นบ	ทำได้บ่อยๆ
เห็นด้วย	ไม่เห็นด้วย	ไม่เห็นด้วย	10.00	ทำเป็นประ	ทำเป็นประ	ทำเป็นประ	ทำเป็นประ	ทำเป็นประ
ไม่เห็นด้วย	ไม่เห็นด้วย	ไม่เห็นด้วย	11.00	ทำได้บ่อยๆ	ทำได้บ่อยๆ	ทำได้เป็นบ	ทำได้เป็นบ	ทำได้เป็นบ
ไม่เห็นด้วย	ไม่เห็นด้วย	ไม่เห็นด้วย	11.00	ทำได้เป็นบ	ทำได้บ่อยๆ	ทำได้บ้าง/	ทำได้เป็นบ	ทำได้บ่อยๆ
ไม่เห็นด้วย	เห็นด้วย	ไม่เห็นด้วย	18.00	ทำได้บ่อยๆ	ทำได้เป็นบ	ทำได้บ่อยๆ	ทำได้บ่อยๆ	ทำได้บ่อยๆ
เห็นด้วย	ไม่เห็นด้วย	เห็นด้วย	17.00	ทำเป็นประ	ทำเป็นประ	ทำได้บ่อยๆ	ทำเป็นประ	ทำเป็นประ
ไม่เห็นด้วย	ไม่เห็นด้วย	ไม่เห็นด้วย	13.00	ทำได้บ่อยๆ	ทำเป็นประ	ทำได้บ่อยๆ	ทำเป็นประ	ทำได้บ่อยๆ
ไม่เห็นด้วยอ	เห็นด้วยอย่	เห็นด้วยอย่	19.00	ทำได้บ่อยๆ	ทำได้บ่อยๆ	ทำได้เป็นบ	ทำได้บ่อยๆ	ทำได้บ่อยๆ
เห็นด้วย	เห็นด้วย	ไม่เห็นด้วย	17.00	ทำเป็นประ	ทำได้บ่อยๆ	ทำเป็นประ	ทำเป็นประ	ทำเป็นประ
ไม่เห็นด้วย	ไม่เห็นด้วย	เห็นด้วย	18.00	ทำได้บ่อยๆ	ทำได้เป็นบ	ทำได้บ่อยๆ	ทำได้บ่อยๆ	ทำได้บ่อยๆ
ไม่เห็นด้วย	ไม่เห็นด้วย	ไม่เห็นด้วย	24.00	ทำได้บ่อยๆ	ทำเป็นประ	ทำได้เป็นบ	ทำเป็นประ	ทำเป็นประ
เห็นด้วย	เห็นด้วย	เห็นด้วย	12.00	ทำได้บ่อยๆ	ทำเป็นประ	ทำได้บ่อยๆ	ทำได้บ่อยๆ	ทำได้บ่อยๆ
ไม่เห็นด้วย	ไม่เห็นด้วย	ไม่เห็นด้วย	17.00	ทำได้บ้าง/	ทำได้บ่อยๆ	ทำได้บ่อยๆ	ทำเป็นประ	ทำได้บ่อยๆ
ไม่เห็นด้วย	ไม่เห็นด้วย	ไม่เห็นด้วย	8.00	ทำเป็นประ	ทำเป็นประ	ทำเป็นประ	ทำเป็นประ	ทำเป็นประ
ไม่เห็นด้วย	ไม่เห็นด้วย	ไม่เห็นด้วย	10.00	ทำได้บ่อยๆ	ทำเป็นประ	ทำได้เป็นบ	ทำได้เป็นบ	ทำได้เป็นบ
เห็นด้วย	เห็นด้วย	ไม่เห็นด้วย	7.00	ทำได้บ้าง/	ทำเป็นประ	ทำได้บ้าง/	ทำได้บ้าง/	ไม่เคยทำเ
ไม่เห็นด้วยอ	ไม่เห็นด้วยอ	ไม่เห็นด้วยอ	17.00	ทำได้เป็นบ	ทำได้บ่อยๆ	ทำได้บ่อยๆ	ทำเป็นประ	ทำเป็นประ
ไม่เห็นด้วย	ไม่เห็นด้วย	ไม่เห็นด้วย	14.00	ทำได้บ่อยๆ	ทำเป็นประ	ทำได้บ่อยๆ	ทำเป็นประ	ทำเป็นประ
เห็นด้วย	ไม่เห็นด้วย	ไม่เห็นด้วย	15.00	ทำได้เป็นบ	ทำได้บ้าง/	ไม่เคยทำเ	ทำได้เป็นบ	ไม่เคยทำเ
ไม่เห็นด้วย	ไม่เห็นด้วย	ไม่เห็นด้วย	15.00	ทำได้บ่อยๆ	ทำเป็นประ	ทำเป็นประ	ทำได้เป็นบ	ทำได้เป็นบ
ไม่เห็นด้วยอ	ไม่เห็นด้วย	ไม่เห็นด้วย	16.00	ทำได้บ่อยๆ	ทำเป็นประ	ทำได้บ้าง/	ทำเป็นประ	ทำได้เป็นบ
เห็นด้วย	ไม่เห็นด้วย	ไม่เห็นด้วย	14.00	ทำได้เป็นบ	ทำได้บ้าง/	ไม่เคยทำเ	ทำได้บ่อยๆ	ทำได้บ่อยๆ
เห็นด้วยอย่	ไม่เห็นด้วย	ไม่เห็นด้วย	15.00	ทำได้บ่อยๆ	ทำได้เป็นบ	ทำได้บ้าง/	ทำเป็นประ	ทำเป็นประ
ไม่เห็นด้วย	ไม่เห็นด้วย	ไม่เห็นด้วย	15.00	ทำเป็นประ	ทำเป็นประ	ทำเป็นประ	ทำเป็นประ	ทำเป็นประ
เห็นด้วย	ไม่เห็นด้วย	ไม่เห็นด้วย	19.00	ทำได้เป็นบ	ทำได้เป็นบ	ทำได้เป็นบ	ทำได้บ้าง/	ทำได้เป็นบ
ไม่เห็นด้วย	ไม่เห็นด้วยอ	ไม่เห็นด้วย	12.00	ทำได้บ่อยๆ	ทำเป็นประ	ทำได้บ่อยๆ	ทำได้บ่อยๆ	ทำเป็นประ
ไม่เห็นด้วย	ไม่เห็นด้วย	ไม่เห็นด้วย	16.00	ทำได้บ่อยๆ	ทำได้บ่อยๆ	ทำได้บ่อยๆ	ทำได้บ่อยๆ	ทำได้บ่อยๆ
เห็นด้วย	ไม่เห็นด้วย	ไม่เห็นด้วย	16.00	ทำได้บ่อยๆ	ทำเป็นประ	ทำได้เป็นบ	ทำได้บ่อยๆ	ทำเป็นประ
ไม่เห็นด้วย	ไม่เห็นด้วยอ	ไม่เห็นด้วย	14.00	ทำเป็นประ	ทำเป็นประ	ทำได้บ่อยๆ	ทำได้บ่อยๆ	ทำเป็นประ
ไม่เห็นด้วย	ไม่เห็นด้วย	ไม่เห็นด้วย	17.00	ทำได้เป็นบ	ทำได้เป็นบ	ทำเป็นประ	ทำเป็นประ	ทำเป็นประ
ไม่เห็นด้วย	เห็นด้วย	ไม่เห็นด้วย	18.00	ทำได้เป็นบ	ทำได้บ่อยๆ	ทำได้บ่อยๆ	ทำได้บ่อยๆ	ทำได้เป็นบ
เห็นด้วย	ไม่เห็นด้วย	ไม่เห็นด้วย	16.00	ทำได้เป็นบ	ทำได้บ่อยๆ	ทำได้บ่อยๆ	ทำได้บ่อยๆ	ทำได้เป็นบ
ไม่เห็นด้วย	ไม่เห็นด้วย	ไม่เห็นด้วย	15.00	ทำได้เป็นบ	ทำได้เป็นบ	ทำได้เป็นบ	ทำได้เป็นบ	ทำได้บ้าง/
ไม่เห็นด้วย	ไม่เห็นด้วย	ไม่เห็นด้วย	17.00	ทำได้บ่อยๆ	ทำได้บ่อยๆ	ทำเป็นประ	ทำเป็นประ	ทำเป็นประ
เห็นด้วย	เห็นด้วย	เห็นด้วย	14.00	ทำได้เป็นบ	ทำได้เป็นบ	ทำได้บ้าง/	ทำได้เป็นบ	ทำได้เป็นบ
ไม่เห็นด้วย	เห็นด้วย	เห็นด้วย	18.00	ไม่เคยทำเ	ไม่เคยทำเ	ไม่เคยทำเ	ไม่เคยทำเ	ไม่เคยทำเ
ไม่เห็นด้วยอ	ไม่เห็นด้วยอ	ไม่เห็นด้วยอ	16.00	ทำได้บ่อยๆ	ทำได้บ่อยๆ	ทำได้เป็นบ	ทำได้บ่อยๆ	ทำได้บ่อยๆ
ไม่เห็นด้วย	ไม่เห็นด้วยอ	ไม่เห็นด้วย	18.00	ทำได้บ่อยๆ	ทำได้เป็นบ	ทำได้เป็นบ	ทำได้บ่อยๆ	ทำเป็นประ
ไม่เห็นด้วย	เห็นด้วย	เห็นด้วย	17.00	ทำได้เป็นบ	ทำเป็นประ	ไม่เคยทำเ	ทำได้เป็นบ	ทำได้บ้าง/
ไม่เห็นด้วย	ไม่เห็นด้วย	ไม่เห็นด้วย	17.00	ทำได้บ่อยๆ	ทำเป็นประ	ทำได้บ่อยๆ	ทำได้บ่อยๆ	ทำเป็นประ
ไม่เห็นด้วย	ไม่เห็นด้วย	ไม่เห็นด้วย	15.00	ทำได้บ่อยๆ	ทำได้บ่อยๆ	ทำได้บ่อยๆ	ทำได้บ่อยๆ	ทำเป็นประ

HeLD6	HeLD7	HeLD8	HeLD9	HeLD10	HeLD11	HeLD12	HeLD13	HeLD14
ทำได้บ่อยๆ	ทำได้บ่อยๆ	ทำได้บ่อยๆ	ทำเป็นประ	ทำเป็นประ	ทำได้เป็นบ	ทำได้บ่อยๆ	ทำได้บ่อยๆ	ทำได้บ่อยๆ
ทำเป็นประ	ทำได้บ่อยๆ	ทำได้เป็นบ	ทำได้บ่อยๆ	ทำได้บ่อยๆ	ทำเป็นประ	ทำได้บ่อยๆ	ทำเป็นประ	ทำเป็นประ
ทำเป็นประ	ทำได้บ้าง/	ทำได้บ่อยๆ	ทำเป็นประ	ทำได้บ้าง/	ทำได้เป็นบ	ทำได้บ่อยๆ	ทำเป็นประ	ทำได้บ่อยๆ
ทำเป็นประ	ทำเป็นประ	ทำได้บ่อยๆ	ทำเป็นประ	ทำเป็นประ	ทำได้บ่อยๆ	ทำได้บ่อยๆ	ทำได้บ่อยๆ	ทำได้บ่อยๆ
ทำได้บ้าง/	ทำได้เป็นบ	ทำได้เป็นบ	ทำได้บ่อยๆ	ทำได้บ่อยๆ	ไม่เคยทำเ	ทำได้บ้าง/	ทำได้เป็นบ	ทำได้บ่อยๆ
ทำเป็นประ	ทำได้บ้าง/	ทำได้บ้าง/	ทำได้บ่อยๆ	ทำได้บ่อยๆ	ทำได้เป็นบ	ทำได้บ้าง/	ทำเป็นประ	ทำเป็นประ
ทำได้เป็นบ	ทำได้เป็นบ	ทำได้เป็นบ	ทำได้บ้าง/	ทำได้เป็นบ	ไม่เคยทำเ	ไม่เคยทำเ	ไม่เคยทำเ	ทำได้บ้าง/
ทำได้บ่อยๆ	ทำเป็นประ	ทำเป็นประ	ทำเป็นประ	ทำเป็นประ	ทำเป็นประ	ทำเป็นประ	ทำเป็นประ	ทำเป็นประ
ทำได้เป็นบ	ทำได้เป็นบ	ทำได้เป็นบ	ทำได้เป็นบ	ทำได้เป็นบ	ทำได้เป็นบ	ทำได้เป็นบ	ทำได้เป็นบ	ทำได้เป็นบ
ทำเป็นประ	ทำได้บ่อยๆ	ทำเป็นประ	ทำได้บ่อยๆ	ทำได้บ่อยๆ	ทำได้บ่อยๆ	ทำได้เป็นบ	ทำเป็นประ	ทำเป็นประ
ทำได้เป็นบ	ทำได้เป็นบ	ทำได้เป็นบ	ทำได้บ่อยๆ	ทำได้บ่อยๆ	ทำได้เป็นบ	ทำได้เป็นบ	ทำได้บ่อยๆ	ทำได้บ่อยๆ
ทำเป็นประ	ทำเป็นประ	ทำได้บ่อยๆ	ทำได้บ่อยๆ	ทำเป็นประ	ทำได้บ่อยๆ	ทำเป็นประ	ทำเป็นประ	ทำได้บ่อยๆ
ทำได้บ่อยๆ	ไม่เคยทำเ	ทำได้บ่อยๆ	ทำได้เป็นบ	ทำได้เป็นบ	ทำได้เป็นบ	ทำได้เป็นบ	ทำได้บ่อยๆ	ทำได้เป็นบ
ทำเป็นประ	ทำเป็นประ	ทำได้บ่อยๆ	ทำได้บ่อยๆ	ทำได้บ่อยๆ	ทำได้บ่อยๆ	ทำได้บ่อยๆ	ทำได้บ่อยๆ	ทำได้บ่อยๆ
ทำได้บ่อยๆ	ไม่เคยทำเ	ทำได้เป็นบ	ทำได้บ่อยๆ	ทำได้เป็นบ	ทำได้เป็นบ	ทำได้เป็นบ	ทำได้บ่อยๆ	ทำได้เป็นบ
ทำได้บ้าง/	ทำได้เป็นบ	ทำได้บ่อยๆ	ทำได้บ่อยๆ	ทำได้เป็นบ	ทำได้บ้าง/	ทำได้เป็นบ	ทำได้บ่อยๆ	ทำได้เป็นบ
ทำได้เป็นบ	ทำได้บ้าง/	ทำได้บ้าง/	ทำได้บ้าง/	ทำได้บ้าง/	ทำได้เป็นบ	ทำได้บ้าง/	ทำได้เป็นบ	ทำได้เป็นบ
ทำได้เป็นบ	ทำเป็นประ	ทำเป็นประ	ทำเป็นประ	ทำได้บ่อยๆ	ทำได้เป็นบ	ทำได้เป็นบ	ทำได้บ่อยๆ	ทำได้บ่อยๆ
ทำได้บ้าง/	ทำได้บ้าง/	ทำได้บ่อยๆ	ทำได้บ่อยๆ	ทำได้บ่อยๆ	ทำได้บ่อยๆ	ทำเป็นประ	ทำเป็นประ	ทำเป็นประ
ทำได้เป็นบ	ทำเป็นประ	ทำเป็นประ	ทำได้บ่อยๆ	ทำได้บ่อยๆ	ทำได้บ่อยๆ	ทำเป็นประ	ทำเป็นประ	ทำได้บ่อยๆ
ทำได้บ่อยๆ	ทำได้บ่อยๆ	ทำได้บ่อยๆ	ทำได้เป็นบ	ทำได้เป็นบ	ทำได้เป็นบ	ทำได้เป็นบ	ทำได้บ่อยๆ	ทำได้บ่อยๆ
ทำได้บ้าง/	ทำได้บ้าง/	ทำได้เป็นบ	ทำได้เป็นบ	ทำได้เป็นบ	ทำได้บ้าง/	ทำได้เป็นบ	ทำได้เป็นบ	ทำได้บ้าง/
ทำได้บ้าง/	ทำได้เป็นบ	ทำเป็นประ	ทำเป็นประ	ทำเป็นประ	ทำได้เป็นบ	ทำเป็นประ	ทำเป็นประ	ทำเป็นประ
ทำได้เป็นบ	ทำได้บ้าง/	ทำได้บ้าง/	ทำได้เป็นบ	ทำได้บ้าง/	ทำได้บ้าง/	ทำได้บ่อยๆ	ทำได้บ้าง/	ทำได้บ้าง/
ทำได้บ่อยๆ	ทำได้บ่อยๆ	ทำได้บ่อยๆ	ทำได้บ่อยๆ	ทำได้บ่อยๆ	ทำได้บ่อยๆ	ทำได้บ่อยๆ	ทำเป็นประ	ทำเป็นประ
ทำเป็นประ	ทำได้เป็นบ	ทำได้เป็นบ	ทำได้เป็นบ	ทำได้บ่อยๆ	ทำได้บ่อยๆ	ทำได้บ่อยๆ	ทำได้บ่อยๆ	ทำได้บ่อยๆ
ทำเป็นประ	ทำได้บ่อยๆ	ทำได้บ้าง/	ทำได้บ่อยๆ	ทำได้บ่อยๆ	ทำได้เป็นบ	ทำได้บ่อยๆ	ทำได้บ่อยๆ	ทำได้บ่อยๆ
ทำเป็นประ	ทำได้บ่อยๆ	ทำได้เป็นบ	ทำได้บ่อยๆ	ทำได้บ่อยๆ	ทำได้บ้าง/	ทำได้บ่อยๆ	ทำเป็นประ	ทำเป็นประ
ทำเป็นประ	ทำเป็นประ	ทำเป็นประ	ทำเป็นประ	ทำเป็นประ	ทำได้บ่อยๆ	ทำได้บ่อยๆ	ทำเป็นประ	ทำเป็นประ
ทำเป็นประ	ทำได้บ่อยๆ	ทำได้บ่อยๆ	ทำเป็นประ	ทำเป็นประ	ทำได้บ่อยๆ	ทำได้บ่อยๆ	ทำเป็นประ	ทำเป็นประ
ทำได้บ่อยๆ	ทำได้บ่อยๆ	ทำได้บ่อยๆ	ทำได้บ่อยๆ	ทำได้บ่อยๆ	ทำได้เป็นบ	ทำได้บ่อยๆ	ทำได้เป็นบ	ทำได้เป็นบ
ทำเป็นประ	ทำเป็นประ	ทำเป็นประ	ทำได้บ่อยๆ	ทำได้บ่อยๆ	ทำเป็นประ	ทำเป็นประ	ทำเป็นประ	ทำเป็นประ
ทำได้เป็นบ	ทำได้เป็นบ	ทำได้บ่อยๆ	ทำได้บ่อยๆ	ทำได้บ่อยๆ	ทำได้เป็นบ	ทำได้เป็นบ	ทำเป็นประ	ทำเป็นประ
ทำได้บ่อยๆ	ทำได้บ่อยๆ	ทำได้บ่อยๆ	ทำได้บ่อยๆ	ทำได้บ่อยๆ	ทำได้บ่อยๆ	ทำได้บ่อยๆ	ทำได้บ่อยๆ	ทำได้บ่อยๆ
ทำเป็นประ	ทำเป็นประ	ทำเป็นประ	ทำเป็นประ	ทำเป็นประ	ทำเป็นประ	ทำเป็นประ	ทำเป็นประ	ทำเป็นประ
ทำเป็นประ	ทำเป็นประ	ทำเป็นประ	ทำเป็นประ	ทำเป็นประ	ทำได้บ่อยๆ	ทำได้บ่อยๆ	ทำเป็นประ	ทำเป็นประ
ทำได้บ่อยๆ	ทำได้บ่อยๆ	ทำได้เป็นบ	ทำได้บ่อยๆ	ทำได้บ่อยๆ	ทำได้เป็นบ	ไม่เคยทำเ	ทำเป็นประ	ทำเป็นประ
ทำได้บ้าง/	ทำได้บ่อยๆ	ทำได้บ้าง/	ทำได้เป็นบ	ทำได้บ่อยๆ	ทำได้เป็นบ	ทำได้บ่อยๆ	ทำได้เป็นบ	ทำได้เป็นบ
ทำได้บ่อยๆ	ทำเป็นประ	ทำเป็นประ	ทำเป็นประ	ทำเป็นประ	ทำได้บ่อยๆ	ทำเป็นประ	ทำเป็นประ	ทำเป็นประ
ไม่เคยทำเ	ไม่เคยทำเ	ไม่เคยทำเ	ไม่เคยทำเ	ทำได้บ้าง/	ไม่เคยทำเ	ทำได้บ้าง/	ทำได้บ้าง/	ทำได้เป็นบ
ทำได้เป็นบ	ทำได้เป็นบ	ทำได้บ่อยๆ	ทำได้บ่อยๆ	ทำได้บ่อยๆ	ทำได้เป็นบ	ทำได้เป็นบ	ทำได้บ่อยๆ	ทำได้เป็นบ
ทำได้เป็นบ	ทำได้บ้าง/	ทำได้บ่อยๆ	ทำได้บ่อยๆ	ทำได้บ่อยๆ	ทำได้เป็นบ	ทำได้บ่อยๆ	ทำได้บ่อยๆ	ทำได้บ่อยๆ
ไม่เคยทำเ	ไม่เคยทำเ	ไม่เคยทำเ	ทำได้บ้าง/	ทำได้บ้าง/	ไม่เคยทำเ	ไม่เคยทำเ	ทำได้บ้าง/	ไม่เคยทำเ
ทำได้บ่อยๆ	ทำได้บ่อยๆ	ทำได้บ้าง/	ทำได้บ้าง/	ทำได้บ้าง/	ทำได้บ้าง/	ทำได้บ้าง/	ทำได้บ้าง/	ทำได้บ้าง/
ทำเป็นประ	ทำเป็นประ	ทำเป็นประ	ทำเป็นประ	ทำเป็นประ	ทำได้บ่อยๆ	ทำได้บ่อยๆ	ทำเป็นประ	ทำเป็นประ
ทำเป็นประ	ทำเป็นประ	ทำได้บ้าง/	ทำได้เป็นบ	ทำได้บ้าง/	ทำได้บ้าง/	ทำได้บ้าง/	ทำเป็นประ	ทำได้เป็นบ
ทำเป็นประ	ทำได้บ่อยๆ	ทำเป็นประ	ทำเป็นประ	ทำเป็นประ	ทำได้บ่อยๆ	ทำได้เป็นบ	ทำได้บ่อยๆ	ทำได้บ่อยๆ
ทำเป็นประ	ทำได้บ่อยๆ	ทำได้บ่อยๆ	ทำเป็นประ	ทำได้บ่อยๆ	ทำเป็นประ	ทำเป็นประ	ทำได้บ่อยๆ	ทำได้บ่อยๆ
ทำได้เป็นบ	ทำได้เป็นบ	ทำได้เป็นบ	ทำได้เป็นบ	ทำได้เป็นบ	ทำได้เป็นบ	ทำได้เป็นบ	ทำได้เป็นบ	ทำได้เป็นบ
ทำได้บ่อยๆ	ทำได้เป็นบ	ทำได้บ่อยๆ	ทำได้เป็นบ	ทำได้บ่อยๆ	ทำได้เป็นบ	ทำได้บ่อยๆ	ทำได้บ่อยๆ	ทำได้เป็นบ

ทำได้บ่อยๆ	ทำได้บ่อยๆ	ทำได้บ่อยๆ	ทำได้บ่อยๆ	ทำได้บ่อยๆ	ทำได้บ่อยๆ	ทำได้บ่อยๆ	ทำได้บ่อยๆ	ทำได้บ่อยๆ
ทำได้บ้าง/	ไม่เคยทำเล	ทำเป็นประ	ทำได้เป็นบ	ไม่เคยทำเล	ไม่เคยทำเล	ทำได้บ่อยๆ	ทำได้เป็นบ	ทำได้บ่อยๆ
ทำได้บ่อยๆ	ทำได้บ่อยๆ	ทำได้บ่อยๆ	ทำเป็นประ	ทำเป็นประ	ทำเป็นประ	ทำได้บ่อยๆ	ทำเป็นประ	ทำเป็นประ
ทำเป็นประ	ทำได้เป็นบ	ทำเป็นประ	ทำได้บ่อยๆ	ทำเป็นประ	ทำได้เป็นบ	ทำได้เป็นบ	ทำได้เป็นบ	ทำได้เป็นบ
ทำได้บ่อยๆ	ทำได้บ่อยๆ	ทำได้บ่อยๆ	ทำได้บ่อยๆ	ทำได้บ่อยๆ	ทำได้บ่อยๆ	ทำได้บ่อยๆ	ทำได้บ่อยๆ	ทำได้บ่อยๆ
ทำได้บ่อยๆ	ทำได้เป็นบ	ทำเป็นประ	ทำได้เป็นบ	ทำได้เป็นบ	ทำเป็นประ	ทำได้เป็นบ	ทำเป็นประ	ทำเป็นประ
ทำได้บ่อยๆ	ทำได้เป็นบ	ทำได้บ่อยๆ	ทำได้บ่อยๆ	ทำได้บ่อยๆ	ทำได้บ่อยๆ	ทำได้บ่อยๆ	ทำได้บ่อยๆ	ทำได้บ่อยๆ
ทำได้บ้าง/	ไม่เคยทำเล	ทำได้บ้าง/	ทำได้บ่อยๆ	ทำได้บ้าง/	ไม่เคยทำเล	ทำได้บ่อยๆ	ทำได้บ่อยๆ	ไม่เคยทำเล
ทำได้เป็นบ	ทำได้เป็นบ	ทำได้เป็นบ	ทำได้บ่อยๆ	ทำได้บ่อยๆ	ทำได้บ่อยๆ	ทำได้บ่อยๆ	ทำได้บ่อยๆ	ทำได้บ่อยๆ
ทำเป็นประ	ทำเป็นประ	ทำได้บ่อยๆ	ทำเป็นประ	ทำเป็นประ	ทำได้เป็นบ	ทำได้บ่อยๆ	ทำเป็นประ	ทำเป็นประ
ทำเป็นประ	ทำเป็นประ	ทำได้เป็นบ	ทำเป็นประ	ทำเป็นประ	ทำได้บ่อยๆ	ทำเป็นประ	ทำได้เป็นบ	ทำได้บ่อยๆ
ทำได้เป็นบ	ทำได้เป็นบ	ทำได้บ่อยๆ	ทำได้บ่อยๆ	ทำได้บ่อยๆ	ไม่เคยทำเล	ทำได้เป็นบ	ทำได้บ่อยๆ	ทำเป็นประ
ทำเป็นประ	ทำเป็นประ	ทำเป็นประ	ทำเป็นประ	ทำเป็นประ	ทำเป็นประ	ทำเป็นประ	ทำเป็นประ	ทำเป็นประ
ทำเป็นประ	ทำเป็นประ	ทำเป็นประ	ทำเป็นประ	ไม่เคยทำเล	ทำได้บ่อยๆ	ทำเป็นประ	ทำเป็นประ	ทำเป็นประ
ทำได้เป็นบ	ทำเป็นประ	ทำเป็นประ	ทำเป็นประ	ทำเป็นประ	ทำเป็นประ	ทำได้บ่อยๆ	ทำเป็นประ	ทำเป็นประ
ทำได้บ่อยๆ	ทำได้บ่อยๆ	ทำได้บ่อยๆ	ทำได้บ่อยๆ	ทำได้บ่อยๆ	ทำได้บ่อยๆ	ทำได้บ่อยๆ	ทำเป็นประ	ทำได้บ่อยๆ
ทำได้บ่อยๆ	ทำได้บ่อยๆ	ทำได้บ่อยๆ	ทำได้บ่อยๆ	ทำได้บ่อยๆ	ทำได้บ่อยๆ	ทำได้บ่อยๆ	ทำได้บ่อยๆ	ทำได้บ่อยๆ
ทำได้เป็นบ	ทำได้เป็นบ	ทำได้เป็นบ	ทำได้เป็นบ	ทำได้เป็นบ	ทำได้เป็นบ	ทำได้เป็นบ	ทำได้เป็นบ	ทำได้เป็นบ
ทำได้เป็นบ	ทำได้เป็นบ	ทำได้เป็นบ	ทำได้เป็นบ	ทำได้เป็นบ	ทำได้เป็นบ	ทำได้เป็นบ	ทำได้เป็นบ	ทำได้เป็นบ
ทำได้เป็นบ	ทำได้เป็นบ	ทำได้บ่อยๆ	ทำได้บ่อยๆ	ทำได้เป็นบ	ทำได้เป็นบ	ทำได้เป็นบ	ทำได้บ่อยๆ	ทำได้บ่อยๆ
ทำได้บ่อยๆ	ทำได้บ่อยๆ	ทำได้บ่อยๆ	ทำได้บ่อยๆ	ทำได้บ่อยๆ	ทำได้บ่อยๆ	ทำได้บ่อยๆ	ทำได้บ่อยๆ	ทำได้บ่อยๆ
ทำได้เป็นบ	ทำได้บ่อยๆ	ทำได้บ่อยๆ	ทำได้บ้าง/	ทำได้บ้าง/	ทำได้เป็นบ	ทำได้เป็นบ	ทำได้บ่อยๆ	ทำได้บ่อยๆ
ทำเป็นประ	ทำเป็นประ	ทำเป็นประ	ทำได้บ่อยๆ	ทำเป็นประ	ทำเป็นประ	ทำเป็นประ	ทำเป็นประ	ทำเป็นประ
ทำได้เป็นบ	ทำได้บ้าง/	ทำได้บ่อยๆ	ทำได้บ่อยๆ	ทำได้บ่อยๆ	ทำได้บ่อยๆ	ทำได้บ่อยๆ	ทำได้บ่อยๆ	ทำได้บ่อยๆ
ทำเป็นประ	ทำเป็นประ	ทำเป็นประ	ทำได้บ่อยๆ	ทำได้บ่อยๆ	ทำเป็นประ	ทำเป็นประ	ทำได้บ่อยๆ	ทำได้บ่อยๆ
ทำได้บ่อยๆ	ทำได้บ้าง/	ไม่เคยทำเล	ทำได้บ้าง/	ทำได้บ้าง/	ทำได้เป็นบ	ทำได้บ้าง/	ทำได้เป็นบ	ทำได้เป็นบ
ทำเป็นประ	ทำเป็นประ	ทำเป็นประ	ทำเป็นประ	ทำเป็นประ	ทำเป็นประ	ทำเป็นประ	ทำเป็นประ	ทำเป็นประ
ทำได้บ่อยๆ	ทำได้บ่อยๆ	ทำได้บ่อยๆ	ทำได้บ่อยๆ	ทำได้บ่อยๆ	ทำได้บ่อยๆ	ทำได้บ่อยๆ	ทำได้บ่อยๆ	ทำได้บ่อยๆ
ทำเป็นประ	ทำเป็นประ	ทำเป็นประ	ทำเป็นประ	ทำเป็นประ	ทำเป็นประ	ทำเป็นประ	ทำเป็นประ	ทำเป็นประ
ทำเป็นประ	ทำเป็นประ	ทำเป็นประ	ทำเป็นประ	ทำเป็นประ	ทำเป็นประ	ทำเป็นประ	ทำเป็นประ	ทำเป็นประ
ทำได้บ่อยๆ	ทำได้บ่อยๆ	ทำได้บ่อยๆ	ทำได้บ่อยๆ	ทำได้บ่อยๆ	ทำได้บ่อยๆ	ทำได้บ่อยๆ	ทำเป็นประ	ทำเป็นประ
ทำเป็นประ	ทำเป็นประ	ทำได้บ่อยๆ	ทำเป็นประ	ทำเป็นประ	ทำเป็นประ	ทำเป็นประ	ทำเป็นประ	ทำเป็นประ
ทำเป็นประ	ทำเป็นประ	ทำเป็นประ	ทำเป็นประ	ทำเป็นประ	ทำเป็นประ	ทำเป็นประ	ทำเป็นประ	ทำเป็นประ
ทำเป็นประ	ทำเป็นประ	ทำเป็นประ	ทำเป็นประ	ทำเป็นประ	ทำเป็นประ	ทำเป็นประ	ทำเป็นประ	ทำเป็นประ
ทำเป็นประ	ทำเป็นประ	ทำได้บ่อยๆ	ทำเป็นประ	ทำเป็นประ	ทำเป็นประ	ทำเป็นประ	ทำได้บ่อยๆ	ทำได้บ่อยๆ
ทำได้บ่อยๆ	ทำได้เป็นบ	ทำได้เป็นบ	ทำได้เป็นบ	ทำได้เป็นบ	ทำเป็นประ	ทำได้บ่อยๆ	ทำเป็นประ	ทำเป็นประ
ทำเป็นประ	ทำเป็นประ	ทำเป็นประ	ทำเป็นประ	ทำเป็นประ	ทำเป็นประ	ทำเป็นประ	ทำเป็นประ	ทำเป็นประ
ทำได้เป็นบ	ทำได้บ้าง/	ทำได้บ้าง/	ทำได้บ้าง/	ทำได้บ้าง/	ทำได้บ้าง/	ทำได้เป็นบ	ทำได้บ้าง/	ทำได้บ้าง/
ทำเป็นประ	ทำได้บ้าง/	ทำได้เป็นบ	ทำได้บ่อยๆ	ทำได้บ่อยๆ	ทำเป็นประ	ทำเป็นประ	ทำได้บ่อยๆ	ทำได้บ่อยๆ
ทำได้บ้าง/	ทำได้เป็นบ	ทำได้เป็นบ	ทำได้บ่อยๆ	ทำได้เป็นบ	ทำได้เป็นบ	ทำได้บ่อยๆ	ทำได้เป็นบ	ทำได้บ่อยๆ
ทำเป็นประ	ทำได้บ่อยๆ	ทำได้บ่อยๆ	ทำเป็นประ	ทำได้บ่อยๆ	ทำได้บ่อยๆ	ทำเป็นประ	ทำได้บ่อยๆ	ทำได้บ่อยๆ
ทำได้บ่อยๆ	ทำได้บ้าง/	ทำได้บ่อยๆ	ทำได้บ่อยๆ	ทำได้บ่อยๆ	ทำได้เป็นบ	ทำได้เป็นบ	ทำได้บ่อยๆ	ทำได้บ่อยๆ
ทำเป็นประ	ทำเป็นประ	ทำเป็นประ	ทำเป็นประ	ทำเป็นประ	ทำเป็นประ	ทำเป็นประ	ทำเป็นประ	ทำเป็นประ
ทำได้บ่อยๆ	ทำได้บ่อยๆ	ทำได้เป็นบ	ทำได้เป็นบ	ทำได้บ่อยๆ	ทำได้บ่อยๆ	ทำได้บ่อยๆ	ทำได้เป็นบ	ทำได้บ่อยๆ
ทำได้เป็นบ	ทำได้เป็นบ	ทำได้เป็นบ	ทำได้บ้าง/	ทำได้เป็นบ	ทำได้บ้าง/	ทำได้เป็นบ	ทำได้เป็นบ	ทำได้เป็นบ
ทำได้บ่อยๆ	ทำได้บ่อยๆ	ทำได้เป็นบ	ทำได้บ่อยๆ	ทำได้เป็นบ	ทำได้บ่อยๆ	ทำได้เป็นบ	ทำได้บ่อยๆ	ทำได้บ่อยๆ
ทำเป็นประ	ทำเป็นประ	ทำเป็นประ	ทำเป็นประ	ทำเป็นประ	ทำเป็นประ	ทำเป็นประ	ทำเป็นประ	ทำเป็นประ
ทำได้เป็นบ	ทำได้บ้าง/	ทำได้เป็นบ	ทำได้บ้าง/	ทำได้เป็นบ	ทำได้เป็นบ	ทำได้เป็นบ	ทำได้เป็นบ	ทำได้เป็นบ
ทำได้บ่อยๆ	ทำได้บ้าง/	ทำได้บ้าง/	ทำได้เป็นบ	ทำได้บ่อยๆ	ทำได้บ่อยๆ	ทำได้เป็นบ	ทำได้บ้าง/	ทำได้บ่อยๆ
ทำเป็นประ	ทำได้เป็นบ	ทำได้เป็นบ	ทำได้เป็นบ	ทำได้เป็นบ	ทำได้บ่อยๆ	ทำได้บ่อยๆ	ทำเป็นประ	ทำเป็นประ
ทำได้บ้าง/	ไม่เคยทำเล	ไม่เคยทำเล	ทำได้บ้าง/	ทำได้บ้าง/	ทำได้เป็นบ	ทำได้เป็นบ	ทำได้เป็นบ	ทำได้เป็นบ

ทำได้บ่อยๆ	ทำได้บ่อยๆ	ทำได้เป็นบ	ทำได้บ่อยๆ	ทำได้เป็นบ	ทำได้เป็นบ	ทำได้บ่อยๆ	ทำได้บ่อยๆ	ทำได้บ่อยๆ
ทำได้บ่อยๆ	ทำได้บ่อยๆ	ทำได้บ่อยๆ	ทำได้บ่อยๆ	ทำได้บ่อยๆ	ทำได้บ่อยๆ	ทำได้บ่อยๆ	ทำได้บ่อยๆ	ทำได้บ่อยๆ
ทำเป็นประ	ทำได้บ้าง/	ทำได้บ้าง/	ทำเป็นประ	ทำได้บ่อยๆ	ทำได้บ้าง/	ทำได้บ้าง/	ทำเป็นประ	ทำได้บ้าง/
ทำได้บ่อยๆ	ทำได้บ่อยๆ	ทำได้บ่อยๆ	ทำได้บ่อยๆ	ทำได้บ่อยๆ	ทำได้บ่อยๆ	ทำได้บ่อยๆ	ทำได้บ่อยๆ	ทำได้บ่อยๆ
ทำเป็นประ	ทำได้บ่อยๆ	ทำได้เป็นบ	ทำได้บ่อยๆ	ทำเป็นประ	ทำได้บ่อยๆ	ทำได้บ่อยๆ	ทำได้บ่อยๆ	ทำได้บ่อยๆ
ทำได้เป็นบ	ทำได้บ่อยๆ	ทำได้เป็นบ	ทำได้บ่อยๆ	ทำได้บ่อยๆ	ทำได้เป็นบ	ทำได้เป็นบ	ทำได้บ่อยๆ	ทำได้บ่อยๆ
ทำเป็นประ	ทำได้เป็นบ	ทำได้บ้าง/	ทำได้บ่อยๆ	ทำได้เป็นบ	ทำได้บ่อยๆ	ทำได้เป็นบ	ทำได้บ่อยๆ	ทำได้บ่อยๆ
ทำเป็นประ	ทำได้บ่อยๆ	ทำเป็นประ	ทำเป็นประ	ทำได้บ่อยๆ	ทำได้เป็นบ	ทำได้เป็นบ	ทำได้บ่อยๆ	ทำเป็นประ
ทำเป็นประ	ทำเป็นประ	ทำเป็นประ	ทำเป็นประ	ทำเป็นประ	ทำได้บ่อยๆ	ทำเป็นประ	ทำได้บ่อยๆ	ทำเป็นประ
ทำได้บ่อยๆ	ทำได้บ้าง/	ทำเป็นประ	ทำเป็นประ	ทำเป็นประ	ทำเป็นประ	ทำเป็นประ	ทำได้บ่อยๆ	ทำได้บ่อยๆ
ทำได้บ้าง/	ทำได้บ่อยๆ	ทำได้บ้าง/	ทำได้บ่อยๆ	ทำได้บ่อยๆ	ทำได้บ้าง/	ทำได้บ่อยๆ	ทำได้บ่อยๆ	ทำได้บ่อยๆ
ทำได้บ่อยๆ	ทำได้บ้าง/	ทำเป็นประ	ทำเป็นประ	ทำเป็นประ	ทำเป็นประ	ทำเป็นประ	ทำได้บ่อยๆ	ทำได้บ่อยๆ
ทำได้บ่อยๆ	ทำได้บ่อยๆ	ทำได้บ่อยๆ	ทำได้บ่อยๆ	ทำได้บ้าง/	ทำได้เป็นบ	ทำได้บ่อยๆ	ทำได้บ่อยๆ	ทำได้บ่อยๆ
ทำได้บ่อยๆ	ทำได้บ่อยๆ	ทำได้เป็นบ	ทำได้เป็นบ	ทำได้บ่อยๆ	ทำได้บ่อยๆ	ทำได้บ่อยๆ	ทำได้บ่อยๆ	ทำได้บ่อยๆ
ทำเป็นประ	ทำเป็นประ	ทำเป็นประ	ทำเป็นประ	ทำได้เป็นบ	ทำได้เป็นบ	ทำเป็นประ	ทำเป็นประ	ทำได้บ่อยๆ
ทำได้บ่อยๆ	ไม่เคยทำเ	ทำได้บ่อยๆ	ทำได้เป็นบ	ทำได้เป็นบ	ทำได้บ่อยๆ	ทำได้บ่อยๆ	ทำได้บ่อยๆ	ทำได้เป็นบ
ทำได้เป็นบ	ทำเป็นประ	ทำได้เป็นบ	ทำได้เป็นบ	ทำได้เป็นบ	ทำเป็นประ	ทำเป็นประ	ทำได้เป็นบ	ทำเป็นประ
ทำได้เป็นบ	ทำได้เป็นบ	ทำได้เป็นบ	ทำได้เป็นบ	ทำได้เป็นบ	ทำได้บ้าง/	ทำได้เป็นบ	ทำได้เป็นบ	ทำได้เป็นบ
ทำได้บ้าง/	ไม่เคยทำเ	ไม่เคยทำเ	ทำได้เป็นบ	ทำได้เป็นบ	ทำได้บ้าง/	ทำได้บ้าง/	ทำได้บ้าง/	ทำได้บ้าง/
ทำเป็นประ	ทำเป็นประ	ทำเป็นประ	ทำเป็นประ	ทำเป็นประ	ทำเป็นประ	ทำเป็นประ	ทำเป็นประ	ทำเป็นประ
ทำเป็นประ	ทำเป็นประ	ทำเป็นประ	ทำเป็นประ	ทำเป็นประ	ทำเป็นประ	ทำเป็นประ	ทำเป็นประ	ทำเป็นประ
ทำได้บ้าง/	ทำเป็นประ	ทำเป็นประ	ทำเป็นประ	ทำเป็นประ	ทำได้เป็นบ	ทำเป็นประ	ทำเป็นประ	ทำเป็นประ
ทำได้บ่อยๆ	ทำได้บ่อยๆ	ทำได้บ่อยๆ	ทำได้บ่อยๆ	ทำได้บ่อยๆ	ทำได้เป็นบ	ทำได้บ่อยๆ	ทำเป็นประ	ทำเป็นประ
ทำได้บ่อยๆ	ทำได้เป็นบ	ทำได้เป็นบ	ไม่เคยทำเ	ไม่เคยทำเ	ทำได้เป็นบ	ไม่เคยทำเ	ทำได้บ่อยๆ	ทำได้บ่อยๆ
ทำได้บ่อยๆ	ทำได้บ่อยๆ	ทำได้บ่อยๆ	ทำได้เป็นบ	ทำได้เป็นบ	ทำได้เป็นบ	ทำได้เป็นบ	ทำได้บ่อยๆ	ทำได้บ่อยๆ
ทำได้บ่อยๆ	ทำได้เป็นบ	ทำได้บ่อยๆ	ทำเป็นประ	ทำเป็นประ	ทำได้บ่อยๆ	ทำได้บ่อยๆ	ทำได้บ่อยๆ	ทำได้บ่อยๆ
ทำเป็นประ	ทำได้บ่อยๆ	ทำเป็นประ	ทำได้บ้าง/	ทำได้เป็นบ	ทำได้เป็นบ	ทำได้บ้าง/	ทำได้บ่อยๆ	ทำเป็นประ
ทำได้เป็นบ	ไม่เคยทำเ	ทำได้เป็นบ	ทำได้บ่อยๆ	ทำได้บ่อยๆ	ทำได้เป็นบ	ทำได้เป็นบ	ทำได้เป็นบ	ทำได้เป็นบ
ทำได้บ่อยๆ	ทำได้บ่อยๆ	ทำได้บ่อยๆ	ทำได้บ่อยๆ	ทำได้บ่อยๆ	ทำได้บ่อยๆ	ทำได้บ่อยๆ	ทำได้เป็นบ	ทำได้เป็นบ
ทำเป็นประ	ทำได้บ่อยๆ	ทำได้บ่อยๆ	ทำได้บ่อยๆ	ทำได้บ่อยๆ	ทำได้บ่อยๆ	ทำได้บ่อยๆ	ทำได้บ่อยๆ	ทำได้บ่อยๆ
ทำได้บ่อยๆ	ทำได้บ่อยๆ	ทำได้บ่อยๆ	ทำได้บ่อยๆ	ทำได้บ่อยๆ	ทำได้บ่อยๆ	ทำได้บ่อยๆ	ทำได้บ่อยๆ	ทำได้บ่อยๆ
ทำได้บ่อยๆ	ทำเป็นประ	ทำเป็นประ	ทำเป็นประ	ทำเป็นประ	ทำได้บ่อยๆ	ทำได้บ่อยๆ	ทำเป็นประ	ทำเป็นประ
ทำเป็นประ	ทำเป็นประ	ทำได้บ่อยๆ	ทำเป็นประ	ทำได้บ่อยๆ	ทำได้บ่อยๆ	ทำเป็นประ	ทำเป็นประ	ทำเป็นประ
ทำเป็นประ	ทำได้บ่อยๆ	ทำได้บ่อยๆ	ทำได้บ่อยๆ	ทำได้เป็นบ	ทำได้เป็นบ	ทำได้เป็นบ	ทำเป็นประ	ทำได้บ่อยๆ
ทำได้เป็นบ	ทำเป็นประ	ทำเป็นประ	ทำได้บ่อยๆ	ทำเป็นประ	ทำได้บ่อยๆ	ทำได้เป็นบ	ทำได้บ่อยๆ	ทำได้บ่อยๆ
ทำได้บ่อยๆ	ทำได้เป็นบ	ทำได้เป็นบ	ทำเป็นประ	ทำเป็นประ	ทำได้บ่อยๆ	ทำได้บ่อยๆ	ทำเป็นประ	ทำเป็นประ
ทำเป็นประ	ทำได้บ่อยๆ	ทำได้บ่อยๆ	ทำได้บ่อยๆ	ทำได้บ่อยๆ	ทำได้บ่อยๆ	ทำได้บ่อยๆ	ทำได้บ่อยๆ	ทำได้เป็นบ
ทำได้บ่อยๆ	ทำได้บ้าง/	ทำได้เป็นบ	ไม่เคยทำเ	ทำได้บ่อยๆ	ทำเป็นประ	ทำได้เป็นบ	ทำเป็นประ	ทำได้บ่อยๆ
ทำได้เป็นบ	ทำได้เป็นบ	ทำได้บ่อยๆ	ทำได้บ่อยๆ	ทำได้บ่อยๆ	ทำได้เป็นบ	ทำได้บ้าง/	ทำได้บ่อยๆ	ทำได้บ่อยๆ
ทำได้บ่อยๆ	ทำได้บ่อยๆ	ทำได้บ้าง/	ทำได้บ้าง/	ทำเป็นประ	ทำเป็นประ	ทำได้บ่อยๆ	ทำได้บ่อยๆ	ทำเป็นประ
ทำเป็นประ	ทำได้เป็นบ	ทำได้เป็นบ	ทำเป็นประ	ทำเป็นประ	ทำเป็นประ	ทำได้บ่อยๆ	ทำเป็นประ	ทำเป็นประ
ทำเป็นประ	ทำได้เป็นบ	ทำได้เป็นบ	ทำเป็นประ	ทำได้บ้าง/	ทำได้บ่อยๆ	ทำได้เป็นบ	ทำได้เป็นบ	ทำได้เป็นบ
ทำเป็นประ	ทำได้บ่อยๆ	ไม่เคยทำเ	ทำได้เป็นบ	ทำได้บ่อยๆ	ทำเป็นประ	ทำเป็นประ	ทำเป็นประ	ทำเป็นประ
ทำได้เป็นบ	ทำได้เป็นบ	ทำได้บ่อยๆ	ทำได้บ้าง/	ทำได้เป็นบ	ทำได้บ้าง/	ทำได้เป็นบ	ทำได้บ้าง/	ทำได้เป็นบ
ทำได้บ่อยๆ	ทำได้บ่อยๆ	ทำได้บ่อยๆ	ทำได้บ่อยๆ	ทำได้บ่อยๆ	ทำได้บ่อยๆ	ทำเป็นประ	ทำเป็นประ	ทำได้บ่อยๆ
ทำได้บ้าง/	ทำได้เป็นบ	ทำเป็นประ	ไม่เคยทำเ	ทำได้บ้าง/	ทำได้บ้าง/	ทำได้บ้าง/	ทำได้เป็นบ	ทำได้บ้าง/
ทำได้บ่อยๆ	ทำได้บ่อยๆ	ทำได้บ่อยๆ	ทำเป็นประ	ทำได้บ่อยๆ	ทำได้บ่อยๆ	ทำได้บ่อยๆ	ทำได้บ่อยๆ	ทำได้บ่อยๆ
ทำเป็นประ	ทำเป็นประ	ทำเป็นประ	ทำได้บ่อยๆ	ทำได้บ่อยๆ	ทำได้เป็นบ	ทำได้เป็นบ	ทำได้บ่อยๆ	ทำได้บ่อยๆ
ทำได้เป็นบ	ทำได้บ้าง/	ทำได้บ้าง/	ทำได้บ้าง/	ทำได้บ้าง/	ทำได้บ้าง/	ทำได้เป็นบ	ทำได้เป็นบ	ทำได้เป็นบ
ทำเป็นประ	ทำเป็นประ	ทำเป็นประ	ทำได้บ่อยๆ	ทำได้บ่อยๆ	ทำได้บ่อยๆ	ทำเป็นประ	ทำเป็นประ	ทำเป็นประ
ทำได้บ้าง/	ทำได้บ้าง/	ทำได้บ้าง/	ทำได้บ่อยๆ	ทำได้เป็นบ	ทำได้เป็นบ	ทำได้เป็นบ	ทำได้เป็นบ	ทำได้เป็นบ

ทำได้บ่อยๆ ทำได้เป็นบ ทำเป็นประ ทำได้บ่อยๆ ทำได้บ่อยๆ ทำได้บ่อยๆ ทำได้บ่อยๆ ทำได้บ่อยๆ ทำได้บ่อยๆ

ทำได้บ่อยๆ ทำได้บ่อยๆ ทำได้บ่อยๆ ทำเป็นประ ทำเป็นประ ทำได้บ่อยๆ ทำได้เป็นบ ทำได้บ่อยๆ ทำได้บ่อยๆ

ทำได้บ่อยๆ ทำเป็นประ ทำเป็นประ ทำได้บ่อยๆ ทำได้บ่อยๆ ทำได้บ่อยๆ ทำเป็นประ ทำเป็นประ ทำได้บ่อยๆ

ทำเป็นประ ทำเป็นประ ทำเป็นประ ทำเป็นประ ทำเป็นประ ทำเป็นประ ทำเป็นประ ทำเป็นประ ทำเป็นประ

ทำเป็นประ ทำเป็นประ ทำเป็นประ ทำเป็นประ ทำเป็นประ ทำเป็นประ ทำเป็นประ ทำเป็นประ ทำเป็นประ

ทำได้บ้าง/ ทำได้บ้าง/ ทำได้บ้าง/ ทำได้บ้าง/ ทำได้บ้าง/ ทำได้เป็นบ ทำได้บ้าง/ ทำได้บ้าง/ ทำได้เป็นบ

ไม่เคยทำเ ทำเป็นประ ทำเป็นประ ทำเป็นประ ทำเป็นประ ทำเป็นประ ทำเป็นประ ทำเป็นประ ทำเป็นประ

ทำเป็นประ ทำได้เป็นบ ทำได้บ่อยๆ ทำได้บ่อยๆ ทำได้เป็นบ ทำได้บ่อยๆ ทำได้บ่อยๆ ทำได้บ่อยๆ ทำได้บ่อยๆ

ทำได้เป็นบ ทำเป็นประ ทำเป็นประ ทำเป็นประ ทำเป็นประ ทำได้บ่อยๆ ทำได้บ่อยๆ ทำได้บ่อยๆ ทำได้บ่อยๆ

ทำได้บ่อยๆ ทำได้เป็นบ ทำได้บ่อยๆ ทำได้บ่อยๆ ทำได้บ่อยๆ ทำได้บ้าง/ ทำได้เป็นบ ทำเป็นประ ทำเป็นประ

ทำเป็นประ ทำเป็นประ ทำเป็นประ ทำเป็นประ ทำได้บ่อยๆ ทำได้บ่อยๆ ทำได้บ่อยๆ ทำได้บ่อยๆ ทำได้บ่อยๆ

ทำได้เป็นบ ไม่เคยทำเ ทำได้บ่อยๆ ทำได้บ้าง/ ทำได้บ้าง/ ทำได้เป็นบ ทำได้บ้าง/ ทำได้บ่อยๆ ทำได้เป็นบ

ทำได้บ่อยๆ ทำได้บ่อยๆ ทำได้เป็นบ ทำได้บ่อยๆ ทำได้บ่อยๆ ทำได้เป็นบ ทำได้บ่อยๆ ทำเป็นประ ทำเป็นประ

ทำได้บ่อยๆ ทำได้บ้าง/ ทำได้เป็นบ ทำได้บ่อยๆ ทำได้บ่อยๆ ทำได้บ่อยๆ ทำได้บ่อยๆ ทำได้บ่อยๆ ทำได้บ่อยๆ

ทำได้บ่อยๆ ทำได้เป็นบ ไม่เคยทำเ ไม่เคยทำเ ไม่เคยทำเ ไม่เคยทำเ ทำได้บ่อยๆ ทำได้บ่อยๆ ไม่เคยทำเ

ทำเป็นประ ทำเป็นประ ทำเป็นประ ทำได้บ่อยๆ ทำได้บ่อยๆ ทำเป็นประ ทำเป็นประ ทำได้บ่อยๆ ทำได้บ่อยๆ

ทำได้เป็นบ ทำได้บ้าง/ ทำเป็นประ ทำเป็นประ ทำได้บ่อยๆ ทำได้บ่อยๆ ทำได้บ่อยๆ ทำเป็นประ ทำเป็นประ

ทำได้บ่อยๆ ทำได้บ่อยๆ ทำเป็นประ ทำได้บ่อยๆ ทำได้เป็นบ ทำได้เป็นบ ทำได้เป็นบ ทำได้เป็นบ ทำได้เป็นบ

ทำได้เป็นบ ทำได้เป็นบ ทำได้บ่อยๆ ทำได้บ่อยๆ ทำได้เป็นบ ทำได้บ่อยๆ ทำได้บ่อยๆ ทำเป็นประ ทำเป็นประ

ทำได้เป็นบ ทำได้บ่อยๆ ทำเป็นประ ทำได้บ่อยๆ ทำเป็นประ ทำได้บ่อยๆ ทำได้บ่อยๆ ทำเป็นประ ทำได้บ่อยๆ

ทำเป็นประ ทำได้บ่อยๆ ทำเป็นประ ทำเป็นประ ทำเป็นประ ทำได้บ่อยๆ ทำได้บ่อยๆ ทำเป็นประ ทำเป็นประ

ทำเป็นประ ทำได้บ่อยๆ ทำได้บ่อยๆ ทำได้บ่อยๆ ทำได้บ่อยๆ ทำได้บ่อยๆ ทำเป็นประ ทำได้บ่อยๆ ทำได้บ่อยๆ

ทำได้เป็นบ ทำได้บ่อยๆ ทำได้บ่อยๆ ทำได้เป็นบ ทำได้เป็นบ ทำได้เป็นบ ทำได้บ้าง/ ทำได้เป็นบ ทำได้เป็นบ

ทำเป็นประ ทำเป็นประ ทำได้บ่อยๆ ทำได้บ่อยๆ ทำได้บ่อยๆ ทำได้บ่อยๆ ทำได้บ่อยๆ ทำเป็นประ ทำเป็นประ

ทำเป็นประ ทำเป็นประ ทำได้บ่อยๆ ทำได้บ่อยๆ ทำได้บ่อยๆ ทำได้บ่อยๆ ทำได้บ่อยๆ ทำเป็นประ ทำเป็นประ

ไม่เคยทำเ ทำได้บ้าง/ ทำได้เป็นบ ทำได้เป็นบ ทำได้เป็นบ ทำได้เป็นบ ทำได้เป็นบ ทำได้บ่อยๆ ทำได้เป็นบ

ทำเป็นประ ทำได้บ่อยๆ ทำเป็นประ ทำได้บ่อยๆ ทำเป็นประ ทำเป็นประ ทำได้บ่อยๆ ทำได้บ่อยๆ ทำได้บ่อยๆ

ทำได้เป็นบ ทำได้เป็นบ ทำได้บ้าง/ ทำได้เป็นบ ทำได้เป็นบ ทำได้เป็นบ ทำได้บ่อยๆ ทำได้บ่อยๆ ทำได้บ่อยๆ

ไม่เคยทำเ ไม่เคยทำเ ไม่เคยทำเ ไม่เคยทำเ ไม่เคยทำเ ไม่เคยทำเ ไม่เคยทำเ ไม่เคยทำเ ไม่เคยทำเ

ทำได้บ่อยๆ ทำได้บ่อยๆ ทำเป็นประ ทำเป็นประ ทำเป็นประ ทำได้บ่อยๆ ทำได้บ่อยๆ ทำเป็นประ ทำได้บ่อยๆ

ทำเป็นประ ทำได้เป็นบ ทำได้เป็นบ ทำได้บ่อยๆ ทำเป็นประ ทำได้บ่อยๆ ทำได้บ่อยๆ ทำได้บ่อยๆ ทำเป็นประ

ทำได้เป็นบ ทำได้เป็นบ ทำได้บ่อยๆ ทำได้เป็นบ ทำได้บ่อยๆ ทำได้บ่อยๆ ทำได้เป็นบ ทำได้บ่อยๆ ทำได้บ่อยๆ

ทำเป็นประ ทำได้บ่อยๆ ทำได้บ่อยๆ ทำได้บ่อยๆ ทำได้บ่อยๆ ทำได้บ่อยๆ ทำได้บ่อยๆ ทำได้บ่อยๆ ทำเป็นประ

ทำเป็นประ ทำเป็นประ ทำเป็นประ ไม่เคยทำเ ไม่เคยทำเ ไม่เคยทำเ ไม่เคยทำเ ไม่เคยทำเ ไม่เคยทำเ

ทำเป็นประ ทำเป็นประ ทำได้เป็นบ ทำเป็นประ ทำเป็นประ ทำได้บ่อยๆ ทำได้บ่อยๆ ทำเป็นประ ทำเป็นประ

ทำได้บ่อยๆ ทำเป็นประ ทำเป็นประ ทำเป็นประ ทำเป็นประ ทำเป็นประ ทำเป็นประ ทำเป็นประ ทำเป็นประ

ทำได้เป็นบ ทำได้เป็นบ ทำได้บ้าง/ ทำได้เป็นบ ทำได้เป็นบ ทำได้เป็นบ ทำได้บ่อยๆ ทำได้บ่อยๆ ทำได้บ่อยๆ

ทำเป็นประ ทำได้บ่อยๆ ทำเป็นประ ทำได้บ่อยๆ ทำได้บ่อยๆ ทำเป็นประ ทำเป็นประ ทำเป็นประ ทำได้บ่อยๆ

ทำเป็นประ ทำเป็นประ ทำเป็นประ ทำเป็นประ ทำเป็นประ ทำได้เป็นบ ทำเป็นประ ทำเป็นประ ทำเป็นประ

ทำได้เป็นบ ทำได้เป็นบ ทำได้เป็นบ ทำเป็นประ ทำได้บ่อยๆ ทำได้เป็นบ ทำได้เป็นบ ทำได้บ่อยๆ ทำได้เป็นบ

ทำได้เป็นบ ทำได้บ่อยๆ ทำได้บ่อยๆ ทำได้บ่อยๆ ทำได้บ่อยๆ ทำได้เป็นบ ทำได้บ่อยๆ ทำได้เป็นบ ทำได้บ่อยๆ

ทำได้เป็นบ ไม่เคยทำเ ไม่เคยทำเ ไม่เคยทำเ ทำได้บ้าง/ ทำได้เป็นบ ทำได้เป็นบ ทำได้เป็นบ ทำได้เป็นบ

ทำเป็นประ ทำเป็นประ ทำเป็นประ ทำได้บ้าง/ ทำเป็นประ ทำเป็นประ ทำเป็นประ ทำเป็นประ ทำเป็นประ

ทำได้เป็นบ ทำได้บ่อยๆ ทำได้บ่อยๆ ทำได้บ่อยๆ ทำเป็นประ ทำได้บ่อยๆ ทำได้บ่อยๆ ทำได้บ่อยๆ ทำได้เป็นบ

ทำได้บ่อยๆ ทำได้เป็นบ ทำได้เป็นบ ทำได้บ่อยๆ ไม่เคยทำเ ทำได้บ่อยๆ ทำได้บ่อยๆ ทำได้บ่อยๆ ทำได้บ่อยๆ

ทำเป็นประ ทำได้บ่อยๆ ทำได้บ่อยๆ ทำเป็นประ ทำเป็นประ ทำเป็นประ ทำเป็นประ ทำได้บ่อยๆ ทำได้บ่อยๆ

ทำได้บ่อยๆ ทำได้บ่อยๆ ทำได้บ่อยๆ ทำได้บ่อยๆ ทำได้เป็นบ ทำได้บ่อยๆ ทำได้เป็นบ ทำได้บ่อยๆ ทำได้บ่อยๆ

ทำได้บ่อยๆ ไม่เคยทำเ ทำได้เป็นบ ทำได้บ่อยๆ ทำได้บ่อยๆ ทำได้เป็นบ ทำได้เป็นบ ทำได้เป็นบ ทำได้บ่อยๆ

ทำได้บ่อยๆ ทำเป็นประ ทำเป็นประ ทำเป็นประ ทำเป็นประ ทำได้เป็นบ ทำเป็นประ ทำเป็นประ ทำเป็นประ

ทำได้บ่อยๆ ทำได้เป็นบ ทำได้บ่อยๆ ทำได้เป็นบ ทำได้บ่อยๆ ทำได้บ่อยๆ ทำได้บ่อยๆ ทำได้บ่อยๆ ทำได้บ่อยๆ

ทำเป็นประ ทำเป็นประ ทำได้บ่อยๆ ทำเป็นประ ทำเป็นประ ทำเป็นประ ทำเป็นประ ทำได้บ่อยๆ ทำได้บ่อยๆ

ทำได้บ่อยๆ	ทำได้เป็นบ	ทำได้บ่อยๆ	ทำได้บ่อยๆ	ทำได้บ่อยๆ	ทำได้บ่อยๆ	ทำได้บ่อยๆ	ทำได้บ่อยๆ	ทำได้บ่อยๆ
ทำเป็นประ	ทำเป็นประ	ทำเป็นประ	ทำได้บ่อยๆ	ทำได้บ่อยๆ	ทำได้บ่อยๆ	ทำได้บ่อยๆ	ทำได้บ่อยๆ	ทำเป็นประ
ทำเป็นประ	ทำเป็นประ	ทำเป็นประ	ทำเป็นประ	ทำเป็นประ	ทำได้บ่อยๆ	ทำเป็นประ	ทำเป็นประ	ทำเป็นประ
ทำเป็นประ	ทำได้บ่อยๆ	ไม่เคยทำเ	ทำได้บ่อยๆ	ทำได้บ่อยๆ	ทำได้บ่อยๆ	ทำได้บ่อยๆ	ทำเป็นประ	ทำเป็นประ
ทำได้เป็นบ	ทำได้เป็นบ	ทำได้เป็นบ	ทำได้เป็นบ	ทำได้เป็นบ	ทำได้บ้าง/	ทำได้บ้าง/	ทำได้เป็นบ	ทำได้เป็นบ
ทำได้บ่อยๆ	ทำเป็นประ	ทำเป็นประ	ทำเป็นประ	ทำได้บ่อยๆ	ทำได้บ้าง/	ทำได้เป็นบ	ทำได้เป็นบ	ทำได้บ่อยๆ
ทำได้บ่อยๆ	ทำได้บ่อยๆ	ทำได้บ่อยๆ	ทำได้บ่อยๆ	ทำได้บ่อยๆ	ทำได้บ่อยๆ	ทำได้บ่อยๆ	ทำได้บ่อยๆ	ทำได้บ่อยๆ
ทำได้บ่อยๆ	ทำได้บ่อยๆ	ทำได้บ่อยๆ	ทำได้บ่อยๆ	ทำได้บ่อยๆ	ทำได้เป็นบ	ทำได้บ่อยๆ	ทำได้เป็นบ	ทำได้บ่อยๆ
ทำได้เป็นบ	ทำได้เป็นบ	ทำได้เป็นบ	ทำได้เป็นบ	ทำได้เป็นบ	ทำได้เป็นบ	ทำได้เป็นบ	ทำได้เป็นบ	ทำได้เป็นบ
ทำได้บ่อยๆ	ทำได้เป็นบ	ทำได้เป็นบ	ทำได้บ่อยๆ	ทำได้บ่อยๆ	ทำได้บ่อยๆ	ทำได้บ่อยๆ	ทำได้บ่อยๆ	ทำได้บ่อยๆ
ทำเป็นประ	ทำเป็นประ	ทำเป็นประ	ทำได้บ่อยๆ	ทำได้บ่อยๆ	ทำได้บ่อยๆ	ทำได้บ่อยๆ	ทำได้บ่อยๆ	ทำได้บ่อยๆ
ทำได้บ่อยๆ	ทำได้บ่อยๆ	ทำได้บ่อยๆ	ทำได้บ่อยๆ	ทำได้เป็นบ	ทำได้บ่อยๆ	ทำได้บ่อยๆ	ทำได้เป็นบ	ทำได้บ่อยๆ
ทำได้บ่อยๆ	ทำได้บ่อยๆ	ทำได้บ่อยๆ	ทำได้บ่อยๆ	ทำได้เป็นบ	ทำได้เป็นบ	ทำได้เป็นบ	ทำได้เป็นบ	ทำได้เป็นบ
ทำเป็นประ	ทำเป็นประ	ทำได้บ่อยๆ	ทำได้เป็นบ	ทำได้เป็นบ	ทำได้บ่อยๆ	ทำได้บ้าง/	ทำได้เป็นบ	ทำได้บ่อยๆ
ทำเป็นประ	ทำได้เป็นบ	ทำได้บ่อยๆ	ทำได้บ่อยๆ	ทำได้เป็นบ	ทำได้เป็นบ	ทำได้บ่อยๆ	ทำได้เป็นบ	ทำได้บ่อยๆ
ทำได้เป็นบ	ทำได้บ่อยๆ	ทำได้บ่อยๆ	ทำได้บ่อยๆ	ทำได้เป็นบ	ทำได้บ่อยๆ	ทำได้เป็นบ	ทำได้บ่อยๆ	ทำเป็นประ
ทำได้บ่อยๆ	ทำได้เป็นบ	ทำได้เป็นบ	ทำได้เป็นบ	ทำได้บ่อยๆ	ทำได้บ่อยๆ	ทำได้บ่อยๆ	ทำได้บ่อยๆ	ทำได้บ่อยๆ
ทำได้เป็นบ	ทำได้บ่อยๆ	ทำได้บ่อยๆ	ทำได้เป็นบ	ทำได้บ่อยๆ	ทำได้เป็นบ	ทำได้บ่อยๆ	ทำได้บ่อยๆ	ทำได้เป็นบ
ทำได้บ่อยๆ	ทำได้เป็นบ	ทำได้บ่อยๆ	ทำได้บ่อยๆ	ทำได้เป็นบ	ทำได้บ่อยๆ	ทำได้บ่อยๆ	ทำได้เป็นบ	ทำได้บ่อยๆ
ทำได้บ่อยๆ	ทำเป็นประ	ทำเป็นประ	ทำได้บ่อยๆ	ทำได้เป็นบ	ทำได้บ้าง/	ทำได้บ่อยๆ	ทำเป็นประ	ทำเป็นประ
ทำเป็นประ	ทำเป็นประ	ทำเป็นประ	ทำได้บ่อยๆ	ทำได้บ่อยๆ	ทำได้บ้าง/	ทำได้บ่อยๆ	ทำได้บ่อยๆ	ทำได้บ่อยๆ
ทำได้บ่อยๆ	ทำได้บ่อยๆ	ทำได้บ่อยๆ	ทำเป็นประ	ทำได้บ่อยๆ	ทำได้บ้าง/	ทำได้บ้าง/	ทำได้เป็นบ	ทำได้เป็นบ
ทำได้เป็นบ	ทำได้บ้าง/	ทำได้บ้าง/	ทำเป็นประ	ทำเป็นประ	ทำได้บ่อยๆ	ทำได้บ่อยๆ	ทำได้บ่อยๆ	ทำได้เป็นบ
ทำเป็นประ	ทำได้บ้าง/	ทำได้เป็นบ	ทำเป็นประ	ทำเป็นประ	ทำได้บ่อยๆ	ทำได้เป็นบ	ทำเป็นประ	ทำเป็นประ
ทำได้บ่อยๆ	ทำได้เป็นบ	ทำได้เป็นบ	ทำได้เป็นบ	ทำได้เป็นบ	ทำได้บ่อยๆ	ทำได้เป็นบ	ทำได้บ่อยๆ	ทำได้บ่อยๆ
ทำได้เป็นบ	ทำได้เป็นบ	ทำได้เป็นบ	ทำได้เป็นบ	ทำได้บ้าง/	ทำได้เป็นบ	ทำได้เป็นบ	ทำได้เป็นบ	ทำได้เป็นบ
ทำได้บ่อยๆ	ทำได้บ่อยๆ	ทำได้เป็นบ	ทำได้บ่อยๆ	ทำได้บ่อยๆ	ทำได้บ่อยๆ	ทำได้บ่อยๆ	ทำได้บ่อยๆ	ทำได้บ่อยๆ
ทำได้เป็นบ	ทำได้บ่อยๆ	ทำได้บ่อยๆ	ทำเป็นประ	ทำเป็นประ	ทำได้เป็นบ	ทำได้เป็นบ	ทำได้บ่อยๆ	ทำได้บ่อยๆ
ทำได้เป็นบ	ทำได้บ่อยๆ	ทำเป็นประ	ทำได้บ่อยๆ	ทำได้บ่อยๆ	ทำได้เป็นบ	ทำได้เป็นบ	ทำได้บ่อยๆ	ทำได้บ่อยๆ
ทำได้บ่อยๆ	ทำได้บ่อยๆ	ทำได้บ่อยๆ	ทำได้บ่อยๆ	ทำได้บ่อยๆ	ทำได้เป็นบ	ทำได้บ่อยๆ	ทำได้บ่อยๆ	ทำได้บ่อยๆ
ทำได้เป็นบ	ทำได้เป็นบ	ทำได้บ่อยๆ	ทำได้บ่อยๆ	ทำได้เป็นบ	ทำได้เป็นบ	ทำได้บ่อยๆ	ทำได้บ่อยๆ	ทำได้บ่อยๆ
ทำเป็นประ	ทำเป็นประ	ทำเป็นประ	ทำเป็นประ	ทำเป็นประ	ทำได้บ่อยๆ	ทำเป็นประ	ทำเป็นประ	ทำเป็นประ
ทำได้บ่อยๆ	ทำได้เป็นบ	ทำเป็นประ	ทำเป็นประ	ทำเป็นประ	ทำได้บ่อยๆ	ทำได้บ่อยๆ	ทำได้เป็นบ	ทำเป็นประ
ทำเป็นประ	ทำได้บ้าง/	ทำเป็นประ	ทำได้บ่อยๆ	ทำได้บ่อยๆ	ทำได้บ่อยๆ	ทำได้บ่อยๆ	ทำเป็นประ	ทำได้บ่อยๆ
ทำเป็นประ	ทำเป็นประ	ทำเป็นประ	ทำเป็นประ	ทำได้บ่อยๆ	ทำได้บ่อยๆ	ทำได้บ่อยๆ	ทำได้บ่อยๆ	ทำได้เป็นบ
ทำได้บ่อยๆ	ทำได้บ่อยๆ	ทำได้บ่อยๆ	ทำได้เป็นบ	ทำได้เป็นบ	ทำได้บ้าง/	ทำได้บ่อยๆ	ทำได้เป็นบ	ทำได้บ่อยๆ
ทำเป็นประ	ทำเป็นประ	ทำเป็นประ	ทำได้บ่อยๆ	ทำได้บ่อยๆ	ทำได้เป็นบ	ทำได้เป็นบ	ทำเป็นประ	ทำเป็นประ
ทำได้บ่อยๆ	ทำได้เป็นบ	ทำได้บ่อยๆ	ทำได้บ่อยๆ	ทำเป็นประ	ทำได้เป็นบ	ทำได้บ่อยๆ	ทำได้บ่อยๆ	ทำได้บ่อยๆ
ทำได้บ่อยๆ	ทำได้บ้าง/	ทำได้เป็นบ	ทำได้บ่อยๆ	ทำได้เป็นบ	ทำได้เป็นบ	ทำได้บ่อยๆ	ทำได้บ่อยๆ	ทำได้เป็นบ
ทำเป็นประ	ทำเป็นประ	ทำเป็นประ	ทำเป็นประ	ทำเป็นประ	ทำเป็นประ	ทำเป็นประ	ทำเป็นประ	ทำเป็นประ
ทำได้เป็นบ	ทำได้บ่อยๆ	ทำได้เป็นบ	ทำได้บ่อยๆ	ทำได้บ้าง/	ทำได้บ่อยๆ	ทำได้เป็นบ	ทำได้เป็นบ	ทำได้บ้าง/
ทำได้บ่อยๆ	ทำได้บ่อยๆ	ทำเป็นประ	ทำได้บ่อยๆ	ทำได้เป็นบ	ทำได้บ่อยๆ	ทำได้เป็นบ	ทำได้บ้าง/	ทำได้เป็นบ
ทำเป็นประ	ทำเป็นประ	ทำเป็นประ	ทำได้เป็นบ	ทำได้บ่อยๆ	ทำได้เป็นบ	ทำเป็นประ	ทำเป็นประ	ทำเป็นประ
ทำได้บ่อยๆ	ทำเป็นประ	ทำเป็นประ	ทำได้บ่อยๆ	ทำได้บ่อยๆ	ทำได้เป็นบ	ทำได้บ่อยๆ	ทำเป็นประ	ทำเป็นประ
ทำได้เป็นบ	ทำได้บ่อยๆ	ทำได้บ่อยๆ	ทำได้บ้าง/	ทำได้บ้าง/	ทำได้บ้าง/	ทำได้เป็นบ	ทำได้เป็นบ	ทำได้เป็นบ
ทำได้เป็นบ	ทำเป็นประ	ทำเป็นประ	ทำได้บ่อยๆ	ทำได้บ่อยๆ	ทำได้บ่อยๆ	ทำเป็นประ	ทำได้เป็นบ	ทำเป็นประ
ทำได้บ่อยๆ	ทำได้บ่อยๆ	ทำเป็นประ	ทำได้บ่อยๆ	ทำได้บ่อยๆ	ทำได้บ่อยๆ	ทำได้บ่อยๆ	ทำได้บ่อยๆ	ทำได้บ่อยๆ
ทำได้บ่อยๆ	ทำได้เป็นบ	ทำได้บ่อยๆ	ทำได้บ่อยๆ	ทำได้บ่อยๆ	ทำได้เป็นบ	ทำได้บ่อยๆ	ทำได้เป็นบ	ทำได้บ่อยๆ
ทำได้บ่อยๆ	ทำได้บ่อยๆ	ทำได้บ่อยๆ	ทำได้บ่อยๆ	ทำได้บ่อยๆ	ทำได้เป็นบ	ทำได้บ่อยๆ	ทำได้บ่อยๆ	ทำได้เป็นบ
ทำได้เป็นบ	ไม่เคยทำเ	ทำเป็นประ	ทำเป็นประ	ทำได้เป็นบ	ทำได้เป็นบ	ทำได้เป็นบ	ทำได้บ่อยๆ	ทำได้บ้าง/
ทำได้เป็นบ	ทำได้เป็นบ	ทำได้บ่อยๆ	ทำได้บ้าง/	ทำได้เป็นบ	ทำได้เป็นบ	ทำได้เป็นบ	ทำได้เป็นบ	ทำได้เป็นบ

ทำได้บ่อยๆ	ทำได้บ่อยๆ	ทำได้บ่อยๆ	ทำได้บ่อยๆ	ทำได้บ่อยๆ	ทำได้เป็นบ	ทำได้บ่อยๆ	ทำได้เป็นบ	ทำได้บ่อยๆ
ทำได้เป็นบ	ทำได้เป็นบ	ทำเป็นประ	ทำได้บ่อยๆ	ทำได้บ่อยๆ	ทำได้เป็นบ	ทำได้เป็นบ	ทำได้เป็นบ	ทำได้บ่อยๆ
ทำเป็นประ	ทำเป็นประ	ทำเป็นประ	ทำเป็นประ	ทำเป็นประ	ทำได้บ่อยๆ	ทำได้บ่อยๆ	ทำเป็นประ	ทำเป็นประ
ไม่เคยทำเ	ไม่เคยทำเ	ทำได้บ้าง/	ทำได้บ้าง/	ทำได้บ้าง/	ทำได้บ้าง/	ทำได้บ่อยๆ	ทำได้บ่อยๆ	ทำได้เป็นบ
ทำเป็นประ	ทำเป็นประ	ทำเป็นประ	ทำเป็นประ	ทำเป็นประ	ทำได้บ่อยๆ	ทำได้บ่อยๆ	ทำเป็นประ	ทำเป็นประ
ทำได้เป็นบ	ทำได้บ่อยๆ	ทำได้บ่อยๆ	ทำได้เป็นบ	ทำได้บ่อยๆ	ทำได้เป็นบ	ทำได้เป็นบ	ทำได้เป็นบ	ทำได้เป็นบ
ทำได้เป็นบ	ทำได้เป็นบ	ทำได้เป็นบ	ทำได้เป็นบ	ทำได้เป็นบ	ทำได้เป็นบ	ทำได้เป็นบ	ทำได้เป็นบ	ทำได้เป็นบ
ทำเป็นประ	ทำได้บ่อยๆ	ทำเป็นประ	ทำเป็นประ	ทำเป็นประ	ทำเป็นประ	ทำเป็นประ	ทำเป็นประ	ทำเป็นประ
ทำเป็นประ	ทำเป็นประ	ทำเป็นประ	ทำเป็นประ	ทำเป็นประ	ทำเป็นประ	ทำเป็นประ	ทำเป็นประ	ทำเป็นประ
ทำได้บ่อยๆ	ทำได้เป็นบ	ทำได้บ่อยๆ	ทำเป็นประ	ทำเป็นประ	ทำเป็นประ	ทำเป็นประ	ทำเป็นประ	ทำได้บ่อยๆ
ทำเป็นประ	ทำเป็นประ	ทำเป็นประ	ทำเป็นประ	ทำเป็นประ	ทำเป็นประ	ทำเป็นประ	ทำเป็นประ	ทำเป็นประ
ทำได้บ้าง/	ไม่เคยทำเ	ไม่เคยทำเ	ทำได้เป็นบ	ทำได้เป็นบ	ทำได้บ้าง/	ทำได้บ้าง/	ทำได้เป็นบ	ทำได้เป็นบ
ทำได้บ้าง/	ไม่เคยทำเ	ไม่เคยทำเ	ไม่เคยทำเ	ไม่เคยทำเ	ไม่เคยทำเ	ไม่เคยทำเ	ไม่เคยทำเ	ไม่เคยทำเ
ทำได้เป็นบ	ทำได้บ่อยๆ	ทำได้บ่อยๆ	ทำได้บ่อยๆ	ทำได้บ่อยๆ	ทำได้เป็นบ	ทำได้เป็นบ	ทำได้เป็นบ	ทำได้บ่อยๆ
ทำได้บ่อยๆ	ทำได้บ้าง/	ทำได้เป็นบ	ทำได้บ่อยๆ	ทำได้บ่อยๆ	ทำได้เป็นบ	ทำได้เป็นบ	ทำได้บ่อยๆ	ทำเป็นประ
ทำได้บ้าง/	ทำได้บ้าง/	ทำได้บ่อยๆ	ทำได้เป็นบ	ทำได้บ้าง/	ทำได้บ้าง/	ทำได้บ่อยๆ	ทำได้เป็นบ	ทำได้เป็นบ
ทำได้บ่อยๆ	ทำเป็นประ	ทำเป็นประ	ทำได้เป็นบ	ทำเป็นประ	ทำได้บ่อยๆ	ทำได้บ่อยๆ	ทำเป็นประ	ทำเป็นประ
ทำเป็นประ	ทำเป็นประ	ทำเป็นประ	ทำเป็นประ	ทำเป็นประ	ทำเป็นประ	ทำเป็นประ	ทำเป็นประ	ทำเป็นประ
ทำเป็นประ	ทำเป็นประ	ทำเป็นประ	ทำเป็นประ	ทำเป็นประ	ทำได้บ่อยๆ	ทำได้บ่อยๆ	ทำเป็นประ	ทำเป็นประ
ทำเป็นประ	ทำได้บ้าง/	ทำได้เป็นบ	ทำได้บ้าง/	ไม่เคยทำเ	ไม่เคยทำเ	ทำได้เป็นบ	ทำได้เป็นบ	ทำได้เป็นบ
ทำเป็นประ	ทำเป็นประ	ทำได้บ่อยๆ	ไม่เคยทำเ	ไม่เคยทำเ	ไม่เคยทำเ	ไม่เคยทำเ	ไม่เคยทำเ	ไม่เคยทำเ
ไม่เคยทำเ	ทำได้เป็นบ	ทำได้เป็นบ	ไม่เคยทำเ	ทำได้บ่อยๆ	ทำได้เป็นบ	ทำได้เป็นบ	ไม่เคยทำเ	ไม่เคยทำเ
ทำได้บ้าง/	ทำได้บ้าง/	ทำได้บ่อยๆ	ทำได้เป็นบ	ทำได้บ่อยๆ	ทำได้บ่อยๆ	ทำได้บ่อยๆ	ทำได้บ่อยๆ	ทำได้บ่อยๆ
ทำได้บ้าง/	ทำได้บ้าง/	ทำได้เป็นบ	ทำได้เป็นบ	ทำได้บ่อยๆ	ทำได้บ่อยๆ	ทำได้บ่อยๆ	ทำได้บ่อยๆ	ทำได้บ่อยๆ
ทำได้เป็นบ	ทำได้บ่อยๆ	ทำได้บ่อยๆ	ทำได้บ่อยๆ	ทำได้บ่อยๆ	ทำได้บ่อยๆ	ทำได้บ่อยๆ	ทำได้บ่อยๆ	ทำได้บ่อยๆ
ทำได้บ่อยๆ	ทำได้บ่อยๆ	ทำได้บ่อยๆ	ทำเป็นประ	ทำเป็นประ	ทำเป็นประ	ทำเป็นประ	ทำได้บ่อยๆ	ทำได้บ่อยๆ
ทำเป็นประ	ทำได้เป็นบ	ทำเป็นประ	ทำเป็นประ	ทำเป็นประ	ทำเป็นประ	ทำเป็นประ	ทำเป็นประ	ทำเป็นประ
ทำได้บ่อยๆ	ไม่เคยทำเ	ทำเป็นประ	ทำเป็นประ	ทำเป็นประ	ทำเป็นประ	ทำได้บ่อยๆ	ทำเป็นประ	ทำเป็นประ
ทำเป็นประ	ทำได้เป็นบ	ทำได้เป็นบ	ทำได้เป็นบ	ทำได้บ่อยๆ	ทำได้เป็นบ	ทำได้เป็นบ	ทำได้เป็นบ	ทำได้เป็นบ
ทำเป็นประ	ทำได้เป็นบ	ทำได้บ่อยๆ	ทำได้บ่อยๆ	ทำได้เป็นบ	ทำได้บ่อยๆ	ทำเป็นประ	ทำเป็นประ	ทำได้บ่อยๆ
ทำเป็นประ	ทำได้เป็นบ	ทำเป็นประ	ทำได้บ้าง/	ทำได้บ้าง/	ทำได้บ้าง/	ทำได้บ่อยๆ	ทำได้บ่อยๆ	ทำได้บ่อยๆ
ทำเป็นประ	ทำเป็นประ	ทำเป็นประ	ทำเป็นประ	ทำเป็นประ	ทำเป็นประ	ทำเป็นประ	ทำเป็นประ	ทำเป็นประ
ทำได้เป็นบ	ทำได้เป็นบ	ทำเป็นประ	ทำได้บ่อยๆ	ทำได้บ่อยๆ	ทำได้เป็นบ	ทำได้เป็นบ	ทำได้เป็นบ	ทำได้บ่อยๆ
ไม่เคยทำเ	ไม่เคยทำเ	ทำได้บ้าง/	ทำได้บ้าง/	ทำได้บ้าง/	ทำได้บ้าง/	ทำได้บ่อยๆ	ทำได้บ่อยๆ	ทำได้เป็นบ
ทำเป็นประ	ทำเป็นประ	ทำเป็นประ	ทำเป็นประ	ทำเป็นประ	ทำได้บ่อยๆ	ทำได้บ่อยๆ	ทำเป็นประ	ทำเป็นประ
ทำได้เป็นบ	ทำได้บ่อยๆ	ทำได้บ่อยๆ	ทำได้เป็นบ	ทำได้บ่อยๆ	ทำได้เป็นบ	ทำได้เป็นบ	ทำได้เป็นบ	ทำได้เป็นบ
ทำได้เป็นบ	ทำได้เป็นบ	ทำได้เป็นบ	ทำได้เป็นบ	ทำได้เป็นบ	ทำได้เป็นบ	ทำได้เป็นบ	ทำได้เป็นบ	ทำได้เป็นบ
ทำเป็นประ	ทำได้บ่อยๆ	ทำเป็นประ	ทำเป็นประ	ทำเป็นประ	ทำเป็นประ	ทำเป็นประ	ทำเป็นประ	ทำเป็นประ
ทำเป็นประ	ทำเป็นประ	ทำเป็นประ	ทำเป็นประ	ทำเป็นประ	ทำเป็นประ	ทำเป็นประ	ทำเป็นประ	ทำเป็นประ
ทำได้บ่อยๆ	ทำได้เป็นบ	ทำได้บ่อยๆ	ทำเป็นประ	ทำเป็นประ	ทำเป็นประ	ทำเป็นประ	ทำเป็นประ	ทำได้บ่อยๆ
ทำเป็นประ	ทำเป็นประ	ทำเป็นประ	ทำเป็นประ	ทำเป็นประ	ทำเป็นประ	ทำเป็นประ	ทำเป็นประ	ทำเป็นประ
ทำได้บ้าง/	ไม่เคยทำเ	ไม่เคยทำเ	ทำได้เป็นบ	ทำได้เป็นบ	ทำได้บ้าง/	ทำได้บ้าง/	ทำได้เป็นบ	ทำได้เป็นบ
ทำได้บ้าง/	ไม่เคยทำเ	ไม่เคยทำเ	ไม่เคยทำเ	ไม่เคยทำเ	ไม่เคยทำเ	ไม่เคยทำเ	ไม่เคยทำเ	ไม่เคยทำเ
ทำได้เป็นบ	ทำได้บ่อยๆ	ทำได้บ่อยๆ	ทำได้บ่อยๆ	ทำได้บ่อยๆ	ทำได้เป็นบ	ทำได้เป็นบ	ทำได้เป็นบ	ทำได้บ่อยๆ
ทำได้บ่อยๆ	ทำได้บ้าง/	ทำได้เป็นบ	ทำได้บ่อยๆ	ทำได้บ่อยๆ	ทำได้เป็นบ	ทำได้เป็นบ	ทำได้บ่อยๆ	ทำเป็นประ
ทำได้บ้าง/	ทำได้บ้าง/	ทำได้บ่อยๆ	ทำได้เป็นบ	ทำได้บ้าง/	ทำได้บ้าง/	ทำได้บ่อยๆ	ทำได้เป็นบ	ทำได้เป็นบ
ทำได้บ่อยๆ	ทำเป็นประ	ทำเป็นประ	ทำได้เป็นบ	ทำเป็นประ	ทำได้บ่อยๆ	ทำได้บ่อยๆ	ทำเป็นประ	ทำเป็นประ
ทำเป็นประ	ทำเป็นประ	ทำเป็นประ	ทำเป็นประ	ทำเป็นประ	ทำได้บ่อยๆ	ทำได้บ่อยๆ	ทำเป็นประ	ทำเป็นประ
ทำเป็นประ	ทำเป็นประ	ทำเป็นประ	ทำเป็นประ	ทำเป็นประ	ทำได้บ่อยๆ	ทำได้บ่อยๆ	ทำเป็นประ	ทำเป็นประ
ทำเป็นประ	ทำได้บ้าง/	ทำได้เป็นบ	ไม่เคยทำเ	ไม่เคยทำเ	ไม่เคยทำเ	ทำได้เป็นบ	ทำได้เป็นบ	ทำได้เป็นบ
ทำเป็นประ	ทำเป็นประ	ทำได้บ่อยๆ	ไม่เคยทำเ	ไม่เคยทำเ	ไม่เคยทำเ	ไม่เคยทำเ	ไม่เคยทำเ	ไม่เคยทำเ

ไม่เคยทำเ	ทำได้เป็นบ	ทำได้เป็นบ	ไม่เคยทำเ	ทำได้บ่อยๆ	ทำได้เป็นบ	ทำได้เป็นบ	ไม่เคยทำเ	ไม่เคยทำเ
ทำได้บ้าง/	ทำได้บ้าง/	ทำได้บ่อยๆ	ทำได้เป็นบ	ทำได้บ่อยๆ	ทำได้บ่อยๆ	ทำได้บ่อยๆ	ทำได้บ่อยๆ	ทำได้บ่อยๆ
ทำได้บ้าง/	ทำได้บ้าง/	ทำได้เป็นบ	ทำได้เป็นบ	ทำได้บ่อยๆ	ทำได้บ่อยๆ	ทำได้บ่อยๆ	ทำได้บ่อยๆ	ทำได้บ่อยๆ
ทำได้เป็นบ	ทำได้บ่อยๆ	ทำได้บ่อยๆ	ทำได้บ่อยๆ	ทำได้บ่อยๆ	ทำได้บ่อยๆ	ทำได้บ่อยๆ	ทำได้บ่อยๆ	ทำได้บ่อยๆ
ทำได้บ่อยๆ	ทำได้บ่อยๆ	ทำได้บ่อยๆ	ทำเป็นประ	ทำเป็นประ	ทำเป็นประ	ทำเป็นประ	ทำได้บ่อยๆ	ทำได้บ่อยๆ
ทำได้เป็นบ	ทำได้เป็นบ	ทำได้บ้าง/	ทำได้เป็นบ	ทำได้เป็นบ	ไม่เคยทำเ	ไม่เคยทำเ	ทำได้บ้าง/	ทำได้เป็นบ
ทำเป็นประ	ทำได้บ่อยๆ	ทำเป็นประ	ทำเป็นประ	ทำเป็นประ	ทำได้บ่อยๆ	ทำเป็นประ	ทำเป็นประ	ทำเป็นประ
ทำได้บ่อยๆ	ทำได้บ่อยๆ	ทำได้บ่อยๆ	ทำได้บ่อยๆ	ทำได้บ่อยๆ	ทำได้บ่อยๆ	ทำได้บ่อยๆ	ทำได้บ่อยๆ	ทำได้บ่อยๆ
ทำได้บ่อยๆ	ทำได้บ่อยๆ	ทำได้บ่อยๆ	ทำเป็นประ	ทำเป็นประ	ทำเป็นประ	ทำเป็นประ	ทำได้บ่อยๆ	ทำได้บ่อยๆ
ทำได้เป็นบ	ทำได้เป็นบ	ทำเป็นประ	ทำได้บ่อยๆ	ทำได้บ่อยๆ	ทำได้เป็นบ	ทำได้เป็นบ	ทำได้เป็นบ	ทำได้บ่อยๆ
ไม่เคยทำเ	ไม่เคยทำเ	ทำได้บ้าง/	ทำได้บ้าง/	ทำได้บ้าง/	ทำได้บ้าง/	ทำได้บ่อยๆ	ทำได้บ่อยๆ	ทำได้เป็นบ
ทำเป็นประ	ทำเป็นประ	ทำเป็นประ	ทำเป็นประ	ทำเป็นประ	ทำได้บ่อยๆ	ทำได้บ่อยๆ	ทำเป็นประ	ทำเป็นประ
ทำได้เป็นบ	ทำได้บ่อยๆ	ทำได้บ่อยๆ	ทำได้เป็นบ	ทำได้บ่อยๆ	ทำได้เป็นบ	ทำได้เป็นบ	ทำได้เป็นบ	ทำได้เป็นบ
ทำได้เป็นบ	ทำได้เป็นบ	ทำได้เป็นบ	ทำได้เป็นบ	ทำได้เป็นบ	ทำได้เป็นบ	ทำได้เป็นบ	ทำได้เป็นบ	ทำได้เป็นบ
ทำเป็นประ	ทำได้บ่อยๆ	ทำเป็นประ	ทำเป็นประ	ทำเป็นประ	ทำเป็นประ	ทำเป็นประ	ทำเป็นประ	ทำเป็นประ
ทำเป็นประ	ทำเป็นประ	ทำเป็นประ	ทำเป็นประ	ทำเป็นประ	ทำเป็นประ	ทำเป็นประ	ทำเป็นประ	ทำเป็นประ
ทำได้บ่อยๆ	ทำได้เป็นบ	ทำได้บ่อยๆ	ทำเป็นประ	ทำเป็นประ	ทำเป็นประ	ทำเป็นประ	ทำเป็นประ	ทำได้บ่อยๆ
ทำเป็นประ	ทำเป็นประ	ทำเป็นประ	ทำเป็นประ	ทำเป็นประ	ทำเป็นประ	ทำเป็นประ	ทำเป็นประ	ทำเป็นประ
ทำได้บ้าง/	ไม่เคยทำเ	ไม่เคยทำเ	ทำได้เป็นบ	ทำได้เป็นบ	ทำได้บ้าง/	ทำได้บ้าง/	ทำได้เป็นบ	ทำได้เป็นบ
ไม่เคยทำเ	ทำได้บ้าง/	ไม่เคยทำเ	ไม่เคยทำเ	ไม่เคยทำเ	ไม่เคยทำเ	ไม่เคยทำเ	ไม่เคยทำเ	ไม่เคยทำเ
ทำได้เป็นบ	ทำได้บ่อยๆ	ทำได้บ่อยๆ	ทำได้บ่อยๆ	ทำได้บ่อยๆ	ทำได้เป็นบ	ทำได้เป็นบ	ทำได้เป็นบ	ทำได้บ่อยๆ
ทำได้บ่อยๆ	ทำได้บ้าง/	ทำได้เป็นบ	ทำได้บ่อยๆ	ทำได้บ่อยๆ	ทำได้เป็นบ	ทำได้เป็นบ	ทำได้บ่อยๆ	ทำเป็นประ
ทำได้บ้าง/	ทำได้บ้าง/	ทำได้บ่อยๆ	ทำได้เป็นบ	ทำได้บ้าง/	ทำได้บ้าง/	ทำได้บ่อยๆ	ทำได้เป็นบ	ทำได้เป็นบ
ทำได้บ่อยๆ	ทำเป็นประ	ทำเป็นประ	ทำได้เป็นบ	ทำเป็นประ	ทำได้บ่อยๆ	ทำได้บ่อยๆ	ทำเป็นประ	ทำเป็นประ
ทำได้บ่อยๆ	ทำได้บ่อยๆ	ทำได้บ่อยๆ	ทำได้บ่อยๆ	ทำได้บ่อยๆ	ทำได้บ่อยๆ	ทำได้บ่อยๆ	ทำได้บ่อยๆ	ทำได้บ่อยๆ
ทำได้เป็นบ	ทำได้เป็นบ	ทำได้บ่อยๆ	ทำได้บ่อยๆ	ทำได้เป็นบ	ทำได้เป็นบ	ทำได้เป็นบ	ทำได้เป็นบ	ทำได้บ่อยๆ
ทำเป็นประ	ทำเป็นประ	ทำเป็นประ	ไม่เคยทำเ	ทำเป็นประ	ทำเป็นประ	ทำเป็นประ	ทำเป็นประ	ทำเป็นประ
ทำได้เป็นบ	ทำได้เป็นบ	ทำได้เป็นบ	ทำได้เป็นบ	ไม่เคยทำเ	ทำได้เป็นบ	ทำได้บ้าง/	ทำได้เป็นบ	ทำได้เป็นบ
ทำได้บ่อยๆ	ทำได้บ่อยๆ	ทำได้บ่อยๆ	ทำได้บ่อยๆ	ทำได้บ่อยๆ	ทำได้บ่อยๆ	ทำได้บ่อยๆ	ทำได้บ่อยๆ	ทำได้บ่อยๆ
ทำได้เป็นบ	ทำได้เป็นบ	ทำได้เป็นบ	ทำได้เป็นบ	ทำได้เป็นบ	ทำได้เป็นบ	ทำได้เป็นบ	ทำได้เป็นบ	ทำได้เป็นบ
ทำเป็นประ	ทำเป็นประ	ทำเป็นประ	ทำเป็นประ	ทำเป็นประ	ทำเป็นประ	ทำเป็นประ	ทำเป็นประ	ทำเป็นประ
ทำเป็นประ	ทำได้บ้าง/	ทำได้บ้าง/	ทำได้บ่อยๆ	ทำได้บ้าง/	ทำได้บ่อยๆ	ทำได้บ่อยๆ	ทำได้บ่อยๆ	ทำได้บ่อยๆ
ทำเป็นประ	ทำเป็นประ	ทำได้บ่อยๆ	ทำเป็นประ	ทำได้บ่อยๆ	ทำได้บ่อยๆ	ทำได้บ่อยๆ	ทำได้บ่อยๆ	ทำได้บ่อยๆ
ทำได้เป็นบ	ทำได้เป็นบ	ทำได้เป็นบ	ทำได้เป็นบ	ทำได้เป็นบ	ทำได้เป็นบ	ทำได้เป็นบ	ทำได้เป็นบ	ทำได้เป็นบ
ทำเป็นประ	ทำเป็นประ	ทำเป็นประ	ทำเป็นประ	ทำเป็นประ	ทำเป็นประ	ทำเป็นประ	ทำเป็นประ	ทำเป็นประ
ทำได้เป็นบ	ทำได้บ่อยๆ	ทำได้เป็นบ	ทำได้บ่อยๆ	ทำได้บ่อยๆ	ทำได้บ่อยๆ	ทำได้บ่อยๆ	ทำได้เป็นบ	ทำได้เป็นบ
ทำเป็นประ	ทำเป็นประ	ทำเป็นประ	ทำเป็นประ	ทำเป็นประ	ทำเป็นประ	ทำเป็นประ	ทำเป็นประ	ทำเป็นประ
ทำเป็นประ	ทำได้เป็นบ	ทำได้บ่อยๆ	ทำได้บ่อยๆ	ทำได้บ่อยๆ	ทำได้บ่อยๆ	ทำได้บ่อยๆ	ทำได้บ่อยๆ	ทำได้บ่อยๆ
ทำได้บ่อยๆ	ทำได้บ่อยๆ	ทำได้บ่อยๆ	ทำได้เป็นบ	ทำได้เป็นบ	ทำได้เป็นบ	ทำได้เป็นบ	ทำได้เป็นบ	ทำได้เป็นบ
ทำเป็นประ	ทำเป็นประ	ทำเป็นประ	ทำเป็นประ	ทำเป็นประ	ทำเป็นประ	ทำเป็นประ	ทำเป็นประ	ทำเป็นประ
ทำได้บ่อยๆ	ทำได้บ่อยๆ	ทำได้บ่อยๆ	ทำได้บ่อยๆ	ทำได้บ่อยๆ	ทำได้บ่อยๆ	ทำได้บ่อยๆ	ทำได้บ่อยๆ	ทำได้บ่อยๆ
ทำได้บ่อยๆ	ทำได้บ่อยๆ	ทำได้บ่อยๆ	ทำได้บ่อยๆ	ทำได้บ่อยๆ	ทำได้บ่อยๆ	ทำได้บ่อยๆ	ทำได้บ่อยๆ	ทำได้บ่อยๆ
ทำเป็นประ	ทำเป็นประ	ทำเป็นประ	ทำเป็นประ	ทำเป็นประ	ทำเป็นประ	ทำเป็นประ	ทำเป็นประ	ทำเป็นประ
ทำเป็นประ	ทำเป็นประ	ทำเป็นประ	ทำเป็นประ	ทำเป็นประ	ทำเป็นประ	ทำเป็นประ	ทำเป็นประ	ทำเป็นประ
ทำได้บ่อยๆ	ทำได้บ่อยๆ	ทำได้บ่อยๆ	ทำได้บ่อยๆ	ทำได้บ่อยๆ	ทำได้บ่อยๆ	ทำได้บ่อยๆ	ทำได้บ่อยๆ	ทำได้บ่อยๆ
ทำได้เป็นบ	ทำได้เป็นบ	ทำได้บ่อยๆ	ทำได้เป็นบ	ทำได้เป็นบ	ทำได้เป็นบ	ทำได้เป็นบ	ทำได้เป็นบ	ทำได้เป็นบ
ทำได้บ่อยๆ	ทำได้บ่อยๆ	ทำได้บ่อยๆ	ทำได้บ่อยๆ	ทำได้บ่อยๆ	ทำได้บ่อยๆ	ทำได้บ่อยๆ	ทำได้บ่อยๆ	ทำได้บ่อยๆ
ทำได้เป็นบ	ทำได้เป็นบ	ทำได้เป็นบ	ทำได้เป็นบ	ทำได้เป็นบ	ทำได้เป็นบ	ทำได้เป็นบ	ทำได้เป็นบ	ทำได้เป็นบ
ทำเป็นประ	ทำเป็นประ	ทำได้บ่อยๆ	ทำได้บ่อยๆ	ทำได้บ่อยๆ	ทำได้บ่อยๆ	ทำได้บ่อยๆ	ทำเป็นประ	ทำเป็นประ
ไม่เคยทำเ	ทำได้บ้าง/	ทำได้เป็นบ	ทำได้เป็นบ	ทำได้เป็นบ	ทำได้เป็นบ	ทำได้เป็นบ	ทำได้บ่อยๆ	ทำได้เป็นบ
ทำเป็นประ	ทำได้บ่อยๆ	ทำเป็นประ	ทำได้บ่อยๆ	ทำเป็นประ	ทำเป็นประ	ทำได้บ่อยๆ	ทำได้บ่อยๆ	ทำได้บ่อยๆ

ทำได้เป็นบ	ทำได้เป็นบ	ทำได้บ้าง/	ทำได้เป็นบ	ทำได้เป็นบ	ทำได้เป็นบ	ทำได้บ่อยๆ	ทำได้บ่อยๆ	ทำได้บ่อยๆ
ไม่เคยทำเล	ไม่เคยทำเล	ไม่เคยทำเล	ไม่เคยทำเล	ไม่เคยทำเล	ไม่เคยทำเล	ไม่เคยทำเล	ไม่เคยทำเล	ไม่เคยทำเล
ทำได้บ่อยๆ	ทำได้บ่อยๆ	ทำเป็นประ	ทำเป็นประ	ทำเป็นประ	ทำได้บ่อยๆ	ทำได้บ่อยๆ	ทำเป็นประ	ทำได้บ่อยๆ
ทำเป็นประ	ทำได้เป็นบ	ทำได้เป็นบ	ทำได้บ่อยๆ	ทำเป็นประ	ทำได้บ่อยๆ	ทำได้บ่อยๆ	ทำได้บ่อยๆ	ทำเป็นประ
ทำได้เป็นบ	ทำได้เป็นบ	ทำได้บ่อยๆ	ทำได้เป็นบ	ทำได้บ่อยๆ	ทำได้บ่อยๆ	ทำได้เป็นบ	ทำได้บ่อยๆ	ทำได้บ่อยๆ
ทำเป็นประ	ทำได้บ่อยๆ	ทำได้บ่อยๆ	ทำได้บ่อยๆ	ทำได้บ่อยๆ	ทำได้บ่อยๆ	ทำได้บ่อยๆ	ทำได้บ่อยๆ	ทำเป็นประ
ทำเป็นประ	ทำเป็นประ	ทำเป็นประ	ไม่เคยทำเล	ไม่เคยทำเล	ไม่เคยทำเล	ไม่เคยทำเล	ไม่เคยทำเล	ไม่เคยทำเล
ทำได้เป็นบ	ทำได้เป็นบ	ทำได้เป็นบ	ทำเป็นประ	ทำได้บ่อยๆ	ทำได้เป็นบ	ทำได้เป็นบ	ทำได้บ่อยๆ	ทำได้เป็นบ
ทำเป็นประ	ทำได้บ่อยๆ	ทำเป็นประ	ทำได้บ่อยๆ	ทำได้บ่อยๆ	ทำเป็นประ	ทำเป็นประ	ทำเป็นประ	ทำได้บ่อยๆ
ทำเป็นประ	ทำเป็นประ	ทำเป็นประ	ทำเป็นประ	ทำเป็นประ	ทำได้เป็นบ	ทำเป็นประ	ทำเป็นประ	ทำเป็นประ
ทำได้เป็นบ	ทำได้เป็นบ	ทำได้เป็นบ	ทำเป็นประ	ทำได้บ่อยๆ	ทำได้เป็นบ	ทำได้เป็นบ	ทำได้บ่อยๆ	ทำได้เป็นบ
ทำได้เป็นบ	ทำได้บ่อยๆ	ทำได้บ่อยๆ	ทำได้บ่อยๆ	ทำได้บ่อยๆ	ทำได้เป็นบ	ทำได้บ่อยๆ	ทำได้เป็นบ	ทำได้บ่อยๆ
ทำได้เป็นบ	ไม่เคยทำเล	ไม่เคยทำเล	ไม่เคยทำเล	ทำได้บ้าง/	ทำได้เป็นบ	ทำได้เป็นบ	ทำได้เป็นบ	ทำได้เป็นบ
ทำเป็นประ	ทำเป็นประ	ทำเป็นประ	ทำได้บ้าง/	ทำเป็นประ	ทำเป็นประ	ทำเป็นประ	ทำเป็นประ	ทำเป็นประ
ทำได้เป็นบ	ทำได้บ่อยๆ	ทำได้บ่อยๆ	ทำได้บ่อยๆ	ทำเป็นประ	ทำได้บ่อยๆ	ทำได้บ่อยๆ	ทำได้บ่อยๆ	ทำได้เป็นบ
ทำได้บ่อยๆ	ทำได้เป็นบ	ทำได้เป็นบ	ทำได้บ่อยๆ	ไม่เคยทำเล	ทำได้บ่อยๆ	ทำได้บ่อยๆ	ทำได้บ่อยๆ	ทำได้บ่อยๆ
ทำเป็นประ	ทำได้บ่อยๆ	ทำได้บ่อยๆ	ทำเป็นประ	ทำเป็นประ	ทำเป็นประ	ทำเป็นประ	ทำได้บ่อยๆ	ทำได้บ่อยๆ
ทำได้บ่อยๆ	ทำได้บ่อยๆ	ทำได้บ่อยๆ	ทำได้บ่อยๆ	ทำได้เป็นบ	ทำได้บ่อยๆ	ทำได้เป็นบ	ทำได้บ่อยๆ	ทำได้บ่อยๆ
ทำได้บ่อยๆ	ไม่เคยทำเล	ทำได้เป็นบ	ทำได้บ่อยๆ	ทำได้บ่อยๆ	ทำได้เป็นบ	ทำได้เป็นบ	ทำได้เป็นบ	ทำได้บ่อยๆ
ทำได้บ่อยๆ	ทำเป็นประ	ทำเป็นประ	ทำเป็นประ	ทำเป็นประ	ทำได้เป็นบ	ทำเป็นประ	ทำเป็นประ	ทำเป็นประ
ทำได้บ่อยๆ	ทำได้เป็นบ	ทำได้บ่อยๆ	ทำได้เป็นบ	ทำได้บ่อยๆ	ทำได้บ่อยๆ	ทำได้บ่อยๆ	ทำได้บ่อยๆ	ทำได้บ่อยๆ
ทำเป็นประ	ทำเป็นประ	ทำได้บ่อยๆ	ทำเป็นประ	ทำเป็นประ	ทำเป็นประ	ทำเป็นประ	ทำได้บ่อยๆ	ทำได้บ่อยๆ
ทำได้บ่อยๆ	ทำได้เป็นบ	ทำได้บ่อยๆ	ทำได้บ่อยๆ	ทำได้บ่อยๆ	ทำได้บ่อยๆ	ทำได้บ่อยๆ	ทำได้บ่อยๆ	ทำได้บ่อยๆ
ทำได้เป็นบ	ทำเป็นประ	ทำเป็นประ	ทำเป็นประ	ทำเป็นประ	ทำได้บ่อยๆ	ทำได้บ่อยๆ	ทำได้บ่อยๆ	ทำได้บ่อยๆ
ทำได้บ่อยๆ	ทำได้เป็นบ	ทำได้บ่อยๆ	ทำได้บ่อยๆ	ทำได้บ่อยๆ	ทำได้บ้าง/	ทำได้เป็นบ	ทำเป็นประ	ทำเป็นประ
ทำเป็นประ	ทำเป็นประ	ทำเป็นประ	ทำเป็นประ	ทำได้บ่อยๆ	ทำได้บ่อยๆ	ทำได้บ่อยๆ	ทำได้บ่อยๆ	ทำได้บ่อยๆ
ทำได้เป็นบ	ไม่เคยทำเล	ทำได้บ่อยๆ	ทำได้บ้าง/	ทำได้บ้าง/	ทำได้เป็นบ	ทำได้บ้าง/	ทำได้บ่อยๆ	ทำได้เป็นบ
ทำได้บ่อยๆ	ทำได้บ่อยๆ	ทำได้เป็นบ	ทำได้บ่อยๆ	ทำได้บ่อยๆ	ทำได้เป็นบ	ทำได้บ่อยๆ	ทำเป็นประ	ทำเป็นประ
ทำได้บ่อยๆ	ทำได้บ้าง/	ทำได้เป็นบ	ทำได้บ่อยๆ	ทำได้บ่อยๆ	ทำได้บ่อยๆ	ทำได้บ่อยๆ	ทำได้บ่อยๆ	ทำได้บ่อยๆ
ทำได้บ่อยๆ	ทำได้เป็นบ	ไม่เคยทำเล	ไม่เคยทำเล	ไม่เคยทำเล	ไม่เคยทำเล	ทำได้บ่อยๆ	ทำได้บ่อยๆ	ไม่เคยทำเล
ทำเป็นประ	ทำเป็นประ	ทำเป็นประ	ทำได้บ่อยๆ	ทำได้บ่อยๆ	ทำเป็นประ	ทำเป็นประ	ทำได้บ่อยๆ	ทำได้บ่อยๆ
ทำได้เป็นบ	ทำได้บ้าง/	ทำเป็นประ	ทำเป็นประ	ทำได้บ่อยๆ	ทำได้บ่อยๆ	ทำได้บ่อยๆ	ทำเป็นประ	ทำเป็นประ
ทำได้บ่อยๆ	ทำได้บ่อยๆ	ทำเป็นประ	ทำได้บ่อยๆ	ทำได้เป็นบ	ทำได้เป็นบ	ทำได้เป็นบ	ทำได้เป็นบ	ทำได้เป็นบ
ทำได้เป็นบ	ทำได้เป็นบ	ทำได้บ่อยๆ	ทำได้บ่อยๆ	ทำได้เป็นบ	ทำได้บ่อยๆ	ทำได้บ่อยๆ	ทำเป็นประ	ทำเป็นประ
ทำได้เป็นบ	ทำได้บ่อยๆ	ทำเป็นประ	ทำได้บ่อยๆ	ทำเป็นประ	ทำได้บ่อยๆ	ทำได้บ่อยๆ	ทำเป็นประ	ทำได้บ่อยๆ
ทำเป็นประ	ทำได้บ่อยๆ	ทำเป็นประ	ทำเป็นประ	ทำเป็นประ	ทำได้บ่อยๆ	ทำได้บ่อยๆ	ทำเป็นประ	ทำเป็นประ
ทำเป็นประ	ทำได้บ่อยๆ	ทำได้บ่อยๆ	ทำได้บ่อยๆ	ทำได้บ่อยๆ	ทำได้บ่อยๆ	ทำเป็นประ	ทำได้บ่อยๆ	ทำได้บ่อยๆ
ทำได้เป็นบ	ทำได้บ่อยๆ	ทำได้บ่อยๆ	ทำได้เป็นบ	ทำได้เป็นบ	ทำได้เป็นบ	ทำได้บ้าง/	ทำได้เป็นบ	ทำได้เป็นบ
ทำเป็นประ	ทำเป็นประ	ทำได้บ่อยๆ	ทำได้บ่อยๆ	ทำได้บ่อยๆ	ทำได้บ่อยๆ	ทำได้บ่อยๆ	ทำเป็นประ	ทำเป็นประ
ทำเป็นประ	ทำเป็นประ	ทำได้บ่อยๆ	ทำได้บ่อยๆ	ทำได้บ่อยๆ	ทำได้บ่อยๆ	ทำได้บ่อยๆ	ทำเป็นประ	ทำเป็นประ
ไม่เคยทำเล	ทำได้บ้าง/	ทำได้เป็นบ	ทำได้เป็นบ	ทำได้เป็นบ	ทำได้เป็นบ	ทำได้เป็นบ	ทำได้บ่อยๆ	ทำได้เป็นบ
ทำเป็นประ	ทำได้บ่อยๆ	ทำเป็นประ	ทำได้บ่อยๆ	ทำเป็นประ	ทำเป็นประ	ทำได้บ่อยๆ	ทำได้บ่อยๆ	ทำได้บ่อยๆ
ทำได้เป็นบ	ทำได้เป็นบ	ทำได้บ้าง/	ทำได้เป็นบ	ทำได้เป็นบ	ทำได้เป็นบ	ทำได้บ่อยๆ	ทำได้บ่อยๆ	ทำได้บ่อยๆ
ไม่เคยทำเล	ไม่เคยทำเล	ไม่เคยทำเล	ไม่เคยทำเล	ไม่เคยทำเล	ไม่เคยทำเล	ไม่เคยทำเล	ไม่เคยทำเล	ไม่เคยทำเล
ทำได้บ่อยๆ	ทำได้บ่อยๆ	ทำเป็นประ	ทำเป็นประ	ทำเป็นประ	ทำได้บ่อยๆ	ทำได้บ่อยๆ	ทำเป็นประ	ทำได้บ่อยๆ
ทำเป็นประ	ทำได้เป็นบ	ทำได้เป็นบ	ทำได้บ่อยๆ	ทำเป็นประ	ทำได้บ่อยๆ	ทำได้บ่อยๆ	ทำได้บ่อยๆ	ทำเป็นประ
ทำได้เป็นบ	ทำเป็นประ	ทำเป็นประ	ทำได้บ่อยๆ	ทำได้บ่อยๆ	ทำได้บ่อยๆ	ทำเป็นประ	ทำเป็นประ	ทำได้บ่อยๆ
ทำได้บ่อยๆ	ทำได้บ่อยๆ	ทำได้บ่อยๆ	ทำได้เป็นบ	ทำได้เป็นบ	ทำได้เป็นบ	ทำได้เป็นบ	ทำได้บ่อยๆ	ทำได้บ่อยๆ
ทำเป็นประ	ทำเป็นประ	ทำเป็นประ	ทำได้บ่อยๆ	ทำได้บ่อยๆ	ทำเป็นประ	ทำเป็นประ	ทำได้บ่อยๆ	ทำได้บ่อยๆ

Sum_HeL	oral1	oral2	oral3	oral4	oral5	oral6	oral7	oral8
43	ปฏิบัติประจำ	ปฏิบัติประจำ	ปฏิบัติประจำ	ปฏิบัติประจำ	ปฏิบัติบางค	ปฏิบัติประจำ	ปฏิบัติบ่อย	ปฏิบัติบางค
47	ปฏิบัติประจำ	ปฏิบัติประจำ	ปฏิบัติบางค	ปฏิบัติประจำ	ปฏิบัติบางค	ปฏิบัติประจำ	ปฏิบัติบางค	ปฏิบัติบ่อย
40	ปฏิบัติประจำ	ปฏิบัติประจำ	ปฏิบัติบ่อย	ปฏิบัติประจำ	ไม่ปฏิบัติเล	ปฏิบัติบ่อย	ปฏิบัติบางค	ปฏิบัติบ่อย
46	ปฏิบัติประจำ	ปฏิบัติประจำ	ปฏิบัติประจำ	ปฏิบัติประจำ	ไม่ปฏิบัติเล	ปฏิบัติประจำ	ปฏิบัติบางค	ไม่ปฏิบัติเล
20	ปฏิบัติประจำ	ปฏิบัติบางค	ปฏิบัติบ่อย	ปฏิบัติบางค	ไม่ปฏิบัติเล	ไม่ปฏิบัติเล	ปฏิบัติบางค	ปฏิบัติบ่อย
37	ปฏิบัติประจำ	ปฏิบัติประจำ	ปฏิบัติประจำ	ปฏิบัติประจำ	ไม่ปฏิบัติเล	ปฏิบัติประจำ	ปฏิบัติประจำ	ปฏิบัติประจำ
14	ปฏิบัติบางค	ปฏิบัติบางค	ปฏิบัติบ่อย	ปฏิบัติบ่อย	ไม่ปฏิบัติเล	ปฏิบัติบางค	ปฏิบัติบางค	ปฏิบัติบ่อย
55	ปฏิบัติประจำ	ปฏิบัติประจำ	ปฏิบัติประจำ	ปฏิบัติประจำ	ปฏิบัติประจำ	ปฏิบัติประจำ	ปฏิบัติบางค	ปฏิบัติบางค
28	ปฏิบัติบ่อย	ปฏิบัติบางค	ปฏิบัติบางค	ปฏิบัติบ่อย	ปฏิบัติบ่อย	ปฏิบัติบางค	ปฏิบัติบางค	ปฏิบัติบ่อย
42	ปฏิบัติประจำ	ปฏิบัติบ่อย	ปฏิบัติประจำ	ปฏิบัติประจำ	ไม่ปฏิบัติเล	ปฏิบัติบางค	ปฏิบัติประจำ	ปฏิบัติประจำ
33	ปฏิบัติประจำ	ปฏิบัติประจำ	ปฏิบัติประจำ	ปฏิบัติบางค	ปฏิบัติบางค	ปฏิบัติบ่อย	ปฏิบัติประจำ	ปฏิบัติบ่อย
51	ปฏิบัติประจำ	ปฏิบัติประจำ	ปฏิบัติประจำ	ปฏิบัติบางค	ไม่ปฏิบัติเล	ปฏิบัติประจำ	ปฏิบัติประจำ	ปฏิบัติบางค
32	ปฏิบัติประจำ	ปฏิบัติประจำ	ปฏิบัติประจำ	ปฏิบัติประจำ	ปฏิบัติบางค	ปฏิบัติบางค	ปฏิบัติบางค	ปฏิบัติบางค
45	ปฏิบัติบางค	ปฏิบัติบ่อย	ปฏิบัติบางค	ปฏิบัติบ่อย	ปฏิบัติบางค	ปฏิบัติบ่อย	ปฏิบัติบ่อย	ปฏิบัติบ่อย
31	ปฏิบัติประจำ	ปฏิบัติประจำ	ปฏิบัติประจำ	ปฏิบัติบางค	ปฏิบัติบางค	ปฏิบัติประจำ	ปฏิบัติบางค	ปฏิบัติบางค
30	ปฏิบัติบางค	ปฏิบัติบางค	ปฏิบัติบ่อย	ปฏิบัติบ่อย	ปฏิบัติบางค	ปฏิบัติบ่อย	ปฏิบัติบางค	ปฏิบัติบางค
27	ปฏิบัติประจำ	ปฏิบัติประจำ	ปฏิบัติบางค	ปฏิบัติประจำ	ปฏิบัติบางค	ปฏิบัติประจำ	ปฏิบัติประจำ	ปฏิบัติบางค
39	ปฏิบัติประจำ	ปฏิบัติบ่อย	ปฏิบัติบ่อย	ปฏิบัติบางค	ไม่ปฏิบัติเล	ปฏิบัติบางค	ปฏิบัติบางค	ปฏิบัติบางค
39	ปฏิบัติประจำ	ปฏิบัติบางค	ปฏิบัติประจำ	ปฏิบัติประจำ	ปฏิบัติบางค	ไม่ปฏิบัติเล	ปฏิบัติบ่อย	ปฏิบัติบางค
39	ปฏิบัติประจำ	ปฏิบัติประจำ	ปฏิบัติประจำ	ปฏิบัติประจำ	ปฏิบัติบางค	ปฏิบัติประจำ	ปฏิบัติบ่อย	ปฏิบัติบางค
40	ปฏิบัติประจำ	ปฏิบัติบ่อย	ปฏิบัติประจำ	ปฏิบัติบางค	ปฏิบัติประจำ	ปฏิบัติบ่อย	ปฏิบัติบ่อย	ปฏิบัติบ่อย
18	ปฏิบัติบ่อย	ปฏิบัติบ่อย	ปฏิบัติบางค	ปฏิบัติประจำ	ปฏิบัติบ่อย	ปฏิบัติบางค	ปฏิบัติประจำ	ปฏิบัติบ่อย
44	ปฏิบัติประจำ	ปฏิบัติประจำ	ไม่ปฏิบัติเล	ปฏิบัติประจำ	ปฏิบัติบางค	ปฏิบัติประจำ	ปฏิบัติบ่อย	ปฏิบัติบ่อย
22	ปฏิบัติประจำ	ปฏิบัติบ่อย	ปฏิบัติประจำ	ปฏิบัติประจำ	ปฏิบัติบ่อย	ปฏิบัติบ่อย	ปฏิบัติบ่อย	ปฏิบัติบ่อย
44	ปฏิบัติประจำ	ปฏิบัติประจำ	ปฏิบัติประจำ	ปฏิบัติประจำ	ปฏิบัติบางค	ปฏิบัติประจำ	ปฏิบัติบ่อย	ปฏิบัติบ่อย
48	ปฏิบัติประจำ	ปฏิบัติบ่อย	ปฏิบัติบ่อย	ปฏิบัติบางค	ปฏิบัติบ่อย	ปฏิบัติประจำ	ปฏิบัติบางค	ปฏิบัติบางค
41	ปฏิบัติประจำ	ปฏิบัติบ่อย	ปฏิบัติบางค	ปฏิบัติบางค	ปฏิบัติบางค	ปฏิบัติบางค	ปฏิบัติประจำ	ปฏิบัติบ่อย
42	ปฏิบัติบางค	ปฏิบัติประจำ	ปฏิบัติบางค	ปฏิบัติบางค	ไม่ปฏิบัติเล	ปฏิบัติบ่อย	ปฏิบัติประจำ	ปฏิบัติประจำ
53	ปฏิบัติบ่อย	ปฏิบัติประจำ	ปฏิบัติประจำ	ปฏิบัติบางค	ไม่ปฏิบัติเล	ปฏิบัติประจำ	ปฏิบัติประจำ	ปฏิบัติบ่อย
48	ปฏิบัติประจำ	ปฏิบัติประจำ	ปฏิบัติประจำ	ปฏิบัติประจำ	ไม่ปฏิบัติเล	ปฏิบัติบางค	ปฏิบัติบางค	ปฏิบัติบางค
36	ปฏิบัติบางค	ปฏิบัติบางค	ปฏิบัติบางค	ปฏิบัติบางค	ปฏิบัติบางค	ปฏิบัติบางค	ปฏิบัติบางค	ปฏิบัติบางค
54	ปฏิบัติประจำ	ปฏิบัติประจำ	ปฏิบัติประจำ	ปฏิบัติบางค	ไม่ปฏิบัติเล	ปฏิบัติบางค	ปฏิบัติประจำ	ปฏิบัติประจำ
39	ปฏิบัติประจำ	ปฏิบัติประจำ	ปฏิบัติประจำ	ปฏิบัติบางค	ไม่ปฏิบัติเล	ปฏิบัติประจำ	ปฏิบัติประจำ	ปฏิบัติประจำ
40	ปฏิบัติบ่อย	ปฏิบัติบ่อย	ปฏิบัติบ่อย	ปฏิบัติบ่อย	ปฏิบัติบ่อย	ปฏิบัติบางค	ปฏิบัติบ่อย	ปฏิบัติบ่อย
53	ปฏิบัติประจำ	ปฏิบัติประจำ	ปฏิบัติประจำ	ปฏิบัติบ่อย	ปฏิบัติบางค	ปฏิบัติบ่อย	ปฏิบัติบางค	ปฏิบัติบางค
50	ปฏิบัติบางค	ปฏิบัติบางค	ปฏิบัติบ่อย	ปฏิบัติประจำ	ปฏิบัติบางค	ปฏิบัติบ่อย	ปฏิบัติบ่อย	ปฏิบัติประจำ
40	ปฏิบัติบ่อย	ปฏิบัติบางค	ปฏิบัติบางค	ไม่ปฏิบัติเล	ไม่ปฏิบัติเล	ปฏิบัติบางค	ปฏิบัติบางค	ปฏิบัติบางค
25	ปฏิบัติประจำ	ปฏิบัติประจำ	ปฏิบัติประจำ	ปฏิบัติประจำ	ไม่ปฏิบัติเล	ปฏิบัติบางค	ปฏิบัติบ่อย	ปฏิบัติบ่อย
45	ปฏิบัติประจำ	ปฏิบัติประจำ	ปฏิบัติประจำ	ปฏิบัติประจำ	ปฏิบัติบางค	ปฏิบัติบางค	ปฏิบัติบ่อย	ปฏิบัติบางค
11	ปฏิบัติประจำ	ปฏิบัติบ่อย	ปฏิบัติประจำ	ปฏิบัติบางค	ไม่ปฏิบัติเล	ไม่ปฏิบัติเล	ปฏิบัติประจำ	ปฏิบัติประจำ
34	ปฏิบัติประจำ	ปฏิบัติบ่อย	ปฏิบัติบางค	ปฏิบัติบ่อย	ไม่ปฏิบัติเล	ปฏิบัติบ่อย	ปฏิบัติบ่อย	ปฏิบัติบ่อย
37	ปฏิบัติประจำ	ปฏิบัติประจำ	ปฏิบัติบางค	ปฏิบัติบางค	ไม่ปฏิบัติเล	ปฏิบัติบางค	ปฏิบัติบ่อย	ปฏิบัติบ่อย
9	ปฏิบัติบางค	ปฏิบัติบ่อย	ปฏิบัติบางค	ไม่ปฏิบัติเล	ปฏิบัติบ่อย	ปฏิบัติบางค	ปฏิบัติประจำ	ปฏิบัติประจำ
25	ปฏิบัติบางค	ปฏิบัติบางค	ปฏิบัติบางค	ปฏิบัติบางค	ปฏิบัติบางค	ปฏิบัติบางค	ปฏิบัติบางค	ปฏิบัติบางค
47	ปฏิบัติบางค	ปฏิบัติบ่อย	ปฏิบัติบ่อย	ปฏิบัติบางค	ปฏิบัติบางค	ปฏิบัติบ่อย	ปฏิบัติประจำ	ปฏิบัติประจำ
30	ปฏิบัติประจำ	ปฏิบัติบางค	ปฏิบัติบางค	ปฏิบัติบางค	ปฏิบัติบางค	ปฏิบัติบางค	ปฏิบัติประจำ	ปฏิบัติประจำ
49	ปฏิบัติประจำ	ปฏิบัติประจำ	ปฏิบัติบ่อย	ปฏิบัติบางค	ปฏิบัติบางค	ไม่ปฏิบัติเล	ปฏิบัติบ่อย	ปฏิบัติบ่อย
50	ปฏิบัติประจำ	ปฏิบัติประจำ	ปฏิบัติประจำ	ปฏิบัติประจำ	ปฏิบัติบางค	ปฏิบัติบ่อย	ปฏิบัติบางค	ปฏิบัติบางค
28	ปฏิบัติประจำ	ปฏิบัติบ่อย	ปฏิบัติประจำ	ปฏิบัติบ่อย	ปฏิบัติบางค	ปฏิบัติบางค	ปฏิบัติบางค	ปฏิบัติบางค
35	ปฏิบัติประจำ	ปฏิบัติประจำ	ปฏิบัติประจำ	ปฏิบัติบางค	ปฏิบัติบางค	ปฏิบัติบางค	ปฏิบัติบ่อย	ปฏิบัติบ่อย

40	ปฏิบัติประจํ	ปฏิบัติประจํ	ปฏิบัติประจํ	ปฏิบัติบางค	ปฏิบัติบางค	ปฏิบัติบ่อย	ปฏิบัติบ่อย	ปฏิบัติบางค
23	ปฏิบัติบางค	ปฏิบัติประจํ	ปฏิบัติประจํ	ปฏิบัติประจํ	ไม่ปฏิบัติเล	ปฏิบัติบ่อย	ปฏิบัติบางค	ปฏิบัติบางค
48	ปฏิบัติประจํ	ปฏิบัติประจํ	ปฏิบัติบ่อย	ปฏิบัติบ่อย	ปฏิบัติบางค	ปฏิบัติบางค	ปฏิบัติบางค	ปฏิบัติบ่อย
41	ปฏิบัติประจํ	ปฏิบัติประจํ	ปฏิบัติประจํ	ปฏิบัติประจํ	ปฏิบัติบ่อย	ปฏิบัติประจํ	ปฏิบัติบ่อย	ปฏิบัติบ่อย
44	ปฏิบัติประจํ	ปฏิบัติประจํ	ปฏิบัติประจํ	ปฏิบัติบางค	ปฏิบัติบางค	ปฏิบัติบางค	ไม่ปฏิบัติเล	ไม่ปฏิบัติเล
37	ปฏิบัติประจํ	ปฏิบัติประจํ	ปฏิบัติประจํ	ไม่ปฏิบัติเล	ไม่ปฏิบัติเล	ไม่ปฏิบัติเล	ปฏิบัติประจํ	ปฏิบัติประจํ
42	ปฏิบัติประจํ	ปฏิบัติบ่อย	ปฏิบัติประจํ	ปฏิบัติประจํ	ไม่ปฏิบัติเล	ปฏิบัติบ่อย	ปฏิบัติบางค	ปฏิบัติบางค
16	ปฏิบัติบางค	ปฏิบัติบ่อย	ไม่ปฏิบัติเล	ปฏิบัติบางค	ปฏิบัติบางค	ปฏิบัติบางค	ปฏิบัติบ่อย	ปฏิบัติประจํ
36	ปฏิบัติประจํ	ปฏิบัติประจํ	ปฏิบัติประจํ	ปฏิบัติประจํ	ปฏิบัติประจํ	ปฏิบัติบางค	ปฏิบัติบางค	ปฏิบัติบ่อย
41	ปฏิบัติประจํ	ปฏิบัติประจํ	ปฏิบัติประจํ	ปฏิบัติบางค	ไม่ปฏิบัติเล	ปฏิบัติบางค	ไม่ปฏิบัติเล	ไม่ปฏิบัติเล
48	ปฏิบัติประจํ	ปฏิบัติประจํ	ปฏิบัติประจํ	ปฏิบัติประจํ	ปฏิบัติประจํ	ปฏิบัติประจํ	ปฏิบัติบางค	ปฏิบัติบางค
29	ปฏิบัติประจํ	ปฏิบัติประจํ	ปฏิบัติบางค	ปฏิบัติประจํ	ไม่ปฏิบัติเล	ปฏิบัติบางค	ปฏิบัติบ่อย	ปฏิบัติบางค
56	ปฏิบัติประจํ	ปฏิบัติประจํ	ปฏิบัติประจํ	ปฏิบัติประจํ	ปฏิบัติบางค	ปฏิบัติประจํ	ปฏิบัติบ่อย	ปฏิบัติบางค
49	ปฏิบัติประจํ	ปฏิบัติบ่อย	ปฏิบัติประจํ	ปฏิบัติประจํ	ปฏิบัติบางค	ปฏิบัติประจํ	ปฏิบัติประจํ	ปฏิบัติประจํ
50	ปฏิบัติประจํ	ปฏิบัติประจํ	ปฏิบัติประจํ	ปฏิบัติประจํ	ไม่ปฏิบัติเล	ปฏิบัติบ่อย	ปฏิบัติบางค	ปฏิบัติบางค
45	ปฏิบัติประจํ	ปฏิบัติบ่อย	ปฏิบัติบ่อย	ปฏิบัติประจํ	ไม่ปฏิบัติเล	ปฏิบัติบ่อย	ปฏิบัติบ่อย	ปฏิบัติบางค
38	ปฏิบัติประจํ	ปฏิบัติบางค	ปฏิบัติประจํ	ปฏิบัติประจํ	ไม่ปฏิบัติเล	ปฏิบัติบางค	ปฏิบัติบางค	ไม่ปฏิบัติเล
30	ปฏิบัติบ่อย	ปฏิบัติบ่อย	ปฏิบัติบ่อย	ปฏิบัติบางค	ปฏิบัติบางค	ปฏิบัติบางค	ปฏิบัติบ่อย	ปฏิบัติบ่อย
28	ปฏิบัติบางค	ปฏิบัติบางค	ปฏิบัติบางค	ปฏิบัติบางค	ปฏิบัติบางค	ปฏิบัติบางค	ปฏิบัติบ่อย	ปฏิบัติบ่อย
34	ปฏิบัติบางค	ปฏิบัติบางค	ปฏิบัติบ่อย	ปฏิบัติบางค	ปฏิบัติบางค	ปฏิบัติบางค	ปฏิบัติบ่อย	ปฏิบัติบ่อย
42	ปฏิบัติประจํ	ปฏิบัติประจํ	ปฏิบัติประจํ	ปฏิบัติประจํ	ปฏิบัติประจํ	ปฏิบัติประจํ	ปฏิบัติประจํ	ปฏิบัติบ่อย
34	ปฏิบัติประจํ	ปฏิบัติประจํ	ปฏิบัติประจํ	ปฏิบัติประจํ	ปฏิบัติบางค	ปฏิบัติประจํ	ปฏิบัติบางค	ปฏิบัติบางค
54	ปฏิบัติประจํ	ปฏิบัติประจํ	ปฏิบัติประจํ	ปฏิบัติประจํ	ปฏิบัติบางค	ปฏิบัติบางค	ปฏิบัติประจํ	ปฏิบัติประจํ
36	ปฏิบัติประจํ	ปฏิบัติประจํ	ปฏิบัติประจํ	ปฏิบัติบางค	ไม่ปฏิบัติเล	ปฏิบัติบางค	ปฏิบัติบ่อย	ปฏิบัติบ่อย
48	ปฏิบัติประจํ	ปฏิบัติประจํ	ปฏิบัติประจํ	ปฏิบัติบ่อย	ปฏิบัติบ่อย	ปฏิบัติประจํ	ปฏิบัติบ่อย	ปฏิบัติประจํ
18	ปฏิบัติประจํ	ปฏิบัติบางค	ปฏิบัติบางค	ปฏิบัติบางค	ปฏิบัติบางค	ปฏิบัติบางค	ปฏิบัติบางค	ปฏิบัติบางค
56	ปฏิบัติประจํ	ปฏิบัติประจํ	ปฏิบัติบางค	ปฏิบัติบางค	ปฏิบัติบางค	ปฏิบัติบางค	ปฏิบัติบางค	ปฏิบัติบางค
42	ปฏิบัติประจํ	ปฏิบัติประจํ	ปฏิบัติประจํ	ปฏิบัติประจํ	ปฏิบัติประจํ	ปฏิบัติบ่อย	ปฏิบัติประจํ	ปฏิบัติบ่อย
56	ปฏิบัติประจํ	ปฏิบัติประจํ	ปฏิบัติประจํ	ปฏิบัติประจํ	ปฏิบัติประจํ	ปฏิบัติประจํ	ปฏิบัติประจํ	ปฏิบัติบางค
56	ปฏิบัติประจํ	ปฏิบัติประจํ	ปฏิบัติประจํ	ปฏิบัติบ่อย	ปฏิบัติบ่อย	ปฏิบัติบ่อย	ปฏิบัติบ่อย	ปฏิบัติบ่อย
44	ปฏิบัติประจํ	ปฏิบัติประจํ	ปฏิบัติบางค	ปฏิบัติบางค	ปฏิบัติบางค	ปฏิบัติบางค	ปฏิบัติบางค	ปฏิบัติบางค
54	ปฏิบัติประจํ	ปฏิบัติประจํ	ปฏิบัติประจํ	ปฏิบัติบางค	ปฏิบัติบางค	ปฏิบัติบางค	ปฏิบัติบางค	ปฏิบัติบางค
56	ปฏิบัติประจํ	ปฏิบัติประจํ	ปฏิบัติประจํ	ปฏิบัติประจํ	ปฏิบัติประจํ	ปฏิบัติประจํ	ปฏิบัติประจํ	ปฏิบัติประจํ
56	ปฏิบัติบ่อย	ปฏิบัติบ่อย	ปฏิบัติบ่อย	ปฏิบัติบางค	ปฏิบัติบางค	ปฏิบัติบางค	ปฏิบัติบ่อย	ปฏิบัติบ่อย
51	ปฏิบัติบ่อย	ปฏิบัติบ่อย	ปฏิบัติบ่อย	ปฏิบัติบางค	ปฏิบัติบ่อย	ปฏิบัติบางค	ปฏิบัติบ่อย	ปฏิบัติบางค
40	ปฏิบัติบ่อย	ปฏิบัติบางค	ปฏิบัติบางค	ปฏิบัติบางค	ปฏิบัติบางค	ปฏิบัติบางค	ปฏิบัติบ่อย	ปฏิบัติบางค
56	ปฏิบัติประจํ	ปฏิบัติประจํ	ปฏิบัติประจํ	ปฏิบัติประจํ	ปฏิบัติบ่อย	ปฏิบัติประจํ	ปฏิบัติบ่อย	ปฏิบัติบ่อย
22	ปฏิบัติประจํ	ปฏิบัติบ่อย	ปฏิบัติบ่อย	ปฏิบัติบางค	ปฏิบัติบางค	ปฏิบัติบางค	ปฏิบัติบางค	ปฏิบัติบางค
42	ปฏิบัติประจํ	ปฏิบัติบ่อย	ปฏิบัติประจํ	ปฏิบัติบางค	ปฏิบัติบางค	ปฏิบัติบางค	ปฏิบัติบ่อย	ปฏิบัติบ่อย
29	ปฏิบัติประจํ	ปฏิบัติบ่อย	ปฏิบัติบ่อย	ปฏิบัติบ่อย	ปฏิบัติบางค	ปฏิบัติบ่อย	ปฏิบัติบ่อย	ปฏิบัติบ่อย
46	ปฏิบัติบางค	ปฏิบัติบ่อย	ปฏิบัติบ่อย	ปฏิบัติบ่อย	ปฏิบัติบางค	ปฏิบัติบ่อย	ปฏิบัติบางค	ปฏิบัติบางค
37	ปฏิบัติประจํ	ปฏิบัติประจํ	ปฏิบัติประจํ	ปฏิบัติประจํ	ไม่ปฏิบัติเล	ปฏิบัติประจํ	ปฏิบัติประจํ	ปฏิบัติบางค
56	ปฏิบัติประจํ	ปฏิบัติประจํ	ปฏิบัติประจํ	ปฏิบัติประจํ	ปฏิบัติประจํ	ปฏิบัติประจํ	ปฏิบัติบ่อย	ปฏิบัติบางค
36	ปฏิบัติประจํ	ปฏิบัติประจํ	ปฏิบัติประจํ	ปฏิบัติประจํ	ไม่ปฏิบัติเล	ปฏิบัติบ่อย	ปฏิบัติประจํ	ปฏิบัติบ่อย
26	ปฏิบัติบางค	ปฏิบัติบางค	ปฏิบัติบ่อย	ปฏิบัติบางค	ไม่ปฏิบัติเล	ปฏิบัติบางค	ปฏิบัติบ่อย	ปฏิบัติบางค
36	ปฏิบัติประจํ	ปฏิบัติประจํ	ปฏิบัติประจํ	ปฏิบัติบ่อย	ปฏิบัติบางค	ปฏิบัติบ่อย	ปฏิบัติบ่อย	ปฏิบัติบางค
56	ปฏิบัติประจํ	ปฏิบัติประจํ	ปฏิบัติบ่อย	ปฏิบัติบางค	ปฏิบัติประจํ	ปฏิบัติประจํ	ปฏิบัติประจํ	ปฏิบัติประจํ
21	ปฏิบัติประจํ	ปฏิบัติประจํ	ปฏิบัติประจํ	ปฏิบัติประจํ	ปฏิบัติบางค	ปฏิบัติประจํ	ปฏิบัติประจํ	ปฏิบัติประจํ
29	ปฏิบัติบางค	ปฏิบัติบางค	ปฏิบัติบางค	ไม่ปฏิบัติเล	ปฏิบัติบางค	ปฏิบัติบางค	ปฏิบัติบางค	ปฏิบัติบางค
38	ปฏิบัติบางค	ปฏิบัติประจํ	ปฏิบัติประจํ	ปฏิบัติประจํ	ปฏิบัติประจํ	ปฏิบัติประจํ	ปฏิบัติบ่อย	ปฏิบัติบ่อย
15	ปฏิบัติประจํ	ปฏิบัติบ่อย	ปฏิบัติประจํ	ปฏิบัติประจํ	ปฏิบัติบางค	ปฏิบัติบ่อย	ปฏิบัติประจํ	ปฏิบัติประจํ

39	ปฏิบัติประจำ	ปฏิบัติประจำ	ปฏิบัติประจำ	ปฏิบัติประจำ	ปฏิบัติบางค	ปฏิบัติประจำ	ปฏิบัติประจำ	ปฏิบัติประจำ
42	ปฏิบัติบ่อย	ปฏิบัติบ่อย	ปฏิบัติประจำ	ปฏิบัติประจำ	ปฏิบัติบางค	ปฏิบัติบ่อย	ปฏิบัติประจำ	ปฏิบัติบ่อย
35	ปฏิบัติบ่อย	ปฏิบัติบางค	ไม่ปฏิบัติเล	ปฏิบัติบางค	ไม่ปฏิบัติเล	ปฏิบัติบางค	ปฏิบัติบ่อย	ปฏิบัติบ่อย
42	ปฏิบัติบ่อย	ปฏิบัติบ่อย	ปฏิบัติบ่อย	ปฏิบัติบางค	ไม่ปฏิบัติเล	ปฏิบัติบางค	ปฏิบัติบ่อย	ปฏิบัติบางค
38	ปฏิบัติประจำ	ปฏิบัติบ่อย	ปฏิบัติประจำ	ปฏิบัติบ่อย	ปฏิบัติบ่อย	ปฏิบัติบ่อย	ปฏิบัติบ่อย	ปฏิบัติบ่อย
36	ปฏิบัติบ่อย	ปฏิบัติบ่อย	ปฏิบัติบ่อย	ปฏิบัติบางค	ปฏิบัติบางค	ปฏิบัติบางค	ปฏิบัติบางค	ปฏิบัติบ่อย
38	ปฏิบัติประจำ	ปฏิบัติประจำ	ปฏิบัติประจำ	ปฏิบัติประจำ	ไม่ปฏิบัติเล	ปฏิบัติบางค	ปฏิบัติประจำ	ปฏิบัติประจำ
47	ปฏิบัติบางค	ปฏิบัติบ่อย	ปฏิบัติประจำ	ปฏิบัติบางค	ปฏิบัติบางค	ปฏิบัติบางค	ปฏิบัติประจำ	ปฏิบัติประจำ
52	ปฏิบัติประจำ	ปฏิบัติประจำ	ปฏิบัติบ่อย	ปฏิบัติบ่อย	ปฏิบัติประจำ	ปฏิบัติบ่อย	ปฏิบัติประจำ	ปฏิบัติประจำ
42	ปฏิบัติประจำ	ปฏิบัติประจำ	ปฏิบัติประจำ	ปฏิบัติบ่อย	ปฏิบัติบ่อย	ปฏิบัติบางค	ปฏิบัติประจำ	ปฏิบัติบ่อย
29	ปฏิบัติประจำ	ปฏิบัติประจำ	ปฏิบัติประจำ	ปฏิบัติประจำ	ปฏิบัติบางค	ปฏิบัติบางค	ปฏิบัติบ่อย	ปฏิบัติบ่อย
45	ปฏิบัติประจำ	ปฏิบัติประจำ	ปฏิบัติประจำ	ปฏิบัติบางค	ไม่ปฏิบัติเล	ปฏิบัติบ่อย	ปฏิบัติประจำ	ปฏิบัติบ่อย
40	ปฏิบัติบ่อย	ปฏิบัติบ่อย	ปฏิบัติประจำ	ปฏิบัติประจำ	ปฏิบัติประจำ	ปฏิบัติบางค	ปฏิบัติบางค	ไม่ปฏิบัติเล
38	ปฏิบัติประจำ	ปฏิบัติบ่อย	ปฏิบัติบ่อย	ปฏิบัติบางค	ไม่ปฏิบัติเล	ปฏิบัติบางค	ปฏิบัติบ่อย	ปฏิบัติบางค
43	ปฏิบัติประจำ	ปฏิบัติประจำ	ปฏิบัติประจำ	ปฏิบัติบางค	ไม่ปฏิบัติเล	ปฏิบัติบางค	ปฏิบัติประจำ	ปฏิบัติประจำ
34	ปฏิบัติบ่อย	ปฏิบัติบ่อย	ปฏิบัติบางค	ปฏิบัติบางค	ไม่ปฏิบัติเล	ปฏิบัติประจำ	ปฏิบัติบ่อย	ไม่ปฏิบัติเล
42	ปฏิบัติประจำ	ปฏิบัติประจำ	ปฏิบัติประจำ	ปฏิบัติประจำ	ปฏิบัติบ่อย	ปฏิบัติประจำ	ปฏิบัติบ่อย	ปฏิบัติบ่อย
28	ปฏิบัติประจำ	ปฏิบัติประจำ	ปฏิบัติประจำ	ปฏิบัติบางค	ไม่ปฏิบัติเล	ปฏิบัติบางค	ปฏิบัติประจำ	ปฏิบัติบางค
19	ปฏิบัติบางค	ปฏิบัติบ่อย	ปฏิบัติบ่อย	ปฏิบัติบางค	ปฏิบัติบางค	ปฏิบัติบางค	ปฏิบัติบ่อย	ปฏิบัติบ่อย
56	ปฏิบัติประจำ	ปฏิบัติประจำ	ปฏิบัติประจำ	ปฏิบัติประจำ	ปฏิบัติบ่อย	ปฏิบัติบางค	ปฏิบัติประจำ	ปฏิบัติบ่อย
55	ปฏิบัติประจำ	ปฏิบัติประจำ	ปฏิบัติประจำ	ปฏิบัติบ่อย	ปฏิบัติบางค	ปฏิบัติบ่อย	ปฏิบัติประจำ	ปฏิบัติประจำ
40	ปฏิบัติบางค	ปฏิบัติบ่อย	ปฏิบัติบางค	ไม่ปฏิบัติเล	ไม่ปฏิบัติเล	ปฏิบัติบางค	ปฏิบัติประจำ	ปฏิบัติบ่อย
42	ปฏิบัติประจำ	ปฏิบัติประจำ	ปฏิบัติบ่อย	ปฏิบัติประจำ	ปฏิบัติบางค	ปฏิบัติบ่อย	ปฏิบัติบางค	ปฏิบัติบางค
26	ปฏิบัติบ่อย	ปฏิบัติบ่อย	ปฏิบัติบ่อย	ปฏิบัติประจำ	ปฏิบัติบางค	ปฏิบัติประจำ	ปฏิบัติประจำ	ปฏิบัติบ่อย
38	ปฏิบัติประจำ	ปฏิบัติประจำ	ไม่ปฏิบัติเล	ปฏิบัติประจำ	ปฏิบัติบ่อย	ปฏิบัติบ่อย	ปฏิบัติบ่อย	ปฏิบัติบางค
41	ปฏิบัติประจำ	ปฏิบัติประจำ	ปฏิบัติประจำ	ปฏิบัติบางค	ปฏิบัติบางค	ปฏิบัติบ่อย	ปฏิบัติบ่อย	ปฏิบัติบางค
39	ปฏิบัติประจำ	ปฏิบัติบ่อย	ปฏิบัติประจำ	ปฏิบัติประจำ	ปฏิบัติบางค	ปฏิบัติประจำ	ไม่ปฏิบัติเล	ไม่ปฏิบัติเล
29	ปฏิบัติประจำ	ปฏิบัติบ่อย	ปฏิบัติประจำ	ปฏิบัติประจำ	ปฏิบัติประจำ	ปฏิบัติประจำ	ปฏิบัติบางค	ปฏิบัติบางค
36	ปฏิบัติประจำ	ปฏิบัติประจำ	ปฏิบัติบ่อย	ปฏิบัติบ่อย	ปฏิบัติบางค	ปฏิบัติประจำ	ปฏิบัติประจำ	ปฏิบัติประจำ
41	ปฏิบัติบางค	ปฏิบัติบางค	ปฏิบัติบางค	ปฏิบัติบางค	ปฏิบัติบางค	ปฏิบัติบางค	ปฏิบัติบางค	ปฏิบัติบางค
44	ปฏิบัติประจำ	ปฏิบัติบางค	ปฏิบัติประจำ	ปฏิบัติบางค	ไม่ปฏิบัติเล	ปฏิบัติบางค	ปฏิบัติบางค	ปฏิบัติบางค
50	ปฏิบัติบ่อย	ปฏิบัติบางค	ปฏิบัติบางค	ไม่ปฏิบัติเล	ไม่ปฏิบัติเล	ปฏิบัติบ่อย	ปฏิบัติบ่อย	ไม่ปฏิบัติเล
43	ปฏิบัติประจำ	ปฏิบัติประจำ	ปฏิบัติประจำ	ปฏิบัติประจำ	ไม่ปฏิบัติเล	ปฏิบัติบางค	ปฏิบัติบางค	ปฏิบัติบางค
41	ปฏิบัติประจำ	ปฏิบัติประจำ	ปฏิบัติประจำ	ปฏิบัติประจำ	ไม่ปฏิบัติเล	ปฏิบัติประจำ	ปฏิบัติบ่อย	ปฏิบัติบางค
39	ปฏิบัติบางค	ปฏิบัติบางค	ปฏิบัติบ่อย	ปฏิบัติบางค	ไม่ปฏิบัติเล	ปฏิบัติบางค	ปฏิบัติบางค	ปฏิบัติบางค
45	ปฏิบัติประจำ	ปฏิบัติบ่อย	ปฏิบัติประจำ	ปฏิบัติบางค	ไม่ปฏิบัติเล	ปฏิบัติบางค	ปฏิบัติบ่อย	ปฏิบัติบ่อย
41	ปฏิบัติประจำ	ปฏิบัติประจำ	ปฏิบัติประจำ	ปฏิบัติประจำ	ปฏิบัติบ่อย	ปฏิบัติประจำ	ปฏิบัติบางค	ปฏิบัติบ่อย
18	ปฏิบัติประจำ	ปฏิบัติบ่อย	ปฏิบัติประจำ	ปฏิบัติประจำ	ปฏิบัติประจำ	ปฏิบัติบางค	ปฏิบัติบ่อย	ปฏิบัติบางค
34	ปฏิบัติประจำ	ปฏิบัติประจำ	ปฏิบัติบางค	ปฏิบัติบางค	ปฏิบัติบางค	ปฏิบัติบางค	ปฏิบัติบ่อย	ปฏิบัติบ่อย
39	ปฏิบัติประจำ	ปฏิบัติประจำ	ปฏิบัติบางค	ปฏิบัติประจำ	ปฏิบัติบางค	ปฏิบัติบ่อย	ปฏิบัติบ่อย	ปฏิบัติบ่อย
50	ปฏิบัติบ่อย	ปฏิบัติประจำ	ไม่ปฏิบัติเล	ปฏิบัติประจำ	ปฏิบัติบางค	ปฏิบัติบ่อย	ปฏิบัติบ่อย	ปฏิบัติประจำ
38	ปฏิบัติประจำ	ปฏิบัติประจำ	ปฏิบัติประจำ	ปฏิบัติประจำ	ปฏิบัติบ่อย	ปฏิบัติบ่อย	ปฏิบัติประจำ	ปฏิบัติประจำ
37	ปฏิบัติประจำ	ปฏิบัติประจำ	ปฏิบัติบ่อย	ปฏิบัติบ่อย	ปฏิบัติประจำ	ปฏิบัติบ่อย	ปฏิบัติประจำ	ปฏิบัติประจำ
28	ปฏิบัติประจำ	ปฏิบัติบ่อย	ปฏิบัติบ่อย	ปฏิบัติบางค	ไม่ปฏิบัติเล	ปฏิบัติบางค	ปฏิบัติบางค	ปฏิบัติบางค
44	ปฏิบัติประจำ	ปฏิบัติประจำ	ปฏิบัติประจำ	ปฏิบัติประจำ	ปฏิบัติบางค	ปฏิบัติบ่อย	ปฏิบัติประจำ	ปฏิบัติประจำ
25	ปฏิบัติประจำ	ปฏิบัติบางค	ปฏิบัติบางค	ปฏิบัติบางค	ไม่ปฏิบัติเล	ปฏิบัติประจำ	ปฏิบัติบ่อย	ปฏิบัติบ่อย
43	ปฏิบัติบ่อย	ปฏิบัติประจำ	ปฏิบัติบ่อย	ปฏิบัติประจำ	ปฏิบัติบางค	ปฏิบัติบ่อย	ปฏิบัติบางค	ปฏิบัติบางค
43	ปฏิบัติประจำ	ปฏิบัติประจำ	ปฏิบัติบ่อย	ปฏิบัติประจำ	ปฏิบัติบางค	ปฏิบัติประจำ	ปฏิบัติบางค	ปฏิบัติบางค
20	ปฏิบัติประจำ	ปฏิบัติประจำ	ปฏิบัติประจำ	ปฏิบัติบางค	ปฏิบัติประจำ	ปฏิบัติประจำ	ปฏิบัติบ่อย	ปฏิบัติประจำ
52	ปฏิบัติประจำ	ปฏิบัติประจำ	ปฏิบัติประจำ	ปฏิบัติบางค	ปฏิบัติบางค	ปฏิบัติบางค	ปฏิบัติบ่อย	ปฏิบัติบ่อย
27	ปฏิบัติประจำ	ปฏิบัติประจำ	ปฏิบัติประจำ	ปฏิบัติบางค	ไม่ปฏิบัติเล	ปฏิบัติบางค	ปฏิบัติบางค	ไม่ปฏิบัติเล

38	ปฏิบัติประจำ	ปฏิบัติบางค	ปฏิบัติประจำ	ปฏิบัติประจำ	ไม่ปฏิบัติเล	ไม่ปฏิบัติเล	ปฏิบัติประจำ	ปฏิบัติบางค
43	ปฏิบัติประจำ	ปฏิบัติบางค	ปฏิบัติบ่อย	ปฏิบัติประจำ	ปฏิบัติบางค	ปฏิบัติบ่อย	ปฏิบัติบ่อย	ปฏิบัติบ่อย
46	ปฏิบัติประจำ	ปฏิบัติประจำ	ปฏิบัติบางค	ปฏิบัติประจำ	ไม่ปฏิบัติเล	ปฏิบัติบ่อย	ปฏิบัติบางค	ไม่ปฏิบัติเล
54	ปฏิบัติประจำ	ปฏิบัติประจำ	ปฏิบัติบ่อย	ปฏิบัติบ่อย	ปฏิบัติบ่อย	ปฏิบัติบ่อย	ปฏิบัติบ่อย	ปฏิบัติบ่อย
56	ปฏิบัติประจำ	ปฏิบัติประจำ	ปฏิบัติประจำ	ปฏิบัติประจำ	ปฏิบัติบางค	ปฏิบัติประจำ	ปฏิบัติบางค	ปฏิบัติประจำ
16	ปฏิบัติประจำ	ปฏิบัติประจำ	ปฏิบัติประจำ	ปฏิบัติบางค	ไม่ปฏิบัติเล	ปฏิบัติประจำ	ปฏิบัติประจำ	ปฏิบัติบางค
56	ปฏิบัติบ่อย	ปฏิบัติบ่อย	ปฏิบัติบ่อย	ปฏิบัติบ่อย	ปฏิบัติบ่อย	ปฏิบัติบ่อย	ปฏิบัติบ่อย	ปฏิบัติบ่อย
45	ปฏิบัติบางค	ปฏิบัติบางค	ปฏิบัติบางค	ไม่ปฏิบัติเล	ไม่ปฏิบัติเล	ไม่ปฏิบัติเล	ปฏิบัติบ่อย	ปฏิบัติบางค
37	ปฏิบัติบ่อย	ปฏิบัติประจำ	ปฏิบัติประจำ	ปฏิบัติบางค	ไม่ปฏิบัติเล	ไม่ปฏิบัติเล	ปฏิบัติประจำ	ไม่ปฏิบัติเล
41	ปฏิบัติประจำ	ปฏิบัติประจำ	ปฏิบัติประจำ	ปฏิบัติประจำ	ปฏิบัติบ่อย	ปฏิบัติบางค	ปฏิบัติบางค	ปฏิบัติบางค
49	ปฏิบัติประจำ	ปฏิบัติประจำ	ปฏิบัติประจำ	ปฏิบัติประจำ	ปฏิบัติประจำ	ปฏิบัติบ่อย	ปฏิบัติบ่อย	ปฏิบัติบางค
20	ปฏิบัติประจำ	ปฏิบัติประจำ	ปฏิบัติบางค	ปฏิบัติบ่อย	ไม่ปฏิบัติเล	ปฏิบัติประจำ	ปฏิบัติบางค	ปฏิบัติบางค
42	ปฏิบัติประจำ	ปฏิบัติประจำ	ปฏิบัติประจำ	ปฏิบัติประจำ	ปฏิบัติบางค	ปฏิบัติประจำ	ปฏิบัติประจำ	ปฏิบัติบางค
38	ปฏิบัติประจำ	ปฏิบัติบ่อย	ปฏิบัติประจำ	ปฏิบัติบ่อย	ไม่ปฏิบัติเล	ปฏิบัติบ่อย	ปฏิบัติบ่อย	ปฏิบัติบางค
20	ปฏิบัติประจำ	ปฏิบัติประจำ	ปฏิบัติประจำ	ปฏิบัติบางค	ไม่ปฏิบัติเล	ปฏิบัติประจำ	ปฏิบัติบ่อย	ไม่ปฏิบัติเล
46	ปฏิบัติบางค	ปฏิบัติบางค	ปฏิบัติบางค	ปฏิบัติบางค	ปฏิบัติบางค	ปฏิบัติบางค	ปฏิบัติบางค	ปฏิบัติบางค
48	ปฏิบัติประจำ	ปฏิบัติประจำ	ปฏิบัติประจำ	ปฏิบัติประจำ	ไม่ปฏิบัติเล	ปฏิบัติบ่อย	ปฏิบัติบางค	ปฏิบัติบางค
33	ปฏิบัติประจำ	ปฏิบัติบ่อย	ปฏิบัติบ่อย	ปฏิบัติบ่อย	ไม่ปฏิบัติเล	ปฏิบัติบางค	ปฏิบัติบางค	ปฏิบัติบางค
43	ปฏิบัติประจำ	ปฏิบัติบ่อย	ปฏิบัติประจำ	ปฏิบัติบางค	ปฏิบัติบางค	ปฏิบัติบ่อย	ปฏิบัติบ่อย	ปฏิบัติบางค
44	ปฏิบัติบ่อย	ปฏิบัติบางค	ปฏิบัติบ่อย	ปฏิบัติบางค	ไม่ปฏิบัติเล	ปฏิบัติบางค	ปฏิบัติบางค	ปฏิบัติบางค
49	ปฏิบัติประจำ	ปฏิบัติบ่อย	ปฏิบัติประจำ	ปฏิบัติบ่อย	ปฏิบัติบางค	ปฏิบัติบ่อย	ปฏิบัติบ่อย	ปฏิบัติบ่อย
47	ปฏิบัติประจำ	ปฏิบัติบ่อย	ปฏิบัติประจำ	ปฏิบัติบางค	ไม่ปฏิบัติเล	ปฏิบัติประจำ	ปฏิบัติบางค	ปฏิบัติบางค
35	ปฏิบัติบ่อย	ปฏิบัติบ่อย	ปฏิบัติบางค	ปฏิบัติบางค	ไม่ปฏิบัติเล	ปฏิบัติบ่อย	ปฏิบัติบ่อย	ปฏิบัติบางค
44	ปฏิบัติประจำ	ปฏิบัติประจำ	ปฏิบัติประจำ	ปฏิบัติประจำ	ปฏิบัติบางค	ปฏิบัติบ่อย	ปฏิบัติประจำ	ปฏิบัติบ่อย
44	ปฏิบัติประจำ	ปฏิบัติประจำ	ปฏิบัติประจำ	ปฏิบัติประจำ	ปฏิบัติบ่อย	ปฏิบัติบางค	ปฏิบัติบางค	ปฏิบัติประจำ
25	ปฏิบัติบ่อย	ปฏิบัติบางค	ปฏิบัติบ่อย	ปฏิบัติบางค	ไม่ปฏิบัติเล	ไม่ปฏิบัติเล	ปฏิบัติบ่อย	ปฏิบัติบางค
49	ปฏิบัติบ่อย	ปฏิบัติประจำ	ปฏิบัติประจำ	ปฏิบัติบางค	ปฏิบัติบางค	ปฏิบัติบางค	ปฏิบัติบ่อย	ปฏิบัติบ่อย
29	ปฏิบัติบ่อย	ปฏิบัติบางค	ปฏิบัติบ่อย	ไม่ปฏิบัติเล	ไม่ปฏิบัติเล	ไม่ปฏิบัติเล	ปฏิบัติบ่อย	ปฏิบัติบางค
0	ปฏิบัติประจำ	ปฏิบัติประจำ	ปฏิบัติประจำ	ปฏิบัติประจำ	ปฏิบัติบางค	ปฏิบัติบางค	ปฏิบัติบางค	ปฏิบัติบางค
45	ปฏิบัติประจำ	ปฏิบัติบ่อย	ปฏิบัติประจำ	ปฏิบัติประจำ	ปฏิบัติบางค	ปฏิบัติบ่อย	ปฏิบัติบางค	ปฏิบัติบางค
42	ปฏิบัติบ่อย	ปฏิบัติประจำ	ปฏิบัติประจำ	ปฏิบัติบ่อย	ไม่ปฏิบัติเล	ปฏิบัติบ่อย	ปฏิบัติบ่อย	ปฏิบัติบางค
34	ปฏิบัติประจำ	ปฏิบัติบ่อย	ปฏิบัติประจำ	ปฏิบัติประจำ	ปฏิบัติบ่อย	ปฏิบัติบ่อย	ปฏิบัติบ่อย	ปฏิบัติบ่อย
45	ปฏิบัติประจำ	ปฏิบัติบ่อย	ปฏิบัติประจำ	ปฏิบัติบางค	ปฏิบัติบางค	ปฏิบัติบ่อย	ปฏิบัติบ่อย	ปฏิบัติบ่อย
21	ปฏิบัติประจำ	ปฏิบัติบางค	ปฏิบัติบางค	ปฏิบัติบางค	ปฏิบัติบางค	ปฏิบัติประจำ	ปฏิบัติบางค	ปฏิบัติบางค
50	ปฏิบัติประจำ	ปฏิบัติประจำ	ปฏิบัติบางค	ปฏิบัติประจำ	ปฏิบัติบ่อย	ปฏิบัติประจำ	ปฏิบัติบางค	ปฏิบัติบางค
52	ปฏิบัติประจำ	ปฏิบัติประจำ	ปฏิบัติประจำ	ปฏิบัติประจำ	ปฏิบัติบ่อย	ปฏิบัติบ่อย	ปฏิบัติประจำ	ปฏิบัติประจำ
29	ปฏิบัติบ่อย	ปฏิบัติบางค	ปฏิบัติบ่อย	ไม่ปฏิบัติเล	ไม่ปฏิบัติเล	ไม่ปฏิบัติเล	ปฏิบัติบ่อย	ปฏิบัติบางค
45	ปฏิบัติประจำ	ปฏิบัติบ่อย	ปฏิบัติประจำ	ปฏิบัติบางค	ไม่ปฏิบัติเล	ปฏิบัติบางค	ปฏิบัติประจำ	ปฏิบัติประจำ
54	ปฏิบัติประจำ	ปฏิบัติประจำ	ปฏิบัติประจำ	ปฏิบัติประจำ	ปฏิบัติบางค	ปฏิบัติบ่อย	ปฏิบัติบางค	ปฏิบัติบ่อย
34	ปฏิบัติบ่อย	ปฏิบัติบ่อย	ปฏิบัติบ่อย	ปฏิบัติบางค	ปฏิบัติบางค	ปฏิบัติบ่อย	ปฏิบัติบ่อย	ปฏิบัติบ่อย
32	ปฏิบัติบ่อย	ปฏิบัติบ่อย	ปฏิบัติบ่อย	ปฏิบัติบ่อย	ปฏิบัติบางค	ปฏิบัติบางค	ปฏิบัติบ่อย	ปฏิบัติบ่อย
25	ปฏิบัติประจำ	ปฏิบัติประจำ	ปฏิบัติประจำ	ปฏิบัติประจำ	ปฏิบัติบางค	ปฏิบัติประจำ	ปฏิบัติบางค	ปฏิบัติบางค
52	ปฏิบัติประจำ	ปฏิบัติประจำ	ปฏิบัติประจำ	ปฏิบัติประจำ	ปฏิบัติบ่อย	ปฏิบัติประจำ	ปฏิบัติบ่อย	ปฏิบัติบ่อย
43	ปฏิบัติประจำ	ปฏิบัติประจำ	ปฏิบัติประจำ	ปฏิบัติประจำ	ปฏิบัติบางค	ปฏิบัติประจำ	ปฏิบัติบางค	ปฏิบัติบางค
17	ปฏิบัติบ่อย	ปฏิบัติบ่อย	ปฏิบัติประจำ	ปฏิบัติประจำ	ปฏิบัติประจำ	ปฏิบัติบางค	ปฏิบัติบ่อย	ปฏิบัติบางค
51	ปฏิบัติบ่อย	ปฏิบัติบ่อย	ปฏิบัติประจำ	ปฏิบัติบ่อย	ปฏิบัติบางค	ปฏิบัติประจำ	ปฏิบัติบ่อย	ปฏิบัติบ่อย
39	ปฏิบัติบ่อย	ปฏิบัติบ่อย	ปฏิบัติบ่อย	ปฏิบัติบ่อย	ไม่ปฏิบัติเล	ไม่ปฏิบัติเล	ปฏิบัติบ่อย	ปฏิบัติบางค
37	ปฏิบัติประจำ	ปฏิบัติประจำ	ปฏิบัติประจำ	ปฏิบัติประจำ	ปฏิบัติบางค	ปฏิบัติบ่อย	ปฏิบัติบางค	ปฏิบัติบางค
49	ปฏิบัติประจำ	ปฏิบัติบ่อย	ปฏิบัติประจำ	ปฏิบัติบ่อย	ไม่ปฏิบัติเล	ปฏิบัติบางค	ปฏิบัติบ่อย	ปฏิบัติประจำ
39	ปฏิบัติบ่อย	ปฏิบัติบ่อย	ปฏิบัติบ่อย	ปฏิบัติประจำ	ปฏิบัติบางค	ปฏิบัติประจำ	ปฏิบัติประจำ	ปฏิบัติประจำ
53	ปฏิบัติประจำ	ปฏิบัติบางค	ปฏิบัติประจำ	ปฏิบัติประจำ	ไม่ปฏิบัติเล	ปฏิบัติประจำ	ปฏิบัติประจำ	ปฏิบัติประจำ

39	ปฏิบัติประจำ	ปฏิบัติประจำ	ปฏิบัติประจำ	ปฏิบัติประจำ	ปฏิบัติบาง	ปฏิบัติประจำ	ปฏิบัติบาง	ปฏิบัติบาง
47	ปฏิบัติประจำ	ปฏิบัติประจำ	ปฏิบัติประจำ	ปฏิบัติประจำ	ปฏิบัติบ่อย	ปฏิบัติประจำ	ปฏิบัติบาง	ปฏิบัติบาง
55	ปฏิบัติประจำ	ปฏิบัติประจำ	ปฏิบัติประจำ	ปฏิบัติบ่อย	ไม่ปฏิบัติเล	ปฏิบัติประจำ	ปฏิบัติบ่อย	ปฏิบัติบาง
46	ปฏิบัติประจำ	ปฏิบัติบาง	ปฏิบัติประจำ	ปฏิบัติบาง	ปฏิบัติบาง	ปฏิบัติบาง	ปฏิบัติบาง	ปฏิบัติบาง
25	ปฏิบัติบ่อย	ปฏิบัติบาง	ปฏิบัติประจำ	ปฏิบัติบ่อย	ไม่ปฏิบัติเล	ปฏิบัติบ่อย	ปฏิบัติประจำ	ปฏิบัติประจำ
44	ปฏิบัติประจำ	ปฏิบัติประจำ	ปฏิบัติประจำ	ปฏิบัติบ่อย	ปฏิบัติบาง	ปฏิบัติบ่อย	ปฏิบัติบาง	ปฏิบัติบาง
42	ปฏิบัติบ่อย	ปฏิบัติบ่อย	ปฏิบัติบ่อย	ปฏิบัติบ่อย	ปฏิบัติบ่อย	ปฏิบัติบ่อย	ปฏิบัติบ่อย	ปฏิบัติบาง
40	ปฏิบัติประจำ	ปฏิบัติบ่อย	ปฏิบัติประจำ	ปฏิบัติบาง	ปฏิบัติบาง	ไม่ปฏิบัติเล	ปฏิบัติบ่อย	ปฏิบัติบ่อย
28	ปฏิบัติบาง	ปฏิบัติบาง	ปฏิบัติบาง	ปฏิบัติบาง	ปฏิบัติบาง	ปฏิบัติบาง	ปฏิบัติบ่อย	ปฏิบัติบาง
41	ปฏิบัติประจำ	ปฏิบัติบาง	ปฏิบัติบ่อย	ปฏิบัติบาง	ไม่ปฏิบัติเล	ปฏิบัติประจำ	ปฏิบัติบ่อย	ปฏิบัติบ่อย
49	ปฏิบัติบ่อย	ปฏิบัติบ่อย	ปฏิบัติบ่อย	ปฏิบัติบ่อย	ปฏิบัติบ่อย	ปฏิบัติบ่อย	ปฏิบัติบ่อย	ปฏิบัติบ่อย
39	ปฏิบัติบ่อย	ปฏิบัติบ่อย	ปฏิบัติประจำ	ปฏิบัติประจำ	ปฏิบัติประจำ	ปฏิบัติบ่อย	ปฏิบัติประจำ	ปฏิบัติประจำ
37	ปฏิบัติบ่อย	ปฏิบัติบ่อย	ปฏิบัติบ่อย	ปฏิบัติบ่อย	ปฏิบัติประจำ	ปฏิบัติประจำ	ปฏิบัติประจำ	ปฏิบัติบ่อย
41	ปฏิบัติบ่อย	ปฏิบัติบ่อย	ปฏิบัติบ่อย	ปฏิบัติบ่อย	ปฏิบัติบาง	ปฏิบัติบาง	ปฏิบัติบ่อย	ปฏิบัติบ่อย
37	ปฏิบัติบ่อย	ปฏิบัติบ่อย	ปฏิบัติบ่อย	ปฏิบัติบ่อย	ปฏิบัติบาง	ปฏิบัติบ่อย	ปฏิบัติบ่อย	ปฏิบัติประจำ
40	ปฏิบัติบ่อย	ปฏิบัติบ่อย	ปฏิบัติบ่อย	ปฏิบัติบาง	ปฏิบัติบ่อย	ปฏิบัติบาง	ปฏิบัติบ่อย	ปฏิบัติบ่อย
36	ปฏิบัติบ่อย	ปฏิบัติบ่อย	ปฏิบัติบ่อย	ปฏิบัติบ่อย	ปฏิบัติบาง	ปฏิบัติบาง	ปฏิบัติบาง	ปฏิบัติบาง
38	ปฏิบัติบ่อย	ปฏิบัติบ่อย	ปฏิบัติบาง	ปฏิบัติบ่อย	ปฏิบัติบ่อย	ปฏิบัติบ่อย	ปฏิบัติบ่อย	ปฏิบัติบ่อย
37	ปฏิบัติประจำ	ปฏิบัติบ่อย	ปฏิบัติประจำ	ปฏิบัติบ่อย	ปฏิบัติบ่อย	ปฏิบัติประจำ	ปฏิบัติประจำ	ปฏิบัติประจำ
45	ปฏิบัติประจำ	ปฏิบัติประจำ	ปฏิบัติประจำ	ปฏิบัติบาง	ไม่ปฏิบัติเล	ปฏิบัติบาง	ปฏิบัติบ่อย	ปฏิบัติบาง
46	ปฏิบัติประจำ	ปฏิบัติประจำ	ปฏิบัติประจำ	ปฏิบัติบ่อย	ไม่ปฏิบัติเล	ปฏิบัติประจำ	ปฏิบัติบาง	ปฏิบัติบาง
34	ปฏิบัติประจำ	ปฏิบัติประจำ	ปฏิบัติประจำ	ปฏิบัติประจำ	ปฏิบัติบาง	ปฏิบัติบ่อย	ปฏิบัติบ่อย	ปฏิบัติบาง
32	ปฏิบัติบ่อย	ปฏิบัติบ่อย	ปฏิบัติบ่อย	ไม่ปฏิบัติเล	ไม่ปฏิบัติเล	ไม่ปฏิบัติเล	ปฏิบัติบ่อย	ปฏิบัติบาง
48	ปฏิบัติประจำ	ปฏิบัติประจำ	ปฏิบัติประจำ	ปฏิบัติประจำ	ปฏิบัติประจำ	ปฏิบัติประจำ	ปฏิบัติบาง	ปฏิบัติบาง
32	ปฏิบัติบาง	ปฏิบัติบาง	ปฏิบัติประจำ	ไม่ปฏิบัติเล	ไม่ปฏิบัติเล	ปฏิบัติบาง	ไม่ปฏิบัติเล	ปฏิบัติบาง
25	ปฏิบัติบ่อย	ปฏิบัติบ่อย	ปฏิบัติบ่อย	ปฏิบัติบ่อย	ปฏิบัติบาง	ปฏิบัติบาง	ปฏิบัติบ่อย	ปฏิบัติบาง
41	ปฏิบัติประจำ	ปฏิบัติประจำ	ปฏิบัติประจำ	ปฏิบัติประจำ	ปฏิบัติประจำ	ปฏิบัติประจำ	ปฏิบัติประจำ	ปฏิบัติประจำ
39	ปฏิบัติประจำ	ปฏิบัติประจำ	ปฏิบัติประจำ	ปฏิบัติบ่อย	ปฏิบัติบาง	ปฏิบัติบาง	ปฏิบัติบ่อย	ปฏิบัติบ่อย
39	ปฏิบัติประจำ	ปฏิบัติประจำ	ปฏิบัติบ่อย	ปฏิบัติบ่อย	ปฏิบัติประจำ	ปฏิบัติบ่อย	ปฏิบัติประจำ	ปฏิบัติบ่อย
39	ปฏิบัติประจำ	ปฏิบัติบ่อย	ปฏิบัติประจำ	ปฏิบัติประจำ	ปฏิบัติประจำ	ปฏิบัติประจำ	ปฏิบัติประจำ	ปฏิบัติประจำ
36	ปฏิบัติประจำ	ปฏิบัติประจำ	ปฏิบัติบ่อย	ปฏิบัติประจำ	ปฏิบัติประจำ	ปฏิบัติประจำ	ปฏิบัติบ่อย	ปฏิบัติบ่อย
54	ปฏิบัติประจำ	ปฏิบัติประจำ	ปฏิบัติบ่อย	ปฏิบัติประจำ	ปฏิบัติบ่อย	ปฏิบัติบ่อย	ปฏิบัติบาง	ปฏิบัติบาง
44	ปฏิบัติประจำ	ปฏิบัติประจำ	ปฏิบัติบ่อย	ปฏิบัติบาง	ปฏิบัติประจำ	ปฏิบัติประจำ	ปฏิบัติประจำ	ปฏิบัติประจำ
43	ปฏิบัติประจำ	ปฏิบัติประจำ	ปฏิบัติประจำ	ปฏิบัติประจำ	ปฏิบัติประจำ	ปฏิบัติประจำ	ปฏิบัติบาง	ปฏิบัติบาง
48	ปฏิบัติประจำ	ปฏิบัติประจำ	ปฏิบัติประจำ	ปฏิบัติบ่อย	ปฏิบัติประจำ	ปฏิบัติบ่อย	ปฏิบัติประจำ	ปฏิบัติประจำ
39	ปฏิบัติประจำ	ปฏิบัติบ่อย	ปฏิบัติบ่อย	ปฏิบัติบาง	ปฏิบัติบาง	ปฏิบัติบาง	ปฏิบัติบาง	ปฏิบัติบาง
47	ปฏิบัติบ่อย	ปฏิบัติบ่อย	ปฏิบัติบ่อย	ปฏิบัติบ่อย	ปฏิบัติบาง	ปฏิบัติบาง	ปฏิบัติบ่อย	ปฏิบัติบาง
45	ปฏิบัติประจำ	ปฏิบัติบ่อย	ปฏิบัติประจำ	ปฏิบัติบ่อย	ปฏิบัติบาง	ปฏิบัติบ่อย	ปฏิบัติบ่อย	ปฏิบัติบ่อย
34	ปฏิบัติประจำ	ปฏิบัติประจำ	ปฏิบัติบ่อย	ปฏิบัติบ่อย	ปฏิบัติบาง	ปฏิบัติบ่อย	ปฏิบัติบ่อย	ปฏิบัติประจำ
55	ปฏิบัติประจำ	ปฏิบัติประจำ	ปฏิบัติประจำ	ปฏิบัติประจำ	ปฏิบัติบาง	ปฏิบัติบ่อย	ปฏิบัติบาง	ไม่ปฏิบัติเล
32	ปฏิบัติประจำ	ปฏิบัติบ่อย	ปฏิบัติประจำ	ปฏิบัติประจำ	ปฏิบัติบ่อย	ปฏิบัติประจำ	ปฏิบัติบ่อย	ปฏิบัติประจำ
34	ปฏิบัติบ่อย	ปฏิบัติบาง	ปฏิบัติบ่อย	ปฏิบัติบาง	ปฏิบัติบ่อย	ปฏิบัติบ่อย	ปฏิบัติบาง	ปฏิบัติบาง
51	ปฏิบัติประจำ	ปฏิบัติบาง	ปฏิบัติบ่อย	ปฏิบัติบ่อย	ปฏิบัติบาง	ปฏิบัติประจำ	ปฏิบัติบ่อย	ปฏิบัติบาง
46	ปฏิบัติประจำ	ปฏิบัติบ่อย	ปฏิบัติประจำ	ปฏิบัติบ่อย	ไม่ปฏิบัติเล	ปฏิบัติบาง	ปฏิบัติบาง	ปฏิบัติบาง
29	ปฏิบัติประจำ	ปฏิบัติบ่อย	ปฏิบัติบ่อย	ปฏิบัติบาง	ไม่ปฏิบัติเล	ปฏิบัติบ่อย	ปฏิบัติบ่อย	ปฏิบัติบาง
45	ปฏิบัติประจำ	ปฏิบัติประจำ	ปฏิบัติประจำ	ปฏิบัติประจำ	ปฏิบัติบาง	ปฏิบัติบ่อย	ปฏิบัติบ่อย	ปฏิบัติบ่อย
47	ปฏิบัติบ่อย	ปฏิบัติบ่อย	ปฏิบัติบ่อย	ปฏิบัติบ่อย	ปฏิบัติประจำ	ปฏิบัติบ่อย	ปฏิบัติประจำ	ปฏิบัติประจำ
37	ปฏิบัติประจำ	ปฏิบัติประจำ	ปฏิบัติบ่อย	ปฏิบัติบ่อย	ปฏิบัติประจำ	ปฏิบัติประจำ	ปฏิบัติบ่อย	ปฏิบัติบ่อย
39	ปฏิบัติประจำ	ปฏิบัติประจำ	ปฏิบัติบ่อย	ปฏิบัติบ่อย	ปฏิบัติประจำ	ปฏิบัติประจำ	ปฏิบัติบ่อย	ปฏิบัติประจำ
27	ปฏิบัติประจำ	ปฏิบัติประจำ	ปฏิบัติประจำ	ปฏิบัติประจำ	ปฏิบัติบาง	ไม่ปฏิบัติเล	ปฏิบัติประจำ	ปฏิบัติบ่อย
30	ปฏิบัติบ่อย	ปฏิบัติบ่อย	ปฏิบัติประจำ	ปฏิบัติประจำ	ปฏิบัติบ่อย	ปฏิบัติประจำ	ปฏิบัติบาง	ปฏิบัติบาง

38	ปฏิบัติบ่อย	ปฏิบัติบ่อย	ปฏิบัติบางค	ปฏิบัติบ่อย	ปฏิบัติบ่อย	ปฏิบัติบางค	ปฏิบัติบ่อย	ปฏิบัติบ่อย
32	ปฏิบัติประจำ	ปฏิบัติประจำ	ปฏิบัติประจำ	ปฏิบัติบางค	ปฏิบัติบางค	ปฏิบัติบ่อย	ปฏิบัติประจำ	ปฏิบัติประจำ
54	ปฏิบัติประจำ	ปฏิบัติประจำ	ปฏิบัติประจำ	ปฏิบัติประจำ	ปฏิบัติบ่อย	ปฏิบัติประจำ	ปฏิบัติบ่อย	ปฏิบัติบางค
12	ปฏิบัติบ่อย	ปฏิบัติประจำ	ปฏิบัติประจำ	ปฏิบัติบางค	ไม่ปฏิบัติเล	ไม่ปฏิบัติเล	ปฏิบัติบางค	ปฏิบัติบางค
51	ปฏิบัติประจำ	ปฏิบัติประจำ	ปฏิบัติประจำ	ปฏิบัติประจำ	ปฏิบัติบางค	ปฏิบัติประจำ	ปฏิบัติประจำ	ปฏิบัติประจำ
32	ปฏิบัติประจำ	ปฏิบัติประจำ	ปฏิบัติประจำ	ปฏิบัติบางค	ปฏิบัติบางค	ปฏิบัติบางค	ปฏิบัติบ่อย	ปฏิบัติบางค
29	ปฏิบัติประจำ	ปฏิบัติบ่อย	ปฏิบัติบ่อย	ปฏิบัติบ่อย	ปฏิบัติประจำ	ปฏิบัติประจำ	ปฏิบัติบ่อย	ปฏิบัติบ่อย
54	ปฏิบัติประจำ	ปฏิบัติประจำ	ปฏิบัติประจำ	ปฏิบัติประจำ	ปฏิบัติประจำ	ปฏิบัติประจำ	ปฏิบัติบ่อย	ปฏิบัติบางค
56	ปฏิบัติประจำ	ปฏิบัติประจำ	ปฏิบัติประจำ	ปฏิบัติบ่อย	ปฏิบัติบ่อย	ปฏิบัติประจำ	ปฏิบัติบ่อย	ปฏิบัติบางค
49	ปฏิบัติประจำ	ปฏิบัติประจำ	ปฏิบัติประจำ	ปฏิบัติประจำ	ปฏิบัติประจำ	ปฏิบัติประจำ	ปฏิบัติประจำ	ปฏิบัติประจำ
56	ปฏิบัติประจำ	ปฏิบัติประจำ	ปฏิบัติประจำ	ปฏิบัติประจำ	ปฏิบัติประจำ	ปฏิบัติบางค	ปฏิบัติบางค	ปฏิบัติบางค
19	ปฏิบัติประจำ	ปฏิบัติบางค	ปฏิบัติประจำ	ปฏิบัติบางค	ไม่ปฏิบัติเล	ปฏิบัติบางค	ปฏิบัติบ่อย	ปฏิบัติบ่อย
4	ปฏิบัติบางค	ปฏิบัติบ่อย	ปฏิบัติบางค	ปฏิบัติบางค	ไม่ปฏิบัติเล	ปฏิบัติบางค	ไม่ปฏิบัติเล	ปฏิบัติบางค
37	ปฏิบัติประจำ	ปฏิบัติบ่อย	ปฏิบัติบ่อย	ปฏิบัติบ่อย	ปฏิบัติบางค	ปฏิบัติบางค	ปฏิบัติบางค	ปฏิบัติบางค
30	ปฏิบัติประจำ	ปฏิบัติบางค	ปฏิบัติประจำ	ปฏิบัติบางค	ปฏิบัติประจำ	ปฏิบัติประจำ	ปฏิบัติประจำ	ปฏิบัติประจำ
28	ปฏิบัติประจำ	ปฏิบัติบ่อย	ปฏิบัติบางค	ปฏิบัติประจำ	ปฏิบัติประจำ	ปฏิบัติประจำ	ปฏิบัติประจำ	ปฏิบัติประจำ
44	ปฏิบัติบ่อย	ปฏิบัติประจำ	ปฏิบัติประจำ	ปฏิบัติบ่อย	ปฏิบัติบ่อย	ปฏิบัติบางค	ปฏิบัติบ่อย	ปฏิบัติประจำ
56	ปฏิบัติประจำ	ปฏิบัติประจำ	ปฏิบัติประจำ	ปฏิบัติประจำ	ปฏิบัติบ่อย	ปฏิบัติประจำ	ปฏิบัติบ่อย	ปฏิบัติบางค
47	ปฏิบัติประจำ	ปฏิบัติบ่อย	ปฏิบัติประจำ	ปฏิบัติประจำ	ปฏิบัติบางค	ปฏิบัติบางค	ปฏิบัติบางค	ปฏิบัติบางค
20	ปฏิบัติประจำ	ปฏิบัติบ่อย	ปฏิบัติบางค	ปฏิบัติบางค	ปฏิบัติบ่อย	ปฏิบัติบางค	ปฏิบัติบางค	ปฏิบัติประจำ
18	ปฏิบัติบางค	ปฏิบัติบางค	ปฏิบัติประจำ	ปฏิบัติประจำ	ไม่ปฏิบัติเล	ปฏิบัติบางค	ปฏิบัติบ่อย	ปฏิบัติบ่อย
18	ปฏิบัติประจำ	ปฏิบัติประจำ	ปฏิบัติบางค	ปฏิบัติบ่อย	ปฏิบัติบางค	ไม่ปฏิบัติเล	ปฏิบัติประจำ	ปฏิบัติประจำ
32	ปฏิบัติประจำ	ปฏิบัติประจำ	ปฏิบัติประจำ	ปฏิบัติประจำ	ปฏิบัติบางค	ปฏิบัติบ่อย	ปฏิบัติประจำ	ปฏิบัติประจำ
32	ปฏิบัติประจำ	ปฏิบัติบ่อย	ปฏิบัติบางค	ปฏิบัติประจำ	ปฏิบัติประจำ	ปฏิบัติบางค	ปฏิบัติประจำ	ปฏิบัติบางค
41	ปฏิบัติประจำ	ปฏิบัติบ่อย	ปฏิบัติบ่อย	ปฏิบัติบ่อย	ปฏิบัติบางค	ปฏิบัติบ่อย	ปฏิบัติบางค	ปฏิบัติบางค
49	ปฏิบัติประจำ	ปฏิบัติประจำ	ปฏิบัติประจำ	ปฏิบัติประจำ	ปฏิบัติบ่อย	ปฏิบัติประจำ	ปฏิบัติบ่อย	ปฏิบัติบ่อย
52	ปฏิบัติประจำ	ปฏิบัติประจำ	ปฏิบัติบ่อย	ปฏิบัติบางค	ปฏิบัติบ่อย	ปฏิบัติบ่อย	ปฏิบัติบางค	ปฏิบัติบางค
47	ปฏิบัติประจำ	ปฏิบัติประจำ	ปฏิบัติประจำ	ปฏิบัติประจำ	ปฏิบัติบางค	ปฏิบัติบ่อย	ปฏิบัติประจำ	ปฏิบัติประจำ
33	ปฏิบัติประจำ	ปฏิบัติบางค	ปฏิบัติประจำ	ปฏิบัติบางค	ปฏิบัติบ่อย	ปฏิบัติประจำ	ปฏิบัติบางค	ปฏิบัติบ่อย
41	ปฏิบัติประจำ	ปฏิบัติบางค	ปฏิบัติประจำ	ปฏิบัติบางค	ปฏิบัติบ่อย	ปฏิบัติประจำ	ปฏิบัติบางค	ปฏิบัติบ่อย
38	ปฏิบัติประจำ	ปฏิบัติประจำ	ปฏิบัติประจำ	ปฏิบัติบ่อย	ปฏิบัติประจำ	ปฏิบัติประจำ	ปฏิบัติประจำ	ปฏิบัติบางค
56	ปฏิบัติประจำ	ปฏิบัติประจำ	ปฏิบัติประจำ	ปฏิบัติบางค	ปฏิบัติบางค	ปฏิบัติบางค	ปฏิบัติบ่อย	ปฏิบัติบางค
32	ปฏิบัติประจำ	ปฏิบัติประจำ	ปฏิบัติประจำ	ปฏิบัติบางค	ปฏิบัติบางค	ปฏิบัติบ่อย	ปฏิบัติประจำ	ปฏิบัติประจำ
12	ปฏิบัติบ่อย	ปฏิบัติประจำ	ปฏิบัติประจำ	ปฏิบัติบางค	ไม่ปฏิบัติเล	ไม่ปฏิบัติเล	ปฏิบัติบางค	ปฏิบัติบางค
51	ปฏิบัติประจำ	ปฏิบัติประจำ	ปฏิบัติประจำ	ปฏิบัติประจำ	ปฏิบัติบางค	ปฏิบัติประจำ	ปฏิบัติประจำ	ปฏิบัติประจำ
32	ปฏิบัติประจำ	ปฏิบัติประจำ	ปฏิบัติประจำ	ปฏิบัติบางค	ปฏิบัติบางค	ปฏิบัติบางค	ปฏิบัติบ่อย	ปฏิบัติบางค
29	ปฏิบัติประจำ	ปฏิบัติบ่อย	ปฏิบัติบ่อย	ปฏิบัติบ่อย	ปฏิบัติประจำ	ปฏิบัติประจำ	ปฏิบัติบ่อย	ปฏิบัติบ่อย
54	ปฏิบัติประจำ	ปฏิบัติประจำ	ปฏิบัติประจำ	ปฏิบัติประจำ	ปฏิบัติประจำ	ปฏิบัติประจำ	ปฏิบัติบ่อย	ปฏิบัติบางค
56	ปฏิบัติประจำ	ปฏิบัติประจำ	ปฏิบัติประจำ	ปฏิบัติบ่อย	ปฏิบัติบ่อย	ปฏิบัติประจำ	ปฏิบัติบ่อย	ปฏิบัติบางค
49	ปฏิบัติประจำ	ปฏิบัติประจำ	ปฏิบัติประจำ	ปฏิบัติประจำ	ปฏิบัติประจำ	ปฏิบัติประจำ	ปฏิบัติประจำ	ปฏิบัติบางค
56	ปฏิบัติประจำ	ปฏิบัติประจำ	ปฏิบัติประจำ	ปฏิบัติประจำ	ปฏิบัติประจำ	ปฏิบัติบางค	ปฏิบัติบางค	ปฏิบัติบางค
19	ปฏิบัติประจำ	ปฏิบัติบางค	ปฏิบัติประจำ	ปฏิบัติบางค	ไม่ปฏิบัติเล	ปฏิบัติบางค	ปฏิบัติบ่อย	ปฏิบัติบ่อย
4	ปฏิบัติบางค	ปฏิบัติบ่อย	ปฏิบัติบางค	ปฏิบัติบางค	ไม่ปฏิบัติเล	ปฏิบัติบางค	ไม่ปฏิบัติเล	ปฏิบัติบางค
37	ปฏิบัติประจำ	ปฏิบัติบ่อย	ปฏิบัติบ่อย	ปฏิบัติบ่อย	ปฏิบัติบางค	ปฏิบัติบางค	ปฏิบัติบางค	ปฏิบัติบางค
30	ปฏิบัติประจำ	ปฏิบัติบางค	ปฏิบัติประจำ	ปฏิบัติบางค	ปฏิบัติประจำ	ปฏิบัติประจำ	ปฏิบัติประจำ	ปฏิบัติประจำ
28	ปฏิบัติประจำ	ปฏิบัติบ่อย	ปฏิบัติบางค	ปฏิบัติประจำ	ปฏิบัติประจำ	ปฏิบัติประจำ	ปฏิบัติประจำ	ปฏิบัติประจำ
49	ปฏิบัติบ่อย	ปฏิบัติประจำ	ปฏิบัติประจำ	ปฏิบัติบ่อย	ปฏิบัติบ่อย	ปฏิบัติบางค	ปฏิบัติบ่อย	ปฏิบัติประจำ
54	ปฏิบัติประจำ	ปฏิบัติประจำ	ปฏิบัติประจำ	ปฏิบัติประจำ	ปฏิบัติบ่อย	ปฏิบัติประจำ	ปฏิบัติบ่อย	ปฏิบัติบางค
47	ปฏิบัติประจำ	ปฏิบัติบ่อย	ปฏิบัติประจำ	ปฏิบัติประจำ	ปฏิบัติบางค	ปฏิบัติบางค	ปฏิบัติบางค	ปฏิบัติบางค
19	ปฏิบัติประจำ	ปฏิบัติบ่อย	ปฏิบัติบางค	ปฏิบัติบางค	ปฏิบัติบางค	ปฏิบัติบ่อย	ปฏิบัติบางค	ปฏิบัติบางค
18	ปฏิบัติบางค	ปฏิบัติบางค	ปฏิบัติประจำ	ปฏิบัติประจำ	ไม่ปฏิบัติเล	ปฏิบัติบางค	ปฏิบัติบ่อย	ปฏิบัติบ่อย

18	ปฏิบัติประจำ	ปฏิบัติประจำ	ปฏิบัติบางค	ปฏิบัติบ่อยๆ	ปฏิบัติบางค	ไม่ปฏิบัติเล	ปฏิบัติประจำ	ปฏิบัติประจำ
32	ปฏิบัติประจำ	ปฏิบัติประจำ	ปฏิบัติประจำ	ปฏิบัติประจำ	ปฏิบัติบางค	ปฏิบัติบ่อยๆ	ปฏิบัติประจำ	ปฏิบัติประจำ
32	ปฏิบัติประจำ	ปฏิบัติบ่อยๆ	ปฏิบัติบางค	ปฏิบัติประจำ	ปฏิบัติประจำ	ปฏิบัติบางค	ปฏิบัติประจำ	ปฏิบัติบางค
41	ปฏิบัติประจำ	ปฏิบัติบ่อยๆ	ปฏิบัติบ่อยๆ	ปฏิบัติบ่อยๆ	ปฏิบัติบางค	ปฏิบัติบ่อยๆ	ปฏิบัติบางค	ปฏิบัติบางค
49	ปฏิบัติประจำ	ปฏิบัติประจำ	ปฏิบัติประจำ	ปฏิบัติประจำ	ปฏิบัติบ่อยๆ	ปฏิบัติประจำ	ปฏิบัติบ่อยๆ	ปฏิบัติบ่อยๆ
18	ปฏิบัติบ่อยๆ	ปฏิบัติบ่อยๆ	ปฏิบัติบ่อยๆ	ปฏิบัติบางค	ปฏิบัติบางค	ไม่ปฏิบัติเล	ปฏิบัติบางค	ปฏิบัติบางค
53	ปฏิบัติบ่อยๆ	ปฏิบัติบ่อยๆ	ปฏิบัติบ่อยๆ	ปฏิบัติบ่อยๆ	ปฏิบัติบางค	ปฏิบัติบางค	ปฏิบัติบ่อยๆ	ปฏิบัติบางค
42	ปฏิบัติบ่อยๆ	ปฏิบัติบ่อยๆ	ปฏิบัติบ่อยๆ	ปฏิบัติบ่อยๆ	ปฏิบัติบ่อยๆ	ปฏิบัติบ่อยๆ	ปฏิบัติบางค	ปฏิบัติบางค
42	ปฏิบัติประจำ	ปฏิบัติบ่อยๆ	ปฏิบัติประจำ	ปฏิบัติบ่อยๆ	ไม่ปฏิบัติเล	ไม่ปฏิบัติเล	ปฏิบัติบ่อยๆ	ปฏิบัติบ่อยๆ
32	ปฏิบัติประจำ	ปฏิบัติประจำ	ปฏิบัติประจำ	ปฏิบัติบางค	ปฏิบัติบางค	ปฏิบัติบ่อยๆ	ปฏิบัติประจำ	ปฏิบัติประจำ
12	ปฏิบัติบ่อยๆ	ปฏิบัติประจำ	ปฏิบัติประจำ	ปฏิบัติบางค	ไม่ปฏิบัติเล	ไม่ปฏิบัติเล	ปฏิบัติบางค	ปฏิบัติบางค
51	ปฏิบัติบ่อยๆ	ปฏิบัติประจำ	ปฏิบัติประจำ	ปฏิบัติบางค	ไม่ปฏิบัติเล	ไม่ปฏิบัติเล	ปฏิบัติบางค	ปฏิบัติบางค
32	ปฏิบัติประจำ	ปฏิบัติประจำ	ปฏิบัติประจำ	ปฏิบัติประจำ	ปฏิบัติบางค	ปฏิบัติบางค	ปฏิบัติบางค	ปฏิบัติบ่อยๆ
28	ปฏิบัติประจำ	ปฏิบัติบ่อยๆ	ปฏิบัติบ่อยๆ	ปฏิบัติบ่อยๆ	ปฏิบัติประจำ	ปฏิบัติประจำ	ปฏิบัติบ่อยๆ	ปฏิบัติบ่อยๆ
54	ปฏิบัติประจำ	ปฏิบัติประจำ	ปฏิบัติประจำ	ปฏิบัติประจำ	ปฏิบัติประจำ	ปฏิบัติประจำ	ปฏิบัติบ่อยๆ	ปฏิบัติบางค
56	ปฏิบัติประจำ	ปฏิบัติประจำ	ปฏิบัติประจำ	ปฏิบัติบ่อยๆ	ปฏิบัติบ่อยๆ	ปฏิบัติประจำ	ปฏิบัติบ่อยๆ	ปฏิบัติบางค
49	ปฏิบัติประจำ	ปฏิบัติประจำ	ปฏิบัติประจำ	ปฏิบัติประจำ	ปฏิบัติประจำ	ปฏิบัติประจำ	ปฏิบัติประจำ	ปฏิบัติบางค
56	ปฏิบัติประจำ	ปฏิบัติประจำ	ปฏิบัติประจำ	ปฏิบัติประจำ	ปฏิบัติประจำ	ปฏิบัติบางค	ปฏิบัติบางค	ปฏิบัติบางค
19	ปฏิบัติประจำ	ปฏิบัติบางค	ปฏิบัติประจำ	ปฏิบัติบางค	ไม่ปฏิบัติเล	ปฏิบัติบางค	ปฏิบัติบ่อยๆ	ปฏิบัติบ่อยๆ
5	ปฏิบัติบางค	ปฏิบัติบ่อยๆ	ปฏิบัติบางค	ปฏิบัติบางค	ไม่ปฏิบัติเล	ปฏิบัติบางค	ไม่ปฏิบัติเล	ปฏิบัติบางค
37	ปฏิบัติประจำ	ปฏิบัติบ่อยๆ	ปฏิบัติบ่อยๆ	ปฏิบัติบ่อยๆ	ปฏิบัติบางค	ปฏิบัติบางค	ปฏิบัติบางค	ปฏิบัติบางค
30	ปฏิบัติประจำ	ปฏิบัติบางค	ปฏิบัติประจำ	ปฏิบัติบางค	ปฏิบัติประจำ	ปฏิบัติประจำ	ปฏิบัติประจำ	ปฏิบัติประจำ
29	ปฏิบัติประจำ	ปฏิบัติบ่อยๆ	ปฏิบัติบางค	ปฏิบัติประจำ	ปฏิบัติประจำ	ปฏิบัติประจำ	ปฏิบัติประจำ	ปฏิบัติประจำ
44	ปฏิบัติบ่อยๆ	ปฏิบัติประจำ	ปฏิบัติประจำ	ปฏิบัติบ่อยๆ	ปฏิบัติบ่อยๆ	ปฏิบัติบางค	ปฏิบัติบ่อยๆ	ปฏิบัติประจำ
42	ปฏิบัติประจำ	ปฏิบัติประจำ	ปฏิบัติประจำ	ปฏิบัติประจำ	ปฏิบัติประจำ	ปฏิบัติบางค	ปฏิบัติบางค	ปฏิบัติบางค
30	ปฏิบัติบ่อยๆ	ปฏิบัติบางค	ปฏิบัติประจำ	ปฏิบัติบางค	ไม่ปฏิบัติเล	ไม่ปฏิบัติเล	ปฏิบัติบ่อยๆ	ปฏิบัติบ่อยๆ
51	ปฏิบัติประจำ	ปฏิบัติประจำ	ปฏิบัติประจำ	ปฏิบัติประจำ	ปฏิบัติประจำ	ปฏิบัติบ่อยๆ	ปฏิบัติบางค	ปฏิบัติบางค
21	ปฏิบัติบางค	ปฏิบัติบางค	ปฏิบัติบ่อยๆ	ปฏิบัติบางค	ไม่ปฏิบัติเล	ไม่ปฏิบัติเล	ปฏิบัติบางค	ปฏิบัติบางค
41	ปฏิบัติประจำ	ปฏิบัติบ่อยๆ	ปฏิบัติบ่อยๆ	ปฏิบัติบางค	ปฏิบัติบางค	ปฏิบัติบางค	ปฏิบัติบางค	ปฏิบัติบางค
26	ปฏิบัติบางค	ปฏิบัติบางค	ปฏิบัติบางค	ปฏิบัติบางค	ไม่ปฏิบัติเล	ไม่ปฏิบัติเล	ปฏิบัติบ่อยๆ	ปฏิบัติบ่อยๆ
54	ปฏิบัติประจำ	ปฏิบัติบ่อยๆ	ปฏิบัติประจำ	ปฏิบัติประจำ	ปฏิบัติบ่อยๆ	ปฏิบัติบางค	ปฏิบัติบางค	ปฏิบัติบางค
30	ปฏิบัติบางค	ปฏิบัติบางค	ปฏิบัติบางค	ปฏิบัติบางค	ปฏิบัติบางค	ปฏิบัติบางค	ปฏิบัติบางค	ปฏิบัติบางค
46	ปฏิบัติบ่อยๆ	ปฏิบัติบางค	ปฏิบัติบ่อยๆ	ปฏิบัติบางค	ปฏิบัติบางค	ปฏิบัติบางค	ปฏิบัติบางค	ปฏิบัติบางค
25	ปฏิบัติบางค	ปฏิบัติบางค	ปฏิบัติบางค	ปฏิบัติบางค	ไม่ปฏิบัติเล	ไม่ปฏิบัติเล	ไม่ปฏิบัติเล	ปฏิบัติบางค
56	ปฏิบัติบ่อยๆ	ปฏิบัติบ่อยๆ	ปฏิบัติบ่อยๆ	ปฏิบัติบ่อยๆ	ปฏิบัติบ่อยๆ	ปฏิบัติบ่อยๆ	ปฏิบัติบ่อยๆ	ปฏิบัติบ่อยๆ
37	ปฏิบัติบางค	ปฏิบัติบางค	ปฏิบัติบางค	ปฏิบัติบางค	ปฏิบัติบางค	ปฏิบัติบางค	ปฏิบัติบางค	ปฏิบัติบางค
56	ปฏิบัติบ่อยๆ	ปฏิบัติบ่อยๆ	ปฏิบัติบ่อยๆ	ปฏิบัติบางค	ไม่ปฏิบัติเล	ไม่ปฏิบัติเล	ปฏิบัติบ่อยๆ	ปฏิบัติประจำ
43	ปฏิบัติประจำ	ปฏิบัติบางค	ปฏิบัติบ่อยๆ	ปฏิบัติบ่อยๆ	ปฏิบัติบางค	ปฏิบัติบางค	ปฏิบัติบ่อยๆ	ปฏิบัติบ่อยๆ
35	ปฏิบัติประจำ	ปฏิบัติบางค	ปฏิบัติบ่อยๆ	ปฏิบัติบ่อยๆ	ปฏิบัติบางค	ปฏิบัติบางค	ปฏิบัติบ่อยๆ	ปฏิบัติบ่อยๆ
56	ปฏิบัติประจำ	ปฏิบัติประจำ	ปฏิบัติประจำ	ปฏิบัติประจำ	ปฏิบัติประจำ	ปฏิบัติประจำ	ปฏิบัติประจำ	ปฏิบัติประจำ
39	ปฏิบัติประจำ	ปฏิบัติบ่อยๆ	ปฏิบัติประจำ	ปฏิบัติบ่อยๆ	ปฏิบัติบางค	ปฏิบัติบางค	ปฏิบัติบ่อยๆ	ปฏิบัติบ่อยๆ
39	ปฏิบัติบ่อยๆ	ปฏิบัติบางค	ปฏิบัติบ่อยๆ	ปฏิบัติบางค	ไม่ปฏิบัติเล	ไม่ปฏิบัติเล	ปฏิบัติประจำ	ปฏิบัติประจำ
56	ปฏิบัติประจำ	ปฏิบัติประจำ	ปฏิบัติประจำ	ปฏิบัติบางค	ปฏิบัติบ่อยๆ	ปฏิบัติบางค	ปฏิบัติบ่อยๆ	ปฏิบัติบ่อยๆ
56	ปฏิบัติประจำ	ปฏิบัติประจำ	ปฏิบัติประจำ	ปฏิบัติประจำ	ปฏิบัติบ่อยๆ	ปฏิบัติประจำ	ปฏิบัติบ่อยๆ	ปฏิบัติบางค
39	ปฏิบัติบางค	ปฏิบัติบางค	ปฏิบัติบางค	ปฏิบัติบางค	ปฏิบัติบางค	ปฏิบัติบางค	ปฏิบัติบางค	ปฏิบัติบางค
27	ปฏิบัติบางค	ปฏิบัติบางค	ปฏิบัติบ่อยๆ	ปฏิบัติบางค	ปฏิบัติบางค	ปฏิบัติบางค	ปฏิบัติบ่อยๆ	ปฏิบัติบ่อยๆ
41	ปฏิบัติบ่อยๆ	ปฏิบัติบ่อยๆ	ปฏิบัติบ่อยๆ	ปฏิบัติบ่อยๆ	ปฏิบัติบางค	ปฏิบัติบางค	ปฏิบัติบ่อยๆ	ปฏิบัติบ่อยๆ
25	ปฏิบัติบางค	ปฏิบัติบางค	ปฏิบัติบางค	ปฏิบัติบางค	ปฏิบัติบางค	ปฏิบัติบางค	ปฏิบัติบางค	ปฏิบัติบางค
44	ปฏิบัติประจำ	ปฏิบัติประจำ	ปฏิบัติประจำ	ปฏิบัติประจำ	ปฏิบัติบ่อยๆ	ปฏิบัติบางค	ปฏิบัติบางค	ปฏิบัติประจำ
25	ปฏิบัติบ่อยๆ	ปฏิบัติบางค	ปฏิบัติบ่อยๆ	ปฏิบัติบางค	ไม่ปฏิบัติเล	ไม่ปฏิบัติเล	ปฏิบัติบ่อยๆ	ปฏิบัติบางค
49	ปฏิบัติบ่อยๆ	ปฏิบัติประจำ	ปฏิบัติประจำ	ปฏิบัติบางค	ปฏิบัติบางค	ปฏิบัติบางค	ปฏิบัติบ่อยๆ	ปฏิบัติบ่อยๆ

29	ปฏิบัติบ่อย	ปฏิบัติบางค	ปฏิบัติบ่อย	ไม่ปฏิบัติเล	ไม่ปฏิบัติเล	ไม่ปฏิบัติเล	ปฏิบัติบ่อย	ปฏิบัติบางค
0	ปฏิบัติประจำ	ปฏิบัติประจำ	ปฏิบัติประจำ	ปฏิบัติประจำ	ปฏิบัติบางค	ปฏิบัติบางค	ปฏิบัติบางค	ปฏิบัติบางค
45	ปฏิบัติประจำ	ปฏิบัติบ่อย	ปฏิบัติประจำ	ปฏิบัติประจำ	ปฏิบัติบางค	ปฏิบัติบ่อย	ปฏิบัติบางค	ปฏิบัติบางค
42	ปฏิบัติบ่อย	ปฏิบัติประจำ	ปฏิบัติประจำ	ปฏิบัติบ่อย	ไม่ปฏิบัติเล	ปฏิบัติบ่อย	ปฏิบัติบ่อย	ปฏิบัติบางค
34	ปฏิบัติประจำ	ปฏิบัติบ่อย	ปฏิบัติประจำ	ปฏิบัติประจำ	ปฏิบัติบ่อย	ปฏิบัติบ่อย	ปฏิบัติบ่อย	ปฏิบัติบ่อย
45	ปฏิบัติประจำ	ปฏิบัติบ่อย	ปฏิบัติประจำ	ปฏิบัติบางค	ปฏิบัติบางค	ปฏิบัติบ่อย	ปฏิบัติบ่อย	ปฏิบัติบ่อย
21	ปฏิบัติประจำ	ปฏิบัติบางค	ปฏิบัติบางค	ปฏิบัติบางค	ปฏิบัติบางค	ปฏิบัติประจำ	ปฏิบัติบางค	ปฏิบัติบางค
34	ปฏิบัติบ่อย	ปฏิบัติบ่อย	ปฏิบัติบ่อย	ปฏิบัติบางค	ปฏิบัติบางค	ปฏิบัติบ่อย	ปฏิบัติบ่อย	ปฏิบัติบ่อย
45	ปฏิบัติประจำ	ปฏิบัติบ่อย	ปฏิบัติประจำ	ปฏิบัติบางค	ไม่ปฏิบัติเล	ปฏิบัติบางค	ปฏิบัติประจำ	ปฏิบัติประจำ
54	ปฏิบัติประจำ	ปฏิบัติประจำ	ปฏิบัติประจำ	ปฏิบัติประจำ	ปฏิบัติบางค	ปฏิบัติบ่อย	ปฏิบัติบางค	ปฏิบัติบ่อย
34	ปฏิบัติบ่อย	ปฏิบัติบ่อย	ปฏิบัติบ่อย	ปฏิบัติบางค	ปฏิบัติบางค	ปฏิบัติบ่อย	ปฏิบัติบ่อย	ปฏิบัติบ่อย
32	ปฏิบัติบ่อย	ปฏิบัติบ่อย	ปฏิบัติบ่อย	ปฏิบัติบ่อย	ปฏิบัติบางค	ปฏิบัติบางค	ปฏิบัติบ่อย	ปฏิบัติบ่อย
25	ปฏิบัติประจำ	ปฏิบัติประจำ	ปฏิบัติประจำ	ปฏิบัติประจำ	ปฏิบัติบางค	ปฏิบัติประจำ	ปฏิบัติบางค	ปฏิบัติบางค
52	ปฏิบัติประจำ	ปฏิบัติประจำ	ปฏิบัติประจำ	ปฏิบัติประจำ	ปฏิบัติบ่อย	ปฏิบัติประจำ	ปฏิบัติบ่อย	ปฏิบัติบ่อย
43	ปฏิบัติประจำ	ปฏิบัติประจำ	ปฏิบัติประจำ	ปฏิบัติประจำ	ปฏิบัติบางค	ปฏิบัติประจำ	ปฏิบัติบางค	ปฏิบัติบางค
17	ปฏิบัติบ่อย	ปฏิบัติบ่อย	ปฏิบัติประจำ	ปฏิบัติประจำ	ปฏิบัติประจำ	ปฏิบัติบางค	ปฏิบัติบ่อย	ปฏิบัติบางค
51	ปฏิบัติบ่อย	ปฏิบัติบ่อย	ปฏิบัติประจำ	ปฏิบัติบ่อย	ปฏิบัติบางค	ปฏิบัติประจำ	ปฏิบัติบ่อย	ปฏิบัติบ่อย
39	ปฏิบัติบ่อย	ปฏิบัติบ่อย	ปฏิบัติบ่อย	ปฏิบัติบ่อย	ไม่ปฏิบัติเล	ไม่ปฏิบัติเล	ปฏิบัติบ่อย	ปฏิบัติบางค
37	ปฏิบัติประจำ	ปฏิบัติประจำ	ปฏิบัติประจำ	ปฏิบัติประจำ	ปฏิบัติบางค	ปฏิบัติบ่อย	ปฏิบัติบางค	ปฏิบัติบางค
49	ปฏิบัติประจำ	ปฏิบัติบ่อย	ปฏิบัติประจำ	ปฏิบัติบ่อย	ไม่ปฏิบัติเล	ปฏิบัติบางค	ปฏิบัติบ่อย	ปฏิบัติประจำ
39	ปฏิบัติบ่อย	ปฏิบัติบ่อย	ปฏิบัติบ่อย	ปฏิบัติประจำ	ปฏิบัติบางค	ปฏิบัติประจำ	ปฏิบัติประจำ	ปฏิบัติประจำ
53	ปฏิบัติประจำ	ปฏิบัติบางค	ปฏิบัติประจำ	ปฏิบัติประจำ	ไม่ปฏิบัติเล	ปฏิบัติประจำ	ปฏิบัติประจำ	ปฏิบัติประจำ
39	ปฏิบัติประจำ	ปฏิบัติประจำ	ปฏิบัติประจำ	ปฏิบัติประจำ	ปฏิบัติบางค	ปฏิบัติประจำ	ปฏิบัติบางค	ปฏิบัติบางค
37	ปฏิบัติบ่อย	ปฏิบัติประจำ	ปฏิบัติประจำ	ปฏิบัติบางค	ไม่ปฏิบัติเล	ไม่ปฏิบัติเล	ปฏิบัติประจำ	ไม่ปฏิบัติเล
41	ปฏิบัติประจำ	ปฏิบัติประจำ	ปฏิบัติประจำ	ปฏิบัติประจำ	ปฏิบัติบ่อย	ปฏิบัติบางค	ปฏิบัติบางค	ปฏิบัติบางค
49	ปฏิบัติประจำ	ปฏิบัติประจำ	ปฏิบัติประจำ	ปฏิบัติประจำ	ปฏิบัติประจำ	ปฏิบัติบ่อย	ปฏิบัติบ่อย	ปฏิบัติบางค
20	ปฏิบัติประจำ	ปฏิบัติประจำ	ปฏิบัติบางค	ปฏิบัติบ่อย	ไม่ปฏิบัติเล	ปฏิบัติประจำ	ปฏิบัติบางค	ปฏิบัติบางค
42	ปฏิบัติประจำ	ปฏิบัติประจำ	ปฏิบัติประจำ	ปฏิบัติประจำ	ปฏิบัติบางค	ปฏิบัติประจำ	ปฏิบัติประจำ	ปฏิบัติบางค
38	ปฏิบัติประจำ	ปฏิบัติบ่อย	ปฏิบัติประจำ	ปฏิบัติบ่อย	ไม่ปฏิบัติเล	ปฏิบัติบ่อย	ปฏิบัติบ่อย	ปฏิบัติบางค
20	ปฏิบัติประจำ	ปฏิบัติประจำ	ปฏิบัติประจำ	ปฏิบัติบางค	ไม่ปฏิบัติเล	ปฏิบัติประจำ	ปฏิบัติบ่อย	ไม่ปฏิบัติเล
46	ปฏิบัติบางค	ปฏิบัติบางค	ปฏิบัติบางค	ปฏิบัติบางค	ปฏิบัติบางค	ปฏิบัติบางค	ปฏิบัติบางค	ปฏิบัติบางค
48	ปฏิบัติประจำ	ปฏิบัติประจำ	ปฏิบัติประจำ	ปฏิบัติประจำ	ไม่ปฏิบัติเล	ปฏิบัติบ่อย	ปฏิบัติบางค	ปฏิบัติบางค
33	ปฏิบัติประจำ	ปฏิบัติบ่อย	ปฏิบัติบ่อย	ปฏิบัติบ่อย	ไม่ปฏิบัติเล	ปฏิบัติบางค	ปฏิบัติบางค	ปฏิบัติบางค
43	ปฏิบัติประจำ	ปฏิบัติบ่อย	ปฏิบัติประจำ	ปฏิบัติบางค	ปฏิบัติบางค	ปฏิบัติบ่อย	ปฏิบัติบ่อย	ปฏิบัติบางค
44	ปฏิบัติบ่อย	ปฏิบัติบางค	ปฏิบัติบ่อย	ปฏิบัติบางค	ไม่ปฏิบัติเล	ปฏิบัติบางค	ปฏิบัติบางค	ปฏิบัติบางค
49	ปฏิบัติประจำ	ปฏิบัติบ่อย	ปฏิบัติประจำ	ปฏิบัติบ่อย	ปฏิบัติบางค	ปฏิบัติบ่อย	ปฏิบัติบ่อย	ปฏิบัติบ่อย
47	ปฏิบัติประจำ	ปฏิบัติบ่อย	ปฏิบัติประจำ	ปฏิบัติบางค	ไม่ปฏิบัติเล	ปฏิบัติประจำ	ปฏิบัติบางค	ปฏิบัติบางค
35	ปฏิบัติบ่อย	ปฏิบัติบ่อย	ปฏิบัติบางค	ปฏิบัติบางค	ไม่ปฏิบัติเล	ปฏิบัติบ่อย	ปฏิบัติบ่อย	ปฏิบัติบางค
44	ปฏิบัติประจำ	ปฏิบัติประจำ	ปฏิบัติประจำ	ปฏิบัติประจำ	ปฏิบัติบางค	ปฏิบัติบ่อย	ปฏิบัติประจำ	ปฏิบัติบ่อย
44	ปฏิบัติประจำ	ปฏิบัติประจำ	ปฏิบัติประจำ	ปฏิบัติประจำ	ปฏิบัติบ่อย	ปฏิบัติบางค	ปฏิบัติบางค	ปฏิบัติประจำ
25	ปฏิบัติบ่อย	ปฏิบัติบางค	ปฏิบัติบ่อย	ปฏิบัติบางค	ไม่ปฏิบัติเล	ไม่ปฏิบัติเล	ปฏิบัติบ่อย	ปฏิบัติบางค
49	ปฏิบัติบ่อย	ปฏิบัติประจำ	ปฏิบัติประจำ	ปฏิบัติบางค	ปฏิบัติบางค	ปฏิบัติบางค	ปฏิบัติบ่อย	ปฏิบัติบ่อย
29	ปฏิบัติบ่อย	ปฏิบัติบางค	ปฏิบัติบ่อย	ไม่ปฏิบัติเล	ไม่ปฏิบัติเล	ไม่ปฏิบัติเล	ปฏิบัติบ่อย	ปฏิบัติบางค
0	ปฏิบัติประจำ	ปฏิบัติประจำ	ปฏิบัติประจำ	ปฏิบัติประจำ	ปฏิบัติบางค	ปฏิบัติบางค	ปฏิบัติบางค	ปฏิบัติบางค
45	ปฏิบัติประจำ	ปฏิบัติบ่อย	ปฏิบัติประจำ	ปฏิบัติประจำ	ปฏิบัติบางค	ปฏิบัติบ่อย	ปฏิบัติบางค	ปฏิบัติบางค
42	ปฏิบัติบ่อย	ปฏิบัติประจำ	ปฏิบัติประจำ	ปฏิบัติบ่อย	ไม่ปฏิบัติเล	ปฏิบัติบ่อย	ปฏิบัติบ่อย	ปฏิบัติบางค
39	ปฏิบัติประจำ	ปฏิบัติประจำ	ปฏิบัติประจำ	ปฏิบัติประจำ	ปฏิบัติบางค	ปฏิบัติประจำ	ปฏิบัติบ่อย	ปฏิบัติบางค
40	ปฏิบัติประจำ	ปฏิบัติบ่อย	ปฏิบัติประจำ	ปฏิบัติบางค	ปฏิบัติประจำ	ปฏิบัติบ่อย	ปฏิบัติบ่อย	ปฏิบัติบ่อย
48	ปฏิบัติประจำ	ปฏิบัติประจำ	ปฏิบัติประจำ	ปฏิบัติบ่อย	ปฏิบัติบ่อย	ปฏิบัติประจำ	ปฏิบัติบ่อย	ปฏิบัติประจำ

oral9	oral10	oral11	oral12	oral13	oral14	oral15	Sum_oral	caries
ปฏิบัติบ่อย	ปฏิบัติบ่อย	ปฏิบัติบางค	ปฏิบัติบ่อย	ปฏิบัติประจำ	ปฏิบัติบ่อย	ไม่ปฏิบัติเล	31	2
ปฏิบัติบ่อย	ปฏิบัติประจำ	ปฏิบัติบางค	ปฏิบัติบ่อย	ปฏิบัติประจำ	ปฏิบัติบ่อย	ไม่ปฏิบัติเล	30	5
ปฏิบัติบ่อย	ปฏิบัติประจำ	ปฏิบัติบ่อย	ปฏิบัติบางค	ปฏิบัติบ่อย	ปฏิบัติบ่อย	ไม่ปฏิบัติเล	28	7
ปฏิบัติบางค	ไม่ปฏิบัติเล	ไม่ปฏิบัติเล	ไม่ปฏิบัติเล	ปฏิบัติประจำ	ไม่ปฏิบัติเล	ไม่ปฏิบัติเล	20	7
ปฏิบัติบางค	ปฏิบัติบ่อย	ปฏิบัติบางค	ไม่ปฏิบัติเล	ปฏิบัติประจำ	ปฏิบัติบ่อย	ไม่ปฏิบัติเล	19	6
ปฏิบัติประจำ	ปฏิบัติประจำ	ปฏิบัติประจำ	ปฏิบัติประจำ	ปฏิบัติประจำ	ปฏิบัติประจำ	ไม่ปฏิบัติเล	39	2
ปฏิบัติบางค	ปฏิบัติบ่อย	ปฏิบัติบ่อย	ปฏิบัติบางค	ปฏิบัติบางค	ปฏิบัติบางค	ไม่ปฏิบัติเล	18	0
ปฏิบัติบางค	ปฏิบัติบางค	ไม่ปฏิบัติเล	ไม่ปฏิบัติเล	ปฏิบัติประจำ	ไม่ปฏิบัติเล	ปฏิบัติบางค	26	0
ปฏิบัติบ่อย	ปฏิบัติบางค	ปฏิบัติบ่อย	ปฏิบัติบางค	ปฏิบัติบ่อย	ปฏิบัติบ่อย	ไม่ปฏิบัติเล	22	0
ปฏิบัติประจำ	ปฏิบัติประจำ	ปฏิบัติประจำ	ปฏิบัติประจำ	ปฏิบัติบางค	ปฏิบัติประจำ	ไม่ปฏิบัติเล	34	2
ปฏิบัติบ่อย	ปฏิบัติบางค	ปฏิบัติบางค	ปฏิบัติบางค	ปฏิบัติประจำ	ปฏิบัติบ่อย	ไม่ปฏิบัติเล	28	0
ปฏิบัติประจำ	ปฏิบัติบางค	ไม่ปฏิบัติเล	ปฏิบัติบางค	ปฏิบัติประจำ	ไม่ปฏิบัติเล	ไม่ปฏิบัติเล	25	0
ปฏิบัติบ่อย	ปฏิบัติบางค	ปฏิบัติบางค	ปฏิบัติบางค	ปฏิบัติบางค	ปฏิบัติบางค	ไม่ปฏิบัติเล	23	0
ปฏิบัติบ่อย	ปฏิบัติบางค	ปฏิบัติบางค	ปฏิบัติบางค	ปฏิบัติบ่อย	ปฏิบัติบ่อย	ไม่ปฏิบัติเล	22	0
ปฏิบัติบางค	ปฏิบัติบางค	ปฏิบัติบางค	ปฏิบัติบางค	ปฏิบัติบ่อย	ไม่ปฏิบัติเล	ไม่ปฏิบัติเล	22	1
ปฏิบัติบางค	ปฏิบัติบางค	ปฏิบัติบางค	ปฏิบัติบางค	ปฏิบัติประจำ	ปฏิบัติบ่อย	ไม่ปฏิบัติเล	20	4
ปฏิบัติบางค	ปฏิบัติประจำ	ปฏิบัติบ่อย	ปฏิบัติบางค	ปฏิบัติประจำ	ปฏิบัติประจำ	ไม่ปฏิบัติเล	31	0
ปฏิบัติบ่อย	ปฏิบัติบ่อย	ปฏิบัติบ่อย	ปฏิบัติบ่อย	ปฏิบัติบางค	ปฏิบัติบางค	ไม่ปฏิบัติเล	21	0
ปฏิบัติบ่อย	ปฏิบัติบางค	ปฏิบัติบางค	ปฏิบัติบางค	ปฏิบัติบ่อย	ปฏิบัติบ่อย	ไม่ปฏิบัติเล	23	0
ปฏิบัติบางค	ปฏิบัติบ่อย	ปฏิบัติบางค	ปฏิบัติบางค	ปฏิบัติบางค	ปฏิบัติบางค	ไม่ปฏิบัติเล	26	1
ปฏิบัติบ่อย	ปฏิบัติบ่อย	ปฏิบัติบางค	ปฏิบัติบางค	ปฏิบัติบางค	ปฏิบัติบางค	ไม่ปฏิบัติเล	26	3
ปฏิบัติบ่อย	ปฏิบัติประจำ	ปฏิบัติบ่อย	ปฏิบัติบางค	ปฏิบัติบ่อย	ปฏิบัติบ่อย	ไม่ปฏิบัติเล	28	4
ปฏิบัติประจำ	ปฏิบัติประจำ	ปฏิบัติบ่อย	ปฏิบัติบางค	ปฏิบัติประจำ	ปฏิบัติประจำ	ไม่ปฏิบัติเล	32	1
ปฏิบัติบางค	ปฏิบัติบางค	ปฏิบัติบ่อย	ปฏิบัติบ่อย	ปฏิบัติบ่อย	ปฏิบัติบ่อย	ปฏิบัติประจำ	32	2
ปฏิบัติบ่อย	ปฏิบัติบ่อย	ปฏิบัติบ่อย	ปฏิบัติประจำ	ปฏิบัติประจำ	ปฏิบัติประจำ	ไม่ปฏิบัติเล	35	1
ปฏิบัติบางค	ปฏิบัติบางค	ปฏิบัติบางค	ปฏิบัติบางค	ปฏิบัติประจำ	ปฏิบัติบ่อย	ไม่ปฏิบัติเล	24	0
ปฏิบัติประจำ	ปฏิบัติประจำ	ปฏิบัติประจำ	ปฏิบัติประจำ	ปฏิบัติบ่อย	ปฏิบัติบ่อย	ไม่ปฏิบัติเล	30	1
ปฏิบัติประจำ	ปฏิบัติประจำ	ปฏิบัติประจำ	ไม่ปฏิบัติเล	ปฏิบัติบางค	ไม่ปฏิบัติเล	ไม่ปฏิบัติเล	24	6
ปฏิบัติประจำ	ปฏิบัติบ่อย	ปฏิบัติบ่อย	ปฏิบัติบางค	ปฏิบัติประจำ	ปฏิบัติบ่อย	ไม่ปฏิบัติเล	30	0
ปฏิบัติบ่อย	ปฏิบัติบ่อย	ปฏิบัติบ่อย	ปฏิบัติบางค	ปฏิบัติประจำ	ไม่ปฏิบัติเล	ไม่ปฏิบัติเล	25	3
ปฏิบัติบางค	ปฏิบัติบางค	ปฏิบัติบางค	ปฏิบัติบางค	ปฏิบัติบางค	ปฏิบัติบางค	ไม่ปฏิบัติเล	14	5
ปฏิบัติประจำ	ปฏิบัติประจำ	ปฏิบัติบ่อย	ปฏิบัติบ่อย	ปฏิบัติประจำ	ไม่ปฏิบัติเล	ปฏิบัติประจำ	33	5
ปฏิบัติบ่อย	ปฏิบัติบ่อย	ปฏิบัติบ่อย	ไม่ปฏิบัติเล	ปฏิบัติบ่อย	ปฏิบัติบ่อย	ไม่ปฏิบัติเล	29	4
ปฏิบัติบ่อย	ปฏิบัติบ่อย	ปฏิบัติบ่อย	ปฏิบัติบ่อย	ปฏิบัติบ่อย	ปฏิบัติบ่อย	ไม่ปฏิบัติเล	27	0
ปฏิบัติบางค	ปฏิบัติบางค	ปฏิบัติบางค	ปฏิบัติบางค	ปฏิบัติบ่อย	ปฏิบัติบ่อย	ไม่ปฏิบัติเล	24	2
ปฏิบัติประจำ	ปฏิบัติประจำ	ปฏิบัติประจำ	ปฏิบัติบางค	ปฏิบัติประจำ	ปฏิบัติประจำ	ไม่ปฏิบัติเล	31	0
ปฏิบัติบางค	ปฏิบัติบางค	ปฏิบัติบางค	ไม่ปฏิบัติเล	ปฏิบัติบ่อย	ปฏิบัติบ่อย	ไม่ปฏิบัติเล	14	14
ปฏิบัติบางค	ปฏิบัติบ่อย	ปฏิบัติบ่อย	ไม่ปฏิบัติเล	ปฏิบัติประจำ	ปฏิบัติบ่อย	ไม่ปฏิบัติเล	27	0
ปฏิบัติบางค	ปฏิบัติบางค	ปฏิบัติบางค	ไม่ปฏิบัติเล	ปฏิบัติบางค	ปฏิบัติบางค	ไม่ปฏิบัติเล	22	0
ปฏิบัติประจำ	ปฏิบัติประจำ	ปฏิบัติบางค	ปฏิบัติบ่อย	ปฏิบัติบางค	ปฏิบัติบางค	ไม่ปฏิบัติเล	26	7
ปฏิบัติบ่อย	ปฏิบัติบ่อย	ปฏิบัติบางค	ปฏิบัติบางค	ปฏิบัติบ่อย	ปฏิบัติบ่อย	ไม่ปฏิบัติเล	24	0
ปฏิบัติบ่อย	ปฏิบัติประจำ	ปฏิบัติบ่อย	ปฏิบัติบางค	ปฏิบัติประจำ	ปฏิบัติบ่อย	ไม่ปฏิบัติเล	26	6
ปฏิบัติประจำ	ปฏิบัติประจำ	ปฏิบัติประจำ	ปฏิบัติบางค	ปฏิบัติบ่อย	ปฏิบัติบ่อย	ไม่ปฏิบัติเล	27	2
ปฏิบัติบางค	ปฏิบัติบางค	ปฏิบัติบางค	ปฏิบัติบางค	ปฏิบัติบางค	ปฏิบัติบางค	ปฏิบัติบางค	15	3
ปฏิบัติบ่อย	ปฏิบัติบ่อย	ปฏิบัติบ่อย	ปฏิบัติบางค	ปฏิบัติบางค	ปฏิบัติบ่อย	ไม่ปฏิบัติเล	25	0
ปฏิบัติประจำ	ปฏิบัติประจำ	ปฏิบัติประจำ	ปฏิบัติบางค	ปฏิบัติบางค	ปฏิบัติบางค	ไม่ปฏิบัติเล	26	0
ปฏิบัติประจำ	ปฏิบัติประจำ	ปฏิบัติประจำ	ปฏิบัติบ่อย	ปฏิบัติบ่อย	ไม่ปฏิบัติเล	ไม่ปฏิบัติเล	27	2
ปฏิบัติบางค	ปฏิบัติบางค	ปฏิบัติบางค	ปฏิบัติบางค	ปฏิบัติบางค	ปฏิบัติบางค	ไม่ปฏิบัติเล	23	0
ปฏิบัติบ่อย	ปฏิบัติบางค	ปฏิบัติบางค	ปฏิบัติบางค	ปฏิบัติบ่อย	ปฏิบัติบ่อย	ไม่ปฏิบัติเล	23	4
ปฏิบัติบางค	ปฏิบัติบางค	ปฏิบัติบางค	ปฏิบัติบางค	ปฏิบัติบ่อย	ไม่ปฏิบัติเล	ไม่ปฏิบัติเล	22	0

ปฏิบัติประจำ	ปฏิบัติประจำ	ปฏิบัติบ่อย	ปฏิบัติบางค	ปฏิบัติบ่อย	ปฏิบัติบ่อย	ไม่ปฏิบัติเล	29	7
ปฏิบัติประจำ	ไม่ปฏิบัติเล	ไม่ปฏิบัติเล	ปฏิบัติประจำ	ปฏิบัติประจำ	ไม่ปฏิบัติเล	ไม่ปฏิบัติเล	23	3
ปฏิบัติบ่อย	ปฏิบัติบ่อย	ปฏิบัติบ่อย	ปฏิบัติบางค	ปฏิบัติประจำ	ปฏิบัติบ่อย	ไม่ปฏิบัติเล	27	0
ปฏิบัติประจำ	ปฏิบัติบ่อย	ปฏิบัติบ่อย	ปฏิบัติประจำ	ปฏิบัติบ่อย	ปฏิบัติบ่อย	ปฏิบัติประจำ	38	0
ไม่ปฏิบัติเล	ปฏิบัติบ่อย	ปฏิบัติบางค	ปฏิบัติบางค	ปฏิบัติบางค	ไม่ปฏิบัติเล	ไม่ปฏิบัติเล	17	2
ปฏิบัติประจำ	ปฏิบัติประจำ	ปฏิบัติประจำ	ไม่ปฏิบัติเล	ปฏิบัติบางค	ปฏิบัติบ่อย	ไม่ปฏิบัติเล	27	2
ปฏิบัติบางค	ปฏิบัติบางค	ปฏิบัติบางค	ปฏิบัติบางค	ปฏิบัติบางค	ปฏิบัติบางค	ไม่ปฏิบัติเล	21	2
ปฏิบัติประจำ	ปฏิบัติประจำ	ปฏิบัติบ่อย	ปฏิบัติบ่อย	ปฏิบัติบางค	ปฏิบัติบางค	ไม่ปฏิบัติเล	23	4
ปฏิบัติบ่อย	ปฏิบัติประจำ	ปฏิบัติบางค	ปฏิบัติบางค	ปฏิบัติประจำ	ไม่ปฏิบัติเล	ปฏิบัติประจำ	32	0
ปฏิบัติบางค	ปฏิบัติบางค	ปฏิบัติบางค	ปฏิบัติบางค	ปฏิบัติบ่อย	ปฏิบัติบ่อย	ไม่ปฏิบัติเล	19	2
ปฏิบัติบางค	ปฏิบัติบางค	ปฏิบัติบางค	ปฏิบัติบางค	ปฏิบัติประจำ	ปฏิบัติบ่อย	ไม่ปฏิบัติเล	29	0
ปฏิบัติบางค	ปฏิบัติบางค	ปฏิบัติบางค	ปฏิบัติบางค	ปฏิบัติบ่อย	ปฏิบัติบ่อย	ไม่ปฏิบัติเล	22	0
ปฏิบัติบ่อย	ปฏิบัติบ่อย	ปฏิบัติบางค	ปฏิบัติบ่อย	ปฏิบัติประจำ	ปฏิบัติบ่อย	ไม่ปฏิบัติเล	31	0
ปฏิบัติประจำ	ปฏิบัติประจำ	ปฏิบัติประจำ	ปฏิบัติบ่อย	ปฏิบัติประจำ	ปฏิบัติประจำ	ไม่ปฏิบัติเล	38	1
ปฏิบัติบางค	ปฏิบัติบางค	ปฏิบัติบางค	ปฏิบัติบางค	ปฏิบัติประจำ	ไม่ปฏิบัติเล	ปฏิบัติประจำ	26	2
ปฏิบัติบ่อย	ปฏิบัติบางค	ปฏิบัติบางค	ปฏิบัติบางค	ปฏิบัติบ่อย	ปฏิบัติบ่อย	ปฏิบัติประจำ	27	0
ปฏิบัติบางค	ปฏิบัติบางค	ปฏิบัติบางค	ปฏิบัติบางค	ปฏิบัติประจำ	ไม่ปฏิบัติเล	ไม่ปฏิบัติเล	19	1
ปฏิบัติบ่อย	ปฏิบัติบ่อย	ปฏิบัติบางค	ปฏิบัติบ่อย	ปฏิบัติบางค	ปฏิบัติบางค	ไม่ปฏิบัติเล	22	5
ปฏิบัติบ่อย	ปฏิบัติบ่อย	ปฏิบัติบ่อย	ปฏิบัติบ่อย	ปฏิบัติบางค	ปฏิบัติบางค	ไม่ปฏิบัติเล	20	0
ปฏิบัติบ่อย	ปฏิบัติบ่อย	ปฏิบัติบ่อย	ปฏิบัติบ่อย	ปฏิบัติบางค	ปฏิบัติบ่อย	ไม่ปฏิบัติเล	22	0
ปฏิบัติบ่อย	ปฏิบัติบ่อย	ปฏิบัติบ่อย	ปฏิบัติบ่อย	ปฏิบัติประจำ	ไม่ปฏิบัติเล	ปฏิบัติประจำ	37	5
ปฏิบัติบ่อย	ปฏิบัติบางค	ปฏิบัติบางค	ไม่ปฏิบัติเล	ปฏิบัติบ่อย	ไม่ปฏิบัติเล	ไม่ปฏิบัติเล	24	0
ปฏิบัติประจำ	ปฏิบัติประจำ	ปฏิบัติประจำ	ปฏิบัติประจำ	ปฏิบัติบ่อย	ปฏิบัติบ่อย	ไม่ปฏิบัติเล	36	6
ปฏิบัติบ่อย	ปฏิบัติบางค	ปฏิบัติบางค	ปฏิบัติบางค	ปฏิบัติประจำ	ปฏิบัติบ่อย	ไม่ปฏิบัติเล	25	1
ปฏิบัติบางค	ปฏิบัติประจำ	ปฏิบัติบ่อย	ปฏิบัติประจำ	ปฏิบัติประจำ	ปฏิบัติบ่อย	ไม่ปฏิบัติเล	35	2
ปฏิบัติบางค	ปฏิบัติบางค	ปฏิบัติบางค	ปฏิบัติบางค	ปฏิบัติประจำ	ปฏิบัติบ่อย	ไม่ปฏิบัติเล	19	5
ปฏิบัติบางค	ปฏิบัติบางค	ปฏิบัติบางค	ปฏิบัติบางค	ปฏิบัติบางค	ปฏิบัติบางค	ไม่ปฏิบัติเล	18	5
ปฏิบัติประจำ	ปฏิบัติบ่อย	ปฏิบัติบ่อย	ปฏิบัติบางค	ปฏิบัติบ่อย	ไม่ปฏิบัติเล	ไม่ปฏิบัติเล	32	2
ปฏิบัติประจำ	ปฏิบัติบ่อย	ปฏิบัติบางค	ปฏิบัติประจำ	ปฏิบัติประจำ	ปฏิบัติบ่อย	ปฏิบัติประจำ	39	0
ปฏิบัติบ่อย	ปฏิบัติบ่อย	ปฏิบัติบ่อย	ปฏิบัติบ่อย	ปฏิบัติบ่อย	ปฏิบัติบ่อย	ปฏิบัติประจำ	34	3
ปฏิบัติบางค	ปฏิบัติบางค	ปฏิบัติบางค	ปฏิบัติบางค	ปฏิบัติบางค	ปฏิบัติบางค	ไม่ปฏิบัติเล	18	0
ปฏิบัติบ่อย	ปฏิบัติบางค	ปฏิบัติบางค	ปฏิบัติบ่อย	ปฏิบัติบางค	ปฏิบัติบางค	ปฏิบัติประจำ	25	0
ปฏิบัติประจำ	ปฏิบัติประจำ	ปฏิบัติประจำ	ปฏิบัติประจำ	ปฏิบัติบ่อย	ปฏิบัติบ่อย	ปฏิบัติประจำ	43	2
ปฏิบัติบ่อย	ปฏิบัติบ่อย	ปฏิบัติบ่อย	ปฏิบัติบ่อย	ปฏิบัติบางค	ปฏิบัติบางค	ไม่ปฏิบัติเล	23	0
ปฏิบัติบ่อย	ปฏิบัติบ่อย	ปฏิบัติบ่อย	ปฏิบัติบางค	ปฏิบัติบางค	ปฏิบัติบางค	ไม่ปฏิบัติเล	22	3
ปฏิบัติบางค	ปฏิบัติบางค	ไม่ปฏิบัติเล	ปฏิบัติบางค	ปฏิบัติบางค	ไม่ปฏิบัติเล	ไม่ปฏิบัติเล	14	4
ปฏิบัติบ่อย	ปฏิบัติบ่อย	ปฏิบัติบ่อย	ปฏิบัติบ่อย	ปฏิบัติบ่อย	ปฏิบัติบ่อย	ปฏิบัติประจำ	36	0
ปฏิบัติบ่อย	ปฏิบัติประจำ	ปฏิบัติบ่อย	ปฏิบัติบางค	ปฏิบัติบางค	ปฏิบัติบางค	ไม่ปฏิบัติเล	22	7
ปฏิบัติบ่อย	ปฏิบัติบ่อย	ปฏิบัติบางค	ปฏิบัติบ่อย	ปฏิบัติบ่อย	ปฏิบัติบ่อย	ไม่ปฏิบัติเล	26	1
ปฏิบัติประจำ	ปฏิบัติบ่อย	ปฏิบัติบางค	ปฏิบัติบ่อย	ปฏิบัติบ่อย	ปฏิบัติประจำ	ปฏิบัติประจำ	32	0
ปฏิบัติบางค	ปฏิบัติบ่อย	ปฏิบัติบ่อย	ปฏิบัติบ่อย	ปฏิบัติบ่อย	ปฏิบัติบ่อย	ไม่ปฏิบัติเล	23	1
ปฏิบัติบางค	ปฏิบัติบางค	ปฏิบัติบางค	ปฏิบัติบางค	ปฏิบัติประจำ	ปฏิบัติบ่อย	ไม่ปฏิบัติเล	28	3
ปฏิบัติบางค	ปฏิบัติบางค	ปฏิบัติบางค	ปฏิบัติบ่อย	ปฏิบัติประจำ	ปฏิบัติบ่อย	ปฏิบัติประจำ	34	2
ปฏิบัติประจำ	ปฏิบัติบ่อย	ไม่ปฏิบัติเล	ปฏิบัติบ่อย	ปฏิบัติบ่อย	ไม่ปฏิบัติเล	ไม่ปฏิบัติเล	28	0
ปฏิบัติบางค	ปฏิบัติบางค	ปฏิบัติบางค	ปฏิบัติบางค	ปฏิบัติบางค	ปฏิบัติบางค	ไม่ปฏิบัติเล	15	3
ไม่ปฏิบัติเล	ปฏิบัติบ่อย	ปฏิบัติบางค	ปฏิบัติบ่อย	ปฏิบัติประจำ	ปฏิบัติบ่อย	ไม่ปฏิบัติเล	27	1
ปฏิบัติประจำ	ปฏิบัติประจำ	ปฏิบัติประจำ	ปฏิบัติประจำ	ปฏิบัติประจำ	ปฏิบัติประจำ	ปฏิบัติประจำ	42	0
ปฏิบัติบ่อย	ปฏิบัติบ่อย	ปฏิบัติบางค	ปฏิบัติบางค	ปฏิบัติประจำ	ปฏิบัติประจำ	ไม่ปฏิบัติเล	34	0
ปฏิบัติบางค	ปฏิบัติบางค	ปฏิบัติบางค	ปฏิบัติบางค	ปฏิบัติบางค	ปฏิบัติบางค	ไม่ปฏิบัติเล	13	3
ปฏิบัติบ่อย	ปฏิบัติประจำ	ปฏิบัติบ่อย	ปฏิบัติประจำ	ปฏิบัติบางค	ไม่ปฏิบัติเล	ปฏิบัติประจำ	34	4
ปฏิบัติประจำ	ปฏิบัติประจำ	ปฏิบัติประจำ	ปฏิบัติประจำ	ปฏิบัติประจำ	ปฏิบัติประจำ	ปฏิบัติประจำ	41	0

ปฏิบัติบางค	ปฏิบัติประจำ	ปฏิบัติบางค	ปฏิบัติประจำ	ปฏิบัติประจำ	ปฏิบัติประจำ	ไม่ปฏิบัติเล	36	3
ปฏิบัติประจำ	ปฏิบัติบางค	ปฏิบัติบางค	ปฏิบัติบางค	ปฏิบัติบ่อย	ปฏิบัติบ่อย	ไม่ปฏิบัติเล	28	0
ปฏิบัติบ่อย	ปฏิบัติบ่อย	ปฏิบัติบางค	ปฏิบัติบางค	ปฏิบัติประจำ	ปฏิบัติบ่อย	ไม่ปฏิบัติเล	20	0
ปฏิบัติบางค	ปฏิบัติบางค	ปฏิบัติบางค	ไม่ปฏิบัติเล	ปฏิบัติบ่อย	ปฏิบัติบ่อย	ไม่ปฏิบัติเล	18	0
ปฏิบัติประจำ	ปฏิบัติบ่อย	ปฏิบัติบ่อย	ปฏิบัติบ่อย	ปฏิบัติบ่อย	ปฏิบัติบ่อย	ปฏิบัติประจำ	34	2
ปฏิบัติบ่อย	ปฏิบัติประจำ	ปฏิบัติบางค	ปฏิบัติบางค	ปฏิบัติบางค	ไม่ปฏิบัติเล	ไม่ปฏิบัติเล	20	0
ปฏิบัติประจำ	ปฏิบัติประจำ	ปฏิบัติบางค	ปฏิบัติบางค	ปฏิบัติประจำ	ปฏิบัติบ่อย	ไม่ปฏิบัติเล	32	2
ปฏิบัติบ่อย	ปฏิบัติประจำ	ปฏิบัติบ่อย	ปฏิบัติบางค	ปฏิบัติบ่อย	ปฏิบัติบ่อย	ไม่ปฏิบัติเล	27	0
ปฏิบัติประจำ	ปฏิบัติประจำ	ปฏิบัติประจำ	ปฏิบัติบ่อย	ปฏิบัติบ่อย	ปฏิบัติประจำ	ไม่ปฏิบัติเล	37	3
ปฏิบัติบ่อย	ปฏิบัติบางค	ปฏิบัติบางค	ปฏิบัติบางค	ปฏิบัติประจำ	ปฏิบัติบ่อย	ปฏิบัติประจำ	32	6
ปฏิบัติบ่อย	ปฏิบัติบ่อย	ปฏิบัติประจำ	ปฏิบัติบางค	ปฏิบัติประจำ	ปฏิบัติบ่อย	ไม่ปฏิบัติเล	31	0
ปฏิบัติบ่อย	ปฏิบัติบ่อย	ปฏิบัติบางค	ปฏิบัติบางค	ปฏิบัติประจำ	ปฏิบัติบ่อย	ไม่ปฏิบัติเล	28	0
ไม่ปฏิบัติเล	ปฏิบัติบางค	ปฏิบัติบางค	ไม่ปฏิบัติเล	ปฏิบัติประจำ	ปฏิบัติบ่อย	ไม่ปฏิบัติเล	22	2
ปฏิบัติบางค	ปฏิบัติบ่อย	ปฏิบัติบางค	ไม่ปฏิบัติเล	ปฏิบัติบ่อย	ปฏิบัติบ่อย	ไม่ปฏิบัติเล	20	2
ปฏิบัติประจำ	ปฏิบัติประจำ	ปฏิบัติบางค	ปฏิบัติบางค	ปฏิบัติบ่อย	ปฏิบัติบ่อย	ไม่ปฏิบัติเล	29	2
ปฏิบัติบางค	ปฏิบัติบางค	ไม่ปฏิบัติเล	ไม่ปฏิบัติเล	ปฏิบัติบ่อย	ปฏิบัติบ่อย	ไม่ปฏิบัติเล	17	2
ปฏิบัติบ่อย	ปฏิบัติบ่อย	ปฏิบัติบ่อย	ปฏิบัติบ่อย	ปฏิบัติบ่อย	ไม่ปฏิบัติเล	ไม่ปฏิบัติเล	31	7
ปฏิบัติบางค	ปฏิบัติบางค	ไม่ปฏิบัติเล	ปฏิบัติบางค	ไม่ปฏิบัติเล	ปฏิบัติบางค	ไม่ปฏิบัติเล	19	5
ปฏิบัติบางค	ปฏิบัติบ่อย	ปฏิบัติบางค	ปฏิบัติบางค	ปฏิบัติบางค	ไม่ปฏิบัติเล	ไม่ปฏิบัติเล	18	4
ปฏิบัติบ่อย	ปฏิบัติบางค	ปฏิบัติบางค	ไม่ปฏิบัติเล	ปฏิบัติประจำ	ปฏิบัติบ่อย	ไม่ปฏิบัติเล	29	6
ปฏิบัติประจำ	ปฏิบัติประจำ	ปฏิบัติประจำ	ปฏิบัติประจำ	ปฏิบัติบ่อย	ปฏิบัติประจำ	ไม่ปฏิบัติเล	37	4
ปฏิบัติบ่อย	ปฏิบัติบางค	ปฏิบัติบางค	ปฏิบัติบางค	ปฏิบัติบางค	ปฏิบัติบ่อย	ไม่ปฏิบัติเล	18	5
ปฏิบัติบางค	ปฏิบัติบางค	ปฏิบัติบางค	ปฏิบัติบางค	ปฏิบัติบ่อย	ปฏิบัติบางค	ไม่ปฏิบัติเล	23	3
ปฏิบัติบ่อย	ปฏิบัติบ่อย	ปฏิบัติบ่อย	ปฏิบัติบ่อย	ปฏิบัติบ่อย	ปฏิบัติบ่อย	ไม่ปฏิบัติเล	30	3
ปฏิบัติบ่อย	ไม่ปฏิบัติเล	ไม่ปฏิบัติเล	ไม่ปฏิบัติเล	ไม่ปฏิบัติเล	ไม่ปฏิบัติเล	ไม่ปฏิบัติเล	18	1
ปฏิบัติบ่อย	ปฏิบัติบางค	ปฏิบัติบางค	ปฏิบัติบางค	ปฏิบัติบางค	ไม่ปฏิบัติเล	ปฏิบัติประจำ	25	3
ปฏิบัติบ่อย	ปฏิบัติประจำ	ปฏิบัติประจำ	ปฏิบัติบางค	ปฏิบัติบ่อย	ปฏิบัติบางค	ไม่ปฏิบัติเล	27	3
ปฏิบัติบางค	ปฏิบัติบางค	ปฏิบัติบางค	ปฏิบัติบางค	ปฏิบัติบางค	ไม่ปฏิบัติเล	ไม่ปฏิบัติเล	24	9
ปฏิบัติประจำ	ปฏิบัติบางค	ไม่ปฏิบัติเล	ปฏิบัติบางค	ปฏิบัติประจำ	ปฏิบัติบ่อย	ไม่ปฏิบัติเล	30	3
ปฏิบัติบางค	ปฏิบัติบางค	ปฏิบัติบางค	ปฏิบัติบางค	ปฏิบัติบางค	ปฏิบัติบางค	ไม่ปฏิบัติเล	14	0
ปฏิบัติบ่อย	ปฏิบัติบางค	ปฏิบัติบางค	ปฏิบัติบางค	ปฏิบัติบ่อย	ไม่ปฏิบัติเล	ปฏิบัติประจำ	21	0
ปฏิบัติบ่อย	ปฏิบัติบ่อย	ปฏิบัติบ่อย	ไม่ปฏิบัติเล	ปฏิบัติบ่อย	ไม่ปฏิบัติเล	ไม่ปฏิบัติเล	16	1
ปฏิบัติบ่อย	ไม่ปฏิบัติเล	ปฏิบัติบางค	ปฏิบัติบางค	ปฏิบัติประจำ	ไม่ปฏิบัติเล	ไม่ปฏิบัติเล	22	0
ปฏิบัติบางค	ปฏิบัติบางค	ปฏิบัติบางค	ปฏิบัติบ่อย	ปฏิบัติประจำ	ปฏิบัติบางค	ไม่ปฏิบัติเล	27	3
ปฏิบัติบ่อย	ปฏิบัติบางค	ปฏิบัติบ่อย	ปฏิบัติบางค	ไม่ปฏิบัติเล	ไม่ปฏิบัติเล	ไม่ปฏิบัติเล	14	4
ปฏิบัติบางค	ปฏิบัติบ่อย	ปฏิบัติบางค	ปฏิบัติบางค	ปฏิบัติประจำ	ปฏิบัติบางค	ไม่ปฏิบัติเล	23	0
ปฏิบัติบางค	ปฏิบัติบางค	ปฏิบัติบางค	ปฏิบัติบ่อย	ปฏิบัติบ่อย	ปฏิบัติบางค	ปฏิบัติประจำ	31	0
ปฏิบัติบางค	ไม่ปฏิบัติเล	ปฏิบัติบางค	ไม่ปฏิบัติเล	ปฏิบัติบางค	ไม่ปฏิบัติเล	ไม่ปฏิบัติเล	21	2
ปฏิบัติบางค	ปฏิบัติบางค	ปฏิบัติบางค	ปฏิบัติบางค	ปฏิบัติประจำ	ปฏิบัติบางค	ไม่ปฏิบัติเล	22	0
ปฏิบัติประจำ	ปฏิบัติบ่อย	ปฏิบัติบางค	ปฏิบัติบางค	ปฏิบัติบ่อย	ปฏิบัติบ่อย	ไม่ปฏิบัติเล	28	0
ปฏิบัติประจำ	ปฏิบัติประจำ	ปฏิบัติประจำ	ไม่ปฏิบัติเล	ปฏิบัติบางค	ปฏิบัติบ่อย	ไม่ปฏิบัติเล	28	7
ปฏิบัติประจำ	ปฏิบัติบ่อย	ไม่ปฏิบัติเล	ปฏิบัติบางค	ปฏิบัติประจำ	ปฏิบัติบางค	ไม่ปฏิบัติเล	32	0
ปฏิบัติบ่อย	ปฏิบัติประจำ	ปฏิบัติบ่อย	ปฏิบัติบ่อย	ปฏิบัติบ่อย	ปฏิบัติประจำ	ปฏิบัติประจำ	38	2
ปฏิบัติประจำ	ปฏิบัติบ่อย	ปฏิบัติบ่อย	ปฏิบัติบางค	ปฏิบัติบางค	ปฏิบัติบ่อย	ไม่ปฏิบัติเล	22	1
ปฏิบัติบ่อย	ปฏิบัติประจำ	ปฏิบัติบางค	ปฏิบัติบางค	ปฏิบัติประจำ	ปฏิบัติบ่อย	ไม่ปฏิบัติเล	33	0
ปฏิบัติบ่อย	ปฏิบัติบางค	ปฏิบัติบางค	ปฏิบัติบางค	ปฏิบัติบางค	ปฏิบัติบางค	ไม่ปฏิบัติเล	20	0
ปฏิบัติบางค	ปฏิบัติบางค	ปฏิบัติบางค	ปฏิบัติบ่อย	ปฏิบัติบ่อย	ปฏิบัติบางค	ปฏิบัติประจำ	26	5
ปฏิบัติบ่อย	ปฏิบัติบ่อย	ปฏิบัติบ่อย	ปฏิบัติบางค	ปฏิบัติบ่อย	ไม่ปฏิบัติเล	ไม่ปฏิบัติเล	26	3
ปฏิบัติประจำ	ไม่ปฏิบัติเล	ไม่ปฏิบัติเล	ปฏิบัติบางค	ปฏิบัติประจำ	ปฏิบัติประจำ	ไม่ปฏิบัติเล	31	2
ปฏิบัติบ่อย	ปฏิบัติบ่อย	ปฏิบัติบ่อย	ปฏิบัติบางค	ปฏิบัติบางค	ไม่ปฏิบัติเล	ไม่ปฏิบัติเล	25	0
ปฏิบัติประจำ	ปฏิบัติบางค	ไม่ปฏิบัติเล	ปฏิบัติบางค	ปฏิบัติประจำ	ปฏิบัติประจำ	ไม่ปฏิบัติเล	23	0

ปฏิบัติบางค	ปฏิบัติบางค	ปฏิบัติบางค	ไม่ปฏิบัติเล	ปฏิบัติประจำ	ปฏิบัติบางค	ไม่ปฏิบัติเล	21	2
ปฏิบัติประจำ	ปฏิบัติบ่อย	ปฏิบัติบ่อย	ปฏิบัติบางค	ปฏิบัติประจำ	ปฏิบัติบางค	ไม่ปฏิบัติเล	28	0
ปฏิบัติบางค	ไม่ปฏิบัติเล	ไม่ปฏิบัติเล	ปฏิบัติบางค	ไม่ปฏิบัติเล	ไม่ปฏิบัติเล	ไม่ปฏิบัติเล	15	4
ปฏิบัติบ่อย	ปฏิบัติบ่อย	ปฏิบัติบ่อย	ปฏิบัติบ่อย	ปฏิบัติบ่อย	ปฏิบัติบ่อย	ไม่ปฏิบัติเล	30	0
ปฏิบัติบางค	ปฏิบัติประจำ	ปฏิบัติบางค	ปฏิบัติบางค	ปฏิบัติประจำ	ไม่ปฏิบัติเล	ไม่ปฏิบัติเล	29	0
ปฏิบัติประจำ	ปฏิบัติบางค	ปฏิบัติบางค	ไม่ปฏิบัติเล	ปฏิบัติประจำ	ไม่ปฏิบัติเล	ไม่ปฏิบัติเล	25	0
ปฏิบัติบางค	ปฏิบัติบางค	ปฏิบัติบางค	ปฏิบัติบ่อย	ปฏิบัติบ่อย	ไม่ปฏิบัติเล	ไม่ปฏิบัติเล	23	0
ปฏิบัติบ่อย	ปฏิบัติบ่อย	ปฏิบัติบางค	ไม่ปฏิบัติเล	ปฏิบัติบ่อย	ปฏิบัติบางค	ไม่ปฏิบัติเล	14	0
ปฏิบัติบางค	ไม่ปฏิบัติเล	ไม่ปฏิบัติเล	ไม่ปฏิบัติเล	ปฏิบัติบางค	ไม่ปฏิบัติเล	ไม่ปฏิบัติเล	14	5
ปฏิบัติบางค	ไม่ปฏิบัติเล	ปฏิบัติบางค	ปฏิบัติบางค	ปฏิบัติบ่อย	ปฏิบัติบางค	ไม่ปฏิบัติเล	23	0
ปฏิบัติบางค	ปฏิบัติบางค	ปฏิบัติบางค	ปฏิบัติบางค	ปฏิบัติบ่อย	ไม่ปฏิบัติเล	ปฏิบัติประจำ	29	2
ปฏิบัติบางค	ปฏิบัติบางค	ปฏิบัติบางค	ปฏิบัติบางค	ปฏิบัติประจำ	ไม่ปฏิบัติเล	ไม่ปฏิบัติเล	21	0
ปฏิบัติประจำ	ปฏิบัติบางค	ไม่ปฏิบัติเล	ปฏิบัติประจำ	ปฏิบัติบ่อย	ปฏิบัติบางค	ไม่ปฏิบัติเล	30	0
ปฏิบัติบางค	ปฏิบัติบ่อย	ปฏิบัติบางค	ปฏิบัติบางค	ไม่ปฏิบัติเล	ไม่ปฏิบัติเล	ไม่ปฏิบัติเล	20	1
ปฏิบัติประจำ	ปฏิบัติบางค	ปฏิบัติบางค	ปฏิบัติประจำ	ไม่ปฏิบัติเล	ปฏิบัติบางค	ไม่ปฏิบัติเล	24	3
ปฏิบัติบางค	ปฏิบัติบางค	ปฏิบัติบางค	ปฏิบัติบางค	ปฏิบัติบางค	ไม่ปฏิบัติเล	ไม่ปฏิบัติเล	13	2
ปฏิบัติบางค	ปฏิบัติบางค	ปฏิบัติบางค	ปฏิบัติบางค	ปฏิบัติประจำ	ไม่ปฏิบัติเล	ไม่ปฏิบัติเล	23	0
ปฏิบัติบางค	ปฏิบัติบางค	ปฏิบัติบ่อย	ปฏิบัติบางค	ปฏิบัติบางค	ปฏิบัติบางค	ไม่ปฏิบัติเล	19	2
ปฏิบัติบางค	ปฏิบัติบางค	ปฏิบัติบางค	ปฏิบัติบ่อย	ปฏิบัติประจำ	ปฏิบัติบางค	ไม่ปฏิบัติเล	24	2
ปฏิบัติบางค	ปฏิบัติบางค	ปฏิบัติบางค	ปฏิบัติบางค	ปฏิบัติบางค	ปฏิบัติบางค	ไม่ปฏิบัติเล	15	2
ปฏิบัติบ่อย	ปฏิบัติบางค	ไม่ปฏิบัติเล	ปฏิบัติบางค	ปฏิบัติประจำ	ปฏิบัติบ่อย	ปฏิบัติบางค	27	0
ปฏิบัติบ่อย	ปฏิบัติบางค	ปฏิบัติบางค	ปฏิบัติบางค	ปฏิบัติบางค	ปฏิบัติบางค	ไม่ปฏิบัติเล	21	0
ปฏิบัติบ่อย	ปฏิบัติบ่อย	ปฏิบัติบางค	ปฏิบัติบางค	ปฏิบัติบางค	ปฏิบัติบางค	ไม่ปฏิบัติเล	19	0
ปฏิบัติบ่อย	ปฏิบัติบ่อย	ปฏิบัติบ่อย	ปฏิบัติบ่อย	ปฏิบัติประจำ	ปฏิบัติบางค	ไม่ปฏิบัติเล	32	3
ปฏิบัติบ่อย	ปฏิบัติบ่อย	ปฏิบัติบ่อย	ปฏิบัติบางค	ปฏิบัติประจำ	ปฏิบัติบ่อย	ไม่ปฏิบัติเล	31	5
ปฏิบัติบางค	ปฏิบัติบางค	ปฏิบัติบ่อย	ปฏิบัติบ่อย	ปฏิบัติประจำ	ปฏิบัติบางค	ไม่ปฏิบัติเล	19	2
ปฏิบัติบางค	ปฏิบัติบางค	ปฏิบัติบางค	ปฏิบัติบ่อย	ปฏิบัติบ่อย	ปฏิบัติบ่อย	ปฏิบัติประจำ	27	2
ปฏิบัติบ่อย	ปฏิบัติบ่อย	ปฏิบัติบ่อย	ปฏิบัติบ่อย	ปฏิบัติบ่อย	ปฏิบัติบางค	ไม่ปฏิบัติเล	19	0
ปฏิบัติบางค	ปฏิบัติประจำ	ปฏิบัติบางค	ไม่ปฏิบัติเล	ปฏิบัติบางค	ปฏิบัติบางค	ไม่ปฏิบัติเล	23	0
ปฏิบัติบ่อย	ปฏิบัติบางค	ปฏิบัติบางค	ปฏิบัติบางค	ปฏิบัติบางค	ปฏิบัติบางค	ไม่ปฏิบัติเล	23	3
ปฏิบัติประจำ	ปฏิบัติบ่อย	ปฏิบัติบางค	ปฏิบัติบางค	ปฏิบัติประจำ	ปฏิบัติบางค	ไม่ปฏิบัติเล	26	0
ปฏิบัติประจำ	ปฏิบัติบ่อย	ปฏิบัติบ่อย	ปฏิบัติบ่อย	ปฏิบัติบ่อย	ปฏิบัติบ่อย	ไม่ปฏิบัติเล	32	4
ปฏิบัติบ่อย	ปฏิบัติบ่อย	ปฏิบัติบ่อย	ปฏิบัติบ่อย	ปฏิบัติบางค	ปฏิบัติบ่อย	ไม่ปฏิบัติเล	27	0
ปฏิบัติบางค	ปฏิบัติบางค	ไม่ปฏิบัติเล	ปฏิบัติบางค	ปฏิบัติบางค	ปฏิบัติบางค	ไม่ปฏิบัติเล	17	0
ปฏิบัติบางค	ปฏิบัติบางค	ปฏิบัติบางค	ปฏิบัติบ่อย	ปฏิบัติบางค	ปฏิบัติบางค	ปฏิบัติประจำ	27	0
ปฏิบัติบ่อย	ปฏิบัติบ่อย	ปฏิบัติบ่อย	ปฏิบัติบ่อย	ปฏิบัติบ่อย	ปฏิบัติบางค	ไม่ปฏิบัติเล	33	3
ปฏิบัติบ่อย	ปฏิบัติบ่อย	ปฏิบัติบ่อย	ปฏิบัติบ่อย	ปฏิบัติบ่อย	ปฏิบัติบางค	ไม่ปฏิบัติเล	19	0
ปฏิบัติประจำ	ปฏิบัติบ่อย	ปฏิบัติบางค	ปฏิบัติประจำ	ปฏิบัติประจำ	ปฏิบัติบางค	ไม่ปฏิบัติเล	29	3
ปฏิบัติบ่อย	ปฏิบัติบ่อย	ปฏิบัติบางค	ปฏิบัติบางค	ปฏิบัติประจำ	ปฏิบัติบางค	ปฏิบัติประจำ	31	0
ปฏิบัติบางค	ปฏิบัติบางค	ปฏิบัติบางค	ปฏิบัติบางค	ปฏิบัติบ่อย	ปฏิบัติบางค	ไม่ปฏิบัติเล	21	0
ปฏิบัติบ่อย	ปฏิบัติบางค	ปฏิบัติบางค	ปฏิบัติบางค	ปฏิบัติบ่อย	ปฏิบัติบางค	ไม่ปฏิบัติเล	22	5
ปฏิบัติประจำ	ปฏิบัติประจำ	ปฏิบัติบางค	ปฏิบัติบ่อย	ปฏิบัติประจำ	ปฏิบัติประจำ	ไม่ปฏิบัติเล	33	0
ปฏิบัติบางค	ปฏิบัติบางค	ปฏิบัติบางค	ปฏิบัติบ่อย	ปฏิบัติประจำ	ไม่ปฏิบัติเล	ปฏิบัติประจำ	32	0
ปฏิบัติบางค	ปฏิบัติบางค	ปฏิบัติบางค	ปฏิบัติบางค	ปฏิบัติบางค	ปฏิบัติบางค	ปฏิบัติประจำ	27	0
ปฏิบัติบ่อย	ปฏิบัติบางค	ปฏิบัติบ่อย	ปฏิบัติประจำ	ปฏิบัติบางค	ปฏิบัติบางค	ปฏิบัติบางค	28	0
ปฏิบัติบ่อย	ปฏิบัติบางค	ปฏิบัติบางค	ปฏิบัติบางค	ปฏิบัติบางค	ปฏิบัติบางค	ไม่ปฏิบัติเล	24	6
ปฏิบัติบางค	ปฏิบัติบางค	ปฏิบัติบางค	ปฏิบัติบ่อย	ปฏิบัติบ่อย	ปฏิบัติบางค	ไม่ปฏิบัติเล	19	3
ปฏิบัติบางค	ปฏิบัติบางค	ปฏิบัติบางค	ปฏิบัติบางค	ปฏิบัติบางค	ปฏิบัติบางค	ไม่ปฏิบัติเล	23	0
ปฏิบัติประจำ	ปฏิบัติประจำ	ปฏิบัติบ่อย	ปฏิบัติบ่อย	ปฏิบัติประจำ	ปฏิบัติบางค	ปฏิบัติประจำ	33	0
ปฏิบัติประจำ	ปฏิบัติประจำ	ปฏิบัติประจำ	ปฏิบัติประจำ	ปฏิบัติประจำ	ปฏิบัติประจำ	ปฏิบัติประจำ	40	0
ปฏิบัติประจำ	ปฏิบัติประจำ	ปฏิบัติประจำ	ปฏิบัติประจำ	ปฏิบัติบางค	ปฏิบัติประจำ	ไม่ปฏิบัติเล	35	4

ปฏิบัติบางค	ปฏิบัติบางค	ปฏิบัติบางค	ปฏิบัติบางค	ปฏิบัติบางค	ปฏิบัติบางค	ไม่ปฏิบัติเล	24	0
ปฏิบัติบ่อย	ปฏิบัติบ่อย	ปฏิบัติบ่อย	ปฏิบัติประจำ	ปฏิบัติประจำ	ปฏิบัติบางค	ไม่ปฏิบัติเล	32	0
ปฏิบัติบางค	ปฏิบัติประจำ	ปฏิบัติบางค	ปฏิบัติบางค	ปฏิบัติประจำ	ปฏิบัติบางค	ไม่ปฏิบัติเล	27	0
ปฏิบัติบางค	ปฏิบัติบ่อย	ปฏิบัติบางค	ปฏิบัติบางค	ปฏิบัติบางค	ปฏิบัติบางค	ไม่ปฏิบัติเล	19	0
ปฏิบัติประจำ	ปฏิบัติประจำ	ปฏิบัติประจำ	ปฏิบัติประจำ	ปฏิบัติประจำ	ปฏิบัติบางค	ไม่ปฏิบัติเล	32	2
ปฏิบัติบางค	ปฏิบัติบางค	ปฏิบัติบางค	ปฏิบัติบางค	ปฏิบัติประจำ	ไม่ปฏิบัติเล	ไม่ปฏิบัติเล	23	3
ปฏิบัติบ่อย	ปฏิบัติบ่อย	ปฏิบัติบางค	ปฏิบัติบ่อย	ปฏิบัติบ่อย	ไม่ปฏิบัติเล	ไม่ปฏิบัติเล	24	4
ปฏิบัติบ่อย	ปฏิบัติบ่อย	ปฏิบัติบ่อย	ไม่ปฏิบัติเล	ปฏิบัติบ่อย	ไม่ปฏิบัติเล	ไม่ปฏิบัติเล	22	0
ปฏิบัติประจำ	ปฏิบัติบ่อย	ปฏิบัติบางค	ปฏิบัติบางค	ปฏิบัติบางค	ปฏิบัติประจำ	ไม่ปฏิบัติเล	20	0
ปฏิบัติประจำ	ปฏิบัติประจำ	ปฏิบัติบางค	ปฏิบัติบางค	ปฏิบัติประจำ	ไม่ปฏิบัติเล	ไม่ปฏิบัติเล	25	0
ปฏิบัติบ่อย	ปฏิบัติบ่อย	ปฏิบัติบ่อย	ปฏิบัติบ่อย	ปฏิบัติบ่อย	ปฏิบัติบ่อย	ไม่ปฏิบัติเล	28	5
ปฏิบัติบ่อย	ปฏิบัติบ่อย	ปฏิบัติบ่อย	ปฏิบัติบ่อย	ปฏิบัติประจำ	ปฏิบัติประจำ	ปฏิบัติประจำ	38	3
ปฏิบัติประจำ	ปฏิบัติประจำ	ปฏิบัติประจำ	ปฏิบัติประจำ	ปฏิบัติบ่อย	ปฏิบัติประจำ	ปฏิบัติประจำ	39	0
ปฏิบัติบางค	ปฏิบัติบ่อย	ปฏิบัติบางค	ปฏิบัติบ่อย	ปฏิบัติบ่อย	ปฏิบัติบ่อย	ไม่ปฏิบัติเล	24	2
ปฏิบัติประจำ	ปฏิบัติบ่อย	ปฏิบัติบ่อย	ปฏิบัติประจำ	ปฏิบัติบ่อย	ปฏิบัติบ่อย	ปฏิบัติประจำ	33	0
ปฏิบัติบ่อย	ปฏิบัติบางค	ปฏิบัติบ่อย	ปฏิบัติบ่อย	ปฏิบัติบ่อย	ปฏิบัติบ่อย	ไม่ปฏิบัติเล	25	0
ปฏิบัติบ่อย	ปฏิบัติบ่อย	ปฏิบัติบ่อย	ปฏิบัติบางค	ปฏิบัติบางค	ไม่ปฏิบัติเล	ไม่ปฏิบัติเล	20	4
ปฏิบัติบางค	ปฏิบัติบ่อย	ปฏิบัติบ่อย	ปฏิบัติบางค	ปฏิบัติบางค	ปฏิบัติบางค	ไม่ปฏิบัติเล	23	1
ปฏิบัติบ่อย	ปฏิบัติบ่อย	ปฏิบัติประจำ	ปฏิบัติบ่อย	ปฏิบัติประจำ	ปฏิบัติประจำ	ปฏิบัติประจำ	39	3
ปฏิบัติประจำ	ปฏิบัติประจำ	ปฏิบัติบ่อย	ปฏิบัติบางค	ปฏิบัติบ่อย	ปฏิบัติบ่อย	ไม่ปฏิบัติเล	27	0
ปฏิบัติบางค	ปฏิบัติบ่อย	ไม่ปฏิบัติเล	ไม่ปฏิบัติเล	ปฏิบัติประจำ	ปฏิบัติบ่อย	ไม่ปฏิบัติเล	24	0
ปฏิบัติบ่อย	ปฏิบัติประจำ	ปฏิบัติประจำ	ปฏิบัติบ่อย	ปฏิบัติประจำ	ปฏิบัติประจำ	ปฏิบัติประจำ	37	0
ปฏิบัติบางค	ปฏิบัติบางค	ปฏิบัติบางค	ปฏิบัติบางค	ปฏิบัติบ่อย	ปฏิบัติบ่อย	ไม่ปฏิบัติเล	17	0
ปฏิบัติบ่อย	ปฏิบัติบางค	ปฏิบัติบางค	ปฏิบัติบ่อย	ปฏิบัติประจำ	ปฏิบัติบางค	ปฏิบัติประจำ	33	2
ปฏิบัติบางค	ปฏิบัติประจำ	ปฏิบัติบางค	ปฏิบัติบางค	ปฏิบัติบ่อย	ปฏิบัติบ่อย	ไม่ปฏิบัติเล	17	1
ปฏิบัติบ่อย	ปฏิบัติบ่อย	ปฏิบัติบางค	ปฏิบัติบางค	ปฏิบัติบ่อย	ปฏิบัติบ่อย	ปฏิบัติประจำ	26	0
ปฏิบัติประจำ	ปฏิบัติบางค	ปฏิบัติบางค	ปฏิบัติบางค	ปฏิบัติประจำ	ปฏิบัติบางค	ไม่ปฏิบัติเล	34	0
ปฏิบัติบ่อย	ปฏิบัติบ่อย	ปฏิบัติบ่อย	ปฏิบัติบ่อย	ปฏิบัติประจำ	ปฏิบัติบ่อย	ปฏิบัติประจำ	33	6
ปฏิบัติบ่อย	ปฏิบัติประจำ	ปฏิบัติบ่อย	ปฏิบัติบ่อย	ปฏิบัติบ่อย	ปฏิบัติบ่อย	ปฏิบัติประจำ	36	0
ปฏิบัติประจำ	ปฏิบัติประจำ	ปฏิบัติบ่อย	ปฏิบัติบ่อย	ปฏิบัติบ่อย	ปฏิบัติประจำ	ปฏิบัติประจำ	41	0
ปฏิบัติประจำ	ปฏิบัติประจำ	ปฏิบัติประจำ	ปฏิบัติบ่อย	ปฏิบัติประจำ	ปฏิบัติประจำ	ไม่ปฏิบัติเล	38	0
ปฏิบัติบ่อย	ปฏิบัติบางค	ปฏิบัติบ่อย	ปฏิบัติบ่อย	ปฏิบัติประจำ	ปฏิบัติบางค	ปฏิบัติประจำ	31	1
ปฏิบัติประจำ	ปฏิบัติประจำ	ปฏิบัติประจำ	ปฏิบัติประจำ	ปฏิบัติประจำ	ปฏิบัติประจำ	ปฏิบัติประจำ	42	0
ปฏิบัติบางค	ปฏิบัติบางค	ปฏิบัติบางค	ปฏิบัติบ่อย	ปฏิบัติประจำ	ปฏิบัติบางค	ไม่ปฏิบัติเล	29	0
ปฏิบัติประจำ	ปฏิบัติบ่อย	ไม่ปฏิบัติเล	ไม่ปฏิบัติเล	ปฏิบัติบางค	ปฏิบัติบางค	ปฏิบัติประจำ	32	0
ปฏิบัติบางค	ปฏิบัติบ่อย	ปฏิบัติบางค	ปฏิบัติบางค	ปฏิบัติประจำ	ปฏิบัติบ่อย	ไม่ปฏิบัติเล	22	0
ปฏิบัติบางค	ปฏิบัติบางค	ปฏิบัติบางค	ปฏิบัติบางค	ปฏิบัติบางค	ปฏิบัติบางค	ไม่ปฏิบัติเล	19	0
ปฏิบัติประจำ	ปฏิบัติประจำ	ปฏิบัติบ่อย	ปฏิบัติบางค	ปฏิบัติบ่อย	ปฏิบัติบางค	ไม่ปฏิบัติเล	29	3
ปฏิบัติประจำ	ปฏิบัติประจำ	ปฏิบัติประจำ	ปฏิบัติประจำ	ปฏิบัติประจำ	ปฏิบัติบ่อย	ปฏิบัติประจำ	38	5
ไม่ปฏิบัติเล	ปฏิบัติบางค	ปฏิบัติบางค	ปฏิบัติบ่อย	ปฏิบัติประจำ	ไม่ปฏิบัติเล	ปฏิบัติประจำ	26	0
ปฏิบัติบ่อย	ปฏิบัติประจำ	ปฏิบัติบางค	ปฏิบัติบ่อย	ปฏิบัติบ่อย	ปฏิบัติบ่อย	ปฏิบัติประจำ	36	0
ปฏิบัติบางค	ปฏิบัติบางค	ปฏิบัติบ่อย	ปฏิบัติบางค	ปฏิบัติบ่อย	ปฏิบัติบ่อย	ปฏิบัติประจำ	24	2
ปฏิบัติบางค	ปฏิบัติบางค	ปฏิบัติบางค	ปฏิบัติบางค	ปฏิบัติประจำ	ปฏิบัติบางค	ไม่ปฏิบัติเล	23	0
ปฏิบัติบางค	ปฏิบัติบ่อย	ปฏิบัติบางค	ปฏิบัติบางค	ปฏิบัติประจำ	ปฏิบัติบ่อย	ไม่ปฏิบัติเล	23	0
ปฏิบัติบางค	ปฏิบัติบ่อย	ปฏิบัติบ่อย	ไม่ปฏิบัติเล	ปฏิบัติประจำ	ปฏิบัติบ่อย	ไม่ปฏิบัติเล	23	6
ปฏิบัติบางค	ปฏิบัติบ่อย	ปฏิบัติบางค	ปฏิบัติบางค	ปฏิบัติประจำ	ปฏิบัติบ่อย	ปฏิบัติประจำ	32	0
ปฏิบัติบ่อย	ปฏิบัติบ่อย	ปฏิบัติประจำ	ปฏิบัติบ่อย	ปฏิบัติบ่อย	ปฏิบัติประจำ	ปฏิบัติประจำ	36	0
ปฏิบัติประจำ	ปฏิบัติประจำ	ปฏิบัติบ่อย	ปฏิบัติประจำ	ปฏิบัติบ่อย	ปฏิบัติประจำ	ปฏิบัติประจำ	39	1
ปฏิบัติประจำ	ปฏิบัติบ่อย	ปฏิบัติบ่อย	ปฏิบัติบ่อย	ปฏิบัติบ่อย	ปฏิบัติบ่อย	ปฏิบัติประจำ	37	5
ปฏิบัติประจำ	ปฏิบัติบางค	ปฏิบัติประจำ	ปฏิบัติประจำ	ปฏิบัติบ่อย	ปฏิบัติบ่อย	ปฏิบัติประจำ	35	0
ปฏิบัติบ่อย	ปฏิบัติบ่อย	ปฏิบัติบ่อย	ปฏิบัติประจำ	ปฏิบัติบางค	ปฏิบัติบางค	ไม่ปฏิบัติเล	28	0

ปฏิบัติบ่อย	ปฏิบัติบางค	ปฏิบัติบ่อย	ปฏิบัติบ่อย	ปฏิบัติบ่อย	ปฏิบัติบ่อย	ปฏิบัติประจำ	28	3
ปฏิบัติประจำ	ปฏิบัติบ่อย	ปฏิบัติบางค	ปฏิบัติบางค	ปฏิบัติบ่อย	ไม่ปฏิบัติเล	ไม่ปฏิบัติเล	28	2
ปฏิบัติบางค	ปฏิบัติบ่อย	ปฏิบัติบางค	ปฏิบัติบ่อย	ปฏิบัติบ่อย	ปฏิบัติบ่อย	ปฏิบัติประจำ	33	0
ปฏิบัติบางค	ปฏิบัติประจำ	ปฏิบัติบ่อย	ไม่ปฏิบัติเล	ปฏิบัติประจำ	ปฏิบัติบางค	ไม่ปฏิบัติเล	21	0
ปฏิบัติประจำ	ปฏิบัติประจำ	ปฏิบัติบ่อย	ปฏิบัติบ่อย	ปฏิบัติบางค	ปฏิบัติบางค	ไม่ปฏิบัติเล	34	4
ปฏิบัติบ่อย	ปฏิบัติบ่อย	ปฏิบัติบ่อย	ปฏิบัติบางค	ปฏิบัติบางค	ไม่ปฏิบัติเล	ไม่ปฏิบัติเล	23	0
ปฏิบัติบ่อย	ปฏิบัติบ่อย	ปฏิบัติบ่อย	ปฏิบัติบางค	ปฏิบัติบางค	ปฏิบัติบ่อย	ไม่ปฏิบัติเล	29	0
ปฏิบัติบางค	ปฏิบัติบางค	ปฏิบัติบ่อย	ปฏิบัติบ่อย	ปฏิบัติบ่อย	ปฏิบัติบางค	ปฏิบัติประจำ	33	0
ปฏิบัติบางค	ปฏิบัติบางค	ปฏิบัติบางค	ปฏิบัติบ่อย	ปฏิบัติประจำ	ปฏิบัติบางค	ปฏิบัติประจำ	31	5
ปฏิบัติบางค	ปฏิบัติบางค	ปฏิบัติประจำ	ปฏิบัติบางค	ปฏิบัติประจำ	ไม่ปฏิบัติเล	ไม่ปฏิบัติเล	33	0
ปฏิบัติบางค	ปฏิบัติบางค	ปฏิบัติบางค	ปฏิบัติบางค	ไม่ปฏิบัติเล	ปฏิบัติบางค	ปฏิบัติประจำ	26	1
ปฏิบัติบางค	ปฏิบัติบ่อย	ปฏิบัติบางค	ปฏิบัติบางค	ปฏิบัติประจำ	ไม่ปฏิบัติเล	ไม่ปฏิบัติเล	21	0
ปฏิบัติบ่อย	ปฏิบัติบ่อย	ปฏิบัติบางค	ปฏิบัติบางค	ปฏิบัติประจำ	ปฏิบัติบ่อย	ไม่ปฏิบัติเล	18	0
ปฏิบัติบ่อย	ปฏิบัติประจำ	ปฏิบัติประจำ	ปฏิบัติบางค	ปฏิบัติประจำ	ปฏิบัติบางค	ปฏิบัติประจำ	29	1
ปฏิบัติบ่อย	ปฏิบัติบ่อย	ปฏิบัติบ่อย	ปฏิบัติบางค	ปฏิบัติบางค	ปฏิบัติบ่อย	ปฏิบัติประจำ	33	3
ปฏิบัติประจำ	ปฏิบัติประจำ	ปฏิบัติประจำ	ปฏิบัติบางค	ปฏิบัติบ่อย	ปฏิบัติบางค	ไม่ปฏิบัติเล	34	2
ปฏิบัติประจำ	ปฏิบัติประจำ	ปฏิบัติประจำ	ปฏิบัติประจำ	ปฏิบัติบ่อย	ไม่ปฏิบัติเล	ไม่ปฏิบัติเล	32	0
ปฏิบัติบางค	ปฏิบัติบ่อย	ปฏิบัติบางค	ปฏิบัติบ่อย	ปฏิบัติบ่อย	ปฏิบัติบ่อย	ปฏิบัติประจำ	33	0
ปฏิบัติบางค	ปฏิบัติบ่อย	ปฏิบัติบ่อย	ปฏิบัติบ่อย	ปฏิบัติบางค	ปฏิบัติประจำ	ไม่ปฏิบัติเล	26	7
ปฏิบัติบางค	ปฏิบัติบางค	ปฏิบัติบางค	ปฏิบัติบางค	ปฏิบัติประจำ	ปฏิบัติบางค	ไม่ปฏิบัติเล	22	0
ปฏิบัติบ่อย	ปฏิบัติประจำ	ปฏิบัติบ่อย	ปฏิบัติบางค	ปฏิบัติประจำ	ปฏิบัติบางค	ไม่ปฏิบัติเล	25	0
ปฏิบัติประจำ	ปฏิบัติประจำ	ปฏิบัติประจำ	ปฏิบัติประจำ	ปฏิบัติประจำ	ปฏิบัติประจำ	ไม่ปฏิบัติเล	34	0
ปฏิบัติประจำ	ปฏิบัติบ่อย	ปฏิบัติบางค	ปฏิบัติบางค	ปฏิบัติประจำ	ปฏิบัติบางค	ไม่ปฏิบัติเล	32	5
ปฏิบัติบางค	ปฏิบัติบ่อย	ปฏิบัติบางค	ปฏิบัติบ่อย	ปฏิบัติบ่อย	ปฏิบัติบางค	ปฏิบัติประจำ	29	0
ปฏิบัติบ่อย	ปฏิบัติบางค	ปฏิบัติบางค	ปฏิบัติบางค	ปฏิบัติบ่อย	ปฏิบัติบางค	ไม่ปฏิบัติเล	22	0
ปฏิบัติบ่อย	ปฏิบัติบ่อย	ปฏิบัติบ่อย	ปฏิบัติบางค	ปฏิบัติบ่อย	ปฏิบัติบางค	ปฏิบัติประจำ	34	2
ปฏิบัติบ่อย	ปฏิบัติบ่อย	ปฏิบัติบ่อย	ปฏิบัติบ่อย	ปฏิบัติประจำ	ปฏิบัติบางค	ปฏิบัติประจำ	30	0
ปฏิบัติประจำ	ปฏิบัติประจำ	ปฏิบัติบ่อย	ปฏิบัติบ่อย	ปฏิบัติบ่อย	ปฏิบัติบ่อย	ปฏิบัติประจำ	38	0
ปฏิบัติบ่อย	ปฏิบัติบ่อย	ปฏิบัติบ่อย	ปฏิบัติบางค	ปฏิบัติประจำ	ปฏิบัติบางค	ไม่ปฏิบัติเล	27	4
ปฏิบัติบ่อย	ปฏิบัติบ่อย	ปฏิบัติบ่อย	ปฏิบัติบางค	ปฏิบัติประจำ	ปฏิบัติบางค	ไม่ปฏิบัติเล	27	0
ปฏิบัติบางค	ปฏิบัติบ่อย	ปฏิบัติบ่อย	ปฏิบัติบ่อย	ปฏิบัติประจำ	ไม่ปฏิบัติเล	ปฏิบัติบางค	32	6
ปฏิบัติบ่อย	ปฏิบัติบ่อย	ปฏิบัติบ่อย	ปฏิบัติบ่อย	ปฏิบัติบ่อย	ปฏิบัติบ่อย	ไม่ปฏิบัติเล	27	0
ปฏิบัติประจำ	ปฏิบัติบ่อย	ปฏิบัติบางค	ปฏิบัติบางค	ปฏิบัติบ่อย	ไม่ปฏิบัติเล	ไม่ปฏิบัติเล	28	0
ปฏิบัติบางค	ปฏิบัติประจำ	ปฏิบัติบ่อย	ไม่ปฏิบัติเล	ปฏิบัติประจำ	ปฏิบัติบางค	ไม่ปฏิบัติเล	21	4
ปฏิบัติประจำ	ปฏิบัติประจำ	ปฏิบัติบ่อย	ปฏิบัติบ่อย	ปฏิบัติบางค	ปฏิบัติบางค	ปฏิบัติประจำ	37	0
ปฏิบัติบ่อย	ปฏิบัติบ่อย	ปฏิบัติบ่อย	ปฏิบัติบางค	ปฏิบัติบางค	ไม่ปฏิบัติเล	ไม่ปฏิบัติเล	23	0
ปฏิบัติบ่อย	ปฏิบัติบ่อย	ปฏิบัติบ่อย	ปฏิบัติบางค	ปฏิบัติบางค	ปฏิบัติบ่อย	ไม่ปฏิบัติเล	29	0
ปฏิบัติบางค	ปฏิบัติบางค	ปฏิบัติบางค	ปฏิบัติบ่อย	ปฏิบัติบ่อย	ปฏิบัติบางค	ปฏิบัติประจำ	32	0
ปฏิบัติบางค	ปฏิบัติบางค	ปฏิบัติบางค	ปฏิบัติบ่อย	ปฏิบัติประจำ	ปฏิบัติบางค	ปฏิบัติประจำ	31	0
ปฏิบัติบางค	ปฏิบัติประจำ	ไม่ปฏิบัติเล	ปฏิบัติบางค	ปฏิบัติประจำ	ไม่ปฏิบัติเล	ไม่ปฏิบัติเล	30	5
ปฏิบัติบางค	ปฏิบัติบางค	ปฏิบัติบางค	ปฏิบัติบางค	ไม่ปฏิบัติเล	ปฏิบัติบางค	ปฏิบัติประจำ	26	2
ปฏิบัติบางค	ปฏิบัติบ่อย	ปฏิบัติบางค	ปฏิบัติบางค	ปฏิบัติประจำ	ไม่ปฏิบัติเล	ไม่ปฏิบัติเล	21	7
ปฏิบัติบ่อย	ปฏิบัติบ่อย	ปฏิบัติบางค	ปฏิบัติบางค	ปฏิบัติประจำ	ปฏิบัติบ่อย	ไม่ปฏิบัติเล	18	0
ปฏิบัติบ่อย	ปฏิบัติประจำ	ปฏิบัติประจำ	ปฏิบัติบางค	ปฏิบัติประจำ	ปฏิบัติบางค	ปฏิบัติประจำ	29	0
ปฏิบัติบ่อย	ปฏิบัติบ่อย	ปฏิบัติบ่อย	ปฏิบัติบางค	ปฏิบัติบางค	ปฏิบัติบ่อย	ปฏิบัติประจำ	33	0
ปฏิบัติประจำ	ปฏิบัติประจำ	ปฏิบัติประจำ	ปฏิบัติบางค	ปฏิบัติบ่อย	ปฏิบัติบางค	ไม่ปฏิบัติเล	34	1
ปฏิบัติประจำ	ปฏิบัติประจำ	ปฏิบัติประจำ	ปฏิบัติประจำ	ปฏิบัติบ่อย	ไม่ปฏิบัติเล	ไม่ปฏิบัติเล	32	6
ปฏิบัติบางค	ปฏิบัติบ่อย	ปฏิบัติบางค	ปฏิบัติบ่อย	ปฏิบัติบ่อย	ปฏิบัติบ่อย	ปฏิบัติประจำ	33	0
ปฏิบัติบ่อย	ปฏิบัติบ่อย	ปฏิบัติบ่อย	ปฏิบัติบางค	ปฏิบัติประจำ	ปฏิบัติบางค	ไม่ปฏิบัติเล	26	1
ปฏิบัติประจำ	ปฏิบัติบางค	ปฏิบัติบางค	ปฏิบัติบางค	ปฏิบัติประจำ	ปฏิบัติบางค	ไม่ปฏิบัติเล	22	4
ปฏิบัติบ่อย	ปฏิบัติประจำ	ปฏิบัติบ่อย	ปฏิบัติบางค	ปฏิบัติประจำ	ปฏิบัติบางค	ไม่ปฏิบัติเล	25	0

ปฏิบัติประจำ	ปฏิบัติประจำ	ปฏิบัติประจำ	ปฏิบัติประจำ	ปฏิบัติประจำ	ปฏิบัติประจำ	ไม่ปฏิบัติเล	34	0
ปฏิบัติประจำ	ปฏิบัติบ่อย(	ปฏิบัติบางค	ปฏิบัติบางค	ปฏิบัติประจำ	ปฏิบัติบางค	ไม่ปฏิบัติเล	32	1
ปฏิบัติบางค	ปฏิบัติบ่อย(	ปฏิบัติบางค	ปฏิบัติบ่อย(	ปฏิบัติบ่อย(	ปฏิบัติบางค	ปฏิบัติประจำ	29	0
ปฏิบัติบ่อย(	ปฏิบัติบางค	ปฏิบัติบางค	ปฏิบัติบางค	ปฏิบัติบ่อย(	ปฏิบัติบางค	ไม่ปฏิบัติเล	22	0
ปฏิบัติบ่อย(	ปฏิบัติบ่อย(	ปฏิบัติบ่อย(	ปฏิบัติบ่อย(	ปฏิบัติบางค	ปฏิบัติบางค	ปฏิบัติประจำ	34	3
ปฏิบัติบางค	ปฏิบัติบางค	ปฏิบัติบางค	ปฏิบัติบ่อย(	ปฏิบัติบ่อย(	ปฏิบัติบางค	ไม่ปฏิบัติเล	18	0
ปฏิบัติบ่อย(	ปฏิบัติบ่อย(	ปฏิบัติบ่อย(	ปฏิบัติบ่อย(	ปฏิบัติประจำ	ปฏิบัติบางค	ไม่ปฏิบัติเล	25	2
ปฏิบัติบางค	ปฏิบัติบางค	ปฏิบัติบางค	ปฏิบัติบางค	ปฏิบัติประจำ	ปฏิบัติบ่อย(	ปฏิบัติประจำ	26	0
ปฏิบัติบางค	ปฏิบัติบ่อย(	ปฏิบัติบางค	ปฏิบัติบ่อย(	ปฏิบัติบ่อย(	ปฏิบัติบ่อย(	ไม่ปฏิบัติเล	24	0
ปฏิบัติประจำ	ปฏิบัติบ่อย(	ปฏิบัติบางค	ปฏิบัติบางค	ปฏิบัติบ่อย(	ไม่ปฏิบัติเล	ไม่ปฏิบัติเล	28	1
ปฏิบัติบางค	ปฏิบัติประจำ	ปฏิบัติบ่อย(	ไม่ปฏิบัติเล	ปฏิบัติประจำ	ปฏิบัติบางค	ไม่ปฏิบัติเล	21	0
ปฏิบัติบางค	ปฏิบัติประจำ	ปฏิบัติบ่อย(	ไม่ปฏิบัติเล	ปฏิบัติประจำ	ปฏิบัติบางค	ไม่ปฏิบัติเล	21	0
ปฏิบัติบางค	ปฏิบัติบ่อย(	ปฏิบัติบ่อย(	ปฏิบัติบางค	ปฏิบัติบางค	ไม่ปฏิบัติเล	ไม่ปฏิบัติเล	24	0
ปฏิบัติบ่อย(	ปฏิบัติบ่อย(	ปฏิบัติบ่อย(	ปฏิบัติบางค	ปฏิบัติบางค	ปฏิบัติบ่อย(	ไม่ปฏิบัติเล	29	1
ปฏิบัติบางค	ปฏิบัติบางค	ปฏิบัติบางค	ปฏิบัติบ่อย(	ปฏิบัติบ่อย(	ปฏิบัติบางค	ปฏิบัติประจำ	32	0
ปฏิบัติบางค	ปฏิบัติบางค	ปฏิบัติบางค	ปฏิบัติบ่อย(	ปฏิบัติประจำ	ปฏิบัติบางค	ปฏิบัติประจำ	31	2
ปฏิบัติบางค	ปฏิบัติประจำ	ไม่ปฏิบัติเล	ปฏิบัติบางค	ปฏิบัติประจำ	ไม่ปฏิบัติเล	ไม่ปฏิบัติเล	30	2
ปฏิบัติบางค	ปฏิบัติบางค	ปฏิบัติบางค	ไม่ปฏิบัติเล	ปฏิบัติบางค	ปฏิบัติบางค	ปฏิบัติประจำ	26	0
ปฏิบัติบางค	ปฏิบัติบ่อย(	ปฏิบัติบางค	ปฏิบัติบางค	ปฏิบัติประจำ	ไม่ปฏิบัติเล	ไม่ปฏิบัติเล	21	0
ปฏิบัติบ่อย(	ปฏิบัติบ่อย(	ปฏิบัติบางค	ปฏิบัติบางค	ปฏิบัติประจำ	ปฏิบัติบ่อย(	ไม่ปฏิบัติเล	18	4
ปฏิบัติบ่อย(	ปฏิบัติประจำ	ปฏิบัติประจำ	ปฏิบัติบางค	ปฏิบัติประจำ	ปฏิบัติบางค	ปฏิบัติประจำ	29	0
ปฏิบัติบ่อย(	ปฏิบัติบ่อย(	ปฏิบัติบ่อย(	ปฏิบัติบางค	ปฏิบัติบางค	ปฏิบัติบ่อย(	ปฏิบัติประจำ	33	0
ปฏิบัติประจำ	ปฏิบัติประจำ	ปฏิบัติประจำ	ปฏิบัติบางค	ปฏิบัติบ่อย(	ปฏิบัติบางค	ไม่ปฏิบัติเล	34	2
ปฏิบัติประจำ	ปฏิบัติประจำ	ปฏิบัติประจำ	ปฏิบัติประจำ	ปฏิบัติบ่อย(	ไม่ปฏิบัติเล	ไม่ปฏิบัติเล	32	0
ปฏิบัติบางค	ปฏิบัติบางค	ปฏิบัติบางค	ปฏิบัติบางค	ปฏิบัติประจำ	ปฏิบัติบางค	ปฏิบัติประจำ	29	2
ปฏิบัติบ่อย(	ปฏิบัติบางค	ปฏิบัติบางค	ปฏิบัติบางค	ปฏิบัติประจำ	ปฏิบัติบางค	ไม่ปฏิบัติเล	20	1
ปฏิบัติบางค	ปฏิบัติบางค	ไม่ปฏิบัติเล	ปฏิบัติบ่อย(	ปฏิบัติประจำ	ปฏิบัติบางค	ปฏิบัติประจำ	30	3
ปฏิบัติบ่อย(	ปฏิบัติบ่อย(	ปฏิบัติบางค	ไม่ปฏิบัติเล	ปฏิบัติบ่อย(	ปฏิบัติบ่อย(	ไม่ปฏิบัติเล	16	0
ปฏิบัติบ่อย(	ปฏิบัติบางค	ไม่ปฏิบัติเล	ปฏิบัติบางค	ปฏิบัติประจำ	ปฏิบัติบางค	ปฏิบัติประจำ	23	0
ปฏิบัติบ่อย(	ปฏิบัติบ่อย(	ปฏิบัติบางค	ไม่ปฏิบัติเล	ปฏิบัติบ่อย(	ปฏิบัติบ่อย(	ไม่ปฏิบัติเล	17	0
ปฏิบัติบางค	ปฏิบัติบางค	ไม่ปฏิบัติเล	ปฏิบัติบางค	ปฏิบัติประจำ	ปฏิบัติบางค	ปฏิบัติประจำ	26	2
ปฏิบัติบางค	ปฏิบัติบางค	ปฏิบัติบางค	ปฏิบัติบางค	ปฏิบัติบางค	ปฏิบัติบางค	ไม่ปฏิบัติเล	14	1
ปฏิบัติบางค	ปฏิบัติบางค	ปฏิบัติบางค	ปฏิบัติบางค	ปฏิบัติบางค	ปฏิบัติบางค	ไม่ปฏิบัติเล	16	0
ปฏิบัติบางค	ปฏิบัติบางค	ปฏิบัติบางค	ไม่ปฏิบัติเล	ปฏิบัติประจำ	ปฏิบัติบางค	ไม่ปฏิบัติเล	12	0
ปฏิบัติบ่อย(	ปฏิบัติบ่อย(	ปฏิบัติบ่อย(	ปฏิบัติบ่อย(	ปฏิบัติบ่อย(	ปฏิบัติบ่อย(	ปฏิบัติประจำ	31	0
ปฏิบัติบางค	ปฏิบัติบางค	ปฏิบัติบางค	ปฏิบัติบางค	ปฏิบัติบ่อย(	ปฏิบัติบางค	ไม่ปฏิบัติเล	15	3
ปฏิบัติประจำ	ปฏิบัติประจำ	ปฏิบัติประจำ	ไม่ปฏิบัติเล	ปฏิบัติประจำ	ปฏิบัติบางค	ปฏิบัติประจำ	28	0
ปฏิบัติบ่อย(	ปฏิบัติบ่อย(	ปฏิบัติบ่อย(	ปฏิบัติบางค	ปฏิบัติประจำ	ปฏิบัติบางค	ไม่ปฏิบัติเล	25	1
ปฏิบัติบ่อย(	ปฏิบัติบ่อย(	ปฏิบัติบ่อย(	ปฏิบัติบางค	ปฏิบัติประจำ	ปฏิบัติบางค	ไม่ปฏิบัติเล	25	0
ปฏิบัติประจำ	ปฏิบัติประจำ	ปฏิบัติประจำ	ปฏิบัติบางค	ปฏิบัติบางค	ปฏิบัติประจำ	ปฏิบัติประจำ	41	0
ปฏิบัติบ่อย(	ปฏิบัติบางค	ปฏิบัติบางค	ปฏิบัติบางค	ปฏิบัติประจำ	ปฏิบัติบางค	ไม่ปฏิบัติเล	25	0
ปฏิบัติประจำ	ปฏิบัติบ่อย(	ปฏิบัติบางค	ไม่ปฏิบัติเล	ปฏิบัติประจำ	ปฏิบัติบางค	ไม่ปฏิบัติเล	22	0
ปฏิบัติบ่อย(	ปฏิบัติบางค	ปฏิบัติบางค	ปฏิบัติบางค	ปฏิบัติประจำ	ไม่ปฏิบัติเล	ปฏิบัติประจำ	28	0
ปฏิบัติบางค	ปฏิบัติประจำ	ปฏิบัติบางค	ปฏิบัติบ่อย(	ปฏิบัติบ่อย(	ปฏิบัติบ่อย(	ปฏิบัติประจำ	34	0
ปฏิบัติบางค	ปฏิบัติบางค	ปฏิบัติบางค	ปฏิบัติบางค	ปฏิบัติบางค	ปฏิบัติบางค	ไม่ปฏิบัติเล	14	1
ปฏิบัติบ่อย(	ปฏิบัติบางค	ปฏิบัติบางค	ไม่ปฏิบัติเล	ปฏิบัติบ่อย(	ปฏิบัติบางค	ไม่ปฏิบัติเล	18	0
ปฏิบัติบ่อย(	ปฏิบัติบ่อย(	ปฏิบัติบางค	ปฏิบัติบางค	ปฏิบัติประจำ	ปฏิบัติบางค	ปฏิบัติประจำ	27	1
ปฏิบัติบางค	ปฏิบัติบางค	ปฏิบัติบางค	ปฏิบัติบางค	ปฏิบัติบางค	ปฏิบัติบางค	ไม่ปฏิบัติเล	14	0
ปฏิบัติบ่อย(	ปฏิบัติบ่อย(	ปฏิบัติบ่อย(	ปฏิบัติบางค	ปฏิบัติประจำ	ปฏิบัติบ่อย(	ไม่ปฏิบัติเล	31	5
ปฏิบัติบางค	ปฏิบัติบางค	ปฏิบัติบ่อย(	ปฏิบัติบ่อย(	ปฏิบัติประจำ	ปฏิบัติบางค	ไม่ปฏิบัติเล	19	2
ปฏิบัติบางค	ปฏิบัติบางค	ปฏิบัติบางค	ปฏิบัติบ่อย(	ปฏิบัติบ่อย(	ปฏิบัติบ่อย(	ปฏิบัติประจำ	27	2

ปฏิบัติบ่อย	ปฏิบัติบ่อย	ปฏิบัติบ่อย	ปฏิบัติบ่อย	ปฏิบัติบ่อย	ปฏิบัติบางค	ไม่ปฏิบัติเล	19	0
ปฏิบัติบางค	ปฏิบัติประจำ	ปฏิบัติบางค	ไม่ปฏิบัติเล	ปฏิบัติบางค	ปฏิบัติบางค	ไม่ปฏิบัติเล	23	0
ปฏิบัติบ่อย	ปฏิบัติบางค	ปฏิบัติบางค	ปฏิบัติบางค	ปฏิบัติบางค	ปฏิบัติบางค	ไม่ปฏิบัติเล	23	3
ปฏิบัติประจำ	ปฏิบัติบ่อย	ปฏิบัติบางค	ปฏิบัติบางค	ปฏิบัติประจำ	ปฏิบัติบางค	ไม่ปฏิบัติเล	26	0
ปฏิบัติประจำ	ปฏิบัติบ่อย	ปฏิบัติบ่อย	ปฏิบัติบ่อย	ปฏิบัติบ่อย	ปฏิบัติบ่อย	ไม่ปฏิบัติเล	32	4
ปฏิบัติบ่อย	ปฏิบัติบ่อย	ปฏิบัติบ่อย	ปฏิบัติบ่อย	ปฏิบัติบางค	ปฏิบัติบ่อย	ไม่ปฏิบัติเล	27	0
ปฏิบัติบางค	ปฏิบัติบางค	ไม่ปฏิบัติเล	ปฏิบัติบางค	ปฏิบัติบางค	ปฏิบัติบางค	ไม่ปฏิบัติเล	17	0
ปฏิบัติบางค	ปฏิบัติบางค	ปฏิบัติบางค	ปฏิบัติบางค	ปฏิบัติบ่อย	ปฏิบัติบางค	ไม่ปฏิบัติเล	21	0
ปฏิบัติประจำ	ปฏิบัติบ่อย	ปฏิบัติบางค	ปฏิบัติประจำ	ปฏิบัติประจำ	ปฏิบัติบางค	ไม่ปฏิบัติเล	29	3
ปฏิบัติบ่อย	ปฏิบัติบ่อย	ปฏิบัติบางค	ปฏิบัติบางค	ปฏิบัติประจำ	ปฏิบัติบางค	ปฏิบัติประจำ	31	0
ปฏิบัติบางค	ปฏิบัติบางค	ปฏิบัติบางค	ปฏิบัติบางค	ปฏิบัติบ่อย	ปฏิบัติบางค	ไม่ปฏิบัติเล	21	0
ปฏิบัติบ่อย	ปฏิบัติบางค	ปฏิบัติบางค	ปฏิบัติบางค	ปฏิบัติบ่อย	ปฏิบัติบางค	ไม่ปฏิบัติเล	22	5
ปฏิบัติประจำ	ปฏิบัติประจำ	ปฏิบัติบางค	ปฏิบัติบ่อย	ปฏิบัติประจำ	ปฏิบัติประจำ	ไม่ปฏิบัติเล	33	0
ปฏิบัติบางค	ปฏิบัติบางค	ปฏิบัติบางค	ปฏิบัติบ่อย	ปฏิบัติประจำ	ไม่ปฏิบัติเล	ปฏิบัติประจำ	32	0
ปฏิบัติบางค	ปฏิบัติบางค	ปฏิบัติบางค	ปฏิบัติบางค	ปฏิบัติบางค	ปฏิบัติบางค	ปฏิบัติประจำ	27	0
ปฏิบัติบ่อย	ปฏิบัติบางค	ปฏิบัติบ่อย	ปฏิบัติประจำ	ปฏิบัติบางค	ปฏิบัติบางค	ปฏิบัติบางค	28	0
ปฏิบัติบ่อย	ปฏิบัติบางค	ปฏิบัติบางค	ปฏิบัติบางค	ปฏิบัติบางค	ปฏิบัติบางค	ไม่ปฏิบัติเล	24	6
ปฏิบัติบางค	ปฏิบัติบางค	ปฏิบัติบางค	ปฏิบัติบ่อย	ปฏิบัติบ่อย	ปฏิบัติบางค	ไม่ปฏิบัติเล	19	3
ปฏิบัติบางค	ปฏิบัติบางค	ปฏิบัติบางค	ปฏิบัติบางค	ปฏิบัติบางค	ปฏิบัติบางค	ไม่ปฏิบัติเล	23	0
ปฏิบัติประจำ	ปฏิบัติประจำ	ปฏิบัติบ่อย	ปฏิบัติบ่อย	ปฏิบัติประจำ	ปฏิบัติบางค	ปฏิบัติประจำ	33	0
ปฏิบัติประจำ	ปฏิบัติประจำ	ปฏิบัติประจำ	ปฏิบัติประจำ	ปฏิบัติประจำ	ปฏิบัติประจำ	ปฏิบัติประจำ	40	0
ปฏิบัติประจำ	ปฏิบัติประจำ	ปฏิบัติประจำ	ปฏิบัติประจำ	ปฏิบัติบางค	ปฏิบัติประจำ	ไม่ปฏิบัติเล	35	4
ปฏิบัติบางค	ปฏิบัติบางค	ปฏิบัติบางค	ปฏิบัติบางค	ปฏิบัติบางค	ปฏิบัติบางค	ไม่ปฏิบัติเล	24	0
ปฏิบัติบางค	ไม่ปฏิบัติเล	ไม่ปฏิบัติเล	ไม่ปฏิบัติเล	ปฏิบัติบางค	ไม่ปฏิบัติเล	ไม่ปฏิบัติเล	14	5
ปฏิบัติบางค	ไม่ปฏิบัติเล	ปฏิบัติบางค	ปฏิบัติบางค	ปฏิบัติบ่อย	ปฏิบัติบางค	ไม่ปฏิบัติเล	23	0
ปฏิบัติบางค	ปฏิบัติบางค	ปฏิบัติบางค	ปฏิบัติบางค	ปฏิบัติบ่อย	ไม่ปฏิบัติเล	ปฏิบัติประจำ	29	2
ปฏิบัติบางค	ปฏิบัติบางค	ปฏิบัติบางค	ปฏิบัติบางค	ปฏิบัติประจำ	ไม่ปฏิบัติเล	ไม่ปฏิบัติเล	21	0
ปฏิบัติประจำ	ปฏิบัติบางค	ไม่ปฏิบัติเล	ปฏิบัติประจำ	ปฏิบัติบ่อย	ปฏิบัติบางค	ไม่ปฏิบัติเล	30	0
ปฏิบัติบางค	ปฏิบัติบ่อย	ปฏิบัติบางค	ปฏิบัติบางค	ไม่ปฏิบัติเล	ไม่ปฏิบัติเล	ไม่ปฏิบัติเล	20	1
ปฏิบัติประจำ	ปฏิบัติบางค	ปฏิบัติบางค	ปฏิบัติประจำ	ไม่ปฏิบัติเล	ปฏิบัติบางค	ไม่ปฏิบัติเล	24	3
ปฏิบัติบางค	ปฏิบัติบางค	ปฏิบัติบางค	ปฏิบัติบางค	ปฏิบัติบางค	ไม่ปฏิบัติเล	ไม่ปฏิบัติเล	13	2
ปฏิบัติบางค	ปฏิบัติบางค	ปฏิบัติบางค	ปฏิบัติบางค	ปฏิบัติประจำ	ไม่ปฏิบัติเล	ไม่ปฏิบัติเล	23	0
ปฏิบัติบางค	ปฏิบัติบางค	ปฏิบัติบ่อย	ปฏิบัติบางค	ปฏิบัติบางค	ปฏิบัติบางค	ไม่ปฏิบัติเล	19	2
ปฏิบัติบางค	ปฏิบัติบางค	ปฏิบัติบางค	ปฏิบัติบ่อย	ปฏิบัติประจำ	ปฏิบัติบางค	ไม่ปฏิบัติเล	24	2
ปฏิบัติบางค	ปฏิบัติบางค	ปฏิบัติบางค	ปฏิบัติบางค	ปฏิบัติบางค	ปฏิบัติบางค	ไม่ปฏิบัติเล	15	2
ปฏิบัติบ่อย	ปฏิบัติบางค	ไม่ปฏิบัติเล	ปฏิบัติบางค	ปฏิบัติประจำ	ปฏิบัติบ่อย	ปฏิบัติบางค	27	0
ปฏิบัติบ่อย	ปฏิบัติบางค	ปฏิบัติบางค	ปฏิบัติบางค	ปฏิบัติบางค	ปฏิบัติบางค	ไม่ปฏิบัติเล	21	0
ปฏิบัติบ่อย	ปฏิบัติบ่อย	ปฏิบัติบางค	ปฏิบัติบางค	ปฏิบัติบางค	ปฏิบัติบางค	ไม่ปฏิบัติเล	19	0
ปฏิบัติบ่อย	ปฏิบัติบ่อย	ปฏิบัติบ่อย	ปฏิบัติบ่อย	ปฏิบัติประจำ	ปฏิบัติบางค	ไม่ปฏิบัติเล	32	3
ปฏิบัติบ่อย	ปฏิบัติบ่อย	ปฏิบัติบ่อย	ปฏิบัติบางค	ปฏิบัติประจำ	ปฏิบัติบ่อย	ไม่ปฏิบัติเล	31	5
ปฏิบัติบางค	ปฏิบัติบางค	ปฏิบัติบ่อย	ปฏิบัติบ่อย	ปฏิบัติประจำ	ปฏิบัติบางค	ไม่ปฏิบัติเล	19	2
ปฏิบัติบางค	ปฏิบัติบางค	ปฏิบัติบางค	ปฏิบัติบ่อย	ปฏิบัติบ่อย	ปฏิบัติบ่อย	ปฏิบัติประจำ	27	2
ปฏิบัติบ่อย	ปฏิบัติบ่อย	ปฏิบัติบ่อย	ปฏิบัติบ่อย	ปฏิบัติบ่อย	ปฏิบัติบางค	ไม่ปฏิบัติเล	19	0
ปฏิบัติบางค	ปฏิบัติประจำ	ปฏิบัติบางค	ไม่ปฏิบัติเล	ปฏิบัติบางค	ปฏิบัติบางค	ไม่ปฏิบัติเล	23	0
ปฏิบัติบ่อย	ปฏิบัติบางค	ปฏิบัติบางค	ปฏิบัติบางค	ปฏิบัติบางค	ปฏิบัติบางค	ไม่ปฏิบัติเล	23	3
ปฏิบัติประจำ	ปฏิบัติบ่อย	ปฏิบัติบางค	ปฏิบัติบางค	ปฏิบัติประจำ	ปฏิบัติบางค	ไม่ปฏิบัติเล	26	0
ปฏิบัติบางค	ปฏิบัติบ่อย	ปฏิบัติบางค	ปฏิบัติบางค	ปฏิบัติบางค	ปฏิบัติบางค	ไม่ปฏิบัติเล	26	1
ปฏิบัติบ่อย	ปฏิบัติบ่อย	ปฏิบัติบางค	ปฏิบัติบางค	ปฏิบัติบางค	ปฏิบัติบางค	ไม่ปฏิบัติเล	26	3
ปฏิบัติบางค	ปฏิบัติประจำ	ปฏิบัติบ่อย	ปฏิบัติประจำ	ปฏิบัติประจำ	ปฏิบัติบ่อย	ไม่ปฏิบัติเล	35	2

gingivitis	calculus	edu_G	อาชีพ_G	สมรส_G	family_G	health_ser	Living_G	Abortion_
มีเหงือกอัก	มีหินน้ำลาย	ม.ปลาย/ปว	ว่างงาน	อยู่ด้วยกันก	ครอบครัวเดี	สิทธิหลักปร	ไม่มีลูก	ไม่เคยแท้ง
มีเหงือกอัก	มีหินน้ำลาย	ม.ปลาย/ปว	ว่างงาน	อยู่ด้วยกันก	ครอบครัวเดี	สิทธิหลักปร	ไม่มีลูก	ไม่เคยแท้ง
มีเหงือกอัก	มีหินน้ำลาย	ม.ต้น	ว่างงาน	อยู่ด้วยกันก	ครอบครัวเดี	สิทธิหลักปร	มีลูกแล้วอย	ไม่เคยแท้ง
มีเหงือกอัก	มีหินน้ำลาย	ป.ตรีขึ้นไป	มีงานทำ	ไม่ได้อยู่กับ	ครอบครัวเดี	สิทธิข้าราช	ไม่มีลูก	ไม่เคยแท้ง
ไม่มีเหงือกอ	มีหินน้ำลาย	ป.ตรีขึ้นไป	ว่างงาน	อยู่ด้วยกันก	ครอบครัวเดี	สิทธิประกัน	มีลูกแล้วอย	ไม่เคยแท้ง
มีเหงือกอัก	มีหินน้ำลาย	ม.ต้น	มีงานทำ	อยู่ด้วยกันก	ครอบครัวเดี	สิทธิหลักปร	ไม่มีลูก	ไม่เคยแท้ง
ไม่มีเหงือกอ	ไม่มีหินน้ำล	ม.ปลาย/ปว	มีงานทำ	อยู่ด้วยกันก	ครอบครัวขย	สิทธิหลักปร	มีลูกแล้วอย	ไม่เคยแท้ง
ไม่มีเหงือกอ	ไม่มีหินน้ำล	ม.ปลาย/ปว	มีงานทำ	อยู่ด้วยกันก	ครอบครัวเดี	สิทธิประกัน	มีลูกแล้วอย	เคยแท้ง
ไม่มีเหงือกอ	ไม่มีหินน้ำล	ปวส.	ว่างงาน	อยู่ด้วยกันก	ครอบครัวขย	สิทธิหลักปร	ไม่มีลูก	ไม่เคยแท้ง
มีเหงือกอัก	มีหินน้ำลาย	ประถมฯ	มีงานทำ	อยู่ด้วยกันก	ครอบครัวเดี	สิทธิหลักปร	ไม่มีลูก	ไม่เคยแท้ง
มีเหงือกอัก	มีหินน้ำลาย	ม.ต้น	มีงานทำ	อยู่ด้วยกันก	ครอบครัวเดี	สิทธิหลักปร	มีลูกแล้วอย	ไม่เคยแท้ง
มีเหงือกอัก	มีหินน้ำลาย	ม.ต้น	ว่างงาน	อยู่ด้วยกันก	ครอบครัวขย	สิทธิหลักปร	มีลูกแล้วอย	ไม่เคยแท้ง
ไม่มีเหงือกอ	ไม่มีหินน้ำล	ประถมฯ	มีงานทำ	อยู่ด้วยกันก	ครอบครัวเดี	สิทธิหลักปร	ไม่มีลูก	ไม่เคยแท้ง
ไม่มีเหงือกอ	ไม่มีหินน้ำล	ม.ปลาย/ปว	ว่างงาน	ไม่ได้อยู่กับ	ครอบครัวเดี	สิทธิหลักปร	ไม่มีลูก	ไม่เคยแท้ง
มีเหงือกอัก	มีหินน้ำลาย	ม.ปลาย/ปว	มีงานทำ	อยู่ด้วยกันก	ครอบครัวเดี	สิทธิหลักปร	ไม่มีลูก	ไม่เคยแท้ง
มีเหงือกอัก	มีหินน้ำลาย	ม.ปลาย/ปว	ว่างงาน	อยู่ด้วยกันก	ครอบครัวเดี	สิทธิหลักปร	ไม่มีลูก	ไม่เคยแท้ง
ไม่มีเหงือกอ	ไม่มีหินน้ำล	ม.ต้น	มีงานทำ	อยู่ด้วยกันก	ครอบครัวเดี	สิทธิหลักปร	ไม่มีลูก	ไม่เคยแท้ง
มีเหงือกอัก	มีหินน้ำลาย	ม.ปลาย/ปว	มีงานทำ	อยู่ด้วยกันก	ครอบครัวเดี	สิทธิประกัน	มีลูกแล้วอย	ไม่เคยแท้ง
ไม่มีเหงือกอ	ไม่มีหินน้ำล	ป.ตรีขึ้นไป	มีงานทำ	อยู่ด้วยกันก	ครอบครัวเดี	สิทธิหลักปร	ไม่มีลูก	ไม่เคยแท้ง
มีเหงือกอัก	มีหินน้ำลาย	ป.ตรีขึ้นไป	มีงานทำ	ไม่ได้อยู่กับ	ครอบครัวเดี	สิทธิข้าราช	ไม่มีลูก	ไม่เคยแท้ง
มีเหงือกอัก	มีหินน้ำลาย	ม.ปลาย/ปว	มีงานทำ	อยู่ด้วยกันก	ครอบครัวเดี	สิทธิข้าราช	ไม่มีลูก	ไม่เคยแท้ง
มีเหงือกอัก	มีหินน้ำลาย	ม.ต้น	ว่างงาน	อยู่ด้วยกันก	ครอบครัวเดี	สิทธิหลักปร	มีลูกแล้วอย	ไม่เคยแท้ง
มีเหงือกอัก	มีหินน้ำลาย	ป.ตรีขึ้นไป	มีงานทำ	อยู่ด้วยกันก	ครอบครัวขย	สิทธิประกัน	มีลูกแล้วอย	ไม่เคยแท้ง
ไม่มีเหงือกอ	ไม่มีหินน้ำล	ประถมฯ	ว่างงาน	อยู่ด้วยกันก	ครอบครัวเดี	สิทธิหลักปร	ไม่มีลูก	ไม่เคยแท้ง
ไม่มีเหงือกอ	ไม่มีหินน้ำล	ม.ปลาย/ปว	มีงานทำ	อยู่ด้วยกันก	ครอบครัวเดี	สิทธิหลักปร	มีลูกแล้วอย	ไม่เคยแท้ง
ไม่มีเหงือกอ	ไม่มีหินน้ำล	ปวส.	ว่างงาน	อยู่ด้วยกันก	ครอบครัวขย	สิทธิหลักปร	มีลูกแล้วอย	เคยแท้ง
มีเหงือกอัก	มีหินน้ำลาย	ปวส.	ว่างงาน	อยู่ด้วยกันก	ครอบครัวเดี	สิทธิหลักปร	ไม่มีลูก	ไม่เคยแท้ง
มีเหงือกอัก	มีหินน้ำลาย	ม.ปลาย/ปว	ว่างงาน	ไม่ได้อยู่กับ	ครอบครัวขย	สิทธิหลักปร	ไม่มีลูก	ไม่เคยแท้ง
ไม่มีเหงือกอ	ไม่มีหินน้ำล	ม.ปลาย/ปว	ว่างงาน	ไม่ได้อยู่กับ	ครอบครัวขย	สิทธิหลักปร	ไม่มีลูก	ไม่เคยแท้ง
มีเหงือกอัก	มีหินน้ำลาย	ม.ปลาย/ปว	มีงานทำ	อยู่ด้วยกันก	ครอบครัวเดี	สิทธิประกัน	มีลูกแล้วอย	ไม่เคยแท้ง
มีเหงือกอัก	มีหินน้ำลาย	ม.ต้น	มีงานทำ	อยู่ด้วยกันก	ครอบครัวเดี	สิทธิหลักปร	ไม่มีลูก	ไม่เคยแท้ง
มีเหงือกอัก	มีหินน้ำลาย	ประถมฯ	ว่างงาน	อยู่ด้วยกันก	ครอบครัวขย	สิทธิหลักปร	ไม่มีลูก	ไม่เคยแท้ง
มีเหงือกอัก	มีหินน้ำลาย	ม.ต้น	มีงานทำ	อยู่ด้วยกันก	ครอบครัวเดี	สิทธิหลักปร	มีลูกแล้วอย	ไม่เคยแท้ง
มีเหงือกอัก	มีหินน้ำลาย	ม.ต้น	มีงานทำ	ไม่ได้อยู่กับ	ครอบครัวเดี	สิทธิหลักปร	ไม่มีลูก	เคยแท้ง
มีเหงือกอัก	มีหินน้ำลาย	ป.ตรีขึ้นไป	มีงานทำ	อยู่ด้วยกันก	ครอบครัวขย	สิทธิหลักปร	ไม่มีลูก	ไม่เคยแท้ง
ไม่มีเหงือกอ	ไม่มีหินน้ำล	ประถมฯ	มีงานทำ	อยู่ด้วยกันก	ครอบครัวเดี	สิทธิหลักปร	มีลูกแล้วอย	ไม่เคยแท้ง
มีเหงือกอัก	มีหินน้ำลาย	ม.ปลาย/ปว	ว่างงาน	อยู่ด้วยกันก	ครอบครัวเดี	สิทธิหลักปร	ไม่มีลูก	ไม่เคยแท้ง
มีเหงือกอัก	มีหินน้ำลาย	ม.ปลาย/ปว	ว่างงาน	อยู่ด้วยกันก	ครอบครัวขย	สิทธิหลักปร	ไม่มีลูก	ไม่เคยแท้ง
มีเหงือกอัก	มีหินน้ำลาย	ป.ตรีขึ้นไป	ว่างงาน	อยู่ด้วยกันก	ครอบครัวเดี	สิทธิประกัน	มีลูกแล้วอย	ไม่เคยแท้ง
มีเหงือกอัก	มีหินน้ำลาย	ม.ปลาย/ปว	ว่างงาน	อยู่ด้วยกันก	ครอบครัวเดี	สิทธิหลักปร	ไม่มีลูก	ไม่เคยแท้ง
มีเหงือกอัก	มีหินน้ำลาย	ม.ปลาย/ปว	มีงานทำ	อยู่ด้วยกันก	ครอบครัวเดี	สิทธิหลักปร	มีลูกแล้วอย	ไม่เคยแท้ง
มีเหงือกอัก	มีหินน้ำลาย	ม.ต้น	ว่างงาน	อยู่ด้วยกันก	ครอบครัวเดี	สิทธิหลักปร	มีลูกแล้วอย	ไม่เคยแท้ง
มีเหงือกอัก	มีหินน้ำลาย	ม.ต้น	มีงานทำ	อยู่ด้วยกันก	ครอบครัวเดี	สิทธิหลักปร	ไม่มีลูก	ไม่เคยแท้ง
มีเหงือกอัก	มีหินน้ำลาย	ปวส.	ว่างงาน	ไม่ได้อยู่กับ	ครอบครัวขย	สิทธิหลักปร	ไม่มีลูก	ไม่เคยแท้ง
มีเหงือกอัก	มีหินน้ำลาย	ม.ปลาย/ปว	ว่างงาน	ไม่ได้อยู่กับ	ครอบครัวเดี	สิทธิหลักปร	ไม่มีลูก	ไม่เคยแท้ง
มีเหงือกอัก	มีหินน้ำลาย	ม.ปลาย/ปว	ว่างงาน	อยู่ด้วยกันก	ครอบครัวเดี	สิทธิประกัน	ไม่มีลูก	ไม่เคยแท้ง
มีเหงือกอัก	มีหินน้ำลาย	ม.ปลาย/ปว	มีงานทำ	ไม่ได้อยู่กับ	ครอบครัวเดี	สิทธิหลักปร	ไม่มีลูก	ไม่เคยแท้ง
มีเหงือกอัก	มีหินน้ำลาย	ประถมฯ	มีงานทำ	อยู่ด้วยกันก	ครอบครัวเดี	สิทธิหลักปร	มีลูกแล้วอย	ไม่เคยแท้ง
มีเหงือกอัก	มีหินน้ำลาย	ประถมฯ	มีงานทำ	อยู่ด้วยกันก	ครอบครัวขย	สิทธิหลักปร	มีลูกแล้วอย	ไม่เคยแท้ง
มีเหงือกอัก	มีหินน้ำลาย	ปวส.	ว่างงาน	อยู่ด้วยกันก	ครอบครัวเดี	สิทธิหลักปร	มีลูกแล้วอย	ไม่เคยแท้ง

มีเหงือกอัก	มีหินน้ำลาย	ม.ปลาย/ปว	มีงานทำ	อยู่ด้วยกันก	ครอบครัวเ	สิทธิหลักป	ไม่มีลูก	ไม่เคยแท้ง
มีเหงือกอัก	มีหินน้ำลาย	ม.ปลาย/ปว	มีงานทำ	อยู่ด้วยกันก	ครอบครัวเ	สิทธิหลักป	มีลูกแล้วอย	ไม่เคยแท้ง
มีเหงือกอัก	มีหินน้ำลาย	ม.ต้น	มีงานทำ	อยู่ด้วยกันก	ครอบครัวข	สิทธิหลักป	ไม่มีลูก	ไม่เคยแท้ง
มีเหงือกอัก	มีหินน้ำลาย	ปวส.	ว่างงาน	อยู่ด้วยกันก	ครอบครัวเ	สิทธิหลักป	ไม่มีลูก	ไม่เคยแท้ง
มีเหงือกอัก	มีหินน้ำลาย	ป.ตรีขึ้นไป	ว่างงาน	อยู่ด้วยกันก	ครอบครัวเ	สิทธิหลักป	มีลูกแล้วอย	เคยแท้ง
มีเหงือกอัก	มีหินน้ำลาย	ม.ต้น	มีงานทำ	อยู่ด้วยกันก	ครอบครัวเ	สิทธิหลักป	ไม่มีลูก	ไม่เคยแท้ง
มีเหงือกอัก	มีหินน้ำลาย	ป.ตรีขึ้นไป	มีงานทำ	อยู่ด้วยกันก	ครอบครัวข	สิทธิประกัน	มีลูกแล้วอย	ไม่เคยแท้ง
มีเหงือกอัก	มีหินน้ำลาย	ม.ต้น	ว่างงาน	ไม่ได้อยู่กับ	ครอบครัวเ	สิทธิหลักป	ไม่มีลูก	ไม่เคยแท้ง
มีเหงือกอัก	มีหินน้ำลาย	ประถมฯ	ว่างงาน	อยู่ด้วยกันก	ครอบครัวเ	สิทธิประกัน	ไม่มีลูก	ไม่เคยแท้ง
มีเหงือกอัก	มีหินน้ำลาย	ม.ปลาย/ปว	ว่างงาน	อยู่ด้วยกันก	ครอบครัวเ	สิทธิหลักป	มีลูกแล้วอย	ไม่เคยแท้ง
มีเหงือกอัก	มีหินน้ำลาย	ม.ต้น	มีงานทำ	อยู่ด้วยกันก	ครอบครัวข	อื่นๆ	มีลูกแล้วอย	ไม่เคยแท้ง
มีเหงือกอัก	มีหินน้ำลาย	ม.ปลาย/ปว	มีงานทำ	อยู่ด้วยกันก	ครอบครัวเ	สิทธิประกัน	มีลูกแล้วอย	ไม่เคยแท้ง
มีเหงือกอัก	มีหินน้ำลาย	ม.ปลาย/ปว	ว่างงาน	อยู่ด้วยกันก	ครอบครัวข	สิทธิหลักป	มีลูกแล้วอย	ไม่เคยแท้ง
มีเหงือกอัก	มีหินน้ำลาย	ม.ปลาย/ปว	มีงานทำ	อยู่ด้วยกันก	ครอบครัวเ	สิทธิหลักป	มีลูกแล้วอย	ไม่เคยแท้ง
มีเหงือกอัก	มีหินน้ำลาย	ป.ตรีขึ้นไป	มีงานทำ	อยู่ด้วยกันก	ครอบครัวข	สิทธิข้าราช	ไม่มีลูก	ไม่เคยแท้ง
มีเหงือกอัก	มีหินน้ำลาย	ม.ต้น	มีงานทำ	อยู่ด้วยกันก	ครอบครัวเ	สิทธิหลักป	ไม่มีลูก	ไม่เคยแท้ง
มีเหงือกอัก	มีหินน้ำลาย	ม.ต้น	ว่างงาน	อยู่ด้วยกันก	ครอบครัวข	สิทธิหลักป	ไม่มีลูก	เคยแท้ง
มีเหงือกอัก	มีหินน้ำลาย	ปวส.	ว่างงาน	อยู่ด้วยกันก	ครอบครัวข	สิทธิประกัน	มีลูกแล้วอย	ไม่เคยแท้ง
มีเหงือกอัก	มีหินน้ำลาย	ม.ปลาย/ปว	มีงานทำ	อยู่ด้วยกันก	ครอบครัวเ	สิทธิหลักป	ไม่มีลูก	ไม่เคยแท้ง
มีเหงือกอัก	มีหินน้ำลาย	ม.ต้น	ว่างงาน	อยู่ด้วยกันก	ครอบครัวเ	สิทธิหลักป	ไม่มีลูก	ไม่เคยแท้ง
มีเหงือกอัก	มีหินน้ำลาย	ม.ต้น	มีงานทำ	อยู่ด้วยกันก	ครอบครัวข	สิทธิหลักป	ไม่มีลูก	ไม่เคยแท้ง
มีเหงือกอัก	มีหินน้ำลาย	ป.ตรีขึ้นไป	มีงานทำ	อยู่ด้วยกันก	ครอบครัวข	สิทธิหลักป	มีลูกแล้วอย	ไม่เคยแท้ง
มีเหงือกอัก	มีหินน้ำลาย	ปวส.	มีงานทำ	อยู่ด้วยกันก	ครอบครัวเ	สิทธิประกัน	มีลูกแล้วอย	ไม่เคยแท้ง
มีเหงือกอัก	มีหินน้ำลาย	ม.ปลาย/ปว	มีงานทำ	อยู่ด้วยกันก	ครอบครัวเ	สิทธิหลักป	มีลูกแล้วอย	ไม่เคยแท้ง
มีเหงือกอัก	มีหินน้ำลาย	ปวส.	มีงานทำ	อยู่ด้วยกันก	ครอบครัวข	สิทธิหลักป	ไม่มีลูก	ไม่เคยแท้ง
มีเหงือกอัก	มีหินน้ำลาย	ม.ปลาย/ปว	มีงานทำ	อยู่ด้วยกันก	ครอบครัวเ	สิทธิหลักป	มีลูกแล้วอย	ไม่เคยแท้ง
มีเหงือกอัก	มีหินน้ำลาย	ป.ตรีขึ้นไป	มีงานทำ	อยู่ด้วยกันก	ครอบครัวข	สิทธิหลักป	ไม่มีลูก	ไม่เคยแท้ง
มีเหงือกอัก	มีหินน้ำลาย	ม.ปลาย/ปว	มีงานทำ	อยู่ด้วยกันก	ครอบครัวเ	สิทธิหลักป	ไม่มีลูก	ไม่เคยแท้ง
มีเหงือกอัก	มีหินน้ำลาย	ปวส.	ว่างงาน	อยู่ด้วยกันก	ครอบครัวเ	สิทธิหลักป	ไม่มีลูก	ไม่เคยแท้ง
มีเหงือกอัก	มีหินน้ำลาย	ม.ปลาย/ปว	ว่างงาน	อยู่ด้วยกันก	ครอบครัวเ	สิทธิหลักป	มีลูกแล้วอย	ไม่เคยแท้ง
มีเหงือกอัก	มีหินน้ำลาย	ม.ปลาย/ปว	มีงานทำ	อยู่ด้วยกันก	ครอบครัวเ	สิทธิหลักป	มีลูกแล้วอย	ไม่เคยแท้ง
มีเหงือกอัก	มีหินน้ำลาย	ม.ต้น	ว่างงาน	อยู่ด้วยกันก	ครอบครัวเ	สิทธิหลักป	มีลูกแล้วอย	ไม่เคยแท้ง
มีเหงือกอัก	มีหินน้ำลาย	ม.ต้น	ว่างงาน	อยู่ด้วยกันก	ครอบครัวข	สิทธิหลักป	ไม่มีลูก	ไม่เคยแท้ง
มีเหงือกอัก	มีหินน้ำลาย	ม.ปลาย/ปว	มีงานทำ	อยู่ด้วยกันก	ครอบครัวเ	สิทธิหลักป	ไม่มีลูก	ไม่เคยแท้ง
มีเหงือกอัก	มีหินน้ำลาย	ม.ปลาย/ปว	มีงานทำ	อยู่ด้วยกันก	ครอบครัวเ	สิทธิหลักป	มีลูกแล้วอย	ไม่เคยแท้ง
มีเหงือกอัก	มีหินน้ำลาย	ม.ต้น	มีงานทำ	อยู่ด้วยกันก	ครอบครัวเ	สิทธิหลักป	มีลูกแล้วอย	ไม่เคยแท้ง
มีเหงือกอัก	มีหินน้ำลาย	ม.ปลาย/ปว	ว่างงาน	อยู่ด้วยกันก	ครอบครัวข	สิทธิหลักป	ไม่มีลูก	ไม่เคยแท้ง
มีเหงือกอัก	มีหินน้ำลาย	ม.ปลาย/ปว	มีงานทำ	อยู่ด้วยกันก	ครอบครัวเ	สิทธิหลักป	ไม่มีลูก	ไม่เคยแท้ง
มีเหงือกอัก	มีหินน้ำลาย	ม.ปลาย/ปว	มีงานทำ	อยู่ด้วยกันก	ครอบครัวเ	สิทธิหลักป	ไม่มีลูก	ไม่เคยแท้ง
มีเหงือกอัก	มีหินน้ำลาย	ม.ปลาย/ปว	มีงานทำ	อยู่ด้วยกันก	ครอบครัวเ	สิทธิหลักป	มีลูกแล้วอย	ไม่เคยแท้ง
มีเหงือกอัก	มีหินน้ำลาย	ม.ต้น	มีงานทำ	อยู่ด้วยกันก	ครอบครัวเ	สิทธิหลักป	มีลูกแล้วอย	ไม่เคยแท้ง
มีเหงือกอัก	มีหินน้ำลาย	ม.ปลาย/ปว	มีงานทำ	อยู่ด้วยกันก	ครอบครัวเ	สิทธิหลักป	มีลูกแล้วอย	ไม่เคยแท้ง
มีเหงือกอัก	มีหินน้ำลาย	ปวส.	ว่างงาน	อยู่ด้วยกันก	ครอบครัวเ	สิทธิหลักป	ไม่มีลูก	ไม่เคยแท้ง
มีเหงือกอัก	มีหินน้ำลาย	ม.ต้น	ว่างงาน	อยู่ด้วยกันก	ครอบครัวเ	สิทธิประกัน	มีลูกแล้วอย	ไม่เคยแท้ง
มีเหงือกอัก	มีหินน้ำลาย	ปวส.	มีงานทำ	อยู่ด้วยกันก	ครอบครัวเ	สิทธิหลักป	มีลูกแล้วอย	ไม่เคยแท้ง
มีเหงือกอัก	มีหินน้ำลาย	ปวส.	มีงานทำ	อยู่ด้วยกันก	ครอบครัวเ	สิทธิหลักป	ไม่มีลูก	ไม่เคยแท้ง
มีเหงือกอัก	มีหินน้ำลาย	ม.ปลาย/ปว	มีงานทำ	อยู่ด้วยกันก	ครอบครัวเ	สิทธิหลักป	มีลูกแล้วอย	ไม่เคยแท้ง
มีเหงือกอัก	มีหินน้ำลาย	ม.ต้น	ว่างงาน	อยู่ด้วยกันก	ครอบครัวข	สิทธิหลักป	ไม่มีลูก	ไม่เคยแท้ง
มีเหงือกอัก	มีหินน้ำลาย	ประถมฯ	มีงานทำ	ไม่ได้อยู่กับ	ครอบครัวเ	สิทธิประกัน	มีลูกแล้วอย	ไม่เคยแท้ง
มีเหงือกอัก	มีหินน้ำลาย	ป.ตรีขึ้นไป	ว่างงาน	ไม่ได้อยู่กับ	ครอบครัวข	สิทธิหลักป	ไม่มีลูก	ไม่เคยแท้ง
มีเหงือกอัก	มีหินน้ำลาย	ปวส.	มีงานทำ	อยู่ด้วยกันก	ครอบครัวข	สิทธิประกัน	มีลูกแล้วอย	ไม่เคยแท้ง

มีเหงือกอัก	มีหินน้ำลาย	ม.ปลาย/ปว	มีงานทำ	อยู่ด้วยกันก	ครอบครัวเดี	สิทธิหลักป	มีลูกแล้วอย	ไม่เคยแท้ง
มีเหงือกอัก	มีหินน้ำลาย	ม.ปลาย/ปว	มีงานทำ	อยู่ด้วยกันก	ครอบครัวข	สิทธิหลักป	มีลูกแล้วอย	เคยแท้ง
มีเหงือกอัก	มีหินน้ำลาย	ม.ปลาย/ปว	ว่างงาน	อยู่ด้วยกันก	ครอบครัวเดี	สิทธิประกัน	ไม่มีลูก	เคยแท้ง
มีเหงือกอัก	มีหินน้ำลาย	ม.ต้น	มีงานทำ	อยู่ด้วยกันก	ครอบครัวเดี	สิทธิหลักป	มีลูกแล้วอย	เคยแท้ง
มีเหงือกอัก	มีหินน้ำลาย	ปวส.	มีงานทำ	อยู่ด้วยกันก	ครอบครัวเดี	สิทธิประกัน	มีลูกแล้วอย	ไม่เคยแท้ง
มีเหงือกอัก	มีหินน้ำลาย	ม.ต้น	มีงานทำ	อยู่ด้วยกันก	ครอบครัวเดี	สิทธิหลักป	มีลูกแล้วอย	ไม่เคยแท้ง
มีเหงือกอัก	มีหินน้ำลาย	ปวส.	ว่างงาน	อยู่ด้วยกันก	ครอบครัวเดี	สิทธิหลักป	ไม่มีลูก	ไม่เคยแท้ง
มีเหงือกอัก	มีหินน้ำลาย	ปวส.	มีงานทำ	อยู่ด้วยกันก	ครอบครัวเดี	สิทธิหลักป	ไม่มีลูก	ไม่เคยแท้ง
มีเหงือกอัก	มีหินน้ำลาย	ม.ปลาย/ปว	ว่างงาน	อยู่ด้วยกันก	ครอบครัวข	สิทธิหลักป	ไม่มีลูก	ไม่เคยแท้ง
มีเหงือกอัก	มีหินน้ำลาย	ปวส.	มีงานทำ	อยู่ด้วยกันก	ครอบครัวเดี	สิทธิหลักป	ไม่มีลูก	ไม่เคยแท้ง
มีเหงือกอัก	มีหินน้ำลาย	ม.ต้น	ว่างงาน	อยู่ด้วยกันก	ครอบครัวเดี	สิทธิหลักป	ไม่มีลูก	ไม่เคยแท้ง
มีเหงือกอัก	มีหินน้ำลาย	ม.ปลาย/ปว	มีงานทำ	อยู่ด้วยกันก	ครอบครัวเดี	สิทธิหลักป	ไม่มีลูก	ไม่เคยแท้ง
มีเหงือกอัก	มีหินน้ำลาย	ประถมฯ	มีงานทำ	อยู่ด้วยกันก	ครอบครัวเดี	สิทธิหลักป	มีลูกแล้วอย	เคยแท้ง
มีเหงือกอัก	มีหินน้ำลาย	ม.ปลาย/ปว	ว่างงาน	อยู่ด้วยกันก	ครอบครัวข	สิทธิหลักป	มีลูกแล้วอย	ไม่เคยแท้ง
มีเหงือกอัก	มีหินน้ำลาย	ม.ปลาย/ปว	มีงานทำ	อยู่ด้วยกันก	ครอบครัวข	สิทธิหลักป	มีลูกแล้วอย	เคยแท้ง
มีเหงือกอัก	มีหินน้ำลาย	ปวส.	ว่างงาน	อยู่ด้วยกันก	ครอบครัวเดี	สิทธิหลักป	มีลูกแล้วอย	เคยแท้ง
มีเหงือกอัก	มีหินน้ำลาย	ม.ปลาย/ปว	ว่างงาน	ไม่ได้อยู่กับ	ครอบครัวเดี	สิทธิหลักป	ไม่มีลูก	ไม่เคยแท้ง
มีเหงือกอัก	มีหินน้ำลาย	ม.ปลาย/ปว	ว่างงาน	อยู่ด้วยกันก	ครอบครัวเดี	สิทธิหลักป	มีลูกแล้วอย	ไม่เคยแท้ง
มีเหงือกอัก	มีหินน้ำลาย	ม.ปลาย/ปว	มีงานทำ	อยู่ด้วยกันก	ครอบครัวเดี	สิทธิหลักป	มีลูกแล้วอย	ไม่เคยแท้ง
มีเหงือกอัก	มีหินน้ำลาย	ป.ตรีขึ้นไป	มีงานทำ	ไม่ได้อยู่กับ	ครอบครัวเดี	สิทธิหลักป	ไม่มีลูก	ไม่เคยแท้ง
มีเหงือกอัก	มีหินน้ำลาย	ม.ปลาย/ปว	มีงานทำ	อยู่ด้วยกันก	ครอบครัวเดี	สิทธิหลักป	ไม่มีลูก	ไม่เคยแท้ง
มีเหงือกอัก	มีหินน้ำลาย	ม.ปลาย/ปว	มีงานทำ	อยู่ด้วยกันก	ครอบครัวเดี	สิทธิหลักป	ไม่มีลูก	ไม่เคยแท้ง
มีเหงือกอัก	มีหินน้ำลาย	ปวส.	มีงานทำ	อยู่ด้วยกันก	ครอบครัวเดี	อื่นๆ	ไม่มีลูก	ไม่เคยแท้ง
มีเหงือกอัก	มีหินน้ำลาย	ประถมฯ	ว่างงาน	อยู่ด้วยกันก	ครอบครัวข	สิทธิหลักป	มีลูกแล้วอย	เคยแท้ง
มีเหงือกอัก	มีหินน้ำลาย	ม.ปลาย/ปว	มีงานทำ	อยู่ด้วยกันก	ครอบครัวเดี	สิทธิหลักป	ไม่มีลูก	ไม่เคยแท้ง
มีเหงือกอัก	มีหินน้ำลาย	ปวส.	ว่างงาน	อยู่ด้วยกันก	ครอบครัวเดี	สิทธิหลักป	ไม่มีลูก	ไม่เคยแท้ง
มีเหงือกอัก	มีหินน้ำลาย	ปวส.	ว่างงาน	อยู่ด้วยกันก	ครอบครัวข	อื่นๆ	ไม่มีลูก	เคยแท้ง
มีเหงือกอัก	มีหินน้ำลาย	ม.ต้น	มีงานทำ	อยู่ด้วยกันก	ครอบครัวข	สิทธิหลักป	มีลูกแล้วอย	ไม่เคยแท้ง
มีเหงือกอัก	มีหินน้ำลาย	ม.ปลาย/ปว	มีงานทำ	อยู่ด้วยกันก	ครอบครัวเดี	สิทธิหลักป	มีลูกแล้วอย	ไม่เคยแท้ง
มีเหงือกอัก	มีหินน้ำลาย	ป.ตรีขึ้นไป	มีงานทำ	อยู่ด้วยกันก	ครอบครัวเดี	สิทธิประกัน	มีลูกแล้วอย	ไม่เคยแท้ง
มีเหงือกอัก	มีหินน้ำลาย	ม.ปลาย/ปว	ว่างงาน	อยู่ด้วยกันก	ครอบครัวเดี	สิทธิหลักป	มีลูกแล้วอย	ไม่เคยแท้ง
มีเหงือกอัก	มีหินน้ำลาย	ม.ปลาย/ปว	มีงานทำ	อยู่ด้วยกันก	ครอบครัวเดี	สิทธิหลักป	ไม่มีลูก	ไม่เคยแท้ง
มีเหงือกอัก	มีหินน้ำลาย	ป.ตรีขึ้นไป	มีงานทำ	อยู่ด้วยกันก	ครอบครัวข	สิทธิหลักป	มีลูกแล้วอย	ไม่เคยแท้ง
มีเหงือกอัก	มีหินน้ำลาย	ม.ปลาย/ปว	ว่างงาน	อยู่ด้วยกันก	ครอบครัวเดี	อื่นๆ	ไม่มีลูก	ไม่เคยแท้ง
มีเหงือกอัก	มีหินน้ำลาย	ม.ปลาย/ปว	มีงานทำ	อยู่ด้วยกันก	ครอบครัวเดี	สิทธิหลักป	ไม่มีลูก	ไม่เคยแท้ง
มีเหงือกอัก	มีหินน้ำลาย	ม.ต้น	ว่างงาน	อยู่ด้วยกันก	ครอบครัวเดี	สิทธิประกัน	มีลูกแล้วอย	ไม่เคยแท้ง
ไม่มีเหงือก	ไม่มีหินน้ำล	ป.ตรีขึ้นไป	ว่างงาน	อยู่ด้วยกันก	ครอบครัวเดี	สิทธิหลักป	ไม่มีลูก	ไม่เคยแท้ง
ไม่มีเหงือก	ไม่มีหินน้ำล	ม.ปลาย/ปว	มีงานทำ	อยู่ด้วยกันก	ครอบครัวเดี	สิทธิหลักป	ไม่มีลูก	ไม่เคยแท้ง
มีเหงือกอัก	มีหินน้ำลาย	ม.ปลาย/ปว	ว่างงาน	อยู่ด้วยกันก	ครอบครัวข	สิทธิหลักป	ไม่มีลูก	ไม่เคยแท้ง
ไม่มีเหงือก	มีหินน้ำลาย	ม.ปลาย/ปว	ว่างงาน	อยู่ด้วยกันก	ครอบครัวข	สิทธิหลักป	มีลูกแล้วอย	ไม่เคยแท้ง
มีเหงือกอัก	มีหินน้ำลาย	ม.ปลาย/ปว	ว่างงาน	อยู่ด้วยกันก	ครอบครัวเดี	สิทธิประกัน	มีลูกแล้วอย	ไม่เคยแท้ง
มีเหงือกอัก	มีหินน้ำลาย	ม.ปลาย/ปว	มีงานทำ	อยู่ด้วยกันก	ครอบครัวเดี	สิทธิหลักป	ไม่มีลูก	ไม่เคยแท้ง
ไม่มีเหงือก	ไม่มีหินน้ำล	ป.ตรีขึ้นไป	มีงานทำ	อยู่ด้วยกันก	ครอบครัวเดี	สิทธิหลักป	มีลูกแล้วอย	ไม่เคยแท้ง
มีเหงือกอัก	มีหินน้ำลาย	ม.ปลาย/ปว	มีงานทำ	อยู่ด้วยกันก	ครอบครัวเดี	สิทธิหลักป	มีลูกแล้วอย	ไม่เคยแท้ง
ไม่มีเหงือก	ไม่มีหินน้ำล	ป.ตรีขึ้นไป	มีงานทำ	อยู่ด้วยกันก	ครอบครัวข	สิทธิหลักป	ไม่มีลูก	ไม่เคยแท้ง
มีเหงือกอัก	มีหินน้ำลาย	ป.ตรีขึ้นไป	ว่างงาน	อยู่ด้วยกันก	ครอบครัวเดี	สิทธิหลักป	ไม่มีลูก	ไม่เคยแท้ง
มีเหงือกอัก	มีหินน้ำลาย	ม.ต้น	มีงานทำ	อยู่ด้วยกันก	ครอบครัวเดี	สิทธิหลักป	มีลูกแล้วอย	ไม่เคยแท้ง
มีเหงือกอัก	มีหินน้ำลาย	ม.ต้น	มีงานทำ	อยู่ด้วยกันก	ครอบครัวเดี	สิทธิหลักป	ไม่มีลูก	ไม่เคยแท้ง
มีเหงือกอัก	มีหินน้ำลาย	ประถมฯ	ว่างงาน	ไม่ได้อยู่กับ	ครอบครัวเดี	สิทธิประกัน	มีลูกแล้วอย	เคยแท้ง
มีเหงือกอัก	มีหินน้ำลาย	ม.ปลาย/ปว	มีงานทำ	อยู่ด้วยกันก	ครอบครัวข	สิทธิหลักป	มีลูกแล้วอย	เคยแท้ง
มีเหงือกอัก	มีหินน้ำลาย	ประถมฯ	มีงานทำ	อยู่ด้วยกันก	ครอบครัวเดี	สิทธิหลักป	มีลูกแล้วอย	ไม่เคยแท้ง

มีเหงือกอัก	มีหินน้ำลาย	ป.ตรีขึ้นไป	ว่างงาน	อยู่ด้วยกันก	ครอบครัวเ	สิทธิหลักป	ไม่มีลูก	ไม่เคยแท้ง
มีเหงือกอัก	มีหินน้ำลาย	ม.ปลาย/ปว	มีงานทำ	อยู่ด้วยกันก	ครอบครัวเ	สิทธิหลักป	มีลูกแล้วอย	ไม่เคยแท้ง
มีเหงือกอัก	มีหินน้ำลาย	ปวส.	มีงานทำ	อยู่ด้วยกันก	ครอบครัวเ	สิทธิหลักป	ไม่มีลูก	ไม่เคยแท้ง
มีเหงือกอัก	มีหินน้ำลาย	ประถมฯ	มีงานทำ	ไม่ได้อยู่กับ	ครอบครัวเ	สิทธิหลักป	มีลูกแล้วอย	ไม่เคยแท้ง
มีเหงือกอัก	มีหินน้ำลาย	ป.ตรีขึ้นไป	มีงานทำ	อยู่ด้วยกันก	ครอบครัวเ	สิทธิข้าราช	มีลูกแล้วอย	ไม่เคยแท้ง
มีเหงือกอัก	มีหินน้ำลาย	ปวส.	ว่างงาน	อยู่ด้วยกันก	ครอบครัวเ	สิทธิประกัน	ไม่มีลูก	ไม่เคยแท้ง
มีเหงือกอัก	มีหินน้ำลาย	ป.ตรีขึ้นไป	มีงานทำ	อยู่ด้วยกันก	ครอบครัวเ	อื่นๆ	มีลูกแล้วอย	ไม่เคยแท้ง
มีเหงือกอัก	มีหินน้ำลาย	ม.ต้น	มีงานทำ	อยู่ด้วยกันก	ครอบครัวเ	สิทธิประกัน	มีลูกแล้วอย	เคยแท้ง
มีเหงือกอัก	มีหินน้ำลาย	ประถมฯ	มีงานทำ	อยู่ด้วยกันก	ครอบครัวเ	สิทธิหลักป	มีลูกแล้วอย	ไม่เคยแท้ง
มีเหงือกอัก	มีหินน้ำลาย	ม.ปลาย/ปว	มีงานทำ	อยู่ด้วยกันก	ครอบครัวเ	สิทธิหลักป	มีลูกแล้วอย	ไม่เคยแท้ง
มีเหงือกอัก	มีหินน้ำลาย	ป.ตรีขึ้นไป	มีงานทำ	อยู่ด้วยกันก	ครอบครัวเ	สิทธิหลักป	มีลูกแล้วอย	ไม่เคยแท้ง
มีเหงือกอัก	มีหินน้ำลาย	ปวส.	มีงานทำ	อยู่ด้วยกันก	ครอบครัวข	สิทธิหลักป	ไม่มีลูก	ไม่เคยแท้ง
มีเหงือกอัก	มีหินน้ำลาย	ม.ปลาย/ปว	มีงานทำ	อยู่ด้วยกันก	ครอบครัวเ	สิทธิหลักป	ไม่มีลูก	ไม่เคยแท้ง
ไม่มีเหงือก	ไม่มีหินน้ำล	ม.ปลาย/ปว	มีงานทำ	อยู่ด้วยกันก	ครอบครัวข	สิทธิหลักป	มีลูกแล้วอย	ไม่เคยแท้ง
มีเหงือกอัก	มีหินน้ำลาย	ม.ต้น	มีงานทำ	อยู่ด้วยกันก	ครอบครัวเ	อื่นๆ	มีลูกแล้วอย	ไม่เคยแท้ง
มีเหงือกอัก	มีหินน้ำลาย	ม.ต้น	มีงานทำ	อยู่ด้วยกันก	ครอบครัวเ	สิทธิหลักป	ไม่มีลูก	ไม่เคยแท้ง
มีเหงือกอัก	มีหินน้ำลาย	ม.ปลาย/ปว	มีงานทำ	อยู่ด้วยกันก	ครอบครัวเ	สิทธิหลักป	มีลูกแล้วอย	เคยแท้ง
ไม่มีเหงือก	ไม่มีหินน้ำล	ป.ตรีขึ้นไป	ว่างงาน	อยู่ด้วยกันก	ครอบครัวข	สิทธิหลักป	ไม่มีลูก	ไม่เคยแท้ง
มีเหงือกอัก	มีหินน้ำลาย	ม.ปลาย/ปว	มีงานทำ	อยู่ด้วยกันก	ครอบครัวเ	สิทธิหลักป	ไม่มีลูก	ไม่เคยแท้ง
มีเหงือกอัก	มีหินน้ำลาย	ม.ต้น	มีงานทำ	อยู่ด้วยกันก	ครอบครัวเ	สิทธิหลักป	ไม่มีลูก	ไม่เคยแท้ง
ไม่มีเหงือก	ไม่มีหินน้ำล	ป.ตรีขึ้นไป	มีงานทำ	อยู่ด้วยกันก	ครอบครัวข	สิทธิหลักป	มีลูกแล้วอย	ไม่เคยแท้ง
มีเหงือกอัก	มีหินน้ำลาย	ปวส.	มีงานทำ	อยู่ด้วยกันก	ครอบครัวข	สิทธิหลักป	มีลูกแล้วอย	ไม่เคยแท้ง
มีเหงือกอัก	มีหินน้ำลาย	ปวส.	ว่างงาน	ไม่ได้อยู่กับ	ครอบครัวเ	สิทธิหลักป	ไม่มีลูก	ไม่เคยแท้ง
มีเหงือกอัก	มีหินน้ำลาย	ม.ต้น	มีงานทำ	อยู่ด้วยกันก	ครอบครัวเ	สิทธิหลักป	ไม่มีลูก	ไม่เคยแท้ง
มีเหงือกอัก	มีหินน้ำลาย	ม.ต้น	มีงานทำ	อยู่ด้วยกันก	ครอบครัวข	สิทธิหลักป	มีลูกแล้วอย	ไม่เคยแท้ง
มีเหงือกอัก	มีหินน้ำลาย	ม.ต้น	มีงานทำ	อยู่ด้วยกันก	ครอบครัวเ	สิทธิหลักป	ไม่มีลูก	ไม่เคยแท้ง
มีเหงือกอัก	มีหินน้ำลาย	ประถมฯ	ว่างงาน	อยู่ด้วยกันก	ครอบครัวเ	สิทธิหลักป	มีลูกแล้วอย	ไม่เคยแท้ง
มีเหงือกอัก	มีหินน้ำลาย	ม.ปลาย/ปว	ว่างงาน	อยู่ด้วยกันก	ครอบครัวข	สิทธิหลักป	ไม่มีลูก	ไม่เคยแท้ง
มีเหงือกอัก	มีหินน้ำลาย	ม.ต้น	มีงานทำ	อยู่ด้วยกันก	ครอบครัวเ	สิทธิหลักป	ไม่มีลูก	ไม่เคยแท้ง
มีเหงือกอัก	มีหินน้ำลาย	ม.ปลาย/ปว	มีงานทำ	อยู่ด้วยกันก	ครอบครัวเ	สิทธิประกัน	ไม่มีลูก	ไม่เคยแท้ง
มีเหงือกอัก	มีหินน้ำลาย	ม.ต้น	มีงานทำ	อยู่ด้วยกันก	ครอบครัวข	สิทธิหลักป	มีลูกแล้วอย	ไม่เคยแท้ง
มีเหงือกอัก	มีหินน้ำลาย	ม.ต้น	มีงานทำ	อยู่ด้วยกันก	ครอบครัวเ	สิทธิหลักป	ไม่มีลูก	ไม่เคยแท้ง
มีเหงือกอัก	มีหินน้ำลาย	ม.ปลาย/ปว	ว่างงาน	อยู่ด้วยกันก	ครอบครัวเ	สิทธิหลักป	มีลูกแล้วอย	ไม่เคยแท้ง
ไม่มีเหงือก	ไม่มีหินน้ำล	ม.ปลาย/ปว	ว่างงาน	อยู่ด้วยกันก	ครอบครัวเ	สิทธิหลักป	ไม่มีลูก	ไม่เคยแท้ง
มีเหงือกอัก	มีหินน้ำลาย	ปวส.	ว่างงาน	อยู่ด้วยกันก	ครอบครัวเ	สิทธิข้าราช	ไม่มีลูก	ไม่เคยแท้ง
มีเหงือกอัก	มีหินน้ำลาย	ม.ปลาย/ปว	มีงานทำ	อยู่ด้วยกันก	ครอบครัวเ	สิทธิหลักป	ไม่มีลูก	ไม่เคยแท้ง
มีเหงือกอัก	มีหินน้ำลาย	ม.ปลาย/ปว	ว่างงาน	อยู่ด้วยกันก	ครอบครัวข	สิทธิหลักป	ไม่มีลูก	ไม่เคยแท้ง
มีเหงือกอัก	มีหินน้ำลาย	ม.ปลาย/ปว	มีงานทำ	อยู่ด้วยกันก	ครอบครัวเ	สิทธิหลักป	มีลูกแล้วอย	ไม่เคยแท้ง
มีเหงือกอัก	มีหินน้ำลาย	ป.ตรีขึ้นไป	มีงานทำ	อยู่ด้วยกันก	ครอบครัวเ	สิทธิประกัน	มีลูกแล้วอย	ไม่เคยแท้ง
ไม่มีเหงือก	ไม่มีหินน้ำล	ปวส.	ว่างงาน	อยู่ด้วยกันก	ครอบครัวเ	สิทธิหลักป	มีลูกแล้วอย	ไม่เคยแท้ง
มีเหงือกอัก	มีหินน้ำลาย	ปวส.	มีงานทำ	อยู่ด้วยกันก	ครอบครัวเ	สิทธิหลักป	มีลูกแล้วอย	ไม่เคยแท้ง
มีเหงือกอัก	มีหินน้ำลาย	ม.ปลาย/ปว	ว่างงาน	อยู่ด้วยกันก	ครอบครัวเ	สิทธิหลักป	ไม่มีลูก	ไม่เคยแท้ง
มีเหงือกอัก	มีหินน้ำลาย	ม.ปลาย/ปว	มีงานทำ	อยู่ด้วยกันก	ครอบครัวเ	สิทธิประกัน	ไม่มีลูก	ไม่เคยแท้ง
มีเหงือกอัก	มีหินน้ำลาย	ปวส.	มีงานทำ	ไม่ได้อยู่กับ	ครอบครัวเ	สิทธิหลักป	มีลูกแล้วอย	ไม่เคยแท้ง
มีเหงือกอัก	มีหินน้ำลาย	ม.ปลาย/ปว	มีงานทำ	อยู่ด้วยกันก	ครอบครัวเ	สิทธิหลักป	มีลูกแล้วอย	ไม่เคยแท้ง
มีเหงือกอัก	มีหินน้ำลาย	ป.ตรีขึ้นไป	มีงานทำ	อยู่ด้วยกันก	ครอบครัวข	สิทธิข้าราช	มีลูกแล้วอย	ไม่เคยแท้ง
มีเหงือกอัก	มีหินน้ำลาย	ประถมฯ	ว่างงาน	อยู่ด้วยกันก	ครอบครัวเ	สิทธิหลักป	มีลูกแล้วอย	เคยแท้ง
มีเหงือกอัก	มีหินน้ำลาย	ม.ต้น	มีงานทำ	อยู่ด้วยกันก	ครอบครัวเ	สิทธิหลักป	มีลูกแล้วอย	ไม่เคยแท้ง
ไม่มีเหงือก	ไม่มีหินน้ำล	ปวส.	ว่างงาน	อยู่ด้วยกันก	ครอบครัวเ	สิทธิหลักป	ไม่มีลูก	ไม่เคยแท้ง
มีเหงือกอัก	มีหินน้ำลาย	ม.ปลาย/ปว	ว่างงาน	ไม่ได้อยู่กับ	ครอบครัวเ	สิทธิหลักป	ไม่มีลูก	ไม่เคยแท้ง
มีเหงือกอัก	มีหินน้ำลาย	ม.ปลาย/ปว	มีงานทำ	อยู่ด้วยกันก	ครอบครัวเ	สิทธิหลักป	ไม่มีลูก	ไม่เคยแท้ง

มีเหงือกอัก	มีหินน้ำลาย	ประถมฯ	มีงานทำ	อยู่ด้วยกันก	ครอบครัวเ	สิทธิหลักป	มีลูกแล้วอย	ไม่เคยแท้ง
ไม่มีเหงือก	ไม่มีหินน้ำล	ม.ปลาย/ปว	มีงานทำ	อยู่ด้วยกันก	ครอบครัวเ	สิทธิหลักป	มีลูกแล้วอย	ไม่เคยแท้ง
ไม่มีเหงือก	ไม่มีหินน้ำล	ม.ปลาย/ปว	มีงานทำ	อยู่ด้วยกันก	ครอบครัวเ	สิทธิหลักป	ไม่มีลูก	ไม่เคยแท้ง
มีเหงือกอัก	มีหินน้ำลาย	ม.ปลาย/ปว	มีงานทำ	อยู่ด้วยกันก	ครอบครัวเ	สิทธิหลักป	ไม่มีลูก	ไม่เคยแท้ง
มีเหงือกอัก	มีหินน้ำลาย	ม.ต้น	มีงานทำ	อยู่ด้วยกันก	ครอบครัวเ	สิทธิหลักป	มีลูกแล้วอย	เคยแท้ง
มีเหงือกอัก	มีหินน้ำลาย	ป.ตรีขึ้นไป	มีงานทำ	อยู่ด้วยกันก	ครอบครัวข	สิทธิข้าราช	มีลูกแล้วอย	ไม่เคยแท้ง
มีเหงือกอัก	มีหินน้ำลาย	ปวส.	ว่างงาน	อยู่ด้วยกันก	ครอบครัวเ	สิทธิหลักป	มีลูกแล้วอย	ไม่เคยแท้ง
มีเหงือกอัก	มีหินน้ำลาย	ม.ต้น	มีงานทำ	อยู่ด้วยกันก	ครอบครัวเ	สิทธิประกัน	มีลูกแล้วอย	ไม่เคยแท้ง
มีเหงือกอัก	มีหินน้ำลาย	ปวส.	มีงานทำ	อยู่ด้วยกันก	ครอบครัวเ	สิทธิหลักป	มีลูกแล้วอย	ไม่เคยแท้ง
มีเหงือกอัก	มีหินน้ำลาย	ปวส.	มีงานทำ	อยู่ด้วยกันก	ครอบครัวเ	สิทธิหลักป	มีลูกแล้วอย	ไม่เคยแท้ง
มีเหงือกอัก	มีหินน้ำลาย	ประถมฯ	มีงานทำ	อยู่ด้วยกันก	ครอบครัวเ	สิทธิหลักป	มีลูกแล้วอย	ไม่เคยแท้ง
มีเหงือกอัก	มีหินน้ำลาย	ม.ปลาย/ปว	มีงานทำ	อยู่ด้วยกันก	ครอบครัวข	สิทธิหลักป	มีลูกแล้วอย	ไม่เคยแท้ง
มีเหงือกอัก	มีหินน้ำลาย	ม.ปลาย/ปว	มีงานทำ	อยู่ด้วยกันก	ครอบครัวข	สิทธิหลักป	มีลูกแล้วอย	ไม่เคยแท้ง
มีเหงือกอัก	มีหินน้ำลาย	ป.ตรีขึ้นไป	มีงานทำ	อยู่ด้วยกันก	ครอบครัวเ	สิทธิหลักป	ไม่มีลูก	ไม่เคยแท้ง
มีเหงือกอัก	มีหินน้ำลาย	ม.ปลาย/ปว	มีงานทำ	อยู่ด้วยกันก	ครอบครัวเ	สิทธิหลักป	ไม่มีลูก	ไม่เคยแท้ง
มีเหงือกอัก	มีหินน้ำลาย	ม.ปลาย/ปว	มีงานทำ	อยู่ด้วยกันก	ครอบครัวเ	สิทธิหลักป	มีลูกแล้วอย	ไม่เคยแท้ง
มีเหงือกอัก	มีหินน้ำลาย	ม.ปลาย/ปว	มีงานทำ	อยู่ด้วยกันก	ครอบครัวเ	สิทธิหลักป	มีลูกแล้วอย	ไม่เคยแท้ง
มีเหงือกอัก	มีหินน้ำลาย	ม.ต้น	ว่างงาน	อยู่ด้วยกันก	ครอบครัวข	สิทธิหลักป	ไม่มีลูก	ไม่เคยแท้ง
มีเหงือกอัก	มีหินน้ำลาย	ปวส.	มีงานทำ	อยู่ด้วยกันก	ครอบครัวเ	สิทธิหลักป	มีลูกแล้วอย	ไม่เคยแท้ง
มีเหงือกอัก	มีหินน้ำลาย	ปวส.	มีงานทำ	อยู่ด้วยกันก	ครอบครัวเ	สิทธิหลักป	มีลูกแล้วอย	ไม่เคยแท้ง
มีเหงือกอัก	มีหินน้ำลาย	ป.ตรีขึ้นไป	มีงานทำ	อยู่ด้วยกันก	ครอบครัวข	สิทธิประกัน	ไม่มีลูก	ไม่เคยแท้ง
มีเหงือกอัก	มีหินน้ำลาย	ปวส.	มีงานทำ	อยู่ด้วยกันก	ครอบครัวข	สิทธิหลักป	ไม่มีลูก	ไม่เคยแท้ง
มีเหงือกอัก	มีหินน้ำลาย	ม.ปลาย/ปว	ว่างงาน	อยู่ด้วยกันก	ครอบครัวเ	สิทธิหลักป	มีลูกแล้วอย	เคยแท้ง
มีเหงือกอัก	มีหินน้ำลาย	ม.ปลาย/ปว	มีงานทำ	อยู่ด้วยกันก	ครอบครัวเ	สิทธิหลักป	มีลูกแล้วอย	ไม่เคยแท้ง
มีเหงือกอัก	มีหินน้ำลาย	ปวส.	ว่างงาน	ไม่ได้อยู่กับ	ครอบครัวข	สิทธิหลักป	ไม่มีลูก	ไม่เคยแท้ง
มีเหงือกอัก	มีหินน้ำลาย	ม.ต้น	มีงานทำ	ไม่ได้อยู่กับ	ครอบครัวเ	สิทธิหลักป	มีลูกแล้วอย	ไม่เคยแท้ง
มีเหงือกอัก	มีหินน้ำลาย	ม.ปลาย/ปว	ว่างงาน	อยู่ด้วยกันก	ครอบครัวข	สิทธิหลักป	ไม่มีลูก	ไม่เคยแท้ง
มีเหงือกอัก	มีหินน้ำลาย	ม.ปลาย/ปว	มีงานทำ	อยู่ด้วยกันก	ครอบครัวเ	สิทธิประกัน	มีลูกแล้วอย	ไม่เคยแท้ง
ไม่มีเหงือก	ไม่มีหินน้ำล	ป.ตรีขึ้นไป	มีงานทำ	อยู่ด้วยกันก	ครอบครัวข	สิทธิข้าราช	มีลูกแล้วอย	ไม่เคยแท้ง
มีเหงือกอัก	มีหินน้ำลาย	ป.ตรีขึ้นไป	มีงานทำ	อยู่ด้วยกันก	ครอบครัวเ	สิทธิหลักป	มีลูกแล้วอย	ไม่เคยแท้ง
มีเหงือกอัก	มีหินน้ำลาย	ม.ปลาย/ปว	มีงานทำ	อยู่ด้วยกันก	ครอบครัวเ	สิทธิประกัน	ไม่มีลูก	ไม่เคยแท้ง
มีเหงือกอัก	มีหินน้ำลาย	ป.ตรีขึ้นไป	มีงานทำ	อยู่ด้วยกันก	ครอบครัวเ	สิทธิหลักป	ไม่มีลูก	ไม่เคยแท้ง
มีเหงือกอัก	มีหินน้ำลาย	ปวส.	มีงานทำ	อยู่ด้วยกันก	ครอบครัวเ	สิทธิประกัน	มีลูกแล้วอย	เคยแท้ง
มีเหงือกอัก	มีหินน้ำลาย	ป.ตรีขึ้นไป	มีงานทำ	อยู่ด้วยกันก	ครอบครัวเ	สิทธิประกัน	มีลูกแล้วอย	ไม่เคยแท้ง
มีเหงือกอัก	มีหินน้ำลาย	ม.ปลาย/ปว	ว่างงาน	อยู่ด้วยกันก	ครอบครัวข	สิทธิหลักป	ไม่มีลูก	ไม่เคยแท้ง
มีเหงือกอัก	มีหินน้ำลาย	ป.ตรีขึ้นไป	มีงานทำ	อยู่ด้วยกันก	ครอบครัวเ	สิทธิข้าราช	ไม่มีลูก	ไม่เคยแท้ง
มีเหงือกอัก	มีหินน้ำลาย	ม.ต้น	ว่างงาน	อยู่ด้วยกันก	ครอบครัวเ	สิทธิหลักป	ไม่มีลูก	ไม่เคยแท้ง
มีเหงือกอัก	มีหินน้ำลาย	ป.ตรีขึ้นไป	มีงานทำ	อยู่ด้วยกันก	ครอบครัวเ	สิทธิหลักป	มีลูกแล้วอย	ไม่เคยแท้ง
มีเหงือกอัก	มีหินน้ำลาย	ปวส.	ว่างงาน	อยู่ด้วยกันก	ครอบครัวเ	สิทธิหลักป	ไม่มีลูก	ไม่เคยแท้ง
มีเหงือกอัก	มีหินน้ำลาย	ปวส.	มีงานทำ	อยู่ด้วยกันก	ครอบครัวเ	สิทธิประกัน	มีลูกแล้วอย	ไม่เคยแท้ง
มีเหงือกอัก	มีหินน้ำลาย	ปวส.	มีงานทำ	อยู่ด้วยกันก	ครอบครัวเ	สิทธิหลักป	มีลูกแล้วอย	ไม่เคยแท้ง
มีเหงือกอัก	มีหินน้ำลาย	ปวส.	มีงานทำ	อยู่ด้วยกันก	ครอบครัวข	สิทธิหลักป	ไม่มีลูก	ไม่เคยแท้ง
ไม่มีเหงือก	ไม่มีหินน้ำล	ม.ปลาย/ปว	มีงานทำ	อยู่ด้วยกันก	ครอบครัวเ	สิทธิหลักป	ไม่มีลูก	ไม่เคยแท้ง
มีเหงือกอัก	มีหินน้ำลาย	ม.ปลาย/ปว	มีงานทำ	อยู่ด้วยกันก	ครอบครัวเ	สิทธิหลักป	ไม่มีลูก	ไม่เคยแท้ง
มีเหงือกอัก	มีหินน้ำลาย	ม.ต้น	ว่างงาน	อยู่ด้วยกันก	ครอบครัวเ	สิทธิหลักป	ไม่มีลูก	ไม่เคยแท้ง
มีเหงือกอัก	มีหินน้ำลาย	ป.ตรีขึ้นไป	มีงานทำ	อยู่ด้วยกันก	ครอบครัวข	สิทธิหลักป	ไม่มีลูก	ไม่เคยแท้ง
มีเหงือกอัก	มีหินน้ำลาย	ปวส.	มีงานทำ	อยู่ด้วยกันก	ครอบครัวข	สิทธิหลักป	ไม่มีลูก	ไม่เคยแท้ง
มีเหงือกอัก	มีหินน้ำลาย	ม.ปลาย/ปว	มีงานทำ	อยู่ด้วยกันก	ครอบครัวข	สิทธิหลักป	ไม่มีลูก	ไม่เคยแท้ง
มีเหงือกอัก	มีหินน้ำลาย	ป.ตรีขึ้นไป	มีงานทำ	อยู่ด้วยกันก	ครอบครัวข	สิทธิประกัน	ไม่มีลูก	ไม่เคยแท้ง
มีเหงือกอัก	มีหินน้ำลาย	ม.ปลาย/ปว	มีงานทำ	อยู่ด้วยกันก	ครอบครัวข	สิทธิหลักป	ไม่มีลูก	ไม่เคยแท้ง
มีเหงือกอัก	มีหินน้ำลาย	ม.ปลาย/ปว	มีงานทำ	อยู่ด้วยกันก	ครอบครัวเ	สิทธิหลักป	ไม่มีลูก	ไม่เคยแท้ง

ไม่มีเหงือก	ไม่มีหินน้ำล	ม.ปลาย/ปว	ว่างงาน	อยู่ด้วยกันก	ครอบครัวเ	สิทธิหลักป	มีลูกแล้วอย	ไม่เคยแท้ง
มีเหงือกอัก	มีหินน้ำลาย	ป.ตรีขึ้นไป	มีงานทำ	อยู่ด้วยกันก	ครอบครัวข	สิทธิประกัน	มีลูกแล้วอย	ไม่เคยแท้ง
มีเหงือกอัก	มีหินน้ำลาย	ป.ตรีขึ้นไป	มีงานทำ	อยู่ด้วยกันก	ครอบครัวเ	สิทธิข้าราช	มีลูกแล้วอย	ไม่เคยแท้ง
ไม่มีเหงือก	ไม่มีหินน้ำล	ม.ต้น	ว่างงาน	อยู่ด้วยกันก	ครอบครัวเ	สิทธิประกัน	ไม่มีลูก	เคยแท้ง
มีเหงือกอัก	มีหินน้ำลาย	ปวส.	มีงานทำ	อยู่ด้วยกันก	ครอบครัวเ	สิทธิหลักป	ไม่มีลูก	ไม่เคยแท้ง
มีเหงือกอัก	มีหินน้ำลาย	ม.ปลาย/ปว	ว่างงาน	อยู่ด้วยกันก	ครอบครัวข	สิทธิประกัน	ไม่มีลูก	ไม่เคยแท้ง
มีเหงือกอัก	มีหินน้ำลาย	ป.ตรีขึ้นไป	มีงานทำ	อยู่ด้วยกันก	ครอบครัวเ	สิทธิประกัน	ไม่มีลูก	ไม่เคยแท้ง
มีเหงือกอัก	มีหินน้ำลาย	ม.ต้น	มีงานทำ	อยู่ด้วยกันก	ครอบครัวเ	สิทธิประกัน	มีลูกแล้วอย	ไม่เคยแท้ง
มีเหงือกอัก	มีหินน้ำลาย	ป.ตรีขึ้นไป	มีงานทำ	อยู่ด้วยกันก	ครอบครัวข	สิทธิหลักป	ไม่มีลูก	ไม่เคยแท้ง
มีเหงือกอัก	มีหินน้ำลาย	ม.ปลาย/ปว	ว่างงาน	อยู่ด้วยกันก	ครอบครัวเ	สิทธิหลักป	มีลูกแล้วอย	ไม่เคยแท้ง
มีเหงือกอัก	มีหินน้ำลาย	ประถมฯ	มีงานทำ	อยู่ด้วยกันก	ครอบครัวเ	สิทธิข้าราช	ไม่มีลูก	ไม่เคยแท้ง
มีเหงือกอัก	มีหินน้ำลาย	ม.ปลาย/ปว	มีงานทำ	อยู่ด้วยกันก	ครอบครัวข	สิทธิหลักป	มีลูกแล้วอย	ไม่เคยแท้ง
มีเหงือกอัก	มีหินน้ำลาย	ประถมฯ	ว่างงาน	อยู่ด้วยกันก	ครอบครัวเ	สิทธิหลักป	มีลูกแล้วอย	เคยแท้ง
มีเหงือกอัก	มีหินน้ำลาย	ปวส.	มีงานทำ	อยู่ด้วยกันก	ครอบครัวเ	สิทธิหลักป	มีลูกแล้วอย	เคยแท้ง
มีเหงือกอัก	มีหินน้ำลาย	ป.ตรีขึ้นไป	มีงานทำ	อยู่ด้วยกันก	ครอบครัวเ	สิทธิหลักป	ไม่มีลูก	ไม่เคยแท้ง
มีเหงือกอัก	มีหินน้ำลาย	ม.ปลาย/ปว	ว่างงาน	อยู่ด้วยกันก	ครอบครัวเ	สิทธิหลักป	ไม่มีลูก	ไม่เคยแท้ง
มีเหงือกอัก	มีหินน้ำลาย	ม.ปลาย/ปว	ว่างงาน	อยู่ด้วยกันก	ครอบครัวข	สิทธิหลักป	ไม่มีลูก	ไม่เคยแท้ง
มีเหงือกอัก	มีหินน้ำลาย	ป.ตรีขึ้นไป	มีงานทำ	อยู่ด้วยกันก	ครอบครัวเ	สิทธิข้าราช	มีลูกแล้วอย	ไม่เคยแท้ง
มีเหงือกอัก	มีหินน้ำลาย	ป.ตรีขึ้นไป	มีงานทำ	อยู่ด้วยกันก	ครอบครัวข	สิทธิหลักป	ไม่มีลูก	ไม่เคยแท้ง
มีเหงือกอัก	มีหินน้ำลาย	ม.ต้น	มีงานทำ	อยู่ด้วยกันก	ครอบครัวเ	สิทธิหลักป	มีลูกแล้วอย	ไม่เคยแท้ง
ไม่มีเหงือก	ไม่มีหินน้ำล	ม.ปลาย/ปว	ว่างงาน	อยู่ด้วยกันก	ครอบครัวเ	สิทธิหลักป	ไม่มีลูก	ไม่เคยแท้ง
มีเหงือกอัก	มีหินน้ำลาย	ม.ปลาย/ปว	ว่างงาน	อยู่ด้วยกันก	ครอบครัวข	สิทธิหลักป	ไม่มีลูก	ไม่เคยแท้ง
มีเหงือกอัก	มีหินน้ำลาย	ป.ตรีขึ้นไป	มีงานทำ	อยู่ด้วยกันก	ครอบครัวเ	สิทธิประกัน	มีลูกแล้วอย	ไม่เคยแท้ง
ไม่มีเหงือก	ไม่มีหินน้ำล	ปวส.	ว่างงาน	อยู่ด้วยกันก	ครอบครัวเ	สิทธิหลักป	มีลูกแล้วอย	เคยแท้ง
มีเหงือกอัก	มีหินน้ำลาย	ป.ตรีขึ้นไป	มีงานทำ	ไม่ได้อยู่กับ	ครอบครัวเ	สิทธิข้าราช	ไม่มีลูก	ไม่เคยแท้ง
มีเหงือกอัก	มีหินน้ำลาย	ป.ตรีขึ้นไป	มีงานทำ	อยู่ด้วยกันก	ครอบครัวเ	สิทธิข้าราช	ไม่มีลูก	ไม่เคยแท้ง
มีเหงือกอัก	มีหินน้ำลาย	ม.ต้น	ว่างงาน	อยู่ด้วยกันก	ครอบครัวเ	สิทธิหลักป	มีลูกแล้วอย	ไม่เคยแท้ง
มีเหงือกอัก	มีหินน้ำลาย	ป.ตรีขึ้นไป	มีงานทำ	อยู่ด้วยกันก	ครอบครัวข	สิทธิประกัน	มีลูกแล้วอย	เคยแท้ง
มีเหงือกอัก	มีหินน้ำลาย	ป.ตรีขึ้นไป	มีงานทำ	อยู่ด้วยกันก	ครอบครัวเ	สิทธิหลักป	ไม่มีลูก	ไม่เคยแท้ง
มีเหงือกอัก	มีหินน้ำลาย	ม.ปลาย/ปว	มีงานทำ	อยู่ด้วยกันก	ครอบครัวข	สิทธิหลักป	ไม่มีลูก	ไม่เคยแท้ง
มีเหงือกอัก	มีหินน้ำลาย	ปวส.	ว่างงาน	ไม่ได้อยู่กับ	ครอบครัวข	สิทธิหลักป	ไม่มีลูก	ไม่เคยแท้ง
มีเหงือกอัก	มีหินน้ำลาย	ป.ตรีขึ้นไป	มีงานทำ	อยู่ด้วยกันก	ครอบครัวเ	สิทธิประกัน	มีลูกแล้วอย	ไม่เคยแท้ง
มีเหงือกอัก	มีหินน้ำลาย	ป.ตรีขึ้นไป	มีงานทำ	อยู่ด้วยกันก	ครอบครัวข	สิทธิประกัน	ไม่มีลูก	ไม่เคยแท้ง
มีเหงือกอัก	มีหินน้ำลาย	ม.ต้น	ว่างงาน	อยู่ด้วยกันก	ครอบครัวเ	สิทธิประกัน	มีลูกแล้วอย	ไม่เคยแท้ง
ไม่มีเหงือก	ไม่มีหินน้ำล	ปวส.	มีงานทำ	อยู่ด้วยกันก	ครอบครัวเ	สิทธิหลักป	ไม่มีลูก	ไม่เคยแท้ง
มีเหงือกอัก	มีหินน้ำลาย	ม.ปลาย/ปว	ว่างงาน	อยู่ด้วยกันก	ครอบครัวข	สิทธิประกัน	ไม่มีลูก	ไม่เคยแท้ง
มีเหงือกอัก	มีหินน้ำลาย	ป.ตรีขึ้นไป	มีงานทำ	อยู่ด้วยกันก	ครอบครัวเ	สิทธิประกัน	ไม่มีลูก	ไม่เคยแท้ง
ไม่มีเหงือก	ไม่มีหินน้ำล	ม.ต้น	มีงานทำ	อยู่ด้วยกันก	ครอบครัวเ	สิทธิประกัน	ไม่มีลูก	ไม่เคยแท้ง
มีเหงือกอัก	มีหินน้ำลาย	ป.ตรีขึ้นไป	มีงานทำ	อยู่ด้วยกันก	ครอบครัวข	สิทธิหลักป	ไม่มีลูก	ไม่เคยแท้ง
มีเหงือกอัก	มีหินน้ำลาย	ม.ปลาย/ปว	ว่างงาน	อยู่ด้วยกันก	ครอบครัวเ	สิทธิหลักป	มีลูกแล้วอย	ไม่เคยแท้ง
มีเหงือกอัก	มีหินน้ำลาย	ป.ตรีขึ้นไป	มีงานทำ	อยู่ด้วยกันก	ครอบครัวเ	สิทธิข้าราช	ไม่มีลูก	ไม่เคยแท้ง
ไม่มีเหงือก	ไม่มีหินน้ำล	ม.ปลาย/ปว	มีงานทำ	อยู่ด้วยกันก	ครอบครัวข	สิทธิหลักป	มีลูกแล้วอย	ไม่เคยแท้ง
มีเหงือกอัก	มีหินน้ำลาย	ประถมฯ	ว่างงาน	อยู่ด้วยกันก	ครอบครัวเ	สิทธิหลักป	มีลูกแล้วอย	ไม่เคยแท้ง
มีเหงือกอัก	มีหินน้ำลาย	ปวส.	มีงานทำ	อยู่ด้วยกันก	ครอบครัวเ	สิทธิหลักป	มีลูกแล้วอย	ไม่เคยแท้ง
มีเหงือกอัก	มีหินน้ำลาย	ป.ตรีขึ้นไป	มีงานทำ	อยู่ด้วยกันก	ครอบครัวเ	สิทธิหลักป	ไม่มีลูก	ไม่เคยแท้ง
มีเหงือกอัก	มีหินน้ำลาย	ม.ปลาย/ปว	ว่างงาน	อยู่ด้วยกันก	ครอบครัวเ	สิทธิหลักป	ไม่มีลูก	ไม่เคยแท้ง
มีเหงือกอัก	มีหินน้ำลาย	ม.ปลาย/ปว	ว่างงาน	อยู่ด้วยกันก	ครอบครัวข	สิทธิหลักป	ไม่มีลูก	ไม่เคยแท้ง
มีเหงือกอัก	มีหินน้ำลาย	ป.ตรีขึ้นไป	มีงานทำ	อยู่ด้วยกันก	ครอบครัวเ	สิทธิข้าราช	มีลูกแล้วอย	ไม่เคยแท้ง
มีเหงือกอัก	มีหินน้ำลาย	ป.ตรีขึ้นไป	มีงานทำ	อยู่ด้วยกันก	ครอบครัวข	สิทธิหลักป	มีลูกแล้วอย	ไม่เคยแท้ง
มีเหงือกอัก	มีหินน้ำลาย	ม.ต้น	มีงานทำ	อยู่ด้วยกันก	ครอบครัวเ	สิทธิหลักป	มีลูกแล้วอย	ไม่เคยแท้ง
มีเหงือกอัก	มีหินน้ำลาย	ม.ปลาย/ปว	ว่างงาน	อยู่ด้วยกันก	ครอบครัวเ	สิทธิหลักป	ไม่มีลูก	ไม่เคยแท้ง

มีเหงือกอัก	มีหินน้ำลาย	ม.ปลาย/ปว	ว่างงาน	อยู่ด้วยกันก	ครอบครัวข	สิทธิหลักป	ไม่มีลูก	ไม่เคยแท้ง
มีเหงือกอัก	มีหินน้ำลาย	ป.ตรีขึ้นไป	มีงานทำ	อยู่ด้วยกันก	ครอบครัวเดี	สิทธิประกัน	มีลูกแล้วอย	ไม่เคยแท้ง
ไม่มีเหงือก	ไม่มีหินน้ำล	ปวส.	ว่างงาน	อยู่ด้วยกันก	ครอบครัวเดี	สิทธิหลักป	มีลูกแล้วอย	เคยแท้ง
มีเหงือกอัก	มีหินน้ำลาย	ปวส.	มีงานทำ	ไม่ได้อยู่กับ	ครอบครัวเดี	สิทธิข้าราช	ไม่มีลูก	ไม่เคยแท้ง
มีเหงือกอัก	มีหินน้ำลาย	ป.ตรีขึ้นไป	มีงานทำ	อยู่ด้วยกันก	ครอบครัวเดี	สิทธิข้าราช	มีลูกแล้วอย	ไม่เคยแท้ง
มีเหงือกอัก	มีหินน้ำลาย	ม.ปลาย/ปว	ว่างงาน	อยู่ด้วยกันก	ครอบครัวเดี	สิทธิหลักป	ไม่มีลูก	ไม่เคยแท้ง
มีเหงือกอัก	มีหินน้ำลาย	ประถมฯ	มีงานทำ	อยู่ด้วยกันก	ครอบครัวเดี	สิทธิหลักป	มีลูกแล้วอย	ไม่เคยแท้ง
มีเหงือกอัก	มีหินน้ำลาย	ป.ตรีขึ้นไป	มีงานทำ	อยู่ด้วยกันก	ครอบครัวเดี	สิทธิข้าราช	ไม่มีลูก	ไม่เคยแท้ง
มีเหงือกอัก	มีหินน้ำลาย	ม.ปลาย/ปว	มีงานทำ	อยู่ด้วยกันก	ครอบครัวเดี	สิทธิหลักป	มีลูกแล้วอย	ไม่เคยแท้ง
มีเหงือกอัก	มีหินน้ำลาย	ม.ต้น	มีงานทำ	อยู่ด้วยกันก	ครอบครัวข	สิทธิประกัน	ไม่มีลูก	ไม่เคยแท้ง
ไม่มีเหงือก	ไม่มีหินน้ำล	ม.ต้น	ว่างงาน	อยู่ด้วยกันก	ครอบครัวเดี	สิทธิประกัน	ไม่มีลูก	เคยแท้ง
มีเหงือกอัก	มีหินน้ำลาย	ปวส.	มีงานทำ	อยู่ด้วยกันก	ครอบครัวเดี	สิทธิหลักป	ไม่มีลูก	ไม่เคยแท้ง
มีเหงือกอัก	มีหินน้ำลาย	ม.ปลาย/ปว	ว่างงาน	อยู่ด้วยกันก	ครอบครัวข	สิทธิประกัน	ไม่มีลูก	ไม่เคยแท้ง
มีเหงือกอัก	มีหินน้ำลาย	ป.ตรีขึ้นไป	มีงานทำ	อยู่ด้วยกันก	ครอบครัวเดี	สิทธิประกัน	ไม่มีลูก	ไม่เคยแท้ง
มีเหงือกอัก	มีหินน้ำลาย	ม.ต้น	มีงานทำ	อยู่ด้วยกันก	ครอบครัวเดี	สิทธิประกัน	ไม่มีลูก	ไม่เคยแท้ง
มีเหงือกอัก	มีหินน้ำลาย	ป.ตรีขึ้นไป	มีงานทำ	อยู่ด้วยกันก	ครอบครัวข	สิทธิหลักป	ไม่มีลูก	ไม่เคยแท้ง
มีเหงือกอัก	มีหินน้ำลาย	ม.ปลาย/ปว	ว่างงาน	อยู่ด้วยกันก	ครอบครัวเดี	สิทธิหลักป	มีลูกแล้วอย	ไม่เคยแท้ง
มีเหงือกอัก	มีหินน้ำลาย	ป.ตรีขึ้นไป	มีงานทำ	อยู่ด้วยกันก	ครอบครัวเดี	สิทธิข้าราช	ไม่มีลูก	ไม่เคยแท้ง
ไม่มีเหงือก	ไม่มีหินน้ำล	ม.ปลาย/ปว	มีงานทำ	อยู่ด้วยกันก	ครอบครัวข	สิทธิหลักป	มีลูกแล้วอย	ไม่เคยแท้ง
มีเหงือกอัก	มีหินน้ำลาย	ประถมฯ	ว่างงาน	อยู่ด้วยกันก	ครอบครัวเดี	สิทธิหลักป	มีลูกแล้วอย	เคยแท้ง
ไม่มีเหงือก	ไม่มีหินน้ำล	ปวส.	มีงานทำ	อยู่ด้วยกันก	ครอบครัวเดี	สิทธิหลักป	มีลูกแล้วอย	ไม่เคยแท้ง
มีเหงือกอัก	มีหินน้ำลาย	ป.ตรีขึ้นไป	มีงานทำ	อยู่ด้วยกันก	ครอบครัวเดี	สิทธิหลักป	ไม่มีลูก	ไม่เคยแท้ง
มีเหงือกอัก	มีหินน้ำลาย	ม.ปลาย/ปว	ว่างงาน	อยู่ด้วยกันก	ครอบครัวเดี	สิทธิหลักป	ไม่มีลูก	ไม่เคยแท้ง
มีเหงือกอัก	มีหินน้ำลาย	ม.ปลาย/ปว	ว่างงาน	อยู่ด้วยกันก	ครอบครัวข	สิทธิหลักป	ไม่มีลูก	ไม่เคยแท้ง
มีเหงือกอัก	มีหินน้ำลาย	ม.ปลาย/ปว	มีงานทำ	อยู่ด้วยกันก	ครอบครัวข	สิทธิหลักป	มีลูกแล้วอย	ไม่เคยแท้ง
มีเหงือกอัก	มีหินน้ำลาย	ม.ต้น	ว่างงาน	อยู่ด้วยกันก	ครอบครัวเดี	สิทธิหลักป	ไม่มีลูก	ไม่เคยแท้ง
มีเหงือกอัก	มีหินน้ำลาย	ป.ตรีขึ้นไป	มีงานทำ	อยู่ด้วยกันก	ครอบครัวเดี	สิทธิข้าราช	ไม่มีลูก	ไม่เคยแท้ง
มีเหงือกอัก	มีหินน้ำลาย	ม.ต้น	มีงานทำ	อยู่ด้วยกันก	ครอบครัวข	สิทธิหลักป	ไม่มีลูก	ไม่เคยแท้ง
มีเหงือกอัก	มีหินน้ำลาย	ป.ตรีขึ้นไป	มีงานทำ	อยู่ด้วยกันก	ครอบครัวเดี	สิทธิประกัน	มีลูกแล้วอย	ไม่เคยแท้ง
มีเหงือกอัก	มีหินน้ำลาย	ประถมฯ	มีงานทำ	อยู่ด้วยกันก	ครอบครัวข	สิทธิหลักป	มีลูกแล้วอย	ไม่เคยแท้ง
มีเหงือกอัก	มีหินน้ำลาย	ปวส.	มีงานทำ	อยู่ด้วยกันก	ครอบครัวเดี	สิทธิหลักป	มีลูกแล้วอย	ไม่เคยแท้ง
มีเหงือกอัก	มีหินน้ำลาย	ม.ต้น	ว่างงาน	อยู่ด้วยกันก	ครอบครัวข	สิทธิหลักป	ไม่มีลูก	ไม่เคยแท้ง
มีเหงือกอัก	มีหินน้ำลาย	ปวส.	มีงานทำ	อยู่ด้วยกันก	ครอบครัวเดี	สิทธิประกัน	ไม่มีลูก	ไม่เคยแท้ง
มีเหงือกอัก	มีหินน้ำลาย	ม.ปลาย/ปว	มีงานทำ	อยู่ด้วยกันก	ครอบครัวข	สิทธิหลักป	มีลูกแล้วอย	ไม่เคยแท้ง
มีเหงือกอัก	มีหินน้ำลาย	ม.ปลาย/ปว	มีงานทำ	อยู่ด้วยกันก	ครอบครัวข	สิทธิหลักป	ไม่มีลูก	ไม่เคยแท้ง
มีเหงือกอัก	มีหินน้ำลาย	ม.ปลาย/ปว	มีงานทำ	อยู่ด้วยกันก	ครอบครัวข	สิทธิหลักป	มีลูกแล้วอย	ไม่เคยแท้ง
ไม่มีเหงือก	ไม่มีหินน้ำล	ม.ต้น	ว่างงาน	อยู่ด้วยกันก	ครอบครัวข	สิทธิหลักป	ไม่มีลูก	ไม่เคยแท้ง
มีเหงือกอัก	มีหินน้ำลาย	ม.ปลาย/ปว	ว่างงาน	อยู่ด้วยกันก	ครอบครัวข	สิทธิหลักป	ไม่มีลูก	ไม่เคยแท้ง
มีเหงือกอัก	มีหินน้ำลาย	ปวส.	มีงานทำ	อยู่ด้วยกันก	ครอบครัวเดี	สิทธิประกัน	มีลูกแล้วอย	ไม่เคยแท้ง
มีเหงือกอัก	มีหินน้ำลาย	ปวส.	มีงานทำ	อยู่ด้วยกันก	ครอบครัวข	สิทธิประกัน	ไม่มีลูก	ไม่เคยแท้ง
ไม่มีเหงือก	ไม่มีหินน้ำล	ปวส.	ว่างงาน	อยู่ด้วยกันก	ครอบครัวเดี	สิทธิหลักป	ไม่มีลูก	ไม่เคยแท้ง
มีเหงือกอัก	มีหินน้ำลาย	ม.ปลาย/ปว	ว่างงาน	อยู่ด้วยกันก	ครอบครัวเดี	สิทธิหลักป	ไม่มีลูก	ไม่เคยแท้ง
มีเหงือกอัก	มีหินน้ำลาย	ป.ตรีขึ้นไป	มีงานทำ	อยู่ด้วยกันก	ครอบครัวเดี	สิทธิข้าราช	มีลูกแล้วอย	ไม่เคยแท้ง
มีเหงือกอัก	มีหินน้ำลาย	ป.ตรีขึ้นไป	มีงานทำ	อยู่ด้วยกันก	ครอบครัวเดี	สิทธิข้าราช	มีลูกแล้วอย	ไม่เคยแท้ง
มีเหงือกอัก	มีหินน้ำลาย	ม.ปลาย/ปว	มีงานทำ	อยู่ด้วยกันก	ครอบครัวเดี	สิทธิหลักป	ไม่มีลูก	ไม่เคยแท้ง
มีเหงือกอัก	มีหินน้ำลาย	ม.ต้น	มีงานทำ	อยู่ด้วยกันก	ครอบครัวเดี	สิทธิหลักป	มีลูกแล้วอย	ไม่เคยแท้ง
มีเหงือกอัก	มีหินน้ำลาย	ปวส.	มีงานทำ	อยู่ด้วยกันก	ครอบครัวเดี	สิทธิประกัน	ไม่มีลูก	ไม่เคยแท้ง
มีเหงือกอัก	มีหินน้ำลาย	ม.ต้น	ว่างงาน	อยู่ด้วยกันก	ครอบครัวข	สิทธิหลักป	ไม่มีลูก	ไม่เคยแท้ง
มีเหงือกอัก	มีหินน้ำลาย	ม.ต้น	มีงานทำ	อยู่ด้วยกันก	ครอบครัวข	สิทธิหลักป	มีลูกแล้วอย	ไม่เคยแท้ง
มีเหงือกอัก	มีหินน้ำลาย	ม.ต้น	มีงานทำ	อยู่ด้วยกันก	ครอบครัวเดี	สิทธิหลักป	ไม่มีลูก	ไม่เคยแท้ง
มีเหงือกอัก	มีหินน้ำลาย	ประถมฯ	ว่างงาน	อยู่ด้วยกันก	ครอบครัวเดี	สิทธิหลักป	มีลูกแล้วอย	ไม่เคยแท้ง

มีเหงือกอัก	มีหินน้ำลาย	ม.ปลาย/ปว	ว่างงาน	อยู่ด้วยกันก	ครอบครัวข	สิทธิหลักป	ไม่มีลูก	ไม่เคยแท้ง
มีเหงือกอัก	มีหินน้ำลาย	ม.ต้น	มีงานทำ	อยู่ด้วยกันก	ครอบครัวเ	สิทธิหลักป	ไม่มีลูก	ไม่เคยแท้ง
มีเหงือกอัก	มีหินน้ำลาย	ม.ปลาย/ปว	มีงานทำ	อยู่ด้วยกันก	ครอบครัวเ	สิทธิประกัน	ไม่มีลูก	ไม่เคยแท้ง
มีเหงือกอัก	มีหินน้ำลาย	ม.ต้น	มีงานทำ	อยู่ด้วยกันก	ครอบครัวข	สิทธิหลักป	มีลูกแล้วอย	ไม่เคยแท้ง
มีเหงือกอัก	มีหินน้ำลาย	ม.ต้น	มีงานทำ	อยู่ด้วยกันก	ครอบครัวเ	สิทธิหลักป	ไม่มีลูก	ไม่เคยแท้ง
มีเหงือกอัก	มีหินน้ำลาย	ม.ปลาย/ปว	ว่างงาน	อยู่ด้วยกันก	ครอบครัวเ	สิทธิหลักป	มีลูกแล้วอย	ไม่เคยแท้ง
ไม่มีเหงือก	ไม่มีหินน้ำล	ม.ปลาย/ปว	ว่างงาน	อยู่ด้วยกันก	ครอบครัวเ	สิทธิหลักป	ไม่มีลูก	ไม่เคยแท้ง
ไม่มีเหงือก	ไม่มีหินน้ำล	ปวส.	ว่างงาน	อยู่ด้วยกันก	ครอบครัวเ	สิทธิหลักป	มีลูกแล้วอย	ไม่เคยแท้ง
มีเหงือกอัก	มีหินน้ำลาย	ม.ปลาย/ปว	มีงานทำ	อยู่ด้วยกันก	ครอบครัวเ	สิทธิหลักป	มีลูกแล้วอย	ไม่เคยแท้ง
มีเหงือกอัก	มีหินน้ำลาย	ป.ตรีขึ้นไป	มีงานทำ	อยู่ด้วยกันก	ครอบครัวเ	สิทธิประกัน	มีลูกแล้วอย	ไม่เคยแท้ง
ไม่มีเหงือก	ไม่มีหินน้ำล	ปวส.	ว่างงาน	อยู่ด้วยกันก	ครอบครัวเ	สิทธิหลักป	มีลูกแล้วอย	ไม่เคยแท้ง
มีเหงือกอัก	มีหินน้ำลาย	ปวส.	มีงานทำ	อยู่ด้วยกันก	ครอบครัวเ	สิทธิหลักป	มีลูกแล้วอย	ไม่เคยแท้ง
มีเหงือกอัก	มีหินน้ำลาย	ม.ปลาย/ปว	ว่างงาน	อยู่ด้วยกันก	ครอบครัวเ	สิทธิหลักป	ไม่มีลูก	ไม่เคยแท้ง
มีเหงือกอัก	มีหินน้ำลาย	ม.ปลาย/ปว	มีงานทำ	อยู่ด้วยกันก	ครอบครัวเ	สิทธิประกัน	ไม่มีลูก	ไม่เคยแท้ง
มีเหงือกอัก	มีหินน้ำลาย	ปวส.	มีงานทำ	ไม่ได้อยู่กับ	ครอบครัวเ	สิทธิหลักป	มีลูกแล้วอย	ไม่เคยแท้ง
มีเหงือกอัก	มีหินน้ำลาย	ม.ปลาย/ปว	มีงานทำ	อยู่ด้วยกันก	ครอบครัวเ	สิทธิหลักป	มีลูกแล้วอย	ไม่เคยแท้ง
มีเหงือกอัก	มีหินน้ำลาย	ป.ตรีขึ้นไป	มีงานทำ	อยู่ด้วยกันก	ครอบครัวข	สิทธิข้าราช	มีลูกแล้วอย	ไม่เคยแท้ง
มีเหงือกอัก	มีหินน้ำลาย	ประถมฯ	ว่างงาน	อยู่ด้วยกันก	ครอบครัวเ	สิทธิหลักป	มีลูกแล้วอย	เคยแท้ง
มีเหงือกอัก	มีหินน้ำลาย	ม.ต้น	มีงานทำ	อยู่ด้วยกันก	ครอบครัวเ	สิทธิหลักป	มีลูกแล้วอย	ไม่เคยแท้ง
ไม่มีเหงือก	ไม่มีหินน้ำล	ปวส.	ว่างงาน	อยู่ด้วยกันก	ครอบครัวเ	สิทธิหลักป	ไม่มีลูก	ไม่เคยแท้ง
มีเหงือกอัก	มีหินน้ำลาย	ม.ปลาย/ปว	ว่างงาน	ไม่ได้อยู่กับ	ครอบครัวเ	สิทธิหลักป	ไม่มีลูก	ไม่เคยแท้ง
มีเหงือกอัก	มีหินน้ำลาย	ม.ปลาย/ปว	มีงานทำ	อยู่ด้วยกันก	ครอบครัวเ	สิทธิหลักป	ไม่มีลูก	ไม่เคยแท้ง
มีเหงือกอัก	มีหินน้ำลาย	ประถมฯ	มีงานทำ	อยู่ด้วยกันก	ครอบครัวเ	สิทธิหลักป	มีลูกแล้วอย	ไม่เคยแท้ง
มีเหงือกอัก	มีหินน้ำลาย	ประถมฯ	มีงานทำ	อยู่ด้วยกันก	ครอบครัวเ	สิทธิหลักป	มีลูกแล้วอย	ไม่เคยแท้ง
มีเหงือกอัก	มีหินน้ำลาย	ม.ปลาย/ปว	มีงานทำ	อยู่ด้วยกันก	ครอบครัวเ	สิทธิหลักป	มีลูกแล้วอย	ไม่เคยแท้ง
มีเหงือกอัก	มีหินน้ำลาย	ป.ตรีขึ้นไป	มีงานทำ	อยู่ด้วยกันก	ครอบครัวเ	สิทธิหลักป	มีลูกแล้วอย	ไม่เคยแท้ง
มีเหงือกอัก	มีหินน้ำลาย	ปวส.	มีงานทำ	อยู่ด้วยกันก	ครอบครัวข	สิทธิหลักป	ไม่มีลูก	ไม่เคยแท้ง
มีเหงือกอัก	มีหินน้ำลาย	ม.ปลาย/ปว	มีงานทำ	อยู่ด้วยกันก	ครอบครัวเ	สิทธิหลักป	ไม่มีลูก	ไม่เคยแท้ง
ไม่มีเหงือก	ไม่มีหินน้ำล	ม.ปลาย/ปว	มีงานทำ	อยู่ด้วยกันก	ครอบครัวข	สิทธิหลักป	มีลูกแล้วอย	ไม่เคยแท้ง
มีเหงือกอัก	มีหินน้ำลาย	ม.ต้น	มีงานทำ	อยู่ด้วยกันก	ครอบครัวเ	อื่นๆ	มีลูกแล้วอย	ไม่เคยแท้ง
มีเหงือกอัก	มีหินน้ำลาย	ม.ต้น	มีงานทำ	อยู่ด้วยกันก	ครอบครัวเ	สิทธิหลักป	ไม่มีลูก	ไม่เคยแท้ง
มีเหงือกอัก	มีหินน้ำลาย	ม.ปลาย/ปว	มีงานทำ	อยู่ด้วยกันก	ครอบครัวเ	สิทธิหลักป	มีลูกแล้วอย	เคยแท้ง
ไม่มีเหงือก	ไม่มีหินน้ำล	ป.ตรีขึ้นไป	ว่างงาน	อยู่ด้วยกันก	ครอบครัวข	สิทธิหลักป	ไม่มีลูก	ไม่เคยแท้ง
มีเหงือกอัก	มีหินน้ำลาย	ม.ปลาย/ปว	มีงานทำ	อยู่ด้วยกันก	ครอบครัวเ	สิทธิหลักป	ไม่มีลูก	ไม่เคยแท้ง
มีเหงือกอัก	มีหินน้ำลาย	ม.ต้น	มีงานทำ	อยู่ด้วยกันก	ครอบครัวเ	สิทธิหลักป	ไม่มีลูก	ไม่เคยแท้ง
ไม่มีเหงือก	ไม่มีหินน้ำล	ป.ตรีขึ้นไป	มีงานทำ	อยู่ด้วยกันก	ครอบครัวข	สิทธิหลักป	มีลูกแล้วอย	ไม่เคยแท้ง
มีเหงือกอัก	มีหินน้ำลาย	ปวส.	มีงานทำ	อยู่ด้วยกันก	ครอบครัวข	สิทธิหลักป	มีลูกแล้วอย	ไม่เคยแท้ง
มีเหงือกอัก	มีหินน้ำลาย	ปวส.	ว่างงาน	ไม่ได้อยู่กับ	ครอบครัวเ	สิทธิหลักป	ไม่มีลูก	ไม่เคยแท้ง
มีเหงือกอัก	มีหินน้ำลาย	ม.ต้น	มีงานทำ	อยู่ด้วยกันก	ครอบครัวเ	สิทธิหลักป	ไม่มีลูก	ไม่เคยแท้ง
มีเหงือกอัก	มีหินน้ำลาย	ม.ต้น	มีงานทำ	อยู่ด้วยกันก	ครอบครัวข	สิทธิหลักป	มีลูกแล้วอย	ไม่เคยแท้ง
มีเหงือกอัก	มีหินน้ำลาย	ม.ต้น	มีงานทำ	อยู่ด้วยกันก	ครอบครัวเ	สิทธิหลักป	ไม่มีลูก	ไม่เคยแท้ง
มีเหงือกอัก	มีหินน้ำลาย	ประถมฯ	ว่างงาน	อยู่ด้วยกันก	ครอบครัวเ	สิทธิหลักป	มีลูกแล้วอย	ไม่เคยแท้ง
มีเหงือกอัก	มีหินน้ำลาย	ม.ปลาย/ปว	ว่างงาน	อยู่ด้วยกันก	ครอบครัวข	สิทธิหลักป	ไม่มีลูก	ไม่เคยแท้ง
มีเหงือกอัก	มีหินน้ำลาย	ม.ต้น	มีงานทำ	อยู่ด้วยกันก	ครอบครัวเ	สิทธิหลักป	ไม่มีลูก	ไม่เคยแท้ง
มีเหงือกอัก	มีหินน้ำลาย	ม.ปลาย/ปว	มีงานทำ	อยู่ด้วยกันก	ครอบครัวเ	สิทธิประกัน	ไม่มีลูก	ไม่เคยแท้ง
มีเหงือกอัก	มีหินน้ำลาย	ม.ต้น	มีงานทำ	อยู่ด้วยกันก	ครอบครัวข	สิทธิหลักป	มีลูกแล้วอย	ไม่เคยแท้ง
มีเหงือกอัก	มีหินน้ำลาย	ป.ตรีขึ้นไป	มีงานทำ	ไม่ได้อยู่กับ	ครอบครัวเ	สิทธิข้าราช	ไม่มีลูก	ไม่เคยแท้ง
มีเหงือกอัก	มีหินน้ำลาย	ม.ปลาย/ปว	มีงานทำ	อยู่ด้วยกันก	ครอบครัวเ	สิทธิข้าราช	ไม่มีลูก	ไม่เคยแท้ง
มีเหงือกอัก	มีหินน้ำลาย	ปวส.	มีงานทำ	อยู่ด้วยกันก	ครอบครัวข	สิทธิหลักป	ไม่มีลูก	ไม่เคยแท้ง

Parity_G	Gravida_G	Alc_G	Smoke_G	Region_G	Suscept_G	Severe_G	Barrier_G	HeLD_G
ไม่เคยคลอ	ตั้งครรภ์ครั้ง	ไม่ดื่ม	ไม่สูบ	พุทธ	ระดับปานก	ระดับสูง	ระดับปานก	ระดับสูง
ไม่เคยคลอ	ตั้งครรภ์ครั้ง	ไม่ดื่ม	ไม่สูบ	พุทธ	ระดับสูง	ระดับสูง	ระดับต่ำ	ระดับสูง
เคยคลอดม	ตั้งครรภ์มาก	ไม่ดื่ม	ไม่สูบ	พุทธ	ระดับสูง	ระดับสูง	ระดับสูง	ระดับสูง
เคยคลอด 1	ตั้งครรภ์มาก	ไม่ดื่ม	ไม่สูบ	พุทธ	ระดับต่ำ	ระดับปานก	ระดับปานก	ระดับสูง
เคยคลอด 1	ตั้งครรภ์มาก	ไม่ดื่ม	ไม่สูบ	พุทธ	ระดับปานก	ระดับปานก	ระดับปานก	ระดับปานก
ไม่เคยคลอ	ตั้งครรภ์ครั้ง	ไม่ดื่ม	ไม่สูบ	พุทธ	ระดับสูง	ระดับสูง	ระดับต่ำ	ระดับปานก
เคยคลอด 1	ตั้งครรภ์มาก	ไม่ดื่ม	ไม่สูบ	พุทธ	ระดับปานก	ระดับปานก	ระดับปานก	ระดับต่ำ
เคยคลอด 1	ตั้งครรภ์มาก	ไม่ดื่ม	ไม่สูบ	พุทธ	ระดับสูง	ระดับสูง	ระดับต่ำ	ระดับสูง
ไม่เคยคลอ	ตั้งครรภ์ครั้ง	ไม่ดื่ม	ไม่สูบ	พุทธ	ระดับสูง	ระดับสูง	ระดับปานก	ระดับปานก
ไม่เคยคลอ	ตั้งครรภ์ครั้ง	ไม่ดื่ม	ไม่สูบ	พุทธ	ระดับปานก	ระดับปานก	ระดับปานก	ระดับสูง
เคยคลอดม	ตั้งครรภ์มาก	ไม่ดื่ม	ไม่สูบ	พุทธ	ระดับสูง	ระดับสูง	ระดับต่ำ	ระดับปานก
เคยคลอด 1	ตั้งครรภ์มาก	ไม่ดื่ม	ไม่สูบ	พุทธ	ระดับปานก	ระดับปานก	ระดับปานก	ระดับสูง
ไม่เคยคลอ	ตั้งครรภ์ครั้ง	ไม่ดื่ม	ไม่สูบ	พุทธ	ระดับปานก	ระดับสูง	ระดับปานก	ระดับปานก
ไม่เคยคลอ	ตั้งครรภ์ครั้ง	ไม่ดื่ม	ไม่สูบ	พุทธ	ระดับสูง	ระดับปานก	ระดับต่ำ	ระดับสูง
ไม่เคยคลอ	ตั้งครรภ์ครั้ง	ไม่ดื่ม	ไม่สูบ	พุทธ	ระดับสูง	ระดับสูง	ระดับปานก	ระดับปานก
ไม่เคยคลอ	ตั้งครรภ์ครั้ง	ไม่ดื่ม	ไม่สูบ	พุทธ	ระดับสูง	ระดับปานก	ระดับปานก	ระดับปานก
ไม่เคยคลอ	ตั้งครรภ์ครั้ง	ไม่ดื่ม	ไม่สูบ	พุทธ	ระดับปานก	ระดับปานก	ระดับปานก	ระดับปานก
เคยคลอด 1	ตั้งครรภ์มาก	ไม่ดื่ม	ไม่สูบ	พุทธ	ระดับปานก	ระดับปานก	ระดับปานก	ระดับสูง
ไม่เคยคลอ	ตั้งครรภ์ครั้ง	ไม่ดื่ม	ไม่สูบ	พุทธ	ระดับปานก	ระดับปานก	ระดับปานก	ระดับสูง
ไม่เคยคลอ	ตั้งครรภ์ครั้ง	ไม่ดื่ม	ไม่สูบ	พุทธ	ระดับปานก	ระดับปานก	ระดับปานก	ระดับสูง
ไม่เคยคลอ	ตั้งครรภ์ครั้ง	ไม่ดื่ม	ไม่สูบ	พุทธ	ระดับปานก	ระดับปานก	ระดับปานก	ระดับสูง
เคยคลอด 1	ตั้งครรภ์มาก	ไม่ดื่ม	ไม่สูบ	พุทธ	ระดับปานก	ระดับสูง	ระดับปานก	ระดับต่ำ
เคยคลอด 1	ตั้งครรภ์มาก	ไม่ดื่ม	ไม่สูบ	พุทธ	ระดับปานก	ระดับสูง	ระดับปานก	ระดับสูง
ไม่เคยคลอ	ตั้งครรภ์ครั้ง	ไม่ดื่ม	ไม่สูบ	พุทธ	ระดับปานก	ระดับปานก	ระดับปานก	ระดับปานก
เคยคลอด 1	ตั้งครรภ์มาก	ไม่ดื่ม	ไม่สูบ	พุทธ	ระดับปานก	ระดับปานก	ระดับต่ำ	ระดับสูง
เคยคลอด 1	ตั้งครรภ์มาก	ไม่ดื่ม	ไม่สูบ	พุทธ	ระดับปานก	ระดับปานก	ระดับปานก	ระดับสูง
ไม่เคยคลอ	ตั้งครรภ์ครั้ง	ไม่ดื่ม	ไม่สูบ	พุทธ	ระดับสูง	ระดับสูง	ระดับปานก	ระดับสูง
ไม่เคยคลอ	ตั้งครรภ์ครั้ง	ไม่ดื่ม	ไม่สูบ	พุทธ	ระดับปานก	ระดับปานก	ระดับสูง	ระดับสูง
ไม่เคยคลอ	ตั้งครรภ์ครั้ง	ไม่ดื่ม	ไม่สูบ	พุทธ	ระดับปานก	ระดับสูง	ระดับปานก	ระดับสูง
เคยคลอด 1	ตั้งครรภ์มาก	ไม่ดื่ม	ไม่สูบ	พุทธ	ระดับปานก	ระดับปานก	ระดับปานก	ระดับสูง
ไม่เคยคลอ	ตั้งครรภ์ครั้ง	ไม่ดื่ม	ไม่สูบ	พุทธ	ระดับปานก	ระดับปานก	ระดับปานก	ระดับปานก
ไม่เคยคลอ	ตั้งครรภ์ครั้ง	ไม่ดื่ม	ไม่สูบ	พุทธ	ระดับปานก	ระดับปานก	ระดับปานก	ระดับสูง
เคยคลอด 1	ตั้งครรภ์มาก	ไม่ดื่ม	ไม่สูบ	พุทธ	ระดับสูง	ระดับสูง	ระดับต่ำ	ระดับสูง
ไม่เคยคลอ	ตั้งครรภ์มาก	ไม่ดื่ม	ไม่สูบ	พุทธ	ระดับสูง	ระดับปานก	ระดับต่ำ	ระดับสูง
ไม่เคยคลอ	ตั้งครรภ์ครั้ง	ไม่ดื่ม	ไม่สูบ	พุทธ	ระดับสูง	ระดับปานก	ระดับต่ำ	ระดับสูง
เคยคลอด 1	ตั้งครรภ์มาก	ไม่ดื่ม	ไม่สูบ	พุทธ	ระดับปานก	ระดับปานก	ระดับปานก	ระดับสูง
ไม่เคยคลอ	ตั้งครรภ์ครั้ง	ไม่ดื่ม	ไม่สูบ	พุทธ	ระดับปานก	ระดับสูง	ระดับปานก	ระดับสูง
ไม่เคยคลอ	ตั้งครรภ์ครั้ง	ไม่ดื่ม	ไม่สูบ	พุทธ	ระดับปานก	ระดับปานก	ระดับปานก	ระดับปานก
เคยคลอด 1	ตั้งครรภ์มาก	ไม่ดื่ม	ไม่สูบ	พุทธ	ระดับปานก	ระดับปานก	ระดับปานก	ระดับสูง
ไม่เคยคลอ	ตั้งครรภ์ครั้ง	ไม่ดื่ม	ไม่สูบ	พุทธ	ระดับสูง	ระดับปานก	ระดับปานก	ระดับต่ำ
เคยคลอด 1	ตั้งครรภ์มาก	ไม่ดื่ม	ไม่สูบ	อื่นๆ	ระดับสูง	ระดับสูง	ระดับปานก	ระดับปานก
เคยคลอดม	ตั้งครรภ์มาก	ไม่ดื่ม	ไม่สูบ	พุทธ	ระดับปานก	ระดับปานก	ระดับปานก	ระดับปานก
ไม่เคยคลอ	ตั้งครรภ์ครั้ง	ดื่ม	ไม่สูบ	พุทธ	ระดับปานก	ระดับปานก	ระดับต่ำ	ระดับต่ำ
ไม่เคยคลอ	ตั้งครรภ์ครั้ง	ไม่ดื่ม	ไม่สูบ	พุทธ	ระดับปานก	ระดับปานก	ระดับต่ำ	ระดับปานก
ไม่เคยคลอ	ตั้งครรภ์ครั้ง	ไม่ดื่ม	ไม่สูบ	พุทธ	ระดับปานก	ระดับปานก	ระดับต่ำ	ระดับสูง
ไม่เคยคลอ	ตั้งครรภ์ครั้ง	ไม่ดื่ม	ไม่สูบ	อื่นๆ	ระดับปานก	ระดับปานก	ระดับปานก	ระดับปานก
ไม่เคยคลอ	ตั้งครรภ์ครั้ง	ไม่ดื่ม	ไม่สูบ	พุทธ	ระดับปานก	ระดับปานก	ระดับปานก	ระดับสูง
เคยคลอด 1	ตั้งครรภ์มาก	ไม่ดื่ม	ไม่สูบ	พุทธ	ระดับสูง	ระดับสูง	ระดับปานก	ระดับสูง
เคยคลอด 1	ตั้งครรภ์มาก	ไม่ดื่ม	ไม่สูบ	พุทธ	ระดับสูง	ระดับสูง	ระดับสูง	ระดับปานก
เคยคลอด 1	ตั้งครรภ์มาก	ไม่ดื่ม	ไม่สูบ	พุทธ	ระดับปานก	ระดับปานก	ระดับปานก	ระดับปานก

ไม่เคยคลอ	ตั้งครรภ์ครั้ง	ไม่ดื่ม	ไม่สูบ	พุทธ	ระดับปานก	ระดับสูง	ระดับปานก	ระดับสูง
เคยคลอดม	ตั้งครรภ์มาก	ไม่ดื่ม	ไม่สูบ	พุทธ	ระดับสูง	ระดับสูง	ระดับปานก	ระดับปานก
ไม่เคยคลอ	ตั้งครรภ์ครั้ง	ไม่ดื่ม	ไม่สูบ	พุทธ	ระดับปานก	ระดับสูง	ระดับปานก	ระดับสูง
ไม่เคยคลอ	ตั้งครรภ์ครั้ง	ไม่ดื่ม	ไม่สูบ	พุทธ	ระดับปานก	ระดับสูง	ระดับปานก	ระดับสูง
ไม่เคยคลอ	ตั้งครรภ์ครั้ง	ดื่ม	ไม่สูบ	พุทธ	ระดับปานก	ระดับสูง	ระดับสูง	ระดับสูง
ไม่เคยคลอ	ตั้งครรภ์ครั้ง	ไม่ดื่ม	ไม่สูบ	พุทธ	ระดับสูง	ระดับสูง	ระดับปานก	ระดับปานก
เคยคลอด 1	ตั้งครรภ์มาก	ไม่ดื่ม	ไม่สูบ	พุทธ	ระดับปานก	ระดับสูง	ระดับปานก	ระดับสูง
ไม่เคยคลอ	ตั้งครรภ์ครั้ง	ไม่ดื่ม	ไม่สูบ	พุทธ	ระดับปานก	ระดับสูง	ระดับปานก	ระดับต่ำ
ไม่เคยคลอ	ตั้งครรภ์ครั้ง	ไม่ดื่ม	ไม่สูบ	พุทธ	ระดับสูง	ระดับสูง	ระดับสูง	ระดับปานก
เคยคลอด 1	ตั้งครรภ์มาก	ไม่ดื่ม	ไม่สูบ	พุทธ	ระดับสูง	ระดับสูง	ระดับปานก	ระดับสูง
เคยคลอดม	ตั้งครรภ์มาก	ไม่ดื่ม	ไม่สูบ	พุทธ	ระดับสูง	ระดับสูง	ระดับปานก	ระดับสูง
เคยคลอด 1	ตั้งครรภ์มาก	ไม่ดื่ม	ไม่สูบ	พุทธ	ระดับปานก	ระดับสูง	ระดับสูง	ระดับปานก
เคยคลอด 1	ตั้งครรภ์มาก	ไม่ดื่ม	ไม่สูบ	อื่นๆ	ระดับปานก	ระดับปานก	ระดับปานก	ระดับสูง
เคยคลอด 1	ตั้งครรภ์มาก	ไม่ดื่ม	ไม่สูบ	พุทธ	ระดับปานก	ระดับสูง	ระดับปานก	ระดับสูง
ไม่เคยคลอ	ตั้งครรภ์ครั้ง	ไม่ดื่ม	ไม่สูบ	พุทธ	ระดับปานก	ระดับสูง	ระดับปานก	ระดับสูง
ไม่เคยคลอ	ตั้งครรภ์ครั้ง	ไม่ดื่ม	ไม่สูบ	พุทธ	ระดับปานก	ระดับสูง	ระดับปานก	ระดับสูง
ไม่เคยคลอ	ตั้งครรภ์มาก	ไม่ดื่ม	ไม่สูบ	พุทธ	ระดับสูง	ระดับปานก	ระดับปานก	ระดับปานก
เคยคลอด 1	ตั้งครรภ์มาก	ไม่ดื่ม	ไม่สูบ	พุทธ	ระดับสูง	ระดับปานก	ระดับปานก	ระดับปานก
ไม่เคยคลอ	ตั้งครรภ์ครั้ง	ไม่ดื่ม	ไม่สูบ	พุทธ	ระดับปานก	ระดับสูง	ระดับปานก	ระดับปานก
ไม่เคยคลอ	ตั้งครรภ์ครั้ง	ไม่ดื่ม	ไม่สูบ	พุทธ	ระดับปานก	ระดับสูง	ระดับต่ำ	ระดับปานก
ไม่เคยคลอ	ตั้งครรภ์ครั้ง	ไม่ดื่ม	ไม่สูบ	พุทธ	ระดับสูง	ระดับสูง	ระดับสูง	ระดับสูง
เคยคลอดม	ตั้งครรภ์มาก	ไม่ดื่ม	ไม่สูบ	พุทธ	ระดับปานก	ระดับปานก	ระดับปานก	ระดับปานก
เคยคลอด 1	ตั้งครรภ์มาก	ไม่ดื่ม	ไม่สูบ	พุทธ	ระดับปานก	ระดับปานก	ระดับปานก	ระดับสูง
เคยคลอด 1	ตั้งครรภ์มาก	ไม่ดื่ม	ไม่สูบ	พุทธ	ระดับปานก	ระดับปานก	ระดับปานก	ระดับปานก
ไม่เคยคลอ	ตั้งครรภ์ครั้ง	ไม่ดื่ม	ไม่สูบ	พุทธ	ระดับปานก	ระดับปานก	ระดับปานก	ระดับสูง
เคยคลอดม	ตั้งครรภ์มาก	ไม่ดื่ม	ไม่สูบ	พุทธ	ระดับปานก	ระดับปานก	ระดับปานก	ระดับต่ำ
ไม่เคยคลอ	ตั้งครรภ์ครั้ง	ไม่ดื่ม	ไม่สูบ	พุทธ	ระดับปานก	ระดับปานก	ระดับปานก	ระดับสูง
ไม่เคยคลอ	ตั้งครรภ์ครั้ง	ไม่ดื่ม	ไม่สูบ	พุทธ	ระดับปานก	ระดับปานก	ระดับปานก	ระดับสูง
ไม่เคยคลอ	ตั้งครรภ์ครั้ง	ไม่ดื่ม	ไม่สูบ	พุทธ	ระดับปานก	ระดับปานก	ระดับปานก	ระดับสูง
เคยคลอด 1	ตั้งครรภ์มาก	ไม่ดื่ม	ไม่สูบ	พุทธ	ระดับสูง	ระดับปานก	ระดับปานก	ระดับสูง
เคยคลอดม	ตั้งครรภ์มาก	ไม่ดื่ม	ไม่สูบ	พุทธ	ระดับปานก	ระดับสูง	ระดับต่ำ	ระดับสูง
เคยคลอด 1	ตั้งครรภ์มาก	ไม่ดื่ม	ไม่สูบ	พุทธ	ระดับสูง	ระดับสูง	ระดับต่ำ	ระดับสูง
ไม่เคยคลอ	ตั้งครรภ์ครั้ง	ไม่ดื่ม	ไม่สูบ	พุทธ	ระดับสูง	ระดับสูง	ระดับสูง	ระดับสูง
ไม่เคยคลอ	ตั้งครรภ์ครั้ง	ไม่ดื่ม	ไม่สูบ	พุทธ	ระดับปานก	ระดับปานก	ระดับปานก	ระดับสูง
เคยคลอดม	ตั้งครรภ์มาก	ไม่ดื่ม	ไม่สูบ	พุทธ	ระดับสูง	ระดับปานก	ระดับต่ำ	ระดับสูง
เคยคลอด 1	ตั้งครรภ์มาก	ไม่ดื่ม	ไม่สูบ	พุทธ	ระดับสูง	ระดับสูง	ระดับปานก	ระดับสูง
ไม่เคยคลอ	ตั้งครรภ์ครั้ง	ไม่ดื่ม	ไม่สูบ	พุทธ	ระดับสูง	ระดับสูง	ระดับต่ำ	ระดับสูง
ไม่เคยคลอ	ตั้งครรภ์ครั้ง	ไม่ดื่ม	ไม่สูบ	พุทธ	ระดับสูง	ระดับสูง	ระดับปานก	ระดับปานก
ไม่เคยคลอ	ตั้งครรภ์ครั้ง	ไม่ดื่ม	ไม่สูบ	พุทธ	ระดับปานก	ระดับสูง	ระดับปานก	ระดับสูง
เคยคลอด 1	ตั้งครรภ์มาก	ไม่ดื่ม	ไม่สูบ	พุทธ	ระดับสูง	ระดับสูง	ระดับสูง	ระดับปานก
เคยคลอดม	ตั้งครรภ์มาก	ไม่ดื่ม	ไม่สูบ	พุทธ	ระดับสูง	ระดับสูง	ระดับปานก	ระดับสูง
เคยคลอด 1	ตั้งครรภ์มาก	ไม่ดื่ม	ไม่สูบ	พุทธ	ระดับปานก	ระดับปานก	ระดับปานก	ระดับปานก
ไม่เคยคลอ	ตั้งครรภ์ครั้ง	ไม่ดื่ม	ไม่สูบ	พุทธ	ระดับปานก	ระดับปานก	ระดับปานก	ระดับสูง
เคยคลอดม	ตั้งครรภ์มาก	ไม่ดื่ม	ไม่สูบ	พุทธ	ระดับปานก	ระดับปานก	ระดับปานก	ระดับปานก
เคยคลอดม	ตั้งครรภ์มาก	ไม่ดื่ม	ไม่สูบ	พุทธ	ระดับปานก	ระดับปานก	ระดับปานก	ระดับปานก
ไม่เคยคลอ	ตั้งครรภ์ครั้ง	ไม่ดื่ม	ไม่สูบ	พุทธ	ระดับปานก	ระดับปานก	ระดับปานก	ระดับปานก
เคยคลอด 1	ตั้งครรภ์มาก	ไม่ดื่ม	ไม่สูบ	พุทธ	ระดับปานก	ระดับสูง	ระดับปานก	ระดับสูง
ไม่เคยคลอ	ตั้งครรภ์ครั้ง	ไม่ดื่ม	ไม่สูบ	พุทธ	ระดับปานก	ระดับปานก	ระดับปานก	ระดับปานก
เคยคลอดม	ตั้งครรภ์มาก	ไม่ดื่ม	ไม่สูบ	พุทธ	ระดับปานก	ระดับปานก	ระดับปานก	ระดับปานก
ไม่เคยคลอ	ตั้งครรภ์ครั้ง	ไม่ดื่ม	ไม่สูบ	พุทธ	ระดับปานก	ระดับสูง	ระดับต่ำ	ระดับปานก
เคยคลอด 1	ตั้งครรภ์มาก	ไม่ดื่ม	ไม่สูบ	พุทธ	ระดับปานก	ระดับปานก	ระดับปานก	ระดับต่ำ

เคยคลอด 1	ตั้งครรภ์มาก	ไม่ดื่ม	ไม่สูบ	พุทธ	ระดับปานก	ระดับปานก	ระดับปานก	ระดับสูง
เคยคลอดม	ตั้งครรภ์มาก	ไม่ดื่ม	ไม่สูบ	พุทธ	ระดับสูง	ระดับสูง	ระดับสูง	ระดับสูง
ไม่เคยคลอ	ตั้งครรภ์มาก	ไม่ดื่ม	ไม่สูบ	พุทธ	ระดับปานก	ระดับสูง	ระดับปานก	ระดับปานก
เคยคลอดม	ตั้งครรภ์มาก	ไม่ดื่ม	ไม่สูบ	พุทธ	ระดับสูง	ระดับสูง	ระดับปานก	ระดับสูง
เคยคลอด 1	ตั้งครรภ์มาก	ไม่ดื่ม	ไม่สูบ	พุทธ	ระดับสูง	ระดับสูง	ระดับสูง	ระดับปานก
เคยคลอด 1	ตั้งครรภ์มาก	ไม่ดื่ม	ไม่สูบ	พุทธ	ระดับสูง	ระดับสูง	ระดับปานก	ระดับปานก
ไม่เคยคลอ	ตั้งครรภ์ครั้ง	ไม่ดื่ม	ไม่สูบ	พุทธ	ระดับสูง	ระดับสูง	ระดับสูง	ระดับปานก
ไม่เคยคลอ	ตั้งครรภ์ครั้ง	ไม่ดื่ม	ไม่สูบ	พุทธ	ระดับสูง	ระดับสูง	ระดับปานก	ระดับสูง
ไม่เคยคลอ	ตั้งครรภ์ครั้ง	ไม่ดื่ม	ไม่สูบ	พุทธ	ระดับปานก	ระดับปานก	ระดับต่ำ	ระดับสูง
ไม่เคยคลอ	ตั้งครรภ์ครั้ง	ไม่ดื่ม	ไม่สูบ	พุทธ	ระดับปานก	ระดับปานก	ระดับปานก	ระดับสูง
ไม่เคยคลอ	ตั้งครรภ์ครั้ง	ไม่ดื่ม	ไม่สูบ	พุทธ	ระดับปานก	ระดับปานก	ระดับปานก	ระดับปานก
ไม่เคยคลอ	ตั้งครรภ์ครั้ง	ไม่ดื่ม	ไม่สูบ	พุทธ	ระดับปานก	ระดับปานก	ระดับปานก	ระดับสูง
เคยคลอดม	ตั้งครรภ์มาก	ไม่ดื่ม	ไม่สูบ	พุทธ	ระดับสูง	ระดับปานก	ระดับปานก	ระดับสูง
เคยคลอดม	ตั้งครรภ์มาก	ไม่ดื่ม	ไม่สูบ	พุทธ	ระดับสูง	ระดับสูง	ระดับต่ำ	ระดับปานก
เคยคลอดม	ตั้งครรภ์มาก	ไม่ดื่ม	ไม่สูบ	พุทธ	ระดับปานก	ระดับปานก	ระดับปานก	ระดับสูง
เคยคลอดม	ตั้งครรภ์มาก	ไม่ดื่ม	ไม่สูบ	พุทธ	ระดับปานก	ระดับสูง	ระดับสูง	ระดับปานก
ไม่เคยคลอ	ตั้งครรภ์ครั้ง	ไม่ดื่ม	ไม่สูบ	พุทธ	ระดับสูง	ระดับปานก	ระดับปานก	ระดับสูง
เคยคลอด 1	ตั้งครรภ์มาก	ไม่ดื่ม	ไม่สูบ	พุทธ	ระดับสูง	ระดับสูง	ระดับสูง	ระดับปานก
เคยคลอดม	ตั้งครรภ์มาก	ไม่ดื่ม	ไม่สูบ	พุทธ	ระดับปานก	ระดับปานก	ระดับปานก	ระดับต่ำ
ไม่เคยคลอ	ตั้งครรภ์ครั้ง	ไม่ดื่ม	ไม่สูบ	พุทธ	ระดับปานก	ระดับปานก	ระดับปานก	ระดับสูง
ไม่เคยคลอ	ตั้งครรภ์ครั้ง	ไม่ดื่ม	ไม่สูบ	พุทธ	ระดับปานก	ระดับสูง	ระดับปานก	ระดับสูง
ไม่เคยคลอ	ตั้งครรภ์ครั้ง	ไม่ดื่ม	ไม่สูบ	พุทธ	ระดับสูง	ระดับสูง	ระดับปานก	ระดับสูง
ไม่เคยคลอ	ตั้งครรภ์ครั้ง	ไม่ดื่ม	ไม่สูบ	พุทธ	ระดับปานก	ระดับปานก	ระดับปานก	ระดับสูง
เคยคลอดม	ตั้งครรภ์มาก	ไม่ดื่ม	ไม่สูบ	พุทธ	ระดับสูง	ระดับสูง	ระดับปานก	ระดับปานก
ไม่เคยคลอ	ตั้งครรภ์ครั้ง	ไม่ดื่ม	ไม่สูบ	พุทธ	ระดับสูง	ระดับสูง	ระดับต่ำ	ระดับปานก
ไม่เคยคลอ	ตั้งครรภ์ครั้ง	ไม่ดื่ม	ไม่สูบ	พุทธ	ระดับต่ำ	ระดับสูง	ระดับปานก	ระดับสูง
ไม่เคยคลอ	ตั้งครรภ์มาก	ไม่ดื่ม	ไม่สูบ	พุทธ	ระดับปานก	ระดับปานก	ระดับต่ำ	ระดับสูง
เคยคลอด 1	ตั้งครรภ์มาก	ไม่ดื่ม	ไม่สูบ	พุทธ	ระดับปานก	ระดับปานก	ระดับปานก	ระดับปานก
เคยคลอด 1	ตั้งครรภ์ครั้ง	ไม่ดื่ม	ไม่สูบ	พุทธ	ระดับปานก	ระดับปานก	ระดับปานก	ระดับปานก
เคยคลอด 1	ตั้งครรภ์ครั้ง	ไม่ดื่ม	ไม่สูบ	พุทธ	ระดับสูง	ระดับสูง	ระดับปานก	ระดับสูง
เคยคลอด 1	ตั้งครรภ์มาก	ไม่ดื่ม	ไม่สูบ	พุทธ	ระดับสูง	ระดับสูง	ระดับต่ำ	ระดับสูง
ไม่เคยคลอ	ตั้งครรภ์ครั้ง	ไม่ดื่ม	ไม่สูบ	พุทธ	ระดับสูง	ระดับปานก	ระดับสูง	ระดับสูง
เคยคลอด 1	ตั้งครรภ์มาก	ไม่ดื่ม	ไม่สูบ	พุทธ	ระดับปานก	ระดับสูง	ระดับปานก	ระดับสูง
ไม่เคยคลอ	ตั้งครรภ์ครั้ง	ไม่ดื่ม	ไม่สูบ	พุทธ	ระดับปานก	ระดับปานก	ระดับต่ำ	ระดับสูง
ไม่เคยคลอ	ตั้งครรภ์ครั้ง	ไม่ดื่ม	ไม่สูบ	พุทธ	ระดับสูง	ระดับสูง	ระดับต่ำ	ระดับสูง
เคยคลอดม	ตั้งครรภ์มาก	ไม่ดื่ม	ไม่สูบ	พุทธ	ระดับปานก	ระดับปานก	ระดับปานก	ระดับสูง
ไม่เคยคลอ	ตั้งครรภ์ครั้ง	ไม่ดื่ม	ไม่สูบ	พุทธ	ระดับปานก	ระดับปานก	ระดับปานก	ระดับสูง
ไม่เคยคลอ	ตั้งครรภ์ครั้ง	ไม่ดื่ม	ไม่สูบ	พุทธ	ระดับสูง	ระดับสูง	ระดับสูง	ระดับต่ำ
ไม่เคยคลอ	ตั้งครรภ์ครั้ง	ไม่ดื่ม	ไม่สูบ	พุทธ	ระดับปานก	ระดับปานก	ระดับปานก	ระดับปานก
เคยคลอด 1	ตั้งครรภ์มาก	ไม่ดื่ม	ไม่สูบ	พุทธ	ระดับสูง	ระดับสูง	ระดับปานก	ระดับสูง
เคยคลอด 1	ตั้งครรภ์มาก	ไม่ดื่ม	ไม่สูบ	พุทธ	ระดับปานก	ระดับปานก	ระดับปานก	ระดับสูง
ไม่เคยคลอ	ตั้งครรภ์ครั้ง	ไม่ดื่ม	ไม่สูบ	พุทธ	ระดับสูง	ระดับสูง	ระดับต่ำ	ระดับปานก
เคยคลอด 1	ตั้งครรภ์มาก	ไม่ดื่ม	ไม่สูบ	พุทธ	ระดับปานก	ระดับปานก	ระดับปานก	ระดับปานก
เคยคลอดม	ตั้งครรภ์มาก	ไม่ดื่ม	ไม่สูบ	พุทธ	ระดับสูง	ระดับสูง	ระดับต่ำ	ระดับปานก
ไม่เคยคลอ	ตั้งครรภ์ครั้ง	ไม่ดื่ม	ไม่สูบ	พุทธ	ระดับปานก	ระดับสูง	ระดับปานก	ระดับสูง
ไม่เคยคลอ	ตั้งครรภ์ครั้ง	ไม่ดื่ม	ไม่สูบ	พุทธ	ระดับสูง	ระดับสูง	ระดับปานก	ระดับปานก
เคยคลอดม	ตั้งครรภ์มาก	ไม่ดื่ม	ไม่สูบ	พุทธ	ระดับปานก	ระดับปานก	ระดับปานก	ระดับสูง
ไม่เคยคลอ	ตั้งครรภ์ครั้ง	ไม่ดื่ม	ไม่สูบ	พุทธ	ระดับปานก	ระดับสูง	ระดับปานก	ระดับสูง
เคยคลอด 1	ตั้งครรภ์มาก	ไม่ดื่ม	ไม่สูบ	พุทธ	ระดับต่ำ	ระดับสูง	ระดับปานก	ระดับปานก
เคยคลอด 1	ตั้งครรภ์มาก	ไม่ดื่ม	ไม่สูบ	พุทธ	ระดับปานก	ระดับปานก	ระดับปานก	ระดับสูง
เคยคลอด 1	ตั้งครรภ์มาก	ไม่ดื่ม	ไม่สูบ	พุทธ	ระดับปานก	ระดับปานก	ระดับสูง	ระดับปานก

ไม่เคยคลอ	ตั้งครรภ์ครั้ง	ไม่ดื่ม	ไม่สูบ	พุทธ	ระดับปานก	ระดับปานก	ระดับปานก	ระดับปานก
เคยคลอด 1	ตั้งครรภ์มาก	ไม่ดื่ม	ไม่สูบ	พุทธ	ระดับปานก	ระดับสูง	ระดับปานก	ระดับสูง
ไม่เคยคลอ	ตั้งครรภ์ครั้ง	ไม่ดื่ม	ไม่สูบ	พุทธ	ระดับปานก	ระดับสูง	ระดับปานก	ระดับสูง
เคยคลอด 1	ตั้งครรภ์มาก	ไม่ดื่ม	ไม่สูบ	พุทธ	ระดับปานก	ระดับปานก	ระดับปานก	ระดับสูง
เคยคลอด 1	ตั้งครรภ์มาก	ไม่ดื่ม	ไม่สูบ	พุทธ	ระดับปานก	ระดับสูง	ระดับปานก	ระดับสูง
ไม่เคยคลอ	ตั้งครรภ์ครั้ง	ไม่ดื่ม	ไม่สูบ	พุทธ	ระดับปานก	ระดับสูง	ระดับต่ำ	ระดับต่ำ
เคยคลอด 1	ตั้งครรภ์มาก	ไม่ดื่ม	ไม่สูบ	พุทธ	ระดับปานก	ระดับปานก	ระดับปานก	ระดับสูง
เคยคลอดม	ตั้งครรภ์มาก	ไม่ดื่ม	ไม่สูบ	พุทธ	ระดับปานก	ระดับปานก	ระดับปานก	ระดับสูง
เคยคลอด 1	ตั้งครรภ์มาก	ไม่ดื่ม	ไม่สูบ	พุทธ	ระดับปานก	ระดับปานก	ระดับปานก	ระดับปานก
เคยคลอดม	ตั้งครรภ์มาก	ไม่ดื่ม	ไม่สูบ	พุทธ	ระดับสูง	ระดับสูง	ระดับสูง	ระดับสูง
เคยคลอดม	ตั้งครรภ์มาก	ไม่ดื่ม	ไม่สูบ	พุทธ	ระดับปานก	ระดับปานก	ระดับปานก	ระดับสูง
ไม่เคยคลอ	ตั้งครรภ์ครั้ง	ไม่ดื่ม	ไม่สูบ	พุทธ	ระดับสูง	ระดับสูง	ระดับปานก	ระดับปานก
ไม่เคยคลอ	ตั้งครรภ์ครั้ง	ไม่ดื่ม	ไม่สูบ	พุทธ	ระดับปานก	ระดับปานก	ระดับต่ำ	ระดับสูง
เคยคลอด 1	ตั้งครรภ์มาก	ไม่ดื่ม	ไม่สูบ	พุทธ	ระดับปานก	ระดับปานก	ระดับปานก	ระดับปานก
เคยคลอด 1	ตั้งครรภ์มาก	ไม่ดื่ม	ไม่สูบ	พุทธ	ระดับสูง	ระดับปานก	ระดับปานก	ระดับปานก
ไม่เคยคลอ	ตั้งครรภ์ครั้ง	ไม่ดื่ม	ไม่สูบ	พุทธ	ระดับปานก	ระดับปานก	ระดับปานก	ระดับสูง
เคยคลอดม	ตั้งครรภ์มาก	ไม่ดื่ม	ไม่สูบ	พุทธ	ระดับปานก	ระดับสูง	ระดับปานก	ระดับสูง
ไม่เคยคลอ	ตั้งครรภ์ครั้ง	ไม่ดื่ม	ไม่สูบ	พุทธ	ระดับสูง	ระดับสูง	ระดับปานก	ระดับปานก
ไม่เคยคลอ	ตั้งครรภ์ครั้ง	ไม่ดื่ม	ไม่สูบ	พุทธ	ระดับปานก	ระดับปานก	ระดับปานก	ระดับสูง
ไม่เคยคลอ	ตั้งครรภ์ครั้ง	ไม่ดื่ม	ไม่สูบ	พุทธ	ระดับปานก	ระดับปานก	ระดับปานก	ระดับสูง
เคยคลอด 1	ตั้งครรภ์มาก	ไม่ดื่ม	ไม่สูบ	พุทธ	ระดับปานก	ระดับปานก	ระดับปานก	ระดับสูง
เคยคลอด 1	ตั้งครรภ์มาก	ไม่ดื่ม	ไม่สูบ	พุทธ	ระดับสูง	ระดับสูง	ระดับปานก	ระดับสูง
ไม่เคยคลอ	ตั้งครรภ์ครั้ง	ไม่ดื่ม	ไม่สูบ	พุทธ	ระดับสูง	ระดับสูง	ระดับต่ำ	ระดับปานก
ไม่เคยคลอ	ตั้งครรภ์ครั้ง	ไม่ดื่ม	ไม่สูบ	พุทธ	ระดับปานก	ระดับปานก	ระดับปานก	ระดับสูง
เคยคลอดม	ตั้งครรภ์มาก	ไม่ดื่ม	ไม่สูบ	พุทธ	ระดับสูง	ระดับสูง	ระดับปานก	ระดับสูง
ไม่เคยคลอ	ตั้งครรภ์ครั้ง	ไม่ดื่ม	ไม่สูบ	พุทธ	ระดับสูง	ระดับสูง	ระดับสูง	ระดับปานก
เคยคลอด 1	ตั้งครรภ์มาก	ไม่ดื่ม	ไม่สูบ	พุทธ	ระดับปานก	ระดับปานก	ระดับปานก	ระดับสูง
ไม่เคยคลอ	ตั้งครรภ์ครั้ง	ไม่ดื่ม	ไม่สูบ	พุทธ	ระดับสูง	ระดับสูง	ระดับต่ำ	ระดับปานก
ไม่เคยคลอ	ตั้งครรภ์ครั้ง	ไม่ดื่ม	ไม่สูบ	พุทธ	ระดับปานก	ระดับปานก	ระดับปานก	ระดับต่ำ
ไม่เคยคลอ	ตั้งครรภ์ครั้ง	ไม่ดื่ม	ไม่สูบ	พุทธ	ระดับปานก	ระดับสูง	ระดับปานก	ระดับสูง
เคยคลอด 1	ตั้งครรภ์มาก	ไม่ดื่ม	ไม่สูบ	พุทธ	ระดับปานก	ระดับสูง	ระดับต่ำ	ระดับสูง
ไม่เคยคลอ	ตั้งครรภ์ครั้ง	ไม่ดื่ม	ไม่สูบ	พุทธ	ระดับปานก	ระดับสูง	ระดับปานก	ระดับปานก
เคยคลอดม	ตั้งครรภ์มาก	ไม่ดื่ม	ไม่สูบ	พุทธ	ระดับปานก	ระดับปานก	ระดับปานก	ระดับสูง
ไม่เคยคลอ	ตั้งครรภ์ครั้ง	ไม่ดื่ม	ไม่สูบ	พุทธ	ระดับสูง	ระดับสูง	ระดับปานก	ระดับปานก
ไม่เคยคลอ	ตั้งครรภ์ครั้ง	ไม่ดื่ม	ไม่สูบ	พุทธ	ระดับสูง	ระดับสูง	ระดับสูง	ระดับสูง
ไม่เคยคลอ	ตั้งครรภ์ครั้ง	ไม่ดื่ม	ไม่สูบ	พุทธ	ระดับปานก	ระดับปานก	ระดับปานก	ระดับสูง
ไม่เคยคลอ	ตั้งครรภ์ครั้ง	ไม่ดื่ม	ไม่สูบ	พุทธ	ระดับปานก	ระดับปานก	ระดับปานก	ระดับปานก
เคยคลอด 1	ตั้งครรภ์มาก	ไม่ดื่ม	ไม่สูบ	พุทธ	ระดับปานก	ระดับสูง	ระดับปานก	ระดับสูง
เคยคลอดม	ตั้งครรภ์มาก	ไม่ดื่ม	ไม่สูบ	พุทธ	ระดับปานก	ระดับปานก	ระดับปานก	ระดับสูง
เคยคลอด 1	ตั้งครรภ์มาก	ไม่ดื่ม	ไม่สูบ	พุทธ	ระดับปานก	ระดับสูง	ระดับปานก	ระดับปานก
เคยคลอด 1	ตั้งครรภ์มาก	ไม่ดื่ม	ไม่สูบ	พุทธ	ระดับสูง	ระดับสูง	ระดับปานก	ระดับปานก
ไม่เคยคลอ	ตั้งครรภ์ครั้ง	ไม่ดื่ม	ไม่สูบ	พุทธ	ระดับสูง	ระดับสูง	ระดับสูง	ระดับปานก
ไม่เคยคลอ	ตั้งครรภ์ครั้ง	ไม่ดื่ม	ไม่สูบ	พุทธ	ระดับปานก	ระดับสูง	ระดับปานก	ระดับสูง
เคยคลอด 1	ตั้งครรภ์มาก	ไม่ดื่ม	ไม่สูบ	พุทธ	ระดับปานก	ระดับสูง	ระดับปานก	ระดับสูง
เคยคลอด 1	ตั้งครรภ์มาก	ไม่ดื่ม	ไม่สูบ	พุทธ	ระดับปานก	ระดับปานก	ระดับปานก	ระดับต่ำ
เคยคลอด 1	ตั้งครรภ์มาก	ไม่ดื่ม	ไม่สูบ	พุทธ	ระดับปานก	ระดับสูง	ระดับปานก	ระดับสูง
เคยคลอด 1	ตั้งครรภ์มาก	ไม่ดื่ม	ไม่สูบ	พุทธ	ระดับปานก	ระดับปานก	ระดับปานก	ระดับสูง
เคยคลอด 1	ตั้งครรภ์มาก	ไม่ดื่ม	ไม่สูบ	พุทธ	ระดับปานก	ระดับปานก	ระดับปานก	ระดับปานก
ไม่เคยคลอ	ตั้งครรภ์ครั้ง	ไม่ดื่ม	ไม่สูบ	พุทธ	ระดับสูง	ระดับปานก	ระดับปานก	ระดับสูง
ไม่เคยคลอ	ตั้งครรภ์ครั้ง	ไม่ดื่ม	ไม่สูบ	พุทธ	ระดับปานก	ระดับปานก	ระดับปานก	ระดับสูง
ไม่เคยคลอ	ตั้งครรภ์ครั้ง	ไม่ดื่ม	ไม่สูบ	พุทธ	ระดับปานก	ระดับปานก	ระดับปานก	ระดับสูง

เคยคลอด 1	ตั้งครรภ์มาก	ไม่ดื่ม	ไม่สูบ	พุทธ	ระดับสูง	ระดับสูง	ระดับต่ำ	ระดับสูง
เคยคลอด 1	ตั้งครรภ์มาก	ไม่ดื่ม	ไม่สูบ	พุทธ	ระดับปานก	ระดับปานก	ระดับปานก	ระดับสูง
ไม่เคยคลอ	ตั้งครรภ์ครั้ง	ไม่ดื่ม	ไม่สูบ	พุทธ	ระดับต่ำ	ระดับสูง	ระดับปานก	ระดับสูง
ไม่เคยคลอ	ตั้งครรภ์ครั้ง	ไม่ดื่ม	ไม่สูบ	พุทธ	ระดับปานก	ระดับปานก	ระดับปานก	ระดับสูง
เคยคลอด 1	ตั้งครรภ์มาก	ไม่ดื่ม	ไม่สูบ	พุทธ	ระดับสูง	ระดับปานก	ระดับปานก	ระดับปานก
เคยคลอดม	ตั้งครรภ์มาก	ไม่ดื่ม	ไม่สูบ	พุทธ	ระดับปานก	ระดับปานก	ระดับปานก	ระดับสูง
เคยคลอด 1	ตั้งครรภ์มาก	ไม่ดื่ม	ไม่สูบ	พุทธ	ระดับปานก	ระดับสูง	ระดับปานก	ระดับสูง
เคยคลอด 1	ตั้งครรภ์มาก	ไม่ดื่ม	ไม่สูบ	พุทธ	ระดับสูง	ระดับสูง	ระดับปานก	ระดับสูง
เคยคลอดม	ตั้งครรภ์มาก	ไม่ดื่ม	ไม่สูบ	พุทธ	ระดับสูง	ระดับสูง	ระดับสูง	ระดับปานก
เคยคลอด 1	ตั้งครรภ์มาก	ไม่ดื่ม	ไม่สูบ	พุทธ	ระดับสูง	ระดับปานก	ระดับปานก	ระดับสูง
เคยคลอดม	ตั้งครรภ์มาก	ไม่ดื่ม	ไม่สูบ	พุทธ	ระดับปานก	ระดับปานก	ระดับปานก	ระดับสูง
เคยคลอด 1	ตั้งครรภ์มาก	ไม่ดื่ม	ไม่สูบ	พุทธ	ระดับสูง	ระดับสูง	ระดับต่ำ	ระดับสูง
เคยคลอดม	ตั้งครรภ์มาก	ไม่ดื่ม	ไม่สูบ	พุทธ	ระดับสูง	ระดับสูง	ระดับต่ำ	ระดับปานก
ไม่เคยคลอ	ตั้งครรภ์ครั้ง	ไม่ดื่ม	ไม่สูบ	พุทธ	ระดับปานก	ระดับปานก	ระดับปานก	ระดับสูง
ไม่เคยคลอ	ตั้งครรภ์ครั้ง	ไม่ดื่ม	ไม่สูบ	พุทธ	ระดับปานก	ระดับปานก	ระดับปานก	ระดับปานก
เคยคลอด 1	ตั้งครรภ์มาก	ไม่ดื่ม	ไม่สูบ	พุทธ	ระดับสูง	ระดับสูง	ระดับปานก	ระดับสูง
เคยคลอด 1	ตั้งครรภ์มาก	ไม่ดื่ม	ไม่สูบ	พุทธ	ระดับปานก	ระดับปานก	ระดับปานก	ระดับปานก
ไม่เคยคลอ	ตั้งครรภ์ครั้ง	ไม่ดื่ม	ไม่สูบ	พุทธ	ระดับปานก	ระดับสูง	ระดับต่ำ	ระดับปานก
เคยคลอด 1	ตั้งครรภ์มาก	ไม่ดื่ม	ไม่สูบ	พุทธ	ระดับสูง	ระดับสูง	ระดับต่ำ	ระดับปานก
เคยคลอด 1	ตั้งครรภ์มาก	ไม่ดื่ม	ไม่สูบ	พุทธ	ระดับปานก	ระดับปานก	ระดับปานก	ระดับสูง
ไม่เคยคลอ	ตั้งครรภ์ครั้ง	ไม่ดื่ม	ไม่สูบ	พุทธ	ระดับปานก	ระดับปานก	ระดับปานก	ระดับสูง
ไม่เคยคลอ	ตั้งครรภ์ครั้ง	ไม่ดื่ม	ไม่สูบ	พุทธ	ระดับต่ำ	ระดับปานก	ระดับปานก	ระดับปานก
เคยคลอดม	ตั้งครรภ์มาก	ไม่ดื่ม	ไม่สูบ	พุทธ	ระดับปานก	ระดับปานก	ระดับปานก	ระดับปานก
ไม่เคยคลอ	ตั้งครรภ์ครั้ง	ไม่ดื่ม	ไม่สูบ	พุทธ	ระดับสูง	ระดับปานก	ระดับต่ำ	ระดับสูง
ไม่เคยคลอ	ตั้งครรภ์ครั้ง	ไม่ดื่ม	ไม่สูบ	พุทธ	ระดับปานก	ระดับปานก	ระดับปานก	ระดับปานก
เคยคลอดม	ตั้งครรภ์มาก	ไม่ดื่ม	ไม่สูบ	พุทธ	ระดับปานก	ระดับปานก	ระดับปานก	ระดับปานก
ไม่เคยคลอ	ตั้งครรภ์ครั้ง	ไม่ดื่ม	ไม่สูบ	อื่นๆ	ระดับปานก	ระดับปานก	ระดับปานก	ระดับสูง
เคยคลอด 1	ตั้งครรภ์มาก	ไม่ดื่ม	ไม่สูบ	พุทธ	ระดับสูง	ระดับสูง	ระดับต่ำ	ระดับสูง
เคยคลอด 1	ตั้งครรภ์มาก	ไม่ดื่ม	ไม่สูบ	พุทธ	ระดับปานก	ระดับสูง	ระดับปานก	ระดับสูง
เคยคลอด 1	ตั้งครรภ์มาก	ไม่ดื่ม	ไม่สูบ	พุทธ	ระดับสูง	ระดับสูง	ระดับปานก	ระดับสูง
ไม่เคยคลอ	ตั้งครรภ์ครั้ง	ไม่ดื่ม	ไม่สูบ	พุทธ	ระดับปานก	ระดับปานก	ระดับปานก	ระดับปานก
เคยคลอด 1	ตั้งครรภ์มาก	ไม่ดื่ม	ไม่สูบ	พุทธ	ระดับปานก	ระดับปานก	ระดับปานก	ระดับสูง
เคยคลอด 1	ตั้งครรภ์มาก	ไม่ดื่ม	ไม่สูบ	พุทธ	ระดับปานก	ระดับปานก	ระดับปานก	ระดับสูง
เคยคลอดม	ตั้งครรภ์มาก	ไม่ดื่ม	ไม่สูบ	พุทธ	ระดับปานก	ระดับปานก	ระดับปานก	ระดับสูง
ไม่เคยคลอ	ตั้งครรภ์ครั้ง	ไม่ดื่ม	ไม่สูบ	พุทธ	ระดับปานก	ระดับปานก	ระดับปานก	ระดับสูง
ไม่เคยคลอ	ตั้งครรภ์ครั้ง	ไม่ดื่ม	ไม่สูบ	พุทธ	ระดับปานก	ระดับปานก	ระดับต่ำ	ระดับสูง
ไม่เคยคลอ	ตั้งครรภ์ครั้ง	ไม่ดื่ม	ไม่สูบ	พุทธ	ระดับปานก	ระดับสูง	ระดับปานก	ระดับสูง
เคยคลอด 1	ตั้งครรภ์มาก	ไม่ดื่ม	ไม่สูบ	พุทธ	ระดับปานก	ระดับสูง	ระดับสูง	ระดับสูง
ไม่เคยคลอ	ตั้งครรภ์ครั้ง	ไม่ดื่ม	ไม่สูบ	พุทธ	ระดับปานก	ระดับปานก	ระดับปานก	ระดับปานก
เคยคลอดม	ตั้งครรภ์มาก	ไม่ดื่ม	ไม่สูบ	พุทธ	ระดับสูง	ระดับสูง	ระดับปานก	ระดับสูง
เคยคลอด 1	ตั้งครรภ์มาก	ไม่ดื่ม	ไม่สูบ	พุทธ	ระดับสูง	ระดับสูง	ระดับปานก	ระดับปานก
ไม่เคยคลอ	ตั้งครรภ์ครั้ง	ไม่ดื่ม	ไม่สูบ	พุทธ	ระดับสูง	ระดับสูง	ระดับปานก	ระดับปานก
ไม่เคยคลอ	ตั้งครรภ์ครั้ง	ไม่ดื่ม	ไม่สูบ	พุทธ	ระดับปานก	ระดับสูง	ระดับปานก	ระดับสูง
เคยคลอด 1	ตั้งครรภ์มาก	ไม่ดื่ม	ไม่สูบ	พุทธ	ระดับปานก	ระดับสูง	ระดับปานก	ระดับสูง
ไม่เคยคลอ	ตั้งครรภ์ครั้ง	ไม่ดื่ม	ไม่สูบ	พุทธ	ระดับปานก	ระดับปานก	ระดับปานก	ระดับปานก
ไม่เคยคลอ	ตั้งครรภ์ครั้ง	ไม่ดื่ม	ไม่สูบ	พุทธ	ระดับปานก	ระดับปานก	ระดับปานก	ระดับสูง
ไม่เคยคลอ	ตั้งครรภ์ครั้ง	ไม่ดื่ม	ไม่สูบ	พุทธ	ระดับปานก	ระดับปานก	ระดับปานก	ระดับสูง
ไม่เคยคลอ	ตั้งครรภ์ครั้ง	ไม่ดื่ม	ไม่สูบ	พุทธ	ระดับปานก	ระดับสูง	ระดับปานก	ระดับปานก
ไม่เคยคลอ	ตั้งครรภ์ครั้ง	ไม่ดื่ม	ไม่สูบ	พุทธ	ระดับปานก	ระดับปานก	ระดับปานก	ระดับสูง
ไม่เคยคลอ	ตั้งครรภ์ครั้ง	ไม่ดื่ม	ไม่สูบ	พุทธ	ระดับปานก	ระดับสูง	ระดับปานก	ระดับปานก
ไม่เคยคลอ	ตั้งครรภ์ครั้ง	ไม่ดื่ม	ไม่สูบ	พุทธ	ระดับปานก	ระดับปานก	ระดับปานก	ระดับปานก

เคยคลอด 1	ตั้งครรภ์มา	ไม่ดื่ม	ไม่สูบ	พุทธ	ระดับปานก	ระดับปานก	ระดับปานก	ระดับปานก
เคยคลอดม	ตั้งครรภ์มา	ไม่ดื่ม	ไม่สูบ	พุทธ	ระดับปานก	ระดับปานก	ระดับปานก	ระดับปานก
เคยคลอด 1	ตั้งครรภ์มา	ไม่ดื่ม	ไม่สูบ	พุทธ	ระดับปานก	ระดับปานก	ระดับต่ำ	ระดับสูง
ไม่เคยคลอ	ตั้งครรภ์มา	ไม่ดื่ม	ไม่สูบ	พุทธ	ระดับปานก	ระดับปานก	ระดับปานก	ระดับต่ำ
ไม่เคยคลอ	ตั้งครรภ์ครั้ง	ไม่ดื่ม	ไม่สูบ	พุทธ	ระดับปานก	ระดับสูง	ระดับปานก	ระดับสูง
ไม่เคยคลอ	ตั้งครรภ์ครั้ง	ไม่ดื่ม	ไม่สูบ	พุทธ	ระดับสูง	ระดับสูง	ระดับปานก	ระดับปานก
ไม่เคยคลอ	ตั้งครรภ์ครั้ง	ไม่ดื่ม	ไม่สูบ	พุทธ	ระดับปานก	ระดับปานก	ระดับปานก	ระดับปานก
ไม่เคยคลอ	ตั้งครรภ์ครั้ง	ไม่ดื่ม	ไม่สูบ	พุทธ	ระดับปานก	ระดับสูง	ระดับปานก	ระดับสูง
ไม่เคยคลอ	ตั้งครรภ์ครั้ง	ไม่ดื่ม	ไม่สูบ	พุทธ	ระดับสูง	ระดับปานก	ระดับปานก	ระดับสูง
เคยคลอด 1	ตั้งครรภ์มา	ไม่ดื่ม	ไม่สูบ	พุทธ	ระดับปานก	ระดับสูง	ระดับปานก	ระดับสูง
ไม่เคยคลอ	ตั้งครรภ์ครั้ง	ไม่ดื่ม	ไม่สูบ	พุทธ	ระดับปานก	ระดับปานก	ระดับต่ำ	ระดับสูง
เคยคลอดม	ตั้งครรภ์มา	ไม่ดื่ม	ไม่สูบ	พุทธ	ระดับปานก	ระดับปานก	ระดับปานก	ระดับต่ำ
เคยคลอดม	ตั้งครรภ์มา	ไม่ดื่ม	ไม่สูบ	พุทธ	ระดับสูง	ระดับสูง	ระดับสูง	ระดับต่ำ
เคยคลอดม	ตั้งครรภ์มา	ไม่ดื่ม	ไม่สูบ	พุทธ	ระดับสูง	ระดับสูง	ระดับปานก	ระดับปานก
ไม่เคยคลอ	ตั้งครรภ์ครั้ง	ไม่ดื่ม	ไม่สูบ	พุทธ	ระดับปานก	ระดับสูง	ระดับปานก	ระดับปานก
ไม่เคยคลอ	ตั้งครรภ์ครั้ง	ไม่ดื่ม	ไม่สูบ	พุทธ	ระดับสูง	ระดับสูง	ระดับปานก	ระดับปานก
ไม่เคยคลอ	ตั้งครรภ์ครั้ง	ไม่ดื่ม	ไม่สูบ	พุทธ	ระดับสูง	ระดับสูง	ระดับปานก	ระดับสูง
เคยคลอด 1	ตั้งครรภ์มา	ไม่ดื่ม	ไม่สูบ	พุทธ	ระดับปานก	ระดับปานก	ระดับปานก	ระดับสูง
ไม่เคยคลอ	ตั้งครรภ์ครั้ง	ไม่ดื่ม	ไม่สูบ	พุทธ	ระดับปานก	ระดับสูง	ระดับปานก	ระดับสูง
เคยคลอดม	ตั้งครรภ์มา	ไม่ดื่ม	ไม่สูบ	พุทธ	ระดับสูง	ระดับสูง	ระดับปานก	ระดับปานก
ไม่เคยคลอ	ตั้งครรภ์ครั้ง	ไม่ดื่ม	ไม่สูบ	พุทธ	ระดับปานก	ระดับสูง	ระดับปานก	ระดับต่ำ
ไม่เคยคลอ	ตั้งครรภ์ครั้ง	ไม่ดื่ม	ไม่สูบ	พุทธ	ระดับปานก	ระดับปานก	ระดับสูง	ระดับต่ำ
เคยคลอด 1	ตั้งครรภ์มา	ไม่ดื่ม	ไม่สูบ	พุทธ	ระดับปานก	ระดับปานก	ระดับปานก	ระดับปานก
เคยคลอดม	ตั้งครรภ์มา	ไม่ดื่ม	ไม่สูบ	พุทธ	ระดับสูง	ระดับสูง	ระดับสูง	ระดับปานก
ไม่เคยคลอ	ตั้งครรภ์ครั้ง	ไม่ดื่ม	ไม่สูบ	พุทธ	ระดับปานก	ระดับสูง	ระดับปานก	ระดับสูง
ไม่เคยคลอ	ตั้งครรภ์ครั้ง	ไม่ดื่ม	ไม่สูบ	พุทธ	ระดับปานก	ระดับสูง	ระดับปานก	ระดับสูง
เคยคลอดม	ตั้งครรภ์มา	ไม่ดื่ม	ไม่สูบ	พุทธ	ระดับปานก	ระดับสูง	ระดับสูง	ระดับสูง
ไม่เคยคลอ	ตั้งครรภ์มา	ไม่ดื่ม	ไม่สูบ	พุทธ	ระดับปานก	ระดับปานก	ระดับต่ำ	ระดับสูง
ไม่เคยคลอ	ตั้งครรภ์ครั้ง	ไม่ดื่ม	ไม่สูบ	พุทธ	ระดับปานก	ระดับปานก	ระดับปานก	ระดับปานก
ไม่เคยคลอ	ตั้งครรภ์ครั้ง	ไม่ดื่ม	ไม่สูบ	พุทธ	ระดับปานก	ระดับปานก	ระดับปานก	ระดับสูง
ไม่เคยคลอ	ตั้งครรภ์ครั้ง	ไม่ดื่ม	ไม่สูบ	พุทธ	ระดับปานก	ระดับปานก	ระดับปานก	ระดับปานก
เคยคลอด 1	ตั้งครรภ์มา	ไม่ดื่ม	ไม่สูบ	พุทธ	ระดับสูง	ระดับสูง	ระดับต่ำ	ระดับสูง
ไม่เคยคลอ	ตั้งครรภ์ครั้ง	ไม่ดื่ม	ไม่สูบ	พุทธ	ระดับปานก	ระดับปานก	ระดับปานก	ระดับปานก
เคยคลอด 1	ตั้งครรภ์มา	ไม่ดื่ม	ไม่สูบ	พุทธ	ระดับปานก	ระดับปานก	ระดับปานก	ระดับต่ำ
เคยคลอด 1	ตั้งครรภ์มา	ไม่ดื่ม	ไม่สูบ	พุทธ	ระดับสูง	ระดับสูง	ระดับปานก	ระดับสูง
ไม่เคยคลอ	ตั้งครรภ์ครั้ง	ไม่ดื่ม	ไม่สูบ	พุทธ	ระดับปานก	ระดับสูง	ระดับปานก	ระดับปานก
ไม่เคยคลอ	ตั้งครรภ์ครั้ง	ไม่ดื่ม	ไม่สูบ	พุทธ	ระดับปานก	ระดับสูง	ระดับสูง	ระดับปานก
ไม่เคยคลอ	ตั้งครรภ์ครั้ง	ไม่ดื่ม	ไม่สูบ	พุทธ	ระดับปานก	ระดับสูง	ระดับปานก	ระดับสูง
ไม่เคยคลอ	ตั้งครรภ์ครั้ง	ไม่ดื่ม	ไม่สูบ	พุทธ	ระดับสูง	ระดับสูง	ระดับปานก	ระดับสูง
เคยคลอด 1	ตั้งครรภ์มา	ไม่ดื่ม	ไม่สูบ	พุทธ	ระดับสูง	ระดับสูง	ระดับปานก	ระดับสูง
ไม่เคยคลอ	ตั้งครรภ์ครั้ง	ไม่ดื่ม	ไม่สูบ	พุทธ	ระดับสูง	ระดับสูง	ระดับปานก	ระดับสูง
เคยคลอดม	ตั้งครรภ์มา	ไม่ดื่ม	ไม่สูบ	พุทธ	ระดับสูง	ระดับปานก	ระดับปานก	ระดับต่ำ
เคยคลอดม	ตั้งครรภ์มา	ไม่ดื่ม	ไม่สูบ	พุทธ	ระดับปานก	ระดับสูง	ระดับปานก	ระดับต่ำ
เคยคลอดม	ตั้งครรภ์มา	ไม่ดื่ม	ไม่สูบ	พุทธ	ระดับปานก	ระดับสูง	ระดับปานก	ระดับปานก
ไม่เคยคลอ	ตั้งครรภ์ครั้ง	ไม่ดื่ม	ไม่สูบ	พุทธ	ระดับสูง	ระดับสูง	ระดับต่ำ	ระดับปานก
ไม่เคยคลอ	ตั้งครรภ์ครั้ง	ไม่ดื่ม	ไม่สูบ	พุทธ	ระดับสูง	ระดับสูง	ระดับปานก	ระดับปานก
ไม่เคยคลอ	ตั้งครรภ์ครั้ง	ไม่ดื่ม	ไม่สูบ	พุทธ	ระดับปานก	ระดับปานก	ระดับต่ำ	ระดับสูง
เคยคลอด 1	ตั้งครรภ์มา	ไม่ดื่ม	ไม่สูบ	พุทธ	ระดับปานก	ระดับสูง	ระดับปานก	ระดับสูง
ไม่เคยคลอ	ตั้งครรภ์ครั้ง	ไม่ดื่ม	ไม่สูบ	พุทธ	ระดับปานก	ระดับสูง	ระดับปานก	ระดับสูง
เคยคลอดม	ตั้งครรภ์มา	ไม่ดื่ม	ไม่สูบ	พุทธ	ระดับปานก	ระดับสูง	ระดับปานก	ระดับต่ำ
ไม่เคยคลอ	ตั้งครรภ์ครั้ง	ไม่ดื่ม	ไม่สูบ	พุทธ	ระดับปานก	ระดับปานก	ระดับปานก	ระดับต่ำ

ไม่เคยคลอ	ตั้งครรภ์ครั้ง	ไม่ดื่ม	ไม่สูบ	พุทธ	ระดับปานก	ระดับสูง	ระดับปานก	ระดับต่ำ
เคยคลอด 1	ตั้งครรภ์มาก	ไม่ดื่ม	ไม่สูบ	พุทธ	ระดับปานก	ระดับปานก	ระดับปานก	ระดับปานก
เคยคลอดม	ตั้งครรภ์มาก	ไม่ดื่ม	ไม่สูบ	พุทธ	ระดับปานก	ระดับปานก	ระดับปานก	ระดับปานก
ไม่เคยคลอ	ตั้งครรภ์ครั้ง	ไม่ดื่ม	ไม่สูบ	พุทธ	ระดับสูง	ระดับสูง	ระดับสูง	ระดับสูง
ไม่เคยคลอ	ตั้งครรภ์ครั้ง	ไม่ดื่ม	ไม่สูบ	พุทธ	ระดับปานก	ระดับปานก	ระดับปานก	ระดับสูง
ไม่เคยคลอ	ตั้งครรภ์ครั้ง	ไม่ดื่ม	ไม่สูบ	พุทธ	ระดับสูง	ระดับปานก	ระดับปานก	ระดับต่ำ
เคยคลอด 1	ตั้งครรภ์มาก	ไม่ดื่ม	ไม่สูบ	พุทธ	ระดับสูง	ระดับปานก	ระดับต่ำ	ระดับสูง
ไม่เคยคลอ	ตั้งครรภ์ครั้ง	ไม่ดื่ม	ไม่สูบ	พุทธ	ระดับสูง	ระดับปานก	ระดับปานก	ระดับสูง
เคยคลอดม	ตั้งครรภ์มาก	ไม่ดื่ม	ไม่สูบ	พุทธ	ระดับปานก	ระดับสูง	ระดับปานก	ระดับสูง
ไม่เคยคลอ	ตั้งครรภ์ครั้ง	ไม่ดื่ม	ไม่สูบ	พุทธ	ระดับสูง	ระดับสูง	ระดับปานก	ระดับปานก
ไม่เคยคลอ	ตั้งครรภ์มาก	ไม่ดื่ม	ไม่สูบ	พุทธ	ระดับสูง	ระดับสูง	ระดับปานก	ระดับต่ำ
ไม่เคยคลอ	ตั้งครรภ์ครั้ง	ไม่ดื่ม	ไม่สูบ	พุทธ	ระดับปานก	ระดับปานก	ระดับต่ำ	ระดับสูง
ไม่เคยคลอ	ตั้งครรภ์ครั้ง	ไม่ดื่ม	ไม่สูบ	พุทธ	ระดับต่ำ	ระดับปานก	ระดับปานก	ระดับปานก
ไม่เคยคลอ	ตั้งครรภ์ครั้ง	ไม่ดื่ม	ไม่สูบ	พุทธ	ระดับสูง	ระดับสูง	ระดับปานก	ระดับปานก
ไม่เคยคลอ	ตั้งครรภ์ครั้ง	ไม่ดื่ม	ไม่สูบ	พุทธ	ระดับปานก	ระดับปานก	ระดับปานก	ระดับสูง
ไม่เคยคลอ	ตั้งครรภ์ครั้ง	ไม่ดื่ม	ไม่สูบ	พุทธ	ระดับสูง	ระดับปานก	ระดับปานก	ระดับสูง
เคยคลอด 1	ตั้งครรภ์มาก	ไม่ดื่ม	ไม่สูบ	พุทธ	ระดับสูง	ระดับสูง	ระดับปานก	ระดับสูง
ไม่เคยคลอ	ตั้งครรภ์ครั้ง	ไม่ดื่ม	ไม่สูบ	พุทธ	ระดับปานก	ระดับปานก	ระดับปานก	ระดับสูง
เคยคลอดม	ตั้งครรภ์มาก	ไม่ดื่ม	ไม่สูบ	พุทธ	ระดับสูง	ระดับปานก	ระดับต่ำ	ระดับต่ำ
เคยคลอดม	ตั้งครรภ์มาก	ไม่ดื่ม	ไม่สูบ	พุทธ	ระดับปานก	ระดับปานก	ระดับปานก	ระดับต่ำ
เคยคลอดม	ตั้งครรภ์มาก	ไม่ดื่ม	ไม่สูบ	พุทธ	ระดับสูง	ระดับสูง	ระดับต่ำ	ระดับปานก
ไม่เคยคลอ	ตั้งครรภ์ครั้ง	ไม่ดื่ม	ไม่สูบ	พุทธ	ระดับสูง	ระดับสูง	ระดับปานก	ระดับปานก
ไม่เคยคลอ	ตั้งครรภ์ครั้ง	ไม่ดื่ม	ไม่สูบ	พุทธ	ระดับปานก	ระดับปานก	ระดับปานก	ระดับปานก
ไม่เคยคลอ	ตั้งครรภ์ครั้ง	ไม่ดื่ม	ไม่สูบ	พุทธ	ระดับปานก	ระดับปานก	ระดับปานก	ระดับสูง
เคยคลอด 1	ตั้งครรภ์มาก	ไม่ดื่ม	ไม่สูบ	พุทธ	ระดับปานก	ระดับปานก	ระดับปานก	ระดับสูง
ไม่เคยคลอ	ตั้งครรภ์ครั้ง	ไม่ดื่ม	ไม่สูบ	พุทธ	ระดับสูง	ระดับสูง	ระดับปานก	ระดับปานก
ไม่เคยคลอ	ตั้งครรภ์ครั้ง	ไม่ดื่ม	ไม่สูบ	พุทธ	ระดับปานก	ระดับสูง	ระดับปานก	ระดับสูง
เคยคลอดม	ตั้งครรภ์มาก	ไม่ดื่ม	ไม่สูบ	พุทธ	ระดับสูง	ระดับสูง	ระดับปานก	ระดับปานก
เคยคลอด 1	ตั้งครรภ์มาก	ไม่ดื่ม	ไม่สูบ	พุทธ	ระดับสูง	ระดับสูง	ระดับปานก	ระดับสูง
เคยคลอดม	ตั้งครรภ์มาก	ไม่ดื่ม	ไม่สูบ	พุทธ	ระดับสูง	ระดับสูง	ระดับสูง	ระดับปานก
เคยคลอด 1	ตั้งครรภ์มาก	ไม่ดื่ม	ไม่สูบ	พุทธ	ระดับปานก	ระดับปานก	ระดับปานก	ระดับสูง
ไม่เคยคลอ	ตั้งครรภ์ครั้ง	ไม่ดื่ม	ไม่สูบ	พุทธ	ระดับปานก	ระดับสูง	ระดับสูง	ระดับปานก
ไม่เคยคลอ	ตั้งครรภ์ครั้ง	ไม่ดื่ม	ไม่สูบ	พุทธ	ระดับสูง	ระดับสูง	ระดับปานก	ระดับสูง
เคยคลอดม	ตั้งครรภ์มาก	ไม่ดื่ม	ไม่สูบ	พุทธ	ระดับปานก	ระดับปานก	ระดับปานก	ระดับปานก
ไม่เคยคลอ	ตั้งครรภ์ครั้ง	ไม่ดื่ม	ไม่สูบ	พุทธ	ระดับปานก	ระดับปานก	ระดับต่ำ	ระดับสูง
เคยคลอด 1	ตั้งครรภ์มาก	ไม่ดื่ม	ไม่สูบ	พุทธ	ระดับสูง	ระดับสูง	ระดับต่ำ	ระดับปานก
ไม่เคยคลอ	ตั้งครรภ์ครั้ง	ไม่ดื่ม	ไม่สูบ	พุทธ	ระดับปานก	ระดับปานก	ระดับต่ำ	ระดับสูง
ไม่เคยคลอ	ตั้งครรภ์ครั้ง	ไม่ดื่ม	ไม่สูบ	พุทธ	ระดับปานก	ระดับสูง	ระดับปานก	ระดับสูง
เคยคลอด 1	ตั้งครรภ์มาก	ไม่ดื่ม	ไม่สูบ	พุทธ	ระดับสูง	ระดับสูง	ระดับปานก	ระดับปานก
ไม่เคยคลอ	ตั้งครรภ์ครั้ง	ไม่ดื่ม	ไม่สูบ	พุทธ	ระดับปานก	ระดับสูง	ระดับปานก	ระดับสูง
ไม่เคยคลอ	ตั้งครรภ์ครั้ง	ไม่ดื่ม	ไม่สูบ	พุทธ	ระดับปานก	ระดับปานก	ระดับปานก	ระดับสูง
ไม่เคยคลอ	ตั้งครรภ์ครั้ง	ไม่ดื่ม	ไม่สูบ	พุทธ	ระดับปานก	ระดับสูง	ระดับปานก	ระดับสูง
เคยคลอด 1	ตั้งครรภ์มาก	ไม่ดื่ม	ไม่สูบ	พุทธ	ระดับสูง	ระดับสูง	ระดับปานก	ระดับสูง
เคยคลอด 1	ตั้งครรภ์มาก	ไม่ดื่ม	ไม่สูบ	พุทธ	ระดับปานก	ระดับปานก	ระดับปานก	ระดับสูง
ไม่เคยคลอ	ตั้งครรภ์ครั้ง	ไม่ดื่ม	ไม่สูบ	พุทธ	ระดับปานก	ระดับปานก	ระดับปานก	ระดับสูง
เคยคลอด 1	ตั้งครรภ์มาก	ไม่ดื่ม	ไม่สูบ	พุทธ	ระดับสูง	ระดับสูง	ระดับสูง	ระดับปานก
ไม่เคยคลอ	ตั้งครรภ์ครั้ง	ไม่ดื่ม	ไม่สูบ	พุทธ	ระดับปานก	ระดับปานก	ระดับปานก	ระดับสูง
ไม่เคยคลอ	ตั้งครรภ์ครั้ง	ไม่ดื่ม	ไม่สูบ	พุทธ	ระดับสูง	ระดับสูง	ระดับปานก	ระดับปานก
เคยคลอดม	ตั้งครรภ์มาก	ไม่ดื่ม	ไม่สูบ	พุทธ	ระดับปานก	ระดับปานก	ระดับปานก	ระดับสูง
ไม่เคยคลอ	ตั้งครรภ์ครั้ง	ไม่ดื่ม	ไม่สูบ	พุทธ	ระดับปานก	ระดับปานก	ระดับปานก	ระดับปานก
เคยคลอด 1	ตั้งครรภ์มาก	ไม่ดื่ม	ไม่สูบ	พุทธ	ระดับปานก	ระดับปานก	ระดับปานก	ระดับสูง

ไม่เคยคลอ	ตั้งครรภ์ครั้ง	ไม่ดื่ม	ไม่สูบ	พุทธ	ระดับปานก	ระดับปานก	ระดับปานก	ระดับปานก
ไม่เคยคลอ	ตั้งครรภ์ครั้ง	ไม่ดื่ม	ไม่สูบ	พุทธ	ระดับสูง	ระดับปานก	ระดับปานก	ระดับต่ำ
ไม่เคยคลอ	ตั้งครรภ์ครั้ง	ไม่ดื่ม	ไม่สูบ	พุทธ	ระดับปานก	ระดับสูง	ระดับต่ำ	ระดับสูง
เคยคลอด 1	ตั้งครรภ์มาก	ไม่ดื่ม	ไม่สูบ	พุทธ	ระดับปานก	ระดับปานก	ระดับปานก	ระดับสูง
ไม่เคยคลอ	ตั้งครรภ์ครั้ง	ไม่ดื่ม	ไม่สูบ	พุทธ	ระดับปานก	ระดับสูง	ระดับปานก	ระดับปานก
เคยคลอดม	ตั้งครรภ์มาก	ไม่ดื่ม	ไม่สูบ	พุทธ	ระดับปานก	ระดับปานก	ระดับปานก	ระดับสูง
ไม่เคยคลอ	ตั้งครรภ์ครั้ง	ไม่ดื่ม	ไม่สูบ	พุทธ	ระดับสูง	ระดับสูง	ระดับสูง	ระดับปานก
เคยคลอด 1	ตั้งครรภ์มาก	ไม่ดื่ม	ไม่สูบ	พุทธ	ระดับสูง	ระดับสูง	ระดับต่ำ	ระดับปานก
เคยคลอด 1	ตั้งครรภ์มาก	ไม่ดื่ม	ไม่สูบ	พุทธ	ระดับปานก	ระดับปานก	ระดับปานก	ระดับสูง
เคยคลอดม	ตั้งครรภ์มาก	ไม่ดื่ม	ไม่สูบ	พุทธ	ระดับปานก	ระดับปานก	ระดับต่ำ	ระดับสูง
เคยคลอด 1	ตั้งครรภ์มาก	ไม่ดื่ม	ไม่สูบ	พุทธ	ระดับปานก	ระดับสูง	ระดับต่ำ	ระดับปานก
เคยคลอด 1	ตั้งครรภ์มาก	ไม่ดื่ม	ไม่สูบ	พุทธ	ระดับสูง	ระดับสูง	ระดับต่ำ	ระดับปานก
ไม่เคยคลอ	ตั้งครรภ์ครั้ง	ไม่ดื่ม	ไม่สูบ	พุทธ	ระดับปานก	ระดับสูง	ระดับปานก	ระดับปานก
ไม่เคยคลอ	ตั้งครรภ์ครั้ง	ไม่ดื่ม	ไม่สูบ	พุทธ	ระดับสูง	ระดับสูง	ระดับปานก	ระดับสูง
เคยคลอด 1	ตั้งครรภ์มาก	ไม่ดื่ม	ไม่สูบ	พุทธ	ระดับปานก	ระดับปานก	ระดับปานก	ระดับสูง
เคยคลอด 1	ตั้งครรภ์มาก	ไม่ดื่ม	ไม่สูบ	พุทธ	ระดับสูง	ระดับสูง	ระดับสูง	ระดับต่ำ
เคยคลอด 1	ตั้งครรภ์มาก	ไม่ดื่ม	ไม่สูบ	พุทธ	ระดับปานก	ระดับปานก	ระดับปานก	ระดับสูง
เคยคลอด 1	ตั้งครรภ์มาก	ไม่ดื่ม	ไม่สูบ	พุทธ	ระดับปานก	ระดับสูง	ระดับปานก	ระดับสูง
เคยคลอด 1	ตั้งครรภ์มาก	ไม่ดื่ม	ไม่สูบ	พุทธ	ระดับปานก	ระดับสูง	ระดับสูง	ระดับปานก
ไม่เคยคลอ	ตั้งครรภ์ครั้ง	ไม่ดื่ม	ไม่สูบ	พุทธ	ระดับสูง	ระดับปานก	ระดับต่ำ	ระดับสูง
ไม่เคยคลอ	ตั้งครรภ์ครั้ง	ไม่ดื่ม	ไม่สูบ	พุทธ	ระดับสูง	ระดับสูง	ระดับปานก	ระดับสูง
ไม่เคยคลอ	ตั้งครรภ์ครั้ง	ไม่ดื่ม	ไม่สูบ	พุทธ	ระดับปานก	ระดับสูง	ระดับต่ำ	ระดับสูง
เคยคลอด 1	ตั้งครรภ์มาก	ไม่ดื่ม	ไม่สูบ	พุทธ	ระดับสูง	ระดับสูง	ระดับต่ำ	ระดับสูง
เคยคลอด 1	ตั้งครรภ์มาก	ไม่ดื่ม	ไม่สูบ	พุทธ	ระดับสูง	ระดับสูง	ระดับต่ำ	ระดับปานก
เคยคลอดม	ตั้งครรภ์มาก	ไม่ดื่ม	ไม่สูบ	พุทธ	ระดับสูง	ระดับสูง	ระดับปานก	ระดับสูง
เคยคลอดม	ตั้งครรภ์มาก	ไม่ดื่ม	ไม่สูบ	พุทธ	ระดับปานก	ระดับปานก	ระดับปานก	ระดับสูง
ไม่เคยคลอ	ตั้งครรภ์ครั้ง	ไม่ดื่ม	ไม่สูบ	พุทธ	ระดับปานก	ระดับปานก	ระดับปานก	ระดับปานก
ไม่เคยคลอ	ตั้งครรภ์ครั้ง	ไม่ดื่ม	ไม่สูบ	พุทธ	ระดับปานก	ระดับปานก	ระดับปานก	ระดับสูง
เคยคลอด 1	ตั้งครรภ์มาก	ไม่ดื่ม	ไม่สูบ	พุทธ	ระดับปานก	ระดับปานก	ระดับปานก	ระดับปานก
เคยคลอด 1	ตั้งครรภ์มาก	ไม่ดื่ม	ไม่สูบ	พุทธ	ระดับสูง	ระดับสูง	ระดับปานก	ระดับปานก
ไม่เคยคลอ	ตั้งครรภ์ครั้ง	ไม่ดื่ม	ไม่สูบ	พุทธ	ระดับปานก	ระดับสูง	ระดับปานก	ระดับสูง
เคยคลอดม	ตั้งครรภ์มาก	ไม่ดื่ม	ไม่สูบ	พุทธ	ระดับปานก	ระดับปานก	ระดับปานก	ระดับสูง
ไม่เคยคลอ	ตั้งครรภ์ครั้ง	ไม่ดื่ม	ไม่สูบ	พุทธ	ระดับปานก	ระดับสูง	ระดับสูง	ระดับปานก
ไม่เคยคลอ	ตั้งครรภ์ครั้ง	ไม่ดื่ม	ไม่สูบ	พุทธ	ระดับสูง	ระดับปานก	ระดับต่ำ	ระดับสูง
ไม่เคยคลอ	ตั้งครรภ์ครั้ง	ไม่ดื่ม	ไม่สูบ	พุทธ	ระดับปานก	ระดับปานก	ระดับปานก	ระดับสูง
เคยคลอด 1	ตั้งครรภ์มาก	ไม่ดื่ม	ไม่สูบ	พุทธ	ระดับปานก	ระดับปานก	ระดับปานก	ระดับสูง
เคยคลอด 1	ตั้งครรภ์มาก	ไม่ดื่ม	ไม่สูบ	พุทธ	ระดับสูง	ระดับสูง	ระดับปานก	ระดับสูง
ไม่เคยคลอ	ตั้งครรภ์ครั้ง	ไม่ดื่ม	ไม่สูบ	พุทธ	ระดับปานก	ระดับสูง	ระดับปานก	ระดับปานก
ไม่เคยคลอ	ตั้งครรภ์ครั้ง	ไม่ดื่ม	ไม่สูบ	พุทธ	ระดับสูง	ระดับปานก	ระดับปานก	ระดับสูง
เคยคลอดม	ตั้งครรภ์มาก	ไม่ดื่ม	ไม่สูบ	พุทธ	ระดับปานก	ระดับปานก	ระดับปานก	ระดับสูง
ไม่เคยคลอ	ตั้งครรภ์ครั้ง	ไม่ดื่ม	ไม่สูบ	พุทธ	ระดับปานก	ระดับปานก	ระดับปานก	ระดับปานก
เคยคลอด 1	ตั้งครรภ์มาก	ไม่ดื่ม	ไม่สูบ	พุทธ	ระดับปานก	ระดับสูง	ระดับปานก	ระดับสูง
ไม่เคยคลอ	ตั้งครรภ์ครั้ง	ไม่ดื่ม	ไม่สูบ	พุทธ	ระดับปานก	ระดับปานก	ระดับปานก	ระดับปานก
ไม่เคยคลอ	ตั้งครรภ์ครั้ง	ไม่ดื่ม	ไม่สูบ	พุทธ	ระดับปานก	ระดับปานก	ระดับปานก	ระดับต่ำ
ไม่เคยคลอ	ตั้งครรภ์ครั้ง	ไม่ดื่ม	ไม่สูบ	พุทธ	ระดับสูง	ระดับสูง	ระดับปานก	ระดับสูง
เคยคลอด 1	ตั้งครรภ์มาก	ไม่ดื่ม	ไม่สูบ	พุทธ	ระดับปานก	ระดับปานก	ระดับปานก	ระดับสูง
ไม่เคยคลอ	ตั้งครรภ์ครั้ง	ไม่ดื่ม	ไม่สูบ	พุทธ	ระดับสูง	ระดับสูง	ระดับปานก	ระดับสูง
ไม่เคยคลอ	ตั้งครรภ์ครั้ง	ไม่ดื่ม	ไม่สูบ	พุทธ	ระดับปานก	ระดับปานก	ระดับปานก	ระดับสูง
ไม่เคยคลอ	ตั้งครรภ์ครั้ง	ไม่ดื่ม	ไม่สูบ	พุทธ	ระดับสูง	ระดับสูง	ระดับปานก	ระดับสูง

Behavior_(	Benefit_G	กลุ่มอายุ	กลุ่มอาชีพ	กลุ่มรายได้	กลุ่มรายจ่าย
พฤติกรรมดี	ระดับปานก	2.00	1.00	1.00	1.00
พฤติกรรมดี	ระดับสูง	2.00	1.00	1.00	1.00
พฤติกรรมดี	ระดับสูง	2.00	1.00	1.00	1.00
พฤติกรรมไ	ระดับปานก	3.00	2.00	3.00	2.00
พฤติกรรมไ	ระดับปานก	5.00	1.00	1.00	1.00
พฤติกรรมดี	ระดับสูง	2.00	3.00	1.00	1.00
พฤติกรรมไ	ระดับปานก	2.00	3.00	1.00	1.00
พฤติกรรมดี	ระดับสูง	3.00	2.00	1.00	1.00
พฤติกรรมไ	ระดับปานก	1.00	1.00	1.00	2.00
พฤติกรรมดี	ระดับปานก	1.00	3.00	1.00	1.00
พฤติกรรมดี	ระดับปานก	4.00	3.00	1.00	1.00
พฤติกรรมดี	ระดับปานก	5.00	1.00	2.00	2.00
พฤติกรรมไ	ระดับสูง	2.00	2.00	2.00	1.00
พฤติกรรมไ	ระดับปานก	2.00	1.00	2.00	1.00
พฤติกรรมไ	ระดับปานก	2.00	3.00	1.00	1.00
พฤติกรรมไ	ระดับปานก	3.00	1.00	1.00	1.00
พฤติกรรมดี	ระดับปานก	2.00	3.00	2.00	4.00
พฤติกรรมไ	ระดับสูง	2.00	3.00	2.00	2.00
พฤติกรรมไ	ระดับปานก	3.00	3.00	2.00	2.00
พฤติกรรมดี	ระดับสูง	4.00	2.00	3.00	2.00
พฤติกรรมดี	ระดับปานก	2.00	2.00	2.00	2.00
พฤติกรรมดี	ระดับปานก	1.00	1.00	1.00	1.00
พฤติกรรมดี	ระดับสูง	3.00	2.00	3.00	2.00
พฤติกรรมดี	ระดับปานก	2.00	1.00	1.00	1.00
พฤติกรรมดี	ระดับปานก	3.00	3.00	2.00	1.00
พฤติกรรมดี	ระดับสูง	4.00	1.00	2.00	2.00
พฤติกรรมดี	ระดับสูง	1.00	1.00	1.00	4.00
พฤติกรรมดี	ระดับสูง	1.00	1.00	1.00	1.00
พฤติกรรมดี	ระดับสูง	1.00	1.00	2.00	1.00
พฤติกรรมดี	ระดับสูง	3.00	3.00	1.00	1.00
พฤติกรรมไ	ระดับปานก	3.00	3.00	2.00	1.00
พฤติกรรมดี	ระดับปานก	2.00	1.00	1.00	1.00
พฤติกรรมดี	ระดับสูง	3.00	3.00	2.00	1.00
พฤติกรรมดี	ระดับสูง	3.00	3.00	1.00	1.00
พฤติกรรมดี	ระดับสูง	4.00	3.00	3.00	2.00
พฤติกรรมดี	ระดับปานก	3.00	3.00	1.00	1.00
พฤติกรรมไ	ระดับสูง	1.00	1.00	1.00	1.00
พฤติกรรมดี	ระดับสูง	2.00	1.00	2.00	2.00
พฤติกรรมไ	ระดับปานก	3.00	1.00	1.00	1.00
พฤติกรรมดี	ระดับสูง	1.00	1.00	1.00	1.00
พฤติกรรมดี	ระดับสูง	3.00	3.00	2.00	3.00
พฤติกรรมดี	ระดับปานก	5.00	1.00	2.00	2.00
พฤติกรรมดี	ระดับปานก	2.00	3.00	1.00	1.00
พฤติกรรมไ	ระดับปานก	2.00	1.00	2.00	2.00
พฤติกรรมดี	ระดับปานก	1.00	1.00	1.00	1.00
พฤติกรรมดี	ระดับปานก	3.00	1.00	#NULL!	#NULL!
พฤติกรรมดี	ระดับปานก	2.00	3.00	2.00	1.00
พฤติกรรมไ	ระดับปานก	4.00	3.00	2.00	1.00
พฤติกรรมไ	ระดับสูง	4.00	3.00	1.00	1.00
พฤติกรรมไ	ระดับสูง	4.00	1.00	#NULL!	#NULL!

พฤติกรรมดี	ระดับสูง	3.00	3.00	2.00	2.00
พฤติกรรมไ	ระดับสูง	5.00	3.00	#NULL!	#NULL!
พฤติกรรมดี	ระดับปานก	1.00	3.00	1.00	1.00
พฤติกรรมดี	ระดับปานก	4.00	1.00	2.00	2.00
พฤติกรรมไ	ระดับสูง	3.00	1.00	2.00	2.00
พฤติกรรมดี	ระดับสูง	2.00	3.00	2.00	4.00
พฤติกรรมไ	ระดับปานก	4.00	2.00	2.00	3.00
พฤติกรรมไ	ระดับสูง	1.00	1.00	1.00	1.00
พฤติกรรมดี	ระดับสูง	1.00	1.00	#NULL!	#NULL!
พฤติกรรมไ	ระดับสูง	5.00	1.00	2.00	1.00
พฤติกรรมดี	ระดับสูง	4.00	3.00	1.00	1.00
พฤติกรรมไ	ระดับสูง	4.00	3.00	1.00	2.00
พฤติกรรมดี	ระดับปานก	4.00	1.00	#NULL!	#NULL!
พฤติกรรมดี	ระดับสูง	4.00	3.00	2.00	2.00
พฤติกรรมดี	ระดับสูง	5.00	2.00	3.00	2.00
พฤติกรรมดี	ระดับสูง	1.00	3.00	1.00	1.00
พฤติกรรมไ	ระดับสูง	3.00	1.00	1.00	1.00
พฤติกรรมไ	ระดับสูง	3.00	1.00	2.00	1.00
พฤติกรรมไ	ระดับปานก	2.00	3.00	1.00	1.00
พฤติกรรมไ	ระดับปานก	1.00	1.00	1.00	1.00
พฤติกรรมดี	ระดับสูง	1.00	3.00	1.00	2.00
พฤติกรรมดี	ระดับสูง	5.00	3.00	4.00	4.00
พฤติกรรมดี	ระดับปานก	4.00	2.00	2.00	1.00
พฤติกรรมดี	ระดับปานก	5.00	3.00	1.00	1.00
พฤติกรรมดี	ระดับปานก	1.00	3.00	1.00	1.00
พฤติกรรมไ	ระดับปานก	4.00	3.00	3.00	2.00
พฤติกรรมไ	ระดับปานก	4.00	2.00	3.00	2.00
พฤติกรรมดี	ระดับปานก	2.00	3.00	2.00	2.00
พฤติกรรมดี	ระดับปานก	2.00	1.00	1.00	1.00
พฤติกรรมดี	ระดับปานก	1.00	1.00	2.00	1.00
พฤติกรรมไ	ระดับปานก	5.00	3.00	#NULL!	#NULL!
พฤติกรรมดี	ระดับสูง	4.00	1.00	2.00	2.00
พฤติกรรมดี	ระดับสูง	1.00	1.00	1.00	1.00
พฤติกรรมไ	ระดับปานก	2.00	3.00	2.00	2.00
พฤติกรรมไ	ระดับสูง	4.00	3.00	2.00	2.00
พฤติกรรมไ	ระดับสูง	3.00	3.00	2.00	1.00
พฤติกรรมดี	ระดับสูง	2.00	1.00	2.00	2.00
พฤติกรรมไ	ระดับสูง	2.00	3.00	2.00	4.00
พฤติกรรมดี	ระดับสูง	2.00	3.00	4.00	1.00
พฤติกรรมดี	ระดับสูง	2.00	3.00	2.00	2.00
พฤติกรรมไ	ระดับสูง	5.00	3.00	2.00	1.00
พฤติกรรมดี	ระดับปานก	5.00	3.00	2.00	2.00
พฤติกรรมดี	ระดับสูง	2.00	1.00	2.00	1.00
พฤติกรรมดี	ระดับปานก	4.00	1.00	2.00	2.00
พฤติกรรมไ	ระดับปานก	3.00	3.00	2.00	1.00
พฤติกรรมดี	ระดับปานก	2.00	3.00	4.00	2.00
พฤติกรรมดี	ระดับสูง	2.00	3.00	2.00	1.00
พฤติกรรมดี	ระดับปานก	3.00	1.00	1.00	1.00
พฤติกรรมไ	ระดับปานก	4.00	3.00	1.00	1.00
พฤติกรรมดี	ระดับสูง	2.00	1.00	1.00	1.00
พฤติกรรมดี	ระดับปานก	3.00	2.00	2.00	1.00

พฤติกรรมดี	ระดับปานก	3.00	3.00	2.00	2.00
พฤติกรรมดี	ระดับสูง	5.00	3.00	2.00	2.00
พฤติกรรมไ	ระดับสูง	4.00	1.00	1.00	1.00
พฤติกรรมไ	ระดับปานก	4.00	3.00	2.00	2.00
พฤติกรรมดี	ระดับสูง	4.00	2.00	2.00	2.00
พฤติกรรมไ	ระดับสูง	2.00	3.00	2.00	2.00
พฤติกรรมดี	ระดับสูง	2.00	1.00	1.00	1.00
พฤติกรรมดี	ระดับสูง	2.00	3.00	1.00	1.00
พฤติกรรมดี	ระดับสูง	2.00	1.00	1.00	1.00
พฤติกรรมดี	ระดับสูง	2.00	3.00	1.00	1.00
พฤติกรรมดี	ระดับปานก	2.00	1.00	1.00	1.00
พฤติกรรมดี	ระดับปานก	1.00	3.00	1.00	#NULL!
พฤติกรรมไ	ระดับปานก	5.00	3.00	1.00	1.00
พฤติกรรมไ	ระดับสูง	2.00	1.00	2.00	2.00
พฤติกรรมดี	ระดับปานก	5.00	3.00	4.00	3.00
พฤติกรรมไ	ระดับสูง	2.00	1.00	1.00	2.00
พฤติกรรมดี	ระดับสูง	2.00	1.00	2.00	2.00
พฤติกรรมไ	ระดับสูง	4.00	1.00	1.00	1.00
พฤติกรรมไ	ระดับสูง	2.00	3.00	1.00	1.00
พฤติกรรมดี	ระดับปานก	4.00	3.00	2.00	2.00
พฤติกรรมดี	ระดับสูง	2.00	3.00	1.00	1.00
พฤติกรรมไ	ระดับสูง	2.00	2.00	2.00	1.00
พฤติกรรมไ	ระดับปานก	2.00	3.00	2.00	2.00
พฤติกรรมดี	ระดับสูง	2.00	1.00	1.00	1.00
พฤติกรรมไ	ระดับสูง	2.00	3.00	1.00	1.00
พฤติกรรมดี	ระดับสูง	2.00	1.00	#NULL!	#NULL!
พฤติกรรมดี	ระดับปานก	2.00	1.00	2.00	1.00
พฤติกรรมดี	ระดับปานก	4.00	3.00	2.00	2.00
พฤติกรรมดี	ระดับต่ำ	3.00	3.00	2.00	1.00
พฤติกรรมไ	ระดับสูง	5.00	2.00	2.00	2.00
พฤติกรรมไ	ระดับสูง	2.00	1.00	1.00	1.00
พฤติกรรมไ	ระดับสูง	2.00	3.00	2.00	1.00
พฤติกรรมไ	ระดับปานก	3.00	3.00	2.00	2.00
พฤติกรรมดี	ระดับปานก	1.00	1.00	#NULL!	1.00
พฤติกรรมไ	ระดับสูง	1.00	3.00	2.00	1.00
พฤติกรรมไ	ระดับปานก	4.00	1.00	1.00	1.00
พฤติกรรมดี	ระดับปานก	2.00	1.00	1.00	1.00
พฤติกรรมไ	ระดับสูง	2.00	3.00	2.00	1.00
พฤติกรรมไ	ระดับปานก	1.00	1.00	1.00	1.00
พฤติกรรมดี	ระดับปานก	2.00	1.00	2.00	2.00
พฤติกรรมดี	ระดับปานก	2.00	1.00	2.00	1.00
พฤติกรรมดี	ระดับสูง	1.00	3.00	1.00	1.00
พฤติกรรมดี	ระดับปานก	4.00	3.00	#NULL!	#NULL!
พฤติกรรมไ	ระดับปานก	4.00	3.00	2.00	2.00
พฤติกรรมดี	ระดับปานก	3.00	2.00	2.00	2.00
พฤติกรรมไ	ระดับสูง	2.00	1.00	1.00	1.00
พฤติกรรมดี	ระดับปานก	2.00	3.00	2.00	1.00
พฤติกรรมดี	ระดับสูง	2.00	3.00	2.00	1.00
พฤติกรรมดี	ระดับสูง	2.00	1.00	1.00	1.00
พฤติกรรมดี	ระดับปานก	2.00	3.00	4.00	4.00
พฤติกรรมไ	ระดับปานก	2.00	3.00	1.00	1.00

พฤติกรรมไ	ระดับปานก	5.00	1.00	1.00	1.00
พฤติกรรมดี	ระดับปานก	3.00	3.00	1.00	1.00
พฤติกรรมไ	ระดับปานก	2.00	3.00	2.00	1.00
พฤติกรรมดี	ระดับปานก	4.00	3.00	2.00	1.00
พฤติกรรมดี	ระดับปานก	4.00	2.00	2.00	1.00
พฤติกรรมดี	ระดับสูง	2.00	1.00	2.00	2.00
พฤติกรรมไ	ระดับปานก	2.00	3.00	2.00	1.00
พฤติกรรมไ	ระดับปานก	5.00	3.00	1.00	2.00
พฤติกรรมไ	ระดับสูง	4.00	3.00	1.00	1.00
พฤติกรรมไ	ระดับสูง	3.00	3.00	2.00	1.00
พฤติกรรมดี	ระดับสูง	4.00	2.00	3.00	2.00
พฤติกรรมไ	ระดับสูง	2.00	3.00	2.00	1.00
พฤติกรรมดี	ระดับปานก	3.00	3.00	2.00	2.00
พฤติกรรมไ	ระดับปานก	5.00	3.00	1.00	1.00
พฤติกรรมดี	ระดับปานก	4.00	3.00	1.00	1.00
พฤติกรรมไ	ระดับปานก	2.00	3.00	3.00	2.00
พฤติกรรมไ	ระดับสูง	5.00	3.00	1.00	1.00
พฤติกรรมไ	ระดับสูง	2.00	1.00	4.00	1.00
พฤติกรรมดี	ระดับปานก	4.00	3.00	2.00	2.00
พฤติกรรมไ	ระดับปานก	2.00	3.00	1.00	1.00
พฤติกรรมดี	ระดับปานก	3.00	3.00	4.00	2.00
พฤติกรรมไ	ระดับสูง	2.00	3.00	1.00	1.00
พฤติกรรมไ	ระดับปานก	2.00	1.00	1.00	1.00
พฤติกรรมดี	ระดับปานก	2.00	3.00	1.00	1.00
พฤติกรรมดี	ระดับสูง	4.00	3.00	2.00	2.00
พฤติกรรมไ	ระดับสูง	2.00	3.00	1.00	1.00
พฤติกรรมดี	ระดับปานก	4.00	1.00	2.00	2.00
พฤติกรรมไ	ระดับสูง	1.00	1.00	1.00	1.00
พฤติกรรมไ	ระดับปานก	2.00	3.00	2.00	2.00
พฤติกรรมไ	ระดับสูง	2.00	3.00	3.00	2.00
พฤติกรรมดี	ระดับสูง	4.00	3.00	1.00	1.00
พฤติกรรมดี	ระดับปานก	2.00	3.00	2.00	1.00
พฤติกรรมดี	ระดับปานก	2.00	1.00	1.00	1.00
พฤติกรรมไ	ระดับสูง	1.00	1.00	#NULL!	#NULL!
พฤติกรรมดี	ระดับสูง	3.00	1.00	3.00	2.00
พฤติกรรมดี	ระดับสูง	3.00	3.00	2.00	2.00
พฤติกรรมไ	ระดับต่ำ	3.00	1.00	1.00	1.00
พฤติกรรมดี	ระดับสูง	3.00	3.00	2.00	1.00
พฤติกรรมดี	ระดับปานก	3.00	3.00	2.00	1.00
พฤติกรรมไ	ระดับสูง	2.00	1.00	1.00	1.00
พฤติกรรมไ	ระดับสูง	3.00	3.00	2.00	2.00
พฤติกรรมดี	ระดับสูง	2.00	1.00	2.00	2.00
พฤติกรรมดี	ระดับปานก	2.00	2.00	2.00	2.00
พฤติกรรมดี	ระดับปานก	4.00	3.00	2.00	1.00
พฤติกรรมดี	ระดับปานก	4.00	3.00	#NULL!	#NULL!
พฤติกรรมดี	ระดับปานก	3.00	2.00	3.00	2.00
พฤติกรรมไ	ระดับสูง	2.00	1.00	2.00	1.00
พฤติกรรมไ	ระดับปานก	5.00	3.00	2.00	2.00
พฤติกรรมดี	ระดับปานก	1.00	1.00	2.00	1.00
พฤติกรรมดี	ระดับปานก	2.00	1.00	1.00	1.00
พฤติกรรมดี	ระดับปานก	3.00	3.00	4.00	4.00

พฤติกรรมดี	ระดับสูง	5.00	3.00	2.00	2.00
พฤติกรรมดี	ระดับปานก	3.00	3.00	2.00	1.00
พฤติกรรมดี	ระดับสูง	2.00	3.00	2.00	1.00
พฤติกรรมไ	ระดับปานก	3.00	3.00	2.00	1.00
พฤติกรรมดี	ระดับสูง	3.00	3.00	2.00	2.00
พฤติกรรมไ	ระดับปานก	5.00	2.00	3.00	3.00
พฤติกรรมดี	ระดับสูง	2.00	1.00	4.00	4.00
พฤติกรรมไ	ระดับปานก	4.00	3.00	2.00	1.00
พฤติกรรมไ	ระดับสูง	4.00	3.00	1.00	1.00
พฤติกรรมดี	ระดับปานก	4.00	3.00	2.00	2.00
พฤติกรรมดี	ระดับปานก	5.00	3.00	2.00	2.00
พฤติกรรมดี	ระดับสูง	3.00	3.00	2.00	1.00
พฤติกรรมดี	ระดับสูง	4.00	3.00	2.00	1.00
พฤติกรรมดี	ระดับปานก	3.00	3.00	2.00	2.00
พฤติกรรมดี	ระดับปานก	2.00	3.00	2.00	2.00
พฤติกรรมดี	ระดับปานก	3.00	3.00	2.00	2.00
พฤติกรรมไ	ระดับปานก	4.00	3.00	2.00	1.00
พฤติกรรมไ	ระดับสูง	1.00	1.00	2.00	2.00
พฤติกรรมดี	ระดับสูง	3.00	3.00	2.00	1.00
พฤติกรรมดี	ระดับปานก	5.00	3.00	2.00	2.00
พฤติกรรมดี	ระดับปานก	4.00	2.00	3.00	2.00
พฤติกรรมดี	ระดับปานก	2.00	3.00	2.00	1.00
พฤติกรรมไ	ระดับสูง	2.00	1.00	1.00	1.00
พฤติกรรมดี	ระดับสูง	4.00	3.00	1.00	#NULL!
พฤติกรรมไ	ระดับปานก	2.00	1.00	#NULL!	#NULL!
พฤติกรรมดี	ระดับปานก	5.00	3.00	#NULL!	#NULL!
พฤติกรรมดี	ระดับสูง	1.00	1.00	#NULL!	#NULL!
พฤติกรรมดี	ระดับสูง	5.00	3.00	2.00	2.00
พฤติกรรมดี	ระดับสูง	4.00	2.00	2.00	2.00
พฤติกรรมดี	ระดับสูง	3.00	3.00	2.00	1.00
พฤติกรรมดี	ระดับปานก	3.00	3.00	2.00	2.00
พฤติกรรมดี	ระดับปานก	4.00	3.00	2.00	2.00
พฤติกรรมดี	ระดับปานก	3.00	2.00	2.00	1.00
พฤติกรรมดี	ระดับปานก	3.00	2.00	2.00	2.00
พฤติกรรมดี	ระดับปานก	1.00	1.00	4.00	2.00
พฤติกรรมไ	ระดับปานก	3.00	2.00	2.00	2.00
พฤติกรรมไ	ระดับสูง	2.00	1.00	1.00	1.00
พฤติกรรมดี	ระดับสูง	3.00	3.00	2.00	2.00
พฤติกรรมดี	ระดับปานก	2.00	1.00	2.00	1.00
พฤติกรรมดี	ระดับสูง	4.00	2.00	2.00	2.00
พฤติกรรมดี	ระดับสูง	4.00	3.00	2.00	1.00
พฤติกรรมดี	ระดับสูง	2.00	3.00	2.00	1.00
พฤติกรรมไ	ระดับปานก	2.00	3.00	1.00	1.00
พฤติกรรมไ	ระดับสูง	3.00	3.00	2.00	2.00
พฤติกรรมไ	ระดับปานก	2.00	1.00	1.00	1.00
พฤติกรรมดี	ระดับปานก	4.00	3.00	2.00	2.00
พฤติกรรมดี	ระดับปานก	2.00	3.00	2.00	1.00
พฤติกรรมดี	ระดับสูง	1.00	3.00	2.00	1.00
พฤติกรรมดี	ระดับปานก	3.00	3.00	2.00	2.00
พฤติกรรมดี	ระดับปานก	1.00	3.00	2.00	1.00
พฤติกรรมดี	ระดับปานก	1.00	3.00	2.00	1.00

พฤติกรรมดี	ระดับปานก	3.00	1.00	1.00	1.00
พฤติกรรมดี	ระดับปานก	4.00	2.00	2.00	1.00
พฤติกรรมดี	ระดับปานก	5.00	2.00	2.00	2.00
พฤติกรรมไ	ระดับสูง	2.00	1.00	1.00	1.00
พฤติกรรมดี	ระดับสูง	4.00	3.00	2.00	1.00
พฤติกรรมไ	ระดับปานก	2.00	1.00	1.00	1.00
พฤติกรรมดี	ระดับปานก	4.00	2.00	2.00	2.00
พฤติกรรมดี	ระดับปานก	2.00	2.00	2.00	2.00
พฤติกรรมดี	ระดับปานก	4.00	3.00	4.00	#NULL!
พฤติกรรมดี	ระดับปานก	2.00	1.00	1.00	1.00
พฤติกรรมดี	ระดับปานก	3.00	3.00	2.00	2.00
พฤติกรรมไ	ระดับปานก	4.00	3.00	2.00	1.00
พฤติกรรมไ	ระดับสูง	3.00	1.00	1.00	1.00
พฤติกรรมดี	ระดับสูง	4.00	3.00	2.00	2.00
พฤติกรรมดี	ระดับสูง	3.00	3.00	2.00	2.00
พฤติกรรมดี	ระดับปานก	1.00	1.00	1.00	1.00
พฤติกรรมดี	ระดับสูง	1.00	1.00	1.00	1.00
พฤติกรรมดี	ระดับปานก	5.00	2.00	2.00	2.00
พฤติกรรมดี	ระดับปานก	4.00	3.00	2.00	2.00
พฤติกรรมไ	ระดับปานก	3.00	3.00	1.00	1.00
พฤติกรรมดี	ระดับสูง	3.00	1.00	2.00	1.00
พฤติกรรมดี	ระดับสูง	1.00	1.00	2.00	2.00
พฤติกรรมดี	ระดับต่ำ	3.00	2.00	2.00	1.00
พฤติกรรมดี	ระดับสูง	4.00	1.00	1.00	1.00
พฤติกรรมไ	ระดับปานก	5.00	2.00	4.00	4.00
พฤติกรรมดี	ระดับปานก	3.00	2.00	2.00	2.00
พฤติกรรมดี	ระดับปานก	2.00	1.00	1.00	1.00
พฤติกรรมดี	ระดับปานก	3.00	2.00	2.00	2.00
พฤติกรรมดี	ระดับปานก	2.00	3.00	2.00	1.00
พฤติกรรมดี	ระดับปานก	2.00	3.00	1.00	1.00
พฤติกรรมดี	ระดับสูง	2.00	1.00	2.00	2.00
พฤติกรรมดี	ระดับสูง	4.00	2.00	4.00	2.00
พฤติกรรมดี	ระดับปานก	3.00	2.00	2.00	1.00
พฤติกรรมไ	ระดับปานก	2.00	1.00	2.00	1.00
พฤติกรรมดี	ระดับสูง	2.00	3.00	2.00	1.00
พฤติกรรมไ	ระดับสูง	2.00	1.00	1.00	1.00
พฤติกรรมดี	ระดับสูง	4.00	2.00	#NULL!	#NULL!
พฤติกรรมดี	ระดับปานก	2.00	2.00	2.00	2.00
พฤติกรรมดี	ระดับสูง	4.00	3.00	3.00	2.00
พฤติกรรมดี	ระดับสูง	2.00	1.00	1.00	1.00
พฤติกรรมดี	ระดับปานก	3.00	3.00	2.00	2.00
พฤติกรรมไ	ระดับปานก	4.00	3.00	2.00	2.00
พฤติกรรมไ	ระดับปานก	3.00	1.00	2.00	2.00
พฤติกรรมดี	ระดับสูง	4.00	3.00	2.00	2.00
พฤติกรรมดี	ระดับปานก	3.00	3.00	2.00	2.00
พฤติกรรมดี	ระดับปานก	1.00	1.00	#NULL!	#NULL!
พฤติกรรมดี	ระดับปานก	1.00	1.00	1.00	1.00
พฤติกรรมดี	ระดับปานก	5.00	2.00	2.00	2.00
พฤติกรรมดี	ระดับสูง	4.00	3.00	2.00	2.00
พฤติกรรมไ	ระดับสูง	3.00	3.00	1.00	1.00
พฤติกรรมดี	ระดับปานก	3.00	1.00	2.00	1.00

พฤติกรรมดี	ระดับสูง	1.00	1.00	2.00	2.00
พฤติกรรมดี	ระดับปานก	3.00	2.00	2.00	1.00
พฤติกรรมดี	ระดับปานก	4.00	1.00	1.00	1.00
พฤติกรรมไ	ระดับสูง	5.00	2.00	4.00	3.00
พฤติกรรมดี	ระดับปานก	3.00	2.00	2.00	2.00
พฤติกรรมไ	ระดับปานก	3.00	1.00	1.00	1.00
พฤติกรรมดี	ระดับปานก	2.00	3.00	2.00	2.00
พฤติกรรมดี	ระดับปานก	3.00	2.00	2.00	2.00
พฤติกรรมดี	ระดับสูง	4.00	3.00	1.00	1.00
พฤติกรรมดี	ระดับสูง	2.00	3.00	2.00	1.00
พฤติกรรมไ	ระดับปานก	2.00	1.00	#NULL!	#NULL!
พฤติกรรมไ	ระดับปานก	2.00	3.00	2.00	1.00
พฤติกรรมดี	ระดับปานก	2.00	1.00	1.00	1.00
พฤติกรรมดี	ระดับปานก	4.00	2.00	#NULL!	#NULL!
พฤติกรรมดี	ระดับปานก	2.00	2.00	2.00	2.00
พฤติกรรมดี	ระดับปานก	4.00	3.00	3.00	2.00
พฤติกรรมดี	ระดับสูง	2.00	1.00	1.00	1.00
พฤติกรรมดี	ระดับสูง	3.00	3.00	2.00	2.00
พฤติกรรมไ	ระดับสูง	4.00	3.00	#NULL!	#NULL!
พฤติกรรมไ	ระดับปานก	3.00	1.00	2.00	2.00
พฤติกรรมดี	ระดับสูง	4.00	3.00	2.00	2.00
พฤติกรรมดี	ระดับสูง	3.00	3.00	2.00	2.00
พฤติกรรมดี	ระดับปานก	1.00	1.00	1.00	1.00
พฤติกรรมดี	ระดับปานก	1.00	1.00	1.00	1.00
พฤติกรรมดี	ระดับปานก	4.00	3.00	1.00	2.00
พฤติกรรมไ	ระดับปานก	3.00	1.00	1.00	1.00
พฤติกรรมดี	ระดับสูง	3.00	2.00	3.00	2.00
พฤติกรรมไ	ระดับสูง	5.00	3.00	2.00	2.00
พฤติกรรมไ	ระดับสูง	3.00	2.00	3.00	2.00
พฤติกรรมไ	ระดับสูง	4.00	3.00	1.00	1.00
พฤติกรรมดี	ระดับปานก	3.00	3.00	3.00	3.00
พฤติกรรมไ	ระดับสูง	3.00	1.00	1.00	1.00
พฤติกรรมไ	ระดับสูง	3.00	2.00	2.00	2.00
พฤติกรรมไ	ระดับปานก	5.00	3.00	1.00	1.00
พฤติกรรมดี	ระดับปานก	3.00	3.00	1.00	2.00
พฤติกรรมไ	ระดับปานก	5.00	3.00	1.00	1.00
พฤติกรรมดี	ระดับปานก	1.00	1.00	1.00	1.00
พฤติกรรมดี	ระดับสูง	2.00	1.00	1.00	1.00
พฤติกรรมดี	ระดับสูง	2.00	3.00	2.00	2.00
พฤติกรรมดี	ระดับสูง	2.00	2.00	2.00	2.00
พฤติกรรมดี	ระดับปานก	1.00	1.00	1.00	1.00
พฤติกรรมไ	ระดับปานก	1.00	1.00	1.00	1.00
พฤติกรรมดี	ระดับปานก	5.00	2.00	4.00	3.00
พฤติกรรมดี	ระดับปานก	5.00	2.00	1.00	2.00
พฤติกรรมไ	ระดับสูง	2.00	3.00	1.00	1.00
พฤติกรรมไ	ระดับสูง	4.00	3.00	1.00	2.00
พฤติกรรมดี	ระดับปานก	1.00	3.00	2.00	2.00
พฤติกรรมไ	ระดับปานก	1.00	1.00	1.00	1.00
พฤติกรรมดี	ระดับสูง	4.00	3.00	2.00	2.00
พฤติกรรมไ	ระดับปานก	2.00	3.00	1.00	1.00
พฤติกรรมดี	ระดับปานก	4.00	1.00	2.00	2.00

พฤติกรรมไ	ระดับปานก	1.00	1.00	1.00	1.00
พฤติกรรมไ	ระดับปานก	2.00	3.00	2.00	2.00
พฤติกรรมไ	ระดับปานก	2.00	3.00	3.00	2.00
พฤติกรรมดี	ระดับปานก	4.00	3.00	1.00	1.00
พฤติกรรมดี	ระดับสูง	2.00	3.00	2.00	1.00
พฤติกรรมดี	ระดับปานก	2.00	1.00	1.00	1.00
พฤติกรรมไ	ระดับสูง	1.00	1.00	#NULL!	#NULL!
พฤติกรรมไ	ระดับสูง	2.00	1.00	1.00	1.00
พฤติกรรมดี	ระดับปานก	3.00	3.00	2.00	1.00
พฤติกรรมดี	ระดับปานก	3.00	3.00	2.00	1.00
พฤติกรรมไ	ระดับปานก	2.00	1.00	1.00	1.00
พฤติกรรมไ	ระดับสูง	3.00	3.00	2.00	2.00
พฤติกรรมดี	ระดับปานก	2.00	1.00	2.00	2.00
พฤติกรรมดี	ระดับสูง	2.00	2.00	2.00	2.00
พฤติกรรมดี	ระดับสูง	4.00	3.00	2.00	1.00
พฤติกรรมดี	ระดับสูง	4.00	3.00	#NULL!	#NULL!
พฤติกรรมดี	ระดับปานก	3.00	2.00	3.00	2.00
พฤติกรรมไ	ระดับสูง	2.00	1.00	2.00	1.00
พฤติกรรมไ	ระดับสูง	5.00	3.00	2.00	2.00
พฤติกรรมดี	ระดับปานก	1.00	1.00	2.00	1.00
พฤติกรรมดี	ระดับปานก	2.00	1.00	1.00	1.00
พฤติกรรมดี	ระดับสูง	3.00	3.00	4.00	4.00
พฤติกรรมดี	ระดับสูง	5.00	3.00	2.00	2.00
พฤติกรรมไ	ระดับสูง	4.00	3.00	1.00	1.00
พฤติกรรมไ	ระดับสูง	3.00	3.00	2.00	1.00
พฤติกรรมดี	ระดับปานก	4.00	2.00	3.00	2.00
พฤติกรรมไ	ระดับสูง	3.00	3.00	2.00	1.00
พฤติกรรมดี	ระดับปานก	3.00	3.00	2.00	2.00
พฤติกรรมไ	ระดับสูง	5.00	3.00	1.00	1.00
พฤติกรรมดี	ระดับสูง	4.00	3.00	1.00	1.00
พฤติกรรมไ	ระดับสูง	3.00	3.00	3.00	2.00
พฤติกรรมไ	ระดับปานก	5.00	3.00	1.00	1.00
พฤติกรรมไ	ระดับสูง	2.00	1.00	4.00	1.00
พฤติกรรมดี	ระดับปานก	4.00	3.00	2.00	2.00
พฤติกรรมไ	ระดับปานก	2.00	3.00	1.00	1.00
พฤติกรรมดี	ระดับสูง	3.00	3.00	4.00	2.00
พฤติกรรมไ	ระดับสูง	2.00	3.00	1.00	1.00
พฤติกรรมไ	ระดับสูง	2.00	1.00	1.00	1.00
พฤติกรรมดี	ระดับสูง	2.00	3.00	1.00	1.00
พฤติกรรมดี	ระดับปานก	4.00	3.00	2.00	2.00
พฤติกรรมไ	ระดับปานก	2.00	3.00	1.00	1.00
พฤติกรรมดี	ระดับสูง	4.00	1.00	2.00	2.00
พฤติกรรมไ	ระดับปานก	2.00	1.00	1.00	1.00
พฤติกรรมไ	ระดับปานก	2.00	3.00	2.00	2.00
พฤติกรรมไ	ระดับสูง	2.00	3.00	3.00	2.00
พฤติกรรมดี	ระดับปานก	4.00	3.00	1.00	1.00
พฤติกรรมดี	ระดับสูง	4.00	2.00	3.00	2.00
พฤติกรรมดี	ระดับสูง	2.00	2.00	2.00	2.00
พฤติกรรมดี	ระดับสูง	1.00	3.00	1.00	1.00
